# Reboot: a straightforward approach to identify genes and splicing isoforms associated with cancer patient prognosis

**DOI:** 10.1093/narcan/zcab024

**Published:** 2021-06-15

**Authors:** Felipe R C dos Santos, Gabriela D A Guardia, Filipe F dos Santos, Daniel T Ohara, Pedro A F Galante

**Affiliations:** Centro de Oncologia Molecular, Hospital Sirio-Libanes, Sao Paulo, SP 01308-060, Brazil; Programa Interunidades em Bioinformatica, Universidade de São Paulo, Sao Paulo, SP 05508-090, Brazil; Centro de Oncologia Molecular, Hospital Sirio-Libanes, Sao Paulo, SP 01308-060, Brazil; Centro de Oncologia Molecular, Hospital Sirio-Libanes, Sao Paulo, SP 01308-060, Brazil; Departamento de Bioquimica, Universidade de Sao Paulo, SP 05508-000, Brazil; Centro de Oncologia Molecular, Hospital Sirio-Libanes, Sao Paulo, SP 01308-060, Brazil; Centro de Oncologia Molecular, Hospital Sirio-Libanes, Sao Paulo, SP 01308-060, Brazil

## Abstract

Nowadays, the massive amount of data generated by modern sequencing technologies provides an unprecedented opportunity to find genes associated with cancer patient prognosis, connecting basic and translational research. However, treating high dimensionality of gene expression data and integrating it with clinical variables are major challenges to perform these analyses. Here, we present Reboot, an integrative approach to find and validate genes and transcripts (splicing isoforms) associated with cancer patient prognosis from high dimensional expression datasets. Reboot innovates by using a multivariate strategy with penalized Cox regression (LASSO method) combined with a bootstrap approach, in addition to statistical tests and plots to support the findings. Applying Reboot on data from 154 glioblastoma patients, we identified a three-gene signature (IKBIP, OSMR, PODNL1) whose increased derived risk score was significantly associated with worse patients’ prognosis. Similarly, Reboot was able to find a seven-splicing isoforms signature related to worse overall survival in 177 pancreatic adenocarcinoma patients with elevated risk scores after uni- and multivariate analyses. In summary, Reboot is an efficient, intuitive and straightforward way of finding genes or splicing isoforms signatures relevant to patient prognosis, which can democratize this kind of analysis and shed light on still under-investigated cancer-related genes and splicing isoforms.

## INTRODUCTION

The improvement of prognostic prediction and the identification of potential biomarkers and therapeutic targets are major interests in oncology (1,2). To achieve these goals, large consortiums have been created, generated and made available an unprecedented amount of data, which includes clinical (e.g. survival time, tumor recurrence and treatment) and molecular information (e.g. mutation and gene expression profiles) from cancer patients (3,4). In particular, a number of studies have shown that alterations in gene expression (5,6) and in splicing profiles (7,8) are pivotal to tumorigenesis. Once these alterations are established, researchers are often interested in pinpointing genes or splicing isoforms impacting the prognosis of patients, which are naturally suitable therapeutic targets or biomarkers.

In this scenario, Cox regression models are the gold standard methodology to find genes or splicing isoforms associated with cancer patient survival. Most commonly, analyses performed on datasets with a large number of covariates are either based on simple univariate regression models or their derived forms for variable selection (9). However, multivariate regression models are more suitable for multifactorial phenomena due to their ability to provide synergistic and antagonistic interrelation for explanatory variables (10,11), a typical condition when dealing with complex diseases like cancer.

Nevertheless, such traditional models are susceptible to data idiosyncrasy. For instance, considering the high number of covariates usually present in gene expression data, it may be a challenging task to build Cox models accounting for all of them with high accuracy (12). In a first attempt to overcome this limitation, some methods such as the Least Absolute Shrinkage and Selection Operator (LASSO) have been implemented to simultaneously estimate coefficients and treat data high dimensionality using variable selection techniques (13). Nonetheless, these implementations ordinarily exhibit poor performance for large datasets, e.g. gene expression data generated by RNA sequencing methodologies, leading to struggling in the algorithms’ convergence steps. Additionally, high collinearity and low variance of gene expression may result in incorrect estimations of the individual contributions of genes and even the identification of redundant variables in a derived model (14). Moreover, finding and testing the prognostic value or biomarker potential of a gene set is a demanding task for researchers and clinicians without extensive bioinformatics training (15). To aid, several computational tools have been created, but still with flaws inherent to them, namely (i) finding genes that are suitable for accomplishing the user’s goals; (ii) difficulties to determine the exact data type and even the appropriate method for user’s experiments; (iii) impossibility to customize analyses and inputs, among others (16). An easy-to-use command-line tool is routinely a worthy and more powerful option.

Here, we present Reboot, an algorithm to identify and validate gene or transcript (splicing isoform) signatures highly associated with patient prognosis from high dimensional datasets. Reboot innovates by using a multivariate strategy with penalized Cox regression—LASSO method combined with a bootstrap approach. Our algorithm deals with collinear variables inherent in gene expression data by preventing redundancies and incorrect estimates, thereby removing genes with low impact on survival, i.e. low expression variance among individuals. Reboot provides complementary statistical tests to bolster gene signatures associated with patient survival or any other endpoint chosen. Furthermore, Reboot generates supporting figures, such as Kaplan–Meier curves and forest plots to facilitate the interpretation of survival outcomes. Finally, Reboot seeks not only genes but also splicing isoforms (transcripts) associated with patient prognosis, successfully managing to cope with the escalation of the analysis and incorporating a deeper level of transcriptomic data interpretation to survival analyses in a practical way.

## MATERIALS AND METHODS

### Usage and performance

Expression and clinical data from TCGA (https://portal.gdc.cancer.gov/) were obtained from individuals that presented only a single primary glioblastoma tumor by an *in-house* R script (toyfordocker.R found in https://galantelab.github.io/reboot). Exclusively for this analysis, gene expression values were obtained (in FPKM) from pre-processed TCGA datasets. The same 50 randomly picked genes were used in all assays with exception of concomitant variation of both group size and number of iterations, in which 500 genes were randomly picked. For time comparisons, laptop and server specifications are: CPU: Intel(R) Xeon(R) Silver 4114, 2.20 GHz, 128 GB of RAM; and CPU: Intel(R) Core (TM) i7-8550U 1.80 GHz, 16 GB of RAM, respectively. All-time assays were computed with the parameter ‘M’ and all others were set default unless otherwise stated. All linear regressions (Pearson’s correlation) and plots were generated in R.

### Gene and transcript expression profiles

We used Kallisto (17) with GENCODE (https://www.gencodegenes.org; v29, as reference to the human transcriptome) to obtain the transcript expression profiles and (with a further step using tximport (18). This approach was used in normal (708 esophagus samples from The Genotype-Tissue Expression [GTEx]) and in cancer samples from The Cancer Genome Atlas (154 samples from glioblastoma, 248 samples from Low grade glioma grade II, 180 samples from triple- negative breast cancer (classified according to (19)) 872 samples non-triple negative breast cancer, 82 samples from esophageal adenocarcinoma and 177 samples from pancreatic adenocarcinoma. To 167 pancreatic samples, we used Kallisto’s result available through the UCSC Xena portal (toil.xenahubs.net/).

### Differential gene expression

Differential gene expression of GBM versus LGG-II, NTN-BRCA versus TN-BRCA, PAAD versus (normal) pancreas, ESCA × (normal) esophagus samples from TCGA (cancer samples) and GTEx (normal samples) was performed using DESeq2 (20), and we considered as up-regulated only genes presenting a |log2FoldChange| ≥ 2 and false discovery rate (FDR) adjusted *P*-value < 0.05.

### Differential transcript expression

All analysis of differential transcripts usage was performed using SUPPA2 (Trincado *et al.* 2018; version 2.3). We considered as significant only transcripts presenting a |ΔPSI| ≥ 0.1 and FDR adjusted *P*-value ≤ 0.05.

### Functional analyses

For Gene Ontology (GO) enrichment analysis, we used ShinyGO (21) and REVIGO (22) web tools. ShinyGO was also used to evaluate cancer hallmarks from the Molecular Signatures Database - MSigDB (www.gsea-msigdb.org/gsea/msigdb/) and KEGG pathways (www.genome.jp/kegg/). Only functional terms with an FDR  <  0.01 (hypergeometric test) were considered relevant. Protein–protein interaction analysis was performed in Cytoscape (23) using the STRING database (24).

### 3D structure prediction

MCFL2-201, MCF2L-232, HTT-201 and HTT-202 transcript nucleotide sequences were submitted to ORFfinder (25) with default values. The longest positive open reading frames (ORFs) were then submitted to Pfam (26). Finally, the amino acid sequences of all transcripts were submitted to Phyre2 (27); version 2.0 for 3D structure prediction in ‘intensive’ mode.

### Drugs and target prediction

To evaluate the clinical relevance of genes and transcripts, we obtained information from three databases: (i) Genes considered either successful, patented, under clinical trial or research drug targets according to the Therapeutic Target Database (TTD) (28); (ii) Drugs and targets information from the Clinical Interpretation of Variants in Cancer (CIViC) (29); and (iii) Genes directly linked to clinical action from the TARGET database (https://ocg.cancer.gov/programs/target). Data from these three databases were then overlapped with the lists of genes and transcripts present in the signatures generated by Reboot.

### Comparison of Reboot against similar tools

We compared Reboot against other similar tools available in the literature (HDMAC) (30), Biospear (31), BhGLM (32) and KM-Plotter (33). Twelve features were evaluated in the comparison: (i) ‘Penalized cox regression’: employment of an algorithm that uses LASSO, Ridge or elastic net regression models; (ii) ‘Bootstrap’: implementation of bootstrap resampling of features; (iii) ‘Web interface’: possibility of running the tool totally or partially in a web interface; (iv) ‘Detailed documentation’: availability of extensive documentation, including usage examples and commands, explanation about main parameters and installation guide; (v) ‘High dimensional data’: computational and methodological support to the analyses of >1000 genes; (vi) ‘Evaluation of clinical parameters’: support to the analysis of clinical data in a multivariate way; (vii) ‘Pre-filtering’: pre-checking steps to evaluate the viability of input variables, e.g. filters of variance and Schoenfeld test; (viii) ‘Integrative approach’: support to both identification of molecular signatures and survival analysis based on produced signatures in an integrative way; (ix) ‘Validation’: support to computational validation of obtained molecular signatures in independent datasets; (x) ‘Graphical signature’: availability of graphical representations for signature regression parameters; (xi) ‘Graphical survival’: availability of graphical outputs in survival analyses, such as Kaplan–Meier curves, score, Hazard ratio visualization; and (xii) ‘Command line’: availability of a command line interface to facilitate the authorization of repetitive tasks (runnings) and integration to other pipelines.

### Reboot web interface

Reboot's web application is implemented in PHP (https://www.php.net) while interface visual contents are constructed using HTML and CSS. Our web application embed gene and transcript signatures generated from four TCGA tumor datasets, namely: BRCA, ESCA, GBM and PAAD.

## RESULTS

### Implementation

**Figure 1.**
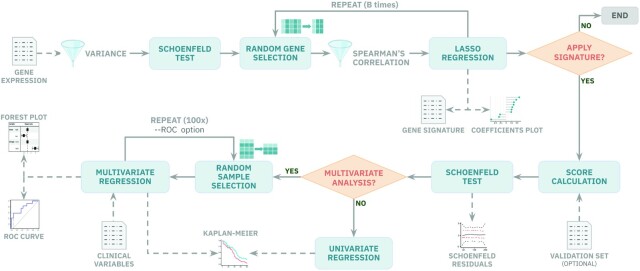
Reboot pipeline automatically integrates robust statistical tests, provides plots and allows users to control parameters straightforwardly. In module I, gene or transcript expression data are filtered for variance and Cox proportional hazard assumptions. Then, genes go through a random bootstrap resampling selection for LASSO regression and signature generation in case they are not significantly correlated. In module II, a signature-based score is created and applied in survival analysis. Users are able to perform multivariate analyses, with or without bootstrap resampling and ROC curves, if clinical data are available. Plots are automatically yielded to the users.

Reboot comprises two major modules: regression and survival (Figure 1). These two modules were designed to work independently, allowing users to identify genetic signatures using the ‘regression’ module, and to test the significance of these signatures in prognosis using the ‘survival’ module, possibly with additional validation datasets. Moreover, we also provide a ‘complete’ mode option which enables the integrated execution of the two modules in case the same dataset is intended to be used in both analyses.

The Reboot ‘regression’ module is an easy-to-run step, which encapsulates statistical models to identify genes or splicing isoforms (transcripts) signatures. In brief, this module starts by checking if the provided dataset has a minimum of 20 variables to perform bootstrap iterations, otherwise a single regression is performed. A minimum of 10 samples is required, given that every iteration performs a 10-fold cross-validation log likelihood task meant for an optimal choice of the LASSO coefficient. Additionally, data attributes with variance lower than a user-defined or default cutoff are removed. This step also checks for minimum variability of endpoint statuses, therefore datasets with 10 or more samples that are not variable enough are hindered. Next, a Schoenfeld test (34) is applied in a univariate way for each remaining attribute in the dataset using the packages ‘survival’ (35) and ‘survminer’ (Kassambara *et al.*, 2019). Every attribute that fails this proportional hazard assumption test is automatically removed from the analysis. After that, a Spearman’s correlation filter is applied to every iteration of the bootstrap process based on the settable fraction of pairs with a correlation coefficient >0.8 and a *P*-value < 0.05. Lastly, also during the bootstrap process, random samplings of attributes to be evaluated in a multivariate analysis are executed. Regression itself is performed using a Least Absolute Shrinkage and Selection Operator (LASSO) algorithm from the R packages ‘penalized’ (36) and ‘survival’.

The next step in Reboot is the ‘survival’ module, which is also easily executable. It receives and tests a gene/transcript signature produced in the previous (regression) module. In this step, the Reboot algorithm first produces and assigns a score for each sample based on the gene/transcript signature coefficients obtained from the ‘regression’ module and their corresponding expression values using the following formula: $\mathop \sum \limits_{n\; = \;1}^n C*\;E$, where ‘*C*’ is the coefficient and ‘*E*’ is the expression value. Next, the Schoenfeld test is applied to verify whether the score addresses the Cox model assumptions. Based on the median value (default) of the obtained scores, all individuals being tested are stratified into two groups, ‘low’ or ‘high’ score. The log-rank test is then performed in order to assess the relevance of the observed differences and to evaluate the relevance of the signature score as a prognostic factor for a given event, such as overall survival, progression- or recurrence-free survival. Finally, a Kaplan–Meier survival curve is generated using the R package ‘survcomp’ (37). Of note, Reboot offers a multivariate option that allows extension of the survival model with additional clinical variables, e.g. therapy, age and gender. If this option is chosen, after applying the Schoenfeld test to all variables, multiple univariate analyses are performed and only those under a minimal threshold (see Materials and Methods section) are selected for the final multivariate model and illustrated in a forest plot using the R package ‘forestmodel’.

Moreover, Reboot has an alternative to the use of the median value as a cutoff to stratify patients into ‘low’ and ‘high’ groups based on gene or transcript expression: a receiver operator characteristic (ROC) curve with the nearest neighbor estimate (NNE) method and the Youden statistics (38). In this case, a patient-oriented bootstrap resampling strategy is performed using the R package ‘sjstats’ (https://CRAN.R-project.org/package = sjstats). In order to derive highly confident and robust results, additional filters are applied such as null data removal, the minimum number of co-variables available and proportionality requirements (39). As a consequence, these filters ensure that the final dataset is composed of at least 70% of patients’ data present in the original one. Additionally, the final dataset also has a minimum of two co-variables to be tested with the score, whose less abundant category’s frequency is not <20%. After 100 cycles, the relevance frequency of each co-variable with the event is calculated and only the ones with at least 25% are plotted.

### Usage and performance

Reboot was designed to be easy-to-install and of straightforward use. To generate a genetic signature, Reboot only requires a matrix of survival data along with gene or transcript expression values as input in the form of a ‘.tsv’ file (https://galantelab.github.io/reboot for further details). In order to test a genetic signature, Reboot requires in addition to survival and expression data, a signature matrix with the previously produced regression coefficients (‘.tsv’ file automatically incorporated in ‘Complete’ mode), which may be manually filtered down for further analyses with a more stringent list of coefficients to avoid false positives (Supplementary Figure S1). In case a multivariate survival analysis is requested by the user, an additional file containing clinical variables to be tested should also be provided (‘.tsv’ file). Supplementary Figure S2 shows examples of inputs to Reboot.

As output, Reboot generates two main textual results (‘.tsv’ files): (i) a list of genes or transcripts that comprise the genetic signature and their corresponding regression coefficients, which explain the contribution of each gene or transcript to the signature, and (ii) the survival impact of the signature score, including hazard ratio estimates, log-rank *P*-values, number of samples and median survival per group, among others. In addition, multiple plots are produced: (i) a lollipop plot, displaying the ten most significant gene or transcript coefficients comprising the signature; (ii) a coefficient histogram, displaying the distribution of all coefficients in the signature; (iii) a proportional hazard assumptions plot (Schoenfeld test); and (iv) a Kaplan–Meier plot (Figure 1). In case the multivariate option is chosen, Reboot returns all files and figures generated in the univariate analysis plus an additional ‘.tsv’ file containing the survival results of the signature score along with all other clinical variables, also visible as a forest plot. Furthermore, if the score stratification is performed with the ROC method, the curve is also available. Finally, a histogram of co-variable frequencies is also provided in case the multivariate option was done with bootstrap resampling.

**Figure 2.**
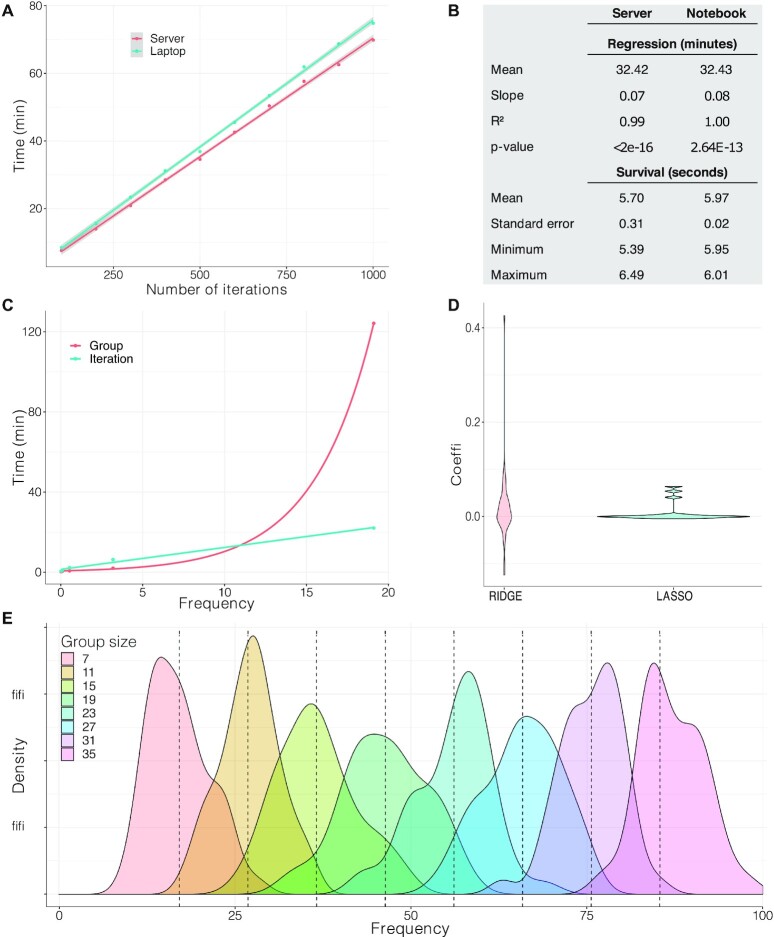
Reboot is computationally efficient, working well in laptops and servers. (**A**) Evaluation of total execution time for a complete run of Reboot in a server and a laptop according to the number of iterations. Number of iterations varied from 100 to 1000 in steps of 100, keeping group size 20 for 145 patients and 50 randomly chosen genes. Survival was performed in multivariate mode. (**B**) Table with extracted parameters obtained in (A). (**C**) Time assay comparing the impact of group size and the number of iterations on execution time. Group size and the number of iterations varied from 3 to 243 in powers of 3 and from 2 to 32 in powers of 2, respectively, in both curves. Legend attribution corresponds to the variable that changed in powers of 3. (**D**) Coefficient distribution profile obtained from LASSO and Ridge algorithms. (**E**) Frequency distribution of attribute selection performed with group size variation. Theoretical average is shown in dashed lines.

In order to analyze the performance and features of Reboot, we built a toy dataset containing clinical (Supplementary Table S1) and randomly picked gene expression data (Supplementary Table S2) from the Cancer Genome Atlas (TCGA). Correlation between the number of iterations and execution time was assessed by varying the number of iterations and keeping group size and number of instances (patients) constant in two independent tests using either a server or a laptop (see Materials and Methods section for details). As expected, a linear behavior for running time was observed and server performance was slightly better than laptop’s performance. Considering Reboot modules separately, ‘regression’ massively accounts for the total running time, as expected (Figure 2A and B). Variations in group size or number of patients were also performed, generating similar results (Supplementary Figure S3).

The frequency of sampling for the analysis follows a distribution in which the expected average is given by equation (1), where ‘*G*’ is the group size, ‘*B*’ the number of iterations, and ‘*N*’ the total number of attributes.

(1) $$\begin{equation*}f = \frac{{BG}}{N}\end{equation*}$$

Since ‘*B*’ and ‘*G*’ are both directly proportional to attribute frequency, we also sought to compute time correlation of different increasing rates of group size and number of iterations with time. For this analysis, a larger group of 500 genes was randomly selected, similar to data retrieved previously (Supplementary Table S3). Both variables were increased by powers of 2 and 3 and multiplied, resulting in two curves containing points with the same frequency (Figure 2C). Group size increase showed lower time consumption for small frequency values, whereas the number of iterations remains linear, even for high values, indicating its superior efficiency for high attribute coverage (Figure 2C).

Additionally, LASSO and Ridge regressions (40) were run with a group size of 10 and 1000 iterations and distributions were built using only non-zero coefficients in order to assess the algorithm's performances (Figure 2D). As expected, the LASSO strategy used in Reboot compresses coefficients more efficiently, denoted by the highly populated regions around zero in relation to Ridge (Figure 2D).

Finally, data obtained for Supplementary Figure S3A was used to compute gene frequency, according to equation (1), by varying ‘*G*’ (Figure 2E). Mean standard deviation for all eight distributions was 4.93, contributing to a reliable uniformity of variable assessment despite the stochastic process associated with the iterative process. Therefore, the frequency of each attribute is recommended to be *N/G*. In accordance with equation (1), ‘*B*’ may be chosen in terms of equation (2).

(2) $$\begin{equation*}B = {(N/G)^2}\end{equation*}$$

Given that a free variation of ‘*B*’ performs better in terms of computational time and prevents bias, ‘*G*’ may be chosen for restricted lower values and ‘*B*’ estimated, with no restrictions.

### Benchmarking Reboot

In terms of features, Reboot was compared to other similar tools currently available in the literature (30–33) in order to evaluate the effectiveness of the steps for jointly or separately obtaining molecular signatures and validating them through survival analysis. Our tool is unique considering: (i) the availability of pre-filtering steps, which is essential in this kind of analysis of bootstrap procedure for signature extraction; (ii) integration; (iii) validation in an external cohort; and (iv) and its modularity of running. The last two features are, together, a trademark of Reboot, allowing users to not only test the generated signature score instantly but also validate it on independent datasets. Other Reboot’s functionalities are shared in a scattered way among the other tools deeply evaluated here (Supplementary Table S4).

In terms of Reboot’s algorithm, Penalized Cox regression models are available in all assessed tools except KM-Plotter (33), which has a slightly different purpose. KM-Plotter has a web page available to users, as well as HD-MAC (30). However, these two tools have their web page services as the only source to perform analyses, whereas Reboot’s web page is intended for exploration and relatively simpler analyses. This is vital, since KM-Plotter is not able to deal with high-dimensional data and HD-MAC (in our hand) frequently throws nonspecific errors when one attempts to input high dimensional data (>1000 genes). Reboot has a web based version and the command line option (recommended), which is the unique alternative for Biospear (31) and BhGLM (32).

Furthermore, the availability of many automated graphical resources in Reboot provides useful paths for quick and deeper analysis procedures. For instance, only Reboot and HD-MAC are able to evaluate clinical data in multivariate Cox regression analysis, while KM-Plotter only allows one to subset the raw dataset based on clinical parameters. Moreover, Reboot provides full detailed documentation in order to allow users to better explore features and parameters, which is similar to what is found in Biospear (31). Altogether, Reboot’s unique features greatly facilitate the identification, evaluation and validation of prognostic biomarkers in a straightforward way, while allowing the fine-tuning of computational parameters during the processing of large amounts of data.

### Using Reboot to identify genes related to prognosis in glioblastoma

To show how straightforward, useful and fast Reboot can be, we have applied it to a previously selected set of 1013 protein-coding genes up-regulated in glioblastoma (GBM) in comparison to low-grade glioma (LGG) patients (log2FoldChange ≥ 2 and FDR adjusted *P*-value < 0.05; Supplementary Table S5). Reboot was executed using the ‘regression’ module parameters ‘-G 10 -P 0.3 -V 0.01 -B 1000’ and its execution took 1.15 h in a standard server (see Materials and Methods section). As a result, we identified 255 genes associated with patients' overall survival (Supplementary Table S6).

**Figure 3.**
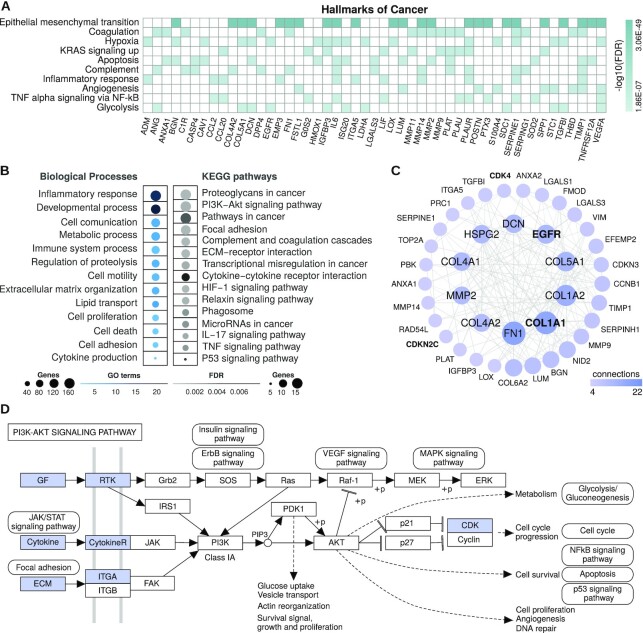
Functional enrichment analyses of genes associated with glioblastoma patients' overall survival using Reboot. (**A**) Top 10 enriched hallmarks of cancer and genes associated with at least 2 of them. (**B**) Groups of enriched GO biological processes and glioblastoma-related KEGG pathways. (**C**) Network of protein–protein interactions from STRING database with cancer driver genes highlighted in bold. (**D**) Schematic diagram of how up-regulation of 15 genes in glioblastoma may lead to activation of the PI3K-Akt signaling pathway, a summarized KEGG’s representation with gene products highlighted in blue (or in grayscale).

To determine whether these 255 genes could be important in GBM patient prognosis, we further investigated them. First, we performed functional enrichment analysis that revealed 131 genes (51.37%) associated with several hallmarks of cancer according to the Molecular Signatures Database (FDR < 0.01, hypergeometric test; Supplementary Table S7, Figure 3A). Among the top 10 enriched hallmarks, we found 49 genes linked to at least two hallmarks relevant for glioblastoma progression and invasion, including those defining epithelial–mesenchymal transition (41), encoding components of blood coagulation (42), as well as genes up-regulated in response to hypoxia (43) and/or by KRAS activation (44), among others. Genes associated with GBM patients’ survival were also enriched in a number of GO biological processes (Supplementary Table S8) and glioblastoma-related KEGG pathways (Supplementary Table S9) (FDR < 0.01, hypergeometric test; Figure 3B). GO groups include, but are not limited to, processes related to inflammatory response, cell adhesion, proliferation and motility, while the glioblastoma-related KEGG pathways with the greatest number of genes were proteoglycans/pathways in cancer, PI3K-Akt signaling pathway and focal adhesion.

We also conducted a protein-protein interaction analysis using these 255 genes, which displayed a highly connected gene network comprising four cancer driver genes according to the Cancer Gene Census (CGC) database (45); Figure 3C: COL1A1, EGFR, CDK4 and CDKN2C. Moreover, according to CGC, other six driver genes were observed in the produced signature, most of them having an oncogenic role (Supplementary Table S10). Several genes associated with GBM initiation and progression were also observed in the network, including EGFR, MMP2, HSPG2 and various members of the collagen gene family (e.g. COL1A1, COL1A2 and COL5A1), which encode components of the extracellular matrix. Of note, fibronectin (FN1) was the top enriched gene in our network. An intracranial GBM xenograft model (46) showed that expression of FN1 promotes cell proliferation and resistance to ionizing irradiation, facilitates cell invasion and enhances angiogenic tumor growth. More recently, Liao *et al.* (47) provided evidence that fibronectin silencing in gliomas is associated with disruption of the PI3K-AKT signaling pathway and subsequent inhibition of cell proliferation, as well as promotion of cell apoptosis and senescence. Accordingly, we observed 15 genes highly expressed in GBM, mostly encoding activators of the PI3K-AKT signaling pathway (Figure 3D), which is frequently activated in glioblastoma (approximately 90%; (48). Of those, we found around 30 genes (e.g. EGFR, CDK4, RUNX1, IL6, RRM2 and VEGFA) with enough support to be considered clinically relevant from either TARGET, TTD or CIViC databases. Furthermore, other four genes are patented targets for drugs and 27 genes are under clinical trials studies according to the TTD database (Supplementary Table S11). Altogether, these 255 candidates contain many genes already reported as relevant to GBM origin, maintenance and progression, suggesting that Reboot consistently selected a gene list potentially related to prognosis in glioblastoma.

### Using Reboot to identify a minimal gene signature relevant to GBM survival

**Figure 4.**
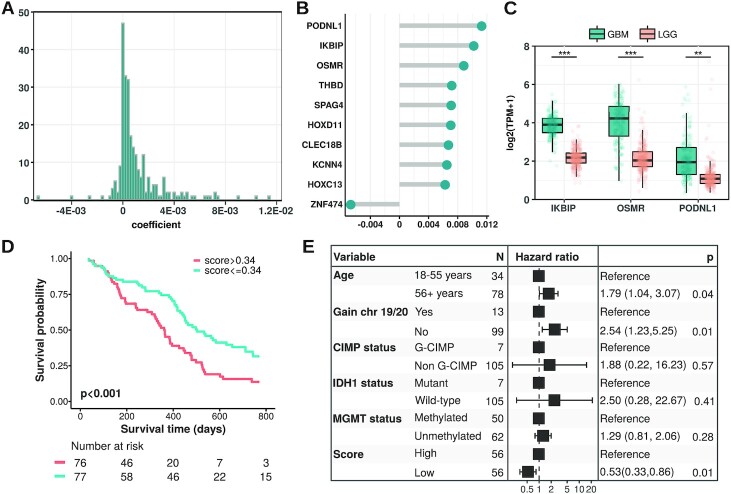
Reboot's application on the glioblastoma dataset. (**A**) Histogram displaying the distribution of all gene coefficients obtained using Reboot ‘regression’. (**B**) Top 10 genes identified as relevant for the prognosis of GBM patients. (**C**) Boxplots displaying the expression values of a 3-gene signature identified in GBM patients with Reboot (Wilcoxon test; **P<0.01, ***P<0.001). (**D**) Kaplan–Meier curve based on the 3-gene signature score identified in GBM patients with Reboot. (**E**) Forest plot of a multivariate model including the 3-gene signature score adjusted for clinical parameters relevant to prognosis in glioblastoma.

Next, we sought to determine the minimum gene set with the highest regression coefficients that are capable of explaining differences in overall survival (OS) of GBM patients using Reboot ‘survival’ module in multivariate mode (run in docker with parameters ‘-M -C’; execution time ∼10 s in a standard laptop). Out of the total 255 genes associated with patients’ overall survival using Reboot (Figure 4A; Supplementary Table S6), we identified three candidates: IKBIP, OSMR and PODNL1. They are among the top 10 genes identified as the most relevant for the prognosis of GBM patients (Figure 4B) and are overexpressed in glioblastoma samples in comparison to low-grade glioma (LGG) (Figure 4C). Moreover, IKBIP, OSMR and PODNL1 combined score has a significant impact on survival of GBM patients (HR = 0.48 95% CI: [0.32–0.71], *P*-value < 0.001; Figure 4D). The median OS for patients with a high score (>0.34) was 335 days, yet for the low score group was 468 days. More importantly, the obtained risk score remained significant (HR = 0.53 95% CI: [0.33–0.86), *P*-value = 0.01, Figure 4E) even when considered together with relevant clinical parameters for GBM patients, including age at diagnosis, chromosome 19/20 co-gain, G-CIMP, *IDH1* mutation and *MGMT* methylation status.

In addition, we attempted to validate this three-gene signature in an independent cohort of 71 primary glioblastoma patients from the Chinese Glioma Genome Atlas, CGGA (47). Similarly, higher combined scores tended to be associated with worse prognosis of GBM patients (HR = 0.66 95% CI: [0.38–1.15], *P*-value = 0.14; Supplementary Figure S4). The median OS for patients with higher scores (>0.44) was 381 days versus 550 days for the low score group. Although we observed a clear separation between the higher and lower score groups in the CGGA cohort, the lack of statistical support might be explained by the smaller CGGA cohort size and sequencing depth (71 samples, ∼22.5 million *reads* on average) compared to TCGA (154 samples, ∼64.8 million *reads* on average). Therefore, this result indicated that Reboot efficiently selected a minimal gene signature whose high expression is associated with worse GBM prognosis.

### Finding alternative splicing isoforms signature relevant to pancreatic adenocarcinoma patients' prognosis with Reboot

**Figure 5.**
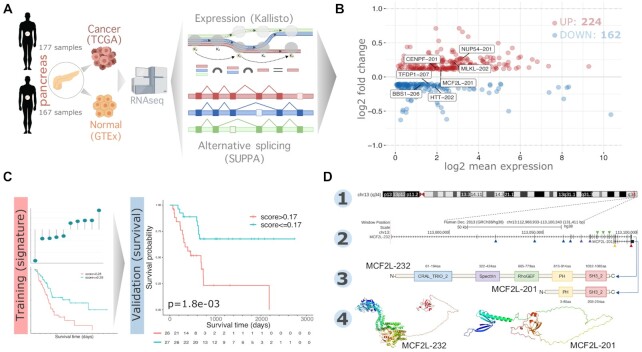
Reboot selects alternative splicing isoforms associated with pancreatic adenocarcinoma tumorigenesis and its patient’s prognosis. (**A**) Selection of alternative splicing isoform (ASI) based on transcript expression data from healthy and tumoral pancreas. (**B**) MA plot showing the mean expression (in TPM) and ΔPSI (percent spliced in) values of all ASI. Highlighted ASIs compose the seven-transcripts signature generated with Reboot. (**C**) ASI data were split into training (70%) and validation (30%) set to find a transcript signature in survival analysis. Kaplan–Meiers made by Reboot when using both the training (HR: 0.4428 [0.2719–0.7211]; *P* = 8e-04) and the validation dataset (HR: 0.2791 [0.1191–0.6541]; *P* = 0.0018) showed a worse survival outcome for patients with higher scores (above median value). Follow-up time (days) is shown in the bottom for each group. (**D**) MCF2L mapping on the reference genome (1). Canonical (longer: MCF2L-232) and ASI (shorter: MCF2L-201) isoforms, respectively (2). Protein domains encoded from canonical (MCF2L-232) and ASI (MCF2L-201) isoforms, respectively (3). Predicted 3D protein structure for canonical (MCF2L-232) and ASI (MCF2L-201) transcripts (4).

Next, we used Reboot to find splicing isoforms related to pancreatic adenocarcinoma (PAAD) patients’ prognosis. We chose this tumor type due to the acclaimed need for new biomarkers in pancreatic ductal adenocarcinoma (PDAC) (49). Moreover, recent studies have provided insights into the importance of alternative splicing for the tumorigenesis, clinical outcomes and identification of novel therapeutic targets in PAAD, evidencing the need for the identification of splicing isoforms relevant to prognosis in this tumor type (49–51). Using SUPPA2 tool (see Materials and Methods section), we found a complete set of alternative splicing isoforms (ASI) between pancreatic adenocarcinomas (PAAD) and healthy pancreatic samples, which fed the Reboot’s algorithm to perform the signature (module I) and the survival (module II) analyses (Figure 5A).

We found 386 significant alternative splicing isoforms, of which 224 and 162 were up-regulated and down-regulated, respectively, in PAAD versus healthy pancreas tissue (Figure 5B). To prove the robustness of Reboot in candidate selection, we randomly split the initial ASI data into training (70%) and validation (30%) sets (Figure 5C). When applying the ‘regression’ module on the training dataset (parameters: -B 100 -G 10 -P 0.3 -V 0.036 -F FALSE; execution time of 4.71 minutes in a standard laptop), a signature with 62 transcripts emerged (Supplementary Table S12 and Supplementary Figure S5). Of those, we found isoforms of three genes with clinical relevance: FCGR2A, RB1 and NAPRT, based on data from TARGET, TTD and CIViC databases (Supplementary Table S11). After setting a cutoff of 0.035 to coefficients, Reboot found a minimal signature of seven transcripts presenting significant survival results: CENPF-201, MLKL-202, NUP54-201, MCF2L-201, TFDP1-207, BBS1-206 and HTT-202 (Figure 5C and Supplementary Figure S6; Supplementary Table S12).

When testing the signature with module II (survival) of Reboot on the validation dataset (53 patients; parameters: -M TRUE -R FALSE -F FALSE; execution time ∼5 s in a standard laptop), we found that patients with higher scores (values above the median) had worse overall survival (HR: 0.2791 [0.1191–0.6541]; *P*-value = 0.0018; Figure 5C and Supplementary Figure S6C). The median OS for patients in the high score group (>0.17) was 684 days, whereas this value could not be calculated in the low score group since less than half of the patients died. Furthermore, this result remained statistically significant after the multivariate analysis, accounting for relevant clinical variables such as age, gender, race, tumor node metastasis (TNM) classification (52), histology and grade (HR: 0.3806 [0.1569–0.923]; *P*-value = 0.0326; Supplementary Figure S6D). Additionally, the same results were observed when applying the score on the training dataset, as expected (Supplementary Figure S6A and B), where the median OS for patients in the high score group (>0.28) was 517 days versus 1332 days for the low score group. Of note, other endpoints such as disease-specific (DSS), progression-free (PFI) or disease-free interval (DFI) may be used instead in order to better fit the data and meet survival requirements (53).

Further investigation was performed for transcripts with major contributions to the genetic score (Supplementary Figure S6). MCF2L-201, which had a significant positive score, lacks three protein domains (RhoGEF, Spectrin and CRAL_TRIO_2), which are all present in the canonical isoform MCF2L-232 (Figure 5D). Regarding the HTT gene, the HTT-202 isoform, which scored negatively in our signature, lacks the huntingtin protein region DUF3652, present in the canonical isoform HTT-201 (Supplementary Figure S7). Taken together, these results demonstrate that Reboot is effective not only to identify relevant genes but also splicing isoforms potentially related to cancer.

### Using Reboot in other tumor types

To further illustrate the usefulness of Reboot, we also analyzed two additional aggressive tumor types using RNA-Seq and clinical data from TCGA: TN BRCA and ESCA. Similar to our previous analyses, Reboot found significant molecular signatures based on gene and transcript expression to these tumors (Supplementary Figure S8). For example, ESCA signature comprises the collagen gene COL4A5 (54), the membrane gene XK (55) and the intracellular signaling messenger DGKA (56). BRCA gene signature includes ATP6V1H, a v-ATPase commonly associated with aggressiveness of different cancer types; MAF1 that regulates RNA-polymerase III and oncogenic pathways (57) and ST14 (suppression of tumorigenicity 14 gene), a protease previously described in association with BRCA (58).

In the transcripts analyses, the minimum isoform based signature for ESCA and TN-BRCA contain one (PDHA1-204) and two (SLC22A31 and CSAG3-202) transcripts, respectively. PDHA1-204, similar to its canonical counterpart PDHA1-206, maintains its functional domain, but has an extra set of 38 amino acids at the N-terminal portion. SLC22A31 belongs to the SLC family, subclassified as an organic ion transporter-related (Oat-related) subclade (59), although specific assays targeting SLC22A31 haven’t been reported, many associations with disease and promising therapeutic targets are expected for SLCs (60). The other transcript, CSAG3-202 is a non-coding version of the canonical CSAG3 (chondrosarcoma-associated gene) transcript. Surprisingly, this gene is part of a large repeated DNA structure whose expression is majorly in (normal) testis and in cancer samples (61). This gene has also been shown to bind to SIRT1, enhancing its activity and promoting tumorigenesis (62).

In terms of treatment options available or under current research for the full list of genes/transcripts in the signatures, we found some interesting potential gene targets for TN BRCA (Supplementary Table S11). CCL5 is under a phase 1 clinical trial for autoimmune diabetes, while ST14, found in the minimal gene signature analysis, is patented-recorded and whose proposed functions include an important role in breast cancer invasion and metastasis according to the TTD database. Remarkably, the majority of potential new targets for TN BRCA were found in the ‘transcripts’ analysis (Supplementary table S11). For instance, both genes NRG1 and CHEK1 have their variations in expression associated with either pre-clinical (CHEK1) or clinical (NRG1) evidence level for drugs against lung small cell carcinoma (CHEK1, prexasertib in combination with olaparib or cisplatin) and lung non-small cell carcinoma (NRG1, patritumab) according to CIViC database.

This pattern was even more evident for ESCA, where isoforms of genes MUTYH, IL15RA and MAP3K4 showed up for TTD database (Supplementary Table S11), even though there are only clinical trials (phase 2) for drugs targeting the interleukin IL15RA. However, many tumor types and non-cancer diseases are being studied under these trials such as pancreatic, bladder and lung cancers. As for MUTYH and MAP3K4, there is only evidence in literature for treatment of degenerative diseases (MUTYH) and melanoma (MAP3K4). It is important to note that these ‘transcripts’-derived predictions require experimental validation in order to directly test the influence of the expression variations found for the isoforms reported in this work.

All gene and transcript signatures derived from GBM, PAAD, BRCA and ESCA tumors are fully available at Reboot's web interface (https://www.bioinfo.mochsl.org.br/reboot/) and may be validated in user-provided datasets.

## DISCUSSION

In the past few years, advances in RNA sequencing technology have provided us an unprecedented opportunity to find novel gene signatures acting as prognostic or diagnostic biomarkers in cancer (63). Notwithstanding, treating high dimensionality of gene expression integrated with clinical variables is a major challenge when performing survival analysis, notably by researchers without extensive training in computational biology. It is therefore an urgent task to establish robust and straightforward methods capable of handling large datasets and finding these potential biomarkers. Here we describe Reboot, a user-friendly algorithm to seek, evaluate and validate genes and splicing isoforms signatures acting as prognostic or diagnostic biomarkers in cancer. Reboot is original and efficient: (i) it combines a multivariate strategy with penalized Cox regression (LASSO method) and a bootstrap approach, plus a variety of statistical tests to find genes or transcripts candidates; (ii) it is easy-to-use, well documented and of simple installation in a standard laptop; (iii) it includes effortless steps to visualize results and to facilitate data interpretation and further analyses in a convenient execution time.

As genetic analyses get wider in order to capture the complexity of intricate diseases such as cancer, a full transcriptome (genes and transcripts [splicing isoforms]) investigation becomes crucial, which significantly raises the dimension of input datasets (64). Availability of tools that manage to escalate genetic score analysis with high dimensional datasets, such as those found in gene expression data using RNA sequencing, are scarce (31,32). In this context, Reboot’s main purpose is to allow users, starting from high dimensional datasets, to find consistent genes or splicing isoforms signatures related to patient prognosis with viable performance. In addition to its command-line interface, which is the most common option for high-performance bioinformatics tools, Reboot is also available in a web interface. To enable the identification of genetic signatures, beyond all filters exclusively implemented in Reboot to automate the data pre-processing step, it uses the LASSO algorithm, a well-established method for variable selection. However, given the high collinearity and low variance of gene expression data, LASSO alone—and similar algorithms, e.g. Ridge or elastic net—may not properly converge in a confident, non-redundant set of prognostic biomarkers (30–32). To overcome this issue, Reboot associates LASSO with an authentic bootstrapping strategy, thus allowing the selection of a more reliable set of genes from a wide range of input dataset dimensions. Beyond that, to the best of our knowledge, there is no state-of-the-art pipeline that automatically integrates the identification of prognostic biomarker signatures from high dimensional data to posterior computational validation of gene and transcript (splicing isoform) signatures, including clinical data for multivariate analyses. Moreover, another Reboot’s trademark is its modularity, where users can either perform a complete analysis (from signature generation to its performance test) or a validation alone, with effortless interpretation of the findings through a number of text and graphical representations. This is outstanding, since some tools prioritize the graphical outputs of their survival analyses (31–33), while others focus on using clinical variables either as subsetting criteria (33) or for multivariate analysis, indeed (30).

We selected and tested Reboot on multiple TCGA tumor datasets. In particular, we focused our analyses on glioblastoma (GBM) and pancreatic adenocarcinoma (PAAD), two cancer types presenting a poor survival rate and limited therapeutic options for their patients (65,66). First, we identified prognostic genes in GBM associated with various processes relevant for glioblastoma tumorigenesis, progression and invasion, e.g. epithelial–mesenchymal transition, inflammatory response and cell proliferation. This list includes genes already described as related to GBM or other gliomas. For instance, MMP2 is highly expressed in gliomas and it was recently associated with stimulation of vasculogenic mimicry in glioma cells (67). HSPG2, in glioma tissues, is related to the transformation of the brain extracellular matrix into the tumour microenvironment and represents a negative prognostic factor in overall and relapse-free survival (68). In particular, the epidermal growth factor receptor (EGFR) is a primary driver of glioblastoma tumorigenesis, contributing mainly to cell proliferation and invasion (50). Moreover, this gene is a predicted successful target for drugs such as Cetuximab in colorectal cancer (69) or Lapatinib in breast cancer (70) according to the Therapeutic Target Database (TTD) (28).

Next, using the ‘survival’ module in multivariate mode, Reboot found a signature containing a minimal of three genes (IKBIP, OSMR and PODNL1) associated with GBM patients’ overall survival. Interestingly, they have emerged as prominent genes in glioblastoma’s studies. IKBIP has been described as a novel p53 target with pro-apoptotic activity, whose high expression is associated with poor prognosis in GBM (71,72). Although in our results *MGMT* methylation was not considered a significant co-variable, another study has identified the gene IKBIP as part of a signature that predicts prognosis only in GBM patients with methylated *MGMT* promoter (73). OSMR, characterized as a novel key regulator of glioblastoma tumorigenesis through EGFRvIII-STAT3 signaling, also correlates with poor prognosis in GBM patients both independently and also as part of a 4-gene signature (71,74). Interestingly, PODNL1 encodes a protein involved in extracellular matrix formation with an unclear role in GBM tumorigenesis. The latter gene up-regulation has also been correlated with the poorest survival rates in GBM patients in distinct studies (75,76). Altogether, it is clear that Reboot identified a valuable set of genes to be further and deeper investigated in GBM.

Second, we used Reboot to seek for alternative splicing isoforms associated with pancreatic adenocarcinoma (PAAD) patients’ prognosis. Indeed, we found in our signature the transcript RB1-201 and, according to the CIViC database, there is preclinical evidence of drugs (e.g. doxorubicin, gemcitabine, mitomycin and fluorouracil) to be used in PAAD patients overexpressing RB1 gene (77). Curiously, we found the yet poorly explored gene FCGR2A (associated with transcript FCGR2A-201 found in our signature) as a predicted successful target for drugs such as SM-101 in non-cancer diseases like Idiopathic thrombocytopenic purpura (78), according to the Therapeutic Target Database (TTD) (28). As for cancer, the CIViC database shows clinical evidence that breast cancer patients could be treated with trastuzumab if the missense variant H167R is present in this gene (79). Therefore, it is reasonable to think that variations in expression of FCGR2A or related isoforms may be good therapeutic targets as well in the future.

As illustrated in our analyses, a genetic score obtained from differentially expressed transcripts stratifies patients with worse and better prognosis as efficiently as from gene analyses. Interestingly, a signature score with only seven transcripts was enough to yield statistical significance in the survival analysis of PAAD patients. Among them, only three isoforms are canonical (CENPF, MLKL, NUP54). Some of these genes (e.g. CENPF, MLKL, TFDP1, MCF2L) have a known influence on cancer, while others (e.g. NUP54, BBS1 and HTT) have been superficially studied under the tumoral context. CENPF, for instance, has been related to worse outcomes and survival in several cancer types (80,81). Another outstanding example is the MLKL gene, which was shown to be up-regulated in pancreatic cancer, as we observed with Reboot, especially in tumor-invasion conditions (82). The transcription factor TFDP1 is a gene with significant somatic copy number alterations and corresponding somatic gene expression changes were observed in papillary thyroid carcinomas (83), even though whose functions remain uncovered in cancer. Additionally, it is considered a prognostic marker in liver cancer (unfavorable), stomach cancer (favorable) and renal cancer (favorable) according to The Human Protein Atlas (84). Inconsistencies in these results may have arisen from a possible divergence of the role of different isoforms from this gene. Our results indicate that an isoform (TFDP1-207, down-regulated in our analysis) other than the canonical (TFDP1-201, up-regulated in our data) is of great significance for PAAD patient prognosis, an evidence that more detailed scrutiny is required for this gene (https://www.proteinatlas.org/ENSG00000198176-TFDP1/pathology). Taken together, it is clear that transcript-centered analysis may shed light on more detailed molecular mechanisms that would not be possible in a gene-based approach.

Among the best-scored transcripts, MCF2L-201, which was found to be up-regulated in PAAD, is a compelling example. The canonical isoform of the MCF2L gene (MCF2L-232) encodes DBL from the guanine exchange factor protein family, known to directly interact and regulate important factors for cell cycle such as Cdc42 and RhoA complexes (85). It has been shown that the minimal and sufficient catalytic activity of DBL is composed of a DH and a PH domain linked in tandem (86). Although MCF2L-201 does not present a DH domain, it keeps a PH and a SH3 domain. PH domains perform essential contact with Cdc42 and RhoA in the DBL structure (87). They are also known to be responsible for protein subcellular localization and phosphoinositide interaction (88). Moreover, SH3 (Src homology 3) domains are abundant in oncogenic pathways such as cell migration and proliferation, distributed along with many different protein structures (89). SH3 domains have also been implicated in pancreatic cancer, specifically due to its relevance for oncogenic pathways (90). Although only a few isoforms of MCF2L have been initially explored, such as MCF2L-203—which does not catalyze guanine nucleotide exchange on CDC42—and MCF2L-205—which, on the other hand, activates CDC42 (91)—MCF2L-201 requires further investigation. Details about how the PH-SH3 protein may act and its role in pancreatic cancer deserve deeper analyses, even though our study provides some guidance on that.

The Huntingtin gene is mostly known to cause Huntington’s disease, being even referenced in a patent to be used as a new therapeutic target to treat this disease (92), by the expansion of the trinucleotide CAG in its first exon. Despite that, it has a wide tissue expression and its trinucleotide expansion has been correlated to tumor progression, including metastasis, and inversely correlated to carcinogenesis (93). Huntingtin transcript HTT-202 is non-canonical and we found it down-regulated in pancreatic tumors. Its protein structure presents neither the characteristic polymorphic trinucleotide repetitive region nor the main huntingtin annotated domain: DUF3652; thus, its function is an enigma. A similar case involves the BBS1 gene since it is most known for its association with the Bardet-Biedl Syndrome (BBS) (94). More interesting is the fact that higher expression of BBS1 was related to better survival in patients with malignant pleural mesothelioma (95), although in our PAAD signature this gene was down-regulated. Furthermore, BBS1 was part of a 15-gene signature associated with bone metastasis in breast carcinomas. Specifically, its up-regulation was correlated to the epithelial to mesenchymal transition status of the tumor (96). Overall, Reboot’s algorithm makes splicing isoform expression analysis feasible in cancer prognosis.

In conclusion, Reboot is a novel algorithm to seek, evaluate, and validate genes and transcripts (splicing isoform) signatures acting as prognostic or diagnostic biomarkers in cancer. Reboot brings novelties by combining a multivariate strategy with penalized Cox regression (LASSO method) and a bootstrap approach, plus a variety of statistical tests to find genes and transcripts candidates. Moreover, Reboot shows its usefulness by identifying prognostic genes and a minimal set of genes associated with glioblastoma patients’ survival and a splicing isoforms signature associated with pancreatic adenocarcinoma. Additionally, Reboot has good performance even running in standard laptops. We believe that Reboot will be of immediate interest to the cancer research community because it will accelerate and democratize the search for genes and transcripts biomarkers, even by researchers and clinicians without extensive bioinformatics training.

## DATA AVAILABILITY

Reboot is implemented in R version 4 and available both as an R script and Docker image that are freely available under the GNU General Public Licence version 3 (GPL3) at https://galantelab.github.io/reboot/. Reboot updates will be announced at its webpage. Docker images will be released along with new versions. Reboot is also available through a web interface at https://www.bioinfo.mochsl.org.br/reboot/.

## Supplementary Material

zcab024_Supplemental_File
