# Supplementary material for: Reboot: a straightforward approach to identify genes and splicing isoforms associated with cancer patient prognosis
Source: NAR Cancer. 2021 Jun 15;3(2):zcab024. doi: 10.1093/narcan/zcab024 (PMC8210018; doi:10.1093/narcan/zcab024)
Supplement: zcab024_Supplemental_File [file zcab024_supplemental_file.pdf]

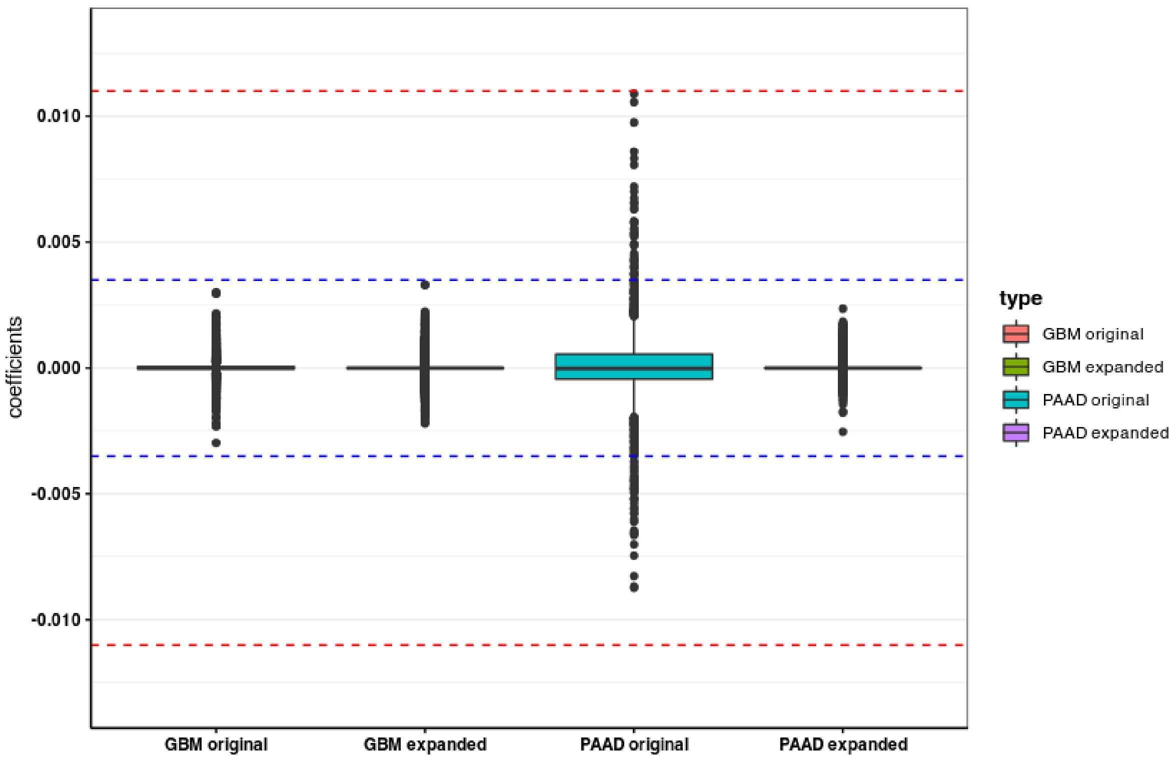

Figure S1: Random bootstrap resampling analyses for GBM and PAAD. Datasets of both GBM original and expanded dataset (genes) and PAAD original (transcripts) and expanded dataset (genes) were randomly resampled (patients and attributes) with bootstrap iterations in order to establish an empirical coefficient cutoff aiming to avoid false-positive results. Red dotted line for transcripts and blue dotted line for genes.

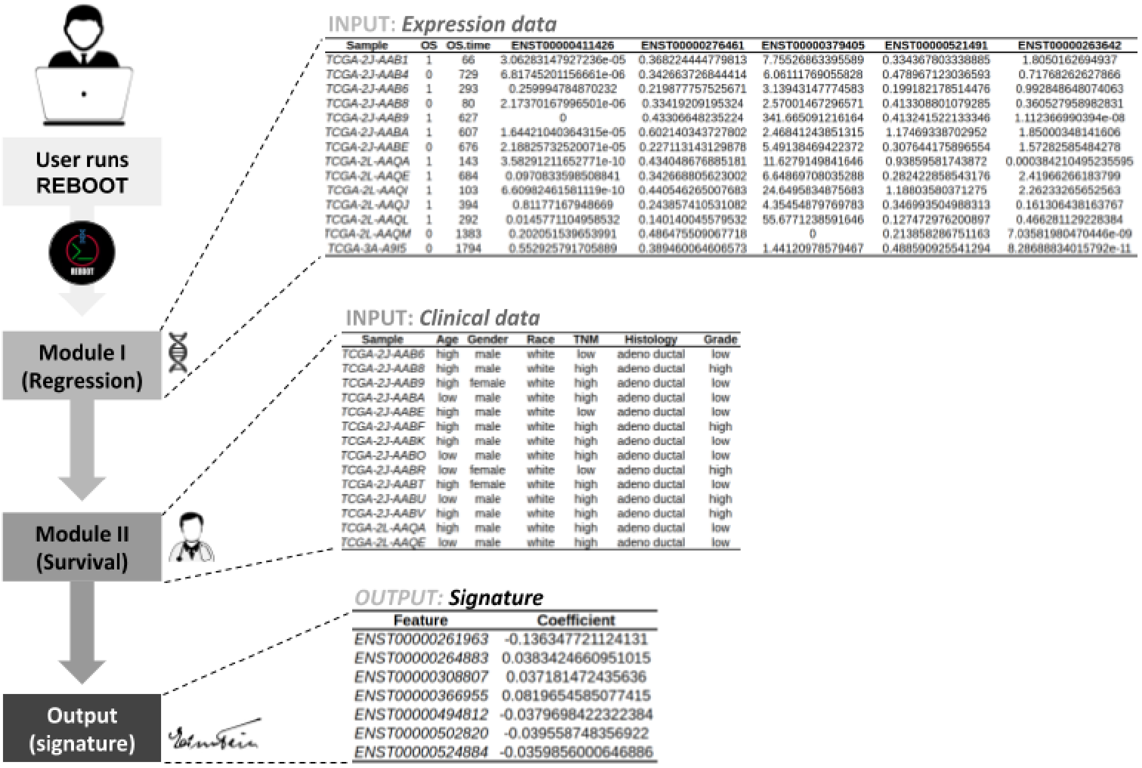

Figure S2: Workflow of inputs to Reboot. "Module I" requires expression data with values in TPM or FPKM. The first three columns must be the sample identifiers, patients' status (alive = 0 or dead = 1) and follow-up times (in days). "Module II" requires clinical data for multivariate analyses, where the sample identifiers match the ones present in expression data. Finally, Reboot produces several textual and graphical outputs such as a genetic signature, which is automatically used to generate a risk score evaluated in survival analyses when the "complete" mode is chosen.

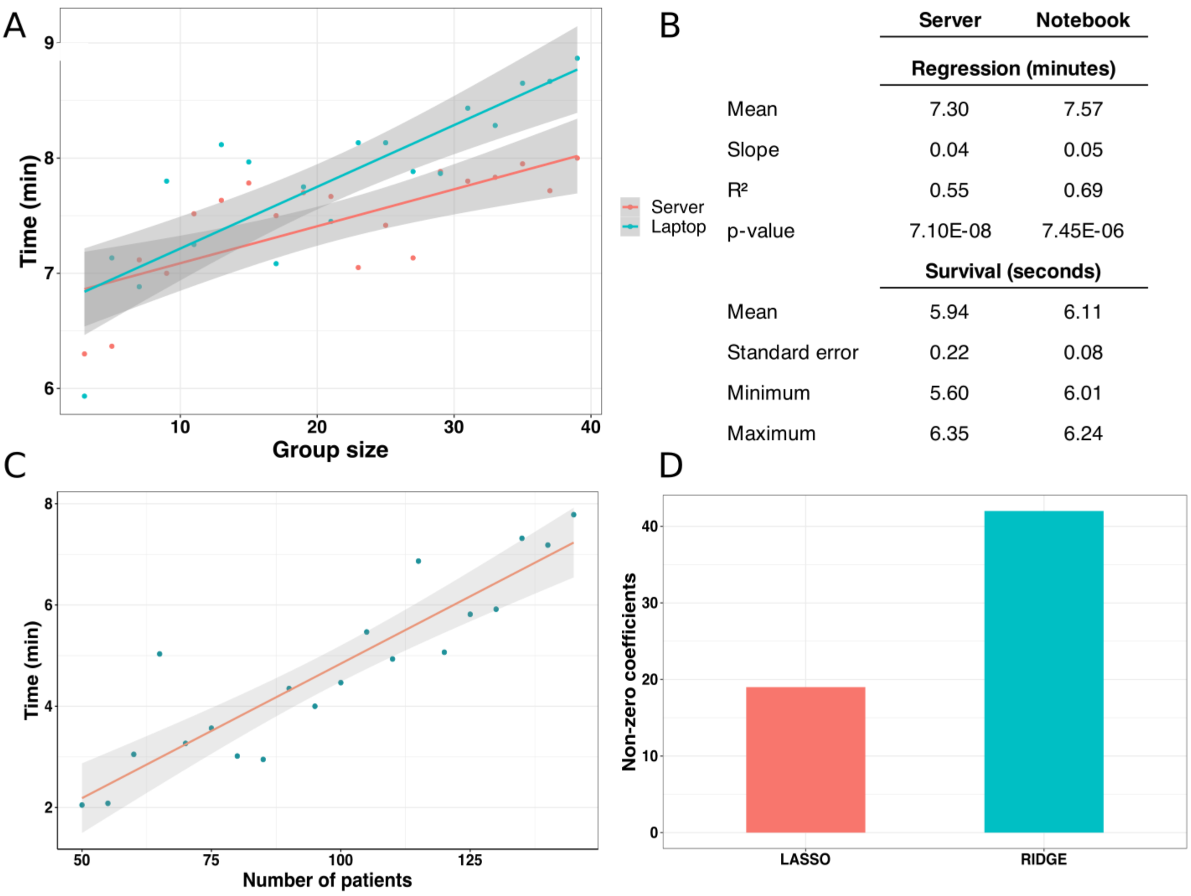

	Server	Notebook
	**Regression (minutes)**	
Mean	7.30	7.57
Slope	0.04	0.05
R^2	0.55	0.69
p-value	7.10E-08	7.45E-06
	**Survival (seconds)**	
Mean	5.94	6.11
Standard error	0.22	0.08
Minimum	5.60	6.01
Maximum	6.35	6.24

Figure S3: Computational assessment of Reboot. (A) Evaluation of group size impact on time performance of Reboot. Number of iterations was set to 100 and remaining parameters default. (B) Table showing numerical results for the assay performed in A splitted by module. (C) Run time analysis for instances (patients) variation. Processes were run in the laptop described in section “Usage and performance”. Group size was set to 20, the number of iterations to 100, and remaining parameters default. (D) Number of non-zero coefficients obtained by running Ridge and LASSO algorithms with group size set to 20, number of iterations set to 500, and remaining parameters default.

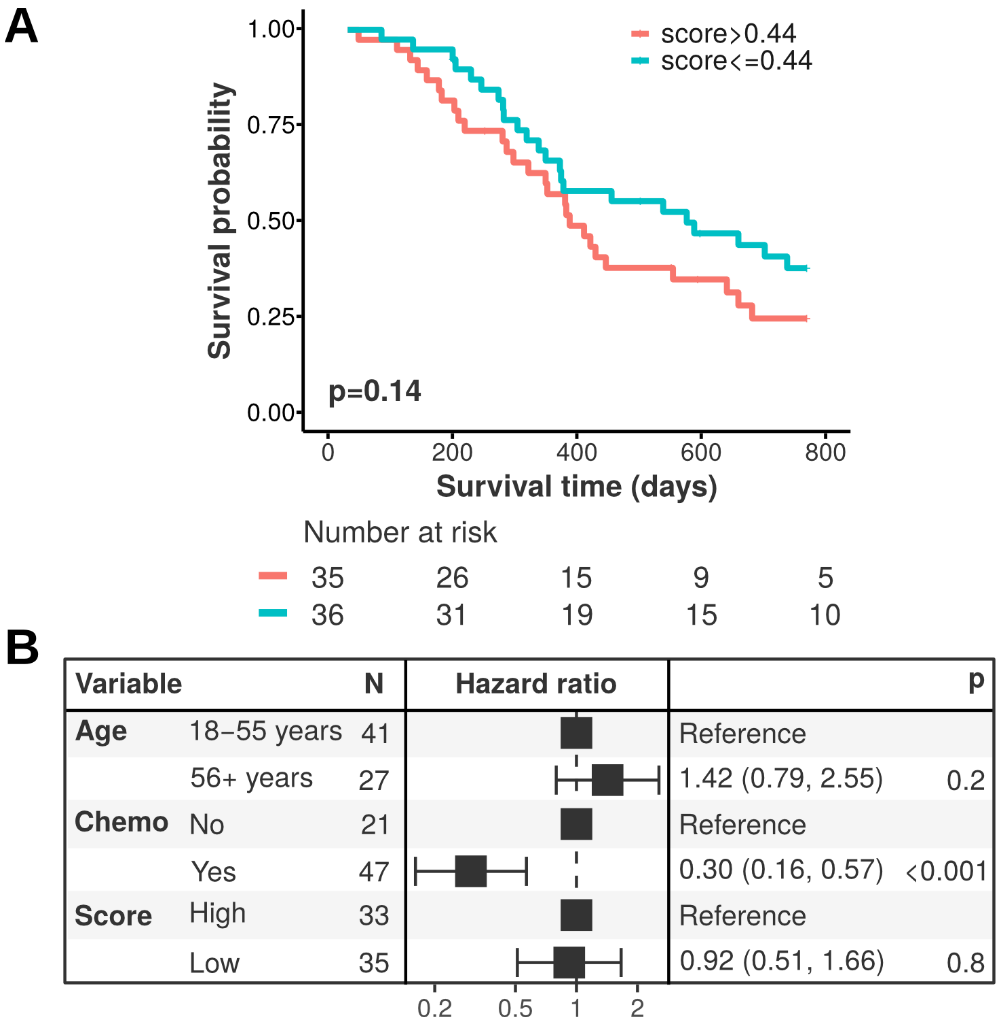

Figure S4: Validation of the 3-gene signature score in glioblastoma patients from the CGGA cohort. (A) Kaplan-Meier curve based on the 3-gene signature score; (B) Forest plot of a multivariate model including the 3-gene signature score along with clinical parameters relevant to prognosis in GBM.

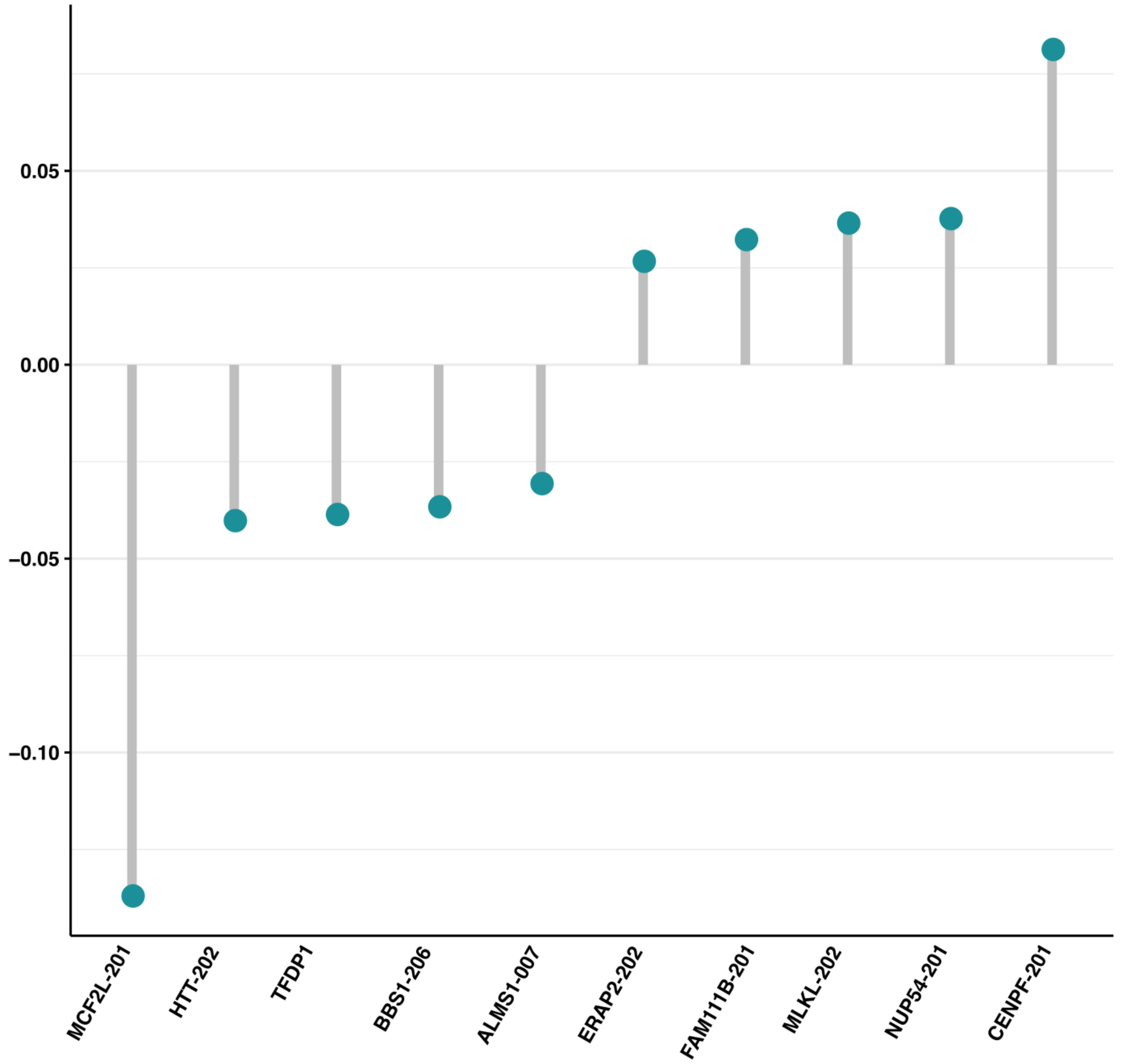

Figure S5: Reboot's Lollipop plot. Top 10 transcripts present in generated signature, which are relevant for PAAD survival, where the higher the coefficients (in module), the greater the influence of that transcript to the final patients' outcome.

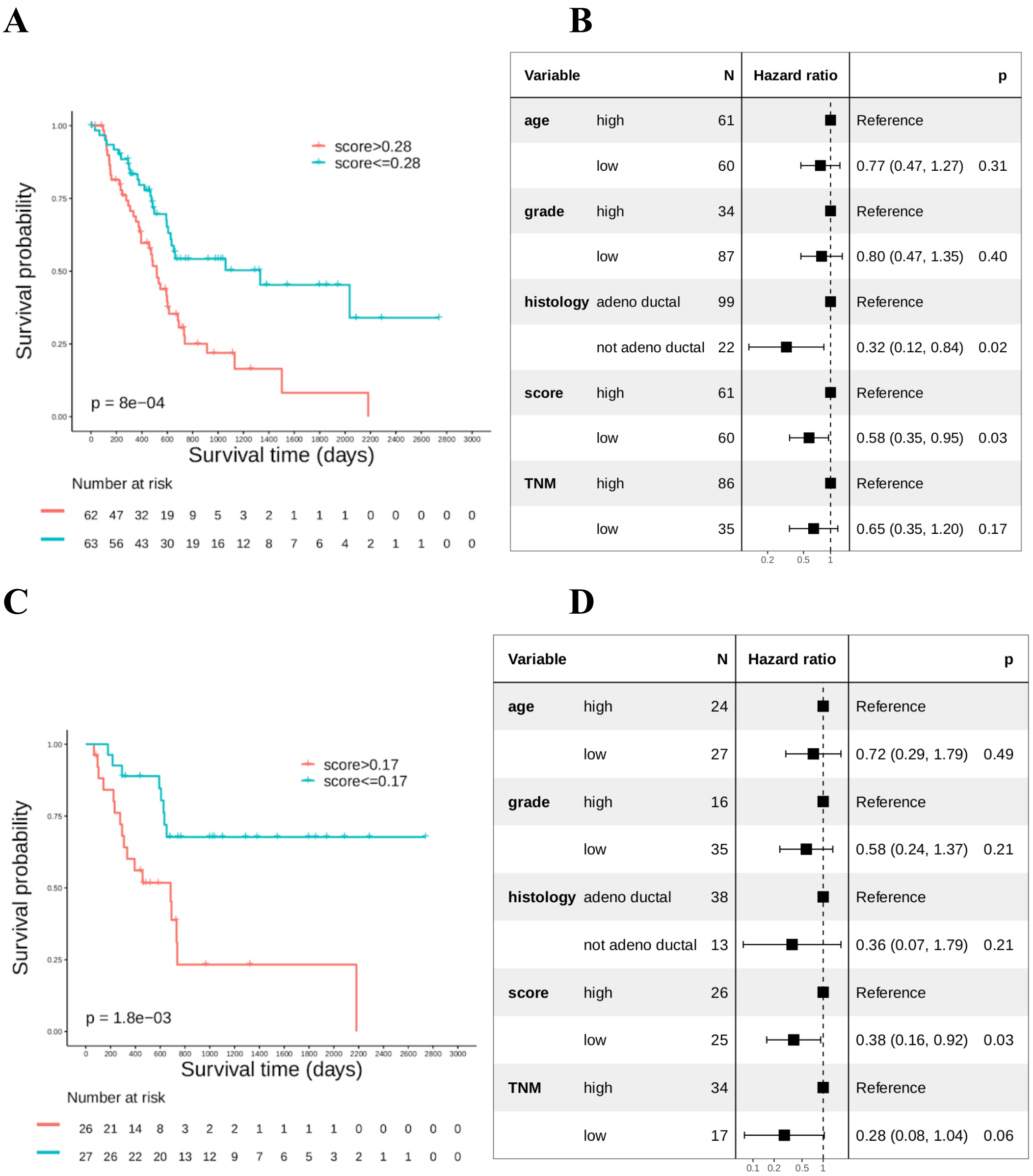

Figure S6: Reboot's outputs of PAAD outcomes. Univariate and multivariate survival analyses for training (A) and (B) and validation (C) and (D) datasets. (A) Kaplan-Meier displaying that patients with higher scores (above median value) have worse prognosis. Follow-up time (days) is shown below curves. (B) Forest plot showing the significance of the score in predicting patients' outcome with correction for other relevant clinical variables. Similarly, (C) Kaplan-Meier curve evidencing that patients with higher scores (above median value) have worse prognosis. Follow-up time (days) is shown below curves. (B) Forest plot confirming the significance of the score in predicting patients' outcome with correction for other relevant clinical variables.

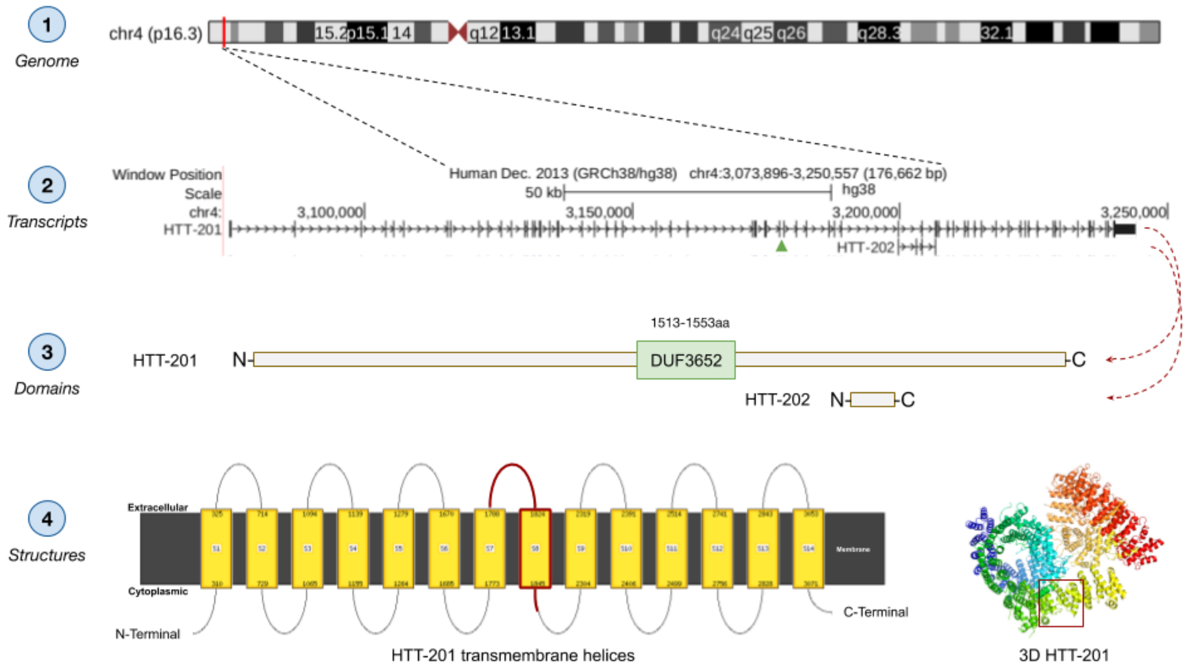

Figure S7: Transcripts of the Huntingtin (HTT) gene. (1) Chromosome location of HTT, which is part of the final signature that produced a score significant for survival of patients with PAAD. (2) Transcripts 201 and 202 of HTT pinpointing the lack of the DUF3652 domain in the much smaller HTT-202 isoform, which is present in the final signature. (3) Transcripts 201 and 202 of HTT evidencing the position of the DUF3652 domain in the canonical HTT-201 isoform. (4) Predicted structure of HTT-201 isoform with its 14 transmembrane helices (left), highlighting in red the portion representative of HTT-202. The modeled 3D structure of HTT-201 (right) is shown in cartoon representation with rainbow coloring, where α-helix secondary structures are the helices and β-sheet secondary structures are the arrows heading to the C-terminal. The portion inside the red box is the part of HTT-201 representative of HTT-202.

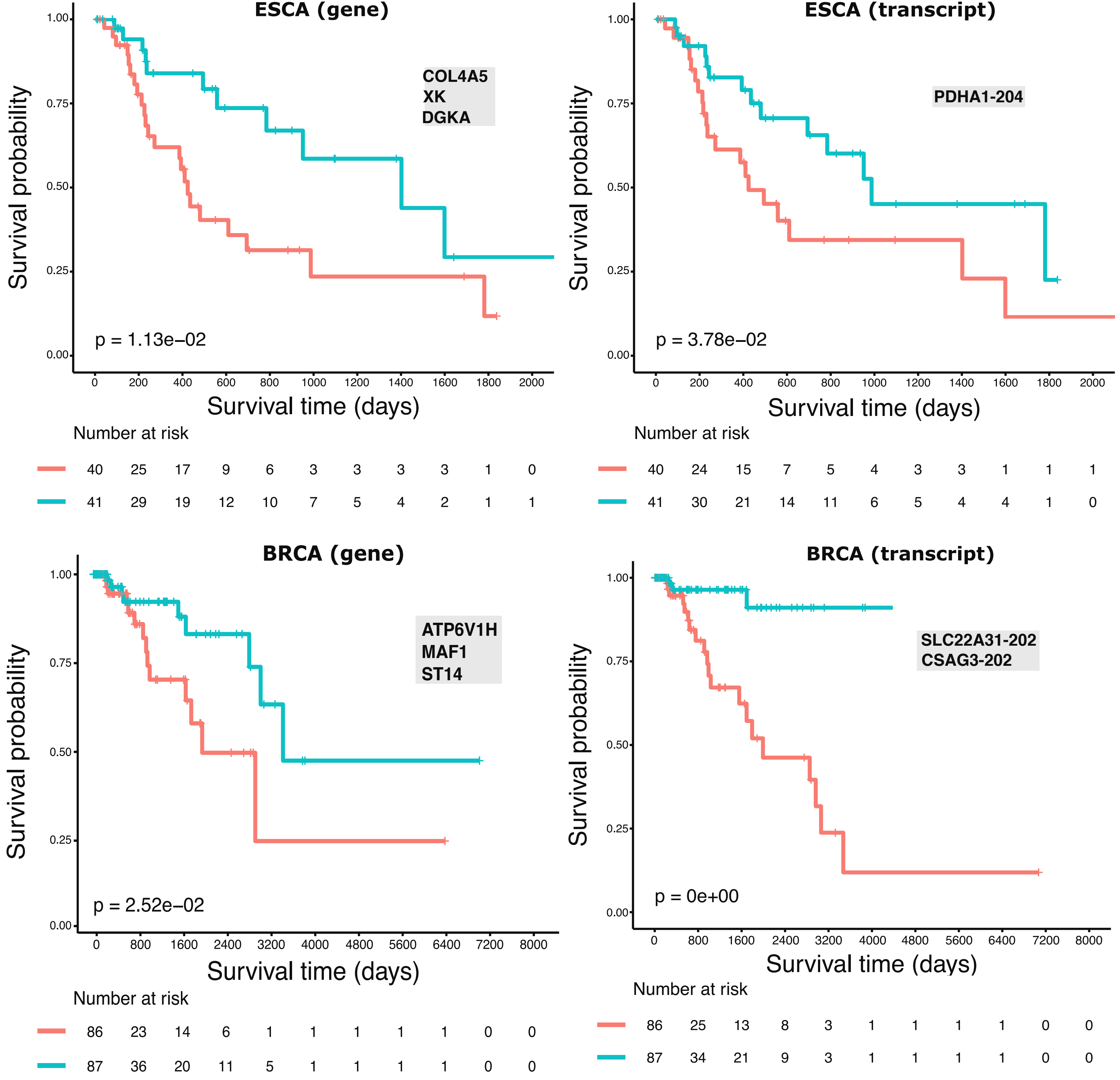

Figure S8: Survival analysis for TN BRCA and ESCA tumors. Prognosis was obtained through minimal signature, p-value lower than 0.05, by median cut off of patients' score. Genes/transcripts signatures are shown in gray boxes for each case.

Table S1. Clinal data from 144 patients with glioblastoma and 9 clinical variables obtained from TCGA.

sample	gender	age	race	ethnicity	cancer.status	karnofsky	chemotherapy	IDH.status	MGMT.status
TCGA.02.0047	male	high	white	not hispanic or latino	with tumor	high	no	wt	unmethylated
TCGA.02.0055	female	high	white	not hispanic or latino	with tumor	high	yes	wt	unmethylated
TCGA.02.2483	male	low	no white	not hispanic or latino	with tumor	high	yes	mut	methylated
TCGA.02.2485	male	low	no white	not hispanic or latino	with tumor	high	yes	wt	unmethylated
TCGA.02.2486	male	high	white	not hispanic or latino	with tumor	high	yes	wt	unmethylated
TCGA.06.0129	male	low	no white	not hispanic or latino	with tumor	high	yes	mut	methylated
TCGA.06.0130	male	low	white	not hispanic or latino	with tumor	high	yes	wt	unmethylated
TCGA.06.0132	male	low	white	not hispanic or latino	with tumor	low	no	wt	NA
TCGA.06.0138	male	low	white	not hispanic or latino	with tumor	high	no	wt	NA
TCGA.06.0139	male	low	white	not hispanic or latino	with tumor	low	yes	wt	unmethylated
TCGA.06.0141	male	high	white	not hispanic or latino	with tumor	high	yes	wt	unmethylated
TCGA.06.0152	male	high	white	not hispanic or latino	with tumor	low	yes	NA	NA
TCGA.06.0157	female	high	white	not hispanic or latino	with tumor	low	no	wt	NA
TCGA.06.0158	male	high	white	not hispanic or latino	with tumor	high	yes	wt	NA
TCGA.06.0168	female	high	white	not hispanic or latino	with tumor	high	yes	wt	NA
TCGA.06.0171	male	high	white	not hispanic or latino	with tumor	high	yes	NA	NA
TCGA.06.0174	male	low	white	not hispanic or latino	with tumor	high	yes	wt	NA
TCGA.06.0178	male	low	white	not hispanic or latino	tumor free	low	yes	mut	NA
TCGA.06.0184	male	high	white	not hispanic or latino	tumor free	high	yes	wt	NA
TCGA.06.0187	male	high	white	not hispanic or latino	with tumor	low	no	wt	NA
TCGA.06.0219	male	high	white	not hispanic or latino	with tumor	low	no	wt	NA
TCGA.06.0221	male	low	white	not hispanic or latino	with tumor	high	yes	NA	NA
TCGA.06.0238	male	low	white	not hispanic or latino	with tumor	high	no	wt	NA
TCGA.06.0644	male	high	no white	not hispanic or latino	with tumor	high	yes	wt	NA
TCGA.06.0645	female	low	white	not hispanic or latino	with tumor	low	no	wt	NA
TCGA.06.0646	male	high	white	not hispanic or latino	with tumor	high	no	wt	NA
TCGA.06.0649	female	high	no white	not hispanic or latino	with tumor	low	no	wt	NA

TCGA.06.0686	male	low	white	not hispanic or latino	with tumor	low	yes	wt	NA
TCGA.06.0743	male	high	white	not hispanic or latino	with tumor	high	yes	wt	NA
TCGA.06.0744	male	high	white	not hispanic or latino	tumor free	high	yes	wt	NA
TCGA.06.0745	male	high	white	not hispanic or latino	with tumor	high	no	wt	NA
TCGA.06.0747	male	low	white	not hispanic or latino	with tumor	high	no	wt	NA
TCGA.06.0749	male	low	no white	not hispanic or latino	with tumor	low	no	wt	NA
TCGA.06.0750	male	low	white	not hispanic or latino	with tumor	high	no	wt	NA
TCGA.06.0878	male	high	white	not hispanic or latino	with tumor	high	yes	wt	unmethylated
TCGA.06.0882	male	low	white	not hispanic or latino	with tumor	low	yes	wt	unmethylated
TCGA.06.1804	female	high	white	not hispanic or latino	with tumor	low	no	wt	methylated
TCGA.06.2557	male	high	no white	not hispanic or latino	with tumor	low	no	wt	unmethylated
TCGA.06.2558	female	high	white	not hispanic or latino	NA	low	no	wt	unmethylated
TCGA.06.2559	male	high	white	not hispanic or latino	with tumor	low	yes	wt	methylated
TCGA.06.2561	female	low	white	not hispanic or latino	with tumor	high	yes	wt	unmethylated
TCGA.06.2562	male	high	white	not hispanic or latino	with tumor	high	yes	wt	unmethylated
TCGA.06.2563	female	high	white	not hispanic or latino	with tumor	high	yes	wt	methylated
TCGA.06.2564	male	low	white	not hispanic or latino	with tumor	high	yes	wt	unmethylated
TCGA.06.2565	male	high	no white	not hispanic or latino	with tumor	high	yes	wt	methylated
TCGA.06.2567	male	high	white	not hispanic or latino	with tumor	high	yes	wt	methylated
TCGA.06.2569	female	low	no white	not hispanic or latino	with tumor	high	no	wt	unmethylated
TCGA.06.2570	female	low	white	not hispanic or latino	with tumor	high	yes	mut	methylated
TCGA.06.5408	female	low	white	not hispanic or latino	with tumor	high	yes	wt	unmethylated
TCGA.06.5410	female	high	white	not hispanic or latino	with tumor	low	no	wt	methylated
TCGA.06.5411	male	low	white	not hispanic or latino	with tumor	high	yes	wt	unmethylated
TCGA.06.5412	female	high	white	not hispanic or latino	with tumor	high	yes	wt	methylated
TCGA.06.5413	male	high	white	not hispanic or latino	with tumor	low	yes	wt	unmethylated
TCGA.06.5414	male	high	white	not hispanic or latino	with tumor	high	yes	wt	unmethylated
TCGA.06.5416	female	low	white	not hispanic or latino	with tumor	high	yes	NA	unmethylated
TCGA.06.5417	female	low	white	not hispanic or latino	with tumor	high	yes	mut	methylated
TCGA.06.5418	female	high	white	not hispanic or latino	with tumor	low	no	wt	unmethylated

TCGA.06.5856	male	low	white	not hispanic or latino	with tumor	low	no	wt	unmethylated
TCGA.06.5858	female	low	white	not hispanic or latino	with tumor	high	yes	wt	unmethylated
TCGA.06.5859	male	high	white	not hispanic or latino	with tumor	low	yes	wt	unmethylated
TCGA.08.0386	male	high	white	not hispanic or latino	with tumor	high	yes	wt	NA
TCGA.12.0616	female	low	white	not hispanic or latino	with tumor	high	yes	wt	NA
TCGA.12.0618	male	low	white	not hispanic or latino	NA	low	yes	wt	NA
TCGA.12.0619	male	high	white	not hispanic or latino	with tumor	high	yes	wt	NA
TCGA.12.0821	male	high	white	not hispanic or latino	with tumor	low	yes	wt	unmethylated
TCGA.12.1597	female	high	white	not hispanic or latino	with tumor	high	yes	wt	unmethylated
TCGA.12.3650	male	low	white	not hispanic or latino	with tumor	high	yes	wt	unmethylated
TCGA.12.3652	male	high	white	not hispanic or latino	with tumor	high	yes	wt	unmethylated
TCGA.12.3653	female	low	white	not hispanic or latino	with tumor	high	yes	wt	unmethylated
TCGA.12.5295	female	high	white	not hispanic or latino	with tumor	low	yes	wt	methylated
TCGA.12.5299	female	low	white	not hispanic or latino	with tumor	high	yes	wt	unmethylated
TCGA.14.0736	male	low	no white	NA	with tumor	high	yes	NA	NA
TCGA.14.0781	male	low	no white	NA	with tumor	low	no	wt	unmethylated
TCGA.14.0787	male	high	no white	not hispanic or latino	NA	low	no	wt	methylated
TCGA.14.0789	male	low	white	NA	NA	low	yes	wt	methylated
TCGA.14.0790	female	high	white	NA	with tumor	low	yes	wt	methylated
TCGA.14.0817	female	high	white	NA	NA	low	no	wt	unmethylated
TCGA.14.0871	female	high	white	NA	NA	low	no	wt	unmethylated
TCGA.14.1402	female	low	white	NA	with tumor	high	yes	NA	NA
TCGA.14.1823	female	low	white	NA	with tumor	high	yes	wt	methylated
TCGA.14.1829	male	low	no white	NA	with tumor	low	yes	wt	unmethylated
TCGA.14.2554	female	low	white	NA	with tumor	low	no	wt	unmethylated
TCGA.16.0846	male	high	white	not hispanic or latino	with tumor	low	no	wt	methylated
TCGA.16.1045	female	low	white	not hispanic or latino	with tumor	low	no	wt	methylated
TCGA.19.0957	female	low	white	not hispanic or latino	with tumor	low	yes	NA	NA
TCGA.19.1389	male	low	white	not hispanic or latino	with tumor	high	yes	NA	NA
TCGA.19.1390	female	high	white	not hispanic or latino	NA	low	no	wt	methylated

TCGA.19.1787	male	low	white	not hispanic or latino	with tumor	high	yes	NA	methylated
TCGA.19.2619	female	low	no white	not hispanic or latino	with tumor	low	yes	wt	methylated
TCGA.19.2620	male	high	white	not hispanic or latino	tumor free	low	yes	wt	methylated
TCGA.19.2624	male	low	white	not hispanic or latino	tumor free	low	no	wt	unmethylated
TCGA.19.2625	female	high	white	not hispanic or latino	tumor free	low	no	wt	unmethylated
TCGA.19.2629	male	high	white	not hispanic or latino	with tumor	low	yes	mut	unmethylated
TCGA.19.5960	male	low	white	not hispanic or latino	tumor free	high	yes	wt	unmethylated
TCGA.26.1442	male	low	white	not hispanic or latino	with tumor	high	yes	mut	methylated
TCGA.26.5132	male	high	white	not hispanic or latino	tumor free	low	yes	wt	methylated
TCGA.26.5133	male	high	white	not hispanic or latino	tumor free	high	yes	wt	unmethylated
TCGA.26.5134	male	high	white	not hispanic or latino	tumor free	low	no	wt	unmethylated
TCGA.26.5136	female	high	white	not hispanic or latino	NA	low	no	wt	methylated
TCGA.26.5139	female	high	white	not hispanic or latino	with tumor	low	no	wt	unmethylated
TCGA.27.1830	male	low	white	not hispanic or latino	with tumor	high	yes	wt	unmethylated
TCGA.27.1831	male	high	white	not hispanic or latino	with tumor	high	yes	wt	unmethylated
TCGA.27.1832	female	high	white	not hispanic or latino	with tumor	high	yes	wt	unmethylated
TCGA.27.1834	male	low	white	not hispanic or latino	with tumor	high	yes	wt	methylated
TCGA.27.1835	female	low	white	not hispanic or latino	with tumor	high	yes	wt	methylated
TCGA.27.1837	male	low	white	not hispanic or latino	with tumor	high	yes	wt	methylated
TCGA.27.2519	male	low	white	not hispanic or latino	with tumor	high	yes	wt	unmethylated
TCGA.27.2521	male	low	white	not hispanic or latino	with tumor	high	yes	mut	methylated
TCGA.27.2523	male	high	white	not hispanic or latino	with tumor	high	yes	wt	methylated
TCGA.27.2524	male	low	white	not hispanic or latino	with tumor	high	yes	wt	unmethylated
TCGA.27.2526	female	high	white	not hispanic or latino	with tumor	low	no	wt	unmethylated
TCGA.27.2528	male	high	white	not hispanic or latino	with tumor	high	yes	wt	methylated
TCGA.28.1747	male	low	white	not hispanic or latino	NA	low	yes	wt	methylated
TCGA.28.1753	male	low	white	hispanic or latino	with tumor	low	no	wt	unmethylated
TCGA.28.2509	female	high	white	not hispanic or latino	NA	high	yes	wt	methylated
TCGA.28.2513	female	high	white	not hispanic or latino	with tumor	high	yes	wt	unmethylated
TCGA.28.2514	male	low	no white	not hispanic or latino	NA	low	yes	wt	unmethylated

TCGA.28.5207	male	high	white	NA	with tumor	low	yes	wt	unmethylated
TCGA.28.5208	male	low	white	NA	with tumor	low	yes	wt	methylated
TCGA.28.5209	female	high	white	NA	NA	low	yes	wt	methylated
TCGA.28.5215	female	high	white	NA	with tumor	high	yes	wt	methylated
TCGA.28.5216	male	low	white	NA	tumor free	high	yes	wt	unmethylated
TCGA.28.5218	male	high	white	NA	NA	low	yes	wt	unmethylated
TCGA.28.5220	male	high	white	NA	with tumor	high	yes	wt	unmethylated
TCGA.32.1970	male	high	white	NA	with tumor	low	yes	wt	unmethylated
TCGA.32.1980	male	high	white	not hispanic or latino	with tumor	low	no	wt	unmethylated
TCGA.32.1982	female	high	white	not hispanic or latino	with tumor	high	yes	wt	methylated
TCGA.32.2615	male	high	white	not hispanic or latino	with tumor	high	yes	wt	unmethylated
TCGA.32.2616	female	low	white	hispanic or latino	with tumor	low	yes	NA	methylated
TCGA.32.2634	male	high	white	not hispanic or latino	tumor free	low	yes	wt	methylated
TCGA.32.2638	male	high	white	not hispanic or latino	tumor free	low	yes	wt	methylated
TCGA.32.4213	female	low	white	not hispanic or latino	with tumor	low	yes	wt	methylated
TCGA.32.5222	male	high	white	not hispanic or latino	with tumor	low	yes	wt	methylated
TCGA.41.2571	male	high	white	not hispanic or latino	with tumor	low	yes	wt	unmethylated
TCGA.41.2572	male	high	white	not hispanic or latino	with tumor	low	yes	wt	unmethylated
TCGA.41.3915	male	low	white	not hispanic or latino	with tumor	low	yes	wt	methylated
TCGA.41.4097	female	high	white	not hispanic or latino	with tumor	low	no	wt	unmethylated
TCGA.41.5651	female	high	no white	not hispanic or latino	with tumor	low	yes	wt	methylated
TCGA.76.4925	male	high	white	not hispanic or latino	NA	high	yes	wt	methylated
TCGA.76.4926	male	high	white	NA	with tumor	high	yes	wt	unmethylated
TCGA.76.4927	male	low	white	NA	with tumor	high	no	wt	unmethylated
TCGA.76.4928	female	high	white	NA	with tumor	high	yes	wt	methylated
TCGA.76.4929	female	high	white	not hispanic or latino	with tumor	high	no	wt	methylated
TCGA.76.4931	female	high	white	NA	with tumor	high	yes	wt	unmethylated
TCGA.76.4932	female	low	white	NA	with tumor	high	yes	wt	methylated

IDH: Isocitrate dehydrogenase 1; MGMT: O-6-methylguanine-DNA methyltransferase; wt: wild type; mut: mutated.

Table S2. Expression (in FPKM) data from 144 patients with glioblastoma and 50 genes from TCGA.

sample	OS	OS.time	ENSG00000230960	ENSG00000274747	ENSG00000104522	ENSG00000258580	ENSG00000135363	ENSG00000198526	ENSG00000120725	ENSG00000261208	ENSG00000223558	ENSG00000163666	ENSG00000170085	ENSG00000258877	ENSG00000189326	ENSG00000223906
TCGA.02.0047	1.000	448.000	0.000	0.000	9.204	0.080	8.980	0.041	9.712	0.038	0.103	0.793	1.936	0.000	0.000	0.000
TCGA.02.0055	1.000	76.000	0.016	0.000	21.978	0.113	7.614	0.023	15.523	0.000	0.029	1.180	1.916	0.000	0.000	0.000
TCGA.02.2483	0.000	466.000	0.000	0.000	27.102	0.000	14.188	0.010	6.346	0.029	0.391	0.765	0.233	0.000	0.000	0.040
TCGA.02.2485	0.000	470.000	0.016	0.000	16.174	0.023	24.699	0.012	4.840	0.000	0.000	1.938	0.180	0.000	0.000	0.000
TCGA.02.2486	1.000	493.000	0.000	0.000	13.188	0.102	35.223	0.013	16.058	0.000	0.033	3.253	0.379	0.000	0.000	0.000
TCGA.06.0129	1.000	989.000	0.000	0.000	10.258	0.020	17.836	0.000	6.110	0.000	0.337	3.542	0.249	0.000	0.000	0.000
TCGA.06.0130	1.000	320.000	0.000	0.000	20.335	0.045	18.333	0.023	10.765	0.065	0.058	3.803	2.375	0.000	0.000	0.000
TCGA.06.0132	1.000	570.000	0.000	0.000	11.003	0.024	17.120	0.000	9.903	0.011	0.000	0.659	1.830	0.000	0.000	0.000
TCGA.06.0138	1.000	674.000	0.019	0.000	13.912	0.000	23.688	0.000	6.080	0.000	0.036	2.365	1.331	0.000	0.000	0.055
TCGA.06.0139	1.000	327.000	0.000	0.000	14.833	0.000	17.352	0.000	11.643	0.077	0.000	1.736	0.891	0.000	0.000	0.000
TCGA.06.0141	1.000	280.000	0.014	0.061	14.173	0.000	25.310	0.031	12.189	0.047	0.000	1.633	1.947	0.000	0.000	0.000
TCGA.06.0152	1.000	359.000	0.014	0.000	10.824	0.020	62.678	0.032	12.759	0.039	0.026	0.975	1.524	0.000	0.000	0.000
TCGA.06.0157	1.000	97.000	0.000	0.065	11.754	0.000	31.265	0.000	7.729	0.010	0.000	0.990	1.413	0.012	0.000	0.000
TCGA.06.0158	1.000	166.000	0.000	0.000	10.132	0.020	25.689	0.020	8.395	0.000	0.051	1.484	1.371	0.000	0.000	0.000
TCGA.06.0168	1.000	579.000	0.000	0.000	16.945	0.000	27.075	0.045	13.957	0.042	0.000	1.113	2.229	0.000	0.000	0.000
TCGA.06.0171	1.000	396.000	0.028	0.061	6.858	0.000	29.110	0.010	13.310	0.009	0.077	1.074	1.346	0.000	0.000	0.000
TCGA.06.0174	1.000	67.000	0.064	0.070	10.503	0.000	14.946	0.023	7.843	0.011	0.000	0.695	0.860	0.000	0.000	0.000
TCGA.06.0178	1.000	1642.000	0.019	0.000	11.622	0.000	9.013	0.014	7.105	0.000	0.070	0.866	0.163	0.000	0.000	0.000
TCGA.06.0184	1.000	1228.000	0.014	0.000	24.213	0.041	35.817	0.000	14.828	0.010	0.106	1.211	0.352	0.000	0.000	0.000
TCGA.06.0187	1.000	801.000	0.000	0.000	10.510	0.000	49.377	0.023	8.476	0.000	0.000	0.606	1.182	0.000	0.000	0.044
TCGA.06.0219	1.000	11.000	0.016	0.000	13.214	0.000	21.605	0.012	7.238	0.011	0.000	2.777	0.272	0.000	0.000	0.000
TCGA.06.0221	1.000	548.000	0.034	0.000	5.387	0.024	7.681	0.037	4.982	0.000	0.094	0.560	0.969	0.000	0.000	0.000
TCGA.06.0238	1.000	359.000	0.050	0.073	7.992	0.071	13.949	0.012	8.545	0.000	0.031	1.235	2.652	0.000	0.000	0.000
TCGA.06.0644	1.000	375.000	0.000	0.000	16.691	0.262	7.753	0.031	17.673	0.010	0.026	0.585	1.099	0.000	0.000	0.000
TCGA.06.0645	1.000	98.000	0.000	0.000	12.595	0.022	34.021	0.012	11.930	0.011	0.029	0.685	1.322	0.000	0.033	0.000
TCGA.06.0646	1.000	136.000	0.016	0.000	13.731	0.023	19.735	0.000	7.681	0.000	0.030	0.834	2.561	0.000	0.000	0.000
TCGA.06.0649	1.000	62.000	0.018	0.081	11.610	0.053	12.114	0.014	14.641	0.000	0.102	2.200	2.210	0.015	0.000	0.053
TCGA.06.0686	1.000	286.000	0.097	0.000	16.557	0.000	19.734	0.048	9.214	0.022	0.000	0.692	2.308	0.027	0.000	0.000
TCGA.06.0743	1.000	679.000	0.000	0.000	10.763	0.023	43.945	0.000	8.449	0.022	0.000	1.861	0.722	0.000	0.000	0.000
TCGA.06.0744	1.000	595.000	0.000	0.000	15.762	0.047	54.637	0.012	3.531	0.000	0.000	1.785	0.184	0.000	0.000	0.000
TCGA.06.0745	1.000	164.000	0.013	0.000	16.767	0.000	37.151	0.010	15.607	0.000	0.000	0.698	1.472	0.000	0.000	0.000
TCGA.06.0747	1.000	82.000	0.000	0.000	8.451	0.020	12.292	0.010	20.115	0.000	0.026	1.748	1.977	0.000	0.000	0.000
TCGA.06.0749	1.000	66.000	0.016	0.000	13.557	0.023	20.810	0.000	10.296	0.000	0.181	1.130	1.798	0.000	0.000	0.000
TCGA.06.0750	1.000	28.000	0.000	0.000	13.309	0.000	28.105	0.000	16.375	0.011	0.378	1.775	1.684	0.000	0.000	0.000
TCGA.06.0878	0.000	218.000	0.000	0.000	9.873	0.000	24.197	0.024	12.636	0.000	0.000	0.884	2.105	0.000	0.000	0.000
TCGA.06.0882	1.000	165.000	0.000	0.000	10.893	0.022	108.259	0.022	10.575	0.000	0.056	1.554	1.452	0.000	0.000	0.043
TCGA.06.1804	1.000	414.000	0.014	0.000	8.516	0.103	12.514	0.021	8.823	0.000	0.186	1.944	1.897	0.000	0.000	0.041
TCGA.06.2557	1.000	33.000	0.000	0.000	11.154	0.000	36.333	0.032	9.730	0.020	0.027	2.400	1.557	0.000	0.000	0.000
TCGA.06.2558	1.000	23.000	0.051	0.000	12.452	0.049	21.804	0.013	7.466	0.012	0.127	0.766	2.631	0.014	0.000	0.000
TCGA.06.2559	1.000	150.000	0.018	0.000	8.594	0.000	27.548	0.013	7.620	0.000	0.000	0.735	2.004	0.015	0.000	0.000
TCGA.06.2561	1.000	282.000	0.070	0.000	9.242	0.025	59.071	0.052	12.259	0.000	0.000	1.706	1.876	0.000	0.000	0.000
TCGA.06.2562	1.000	86.000	0.000	0.000	12.789	0.085	60.316	0.000	11.007	0.000	0.000	0.523	0.386	0.000	0.000	0.000
TCGA.06.2563	0.000	932.000	0.000	0.000	16.818	0.000	40.293	0.012	7.767	0.000	0.000	0.976	0.798	0.000	0.000	0.047
TCGA.06.2564	0.000	181.000	0.014	0.000	9.811	0.040	37.135	0.010	8.710	0.010	0.000	0.868	1.528	0.000	0.000	0.000
TCGA.06.2565	1.000	207.000	0.015	0.000	12.850	0.257	64.705	0.011	8.156	0.000	0.055	1.339	0.658	0.012	0.000	0.000
TCGA.06.2567	1.000	133.000	0.000	0.000	11.547	0.063	11.804	0.000	8.743	0.000	0.054	2.286	3.954	0.000	0.000	0.000
TCGA.06.2569	0.000	13.000	0.000	0.000	47.198	0.000	8.819	0.036	9.351	0.055	0.386	0.753	3.733	0.013	0.000	0.000
TCGA.06.2570	0.000	958.000	0.016	0.000	13.609	0.047	16.724	0.000	7.155	0.000	0.241	0.953	2.809	0.000	0.000	0.000
TCGA.06.5408	1.000	342.000	0.014	0.000	12.293	0.000	27.538	0.010	8.889	0.000	0.052	1.518	2.172	0.012	0.000	0.000
TCGA.06.5410	1.000	108.000	0.013	0.058	9.490	0.057	66.383	0.020	18.380	0.000	0.025	0.780	1.347	0.000	0.000	0.000
TCGA.06.5411	1.000	254.000	0.094	0.000	14.196	0.058	22.828	0.010	7.513	0.000	0.124	0.828	2.287	0.000	0.000	0.000
TCGA.06.5412	1.000	138.000	0.000	0.000	15.623	0.021	73.173	0.042	17.942	0.010	0.053	0.875	0.955	0.000	0.000	0.041
TCGA.06.5413	0.000	268.000	0.000	0.000	15.542	0.000	23.870	0.011	11.526	0.010	0.000	0.812	3.040	0.000	0.000	0.000
TCGA.06.5414	0.000	273.000	0.000	0.000	12.504	0.000	57.083	0.038	9.762	0.009	0.000	0.898	1.174	0.000	0.000	0.037
TCGA.06.5416	0.000	204.000	0.024	0.209	19.321	0.103	11.933	0.000	26.823	0.016	0.486	0.941	2.826	0.000	0.000	0.000
TCGA.06.5417	0.000	155.000	0.062	0.000	8.057	0.000	9.094	0.137	5.998	0.042	0.115	4.173	0.343	0.000	0.000	0.000
TCGA.06.5418	1.000	83.000	0.000	0.000	14.108	0.000	30.560	0.000	12.033	0.000	0.029	1.202	1.756	0.013	0.000	0.000
TCGA.06.5856	1.000	114.000	0.000	0.000	10.683	0.000	28.607	0.032	9.724	0.000	0.000	2.520	1.768	0.000	0.000	0.000
TCGA.06.5858	0.000	187.000	0.000	0.000	10.663	0.021	18.987	0.011	9.426	0.020	0.000	1.206	3.154	0.000	0.000	0.000
TCGA.06.5859	0.000	139.000	0.000	0.000	7.905	0.000	25.852	0.000	22.107	0.000	0.000	1.788	5.933	0.000	0.000	0.000
TCGA.08.0386	1.000	478.000	0.000	0.000	12.904	0.022	12.404	0.012	7.623	0.011	0.000	1.683	0.229	0.013	0.000	0.000
TCGA.12.0616	1.000	439.000	0.025	0.109	18.050	0.036	14.182	0.000	9.125	0.017	0.000	2.459	1.931	0.021	0.000	0.000
TCGA.12.0618	1.000	50.000	0.039	0.000	11.864	0.037	7.941	0.000	8.092	0.009	0.024	0.441	0.186	0.000	0.000	0.000
TCGA.12.0619	1.000	316.000	0.000	0.000	10.509	0.199	14.518	0.020	14.809	0.000	0.000	1.078	3.042	0.023	0.000	0.000
TCGA.12.0821	1.000	323.000	0.015	0.068	7.886	0.000	9.069	0.023	9.558	0.011	0.286	0.592	3.546	0.000	0.000	0.000
TCGA.12.1597	1.000	427.000	0.020	0.087	13.919	0.000	12.567	0.015	9.563	0.014	0.000	0.338	1.220	0.000	0.000	0.000
TCGA.12.3650	1.000	333.000	0.000	0.000	20.680	0.000	28.632	0.020	8.814	0.000	0.000	0.609	0.490	0.000	0.000	0.077
TCGA.12.3652	1.000	1050.000	0.018	0.080	15.153	0.000	41.817	0.040	15.789	0.000	0.000	1.257	1.242	0.000	0.000	0.000
TCGA.12.3653	1.000	357.000	0.000	0.106	17.552	0.035	26.535	0.036	9.988	0.016	0.000	1.516	2.219	0.000	0.000	0.000
TCGA.12.5295	1.000	399.000	0.000	0.000	23.314	0.000	37.258	0.028	15.533	0.013	0.000	1.523	1.071	0.000	0.000	0.000
TCGA.12.5299	1.000	98.000	0.000	0.000	14.025	0.040	44.499	0.000	12.684	0.000	0.000	1.287	1.034	0.000	0.000	0.000
TCGA.14.0736	1.000	460.000	0.000	0.000	10.975	0.000	14.299	0.000	11.707	0.019	0.052	1.765	1.173	0.023	0.000	0.000
TCGA.14.0781	1.000	29.000	0.000	0.000	11.034	0.000	47.041	0.000	15.013	0.017	0.024	0.827	0.712	0.000	0.000	0.000
TCGA.14.0787	1.000	68.000	0.000	0.000	14.811	0.000	61.203	0.018	13.840	0.000	0.325	1.496	0.355	0.000	0.000	0.000
TCGA.14.0789	1.000	342.000	0.000	0.000	7.731	0.000	24.286	0.028	10.646	0.000	0.000	0.652	0.905	0.000	0.000	0.000
TCGA.14.0790	1.000	419.000	0.000	0.000	10.982	0.033	42.148	0.017	5.524	0.016	0.000	1.056	0.476	0.000	0.000	0.000
TCGA.14.0817	1.000	164.000	0.000	0.000	23.827	0.023	21.461	0.036	9.874	0.022	0.000	2.145	0.874	0.000	0.000	0.000
TCGA.14.0871	1.000	880.000	0.000	0.000	47.739	0.000	5.619	0.142	7.174	0.066	0.000	2.470	8.911	0.000	0.000	0.000
TCGA.14.1402	1.000	975.000	0.000	0.000	13.820	0.087	40.465	0.030	6.472	0.014	0.150	2.595	0.200	0.000	0.000	0.000
TCGA.14.1823	1.000	543.000	0.000	0.000	9.168	0.000	17.717	0.015	12.243	0.028	0.000	2.091	1.264	0.000	0.000	0.000
TCGA.14.1829	0.000	218.000	0.056	0.000	14.368	0.053	12.008	0.014	7.155	0.013	0.034	2.294	1.070	0.000	0.000	0.000
TCGA.14.2554	1.000	532.000	0.000	0.000	10.095	0.000	25.308	0.011	10.457	0.000	0.027	1.147	1.681	0.000	0.000	0.000
TCGA.16.0846	1.000	119.000	0.035	0.000	11.944	0.050	26.735	0.000	9.777	0.012	0.032	3.847	2.428	0.000	0.000	0.049
TCGA.16.1045	1.000	883.000	0.020	0.000	9.656	0.000	25.971	0.000	12.156	0.027	0.036	1.771	1.249	0.000	0.000	0.000
TCGA.19.0957	1.000	666.000	0.033	0.000	6.580	0.070	16.372	0.000	10.648	0.011	0.121	0.731	1.315	0.000	0.000	0.000
TCGA.19.1389	1.000	141.000	0.017	0.000	10.604	0.000	32.414	0.013	20.049	0.000	0.193	1.054	2.160	0.014	0.000	0.000
TCGA.19.1390	1.000	772.000	0.027	0.000	11.131	0.020	4.992	0.010	10.545	0.009	0.025	1.193	4.437	0.011	0.000	0.000
TCGA.19.1787	1.000	385.000	0.000	0.000	14.883	0.143	12.400	0.031	14.455	0.000	0.000	2.291	5.982	0.012	0.000	0.000
TCGA.19.2619	0.000	294.000	0.000	0.000	8.633	0.000	61.065	0.010	7.442	0.009	0.000	1.109	0.978	0.011	0.000	0.000
TCGA.19.2620	1.000	148.000	0.000	0.000	8.083	0.073	27.665	0.000	10.247	0.017	0.000	1.404	1.381	0.021	0.000	0.000
TCGA.19.2624	1.000	5.000	0.014	0.000	11.878	0.019	28.574	0.020	8.619	0.000	0.050	1.499	1.930	0.000	0.000	0.077
TCGA.19.2625	1.000	124.000	0.000	0.000	8.618	0.000	18.050	0.010	11.176	0.010	0.026	1.533	2.992	0.000	0.000	0.000
TCGA.19.2629	1.000	501.000	0.000	0.059	11.003	0.019	16.213	0.010	14.743	0.000	0.075	1.622	0.282	0.000	0.000	0.000
TCGA.19.5960	1.000	165.000	0.127	0.000	10.812	0.122	6.101	0.031	6.801	0.000	0.105	2.727	4.425	0.000	0.000	0.000
TCGA.26.1442	0.000	953.000	0.000	0.000	6.810	0.059	26.174	0.040	6.118	0.000	0.202	1.278	0.511	0.011	0.000	0.039
TCGA.26.5132	0.000	286.000	0.000	0.000	16.206	0.000	14.370	0.012	12.297	0.000	0.029	1.971	1.553	0.000	0.034	0.000
TCGA.26.5133	0.000	452.000	0.050	0.000	17.515	0.024	27.179	0.000	8.067	0.000	0.245	1.218	1.866	0.000	0.000	0.000
TCGA.26.5134	0.000	167.000	0.135	1.779	13.491	0.484	13.030	0.000	9.652	0.000	0.375	1.799	3.780	0.000	0.000	0.000
TCGA.26.5136	1.000	37.000	0.000	0.000	13.343	0.123	33.758	0.063	12.119	0.058	0.158	4.783	2.280	0.000	0.000	0.000
TCGA.26.5139	0.000	48.000	0.000	0.000	11.437	0.000	54.468	0.024	11.439	0.011	0.061	0.548	1.208	0.000	0.000	0.000
TCGA.27.1830	1.000	146.000	0.033	0.000	10.832	0.024	12.797	0.012	10.330	0.000	0.367	1.040	1.454	0.000	0.000	0.047
TCGA.27.1831	1.000	505.000	0.065	0.000	16.541	0.000	45.607	0.000	7.841	0.015	0.000	2.048	0.607	0.000	0.000	0.000
TCGA.27.1832	1.000	0.000	0.000	0.000	13.611	0.121	18.077	0.000	21.711	0.019	0.052	3.528	4.078	0.000	0.000	0.000
TCGA.27.1834	1.000	1008.000	0.018	0.000	13.122	0.104	34.682	0.013	9.385	0.037	0.033	2.602	2.503	0.000	0.000	0.000
TCGA.27.1835	1.000	648.000	0.000	0.000	13.402	0.000	82.669	0.015	6.017	0.014	0.307	2.674	1.898	0.017	0.000	0.000
TCGA.27.1837	1.000	427.000	0.000	0.000	13.677	0.000	39.065	0.000	10.013	0.000	0.000	1.433	2.034	0.000	0.000	0.000
TCGA.27.2519	1.000	316.000	0.000	0.000	9.797	0.024	35.687	0.000	16.470	0.011	0.246	1.436	1.387	0.000	0.000	0.000
TCGA.27.2521	1.000	316.000	0.030	0.000	17.240	0.042	6.903	0.033	11.186	0.020	0.055	0.519	0.138	0.000	0.000	0.000
TCGA.27.2523	1.000	489.000	0.000	0.000	14.428	0.020	40.394	0.041	12.224	0.000	0.000	0.960	1.272	0.011	0.000	0.000
TCGA.27.2524	1.000	231.000	0.017	0.000	21.552	0.149	54.666	0.000	16.059	0.000	0.000	0.444	0.960	0.000	0.000	0.000
TCGA.27.2526	1.000	87.000	0.000	0.000	15.711	0.000	74.291	0.000	18.574	0.011	0.061	1.291	1.322	0.000	0.000	0.000
TCGA.27.2528	1.000	480.000	0.000	0.000	10.896	0.022	65.068	0.045	8.224	0.073	0.000	1.163	0.450	0.000	0.000	0.000
TCGA.28.1747	1.000	77.000	0.000	0.000	12.582	0.000	32.718	0.012	9.250	0.000	0.000	1.861	1.274	0.000	0.000	0.000
TCGA.28.1753	0.000	37.000	0.034	0.000	12.226	0.000	22.333	0.000	9.789	0.093	0.252	1.051	0.547	0.000	0.000	0.000
TCGA.28.2509	0.000	145.000	0.016	0.000	12.072	0.023	17.513	0.012	12.632	0.000	0.000	1.859	1.840	0.000	0.000	0.000
TCGA.28.2513	0.000	222.000	0.000	0.075	14.974	0.000	21.474	0.013	14.415	0.000	0.063	0.603	1.768	0.000	0.000	0.000
TCGA.28.2514	0.000	160.000	0.059	0.065	8.608	0.000	33.414	0.000	6.095	0.000	0.027	0.473	1.145	0.000	0.000	0.000
TCGA.28.5207	1.000	275.000	0.017	0.000	14.546	0.000	45.760	0.013	8.636	0.000	0.032	1.516	2.363	0.014	0.000	0.000
TCGA.28.5208	1.000	474.000	0.000	0.000	13.741	0.000	36.164	0.022	10.505	0.000	0.028	1.093	1.254	0.000	0.000	0.000
TCGA.28.5209	0.000	442.000	0.000	0.000	11.439	0.000	46.938	0.021	9.463	0.010	0.026	0.784	1.471	0.000	0.000	0.000
TCGA.28.5215	1.000	237.000	0.115	0.000	12.500	0.000	15.985	0.057	12.293	0.000	0.118	0.886	1.343	0.000	0.000	0.000
TCGA.28.5216	0.000	415.000	0.039	0.000	11.408	0.000	13.200	0.014	12.473	0.013	0.036	1.724	2.217	0.000	0.000	0.000
TCGA.28.5218	1.000	128.000	0.000	0.000	23.944	0.024	7.364	0.050	9.490	0.023	0.031	0.324	2.720	0.000	0.000	0.000
TCGA.28.5220	1.000	319.000	0.000	0.000	14.509	0.000	10.036	0.000	9.335	0.000	0.000	4.197	1.836	0.014	0.000	0.048
TCGA.32.1970	1.000	468.000	0.000	0.000	13.966	0.000	26.047	0.012	10.369	0.011	0.029	1.643	1.357	0.000	0.000	0.045
TCGA.32.1980	1.000	36.000	0.030	0.000	9.182	0.000	40.881	0.033	9.575	0.000	0.055	0.591	1.813	0.000	0.000	0.000
TCGA.32.1982	1.000	142.000	0.000	0.000	16.933	0.000	55.980	0.072	10.556	0.000	0.000	1.303	1.410	0.000	0.000	0.000
TCGA.32.2615	1.000	485.000	0.013	0.057	10.207	0.019	24.755	0.019	10.773	0.026	0.048	2.273	0.804	0.000	0.000	0.000
TCGA.32.2616	1.000	224.000	0.012	0.053	13.384	0.052	35.443	0.000	15.561	0.008	0.000	1.124	1.962	0.000	0.000	0.000
TCGA.32.2634	0.000	693.000	0.013	0.114	10.062	0.000	14.026	0.010	8.867	0.009	0.144	1.590	5.714	0.000	0.000	0.111
TCGA.32.2638	1.000	644.000	0.000	0.108	11.210	0.018	47.678	0.009	9.861	0.000	0.000	1.000	0.347	0.000	0.000	0.000
TCGA.32.4213	0.000	604.000	0.000	0.512	15.333	0.000	32.915	0.029	15.329	0.027	0.000	1.326	0.841	0.000	0.000	0.000
TCGA.32.5222	1.000	323.000	0.000	0.000	14.019	0.000	27.821	0.031	7.741	0.000	0.000	1.317	0.952	0.000	0.000	0.000
TCGA.41.2571	1.000	26.000	0.000	0.079	16.636	0.052	13.230	0.000	10.530	0.025	0.000	1.077	3.147	0.000	0.000	0.000
TCGA.41.2572	1.000	406.000	0.000	0.000	16.745	0.000	61.929	0.014	13.335	0.000	0.000	1.077	0.848	0.000	0.000	0.000
TCGA.41.3915	1.000	360.000	0.000	0.179	10.626	0.000	11.821	0.015	12.221	0.028	0.038	1.653	2.192	0.000	0.000	0.000
TCGA.41.4097	1.000	6.000	0.000	0.000	10.512	0.033	32.161	0.000	12.419	0.031	0.000	1.050	1.316	0.000	0.000	0.000
TCGA.41.5651	1.000	351.000	0.019	0.082	14.585	0.000	15.885	0.014	9.153	0.013	0.275	0.893	0.201	0.000	0.000	0.053
TCGA.76.4925	1.000	146.000	0.000	0.000	9.324	0.000	69.436	0.057	12.307	0.000	0.000	1.824	2.795	0.000	0.000	0.000
TCGA.76.4926	1.000	138.000	0.000	0.000	8.946	0.000	18.437	0.010	10.102	0.009	0.025	1.148	2.118	0.000	0.000	0.000
TCGA.76.4927	1.000	535.000	0.071	0.000	11.564	0.034	41.250	0.017	13.819	0.000	0.000	0.885	1.209	0.000	0.000	0.000
TCGA.76.4928	1.000	94.000	0.049	0.000	12.939	0.000	39.720	0.036	22.077	0.000	0.000	0.817	0.193	0.000	0.000	0.000
TCGA.76.4929	1.000	111.000	0.017	0.000	10.057	0.024	22.930	0.012	14.974	0.000	0.186	3.035	1.912	0.000	0.000	0.048
TCGA.76.4931	1.000	279.000	0.000	0.000	12.504	0.000	39.051	0.011	8.646	0.010	0.028	1.364	2.698	0.012	0.000	0.043
TCGA.76.4932	1.000	1458.000	0.015	0.065	13.544	0.000	62.377	0.022	9.683	0.010	0.000	0.740	0.284	0.000	0.000	0.000

OS: Overall Survival.

ENSG00000228015	ENSG00000255043	ENSG00000255947	ENSG00000265559	ENSG00000233087	ENSG00000129351	ENSG00000265366	ENSG00000233030	ENSG00000279827	ENSG00000274469	ENSG00000204776	ENSG00000229795	ENSG00000241228	ENSG00000197859	ENSG00000259336	ENSG00000251877
0.000	0.007	0.000	0.082	0.291	21.723	0.555	0.175	0.072	0.000	0.000	0.000	0.000	2.974	0.037	0.000
0.000	0.000	0.000	0.070	0.165	14.444	0.409	0.065	0.082	0.000	0.000	0.341	0.047	1.588	0.016	0.000
0.000	0.011	0.000	0.000	0.196	38.023	0.560	0.169	0.128	0.000	0.000	0.076	0.083	2.767	0.014	0.000
0.000	0.000	0.000	0.141	0.250	45.189	0.222	0.054	0.041	0.000	0.000	0.601	0.000	2.885	0.047	0.113
0.151	0.000	0.000	0.000	0.185	13.978	0.247	0.240	0.023	0.000	0.000	0.191	0.000	1.822	0.035	0.000
0.000	0.000	0.000	0.187	0.219	52.033	0.836	0.505	0.091	0.000	0.000	0.378	0.124	3.661	0.056	0.000
0.000	0.000	0.000	0.280	0.055	24.044	0.283	0.356	0.061	0.000	0.000	0.340	0.047	1.819	0.000	0.000
0.000	0.000	0.000	0.074	0.290	11.073	0.398	0.270	0.022	0.000	0.064	0.000	0.099	6.445	0.000	0.000
0.000	0.007	0.056	0.000	0.168	16.089	0.844	0.210	0.175	0.057	0.000	0.208	0.000	3.375	0.019	0.000
0.000	0.000	0.000	0.000	0.391	11.342	0.223	0.127	0.073	0.000	0.429	0.303	0.000	3.371	0.000	0.000
0.000	0.010	0.040	0.000	0.096	13.849	0.247	0.508	0.054	0.000	0.000	0.297	0.041	2.179	0.041	0.000
0.000	0.000	0.000	0.063	0.373	35.932	0.256	0.263	0.037	0.000	0.000	0.308	0.127	4.852	0.028	0.102
0.000	0.006	0.000	0.065	0.231	33.862	0.293	0.122	0.095	0.000	0.000	0.238	0.087	3.241	0.000	0.000
0.000	0.010	0.000	0.000	0.216	35.958	0.219	0.181	0.143	0.000	0.000	0.297	0.041	4.388	0.014	0.098
0.000	0.000	0.000	0.000	0.427	19.360	1.099	0.115	0.000	0.000	0.000	0.000	0.000	4.386	0.000	0.000
0.000	0.005	0.000	0.122	0.144	16.299	0.247	0.492	0.018	0.000	0.000	0.297	0.000	4.192	0.000	0.000
0.000	0.000	0.000	0.071	0.221	38.356	0.285	0.054	0.041	0.000	0.000	0.086	0.000	3.752	0.000	0.000
0.000	0.000	0.000	0.000	0.329	12.597	0.789	0.369	0.000	0.000	0.000	0.000	0.112	1.016	0.037	0.000
0.000	0.000	0.000	0.000	0.100	17.815	0.172	0.275	0.056	0.043	0.000	0.000	0.042	4.346	0.000	0.000
0.000	0.017	0.000	0.000	0.187	22.757	0.214	0.427	0.099	0.000	0.000	0.331	0.091	3.619	0.000	0.000
0.000	0.000	0.000	0.073	0.228	10.769	0.586	0.092	0.064	0.000	0.000	0.353	0.000	1.062	0.000	0.000
0.000	0.019	0.000	0.000	0.560	53.600	1.145	0.121	0.044	0.000	0.000	0.365	0.050	2.543	0.000	0.000
0.000	0.006	0.000	0.073	0.547	19.197	0.461	0.261	0.086	0.000	0.000	0.267	0.000	2.292	0.000	0.118
0.000	0.000	0.000	0.000	0.171	19.248	0.336	0.048	0.036	0.000	0.054	0.000	0.000	1.634	0.000	0.000
0.000	0.006	0.000	0.000	0.191	20.807	0.218	0.171	0.000	0.000	0.000	0.253	0.000	7.570	0.000	0.111
0.139	0.025	0.000	0.000	0.142	30.730	0.551	0.251	0.127	0.000	0.000	0.088	0.048	4.770	0.000	0.000
0.000	0.007	0.000	0.245	0.256	19.065	0.476	0.339	0.262	0.000	0.141	0.397	0.054	3.219	0.000	0.000
0.000	0.012	0.000	0.000	0.282	28.141	1.033	0.219	0.126	0.000	0.000	0.524	0.000	7.117	0.000	0.000
0.000	0.000	0.047	0.143	0.056	26.627	0.450	0.103	0.063	0.000	0.000	0.174	0.000	3.126	0.032	0.115
0.000	0.037	0.000	0.000	0.170	39.994	0.517	0.067	0.084	0.000	0.000	0.175	0.192	5.026	0.032	0.116
0.000	0.010	0.039	0.059	0.163	20.358	0.425	0.085	0.138	0.000	0.000	0.144	0.000	3.887	0.000	0.000
0.119	0.016	0.000	0.000	0.171	43.608	0.307	0.110	0.109	0.042	0.000	0.453	0.083	2.407	0.014	0.100
0.000	0.012	0.000	0.072	0.170	15.737	0.648	0.129	0.127	0.000	0.000	0.176	0.000	6.367	0.000	0.000
0.000	0.012	0.000	0.139	0.219	18.778	0.844	0.236	0.041	0.000	0.000	0.423	0.046	2.910	0.016	0.000
0.000	0.000	0.000	0.294	0.462	26.400	0.726	0.187	0.086	0.000	0.000	0.357	0.000	2.313	0.000	0.000
0.000	0.011	0.000	0.067	0.288	19.918	0.329	0.322	0.019	0.000	0.000	0.162	0.044	3.934	0.000	0.000
0.000	0.022	0.000	0.318	0.325	50.063	0.485	0.469	0.056	0.000	0.055	0.541	0.085	3.210	0.100	0.000
0.000	0.011	0.000	0.191	0.100	36.183	0.399	0.592	0.037	0.000	0.055	0.232	0.085	3.450	0.000	0.000
0.000	0.006	0.000	0.152	0.238	25.792	0.170	0.167	0.066	0.051	0.000	0.276	0.000	7.342	0.000	0.000
0.000	0.007	0.000	0.000	0.184	34.880	0.422	0.106	0.000	0.000	0.000	0.666	0.052	2.941	0.018	0.000
0.000	0.027	0.000	0.156	0.092	22.622	0.455	1.063	0.068	0.000	0.000	0.000	0.000	2.711	0.052	0.000
0.000	0.006	0.043	0.198	0.181	21.335	0.532	0.167	0.058	0.000	0.000	0.320	0.000	5.385	0.029	0.000
0.000	0.019	0.000	0.073	0.171	34.619	0.228	0.080	0.000	0.000	0.000	0.352	0.097	5.308	0.016	0.116
0.000	0.000	0.000	0.124	0.268	27.755	0.670	0.274	0.073	0.042	0.000	0.227	0.000	5.597	0.056	0.000
0.000	0.011	0.000	0.000	0.208	44.297	0.744	0.073	0.058	0.000	0.000	0.403	0.044	3.084	0.030	0.000
0.000	0.000	0.000	0.129	0.203	30.105	0.522	0.252	0.038	0.000	0.000	0.393	0.000	3.602	0.029	0.000
0.000	0.000	0.000	0.142	2.207	43.629	0.735	0.042	0.042	0.000	0.000	0.433	0.000	33.755	0.048	0.000
0.000	0.012	0.000	0.000	0.198	39.555	0.873	0.287	0.063	0.000	0.000	0.088	0.048	6.465	0.000	0.000
0.000	0.000	0.000	0.000	0.319	34.479	0.617	0.132	0.055	0.042	0.000	0.076	0.042	1.640	0.000	0.000
0.000	0.005	0.000	0.000	0.347	12.658	0.106	0.495	0.034	0.000	0.000	0.215	0.118	4.089	0.000	0.000
0.000	0.000	0.000	0.000	0.792	18.409	0.399	0.231	0.104	0.000	0.000	0.216	0.040	4.482	0.000	0.000
0.491	0.005	0.041	0.000	0.300	14.975	0.200	0.205	0.112	0.000	0.000	0.310	0.043	7.177	0.014	0.000
0.000	0.017	0.000	0.065	0.331	23.775	0.146	0.468	0.076	0.000	0.000	0.316	0.043	2.576	0.000	0.000
0.000	0.005	0.000	0.115	0.315	25.551	0.206	0.073	0.117	0.000	0.000	0.209	0.000	3.467	0.013	0.000
0.000	0.036	0.000	0.106	0.166	36.212	1.328	0.090	0.093	0.000	0.000	0.128	0.000	1.357	0.000	0.000
0.000	0.000	0.000	0.000	0.648	26.536	0.865	0.350	0.000	0.000	0.000	0.335	0.000	1.715	0.000	0.000
0.000	0.006	0.000	0.068	0.457	20.234	0.399	0.093	0.020	0.000	0.000	0.499	0.000	2.161	0.000	0.000
0.124	0.000	0.042	0.065	0.228	24.786	0.377	0.329	0.094	0.000	0.000	0.079	0.086	3.466	0.000	0.000
0.000	0.011	0.000	0.000	0.580	25.875	0.260	0.310	0.056	0.000	0.000	0.078	0.000	5.498	0.000	0.103
0.000	0.000	0.000	0.120	0.353	20.596	0.511	0.147	0.088	0.000	0.000	0.146	0.000	0.743	0.000	0.000
0.000	0.006	0.000	0.277	0.299	32.603	0.591	0.270	0.081	0.000	0.000	0.253	0.046	6.796	0.015	0.000
0.000	0.000	0.000	0.110	0.431	27.400	0.345	0.065	0.064	0.000	0.000	0.267	0.000	1.722	0.000	0.000
0.000	0.005	0.037	0.115	0.337	36.554	0.385	0.073	0.000	0.000	0.000	0.556	0.000	1.499	0.000	0.000
0.000	0.000	0.000	0.184	0.169	26.626	0.193	0.702	0.072	0.000	0.000	0.671	0.041	2.765	0.000	0.099
0.132	0.006	0.000	0.000	0.322	27.208	0.338	0.197	0.060	0.000	0.000	0.249	0.046	3.491	0.000	0.000
0.000	0.000	0.000	0.000	0.172	23.887	0.355	0.045	0.051	0.000	0.000	0.214	0.059	5.387	0.020	0.000
0.000	0.000	0.000	0.000	0.378	30.092	0.541	0.041	0.070	0.000	0.000	0.000	0.000	4.766	0.027	0.000
0.000	0.007	0.052	0.000	0.158	16.272	0.325	0.007	0.047	0.000	0.000	0.391	0.000	0.846	0.000	0.000
0.206	0.009	0.000	0.000	0.126	30.573	0.048	0.199	0.062	0.071	0.000	0.779	0.000	2.263	0.024	0.000
0.000	0.007	0.000	0.000	0.265	13.238	0.227	0.014	0.099	0.000	0.000	0.103	0.000	2.746	0.019	0.000
0.000	0.005	0.000	0.000	0.314	17.328	0.221	0.104	0.018	0.000	0.000	0.075	0.082	3.068	0.000	0.000
0.000	0.000	0.000	0.124	0.293	6.431	1.283	0.105	0.036	0.000	0.000	0.000	0.166	1.309	0.056	0.000
0.000	0.000	0.000	0.056	0.354	14.611	0.303	0.191	0.082	0.000	0.000	0.137	0.000	4.016	0.050	0.000
0.000	0.009	0.000	0.111	0.131	25.939	0.449	0.151	0.097	0.000	0.000	0.135	0.000	2.573	0.025	0.000
0.000	0.000	0.000	0.000	0.066	22.545	0.190	0.122	0.025	0.000	0.000	0.412	0.057	3.485	0.000	0.000
0.000	0.009	0.000	0.306	0.200	29.019	0.275	0.138	0.060	0.000	0.000	0.372	0.000	3.027	0.046	0.000
0.137	0.000	0.000	0.000	0.168	17.057	0.128	0.085	0.021	0.000	0.000	0.520	0.095	2.688	0.032	0.000
0.000	0.037	0.000	0.000	0.672	53.916	0.576	0.943	0.000	0.000	0.000	0.000	0.285	1.236	0.000	0.000
0.000	0.000	0.000	0.000	0.141	18.913	1.129	0.137	0.131	0.000	0.000	0.437	0.000	0.739	0.000	0.000
0.000	0.000	0.000	0.000	0.106	11.682	0.483	0.084	0.052	0.000	0.000	0.109	0.000	1.237	0.020	0.000
0.000	0.014	0.000	0.165	0.258	7.813	1.143	0.077	0.048	0.000	0.071	0.300	0.055	0.781	0.000	0.000
0.000	0.000	0.000	0.064	0.152	15.906	0.318	0.071	0.056	0.000	0.000	0.078	0.000	2.015	0.072	0.000
0.000	0.007	0.000	0.000	0.120	26.916	0.481	0.318	0.045	0.000	0.000	0.279	0.000	2.888	0.000	0.000
0.000	0.000	0.000	0.087	0.204	20.007	0.233	0.257	0.051	0.000	0.000	0.000	0.000	3.764	0.000	0.000
0.000	0.012	0.000	0.000	0.199	25.045	0.390	0.147	0.021	0.000	0.000	0.176	0.000	4.236	0.000	0.000
0.148	0.000	0.000	0.000	0.211	14.747	0.483	0.104	0.067	0.000	0.066	0.280	0.051	1.076	0.000	0.000
0.000	0.016	0.000	0.000	0.403	54.956	1.140	0.154	0.124	0.000	0.000	0.220	0.000	2.550	0.068	0.000
0.121	0.000	0.000	0.252	0.099	21.819	0.453	0.476	0.129	0.000	0.000	0.077	0.042	3.591	0.000	0.000
0.000	0.010	0.000	0.115	0.362	27.920	0.465	0.083	0.017	0.000	0.000	0.280	0.000	3.850	0.039	0.000
0.216	0.019	0.000	0.000	0.308	21.782	0.151	0.200	0.033	0.000	0.000	0.682	0.000	3.378	0.000	0.000
0.000	0.010	0.000	0.420	0.235	48.738	0.591	0.086	0.017	0.000	0.000	0.728	0.080	2.754	0.000	0.000
0.119	0.000	0.000	0.186	0.218	27.750	0.777	0.162	0.090	0.000	0.000	0.376	0.000	1.362	0.028	0.000
0.000	0.005	0.000	0.000	0.187	34.809	0.429	0.521	0.070	0.000	0.000	0.290	0.080	4.398	0.027	0.000
0.000	0.016	0.000	0.000	0.221	46.524	0.703	0.191	0.091	0.000	0.000	0.533	0.042	1.913	0.000	0.000
0.000	0.021	0.000	0.000	0.213	23.576	0.840	0.174	0.018	0.000	0.000	0.293	0.081	5.235	0.000	0.097
0.000	0.000	0.046	0.070	0.220	34.190	0.409	0.220	0.061	0.000	0.000	0.511	0.047	6.855	0.016	0.000
0.000	0.013	0.000	0.294	0.173	33.767	0.296	0.037	0.086	0.000	0.000	0.178	0.049	2.581	0.016	0.000
0.000	1.049	0.000	0.898	1.526	39.459	0.134	0.101	0.524	0.000	0.000	0.727	0.998	3.188	0.134	0.000
0.000	0.000	0.000	0.190	0.595	21.196	0.935	0.690	0.055	0.000	0.000	0.000	0.000	4.012	0.000	0.304
0.000	0.000	0.000	0.221	0.173	26.645	0.792	0.181	0.021	0.000	0.000	0.357	0.000	3.383	0.000	0.000
0.000	0.000	0.000	0.073	0.144	26.105	0.657	0.590	0.086	0.000	0.000	0.178	0.098	4.693	0.016	0.000
0.184	0.000	0.000	0.000	0.150	9.826	0.472	0.178	0.084	0.000	0.000	0.116	0.128	1.019	0.000	0.000
0.000	0.000	0.000	0.062	0.098	20.533	0.697	0.337	0.091	0.000	0.000	0.302	0.083	1.747	0.000	0.000
0.000	0.000	0.000	0.000	0.220	20.543	0.324	0.373	0.117	0.000	0.000	0.097	0.000	3.086	0.090	0.000
0.000	0.008	0.000	0.092	0.036	27.612	0.206	0.132	0.107	0.000	0.000	0.558	0.000	4.399	0.021	0.000
0.000	0.014	0.000	0.000	0.379	28.063	0.072	0.232	0.000	0.000	0.000	0.587	0.000	5.611	0.036	0.000
0.142	0.019	0.000	0.000	0.087	21.202	0.397	0.518	0.065	0.000	0.000	0.090	0.000	2.105	0.033	0.000
0.000	0.006	0.000	0.000	0.180	30.623	1.262	0.061	0.096	0.000	0.000	0.000	0.000	3.401	0.000	0.000
0.000	0.005	0.000	0.000	0.217	40.199	0.799	0.109	0.108	0.000	0.000	0.224	0.000	1.982	0.027	0.000
0.000	0.000	0.000	0.000	0.151	19.713	0.311	0.078	0.067	0.000	0.000	0.000	0.051	2.267	0.000	0.000
0.000	0.000	0.000	0.000	0.171	10.306	0.195	0.055	0.085	0.000	0.000	0.000	0.097	2.412	0.000	0.000
0.000	0.017	0.000	0.068	0.294	28.553	0.489	0.029	0.040	0.000	0.000	0.165	0.045	3.767	0.030	0.000
0.000	0.006	0.000	0.000	0.221	26.208	0.379	0.238	0.021	0.000	0.000	0.513	0.000	5.256	0.000	0.000
0.000	0.013	0.000	0.000	0.296	40.814	0.541	0.115	0.088	0.000	0.000	0.183	0.000	3.549	0.000	0.000
0.000	0.006	0.000	0.000	0.225	23.824	0.803	0.200	0.042	0.000	0.000	0.174	0.048	1.047	0.000	0.000
0.000	0.000	0.000	0.076	0.030	19.776	0.273	0.135	0.133	0.000	0.000	0.000	0.000	3.678	0.000	0.000
0.126	0.050	0.000	0.066	0.592	28.109	0.265	0.044	0.000	0.000	0.000	0.239	0.000	4.806	0.015	0.000
0.000	0.000	0.000	0.000	0.477	25.966	0.477	0.116	0.044	0.000	0.000	0.000	0.000	4.034	0.000	0.000
0.000	0.000	0.000	0.000	0.362	30.295	0.532	0.039	0.058	0.000	0.000	0.160	0.000	3.615	0.015	0.000
0.000	0.005	0.000	0.125	0.320	40.513	0.225	0.096	0.073	0.000	0.000	0.381	0.167	2.859	0.056	0.000
0.000	0.000	0.000	0.057	0.334	29.775	0.356	0.293	0.166	0.000	0.000	0.207	0.000	3.714	0.025	0.000
0.000	0.000	0.000	0.000	0.234	24.251	0.498	0.065	0.050	0.000	0.000	0.000	0.000	1.807	0.038	0.000
0.000	0.006	0.000	0.000	0.117	27.217	0.335	0.070	0.065	0.000	0.000	0.182	0.050	1.291	0.033	0.000
0.000	0.006	0.000	0.074	0.320	18.046	0.466	0.673	0.065	0.000	0.000	0.000	0.050	3.007	0.033	0.000
0.134	0.012	0.000	0.209	0.219	35.601	0.250	0.207	0.122	0.000	0.000	0.254	0.046	4.267	0.047	0.000
0.000	0.000	0.000	0.066	0.391	16.739	0.805	0.220	0.097	0.000	0.000	0.242	0.000	6.398	0.000	0.000
0.000	0.000	0.000	0.000	0.255	25.208	0.292	0.202	0.063	0.000	0.000	0.527	0.145	7.440	0.048	0.000
0.110	0.005	0.037	0.000	0.157	29.786	0.360	0.806	0.033	0.000	0.000	0.557	0.038	3.121	0.026	0.092
0.000	0.000	0.069	0.000	0.229	22.837	0.572	0.126	0.186	0.000	0.000	0.194	0.035	1.640	0.024	0.000
0.000	0.010	0.037	0.115	0.271	44.117	1.109	0.380	0.168	0.038	0.000	0.140	0.000	4.394	0.039	0.092
0.000	0.005	0.000	0.109	0.193	29.948	0.123	0.079	0.032	0.000	0.000	0.199	0.000	5.034	0.000	0.000
0.000	0.074	0.000	0.689	0.744	14.722	0.232	0.088	0.101	0.000	0.000	0.209	0.000	4.820	0.000	0.000
0.000	0.000	0.000	0.000	0.391	20.674	0.475	0.074	0.018	0.000	0.000	0.076	0.000	1.805	0.042	0.000
0.000	0.068	0.000	0.320	0.345	32.068	0.538	0.257	0.187	0.000	0.000	0.486	0.000	5.172	0.000	0.000
0.313	0.007	0.000	0.081	0.128	25.823	0.183	0.028	0.048	0.000	0.000	0.198	0.109	3.251	0.000	0.000
0.000	0.008	0.000	0.181	0.355	21.530	0.609	0.253	0.106	0.000	0.000	0.220	0.000	3.969	0.000	0.000
0.000	0.000	0.000	0.102	0.279	20.981	0.547	0.602	0.059	0.000	0.000	0.370	0.135	4.413	0.023	0.000
0.000	0.021	0.054	0.083	0.194	50.381	0.666	0.189	0.072	0.000	0.000	0.200	0.055	4.065	0.000	0.000
0.000	0.025	0.000	0.115	0.721	31.541	0.619	0.263	0.084	0.000	0.000	0.279	0.038	1.825	0.000	0.000
0.227	0.005	0.000	0.059	0.532	23.181	0.608	0.165	0.017	0.000	0.000	0.000	0.000	2.220	0.026	0.000
0.202	0.000	0.000	0.000	0.413	29.995	0.236	0.116	0.061	0.000	0.000	0.000	0.140	7.539	0.000	0.000
0.000	0.005	0.000	0.110	0.194	23.461	0.320	0.218	0.064	0.000	0.000	0.200	0.037	2.072	0.000	0.000
0.000	0.013	0.000	0.149	0.291	22.670	0.966	0.302	0.173	0.000	0.000	0.000	0.000	2.005	0.017	0.000
0.000	0.011	0.000	0.133	0.183	42.336	0.090	0.266	0.117	0.000	0.000	0.081	0.044	3.600	0.000	0.000
0.000	0.000	0.000	0.066	0.385	25.387	0.235	0.105	0.019	0.000	0.000	0.080	0.000	3.118	0.015	0.000

ENSG00000101400	ENSG00000112282	ENSG00000119242	ENSG00000154198	ENSG00000070759	ENSG00000234186	ENSG00000074276	ENSG00000261648	ENSG00000258529	ENSG00000263485	ENSG00000273725	ENSG00000203782	ENSG00000280121	ENSG00000260488	ENSG00000035928	ENSG00000254707
25.553	3.306	6.609	0.000	1.281	0.000	0.227	0.000	0.000	0.000	0.000	0.020	0.000	0.000	9.136	0.000
16.307	3.418	4.079	0.013	1.641	0.171	0.071	0.000	0.000	0.000	0.000	0.017	0.000	0.000	6.946	0.000
5.878	3.017	5.633	0.000	0.970	0.000	0.592	0.000	0.000	0.000	0.000	0.015	0.000	0.000	8.674	0.000
65.701	3.696	7.524	0.000	0.894	0.009	0.448	0.000	0.006	0.000	0.000	0.000	0.000	0.000	8.297	0.000
37.115	2.640	8.329	0.000	2.224	0.000	0.031	0.000	0.000	0.000	0.000	0.000	0.000	0.039	7.390	0.000
6.331	8.460	3.552	0.000	2.381	0.000	0.711	0.000	0.000	0.000	0.000	0.045	0.000	0.031	12.289	0.035
6.600	3.632	7.215	0.013	1.460	0.000	0.326	0.000	0.000	0.000	0.000	0.034	0.000	0.000	9.723	0.000
36.585	2.335	14.118	0.014	2.080	0.009	0.336	0.000	0.000	0.000	0.000	0.054	0.000	0.000	5.050	0.000
33.474	2.698	5.900	0.000	1.901	0.000	0.139	0.000	0.000	0.000	0.000	0.021	0.000	0.000	5.013	0.000
6.796	3.307	4.316	0.000	2.204	0.000	0.168	0.000	0.000	0.000	0.000	0.000	0.000	0.000	3.907	0.000
39.439	2.695	5.946	0.011	1.719	0.022	0.141	0.000	0.000	0.000	0.000	0.030	0.000	0.092	5.727	0.000
19.962	4.164	8.759	0.000	0.961	0.000	0.601	0.000	0.000	0.000	0.067	0.015	0.000	0.032	10.299	0.000
44.737	3.654	5.812	0.000	1.429	0.008	0.216	0.000	0.000	0.000	0.000	0.000	0.037	0.000	9.245	0.000
45.018	4.963	8.671	0.000	2.203	0.000	0.319	0.000	0.000	0.000	0.064	0.015	0.035	0.000	9.611	0.000
71.081	2.745	9.223	0.000	2.066	0.000	0.351	0.000	0.000	0.000	0.000	0.066	0.000	0.136	8.071	0.000
30.014	3.814	5.809	0.000	2.387	0.000	0.134	0.000	0.005	0.000	0.000	0.000	0.000	0.031	8.431	0.000
12.881	3.359	5.502	0.000	0.848	0.000	0.325	0.000	0.000	0.000	0.000	0.034	0.000	0.035	8.190	0.000
7.329	1.760	4.496	0.000	1.732	0.000	0.075	0.000	0.000	0.000	0.000	0.102	0.000	0.000	2.650	0.000
39.848	2.353	5.240	0.000	1.934	0.000	0.046	0.000	0.000	0.000	0.000	0.000	0.000	0.064	7.840	0.000
51.659	1.790	6.439	0.000	1.385	0.000	0.130	0.000	0.000	0.000	0.000	0.000	0.000	0.000	7.696	0.000
20.920	2.223	5.561	0.000	2.919	0.000	0.155	0.000	0.000	0.000	0.000	0.071	0.000	0.000	3.584	0.000
26.916	5.757	11.132	0.000	2.245	0.000	2.839	0.000	0.000	0.075	0.000	0.018	0.000	0.037	12.307	0.000
17.737	2.599	6.524	0.000	1.816	0.000	0.375	0.000	0.000	0.000	0.077	0.018	0.000	0.000	5.988	0.000
14.458	2.201	5.710	0.000	0.953	0.023	0.165	0.000	0.000	0.000	0.000	0.000	0.000	0.062	7.334	0.000
23.129	2.917	6.851	0.000	1.895	0.000	0.199	0.000	0.000	0.000	0.000	0.000	0.039	0.000	6.029	0.000
14.580	3.589	6.322	0.000	1.286	0.000	0.657	0.000	0.000	0.000	0.000	0.106	0.041	0.036	8.203	0.000
30.592	3.497	5.916	0.000	2.768	0.000	0.271	0.000	0.007	0.000	0.000	0.080	0.000	0.082	7.681	0.000
20.832	2.697	10.123	0.000	1.729	0.000	0.404	0.000	0.000	0.000	0.000	0.000	0.000	0.000	7.583	0.000
78.867	3.194	9.539	0.000	1.858	0.000	0.137	0.000	0.000	0.000	0.000	0.035	0.000	0.036	7.085	0.000
22.124	3.417	6.972	0.014	1.250	0.000	0.202	0.000	0.000	0.000	0.000	0.053	0.000	0.000	6.771	0.000
18.761	2.693	7.318	0.000	0.894	0.000	0.170	0.000	0.000	0.000	0.062	0.072	0.000	0.000	6.622	0.000
20.098	4.150	7.247	0.000	1.728	0.000	0.199	0.000	0.000	0.000	0.000	0.061	0.000	0.031	9.150	0.000
78.043	2.364	17.340	0.000	2.723	0.000	0.454	0.000	0.000	0.000	0.000	0.194	0.000	0.000	5.858	0.000
27.901	1.486	8.437	0.000	1.516	0.000	0.238	0.039	0.000	0.000	0.073	0.000	0.000	0.000	6.237	0.000
15.439	4.223	6.180	0.000	1.608	0.000	0.083	0.000	0.000	0.000	0.000	0.000	0.000	0.000	10.318	0.000
90.287	3.319	9.049	0.000	2.143	0.000	0.277	0.000	0.000	0.000	0.000	0.000	0.000	0.000	8.308	0.000
72.164	4.432	10.243	0.000	3.179	0.070	0.900	0.000	0.000	0.000	0.000	0.000	0.000	0.064	11.679	0.000
49.120	3.116	6.704	0.000	2.268	0.016	0.235	0.000	0.000	0.000	0.000	0.000	0.000	0.063	8.683	0.000
27.973	4.436	7.463	0.000	1.603	0.000	0.438	0.000	0.000	0.000	0.000	0.000	0.000	0.000	9.359	0.000
14.224	5.586	4.692	0.000	1.649	0.000	0.338	0.000	0.007	0.000	0.000	0.057	0.000	0.000	11.732	0.000
20.216	3.244	11.951	0.015	1.613	0.000	0.363	0.000	0.000	0.000	0.000	0.114	0.044	0.000	9.942	0.000
25.435	4.121	6.884	0.000	1.036	0.016	0.251	0.000	0.012	0.000	0.069	0.016	0.000	0.033	8.626	0.000
20.390	5.298	8.196	0.014	1.146	0.000	0.085	0.000	0.000	0.000	0.000	0.000	0.000	0.000	9.214	0.040
24.310	5.273	6.481	0.000	1.478	0.000	0.272	0.000	0.000	0.000	0.000	0.030	0.000	0.000	12.445	0.000
16.746	6.279	6.400	0.012	1.234	0.000	0.182	0.000	0.006	0.000	0.000	0.000	0.000	0.000	11.516	0.000
69.855	4.071	4.815	0.000	3.616	0.000	0.334	0.000	0.000	0.000	0.000	0.000	0.000	0.000	12.055	0.000
14.436	11.190	3.142	0.013	1.801	0.000	0.048	0.000	0.000	0.000	0.000	0.017	0.121	0.000	11.722	0.079
9.115	5.048	7.646	0.000	2.637	0.000	0.291	0.000	0.000	0.000	0.076	0.106	0.000	0.036	9.086	0.040
12.621	2.652	7.116	0.000	1.265	0.000	0.238	0.000	0.000	0.000	0.000	0.015	0.000	0.000	13.270	0.000
12.946	2.069	7.131	0.000	2.863	0.000	0.103	0.000	0.000	0.000	0.000	0.029	0.033	0.000	6.872	0.000
33.589	3.104	12.571	0.022	2.509	0.007	0.649	0.000	0.000	0.000	0.000	0.159	0.000	0.030	6.046	0.000
14.571	2.762	7.010	0.000	0.945	0.000	0.090	0.000	0.000	0.000	0.067	0.000	0.000	0.032	7.865	0.000
18.858	2.394	6.670	0.000	1.649	0.000	0.339	0.000	0.000	0.000	0.000	0.000	0.000	0.000	10.541	0.000
19.890	3.799	6.765	0.000	1.204	0.000	0.116	0.000	0.005	0.000	0.000	0.028	0.000	0.000	10.097	0.000
6.532	1.726	8.001	0.000	1.423	0.013	0.932	0.000	0.000	0.000	0.000	0.026	0.000	0.000	7.498	0.059
7.660	6.725	4.906	0.000	1.194	0.000	0.093	0.000	0.000	0.000	0.000	0.000	0.000	0.000	18.620	0.000
26.794	4.194	5.295	0.000	1.218	0.000	0.257	0.000	0.000	0.000	0.000	0.000	0.000	0.000	8.809	0.000
17.832	3.162	6.620	0.000	1.578	0.016	0.385	0.000	0.000	0.000	0.000	0.000	0.000	0.032	13.798	0.000
42.963	4.737	5.914	0.000	1.992	0.000	0.119	0.000	0.000	0.000	0.000	0.000	0.000	0.000	14.276	0.000
52.193	2.620	6.262	0.000	2.270	0.000	0.326	0.000	0.000	0.000	0.000	0.000	0.000	0.000	12.067	0.000
86.080	4.515	6.314	0.000	1.822	0.288	0.591	0.000	0.000	0.000	0.000	0.000	0.000	0.000	9.469	0.000
19.488	2.578	5.571	0.000	0.745	0.000	0.475	0.000	0.000	0.000	0.000	0.000	0.000	0.000	7.418	0.000
19.243	4.453	6.509	0.000	0.853	0.000	0.832	0.000	0.000	0.000	0.060	0.028	0.000	0.057	9.705	0.032
28.469	5.635	5.715	0.000	1.980	0.000	0.407	0.000	0.000	0.000	0.000	0.015	0.000	0.092	9.016	0.034
35.747	4.330	4.595	0.000	1.082	0.000	0.088	0.000	0.000	0.000	0.000	0.033	0.000	0.000	11.236	0.000
56.558	1.061	6.298	0.000	3.542	0.000	0.587	0.000	0.000	0.000	0.000	0.086	0.000	0.088	5.891	0.049
15.173	2.753	5.945	0.000	1.394	0.000	0.189	0.000	0.000	0.000	0.000	0.000	0.000	0.000	6.587	0.000
24.887	2.298	3.999	0.000	0.857	0.000	0.041	0.000	0.000	0.000	0.000	0.000	0.000	0.000	5.348	0.000
45.099	2.999	8.277	0.000	1.236	0.013	0.054	0.000	0.000	0.000	0.000	0.000	0.000	0.000	10.105	0.000
16.400	2.307	6.843	0.000	0.854	0.000	0.062	0.000	0.008	0.000	0.000	0.000	0.000	0.000	5.718	0.000
31.744	1.736	6.283	0.000	0.884	0.000	0.435	0.000	0.000	0.000	0.000	0.015	0.000	0.000	8.360	0.000
51.431	1.279	7.177	0.000	2.966	0.000	0.168	0.000	0.000	0.000	0.000	0.061	0.000	0.062	3.325	0.000
31.831	2.602	6.918	0.000	0.856	0.000	0.098	0.000	0.000	0.000	0.000	0.014	0.000	0.000	7.390	0.000
37.185	2.164	10.076	0.000	1.586	0.000	0.193	0.000	0.000	0.000	0.000	0.027	0.000	0.000	9.819	0.000
12.890	3.301	6.755	0.000	1.646	0.000	0.124	0.000	0.000	0.000	0.000	0.000	0.000	0.000	8.982	0.000
69.730	4.300	6.365	0.000	1.504	0.000	0.092	0.000	0.000	0.000	0.000	0.000	0.000	0.000	9.390	0.000
28.874	2.306	4.678	0.000	2.179	0.000	0.060	0.000	0.000	0.000	0.000	0.035	0.000	0.036	4.601	0.000
12.232	7.106	3.265	0.000	2.204	0.000	0.096	0.000	0.000	0.000	0.000	0.000	0.000	0.000	12.879	0.000
46.366	4.616	3.620	0.000	1.259	0.000	0.056	0.000	0.000	0.000	0.000	0.044	0.000	0.000	4.993	0.000
33.412	3.405	4.483	0.000	1.223	0.000	0.081	0.000	0.000	0.000	0.000	0.000	0.000	0.045	5.155	0.000
13.592	2.690	2.911	0.000	2.102	0.000	0.005	0.000	0.000	0.000	0.000	0.000	0.000	0.000	3.815	0.000
43.981	2.991	8.117	0.000	1.555	0.000	0.051	0.000	0.000	0.000	0.000	0.000	0.073	0.000	7.704	0.000
67.176	2.222	7.652	0.000	2.172	0.000	0.481	0.000	0.000	0.000	0.081	0.000	0.000	0.000	8.203	0.000
25.971	2.800	5.822	0.000	2.834	0.000	0.073	0.000	0.000	0.000	0.000	0.000	0.000	0.043	9.940	0.000
42.790	3.072	8.372	0.000	3.629	0.018	0.516	0.000	0.000	0.000	0.000	0.000	0.041	0.000	11.576	0.000
14.685	2.849	7.368	0.000	0.549	0.000	0.168	0.000	0.000	0.000	0.000	0.038	0.000	0.000	8.190	0.000
12.030	4.565	6.660	0.000	1.454	0.000	0.828	0.000	0.000	0.000	0.064	0.030	0.000	0.060	12.009	0.000
49.026	3.446	5.865	0.000	2.098	0.000	0.747	0.000	0.000	0.000	0.000	0.000	0.036	0.000	12.918	0.000
33.468	2.361	7.147	0.000	0.911	0.000	0.414	0.000	0.000	0.000	0.000	0.014	0.000	0.000	10.403	0.000
66.388	2.714	8.038	0.000	3.205	0.000	0.170	0.000	0.000	0.000	0.000	0.027	0.063	0.000	8.758	0.000
18.701	5.273	8.388	0.000	0.763	0.000	1.107	0.000	0.000	0.000	0.000	0.058	0.000	0.000	7.800	0.067
48.810	2.636	7.692	0.000	1.671	0.008	0.160	0.000	0.000	0.000	0.065	0.015	0.000	0.000	8.229	0.000
6.855	1.999	12.112	0.000	1.230	0.000	0.251	0.000	0.000	0.000	0.063	0.000	0.000	0.000	9.887	0.033
13.165	3.242	7.062	0.000	1.239	0.000	1.255	0.000	0.000	0.000	0.000	0.031	0.000	0.000	19.357	0.000
32.749	3.990	8.113	0.011	1.926	0.007	0.183	0.000	0.000	0.061	0.000	0.044	0.000	0.000	9.522	0.034
71.700	3.619	6.458	0.000	2.038	0.009	0.271	0.000	0.000	0.070	0.000	0.017	0.000	0.035	11.594	0.000
13.817	4.615	5.567	0.000	0.411	0.000	0.165	0.000	0.000	0.000	0.077	0.000	0.000	0.073	11.311	0.082
13.347	3.963	8.367	0.000	1.676	0.000	0.487	0.000	0.000	0.000	0.000	0.292	0.000	0.299	8.831	0.000
56.175	4.948	5.158	0.000	1.403	0.000	0.276	0.000	0.000	0.000	0.000	0.046	0.000	0.000	9.376	0.105
26.301	3.799	8.194	0.000	1.050	0.000	0.157	0.000	0.000	0.000	0.000	0.000	0.000	0.037	9.343	0.000
23.326	4.246	8.585	0.014	1.245	0.009	0.234	0.000	0.000	0.000	0.000	0.018	0.000	0.037	9.707	0.000
35.821	3.716	2.106	0.000	1.349	0.000	0.005	0.000	0.000	0.000	0.000	0.023	0.000	0.048	4.236	0.000
27.511	2.044	4.779	0.000	1.972	0.000	0.722	0.000	0.000	0.000	0.000	0.030	0.035	0.000	8.177	0.000
36.263	3.686	5.812	0.000	1.542	0.000	0.144	0.000	0.000	0.000	0.000	0.000	0.000	0.040	11.308	0.045
86.120	3.624	9.234	0.000	1.835	0.000	0.129	0.000	0.000	0.000	0.000	0.000	0.052	0.000	9.532	0.000
18.655	3.469	7.441	0.000	1.077	0.000	0.651	0.000	0.000	0.000	0.000	0.039	0.000	0.080	4.593	0.000
34.848	2.639	5.822	0.000	1.152	0.000	0.786	0.000	0.000	0.000	0.000	0.018	0.000	0.037	9.254	0.000
14.354	3.579	10.015	0.000	2.152	0.000	0.070	0.000	0.000	0.000	0.000	0.016	0.037	0.000	8.939	0.036
10.326	2.820	6.918	0.000	0.710	0.015	0.438	0.000	0.000	0.000	0.000	0.000	0.000	0.000	8.998	0.034
18.105	1.309	6.316	0.000	0.925	0.009	0.259	0.000	0.000	0.000	0.000	0.056	0.000	0.000	5.315	0.000
156.580	0.999	5.622	0.000	1.527	0.000	0.049	0.000	0.000	0.000	0.000	0.018	0.000	0.000	5.956	0.000
35.352	3.604	5.883	0.013	0.800	0.000	0.153	0.000	0.000	0.000	0.000	0.000	0.000	0.068	11.088	0.000
55.669	3.234	9.219	0.000	2.288	0.000	0.154	0.000	0.000	0.000	0.074	0.034	0.000	0.035	9.009	0.000
16.958	3.674	8.092	0.000	1.675	0.000	0.592	0.000	0.000	0.000	0.159	0.000	0.000	0.000	8.652	0.000
55.203	2.766	5.307	0.000	1.028	0.061	0.233	0.000	0.006	0.000	0.000	0.017	0.000	0.000	10.242	0.000
32.667	2.493	6.715	0.000	0.989	0.000	0.115	0.000	0.000	0.000	0.000	0.000	0.043	0.038	7.073	0.000
12.152	3.404	10.919	0.000	0.788	0.000	0.383	0.000	0.000	0.000	0.000	0.048	0.000	0.000	8.594	0.000
210.731	4.095	9.544	0.000	2.044	0.000	0.507	0.000	0.000	0.000	0.000	0.037	0.000	0.038	11.993	0.000
10.419	4.138	6.348	0.000	0.750	0.008	0.244	0.000	0.000	0.000	0.000	0.016	0.000	0.000	11.452	0.000
20.061	2.256	6.578	0.000	1.030	0.000	0.204	0.000	0.000	0.000	0.000	0.000	0.000	0.000	13.017	0.000
14.369	3.507	5.611	0.000	1.347	0.014	0.296	0.000	0.010	0.000	0.060	0.069	0.000	0.000	12.551	0.000
14.099	2.761	5.807	0.000	1.349	0.000	0.192	0.000	0.000	0.000	0.000	0.021	0.000	0.000	9.823	0.000
11.615	4.451	5.456	0.000	0.912	0.046	0.353	0.000	0.000	0.000	0.000	0.018	0.000	0.000	7.098	0.000
32.354	1.921	8.095	0.000	1.275	0.000	0.058	0.000	0.000	0.000	0.000	0.036	0.000	0.000	9.804	0.083
59.159	4.064	7.912	0.000	2.469	0.000	0.583	0.000	0.000	0.000	0.000	0.068	0.000	0.070	11.259	0.000
33.519	2.417	15.665	0.012	2.666	0.008	0.399	0.000	0.000	0.000	0.000	0.049	0.000	0.033	6.342	0.037
14.357	3.878	5.979	0.000	1.292	0.000	0.195	0.000	0.000	0.000	0.000	0.000	0.000	0.000	7.286	0.000
27.708	2.494	8.780	0.000	2.065	0.000	0.209	0.000	0.000	0.000	0.000	0.014	0.000	0.000	8.360	0.000
75.887	3.087	5.733	0.000	2.242	0.026	0.301	0.000	0.005	0.000	0.056	0.000	0.000	0.027	12.338	0.000
41.682	4.770	5.750	0.000	2.291	0.021	0.891	0.000	0.000	0.000	0.000	0.042	0.000	0.029	14.488	0.000
30.244	4.468	7.166	0.000	2.180	0.007	0.101	0.000	0.000	0.000	0.000	0.027	0.000	0.055	9.424	0.000
22.631	2.163	5.483	0.000	0.700	0.000	0.068	0.000	0.000	0.000	0.000	0.000	0.000	0.000	6.108	0.000
18.801	3.070	5.334	0.023	1.333	0.000	0.052	0.000	0.000	0.000	0.000	0.015	0.000	0.000	8.209	0.000
28.300	1.797	8.527	0.000	3.438	0.010	1.665	0.000	0.000	0.000	0.000	0.020	0.000	0.000	4.522	0.000
35.636	1.920	7.295	0.000	1.288	0.000	0.069	0.000	0.000	0.000	0.000	0.000	0.000	0.000	6.926	0.000
37.827	3.753	6.469	0.000	1.797	0.011	0.076	0.000	0.000	0.000	0.095	0.000	0.000	0.045	10.638	0.000
49.528	2.739	9.743	0.000	1.285	0.000	0.388	0.000	0.000	0.000	0.107	0.074	0.000	0.254	9.441	0.000
14.618	3.333	9.254	0.000	1.483	0.000	0.667	0.000	0.000	0.000	0.000	0.302	0.047	0.000	11.715	0.046
62.585	2.183	7.375	0.000	0.665	0.000	0.123	0.000	0.000	0.000	0.000	0.000	0.000	0.029	9.358	0.000
72.308	3.596	9.791	0.000	1.395	0.000	0.331	0.000	0.000	0.000	0.000	0.029	0.000	0.000	10.373	0.033
29.678	2.389	8.337	0.000	1.853	0.000	0.207	0.000	0.000	0.000	0.000	0.026	0.059	0.000	9.446	0.000
12.179	3.103	6.197	0.000	0.862	0.020	0.169	0.000	0.000	0.000	0.000	0.027	0.000	0.055	4.438	0.031
30.478	2.263	7.478	0.014	1.985	0.000	0.367	0.000	0.000	0.000	0.000	0.036	0.000	0.037	10.026	0.000
36.812	2.539	9.355	0.000	1.615	0.000	0.816	0.000	0.000	0.000	0.000	0.016	0.000	0.067	9.792	0.000
33.518	2.752	6.300	0.000	1.367	0.000	0.107	0.000	0.000	0.000	0.000	0.000	0.000	0.000	10.274	0.000

ENSG00000220924	ENSG00000117682	ENSG00000137218	ENSG00000211958
0.055	6.939	2.442	0.000
0.234	7.949	2.459	0.049
0.083	6.754	4.103	0.000
0.047	7.959	3.630	0.000
0.263	7.659	2.486	0.000
0.208	8.619	3.792	0.000
0.327	12.122	2.542	0.000
0.198	5.971	3.451	0.000
0.000	6.913	3.144	0.000
0.666	6.516	2.065	0.000
0.164	8.261	1.869	0.000
0.169	10.127	3.644	0.000
0.131	8.781	3.190	0.000
0.041	7.846	3.985	0.000
0.182	4.922	3.374	0.000
0.082	5.413	2.255	0.000
0.141	8.595	4.207	0.000
0.112	5.901	3.781	0.000
0.043	11.407	2.536	0.000
0.091	13.324	2.547	0.000
0.243	6.313	2.333	0.000
0.000	7.529	6.417	0.000
0.098	6.058	3.666	0.052
0.334	10.816	1.605	0.000
0.093	8.085	2.750	0.000
0.048	8.202	4.096	0.000
0.055	7.593	2.574	0.058
0.288	7.846	5.892	0.000
0.000	6.486	3.243	0.000
0.096	7.257	4.858	0.000
0.119	4.202	5.795	0.000
0.083	7.012	5.081	0.000
0.048	6.167	4.789	0.000
0.233	8.849	3.496	0.000
0.590	7.708	2.441	0.000
0.267	9.713	3.387	0.000
0.043	7.819	4.137	0.000
0.170	10.740	3.591	0.134
0.152	7.978	4.148	0.000
0.105	8.129	2.780	0.000
0.052	5.213	2.079	0.055
0.220	9.631	2.954	0.000
0.048	8.798	3.091	0.000
0.125	7.498	2.581	0.000
0.133	8.715	3.453	0.000
0.173	8.400	4.074	0.000
0.095	11.186	2.056	0.000
0.289	7.011	4.261	0.000
0.125	10.064	3.760	0.000
0.316	7.139	2.187	0.042
0.159	6.814	7.970	0.000
0.426	7.662	2.627	0.000
0.217	7.955	2.593	0.000
0.077	7.948	2.370	0.000
0.212	7.016	3.853	0.000
0.552	9.115	3.818	0.000
0.137	8.390	3.329	0.000
0.216	9.395	1.963	0.000
0.086	7.232	2.936	0.000
0.160	7.522	2.979	0.000
0.000	11.701	5.378	0.000
0.368	6.971	3.637	0.000
0.115	7.074	7.521	0.000
0.082	13.824	3.874	0.043
0.137	7.211	3.140	0.000
0.118	7.972	4.874	0.000
0.000	7.869	4.241	0.000
0.322	7.075	2.777	0.000
0.357	10.105	3.838	0.000
0.282	10.092	2.057	0.000
0.206	7.438	2.998	0.000
0.582	6.263	2.338	0.263
0.113	7.063	1.983	0.000
0.149	8.764	3.453	0.000
0.170	7.630	1.922	0.000
0.068	6.971	2.670	0.000
0.334	8.509	2.751	0.000
0.572	9.297	3.776	0.000
0.240	6.230	2.087	0.000
0.000	7.655	1.092	0.000
0.440	5.000	0.984	0.232
0.172	5.815	1.625	0.000
0.051	6.388	4.003	0.000
0.058	8.258	2.366	0.000
0.097	6.833	4.143	0.000
0.103	6.921	2.673	0.000
0.162	8.285	6.079	0.000
0.127	11.152	2.465	0.000
0.000	7.449	4.036	0.000
0.075	6.635	3.159	0.000
0.120	7.148	5.557	0.000
0.124	7.974	4.106	0.000
0.120	8.149	4.477	0.000
0.042	9.208	2.743	0.000
0.121	6.264	4.528	0.000
0.140	8.525	4.370	0.000
0.098	10.923	3.700	0.000
0.200	6.019	4.149	0.000
0.253	7.783	4.914	0.000
0.197	7.367	2.880	0.000
0.049	7.129	2.970	0.000
0.256	3.634	1.025	0.000
0.125	5.952	1.933	0.000
0.161	7.878	2.710	0.000
0.123	7.654	3.802	0.000
0.108	8.481	3.213	0.000
0.099	8.713	3.158	0.000
0.131	6.765	4.507	0.000
0.041	8.528	4.328	0.000
0.103	7.561	2.174	0.000
0.243	7.188	1.280	0.000
0.046	8.313	2.439	0.000
0.047	8.094	3.657	0.000
0.403	8.875	3.213	0.000
0.191	11.193	2.098	0.000
0.000	7.376	1.828	0.000
0.044	5.033	3.003	0.000
0.203	8.654	3.277	0.000
0.308	8.078	3.093	0.046
0.168	8.927	2.863	0.000
0.152	8.020	3.328	0.040
0.228	8.538	3.021	0.000
0.250	4.905	1.531	0.158
0.496	8.260	1.957	0.000
0.140	9.622	3.074	0.000
0.178	7.979	5.082	0.000
0.435	6.381	3.413	0.000
0.191	10.152	4.109	0.000
0.071	10.037	2.254	0.000
0.231	5.440	5.464	0.000
0.000	7.943	3.614	0.000
0.345	6.542	2.207	0.000
0.333	7.488	2.340	0.000
0.321	8.658	8.867	0.000
0.327	7.649	2.942	0.000
0.181	7.270	2.991	0.000
0.204	7.648	2.528	0.000
0.165	7.268	5.102	0.000
0.269	10.563	2.034	0.000
0.236	6.792	2.587	0.000
0.352	7.496	2.474	0.000
0.183	7.434	2.112	0.000
0.099	8.750	5.994	0.000
0.312	10.522	4.336	0.000
0.000	20.518	2.964	0.000

Table S3. Expression data (in FPKM) from 144 patients with glioblastoma and 500 genes from TCGA.

sample	OS	OS.time	ENSG00000231058	ENSG00000275127	ENSG00000104529	ENSG00000258728	ENSG00000135372	ENSG00000198569	ENSG00000120729	ENSG00000261395	ENSG00000223624	ENSG00000163682	ENSG00000170099	ENSG00000259030	ENSG00000189366	ENSG00000223982
TCGA.02.0047	1.000	448.000	0.000	0.000	7.119	0.000	6.006	0.161	1.240	0.364	0.354	80.855	0.080	0.079	0.793	0.000
TCGA.02.0055	1.000	76.000	0.000	0.000	13.132	0.000	7.238	0.064	0.291	0.387	0.150	327.347	0.045	0.063	0.000	0.000
TCGA.02.2483	0.000	466.000	0.000	0.000	27.696	0.000	11.981	0.049	0.272	0.344	0.803	363.848	0.020	0.032	0.622	0.000
TCGA.02.2485	0.000	470.000	0.000	0.000	11.477	0.000	13.102	0.203	0.059	0.156	0.455	149.144	0.000	0.113	0.881	0.000
TCGA.02.2486	1.000	493.000	0.000	0.257	10.725	0.000	9.248	0.082	0.212	0.260	0.337	267.012	0.000	0.020	0.224	0.000
TCGA.06.0129	1.000	989.000	0.000	0.000	13.080	0.000	13.500	0.340	2.469	0.000	0.666	94.965	0.000	0.128	1.062	0.000
TCGA.06.0130	1.000	320.000	0.000	0.000	8.848	0.023	10.497	0.182	0.203	0.463	0.150	172.895	0.057	0.031	0.125	0.000
TCGA.06.0132	1.000	570.000	0.000	0.000	7.379	0.000	7.505	0.029	1.643	0.163	0.000	233.771	0.000	0.040	2.398	0.000
TCGA.06.0138	1.000	674.000	0.000	0.000	6.979	0.000	5.475	0.056	1.304	0.189	0.733	282.268	0.000	0.033	3.167	0.000
TCGA.06.0139	1.000	327.000	0.000	0.000	7.321	0.000	5.702	0.000	0.620	0.824	2.135	268.804	0.000	0.040	0.000	0.000
TCGA.06.0141	1.000	280.000	0.000	0.000	5.600	0.020	7.330	0.072	1.193	0.337	0.393	175.149	0.000	0.104	1.024	0.000
TCGA.06.0152	1.000	359.000	0.014	0.000	10.042	0.000	12.199	0.157	0.085	0.140	0.000	84.985	0.000	0.061	1.736	0.000
TCGA.06.0157	1.000	97.000	0.000	0.000	6.541	0.000	11.013	0.051	0.020	0.433	0.420	82.944	0.000	0.042	0.814	0.000
TCGA.06.0158	1.000	166.000	0.000	0.000	11.082	0.020	11.769	0.438	0.292	0.202	0.524	64.511	0.000	0.051	0.674	0.000
TCGA.06.0168	1.000	579.000	0.000	0.000	9.017	0.000	11.054	0.106	0.311	0.601	2.335	135.714	0.000	0.017	0.388	0.000
TCGA.06.0171	1.000	396.000	0.000	0.000	7.007	0.000	8.965	0.151	0.444	0.067	0.131	76.338	0.020	0.045	0.348	0.000
TCGA.06.0174	1.000	67.000	0.000	0.230	10.357	0.023	10.577	0.037	0.300	0.233	0.151	128.007	0.000	0.059	0.628	0.064
TCGA.06.0178	1.000	1642.000	0.000	0.000	8.503	0.000	6.084	0.011	1.042	0.277	0.898	847.948	0.000	0.038	2.862	0.000
TCGA.06.0184	1.000	1228.000	0.000	0.000	10.904	0.021	6.848	0.133	0.899	0.281	0.273	233.361	0.021	0.084	2.223	0.000
TCGA.06.0187	1.000	801.000	0.000	0.000	7.254	0.000	11.704	0.142	0.064	0.451	0.292	86.129	0.011	0.035	0.776	0.000
TCGA.06.0219	1.000	11.000	0.000	0.237	6.794	0.024	6.523	0.047	1.062	0.561	0.311	449.784	0.000	0.021	0.103	0.000
TCGA.06.0221	1.000	548.000	0.016	0.000	7.332	0.000	14.617	0.518	0.459	0.166	1.448	65.965	0.000	0.048	0.641	0.000
TCGA.06.0238	1.000	359.000	0.000	0.000	7.907	0.000	7.714	0.076	2.777	0.486	0.472	343.512	0.024	0.040	0.836	0.000
TCGA.06.0644	1.000	375.000	0.000	0.000	8.476	0.000	11.257	0.041	0.168	0.207	0.535	131.215	0.040	0.012	0.533	0.000
TCGA.06.0645	1.000	98.000	0.015	0.000	9.162	0.000	9.988	0.091	0.252	0.153	0.447	159.284	0.023	0.031	0.989	0.000
TCGA.06.0646	1.000	136.000	0.000	0.000	12.124	0.000	14.993	0.668	0.570	0.319	0.465	64.143	0.000	0.058	0.592	0.000
TCGA.06.0649	1.000	62.000	0.018	0.000	7.962	0.000	8.855	0.341	2.458	0.361	0.525	175.566	0.119	0.060	1.280	0.000
TCGA.06.0686	1.000	286.000	0.032	0.000	10.359	0.000	5.625	0.131	1.149	0.476	0.308	169.454	0.000	0.028	0.666	0.000
TCGA.06.0743	1.000	679.000	0.000	0.000	11.420	0.000	12.548	0.243	0.639	0.237	0.461	111.405	0.012	0.032	2.372	0.000
TCGA.06.0744	1.000	595.000	0.000	0.000	14.240	0.000	8.828	0.423	0.157	0.318	0.772	308.058	0.000	0.049	0.770	0.000
TCGA.06.0745	1.000	164.000	0.000	0.000	12.422	0.000	9.390	0.039	1.525	0.327	0.762	215.439	0.000	0.034	4.178	0.000
TCGA.06.0747	1.000	82.000	0.000	0.203	13.492	0.021	15.512	0.308	0.174	0.137	0.399	88.562	0.000	0.092	1.681	0.000
TCGA.06.0749	1.000	66.000	0.000	0.000	10.064	0.000	8.672	0.066	2.227	0.399	0.155	221.546	0.000	0.021	0.566	0.000
TCGA.06.0750	1.000	28.000	0.000	0.000	7.293	0.000	10.496	0.109	0.318	0.692	0.149	131.124	0.000	0.076	0.099	0.000
TCGA.06.0878	0.000	218.000	0.032	0.000	7.993	0.000	12.347	0.153	0.473	1.461	0.946	147.751	0.000	0.109	1.676	0.000
TCGA.06.0882	1.000	165.000	0.015	0.000	7.924	0.000	9.964	0.269	0.498	0.515	0.000	147.781	0.000	0.075	1.186	0.000
TCGA.06.1804	1.000	414.000	0.000	0.000	21.581	0.000	10.194	3.931	0.733	0.562	0.273	35.872	0.000	0.043	0.408	0.057
TCGA.06.2557	1.000	33.000	0.000	0.000	10.925	0.000	14.035	0.133	0.343	0.140	0.817	96.279	0.000	0.084	0.453	0.000
TCGA.06.2558	1.000	23.000	0.000	0.000	9.434	0.000	8.044	0.109	1.148	0.335	0.325	246.858	0.012	0.071	0.594	0.068
TCGA.06.2559	1.000	150.000	0.000	0.256	11.302	0.000	12.100	0.204	1.365	0.259	0.336	244.350	0.000	0.113	0.307	0.000
TCGA.06.2561	1.000	282.000	0.000	0.000	7.730	0.000	13.651	0.356	0.396	0.258	0.167	105.189	0.000	0.040	0.055	0.000
TCGA.06.2562	1.000	86.000	0.014	0.000	7.369	0.022	11.892	0.180	0.116	0.218	0.000	108.935	0.000	0.057	0.962	0.000
TCGA.06.2563	0.000	932.000	0.000	0.000	10.426	0.024	13.600	0.057	0.098	0.400	0.000	185.555	0.000	0.049	1.626	0.000
TCGA.06.2564	0.000	181.000	0.014	0.000	8.227	0.000	12.692	0.130	0.503	0.137	0.133	108.157	0.000	0.110	1.063	0.000
TCGA.06.2565	1.000	207.000	0.000	0.216	13.284	0.000	11.710	0.708	0.055	0.146	0.284	142.143	0.032	0.053	1.275	0.000
TCGA.06.2567	1.000	133.000	0.000	0.000	9.989	0.000	14.028	0.624	0.221	0.357	0.416	97.833	0.010	0.058	0.368	0.000
TCGA.06.2569	0.000	13.000	0.016	0.232	23.383	0.024	10.682	0.316	0.111	0.236	1.679	870.573	0.000	0.071	0.811	0.000
TCGA.06.2570	0.000	958.000	0.016	0.000	15.257	0.000	6.915	0.207	0.942	0.080	0.154	217.959	0.000	0.067	1.334	0.000
TCGA.06.5408	1.000	342.000	0.000	0.000	8.990	0.000	18.787	0.081	0.344	0.345	0.134	135.391	0.030	0.052	1.290	0.000
TCGA.06.5410	1.000	108.000	0.000	0.000	8.423	0.000	9.622	0.046	0.386	0.586	0.759	183.527	0.000	0.040	0.525	0.053
TCGA.06.5411	1.000	254.000	0.000	0.000	6.874	0.000	7.835	0.178	1.194	0.327	0.254	176.863	0.038	0.063	2.768	0.053
TCGA.06.5412	1.000	138.000	0.000	0.208	9.707	0.000	10.002	0.108	0.139	0.563	0.684	189.027	0.041	0.027	0.522	0.000
TCGA.06.5413	0.000	268.000	0.000	0.000	8.925	0.000	11.044	0.085	0.128	0.502	0.557	176.082	0.011	0.096	1.295	0.000
TCGA.06.5414	0.000	273.000	0.000	0.000	8.606	0.000	11.829	0.090	0.131	0.317	0.123	135.445	0.019	0.039	1.124	0.000
TCGA.06.5416	0.000	204.000	0.000	0.000	10.184	0.000	9.766	0.138	4.202	0.700	2.040	285.556	0.017	0.037	1.054	0.000
TCGA.06.5417	0.000	155.000	0.000	0.000	12.008	0.000	8.500	0.036	4.572	0.608	1.181	322.707	0.000	0.097	1.177	0.000
TCGA.06.5418	1.000	83.000	0.015	0.000	9.641	0.000	12.022	0.303	0.305	0.453	0.147	197.875	0.000	0.070	0.024	0.000
TCGA.06.5856	1.000	114.000	0.000	0.000	6.974	0.000	10.952	0.169	0.879	0.357	0.693	142.077	0.010	0.116	1.773	0.058
TCGA.06.5858	0.000	187.000	0.000	0.000	5.960	0.000	10.941	0.352	0.600	0.638	0.689	82.336	0.000	0.095	0.892	0.000
TCGA.06.5859	0.000	139.000	0.000	0.000	7.118	0.000	14.900	0.336	3.652	0.662	0.257	87.638	0.029	0.015	0.683	0.000
TCGA.08.0386	1.000	478.000	0.000	0.000	8.737	0.023	14.103	0.614	0.647	0.153	0.297	69.933	0.000	0.096	1.209	0.000
TCGA.12.0616	1.000	439.000	0.000	0.000	14.431	0.037	6.611	0.115	0.696	0.728	1.179	425.626	0.000	0.049	2.114	0.000
TCGA.12.0618	1.000	50.000	0.000	0.000	9.377	0.000	13.428	0.231	1.057	0.253	0.859	132.943	0.000	0.101	0.102	0.052
TCGA.12.0619	1.000	316.000	0.000	0.000	11.433	0.000	9.834	0.936	2.057	0.068	0.263	61.127	0.119	0.134	0.066	0.055
TCGA.12.0821	1.000	323.000	0.000	0.000	6.823	0.000	14.620	0.000	1.285	0.302	0.293	167.378	0.033	0.033	1.851	0.000
TCGA.12.1597	1.000	427.000	0.000	0.000	18.632	0.000	7.765	0.057	1.587	0.194	1.884	423.082	0.000	0.039	1.533	0.079
TCGA.12.3650	1.000	333.000	0.000	0.000	18.740	0.000	7.759	0.094	0.238	0.266	0.517	289.161	0.000	0.035	2.232	0.000
TCGA.12.3652	1.000	1050.000	0.000	0.000	20.384	0.027	7.589	0.031	0.392	0.355	1.206	583.961	0.000	0.031	1.574	0.072
TCGA.12.3653	1.000	357.000	0.000	0.000	13.622	0.000	11.809	0.014	0.122	0.000	0.458	87.740	0.000	0.017	0.913	0.000
TCGA.12.5295	1.000	399.000	0.000	0.000	12.279	0.000	7.811	0.033	0.552	0.559	1.267	303.789	0.000	0.016	3.246	0.000
TCGA.12.5299	1.000	98.000	0.000	0.000	9.146	0.000	11.315	0.032	0.160	0.204	0.264	208.893	0.000	0.034	0.635	0.000
TCGA.14.0736	1.000	460.000	0.000	0.000	8.690	0.000	4.244	0.032	1.252	0.274	0.267	498.275	0.121	0.028	1.018	0.000
TCGA.14.0781	1.000	29.000	0.000	0.000	7.034	0.000	10.174	0.272	0.135	0.249	0.242	110.016	0.037	0.029	1.505	0.000
TCGA.14.0787	1.000	68.000	0.024	0.000	9.683	0.000	15.144	0.072	0.092	0.490	0.476	151.668	0.000	0.018	1.661	0.000
TCGA.14.0789	1.000	342.000	0.000	0.000	8.835	0.000	12.187	0.133	0.097	0.654	0.363	134.774	0.027	0.030	0.392	0.000
TCGA.14.0790	1.000	419.000	0.000	0.000	8.538	0.034	10.453	0.120	0.053	0.225	0.000	234.140	0.000	0.043	1.127	0.000
TCGA.14.0817	1.000	164.000	0.000	0.000	16.807	0.000	5.582	0.028	0.585	0.236	1.224	619.468	0.012	0.076	1.804	0.064
TCGA.14.0871	1.000	880.000	0.000	1.396	23.408	0.000	25.315	0.112	0.266	1.889	2.753	312.030	0.000	0.082	0.457	0.000
TCGA.14.1402	1.000	975.000	0.000	0.000	9.854	0.000	6.625	0.070	0.289	0.298	0.193	740.930	0.029	0.087	2.145	0.000
TCGA.14.1823	1.000	543.000	0.000	0.000	8.586	0.000	5.426	0.070	0.084	0.495	0.769	413.950	0.015	0.069	0.894	0.000
TCGA.14.1829	0.000	218.000	0.000	0.000	7.424	0.000	3.672	0.000	1.979	0.998	1.234	736.620	0.000	0.008	5.913	0.074
TCGA.14.2554	1.000	532.000	0.000	0.000	10.411	0.021	9.880	0.050	0.221	0.427	0.829	362.051	0.010	0.027	1.262	0.000
TCGA.16.0846	1.000	119.000	0.000	0.000	8.470	0.000	9.800	0.150	1.644	0.169	0.000	157.753	0.025	0.025	1.090	0.000
TCGA.16.1045	1.000	883.000	0.000	0.000	8.330	0.000	9.644	0.248	0.467	0.191	0.000	135.829	0.000	0.044	0.555	0.000
TCGA.19.0957	1.000	666.000	0.000	0.000	7.614	0.000	10.116	0.094	2.456	0.000	0.466	113.787	0.012	0.109	0.541	0.000
TCGA.19.1389	1.000	141.000	0.000	0.000	5.570	0.000	10.246	0.050	0.184	0.255	0.330	120.935	0.075	0.017	0.548	0.000
TCGA.19.1390	1.000	772.000	0.000	0.197	17.121	0.000	11.196	1.025	1.406	0.267	2.204	79.664	0.000	0.041	1.550	0.000
TCGA.19.1787	1.000	385.000	0.000	0.206	7.880	0.021	9.348	0.164	0.340	0.348	1.217	79.660	0.000	0.049	0.359	0.000
TCGA.19.2619	0.000	294.000	0.000	0.000	4.448	0.019	13.692	0.180	0.269	0.318	0.124	83.282	0.000	0.100	1.210	0.000
TCGA.19.2620	1.000	148.000	0.000	0.000	9.698	0.000	7.667	0.351	0.850	0.619	0.481	160.616	0.000	0.108	0.799	0.101
TCGA.19.2624	1.000	5.000	0.000	0.000	13.110	0.000	12.112	0.180	0.460	0.331	2.184	128.980	0.000	0.196	0.683	0.000
TCGA.19.2625	1.000	124.000	0.000	0.000	11.345	0.000	13.213	0.452	1.021	0.478	0.663	152.043	0.020	0.103	1.894	0.167
TCGA.19.2629	1.000	501.000	0.000	0.000	11.116	0.000	14.962	0.078	15.918	0.264	0.256	127.237	0.039	0.025	1.042	0.000
TCGA.19.5960	1.000	165.000	0.014	0.409	12.603	0.000	9.841	0.971	1.007	0.622	0.806	148.876	0.000	0.259	0.870	0.000
TCGA.26.1442	0.000	953.000	0.000	0.197	11.445	0.000	8.906	0.087	0.439	0.533	0.647	172.882	0.000	0.099	5.676	0.000
TCGA.26.5132	0.000	286.000	0.000	0.000	13.649	0.000	11.647	0.639	0.218	0.155	0.451	125.127	0.000	0.112	1.247	0.000
TCGA.26.5133	0.000	452.000	0.000	0.000	13.484	0.000	8.846	0.096	0.868	0.405	1.259	230.273	0.000	0.016	0.941	0.000
TCGA.26.5134	0.000	167.000	0.000	0.976	10.752	0.000	11.395	0.702	0.776	0.330	0.642	140.673	0.049	0.067	1.279	0.000
TCGA.26.5136	1.000	37.000	0.083	0.000	9.458	0.000	12.918	0.617	0.491	1.881	1.218	140.821	0.000	0.152	0.472	0.000
TCGA.26.5139	0.000	48.000	0.000	0.000	8.320	0.000	11.460	0.192	0.130	0.811	0.000	100.232	0.000	0.066	1.047	0.000
TCGA.27.1830	1.000	146.000	0.000	0.000	10.408	0.000	9.247	0.516	2.980	0.162	0.471	109.146	0.036	0.096	0.730	0.000
TCGA.27.1831	1.000	505.000	0.000	0.000	6.154	0.000	6.186	0.037	0.228	0.950	1.436	849.016	0.000	0.025	3.373	0.000
TCGA.27.1832	1.000	0.000	0.000	0.000	9.842	0.000	14.566	0.826	0.580	0.274	0.267	141.744	0.010	0.036	0.465	0.000
TCGA.27.1834	1.000	1008.000	0.000	0.000	10.107	0.000	11.723	1.159	1.413	0.354	0.172	112.024	0.013	0.077	1.198	0.000
TCGA.27.1835	1.000	648.000	0.000	0.000	8.612	0.031	14.995	0.132	0.419	0.406	0.394	116.836	0.015	0.044	2.585	0.000
TCGA.27.1837	1.000	427.000	0.000	0.000	9.398	0.054	12.751	0.189	0.484	0.355	0.345	71.108	0.000	0.036	1.319	0.000
TCGA.27.2519	1.000	316.000	0.000	0.000	9.481	0.000	10.859	0.086	0.719	0.244	0.316	110.442	0.000	0.031	1.443	0.000
TCGA.27.2521	1.000	316.000	0.000	0.213	25.989	0.087	14.055	0.085	1.412	0.289	0.421	344.535	0.021	0.013	2.912	0.000
TCGA.27.2523	1.000	489.000	0.000	0.000	12.350	0.000	14.185	0.056	0.057	0.136	0.132	161.984	0.000	0.032	1.050	0.000
TCGA.27.2524	1.000	231.000	0.000	0.000	15.446	0.000	11.446	0.070	0.056	0.170	0.660	517.223	0.025	0.022	1.534	0.000
TCGA.27.2526	1.000	87.000	0.016	0.000	9.650	0.000	12.742	0.028	0.090	0.400	0.311	392.783	0.000	0.023	4.004	0.000
TCGA.27.2528	1.000	480.000	0.000	0.222	10.998	0.000	11.361	0.053	0.268	0.225	0.146	203.485	0.000	0.035	1.527	0.000
TCGA.28.1747	1.000	77.000	0.000	0.000	9.574	0.023	12.041	0.174	1.080	0.078	0.302	105.313	0.011	0.070	2.154	0.000
TCGA.28.1753	0.000	37.000	0.000	0.000	9.846	0.000	9.414	0.314	0.172	1.497	0.323	103.973	0.000	0.068	1.020	0.000
TCGA.28.2509	0.000	145.000	0.000	0.000	8.249	0.024	9.432	0.112	0.067	0.237	0.307	161.411	0.012	0.085	1.427	0.000
TCGA.28.2513	0.000	222.000	0.000	0.000	10.602	0.000	8.174	0.119	0.237	0.168	0.326	120.246	0.000	0.046	2.274	0.000
TCGA.28.2514	0.000	160.000	0.000	0.000	7.192	0.000	10.772	0.137	0.789	0.217	0.281	65.950	0.011	0.027	0.724	0.000
TCGA.28.5207	1.000	275.000	0.000	0.000	6.885	0.025	15.350	0.129	0.827	0.419	0.651	141.081	0.000	0.071	0.595	0.000
TCGA.28.5208	1.000	474.000	0.000	0.000	9.594	0.000	12.033	0.034	0.103	0.654	0.706	176.694	0.000	0.059	0.938	0.000
TCGA.28.5209	0.000	442.000	0.014	0.000	8.078	0.000	16.027	0.041	0.111	0.415	0.134	106.107	0.010	0.048	0.469	0.056
TCGA.28.5215	1.000	237.000	0.000	0.000	11.737	0.000	13.145	0.214	1.570	0.375	0.486	91.694	0.037	0.033	1.292	0.000
TCGA.28.5216	0.000	415.000	0.019	0.000	9.551	0.028	7.936	0.078	2.896	0.565	0.732	172.309	0.000	0.060	1.489	0.077
TCGA.28.5218	1.000	128.000	0.000	0.000	16.117	0.000	11.830	0.049	0.047	0.413	0.321	191.868	0.012	0.029	0.213	0.000
TCGA.28.5220	1.000	319.000	0.000	0.000	9.066	0.000	10.374	0.058	0.339	0.819	0.637	181.094	0.012	0.050	1.930	0.000
TCGA.32.1970	1.000	468.000	0.000	0.000	10.939	0.000	14.431	0.427	1.511	0.615	0.747	92.396	0.000	0.047	2.258	0.063
TCGA.32.1980	1.000	36.000	0.000	0.000	6.531	0.000	9.492	0.130	0.924	0.367	0.142	180.914	0.000	0.032	0.947	0.060
TCGA.32.1982	1.000	142.000	0.000	0.000	9.995	0.000	7.767	0.141	0.562	1.196	0.465	255.204	0.000	0.042	1.697	0.000
TCGA.32.2615	1.000	485.000	0.013	0.000	9.949	0.000	8.719	0.567	0.606	0.253	0.246	123.428	0.000	0.096	1.122	0.000
TCGA.32.2616	1.000	224.000	0.000	0.000	8.571	0.000	12.497	0.235	0.154	0.176	0.342	88.493	0.000	0.027	0.605	0.000
TCGA.32.2634	0.000	693.000	0.000	0.000	11.423	0.019	13.965	0.412	1.396	0.444	0.493	181.344	0.037	0.035	1.064	0.000
TCGA.32.2638	1.000	644.000	0.012	0.000	11.472	0.000	10.844	0.143	0.062	0.121	0.235	140.990	0.000	0.023	1.071	0.000
TCGA.32.4213	0.000	604.000	0.000	0.000	11.972	0.000	8.197	0.022	0.214	0.570	0.739	211.281	0.000	0.017	1.902	0.000
TCGA.32.5222	1.000	323.000	0.000	0.000	11.551	0.000	7.217	0.024	0.788	0.755	1.068	363.978	0.000	0.060	0.798	0.112
TCGA.41.2571	1.000	26.000	0.000	0.000	14.061	0.000	14.370	0.573	0.829	0.176	0.514	252.486	0.000	0.033	1.623	0.000
TCGA.41.2572	1.000	406.000	0.000	0.266	13.496	0.000	9.546	0.032	0.270	0.449	0.175	266.769	0.000	0.078	0.667	0.000
TCGA.41.3915	1.000	360.000	0.000	0.000	9.021	0.000	8.005	0.342	0.938	0.898	0.388	223.405	0.103	0.064	1.030	0.000
TCGA.41.4097	1.000	6.000	0.000	0.331	8.025	0.000	11.619	1.032	0.253	0.336	0.871	98.357	0.000	0.049	1.013	0.000
TCGA.41.5651	1.000	351.000	0.000	0.000	23.028	0.000	14.663	0.140	0.685	0.728	0.530	283.925	0.000	0.122	2.114	0.000
TCGA.76.4925	1.000	146.000	0.000	0.000	10.744	0.000	17.906	0.082	0.042	0.317	0.493	111.437	0.000	0.053	0.757	0.000
TCGA.76.4926	1.000	138.000	0.013	0.000	7.495	0.000	11.866	0.407	0.672	0.520	0.126	165.710	0.000	0.066	0.252	0.000
TCGA.76.4927	1.000	535.000	0.000	0.000	10.969	0.000	11.965	0.082	0.480	0.812	1.127	208.808	0.000	0.061	1.198	0.000
TCGA.76.4928	1.000	94.000	0.012	0.000	9.940	0.000	13.851	0.086	0.108	0.242	0.470	194.561	0.036	0.042	0.410	0.000
TCGA.76.4929	1.000	111.000	0.000	0.242	8.731	0.000	18.113	0.484	1.186	0.328	0.318	120.585	0.072	0.040	0.529	0.000
TCGA.76.4931	1.000	279.000	0.015	0.000	14.471	0.000	15.232	0.461	0.028	0.515	0.715	144.003	0.000	0.090	0.950	0.000
TCGA.76.4932	1.000	1458.000	0.000	0.000	9.614	0.000	12.411	0.051	0.054	0.578	0.281	159.925	0.000	0.057	0.886	0.000

OS: Overall Survival.

ENSG00000228084	ENSG00000255189	ENSG00000256235	ENSG00000265927	ENSG00000233203	ENSG00000129354	ENSG00000265692	ENSG00000233129	ENSG00000280059	ENSG00000274825	ENSG00000204852	ENSG00000229887	ENSG00000241438	ENSG00000197891	ENSG00000259489	ENSG00000252061
1.096	0.062	12.247	0.000	0.065	0.000	0.082	0.000	0.000	0.155	4.904	0.129	0.055	0.000	0.000	1.276
0.418	0.079	118.656	0.000	0.495	0.266	0.047	0.055	0.000	0.297	3.928	0.087	0.000	0.000	0.000	1.519
0.515	0.105	13.726	0.000	0.024	0.017	0.135	0.000	0.000	0.470	3.368	0.350	0.000	0.000	0.000	4.441
0.389	0.040	32.528	0.000	0.111	0.029	0.070	0.000	0.000	0.100	5.211	0.110	0.237	0.000	0.000	3.719
1.225	0.059	60.419	0.000	0.031	0.000	0.065	0.000	0.000	0.037	12.052	0.098	0.000	0.000	0.000	0.487
0.712	0.023	6.303	0.000	0.024	0.034	0.268	0.000	0.005	0.205	7.216	0.155	0.083	0.011	0.000	2.115
1.283	0.588	51.648	0.000	0.165	0.009	0.093	0.000	0.000	0.560	5.619	0.065	0.094	0.000	0.000	1.299
0.882	0.083	28.159	0.000	0.000	0.161	0.049	0.000	0.000	0.035	3.897	0.115	0.000	0.007	0.000	1.603
1.881	0.080	52.606	0.000	0.134	0.012	0.298	0.000	0.000	0.563	7.317	0.080	0.057	0.000	0.000	6.087
1.483	0.186	73.215	0.000	0.098	0.000	0.124	0.000	0.000	0.000	5.376	0.155	0.000	0.044	0.000	1.541
1.121	0.091	59.007	0.000	0.192	0.058	0.132	0.000	0.000	0.115	9.079	0.057	0.000	0.005	0.000	1.893
0.464	0.059	25.580	0.000	0.099	0.052	0.052	0.000	0.000	0.030	5.973	0.217	0.000	0.011	0.000	1.371
1.168	0.073	23.570	0.000	0.026	0.000	0.076	0.000	0.000	0.031	5.329	0.020	0.088	0.000	0.000	2.426
0.532	0.000	10.829	0.000	0.072	0.058	0.051	0.000	0.000	0.201	6.562	0.076	0.082	0.005	0.000	1.702
0.873	0.000	18.434	0.000	0.107	0.074	0.045	0.000	0.000	0.128	4.875	0.085	0.000	0.024	0.000	0.843
0.673	0.377	30.442	0.000	0.048	0.075	0.081	0.000	0.000	0.000	4.618	0.057	0.000	0.000	0.000	1.514
1.131	0.000	25.845	0.000	0.111	0.019	0.070	0.000	0.000	0.398	2.804	0.176	0.047	0.006	0.000	1.309
1.918	0.016	19.484	0.000	0.000	0.057	0.014	0.000	0.000	0.551	7.059	0.026	0.056	0.000	0.000	1.814
1.226	0.048	63.972	0.019	0.250	0.043	0.127	0.025	0.000	0.090	2.680	0.079	0.043	0.006	0.000	2.366
0.312	0.051	36.741	0.000	0.214	0.000	0.056	0.000	0.000	0.032	4.395	0.127	0.000	0.000	0.000	2.318
1.963	0.041	59.084	0.000	0.171	0.069	0.072	0.000	0.000	0.137	9.675	0.158	0.000	0.013	0.000	2.247
0.447	0.014	2.715	0.022	0.059	0.092	0.100	0.000	0.000	0.212	2.819	0.164	0.050	0.060	0.023	1.626
1.277	0.014	13.839	0.000	0.058	0.040	0.085	0.000	0.000	0.725	4.256	0.183	0.000	0.007	0.000	5.220
0.486	0.035	54.874	0.000	0.147	0.008	0.093	0.000	0.000	0.205	6.510	0.058	0.000	0.000	0.019	1.931
0.477	0.117	39.876	0.000	0.000	0.009	0.046	0.000	0.000	0.131	3.827	0.087	0.000	0.000	0.000	1.720
1.125	0.027	23.214	0.021	0.057	0.069	0.192	0.000	0.000	0.442	7.555	0.090	0.145	0.019	0.000	0.671
1.198	0.183	23.332	0.024	0.032	0.022	0.325	0.000	0.000	0.077	9.770	0.127	0.164	0.000	0.000	0.758
1.252	0.134	11.479	0.000	0.056	0.020	0.143	0.000	0.000	0.474	3.367	0.224	0.000	0.026	0.000	1.780
0.919	0.040	16.735	0.000	0.056	0.019	0.166	0.000	0.000	0.404	10.784	0.112	0.192	0.000	0.000	1.995
1.156	0.054	14.262	0.000	0.000	0.029	0.108	0.000	0.000	0.475	5.658	0.112	0.000	0.006	0.022	0.892
1.901	0.100	64.861	0.000	0.023	0.024	0.138	0.000	0.000	0.307	9.401	0.092	0.079	0.005	0.000	1.467
0.711	0.012	8.993	0.000	0.000	0.017	0.072	0.000	0.000	0.205	8.227	0.136	0.000	0.000	0.000	1.153
1.192	0.054	11.310	0.000	0.057	0.225	0.168	0.000	0.000	0.170	5.919	0.023	0.000	0.000	0.000	2.907
0.862	0.026	27.994	0.000	0.246	0.009	0.219	0.000	0.000	0.229	9.376	0.043	0.000	0.000	0.000	1.724
0.741	0.000	46.098	0.000	0.000	0.020	0.073	0.000	0.000	0.277	11.666	0.229	0.000	0.000	0.000	0.000
0.855	0.050	39.215	0.000	0.183	0.045	0.044	0.000	0.000	0.031	6.388	0.083	0.045	0.006	0.000	2.062
1.166	0.131	4.529	0.037	0.000	0.086	0.897	0.025	0.000	0.090	11.275	0.040	0.085	0.034	0.000	0.984
1.048	0.332	14.126	0.000	0.000	0.017	0.095	0.000	0.000	0.209	4.174	0.059	0.085	0.000	0.000	0.787
1.494	0.255	12.880	0.000	0.089	0.021	0.151	0.000	0.000	0.357	4.838	0.047	0.051	0.054	0.000	1.642
0.574	0.059	38.148	0.000	0.031	0.021	0.078	0.000	0.000	0.553	5.387	0.098	0.052	0.035	0.000	2.908
0.857	0.116	60.336	0.000	0.183	0.032	0.065	0.000	0.000	0.000	13.090	0.073	0.104	0.000	0.000	2.412
0.875	0.062	61.592	0.000	0.103	0.000	0.044	0.000	0.000	0.124	8.413	0.062	0.000	0.006	0.000	1.834
0.664	0.027	35.507	0.000	0.000	0.010	0.072	0.000	0.000	0.136	7.991	0.113	0.097	0.000	0.000	1.346
0.599	0.023	26.432	0.000	0.024	0.025	0.062	0.024	0.000	0.000	7.926	0.058	0.042	0.011	0.000	0.770
1.276	0.037	54.634	0.000	0.026	0.000	0.121	0.078	0.000	0.094	9.231	0.062	0.044	0.006	0.000	3.282
1.333	0.121	8.422	0.000	0.101	0.053	0.407	0.000	0.000	0.091	10.157	0.020	0.000	0.017	0.000	1.801
0.163	0.013	18.494	0.000	0.056	0.019	0.047	0.000	0.000	0.301	2.011	0.222	0.095	0.006	0.000	5.508
0.825	0.054	13.782	0.000	0.000	0.029	0.084	0.000	0.000	0.271	3.864	0.225	0.145	0.006	0.000	2.230
0.429	0.035	67.282	0.000	0.049	0.017	0.031	0.000	0.000	0.206	6.666	0.156	0.000	0.006	0.000	3.094
0.595	0.176	74.308	0.000	0.093	0.040	0.059	0.000	0.000	0.000	3.747	0.129	0.119	0.000	0.000	2.921
1.523	0.011	16.542	0.000	0.163	0.137	0.039	0.000	0.000	0.084	4.231	0.111	0.080	0.026	0.000	4.590
1.608	0.131	111.218	0.000	0.000	0.009	0.254	0.000	0.000	0.030	7.895	0.099	0.000	0.000	0.000	1.974
0.893	0.049	35.804	0.000	0.102	0.035	0.043	0.000	0.000	0.367	6.041	0.263	0.000	0.006	0.000	1.407
0.763	0.075	31.762	0.000	0.068	0.008	0.038	0.000	0.000	0.000	6.179	0.161	0.000	0.000	0.000	0.355
1.454	0.000	52.069	0.000	0.041	0.043	0.035	0.000	0.000	0.398	6.876	0.264	0.071	0.056	0.000	2.290
0.505	0.000	12.088	0.000	0.000	0.037	0.137	0.000	0.000	0.130	3.322	0.258	0.000	0.049	0.000	5.113
0.784	0.026	52.011	0.000	0.027	0.000	0.091	0.000	0.000	0.097	5.787	0.064	0.046	0.006	0.000	1.906
0.889	0.097	66.637	0.000	0.025	0.026	0.150	0.000	0.000	0.517	5.300	0.141	0.000	0.006	0.020	2.002
0.942	0.048	19.944	0.000	0.025	0.026	0.160	0.000	0.000	0.212	7.294	0.100	0.043	0.034	0.000	0.994
0.604	0.190	24.842	0.000	0.047	0.008	0.089	0.000	0.000	0.254	13.562	0.150	0.000	0.000	0.000	0.928
1.143	0.065	12.479	0.000	0.082	0.019	0.264	0.000	0.000	0.033	5.061	0.086	0.046	0.105	0.000	1.072
0.605	0.103	15.128	0.000	0.086	0.000	0.091	0.000	0.000	0.517	7.033	0.240	0.000	0.000	0.000	1.021
1.312	0.043	21.956	0.000	0.000	0.000	0.133	0.000	0.000	0.242	5.052	0.107	0.038	0.030	0.000	0.709
1.097	0.080	27.351	0.000	0.289	0.042	0.061	0.000	0.000	0.231	5.472	0.096	0.000	0.016	0.000	0.760
1.097	0.000	28.002	0.000	0.000	0.009	0.068	0.000	0.000	0.386	9.706	0.192	0.000	0.000	0.000	2.752
1.369	0.000	35.159	0.385	0.000	0.048	0.058	0.000	0.000	0.579	2.881	0.110	0.000	0.008	0.000	6.255
0.829	0.023	39.459	0.000	0.047	0.000	0.040	0.000	0.000	0.113	5.271	0.150	0.000	0.000	0.000	1.865
1.289	0.000	174.245	0.000	0.063	0.000	0.040	0.000	0.000	0.303	7.867	0.075	0.000	0.000	0.000	1.492
1.175	0.100	17.069	0.000	0.084	0.014	0.071	0.000	0.019	0.302	8.439	0.133	0.000	0.000	0.000	1.984
0.696	0.095	40.492	0.000	0.033	0.000	0.042	0.000	0.000	0.000	8.478	0.132	0.000	0.007	0.000	0.784
0.310	0.011	65.890	0.000	0.048	0.017	0.020	0.000	0.000	0.087	6.100	0.153	0.000	0.000	0.000	1.142
2.109	0.139	60.647	0.036	0.000	0.792	0.124	0.000	0.000	0.059	7.831	0.078	0.000	0.011	0.000	3.848
0.542	0.053	59.617	0.000	0.111	0.031	0.131	0.000	0.000	0.027	5.536	0.053	0.038	0.000	0.000	1.744
1.374	0.062	43.507	0.000	0.044	0.000	0.166	0.000	0.000	0.105	9.456	0.035	0.000	0.000	0.000	3.094
0.466	0.016	62.728	0.000	0.000	0.011	0.028	0.000	0.000	0.040	7.054	0.053	0.057	0.000	0.000	0.787
0.842	0.000	18.779	0.000	0.000	0.014	0.051	0.000	0.000	0.144	6.113	0.127	0.000	0.027	0.000	2.527
1.668	0.080	32.919	0.000	0.084	0.039	0.059	0.000	0.000	0.067	5.464	0.111	0.000	0.000	0.000	1.104
0.196	0.000	7.932	0.000	0.168	0.058	0.142	0.000	0.000	1.409	1.765	0.534	0.000	0.000	0.000	3.973
1.813	0.084	89.820	0.000	0.071	0.012	0.075	0.000	0.000	0.296	9.229	0.028	0.000	0.000	0.000	2.226
0.698	0.067	64.814	0.000	0.000	0.012	0.074	0.000	0.000	0.042	9.021	0.112	0.000	0.000	0.000	3.329
2.223	0.322	92.925	0.000	0.129	0.022	0.000	0.000	0.000	0.077	9.952	0.077	0.055	0.000	0.000	2.798
0.857	0.048	30.605	0.000	0.025	0.009	0.000	0.000	0.000	0.243	12.903	0.121	0.000	0.000	0.000	3.790
0.982	0.071	14.965	0.000	0.000	0.083	0.051	0.000	0.000	1.152	4.114	0.048	0.051	0.054	0.000	3.553
0.635	0.162	51.395	0.000	0.102	0.000	0.043	0.000	0.000	0.000	8.861	0.027	0.000	0.000	0.000	0.268
1.095	0.068	14.495	0.000	0.085	0.010	0.168	0.000	0.006	0.204	3.438	0.135	0.048	0.064	0.000	2.688
0.247	0.072	64.248	0.000	1.086	0.000	0.089	0.000	0.000	0.000	9.543	0.024	0.000	0.000	0.000	1.428
0.721	0.011	11.030	0.000	0.119	0.082	0.201	0.000	0.000	0.825	4.232	0.302	0.081	0.005	0.000	3.556
1.099	0.047	11.993	0.000	0.842	0.026	0.126	0.000	0.000	0.297	8.148	0.118	0.000	0.011	0.000	0.586
0.845	0.043	46.932	0.000	0.226	0.016	0.105	0.000	0.000	0.271	6.194	0.126	0.039	0.020	0.000	1.070
1.286	0.000	22.321	0.000	0.000	0.000	0.112	0.000	0.000	0.000	6.163	0.105	0.000	0.000	0.000	4.168
1.181	0.000	18.874	0.000	0.071	0.073	0.238	0.000	0.000	0.592	5.197	0.075	0.080	0.037	0.000	1.854
1.559	0.023	19.844	0.000	0.485	0.008	0.205	0.000	0.000	0.146	9.823	0.077	0.124	0.022	0.000	0.766
1.067	0.045	13.247	0.000	0.000	0.016	0.050	0.000	0.000	0.084	5.242	0.205	0.080	0.005	0.000	1.663
0.832	0.082	11.004	0.018	0.049	0.051	0.374	0.074	0.000	0.324	12.024	0.234	0.042	0.000	0.000	2.713
1.605	0.011	4.702	0.000	0.000	0.008	0.140	0.024	0.005	0.000	3.259	0.207	0.040	0.054	0.000	1.868
1.477	0.039	21.439	0.000	0.082	0.057	0.279	0.027	0.000	0.198	8.362	0.065	0.047	0.006	0.000	0.433
1.110	0.014	4.496	0.000	0.144	0.040	0.097	0.000	0.000	0.242	5.029	0.206	0.000	0.039	0.000	0.454
0.137	0.056	15.484	0.000	0.235	0.000	0.099	0.000	0.000	1.689	6.004	0.373	0.601	0.053	0.000	3.704
1.389	0.142	13.921	0.000	0.372	0.128	0.880	0.000	0.000	0.356	5.233	0.236	0.000	0.000	0.000	1.172
0.842	0.014	35.054	0.000	0.087	0.010	0.037	0.000	0.000	0.035	7.541	0.183	0.000	0.007	0.000	1.820
0.873	0.109	19.446	0.000	0.086	0.010	0.170	0.000	0.000	0.138	8.795	0.046	0.049	0.026	0.000	3.174
1.228	0.036	111.026	0.000	0.150	0.078	0.095	0.000	0.000	0.045	8.692	0.000	0.128	0.000	0.000	7.402
0.541	0.197	47.640	0.000	0.585	0.008	0.258	0.000	0.000	0.439	12.573	0.039	0.000	0.000	0.000	0.577
0.661	0.195	6.013	0.000	0.000	0.011	0.133	0.000	0.000	0.151	9.608	0.025	0.161	0.000	0.000	0.248
1.305	0.000	17.468	0.000	0.000	0.000	0.183	0.000	0.000	0.086	5.338	0.115	0.062	0.016	0.000	2.275
0.443	0.000	12.455	0.000	0.190	0.044	0.053	0.000	0.000	0.076	4.669	0.100	0.000	0.000	0.000	0.996
1.250	0.055	20.881	0.022	0.087	0.000	0.086	0.000	0.000	0.104	8.334	0.069	0.049	0.098	0.000	4.334
1.859	0.000	12.858	0.000	0.026	0.018	0.043	0.000	0.000	0.000	6.464	0.061	0.000	0.006	0.000	2.632
1.323	0.023	55.663	0.000	0.048	0.008	0.020	0.000	0.000	0.289	6.064	0.134	0.041	0.011	0.000	0.570
0.952	0.029	96.985	0.000	0.121	0.021	0.051	0.000	0.000	0.398	6.247	0.168	0.000	0.000	0.000	2.619
0.964	0.027	28.828	0.000	0.114	0.010	0.072	0.000	0.000	0.205	7.011	0.000	0.000	0.000	0.000	1.572
0.530	0.013	37.800	0.020	0.267	0.055	0.056	0.000	0.000	0.224	6.884	0.149	0.000	0.006	0.000	2.107
0.580	0.026	15.023	0.000	0.083	0.038	0.058	0.000	0.000	0.199	5.349	0.132	0.047	0.006	0.000	3.919
0.829	0.113	20.212	0.000	0.059	0.000	0.150	0.000	0.000	1.063	4.154	0.047	0.101	0.040	0.000	0.933
0.722	0.040	79.737	0.000	0.477	0.000	0.095	0.000	0.000	0.135	6.539	0.022	0.048	0.000	0.000	1.108
0.836	0.142	68.970	0.000	0.030	0.000	0.076	0.000	0.000	0.036	7.987	0.000	0.051	0.000	0.000	2.823
1.322	0.024	59.612	0.000	0.129	0.124	0.043	0.000	0.000	0.339	6.141	0.245	0.044	0.000	0.000	0.203
1.148	0.113	26.834	0.000	0.030	0.010	0.050	0.000	0.000	0.107	7.825	0.095	0.051	0.000	0.000	2.584
0.543	0.049	86.955	0.000	0.000	0.009	0.044	0.000	0.000	0.093	7.144	0.144	0.000	0.000	0.000	1.835
0.431	0.023	51.133	0.000	0.172	0.000	0.021	0.000	0.000	0.206	4.823	0.195	0.042	0.000	0.000	0.776
0.935	0.095	12.986	0.000	0.067	0.015	0.047	0.000	0.000	0.613	4.939	0.177	0.076	0.020	0.000	1.052
0.821	0.032	37.723	0.025	0.201	0.023	0.028	0.000	0.000	0.723	5.582	0.186	0.057	0.015	0.000	1.849
0.514	0.070	22.646	0.000	0.059	0.030	0.025	0.000	0.000	0.633	4.328	0.117	0.000	0.020	0.023	2.082
1.055	0.139	23.672	0.000	0.000	0.000	0.074	0.000	0.000	0.245	11.929	0.231	0.000	0.000	0.000	2.527
0.639	0.052	11.124	0.020	0.055	0.076	0.139	0.000	0.000	0.131	6.456	0.196	0.140	0.037	0.000	1.510
1.188	0.074	30.686	0.019	0.078	0.144	0.077	0.000	0.000	0.094	4.763	0.083	0.000	0.000	0.000	2.879
1.092	0.081	85.956	0.000	0.000	0.059	0.216	0.000	0.000	0.000	6.392	0.068	0.000	0.000	0.000	4.693
0.761	0.139	20.255	0.000	0.067	0.031	0.180	0.000	0.000	0.162	12.898	0.125	0.000	0.000	0.000	1.418
0.730	0.069	9.621	0.016	0.292	0.000	0.141	0.000	0.000	0.225	7.501	0.083	0.036	0.024	0.000	0.822
2.397	0.043	14.556	0.000	0.180	0.094	0.286	0.000	0.000	0.243	6.892	0.215	0.039	0.010	0.000	3.380
0.827	0.112	38.231	0.000	0.021	0.015	0.082	0.021	0.000	0.026	6.385	0.102	0.037	0.005	0.000	1.693
0.789	0.161	31.509	0.000	0.135	0.023	0.171	0.000	0.000	0.081	4.931	0.215	0.000	0.015	0.000	2.665
0.913	0.046	69.590	0.018	0.000	0.008	0.052	0.000	0.000	0.117	7.614	0.213	0.042	0.000	0.000	2.118
1.795	0.000	14.170	0.000	0.031	0.011	0.318	0.000	0.000	0.451	4.217	0.174	0.000	0.199	0.000	3.711
0.672	0.061	24.645	0.000	0.064	0.011	0.027	0.000	0.000	0.268	6.857	0.152	0.000	0.014	0.000	1.260
1.367	0.152	23.662	0.000	0.071	0.000	0.210	0.035	0.000	0.170	7.898	0.085	0.000	0.024	0.000	1.119
0.558	0.133	27.190	0.000	0.159	0.041	0.185	0.000	0.000	0.239	9.852	0.127	0.000	0.018	0.031	1.886
0.302	0.015	11.995	0.000	0.000	0.067	0.082	0.000	0.000	0.543	2.554	0.386	0.000	0.022	0.000	2.042
1.264	0.000	46.995	0.050	0.000	4.760	0.229	0.000	0.000	0.433	5.901	0.179	0.000	0.015	0.000	0.356
1.026	0.022	19.825	0.000	0.046	0.064	0.127	0.000	0.000	0.166	11.532	0.073	0.000	0.005	0.000	3.647
1.012	0.000	27.003	0.000	0.000	0.029	0.035	0.000	0.000	0.148	6.530	0.131	0.000	0.000	0.000	2.278
0.301	0.072	78.334	0.000	0.086	0.022	0.027	0.000	0.000	0.052	9.646	0.137	0.037	0.005	0.017	0.509
1.633	0.014	21.342	0.000	0.029	0.010	0.382	0.000	0.000	0.035	10.674	0.116	0.099	0.020	0.000	2.067
0.428	0.012	39.932	0.000	0.052	0.036	0.188	0.000	0.000	0.220	6.841	0.146	0.045	0.030	0.000	0.826
0.450	0.037	82.132	0.000	0.051	0.000	0.076	0.000	0.000	0.062	6.049	0.204	0.000	0.000	0.000	1.418

ENSG00000101407	ENSG00000112293	ENSG00000119283	ENSG00000154222	ENSG00000070761	ENSG00000234282	ENSG00000074317	ENSG00000261838	ENSG00000258678	ENSG00000263793	ENSG00000274055	ENSG00000203865	ENSG00000280345	ENSG00000260676	ENSG00000036054	ENSG00000254857
5.586	0.564	2.187	4.167	14.902	0.000	17.162	0.000	0.000	0.000	0.000	1.017	0.000	0.000	9.482	0.000
8.315	0.154	0.025	6.031	12.439	0.000	10.248	0.000	0.000	0.000	0.000	0.599	0.088	0.000	12.585	0.000
8.924	0.217	1.205	3.176	32.271	0.000	6.695	0.076	0.044	0.000	0.000	0.655	0.000	0.000	8.560	0.000
8.191	0.380	0.171	3.680	21.066	0.043	9.604	0.000	0.000	0.000	0.000	0.751	0.000	0.000	7.838	0.000
4.416	0.182	0.013	6.238	9.580	0.000	0.471	0.000	0.000	0.000	0.000	1.536	0.000	0.000	9.156	0.000
12.368	0.535	0.598	5.594	32.458	0.038	3.989	0.038	0.131	0.000	0.000	1.358	0.026	0.000	9.831	0.019
8.881	0.081	0.014	5.234	16.262	0.000	0.555	0.127	0.049	0.000	0.000	0.564	0.000	0.000	13.146	0.000
4.637	0.307	0.164	4.479	13.039	0.000	99.136	0.537	0.104	0.000	0.000	0.759	0.000	0.000	8.215	0.000
5.766	0.209	0.391	3.891	26.037	0.000	11.171	0.000	0.000	0.377	0.000	1.149	0.000	0.000	8.926	0.000
4.287	0.137	0.032	5.284	13.025	0.000	1.974	0.000	0.000	0.000	0.000	1.003	0.000	0.000	8.559	0.000
4.476	0.254	0.140	5.573	15.020	0.000	5.079	0.074	0.000	0.000	0.000	0.590	0.000	0.000	9.151	0.000
11.604	0.599	0.094	4.845	14.043	0.000	22.225	0.077	0.000	0.000	0.000	0.835	0.000	0.006	8.675	0.000
9.785	0.562	0.235	3.851	18.990	0.000	1.300	0.040	0.184	0.000	0.000	0.925	0.000	0.013	7.680	0.000
8.852	1.156	0.083	5.846	17.474	0.112	10.578	0.074	0.000	0.000	0.000	1.045	0.000	0.006	7.892	0.000
7.846	0.332	0.202	4.918	11.390	0.000	37.236	0.824	0.000	0.000	0.000	1.263	0.000	0.000	6.657	0.000
6.402	0.258	0.020	6.471	9.013	0.074	0.979	0.074	0.000	0.000	0.000	1.314	0.000	0.000	8.294	0.000
9.325	0.607	1.235	2.796	29.923	0.043	12.084	0.043	0.000	0.000	0.000	0.741	0.000	0.000	8.991	0.000
4.696	0.255	0.253	3.068	63.246	0.051	4.585	0.101	0.000	0.000	0.000	1.646	0.000	0.000	7.280	0.000
6.212	0.144	0.016	5.848	13.858	0.078	3.842	0.039	0.000	0.000	0.000	0.809	0.000	0.000	7.712	0.000
6.851	0.499	0.013	6.535	17.250	0.041	2.405	0.124	0.048	0.000	0.000	1.056	0.000	0.000	7.128	0.000
4.584	0.372	0.056	3.440	23.914	0.000	30.747	0.044	0.051	0.000	0.000	1.108	0.000	0.000	8.333	0.000
9.783	1.365	1.077	5.360	13.556	0.183	77.304	0.136	0.000	0.000	0.000	1.146	0.000	0.000	7.071	0.000
6.895	0.304	1.083	4.006	57.160	0.000	29.453	0.000	0.000	0.000	0.000	1.370	0.000	0.000	9.439	0.000
6.983	0.156	0.004	5.507	19.366	0.000	0.504	0.038	0.000	0.000	0.000	0.465	0.026	0.000	8.588	0.000
6.927	0.335	0.101	5.158	14.196	0.000	10.352	0.000	0.000	0.000	0.000	0.628	0.000	0.000	7.597	0.000
7.072	0.714	3.087	6.448	21.799	0.000	9.078	0.131	0.000	0.000	0.000	1.376	0.000	0.007	8.143	0.000
6.657	0.484	0.097	6.693	23.312	0.050	2.860	0.148	0.000	0.000	0.000	2.524	0.000	0.000	9.964	0.000
6.275	0.325	2.222	4.989	40.103	0.000	25.634	0.087	0.000	0.000	0.000	0.966	0.000	0.000	6.892	0.000
11.719	0.450	0.097	4.874	17.609	0.087	2.819	0.043	0.000	0.000	0.000	1.295	0.000	0.000	6.299	0.000
6.339	0.659	0.044	4.824	26.315	0.000	9.799	0.218	0.000	0.000	0.000	0.827	0.030	0.000	7.415	0.000
10.362	0.315	1.646	3.332	31.398	0.108	12.592	0.036	0.084	0.000	0.000	1.201	0.000	0.000	4.636	0.000
12.236	0.527	0.338	5.253	27.652	0.151	5.473	0.038	0.000	0.000	0.000	0.705	0.000	0.000	7.077	0.000
4.809	0.300	0.295	5.498	16.931	0.132	89.353	0.044	0.000	0.000	0.000	1.385	0.000	0.000	5.586	0.000
6.361	0.255	0.074	6.437	23.896	0.000	4.700	0.000	0.000	0.000	0.000	1.029	0.000	0.000	5.780	0.000
6.683	0.960	0.099	4.730	18.365	0.000	2.443	0.089	0.000	0.000	0.000	0.736	0.000	0.000	9.491	0.000
7.041	0.455	0.064	5.619	14.878	0.243	22.729	0.040	0.047	0.000	0.000	1.285	0.000	0.000	8.474	0.000
12.183	1.072	0.102	9.747	13.816	0.349	12.073	0.154	0.000	0.000	0.000	2.976	0.080	0.000	9.535	0.000
11.230	1.206	0.295	7.790	11.429	0.039	3.575	0.000	0.000	0.000	0.000	1.498	0.000	0.000	7.736	0.000
8.616	0.486	1.562	4.445	33.074	0.046	25.602	0.046	0.000	0.000	0.000	1.305	0.032	0.000	11.501	0.000
8.141	0.191	1.722	5.006	18.214	0.000	8.426	0.000	0.000	0.000	0.000	0.583	0.033	0.000	12.965	0.072
6.173	0.509	0.198	6.535	14.035	0.095	20.885	0.141	0.000	0.000	0.000	1.313	0.000	0.000	8.125	0.000
9.013	0.744	0.042	5.022	15.517	0.000	16.686	0.040	0.046	0.000	0.000	0.732	0.000	0.006	10.654	0.000
10.351	0.765	0.056	4.107	18.220	0.088	15.878	0.263	0.153	0.000	0.000	0.611	0.000	0.000	8.188	0.000
8.159	0.467	0.046	5.459	15.984	0.076	3.779	0.113	0.000	0.000	0.000	1.132	0.000	0.000	10.742	0.000
11.533	1.416	0.030	4.548	17.519	0.081	2.430	0.120	0.047	0.000	0.000	0.712	0.000	0.000	9.749	0.000
9.892	0.347	0.042	6.284	15.166	0.197	5.708	0.352	0.000	0.000	0.000	3.513	0.000	0.000	9.351	0.000
9.271	0.669	0.073	5.392	20.868	0.043	0.437	0.086	0.000	0.000	0.000	1.087	0.000	0.007	10.256	0.000
9.310	0.435	0.682	5.278	30.006	0.000	8.550	0.087	0.152	0.000	0.000	0.941	0.000	0.000	9.132	0.000
8.973	0.698	0.525	5.112	25.190	0.076	5.329	0.151	0.000	0.000	0.000	0.893	0.000	0.000	7.116	0.000
5.381	0.054	0.013	6.391	9.209	0.036	11.256	0.036	0.000	0.000	0.000	1.131	0.000	0.000	7.693	0.000
7.432	1.021	1.180	4.750	32.658	0.036	113.731	0.036	0.042	0.000	0.000	0.826	0.000	0.000	8.584	0.000
5.267	0.467	0.023	5.460	14.949	0.000	9.613	0.039	0.000	0.000	0.000	0.896	0.000	0.000	8.122	0.000
8.585	0.662	0.128	3.941	30.591	0.000	1.895	0.000	0.000	0.000	0.000	1.190	0.000	0.000	11.002	0.000
8.657	0.434	0.039	4.603	12.553	0.035	0.430	0.174	0.000	0.000	0.000	0.624	0.000	0.000	10.607	0.000
6.547	0.277	1.477	4.031	36.427	0.129	12.894	0.000	0.000	0.000	0.000	1.214	0.000	0.000	9.248	0.000
8.269	0.571	0.071	3.772	16.151	0.000	0.742	0.167	0.194	0.000	0.000	1.547	0.000	0.000	17.420	0.000
5.771	0.459	0.020	5.447	10.036	0.042	8.266	0.124	0.000	0.000	0.000	1.145	0.000	0.000	11.848	0.000
11.916	0.359	0.454	4.450	30.770	0.000	5.343	0.000	0.000	0.286	0.000	1.280	0.000	0.000	12.450	0.000
7.012	0.165	0.072	6.535	15.506	0.000	1.144	0.039	0.045	0.000	0.000	1.224	0.000	0.000	8.686	0.000
7.598	1.760	0.343	4.910	14.276	0.146	1.794	0.036	0.042	0.000	0.000	1.428	0.000	0.000	4.642	0.000
9.943	0.364	0.881	8.165	20.065	0.211	12.153	0.042	0.000	0.000	0.000	2.048	0.000	0.000	8.255	0.000
8.024	0.235	0.499	3.073	39.172	0.000	3.702	0.000	0.000	0.000	0.000	1.007	0.000	0.000	9.809	0.000
9.770	0.720	3.368	4.177	15.937	0.070	14.749	0.035	0.040	0.253	0.119	1.035	0.024	0.000	8.730	0.000
8.362	0.375	0.217	8.950	14.079	0.112	0.854	0.074	0.000	0.000	0.000	1.199	0.000	0.000	7.975	0.000
9.082	0.597	0.315	5.093	25.195	0.208	12.366	0.083	0.000	0.000	0.000	1.111	0.000	0.000	11.634	0.000
10.431	0.241	1.245	4.270	27.558	0.000	20.384	0.053	0.000	0.000	0.000	0.794	0.000	0.000	6.013	0.000
6.714	0.456	0.241	3.370	31.904	0.000	11.743	0.000	0.000	0.000	0.000	0.309	0.000	0.000	7.450	0.000
6.560	0.628	0.026	2.272	23.698	0.049	8.491	0.000	0.000	0.000	0.000	0.510	0.000	0.000	10.643	0.000
9.152	1.011	0.034	4.489	19.299	0.130	0.336	0.000	0.000	0.000	0.000	1.018	0.000	0.000	6.821	0.000
6.347	0.350	0.030	3.201	15.874	0.000	1.339	0.000	0.059	0.000	0.000	0.917	0.000	0.000	10.849	0.000
5.079	0.345	0.030	4.097	23.139	0.037	18.668	0.037	0.000	0.000	0.000	0.984	0.000	0.000	9.265	0.000
4.025	0.379	0.080	3.212	15.308	0.000	58.241	0.000	0.088	0.000	0.000	2.916	0.000	0.012	7.093	0.000
4.707	0.248	0.020	6.671	10.465	0.000	2.311	0.034	0.040	0.000	0.000	0.936	0.000	0.005	7.625	0.000
10.377	0.603	0.172	6.481	19.457	0.000	12.698	0.000	0.078	0.000	0.000	1.275	0.000	0.000	7.826	0.000
5.349	0.496	0.033	4.995	10.665	0.258	1.775	0.000	0.060	0.000	0.000	0.631	0.000	0.000	7.657	0.000
7.300	0.343	0.102	4.162	19.342	0.249	0.733	0.000	0.000	0.000	0.000	1.010	0.000	0.000	8.729	0.000
5.485	0.292	0.106	3.992	47.583	0.043	9.162	0.000	0.000	0.000	0.000	1.560	0.000	0.000	9.151	0.000
14.857	0.418	0.069	5.502	11.737	0.000	1.281	0.000	0.000	0.000	0.000	1.463	0.000	0.000	5.991	0.000
7.188	0.510	0.041	2.852	29.599	0.109	17.326	0.000	0.000	0.000	0.000	1.460	0.000	0.000	13.106	0.000
5.161	0.717	0.017	5.626	18.615	0.000	2.951	0.543	0.000	0.000	0.000	3.065	0.000	0.000	9.862	0.000
3.719	0.171	0.180	2.222	32.510	0.000	0.775	0.050	0.000	0.000	0.000	1.285	0.000	0.000	20.073	0.000
5.905	0.515	0.017	4.151	22.548	0.000	8.834	0.078	0.000	0.285	0.000	1.566	0.000	0.000	12.642	0.000
4.606	0.257	1.557	5.686	35.356	0.047	44.162	0.046	0.000	0.000	0.000	1.019	0.000	0.000	6.388	0.000
6.874	1.211	0.159	6.537	14.409	0.053	1.465	0.000	0.000	0.000	0.000	0.815	0.000	0.000	8.725	0.000
5.796	0.185	1.134	8.014	17.160	0.044	17.068	0.000	0.000	0.000	0.151	1.220	0.000	0.000	7.848	0.000
7.576	0.230	0.007	6.932	8.475	0.000	0.518	0.326	0.000	0.000	0.000	0.516	0.000	0.000	8.401	0.000
9.476	0.199	1.995	3.623	27.547	0.074	3.240	0.110	0.000	0.000	0.000	1.159	0.000	0.000	9.245	0.000
7.994	0.196	0.073	9.302	21.877	0.000	1.425	0.115	0.000	0.000	0.000	1.055	0.000	0.000	7.298	0.000
6.105	0.612	0.109	4.320	14.317	0.035	3.906	0.105	0.000	0.000	0.000	0.774	0.000	0.000	6.201	0.000
4.573	0.500	0.090	4.850	15.476	0.137	9.338	0.204	0.079	0.000	0.000	0.754	0.000	0.000	6.851	0.000
10.421	0.446	1.167	7.466	24.810	0.073	22.407	0.073	0.000	0.794	0.125	0.571	0.000	0.006	7.016	0.018
7.019	0.166	0.022	11.682	20.055	0.000	2.157	0.112	0.000	0.000	0.000	1.216	0.000	0.000	5.580	0.000
6.605	0.211	0.834	6.422	27.486	0.000	2.439	0.036	0.126	0.000	0.000	1.305	0.000	0.000	7.344	0.000
14.808	1.005	0.177	5.363	19.085	0.229	18.425	0.076	0.000	0.000	0.000	1.132	0.000	0.000	10.491	0.000
5.276	0.630	0.280	4.904	13.018	0.221	23.364	0.037	0.085	0.000	0.000	1.290	0.051	0.000	6.267	0.000
7.510	0.457	0.032	6.483	19.094	0.000	3.071	0.127	0.000	0.000	0.000	1.300	0.000	0.000	10.480	0.000
8.238	0.421	0.116	5.740	21.898	0.045	0.220	0.000	0.000	0.000	0.000	0.484	0.000	0.000	11.709	0.000
8.784	1.004	2.466	5.309	38.274	0.000	12.717	0.000	0.000	0.000	0.624	1.316	0.000	0.000	7.700	0.000
8.505	0.289	0.104	6.835	21.186	0.115	5.583	0.115	0.000	0.000	0.000	1.249	0.080	0.000	10.055	0.000
5.933	0.368	0.050	5.409	12.982	0.000	14.027	0.044	0.000	0.000	0.000	0.880	0.000	0.000	8.848	0.000
7.920	0.680	0.594	5.458	26.280	0.134	1.634	0.044	0.000	0.000	0.000	1.351	0.000	0.007	11.350	0.000
3.001	0.123	0.022	3.046	25.322	0.000	1.031	0.058	0.000	0.000	0.000	1.507	0.000	0.009	14.944	0.000
8.074	0.224	0.064	6.333	13.882	0.038	1.246	0.038	0.044	0.000	0.000	1.207	0.000	0.000	8.062	0.000
5.928	0.430	0.513	7.875	24.204	0.000	4.149	0.048	0.000	0.354	0.000	1.585	0.000	0.000	12.017	0.000
10.259	0.628	0.033	4.846	16.374	0.000	0.825	0.111	0.065	0.000	0.000	0.718	0.000	0.000	6.986	0.000
5.794	0.422	0.497	4.359	20.390	0.098	13.685	0.000	0.000	0.000	0.000	0.649	0.000	0.000	7.089	0.000
9.355	0.180	0.648	5.048	22.962	0.000	2.349	0.268	0.000	0.000	0.000	1.008	0.000	0.000	9.285	0.000
8.229	0.359	0.821	5.696	21.604	0.080	1.576	0.000	0.000	0.000	0.000	1.542	0.000	0.000	8.693	0.000
11.815	0.959	0.012	3.431	27.227	0.037	12.341	0.037	0.000	0.271	0.000	0.870	0.000	0.000	10.048	0.000
5.744	0.221	0.017	4.012	14.275	0.000	13.240	0.000	0.054	0.000	0.000	0.498	0.000	0.000	7.682	0.000
4.254	0.106	0.119	3.876	19.714	0.044	0.402	0.044	0.000	0.000	0.000	1.675	0.000	0.000	11.007	0.000
8.063	0.540	0.061	2.945	17.418	0.041	9.200	0.124	0.048	0.000	0.000	0.715	0.000	0.000	9.444	0.000
6.384	0.468	0.290	5.133	11.230	0.086	11.453	0.043	0.050	0.000	0.000	0.679	0.000	0.000	6.865	0.000
7.382	0.570	0.315	4.695	19.312	0.092	3.518	0.000	0.106	0.000	0.000	0.442	0.000	0.000	6.670	0.000
10.372	0.140	0.007	6.892	22.183	0.044	1.628	0.043	0.000	0.000	0.000	1.189	0.030	0.000	12.186	0.000
7.937	0.482	0.029	4.792	13.229	0.093	1.206	0.000	0.000	0.000	0.000	0.947	0.000	0.000	9.073	0.000
6.904	1.128	1.298	2.353	16.856	0.000	32.879	0.198	0.139	0.000	0.000	0.825	0.000	0.000	8.398	0.000
8.324	1.107	0.638	5.026	27.260	0.046	37.989	0.000	0.000	0.000	0.000	1.381	0.000	0.000	9.115	0.000
8.900	0.531	0.017	3.424	23.886	0.000	1.991	0.000	0.000	0.000	0.000	0.668	0.000	0.000	9.707	0.000
8.339	0.623	0.022	3.629	18.554	0.038	2.241	0.000	0.000	0.000	0.000	0.513	0.000	0.000	7.945	0.000
8.133	0.578	1.516	5.750	30.983	0.000	3.138	0.034	0.000	0.000	0.000	0.997	0.000	0.000	10.624	0.000
6.354	0.307	2.052	4.689	28.810	0.052	11.340	0.000	0.000	0.000	0.000	0.740	0.000	0.000	13.284	0.000
7.907	0.342	0.034	3.483	12.380	0.091	0.179	0.000	0.000	0.000	0.000	0.466	0.000	0.000	7.639	0.000
6.787	0.227	0.330	5.682	32.580	0.045	0.678	0.000	0.052	0.000	0.000	1.741	0.000	0.000	4.912	0.000
8.799	0.817	0.368	5.711	15.608	0.085	14.120	0.042	0.000	0.000	0.000	1.583	0.000	0.000	7.145	0.000
5.612	0.523	0.244	5.867	18.807	0.081	143.169	0.080	0.000	0.000	0.000	0.625	0.000	0.000	5.875	0.000
6.578	0.621	0.049	4.365	22.291	0.000	17.702	0.000	0.000	0.000	0.000	0.741	0.000	0.000	6.343	0.000
7.167	0.828	0.288	5.689	20.329	0.035	9.334	0.104	0.000	0.000	0.000	1.210	0.000	0.000	7.060	0.000
7.858	0.220	0.121	8.304	16.887	0.032	0.803	0.032	0.000	0.000	0.000	0.856	0.000	0.000	7.955	0.000
19.568	0.561	0.503	5.961	28.737	0.070	14.770	0.035	0.203	0.000	0.000	1.158	0.000	0.000	9.179	0.000
8.948	0.544	0.033	4.241	17.235	0.067	4.559	0.066	0.000	0.000	0.114	0.955	0.000	0.000	8.283	0.000
5.622	0.200	0.100	3.836	15.785	0.000	0.670	0.209	0.121	0.000	0.000	0.694	0.000	0.000	9.265	0.000
6.507	0.380	0.042	3.589	25.029	0.000	4.200	0.000	0.044	0.000	0.000	1.186	0.000	0.000	11.571	0.000
11.653	1.171	5.005	7.511	19.056	0.049	23.423	0.145	0.000	0.000	0.000	1.123	0.000	0.000	5.131	0.000
8.836	0.512	0.008	4.247	15.203	0.000	4.741	0.049	0.000	0.000	0.000	0.577	0.000	0.000	7.460	0.000
7.199	0.265	0.134	5.479	19.656	0.000	2.408	0.109	0.000	0.000	0.000	1.325	0.000	0.000	11.092	0.000
6.262	0.638	0.255	6.020	13.721	0.062	19.042	0.123	0.000	0.000	0.000	0.893	0.000	0.000	9.462	0.000
14.519	0.538	6.758	3.881	39.879	0.000	22.299	0.000	0.058	0.000	0.000	0.957	0.000	0.008	7.640	0.000
12.947	0.407	0.031	6.043	15.411	0.070	8.770	0.035	0.000	0.000	0.000	1.523	0.000	0.000	5.090	0.000
6.334	0.568	0.123	5.189	16.778	0.072	26.953	0.036	0.000	0.000	0.000	1.662	0.000	0.000	9.086	0.000
6.209	0.385	0.203	5.492	20.069	0.064	4.736	0.000	0.000	0.000	0.000	0.860	0.000	0.000	7.207	0.000
6.023	0.445	0.106	5.463	14.788	0.100	2.304	0.000	0.000	0.000	0.000	0.830	0.000	0.000	9.100	0.000
9.426	1.807	0.463	7.303	30.336	0.000	1.544	0.045	0.000	0.000	0.000	1.332	0.000	0.000	5.660	0.000
10.330	0.708	0.146	5.650	21.109	0.122	23.675	0.040	0.000	0.000	0.000	0.782	0.000	0.000	7.651	0.000
6.165	0.280	0.025	6.341	18.024	0.000	6.006	0.000	0.000	0.000	0.000	1.175	0.000	0.000	8.120	0.000

ENSG00000221263	ENSG00000117697	ENSG00000137225	ENSG00000212138	ENSG00000278828	ENSG00000073598	ENSG00000248722	ENSG00000233369	ENSG00000103353	ENSG00000189275	ENSG00000168952	ENSG00000233122	ENSG00000199473	ENSG00000212446	ENSG00000235039	ENSG00000160888
0.000	14.731	0.112	0.000	0.140	0.125	0.000	6.145	10.915	0.073	1.135	0.142	0.000	0.000	0.000	14.510
0.000	9.279	0.071	0.000	0.953	0.121	0.000	2.612	6.055	0.037	0.848	0.095	0.000	0.000	0.000	34.297
0.223	16.152	0.032	0.000	2.619	0.135	0.000	4.271	11.281	0.006	0.260	0.077	0.000	0.000	0.000	53.440
0.000	15.134	0.036	0.000	0.343	0.382	0.000	6.632	8.860	0.019	0.358	0.061	0.000	0.000	0.000	106.235
0.000	17.722	0.020	0.000	1.374	0.085	0.000	4.761	4.551	0.007	0.731	0.068	0.000	0.000	0.000	10.272
0.000	12.419	0.068	0.000	0.980	0.282	0.000	10.905	12.277	0.000	0.728	0.459	0.000	0.000	0.000	17.158
0.750	9.990	0.272	0.000	1.647	0.121	0.000	3.230	11.116	0.000	2.978	0.224	0.000	0.000	0.000	26.040
0.000	15.270	0.044	0.000	0.377	0.016	0.000	5.127	6.181	0.243	5.196	0.018	0.000	0.000	0.043	12.467
0.000	21.855	0.029	0.000	0.540	0.037	0.000	5.961	8.372	0.053	1.181	0.032	0.000	0.000	0.000	27.985
0.000	17.425	0.253	0.000	1.027	0.000	0.000	2.133	4.413	0.000	0.945	0.153	0.000	0.698	0.000	20.334
1.093	7.719	0.135	0.000	3.117	0.013	0.000	4.441	7.975	0.000	0.683	0.090	0.000	0.000	0.000	8.176
0.000	12.073	0.027	0.000	0.768	0.137	0.000	6.461	10.696	0.483	0.787	0.117	0.000	0.000	0.000	27.529
0.000	14.864	0.050	0.000	0.523	0.127	0.000	7.754	8.803	0.000	0.780	0.080	0.000	0.000	0.000	14.637
0.218	10.647	0.041	0.159	0.519	0.106	0.000	10.371	9.415	0.049	0.696	0.233	0.000	0.000	0.000	26.267
0.000	16.021	0.115	0.000	0.859	0.177	0.000	6.023	6.049	0.169	1.945	0.034	0.000	0.000	0.000	14.791
0.219	8.602	0.067	0.000	1.336	0.040	0.000	3.103	6.136	0.000	1.138	0.106	0.000	0.000	0.000	53.454
0.000	14.712	0.018	0.000	0.531	0.305	0.000	10.406	10.621	0.006	2.246	0.070	0.000	0.198	0.000	24.795
0.000	40.378	0.057	0.000	2.682	0.054	0.000	4.206	6.030	0.052	0.417	0.031	0.000	0.000	0.000	8.140
0.000	13.435	0.081	0.000	2.613	0.041	0.000	3.407	5.687	0.006	0.647	0.055	0.000	0.000	0.000	20.201
0.000	9.455	0.029	0.000	2.165	0.206	0.000	5.149	9.872	0.000	0.190	0.117	0.000	0.000	0.000	14.807
0.519	34.175	0.098	0.000	1.409	0.126	0.000	2.246	4.531	0.058	1.767	0.000	0.000	0.000	0.000	10.067
0.000	7.671	0.044	0.588	0.364	0.211	0.000	11.536	12.607	0.067	4.203	0.194	0.000	0.211	0.043	9.996
0.262	21.981	0.050	0.000	3.096	0.095	0.000	7.356	9.445	0.046	3.094	0.081	0.000	0.000	0.000	18.036
0.000	8.739	0.164	0.163	0.969	0.135	0.000	3.056	12.040	0.000	0.156	0.015	0.000	0.000	0.000	11.290
0.248	13.435	0.065	0.000	0.590	0.045	0.000	6.355	6.969	0.000	0.700	0.068	0.000	0.000	0.000	37.859
0.516	11.881	0.122	0.000	0.754	0.141	0.000	7.449	12.446	0.006	0.116	0.258	0.000	0.000	0.000	52.175
0.000	13.546	0.104	0.000	1.903	0.053	0.000	4.572	4.896	0.000	0.258	0.131	0.000	0.000	0.000	16.235
0.000	12.844	6.594	0.000	0.873	0.156	0.000	8.051	8.967	0.077	1.477	0.053	0.000	0.000	0.000	17.821
0.000	11.957	0.079	0.000	0.574	0.046	0.000	4.415	6.152	0.006	0.104	0.132	0.000	0.201	0.000	26.500
0.257	15.293	0.128	0.188	0.245	0.078	0.000	7.005	6.024	0.070	0.359	0.133	0.000	0.000	0.000	28.264
0.000	17.754	0.050	0.000	1.265	0.051	0.000	7.743	9.209	0.005	0.355	0.066	0.000	0.000	0.000	32.705
0.000	9.214	0.021	0.162	0.271	0.081	0.000	6.209	9.324	0.011	0.155	0.161	0.000	0.000	0.000	39.758
0.000	16.457	0.104	0.000	0.140	0.047	0.000	3.353	4.610	0.237	1.904	0.027	0.000	0.000	0.000	20.849
0.000	11.068	0.206	0.000	1.876	0.075	0.000	3.296	8.280	0.006	0.200	0.051	0.000	0.000	0.000	26.402
0.000	12.162	0.062	0.000	0.678	0.191	0.000	7.242	13.738	0.000	0.170	0.109	0.000	0.000	0.000	13.681
0.000	13.115	0.068	0.000	0.453	0.101	0.000	11.559	5.624	0.118	1.626	0.107	0.000	0.000	0.000	23.708
0.227	10.005	0.065	0.166	1.621	0.151	0.000	4.154	12.467	0.643	0.791	0.321	0.000	0.000	0.000	17.439
0.000	16.324	0.113	0.166	0.740	0.165	0.000	5.016	8.419	0.000	0.915	0.251	0.000	0.000	0.000	31.809
0.000	20.430	0.071	0.198	1.104	0.082	0.000	7.421	11.862	0.222	2.491	0.121	0.000	0.000	0.000	18.600
0.000	16.527	0.046	0.000	1.349	0.085	0.000	5.055	10.960	0.056	1.518	0.241	0.000	0.000	0.000	15.834
0.000	11.979	0.066	0.203	0.870	0.067	0.000	4.245	11.993	0.090	1.482	0.259	0.000	0.000	0.000	15.046
0.000	12.937	0.123	0.000	0.400	0.114	0.000	5.681	11.825	0.111	0.772	0.081	0.000	0.000	0.000	19.404
0.000	18.528	0.061	0.000	0.985	0.078	0.000	5.588	10.808	0.051	0.596	0.054	0.000	0.000	0.000	14.617
0.222	14.697	0.037	0.000	0.528	0.094	0.000	12.738	10.635	0.061	0.593	0.245	0.000	0.000	0.000	13.597
0.000	15.321	0.062	0.173	0.756	0.172	0.000	7.427	10.539	0.006	0.202	0.090	0.000	0.000	0.000	27.198
0.000	19.744	0.164	0.000	1.506	0.084	0.000	2.689	8.541	0.023	0.491	0.215	0.000	0.000	0.000	32.446
0.000	13.577	0.114	0.000	1.987	0.123	0.000	3.109	11.121	0.000	3.063	0.333	0.000	0.000	0.000	36.315
0.000	13.280	0.030	0.000	0.577	0.140	0.000	7.857	9.985	0.019	1.046	0.222	0.000	0.000	0.000	12.912
0.000	11.273	0.021	0.000	1.986	0.122	0.000	10.518	14.305	0.006	0.091	0.077	0.000	0.000	0.000	20.769
0.000	15.813	0.085	0.000	2.076	0.051	0.000	4.186	6.170	0.058	1.124	0.087	0.000	0.000	0.000	22.598
0.000	14.327	0.020	0.000	2.289	0.090	0.000	10.422	12.704	0.432	4.954	0.095	0.000	0.000	0.000	17.526
0.228	12.896	0.135	0.167	0.697	0.110	0.000	4.420	7.876	0.028	0.596	0.063	0.000	0.000	0.000	42.849
0.000	24.599	0.291	0.000	2.538	0.197	0.000	13.069	12.115	0.000	0.203	0.096	0.000	0.000	0.000	13.793
0.000	14.264	0.019	0.150	0.627	0.137	0.000	5.195	8.416	0.000	0.068	0.064	0.000	0.000	0.000	14.350
0.000	18.828	0.027	0.000	2.540	0.137	0.000	8.029	12.575	0.000	0.633	0.130	0.000	0.000	0.000	28.214
0.000	20.147	0.093	0.000	1.270	0.119	0.000	6.597	8.939	0.000	0.082	0.204	0.000	0.000	0.000	7.427
0.000	17.323	0.012	0.000	0.648	0.148	0.035	4.361	4.866	0.012	0.262	0.160	0.000	0.000	0.000	17.457
0.000	15.429	0.027	0.000	6.262	0.224	0.000	6.084	13.687	0.000	0.416	0.128	0.000	0.000	0.000	16.678
0.000	12.604	0.103	0.168	2.963	0.195	0.000	5.949	10.967	0.000	0.569	0.150	0.000	0.000	0.000	11.680
0.000	14.350	0.020	0.000	1.469	0.143	0.000	12.517	10.098	0.000	0.203	0.052	0.000	0.000	0.000	42.438
0.000	14.160	0.035	0.000	1.228	0.210	0.000	8.023	8.567	0.381	1.300	0.094	0.000	0.000	0.000	18.072
0.000	20.950	0.019	0.000	0.934	0.143	0.000	9.785	11.158	0.000	0.450	0.054	0.000	0.000	0.000	25.944
0.000	10.919	0.073	0.000	0.514	0.198	0.000	11.183	14.299	0.005	3.135	0.190	0.000	0.000	0.000	13.389
0.000	10.173	0.073	0.000	8.266	0.053	0.000	4.360	8.651	0.000	0.758	0.182	0.000	0.000	0.000	45.939
0.000	20.454	0.098	0.000	1.876	0.044	0.000	11.505	14.714	0.012	0.285	0.093	0.000	0.000	0.000	15.688
0.000	16.856	0.037	0.229	1.557	0.095	0.000	8.158	8.270	0.016	2.335	0.043	0.000	0.000	0.000	15.217
0.000	23.006	0.020	0.315	0.527	0.052	0.000	7.720	8.728	0.021	1.485	0.000	0.000	0.000	0.000	17.940
0.000	24.886	0.014	0.210	1.502	0.000	0.000	4.218	5.713	0.021	0.096	0.020	0.000	0.000	0.000	15.934
0.382	15.790	0.018	0.000	1.608	0.069	0.000	6.168	10.293	0.019	0.076	0.079	0.000	0.000	0.000	8.165
0.000	26.562	0.029	0.000	0.717	0.055	0.000	3.447	7.528	0.000	0.317	0.052	0.000	0.000	0.000	7.942
0.000	15.825	0.005	0.000	1.553	0.120	0.000	6.769	12.456	0.016	0.204	0.038	0.000	0.000	0.000	15.064
0.444	24.334	0.105	0.000	1.328	0.027	0.000	2.999	4.209	0.298	1.869	0.000	0.000	0.000	0.000	7.047
0.806	10.174	0.052	0.000	0.848	0.146	0.000	3.872	9.348	0.000	0.172	0.042	0.000	0.000	0.000	22.763
0.000	15.656	0.019	0.000	0.539	0.144	0.000	6.328	11.470	0.069	0.446	0.055	0.000	0.000	0.000	16.519
0.303	8.293	0.007	0.000	0.370	0.165	0.000	5.666	8.943	0.000	0.265	0.094	0.000	0.000	0.000	20.820
0.000	21.267	0.017	0.000	0.892	0.022	0.000	6.827	7.715	0.000	0.418	0.176	0.000	0.000	0.000	9.906
0.255	18.304	0.103	0.000	1.610	0.093	0.000	3.668	5.505	0.013	0.457	0.044	0.000	0.000	0.000	22.995
0.000	12.403	0.109	0.000	7.996	0.463	0.000	5.375	14.100	0.000	2.994	0.053	0.000	0.000	0.000	46.626
0.000	36.055	0.076	0.000	2.269	0.019	0.000	3.454	5.582	0.048	0.977	0.055	0.000	0.000	0.000	11.042
0.000	24.343	0.053	0.000	0.609	0.039	0.000	8.015	5.186	0.008	0.123	0.011	0.000	0.000	0.000	4.721
0.294	37.825	0.125	0.000	0.838	0.036	0.000	1.347	3.962	0.022	0.584	0.010	0.000	0.000	0.000	7.968
0.691	21.853	0.005	0.000	0.188	0.098	0.000	12.951	5.966	0.057	0.506	0.024	0.000	0.000	0.000	17.180
0.274	12.136	0.052	0.000	0.761	0.033	0.000	3.775	10.140	0.034	2.253	0.066	0.000	0.000	0.000	27.805
0.000	9.484	0.103	0.000	2.985	0.056	0.000	9.144	10.916	0.000	0.371	0.139	0.000	0.000	0.000	11.893
0.000	8.443	0.037	0.000	0.667	0.172	0.000	6.886	8.313	0.013	4.042	0.125	0.000	0.000	0.000	10.127
0.000	9.118	0.052	0.000	0.709	0.216	0.000	1.823	5.369	0.007	0.226	0.057	0.000	0.000	0.000	48.336
0.000	8.045	0.015	0.158	4.124	0.275	0.031	10.784	15.767	0.027	0.565	0.522	0.000	0.000	0.000	29.292
0.000	9.595	0.069	0.000	3.964	0.095	0.000	4.428	8.427	0.000	0.361	0.202	0.000	0.000	0.000	10.108
0.000	5.606	0.088	0.000	0.769	0.137	0.000	9.196	9.528	0.015	0.420	0.078	0.000	0.000	0.000	59.318
0.000	9.215	0.009	0.000	0.626	0.024	0.000	8.224	3.557	0.070	1.228	0.055	0.000	0.000	0.000	24.149
0.000	11.031	0.030	0.000	0.611	0.207	0.000	7.411	9.224	0.415	1.402	0.155	0.143	0.168	0.000	33.233
0.221	11.361	0.010	0.161	0.615	0.187	0.000	2.825	8.327	0.000	0.081	0.084	0.000	0.174	0.000	22.764
0.000	13.005	0.005	0.156	4.751	0.155	0.000	5.859	15.655	0.000	0.451	0.051	0.000	0.000	0.000	13.130
0.000	11.075	0.005	0.000	0.486	0.352	0.000	13.809	18.551	0.072	1.284	0.216	0.000	0.000	0.000	25.440
0.000	10.025	0.015	0.000	0.220	0.091	0.000	5.539	8.321	0.043	1.685	0.253	0.144	0.000	0.000	12.130
0.000	10.910	0.036	0.000	1.275	0.061	0.000	5.156	8.879	0.019	0.336	0.224	0.000	0.000	0.000	16.891
0.262	7.409	0.106	0.000	1.371	0.175	0.000	3.461	11.342	0.007	0.251	0.036	0.000	0.000	0.000	21.160
0.000	11.329	0.000	0.000	14.087	0.324	0.000	5.077	13.100	0.000	1.091	0.258	0.000	0.000	0.000	30.029
0.000	15.426	0.192	0.000	8.181	0.164	0.098	5.514	8.279	0.101	0.726	0.327	0.000	0.000	0.000	13.973
0.000	12.402	0.019	0.000	0.803	0.111	0.000	7.858	10.322	0.013	0.889	0.063	0.000	0.000	0.000	21.279
1.047	11.988	0.093	0.000	1.760	0.143	0.000	8.684	13.534	0.000	0.414	0.217	0.000	0.000	0.000	44.393
0.000	37.749	0.146	0.000	1.323	0.000	0.000	1.974	3.439	0.008	0.117	0.059	0.000	0.000	0.000	9.349
0.222	12.734	0.095	0.000	1.765	0.229	0.000	1.773	9.880	0.011	1.416	0.199	0.000	0.000	0.000	16.302
0.000	13.416	0.081	0.000	1.186	0.156	0.000	6.738	4.942	0.014	0.566	0.197	0.000	0.000	0.000	9.560
0.000	12.897	0.085	0.000	0.803	0.119	0.000	5.197	5.136	0.000	0.092	0.170	0.000	0.000	0.000	22.858
0.575	13.149	0.027	0.000	1.641	0.104	0.000	6.147	11.579	0.057	0.425	0.079	0.000	0.000	0.000	21.774
0.790	15.369	0.037	0.000	2.379	0.271	0.000	6.627	10.981	0.020	0.159	0.054	0.000	0.000	0.042	8.196
0.234	18.028	0.011	0.000	0.921	0.057	0.000	4.924	9.822	0.000	0.153	0.105	0.000	0.000	0.000	9.882
0.000	11.949	0.031	0.000	0.432	0.133	0.000	12.365	10.706	0.005	0.489	0.091	0.000	0.000	0.000	23.227
0.275	10.325	0.033	0.000	1.755	0.083	0.000	3.296	4.743	0.020	0.510	0.038	0.000	0.000	0.000	30.823
0.000	22.331	0.037	0.379	2.271	0.016	0.000	3.958	5.402	0.000	0.097	0.000	0.000	0.000	0.000	6.153
0.000	16.212	0.184	0.178	0.545	0.103	0.000	6.212	9.599	0.006	0.486	0.050	0.000	0.000	0.000	29.393
0.000	15.991	0.012	0.000	0.393	0.076	0.000	5.524	9.124	0.081	0.869	0.043	0.000	0.000	0.000	9.273
0.000	9.951	0.280	0.787	0.914	0.098	0.000	7.006	7.405	0.013	0.875	0.074	0.000	0.000	0.000	20.025
0.000	15.137	0.067	0.000	2.189	0.217	0.000	4.304	11.335	0.000	1.097	0.071	0.000	0.000	0.000	13.464
0.000	11.863	0.064	0.000	0.867	0.033	0.000	5.393	7.810	0.000	0.068	0.037	0.000	0.000	0.000	12.478
0.000	11.321	0.022	0.000	0.398	0.113	0.000	8.949	11.101	0.832	1.496	0.016	0.000	0.000	0.000	17.882
0.271	11.781	0.096	0.000	0.221	0.115	0.000	11.903	11.279	0.027	1.949	0.065	0.000	0.000	0.000	28.753
0.000	18.942	0.061	0.000	0.767	0.085	0.000	9.501	13.366	0.006	0.248	0.057	0.000	0.000	0.000	35.170
0.000	12.919	0.037	0.000	1.368	0.163	0.000	7.119	13.649	0.006	0.359	0.054	0.000	0.000	0.000	20.381
0.000	11.878	0.048	0.148	1.169	0.172	0.000	5.634	14.142	0.010	0.652	0.091	0.000	0.000	0.000	51.285
0.000	14.628	0.036	0.000	1.118	0.185	0.000	4.997	9.651	0.015	0.597	0.032	0.000	0.000	0.000	23.758
0.267	9.019	0.379	0.000	0.345	0.210	0.000	4.453	11.249	0.020	0.932	0.101	0.000	0.000	0.000	36.340
0.000	29.041	0.006	0.000	6.180	0.096	0.000	6.910	8.171	0.020	0.221	0.064	0.000	0.000	0.000	5.680
0.000	11.577	0.029	0.182	1.133	0.090	0.000	11.738	11.213	0.130	0.882	0.129	0.000	0.000	0.000	15.526
0.237	11.299	0.051	0.000	0.258	0.057	0.000	6.832	7.203	0.342	4.038	0.057	0.000	0.000	0.000	14.618
0.000	14.515	0.037	0.000	0.684	0.094	0.000	7.821	6.256	0.019	0.661	0.133	0.000	0.000	0.000	64.954
0.000	9.982	0.068	0.000	2.641	0.198	0.059	4.579	10.420	0.030	0.648	0.198	0.000	0.000	0.000	18.874
0.000	10.665	0.036	0.000	1.547	0.287	0.000	10.618	10.450	0.009	0.144	0.092	0.000	0.000	0.000	10.465
0.000	18.071	0.044	0.000	0.990	0.448	0.000	1.750	12.732	0.020	1.271	0.128	0.000	0.000	0.000	26.621
0.000	7.204	0.065	0.000	0.252	0.071	0.000	4.336	8.820	0.029	0.224	0.094	0.000	0.000	0.000	21.582
0.000	19.225	0.073	0.000	3.219	0.037	0.000	2.694	7.667	0.000	0.224	0.106	0.000	0.000	0.000	11.194
0.000	35.897	0.068	0.000	0.710	0.067	0.000	3.950	6.511	0.006	0.739	0.077	0.000	0.000	0.000	16.942
0.000	9.937	0.014	0.000	2.793	0.311	0.000	8.604	12.454	0.007	1.942	0.167	0.000	0.224	0.000	22.958
0.291	13.863	0.021	0.000	0.810	0.088	0.000	2.615	6.702	0.014	0.130	0.161	0.000	0.000	0.000	17.594
0.000	15.259	0.115	0.000	3.182	0.137	0.000	6.340	8.354	0.000	0.330	0.100	0.000	0.000	0.000	19.022
0.000	7.616	0.034	0.000	1.700	0.066	0.000	2.923	11.343	0.189	0.831	0.150	0.000	0.000	0.000	12.365
0.000	14.722	0.014	0.000	0.540	0.161	0.000	11.228	11.158	0.022	2.789	0.102	0.000	0.000	0.000	17.108
0.000	15.199	0.024	0.000	0.920	0.199	0.030	3.828	7.214	0.000	0.383	0.106	0.000	0.000	0.000	24.715
0.000	16.359	0.020	0.000	0.357	0.038	0.000	9.713	10.512	0.199	1.655	0.022	0.000	0.000	0.000	7.191
0.000	15.717	0.044	0.000	0.868	0.227	0.000	8.178	9.679	0.000	0.665	0.052	0.000	0.000	0.000	21.742
0.000	10.748	0.246	0.000	1.569	0.261	0.000	4.467	9.806	0.000	0.398	0.081	0.000	0.000	0.000	9.414
0.000	12.503	0.038	0.000	1.207	0.241	0.000	13.445	13.270	0.007	0.312	0.091	0.000	0.000	0.000	8.637
0.000	13.297	0.045	0.174	1.699	0.231	0.000	7.406	11.345	0.160	0.616	0.238	0.000	0.000	0.000	16.144
0.000	22.441	0.033	0.000	0.858	0.042	0.000	7.265	10.405	0.006	0.205	0.032	0.000	0.000	0.000	13.807

ENSG00000164825	ENSG00000228793	ENSG00000255899	ENSG00000227979	ENSG00000179902	ENSG00000233499	ENSG00000203585	ENSG00000101460	ENSG00000179919	ENSG00000271889	ENSG00000244720	ENSG00000224297	ENSG00000232557	ENSG00000275409	ENSG00000172809	ENSG00000106278
9.475	0.125	0.000	0.000	6.192	0.000	0.033	23.808	0.000	0.112	0.000	0.000	0.000	0.850	56.906	145.235
0.000	0.320	0.000	0.000	0.443	0.000	0.018	23.968	0.000	0.024	0.022	0.000	0.100	0.152	106.047	8.203
0.118	0.404	0.000	0.000	1.790	0.000	0.025	4.794	0.000	0.021	0.000	0.000	0.000	0.305	145.379	118.122
0.089	1.022	0.000	0.000	2.611	0.000	0.028	17.545	0.000	0.120	0.000	0.000	0.000	0.499	87.083	172.467
1.728	2.154	0.000	0.000	12.594	0.000	0.021	21.967	0.000	0.107	0.000	0.000	0.000	0.811	102.515	46.067
0.156	0.473	0.000	0.166	0.951	0.000	0.016	2.032	0.000	0.127	0.039	0.000	0.089	0.472	58.596	162.763
0.747	0.080	0.000	0.000	0.476	0.000	0.028	22.420	0.000	0.285	0.022	0.000	0.100	0.228	55.018	4.725
0.139	1.380	0.000	0.000	1.835	0.000	0.000	38.290	0.000	0.201	0.000	0.000	0.000	0.683	89.649	55.740
0.107	1.301	0.000	0.000	3.617	0.028	0.045	32.707	0.000	0.203	0.000	0.000	0.000	0.603	110.899	158.521
0.625	1.231	0.000	0.000	0.363	0.000	0.000	22.688	0.000	0.254	0.000	0.000	0.000	0.676	108.247	40.137
0.115	0.372	0.000	0.000	1.531	0.000	0.040	23.152	0.000	0.270	0.000	0.000	0.000	0.531	77.796	159.403
0.119	0.313	0.000	0.000	0.585	0.020	0.017	32.346	0.000	0.043	0.020	0.000	0.000	0.515	51.354	415.942
0.123	0.149	0.000	0.000	2.572	0.000	0.086	28.079	0.000	0.177	0.020	0.038	0.000	0.213	42.870	131.012
0.038	0.930	0.000	0.000	2.005	0.000	0.008	43.808	0.000	0.228	0.000	0.000	0.000	0.597	39.648	156.997
0.171	0.725	0.000	0.000	7.941	0.000	0.000	44.434	0.000	0.092	0.000	0.000	0.000	0.148	77.456	77.596
0.230	1.489	0.000	0.000	1.323	0.000	0.032	16.191	0.000	0.208	0.115	0.000	0.000	0.432	45.135	52.419
0.044	0.805	0.000	0.094	1.234	0.000	0.000	20.473	0.000	0.048	0.000	0.000	0.000	0.383	74.413	269.554
0.368	1.147	0.000	0.000	5.718	0.000	0.000	13.635	0.000	0.057	0.026	0.000	0.000	0.273	257.920	43.904
0.080	3.127	0.000	0.000	1.053	0.000	0.000	34.524	0.000	0.195	0.000	0.000	0.000	0.138	64.433	143.090
0.000	0.207	0.000	0.000	2.268	0.000	0.000	22.265	0.000	0.162	0.000	0.000	0.000	0.555	42.276	147.961
0.091	3.259	0.000	0.000	5.012	0.000	0.000	35.054	0.000	0.222	0.000	0.042	0.000	0.355	158.298	108.416
0.000	1.256	0.000	0.000	1.095	0.000	0.039	19.921	0.000	0.178	0.023	0.000	0.000	0.407	48.490	166.650
0.230	0.698	0.000	0.000	2.442	0.000	0.019	24.087	0.000	0.349	0.000	0.000	0.105	0.279	124.393	122.808
0.000	0.095	0.000	0.000	0.349	0.000	0.016	14.997	0.000	0.148	0.000	0.000	0.000	0.203	61.935	110.097
0.087	0.291	0.000	0.000	0.709	0.000	0.000	38.174	0.000	0.000	0.022	0.000	0.099	0.829	68.241	92.450
0.000	0.495	0.000	0.000	2.809	0.000	0.028	22.837	0.000	0.294	0.000	0.042	0.000	0.470	50.149	196.373
0.256	0.000	0.000	0.000	21.800	0.026	0.021	29.738	0.000	0.166	0.000	0.000	0.000	0.576	68.046	80.572
0.271	0.876	0.000	0.000	1.154	0.000	0.057	32.271	0.000	0.147	0.000	0.000	0.000	0.390	80.464	166.535
0.045	0.055	0.000	0.000	7.521	0.000	0.019	49.131	0.000	0.584	0.045	0.000	0.000	1.672	58.341	111.627
0.000	1.151	0.000	0.000	2.294	0.000	0.019	22.251	0.000	0.171	0.000	0.000	0.000	0.274	156.851	204.974
0.000	0.564	0.000	0.000	3.529	0.000	0.008	69.095	0.000	0.141	0.000	0.000	0.000	0.386	94.806	231.932
0.000	0.260	0.000	0.000	0.860	0.020	0.000	53.543	0.000	0.358	0.000	0.000	0.000	1.315	65.466	187.279
0.045	0.990	0.000	0.000	7.622	0.000	0.019	82.856	0.000	0.049	0.000	0.000	0.000	1.373	94.206	77.237
0.000	0.238	0.000	0.000	7.600	0.000	0.018	34.986	0.000	0.260	0.000	0.040	0.000	0.945	73.368	71.558
0.000	0.615	0.000	0.000	3.896	0.000	0.019	23.577	0.000	0.100	0.000	0.000	0.000	0.798	49.048	281.754
0.000	2.889	0.000	0.000	5.182	0.000	0.026	40.279	0.000	0.204	0.042	0.000	0.000	0.651	62.160	170.101
0.120	0.073	0.000	0.000	3.263	0.041	0.050	15.067	0.000	1.059	0.000	0.000	0.000	0.622	31.739	159.087
0.080	1.305	0.000	0.000	3.027	0.000	0.017	28.135	0.020	0.324	0.040	0.000	0.000	0.931	54.214	78.308
0.143	1.154	0.000	0.101	3.740	0.000	0.040	27.882	0.024	0.154	0.000	0.000	0.000	0.247	83.497	193.542
0.098	1.222	0.000	0.000	1.237	0.000	0.010	16.745	0.000	0.585	0.000	0.000	0.000	0.595	66.642	294.283
0.147	0.267	0.000	0.000	7.254	0.000	0.031	25.729	0.000	0.397	0.000	0.000	0.000	0.465	47.621	243.173
0.041	0.251	0.000	0.000	1.361	0.000	0.043	30.150	0.000	0.134	0.000	0.038	0.000	0.393	56.281	219.609
0.091	0.193	0.000	0.000	0.846	0.000	0.019	29.263	0.000	0.320	0.000	0.000	0.000	0.315	44.887	295.058
0.000	0.118	0.000	0.000	2.948	0.000	0.025	16.521	0.000	0.169	0.000	0.000	0.000	0.338	49.074	379.260
0.083	0.454	0.000	0.000	1.950	0.000	0.078	20.869	0.000	0.180	0.021	0.038	0.000	0.900	54.523	367.175
0.081	0.344	0.068	0.000	5.154	0.000	0.034	16.213	0.000	0.483	0.000	0.000	0.000	0.562	58.464	137.806
0.179	0.515	0.000	0.000	0.415	0.000	0.019	11.947	0.000	0.363	0.000	0.000	0.000	0.155	151.180	2.810
0.136	0.411	0.000	0.000	1.383	0.000	0.057	8.224	0.000	0.196	0.000	0.000	0.000	0.587	80.316	156.559
0.000	0.428	0.000	0.000	1.868	0.000	0.033	35.891	0.000	0.042	0.000	0.000	0.000	0.339	68.862	259.199
0.148	4.063	0.000	0.000	0.574	0.000	0.031	26.866	0.000	0.080	0.000	0.000	0.000	0.096	79.916	70.350
0.224	3.499	0.000	0.000	2.005	0.000	0.031	63.588	0.000	0.242	0.000	0.000	0.000	0.515	91.003	65.616
0.040	0.170	0.000	0.000	0.729	0.000	0.034	17.932	0.000	0.043	0.020	0.000	0.000	0.139	65.800	198.201
0.041	0.272	0.000	0.000	7.768	0.021	0.017	22.199	0.000	0.022	0.000	0.000	0.000	0.141	89.580	174.321
0.000	0.764	0.000	0.000	1.312	0.000	0.008	32.824	0.000	0.039	0.018	0.000	0.000	0.249	50.273	349.657
0.199	0.402	0.000	0.141	2.159	0.000	0.042	17.111	0.000	0.287	0.000	0.061	0.000	0.344	114.598	142.127
0.000	0.524	0.000	0.000	3.012	0.000	0.000	10.979	0.000	0.374	0.000	0.000	0.000	0.299	124.056	159.392
0.043	0.469	0.000	0.000	0.849	0.000	0.000	25.573	0.000	0.163	0.000	0.000	0.000	0.409	92.835	302.328
0.081	0.148	0.000	0.000	2.327	0.000	0.017	21.431	0.000	0.154	0.000	0.000	0.000	9.725	76.473	177.755
0.081	2.494	0.000	0.000	8.810	0.000	0.059	16.466	0.000	0.087	0.020	0.000	0.000	0.523	54.597	154.737
0.038	0.274	0.000	0.000	8.860	0.000	0.016	29.651	0.000	0.061	0.038	0.000	0.000	0.423	53.244	136.170
0.000	0.132	0.000	0.000	2.442	0.000	0.073	27.600	0.022	0.118	0.000	0.000	0.000	0.038	47.235	104.196
0.000	0.460	0.000	0.000	1.790	0.000	0.014	26.559	0.000	0.075	0.000	0.000	0.000	0.597	123.157	150.308
0.108	0.457	0.000	0.000	1.141	0.000	0.008	38.897	0.000	0.136	0.000	0.000	0.000	0.466	73.230	216.708
0.116	0.070	0.000	0.082	1.939	0.020	0.105	20.023	0.000	0.479	0.019	0.000	0.088	0.133	61.175	70.783
0.043	0.599	0.000	0.000	2.694	0.022	0.045	29.777	0.000	0.163	0.000	0.039	0.000	1.003	71.359	134.313
0.055	3.377	0.000	0.000	1.367	0.000	0.023	52.642	0.028	0.060	0.000	0.000	0.000	0.143	108.707	103.914
0.000	2.247	0.000	0.000	1.377	0.000	0.016	42.860	0.000	0.123	0.000	0.000	0.000	0.458	92.651	255.702
0.000	0.550	0.000	0.000	0.938	0.000	0.000	49.195	0.000	0.109	0.000	0.000	0.000	0.218	121.104	185.741
0.201	0.488	0.000	0.000	1.740	0.000	0.000	30.509	0.000	0.073	0.000	0.000	0.000	1.044	47.446	228.200
0.000	1.028	0.000	0.000	4.329	0.000	0.000	42.217	0.000	0.057	0.000	0.000	0.000	0.137	151.506	219.564
0.077	0.070	0.000	0.000	4.904	0.000	0.000	29.114	0.000	0.063	0.000	0.000	0.000	0.167	101.994	316.182
0.859	1.656	0.000	0.000	21.761	0.000	0.016	53.447	0.000	0.084	0.000	0.000	0.000	0.135	124.866	32.508
0.000	0.214	0.000	0.000	1.877	0.018	0.022	22.854	0.000	0.134	0.000	0.000	0.000	0.367	59.640	188.718
0.279	0.211	0.000	0.000	3.429	0.000	0.015	34.969	0.000	0.038	0.000	0.000	0.159	0.301	67.710	237.324
0.053	0.258	0.000	0.000	0.515	0.000	0.000	19.918	0.000	0.058	0.000	0.049	0.121	0.276	55.725	206.732
0.833	0.194	0.000	0.000	2.481	0.000	0.054	20.001	0.000	0.277	0.032	0.000	0.000	0.443	65.140	212.519
0.045	0.353	0.000	0.000	2.550	0.000	0.000	35.789	0.000	0.145	0.000	0.000	0.000	0.271	136.187	83.100
0.000	0.163	0.000	0.000	1.144	0.000	0.000	15.383	0.000	0.581	0.000	0.000	0.000	0.000	144.834	22.941
0.677	0.752	0.000	0.000	2.775	0.000	0.024	36.986	0.000	0.122	0.000	0.000	0.000	0.537	79.447	177.623
0.056	0.136	0.000	0.000	10.546	0.000	0.024	40.699	0.000	0.183	0.000	0.000	0.000	0.243	130.516	74.995
0.052	0.688	0.000	0.000	12.028	0.000	0.022	29.983	0.000	0.112	0.000	0.000	0.000	0.134	146.617	129.932
0.243	0.736	0.000	0.000	6.423	0.000	0.000	43.470	0.000	0.044	0.000	0.000	0.000	0.455	120.785	150.570
0.288	2.126	0.000	0.000	3.032	0.000	0.010	25.764	0.000	0.104	0.000	0.000	0.219	0.706	66.967	76.644
0.109	1.318	0.000	0.000	2.400	0.000	0.023	22.285	0.000	0.324	0.027	0.000	0.000	0.282	49.889	104.072
0.091	6.911	0.000	0.000	0.950	0.023	0.000	24.005	0.000	0.197	0.000	0.000	0.000	0.864	48.895	100.157
0.048	0.234	0.000	0.000	3.308	0.000	0.020	21.536	0.000	0.078	0.024	0.000	0.000	0.209	59.607	57.376
0.076	2.370	0.000	0.000	0.559	0.000	0.008	17.957	0.000	0.103	0.000	0.000	0.000	0.624	47.172	367.130
0.475	2.496	0.000	0.000	3.128	0.000	0.158	13.916	0.000	0.214	0.000	0.000	0.000	0.205	76.990	22.373
0.036	0.329	0.061	0.000	1.512	0.000	0.023	21.909	0.000	0.117	0.000	0.000	0.165	0.657	69.574	209.738
0.070	2.007	0.000	0.000	2.155	0.000	0.030	23.251	0.000	0.076	0.000	0.000	0.000	0.914	62.198	153.993
0.151	0.684	0.000	0.080	1.107	0.000	0.008	26.980	0.000	0.407	0.000	0.000	0.000	1.561	61.950	340.726
0.000	0.400	0.000	0.000	3.834	0.000	0.024	31.553	0.000	0.168	0.000	0.000	0.000	0.537	83.875	63.724
0.075	0.727	0.000	0.000	1.814	0.000	0.000	4.495	0.000	0.101	0.000	0.000	0.000	0.421	81.294	78.868
0.079	0.167	0.000	0.251	2.573	0.000	0.008	17.114	0.000	0.319	0.039	0.000	0.000	0.544	101.958	385.439
0.000	0.965	0.000	0.000	1.702	0.000	0.032	7.025	0.000	0.164	0.000	0.000	0.000	1.835	81.842	99.899
0.088	0.400	0.000	0.000	4.988	0.000	0.046	27.491	0.000	0.333	0.000	0.000	0.100	0.912	62.150	131.522
0.000	0.223	0.000	0.000	1.908	0.000	0.000	1.538	0.000	0.125	0.023	0.000	0.000	0.199	120.240	130.264
0.188	1.821	0.000	0.000	4.218	0.000	0.000	30.230	0.000	0.102	0.000	0.000	0.000	1.462	79.712	195.740
0.238	0.649	0.000	0.000	15.791	0.000	0.000	24.362	0.000	0.129	0.000	0.000	0.000	0.822	75.609	110.299
0.000	0.448	0.000	0.000	4.432	0.000	0.048	31.840	0.000	0.225	0.000	0.000	0.000	0.519	52.555	296.031
0.000	0.111	0.000	0.000	2.671	0.000	0.019	10.609	0.000	0.149	0.046	0.000	0.000	0.636	53.755	129.277
0.000	0.000	0.000	0.000	6.953	0.000	0.038	28.345	0.000	0.260	0.000	0.000	0.000	0.260	154.635	401.111
0.156	0.000	0.000	0.000	50.009	0.000	0.000	32.457	0.000	0.697	0.000	0.000	0.000	0.405	78.028	37.666
0.201	0.305	0.000	0.000	42.865	0.000	0.042	24.748	0.000	0.109	0.000	0.000	0.000	0.652	55.767	123.418
0.000	0.175	0.000	0.000	1.563	0.030	0.036	42.544	0.000	0.312	0.000	0.053	0.000	1.995	87.076	302.414
0.101	0.551	0.000	0.000	2.113	0.000	0.000	23.918	0.000	0.109	0.050	0.000	0.230	0.350	58.730	293.112
0.046	0.112	0.000	0.000	48.708	0.024	0.058	38.007	0.000	0.375	0.000	0.000	0.000	0.360	71.814	141.681
0.041	0.324	0.000	0.000	0.556	0.000	0.017	4.135	0.000	0.178	0.000	0.000	0.000	0.604	124.700	131.721
0.000	0.771	0.000	0.000	1.045	0.000	0.008	38.090	0.000	0.021	0.000	0.000	0.000	0.567	70.214	447.091
0.048	0.322	0.000	0.000	0.598	0.025	0.010	34.302	0.000	0.052	0.000	0.000	0.000	0.376	148.214	116.057
0.091	0.497	0.000	0.000	9.117	0.000	0.000	51.613	0.000	0.000	0.000	0.000	0.000	0.276	121.559	313.436
0.000	0.466	0.000	0.000	2.019	0.000	0.000	31.400	0.000	0.208	0.000	0.000	0.000	0.407	74.494	243.468
0.044	1.392	0.000	0.000	4.001	0.023	0.009	22.675	0.000	0.239	0.000	0.000	0.000	0.420	64.581	188.491
0.095	0.516	0.000	0.000	1.099	0.000	0.020	17.457	0.000	0.102	0.000	0.000	0.000	0.327	48.790	219.314
0.000	0.300	0.000	0.000	4.158	0.023	0.000	41.974	0.000	0.170	0.022	0.000	0.000	0.117	71.402	166.880
0.191	0.260	0.000	0.000	1.644	0.000	0.030	45.725	0.000	0.026	0.000	0.000	0.109	0.289	60.925	116.416
0.000	0.698	0.000	0.000	1.004	0.000	0.009	40.683	0.000	0.134	0.000	0.038	0.000	0.961	46.535	255.662
0.048	0.260	0.000	0.000	6.624	0.000	0.020	33.666	0.000	0.129	0.000	0.000	0.000	1.113	80.674	150.629
0.041	0.226	0.000	0.000	0.961	0.000	0.026	19.089	0.000	0.134	0.021	0.000	0.000	0.250	84.692	218.706
0.000	0.215	0.000	0.000	1.112	0.000	0.008	19.363	0.000	0.128	0.000	0.036	0.000	0.204	55.363	283.855
0.142	0.582	0.000	0.000	8.982	0.000	0.022	13.489	0.000	0.096	0.018	0.000	0.000	0.215	52.170	178.184
0.214	0.584	0.000	0.000	2.282	0.000	0.022	26.475	0.000	0.029	0.000	0.000	0.000	0.371	83.823	119.760
0.094	0.284	0.000	0.000	1.090	0.000	0.029	20.113	0.000	0.051	0.023	0.000	0.000	0.609	95.920	3.300
0.746	0.311	0.000	0.000	22.016	0.000	0.010	20.263	0.000	0.101	0.000	0.000	0.000	0.524	72.516	74.326
0.000	0.133	0.147	0.000	2.219	0.045	0.018	31.605	0.000	0.402	0.022	0.040	0.000	0.265	45.986	202.411
0.167	0.733	0.000	0.000	2.148	0.000	0.009	44.032	0.000	0.135	0.000	0.000	0.000	1.299	80.451	98.458
0.000	0.082	0.000	0.000	1.633	0.000	0.085	30.046	0.000	0.147	0.000	0.000	0.000	0.353	81.277	123.486
0.000	0.719	0.061	0.000	20.466	0.019	0.023	21.508	0.000	0.331	0.018	0.000	0.082	0.777	69.950	102.049
0.000	0.404	0.000	0.000	5.991	0.000	0.014	12.788	0.000	0.036	0.000	0.000	0.000	0.404	61.614	157.371
0.108	1.531	0.000	0.000	9.543	0.000	0.000	31.634	0.000	0.059	0.000	0.000	0.082	0.530	76.413	138.536
0.000	0.541	0.000	0.000	0.917	0.000	0.050	31.030	0.000	0.149	0.000	0.000	0.000	0.534	53.056	185.594
0.000	0.131	0.000	0.000	0.963	0.000	0.023	28.613	0.000	0.058	0.000	0.000	0.000	0.281	77.571	107.872
0.000	0.403	0.000	0.000	1.830	0.000	0.016	25.053	0.000	0.042	0.000	0.000	0.000	0.068	129.383	247.502
0.050	4.349	0.000	0.000	0.816	0.000	0.021	56.451	0.000	0.353	0.000	0.000	0.000	0.434	107.143	113.925
0.051	0.279	0.000	0.000	1.207	0.000	0.000	44.189	0.000	0.221	0.000	0.000	0.000	0.619	76.384	157.611
0.057	0.378	0.000	0.000	8.285	0.000	0.012	24.862	0.000	0.338	0.028	0.000	0.000	0.442	81.028	91.888
0.000	0.155	0.000	0.000	2.962	0.000	0.040	31.488	0.000	0.310	0.000	0.000	0.000	1.158	49.377	100.089
0.104	1.349	0.000	0.000	0.802	0.000	0.033	31.302	0.026	0.112	0.000	0.000	0.118	0.627	96.485	234.421
0.000	0.437	0.000	0.000	0.601	0.000	0.008	28.613	0.000	0.117	0.000	0.000	0.082	0.437	84.897	719.296
0.000	0.493	0.000	0.000	17.414	0.000	0.031	28.878	0.000	0.280	0.000	0.000	0.084	0.096	57.008	169.600
0.066	2.840	0.000	0.000	6.211	0.000	0.028	19.473	0.000	0.036	0.000	0.061	0.000	0.000	79.089	233.115
0.000	0.542	0.000	0.000	0.813	0.000	0.014	20.838	0.000	0.186	0.034	0.032	0.078	0.446	81.974	194.381
0.000	0.904	0.000	0.000	50.239	0.000	0.176	39.347	0.000	0.076	0.000	0.000	0.000	0.524	58.177	122.962
0.042	0.127	0.000	0.000	3.323	0.000	0.018	28.646	0.000	0.204	0.000	0.000	0.000	0.652	64.886	302.741
0.000	0.050	0.000	0.000	3.835	0.000	0.009	21.885	0.000	0.089	0.000	0.000	0.000	0.426	70.831	268.096

ENSG00000222317	ENSG00000253930	ENSG00000278526	ENSG00000233047	ENSG00000212518	ENSG00000136052	ENSG00000200545	ENSG00000266107	ENSG00000227721	ENSG00000255374	ENSG00000231978	ENSG00000169223	ENSG00000199483	ENSG00000230828	ENSG00000260721	ENSG00000066136
0.908	0.155	0.000	0.000	0.000	3.950	0.000	0.330	0.000	0.044	0.000	41.624	0.000	0.000	0.021	7.472
0.000	0.496	0.000	0.000	0.000	2.582	0.000	0.000	0.000	0.000	0.000	97.784	0.000	0.000	0.035	6.776
0.516	0.206	0.000	0.000	0.000	1.665	0.000	0.000	0.000	0.067	0.000	50.906	0.000	0.054	0.000	9.211
1.557	0.466	0.000	0.000	0.000	2.867	0.000	0.000	0.000	0.038	0.000	45.695	0.000	0.000	0.000	8.490
0.217	0.111	0.000	0.000	0.000	2.772	0.000	0.000	0.000	0.000	0.000	71.173	0.000	0.000	0.020	6.367
5.305	0.205	0.000	0.000	0.000	1.680	0.000	0.249	0.000	0.050	0.000	47.940	0.000	0.054	0.000	12.593
0.000	0.528	0.000	0.000	0.000	3.221	0.000	0.000	0.000	0.038	0.137	64.937	0.000	0.000	0.000	10.594
0.408	0.314	0.000	0.000	0.000	2.609	0.000	0.000	0.000	0.000	0.000	43.977	0.000	0.000	0.056	7.414
0.236	0.121	0.000	0.000	0.000	1.864	0.000	0.000	0.000	0.046	0.000	52.091	0.000	0.000	0.000	7.420
0.000	0.117	0.000	0.000	0.000	2.190	0.000	0.000	0.000	0.000	0.000	76.465	0.000	0.000	0.000	6.920
0.337	0.288	0.000	0.000	0.000	4.234	0.000	0.245	0.000	0.000	0.000	64.862	0.000	0.000	0.015	7.785
2.267	0.448	0.000	0.000	0.000	2.446	0.000	0.000	0.000	0.034	0.062	62.479	0.000	0.000	0.016	7.175
0.720	0.185	0.000	0.000	0.000	2.486	0.000	0.000	0.000	0.018	0.000	52.555	0.000	0.000	0.016	6.996
1.514	0.173	0.000	0.000	0.000	2.407	0.000	0.000	0.000	0.049	0.060	43.923	0.000	0.000	0.000	7.770
0.000	0.257	0.000	0.000	0.000	2.047	0.000	0.000	0.000	0.000	0.000	72.224	0.000	0.000	0.411	9.859
0.337	1.210	0.000	0.000	0.000	2.837	0.000	0.000	0.000	0.033	0.060	49.528	0.000	0.000	0.031	6.223
0.388	0.133	0.000	0.000	0.000	1.919	0.000	0.000	0.000	0.000	0.000	67.255	0.000	0.000	0.000	8.387
0.231	0.276	0.000	0.000	0.000	0.673	0.000	0.000	0.000	0.023	0.000	67.748	0.000	0.000	0.021	7.360
0.526	0.210	0.000	0.000	0.000	0.798	0.000	0.000	0.000	0.017	0.000	83.626	0.000	0.000	0.016	9.247
0.188	0.096	0.000	0.000	0.000	2.246	0.000	0.000	0.000	0.018	0.067	52.866	0.000	0.000	0.034	13.146
0.000	0.205	0.000	0.000	0.000	1.663	0.000	0.291	0.000	0.039	0.000	63.071	0.000	0.000	0.037	7.173
2.894	0.177	0.000	0.000	0.000	2.117	0.000	0.300	0.000	0.020	0.000	29.144	0.000	0.065	0.132	9.100
0.202	0.069	0.000	0.000	0.000	1.598	0.000	0.000	0.000	0.020	0.000	56.280	0.000	0.000	0.185	8.932
0.000	1.205	0.000	0.000	0.000	2.156	0.000	0.000	0.000	0.017	0.000	104.874	0.000	0.000	0.000	8.105
0.000	0.262	0.000	0.000	0.000	2.020	0.000	0.000	0.000	0.000	0.068	68.849	0.000	0.000	0.000	7.333
1.790	0.136	0.000	0.000	0.000	4.066	0.000	0.289	0.000	0.136	0.000	59.927	0.000	0.063	0.527	8.428
0.000	0.192	0.000	0.000	0.000	2.390	0.000	0.000	0.000	0.022	0.000	58.881	0.000	0.000	0.021	8.631
0.000	0.203	0.000	0.000	0.000	1.199	0.000	0.000	0.000	0.078	0.000	61.383	0.000	0.000	0.036	8.823
0.395	0.101	0.000	0.000	0.000	1.878	0.000	0.000	0.000	0.000	0.000	58.140	0.000	0.000	0.000	7.562
1.587	0.102	0.000	0.000	0.000	1.667	0.000	0.000	0.000	0.058	0.000	40.590	0.000	0.000	0.000	6.468
0.490	0.056	0.000	0.000	0.000	1.775	0.000	0.000	0.000	0.032	0.000	99.290	0.000	0.000	0.000	9.677
1.197	0.088	0.000	0.000	0.000	2.161	0.000	0.000	0.055	0.000	0.000	93.032	0.000	0.000	0.000	6.805
0.000	0.136	0.000	0.000	0.000	1.583	0.000	0.000	0.065	0.000	0.000	46.204	0.000	0.000	0.018	7.696
0.192	0.131	0.000	0.000	0.000	2.609	0.096	0.000	0.000	0.075	0.000	74.187	0.000	0.000	0.000	7.014
2.025	0.624	0.000	0.000	0.000	5.048	0.000	0.000	0.000	0.079	0.000	66.512	0.000	0.000	0.111	8.178
0.367	0.722	0.000	0.000	0.000	4.507	0.000	0.000	0.000	0.036	0.000	44.364	0.000	0.000	0.201	8.170
4.556	0.090	0.000	0.073	0.000	1.898	0.000	0.255	0.000	0.240	0.000	41.253	0.000	0.000	0.000	11.057
1.575	0.419	0.000	0.000	0.000	1.896	0.000	0.000	0.000	0.017	0.000	58.275	0.000	0.055	0.000	14.444
0.418	0.607	0.000	0.000	0.000	2.249	0.000	0.303	0.000	0.000	0.222	51.110	0.000	0.000	0.038	8.910
2.588	0.148	0.000	0.000	0.000	2.300	0.000	0.000	0.000	0.042	0.000	45.932	0.000	0.000	0.000	7.261
2.146	0.441	0.000	0.022	0.000	9.191	0.000	0.312	0.000	0.021	0.000	47.379	0.000	0.000	0.020	8.897
0.726	0.248	0.000	0.000	0.000	3.961	0.000	0.000	0.000	0.089	0.000	63.371	0.000	0.000	0.050	4.669
3.194	0.376	0.000	0.000	0.000	1.909	0.000	0.000	0.000	0.020	0.000	56.185	0.000	0.000	0.073	8.938
1.713	0.440	0.000	0.000	0.000	2.712	0.000	0.498	0.000	0.000	0.061	43.445	0.000	0.000	0.000	7.453
16.430	0.625	0.000	0.000	0.000	1.920	0.000	0.000	0.000	0.036	0.065	52.949	0.000	0.000	0.000	8.723
0.000	0.305	0.000	0.000	0.000	2.291	0.000	0.000	0.000	0.000	0.063	48.233	0.000	0.000	0.000	8.974
0.196	0.168	0.000	0.000	0.000	1.514	0.000	0.855	0.000	0.058	0.000	37.883	0.000	0.000	0.000	10.341
1.389	0.373	0.000	0.000	0.000	1.008	0.000	0.000	0.000	0.058	0.000	48.129	0.000	0.000	0.018	11.374
0.344	0.206	0.000	0.000	0.000	2.571	0.000	0.000	0.000	0.000	0.000	69.345	0.000	0.000	0.315	11.365
0.000	0.556	0.000	0.000	0.000	2.334	0.000	0.000	0.000	0.032	0.058	78.697	0.000	0.000	0.059	6.441
0.817	0.308	0.000	0.000	0.000	2.804	0.000	0.237	0.000	0.032	0.000	46.410	0.000	0.000	0.015	7.414
0.176	0.331	0.000	0.000	0.000	2.368	0.000	0.000	0.000	0.052	0.000	102.353	0.000	0.000	0.016	7.258
0.716	0.367	0.000	0.000	0.000	3.100	0.000	0.000	0.000	0.000	0.000	64.064	0.000	0.000	0.000	9.280
1.265	0.216	0.000	0.000	0.000	2.215	0.000	0.000	0.000	0.000	0.000	56.394	0.000	0.000	0.014	6.106
2.620	0.249	0.000	0.000	0.000	4.060	0.000	0.000	0.000	0.028	0.000	123.878	0.000	0.000	0.586	9.216
1.517	0.130	0.000	0.000	0.000	1.926	0.000	0.000	0.000	0.000	0.539	19.957	0.000	0.000	0.000	9.836
1.131	0.194	0.000	0.000	0.000	2.166	0.000	0.000	0.000	0.018	0.000	69.461	0.000	0.000	0.000	5.828
0.891	0.457	0.000	0.000	0.000	1.538	0.000	0.000	0.000	0.000	0.000	72.676	0.000	0.000	0.000	12.171
0.708	0.273	0.000	0.000	0.000	4.528	0.000	0.000	0.000	0.052	0.000	47.813	0.000	0.000	0.000	8.119
0.660	0.113	0.000	0.052	0.000	4.214	0.000	0.000	0.000	0.000	0.000	73.775	0.000	0.000	0.000	8.402
1.145	0.131	0.000	0.040	0.000	2.144	0.000	0.000	0.000	0.000	0.068	46.446	0.000	0.000	0.000	12.635
0.606	0.207	0.000	0.000	0.000	1.589	0.000	0.000	0.000	0.000	0.000	68.777	0.000	0.000	0.028	7.696
1.419	0.081	0.000	0.000	0.000	2.171	0.000	0.458	0.000	0.046	0.000	47.578	0.000	0.000	0.000	7.062
0.845	0.434	0.000	0.000	0.000	2.275	0.000	0.000	0.000	0.000	0.000	81.042	0.000	0.000	0.000	9.245
0.377	0.097	0.000	0.000	0.000	3.807	0.000	0.000	0.000	0.000	0.000	58.625	0.000	0.000	0.000	11.621
0.484	0.000	0.000	0.076	0.000	0.825	0.000	0.000	0.000	0.000	0.000	76.557	0.000	0.000	0.022	7.773
0.000	0.114	0.000	0.035	0.000	1.848	0.000	0.000	0.000	0.000	0.000	74.766	0.000	0.000	0.000	9.386
0.000	0.038	0.000	0.000	0.000	1.349	0.000	0.000	0.000	0.000	0.000	126.869	0.000	0.000	0.040	6.027
1.766	0.252	0.000	0.000	0.249	2.067	0.000	0.000	0.000	0.000	0.000	84.915	0.000	0.000	0.000	11.613
0.465	0.080	0.000	0.000	0.000	2.853	0.000	0.000	0.000	0.000	0.083	104.170	0.000	0.000	0.021	9.023
0.169	0.116	0.000	0.018	0.000	2.341	0.000	0.000	0.000	0.000	0.000	87.002	0.000	0.000	0.000	7.678
0.000	0.469	0.000	0.000	0.000	2.812	0.000	0.000	0.000	0.000	0.000	65.325	0.000	0.000	0.063	6.600
0.310	0.319	0.000	0.016	0.000	3.682	0.000	0.000	0.000	0.015	0.000	51.720	0.000	0.000	0.000	5.209
0.000	0.209	0.000	0.000	0.000	2.835	0.000	0.000	0.000	0.000	0.000	77.816	0.000	0.000	0.028	8.149
0.233	0.200	0.000	0.000	0.000	3.031	0.000	0.000	0.000	0.000	0.083	66.444	0.000	0.000	0.000	5.356
2.249	0.096	0.000	0.000	0.000	2.915	0.000	0.000	0.000	0.028	0.100	52.553	0.000	0.000	0.000	7.343
0.000	0.235	0.000	0.000	0.000	1.015	0.000	0.000	0.000	0.019	0.000	75.864	0.000	0.000	0.000	9.033
0.000	0.000	0.000	0.000	0.000	0.723	0.000	0.000	0.000	0.000	0.000	76.903	0.000	0.000	0.000	8.405
0.000	0.169	0.000	0.000	0.000	1.970	0.000	0.000	0.000	0.000	0.000	62.248	0.000	0.000	0.068	6.786
0.741	0.084	0.000	0.000	0.000	4.190	0.000	0.000	0.000	0.000	0.000	56.569	0.000	0.000	0.000	5.765
0.000	0.194	0.000	0.047	0.000	2.448	0.000	0.000	0.000	0.044	0.000	53.738	0.000	0.000	0.021	4.201
0.532	0.152	0.000	0.000	0.000	5.810	0.000	0.000	0.000	0.017	0.000	51.137	0.000	0.000	0.016	5.659
0.211	0.180	0.000	0.066	0.000	2.145	0.000	0.000	0.000	0.062	0.000	42.712	0.000	0.000	0.096	8.352
0.716	0.449	0.000	0.000	0.000	4.801	0.000	0.347	0.000	0.000	0.000	49.515	0.000	0.000	0.000	8.811
1.196	0.205	0.000	0.000	0.000	2.893	0.000	0.290	0.000	0.000	0.000	35.107	0.000	0.000	0.018	6.061
0.000	0.652	0.000	0.000	0.000	6.372	0.000	0.000	0.000	0.041	0.000	78.510	0.164	0.000	0.000	6.105
4.164	0.028	0.000	0.000	0.000	1.850	0.000	0.484	0.000	0.000	0.000	58.371	0.000	0.000	0.000	5.412
0.347	0.089	0.000	0.000	0.000	3.779	0.000	0.000	0.000	0.051	0.185	73.031	0.000	0.000	0.000	16.111
0.476	0.081	0.000	0.000	0.000	4.251	0.000	0.231	0.000	0.047	0.000	48.004	0.000	0.050	0.131	7.262
0.000	0.159	0.000	0.000	0.000	2.276	0.000	0.000	0.000	0.000	0.000	45.340	0.000	0.000	0.000	6.363
2.145	0.169	0.000	0.000	0.000	2.577	0.000	0.000	0.000	0.113	0.000	51.052	0.000	0.000	0.030	9.699
0.511	0.029	0.000	0.000	0.000	2.563	0.000	0.000	0.000	0.017	0.000	52.116	0.000	0.000	0.000	12.486
0.164	0.028	0.000	0.017	0.000	3.837	0.000	0.000	0.000	0.032	0.117	71.220	0.000	0.000	0.000	12.288
3.622	0.059	0.000	0.000	0.000	2.909	0.000	0.000	0.000	0.000	0.061	48.160	0.000	0.000	0.000	11.305
0.998	0.142	0.000	0.017	0.000	1.893	0.000	0.000	0.000	0.049	0.059	25.974	0.000	0.052	0.000	7.437
0.964	0.099	0.000	0.000	0.000	1.657	0.000	0.000	0.000	0.038	0.137	66.052	0.000	0.000	0.000	10.907
1.415	0.104	0.000	0.000	0.000	2.562	0.000	0.294	0.000	0.099	0.000	46.915	0.000	0.000	0.000	10.016
5.768	0.000	0.000	0.000	0.000	2.738	0.000	0.000	0.000	0.000	0.293	48.475	0.000	0.000	0.000	11.072
2.086	0.089	0.000	0.000	0.000	1.413	0.000	0.758	0.000	0.102	0.000	46.941	0.000	0.000	0.000	10.878
1.012	0.173	0.000	0.000	0.000	3.642	0.000	0.294	0.000	0.000	0.072	54.055	0.000	0.000	0.056	7.369
2.219	0.138	0.000	0.021	0.000	2.993	0.000	0.000	0.000	0.000	0.000	42.614	0.000	0.000	0.000	7.550
0.000	0.180	0.000	0.000	0.000	1.517	0.000	0.000	0.000	0.000	0.094	52.030	0.000	0.000	0.241	4.889
0.514	0.205	0.000	0.000	0.000	3.474	0.000	0.000	0.000	0.084	0.061	71.296	0.000	0.000	0.000	8.841
1.103	0.113	0.000	0.000	0.000	4.151	0.000	0.000	0.000	0.000	0.000	31.277	0.000	0.000	0.000	6.917
0.506	0.043	0.000	0.000	0.000	1.692	0.000	0.000	0.000	0.025	0.000	45.530	0.000	0.000	0.000	12.254
1.774	0.228	0.000	0.000	0.000	1.942	0.000	0.000	0.000	0.000	0.000	50.503	0.000	0.000	0.000	7.710
1.015	0.104	0.000	0.021	0.000	4.385	0.000	0.000	0.000	0.020	0.072	52.770	0.000	0.000	0.019	8.455
0.721	0.216	0.000	0.000	0.000	2.085	0.000	0.000	0.000	0.053	0.000	50.753	0.000	0.000	0.016	9.650
0.676	0.000	0.000	0.000	0.000	1.431	0.000	0.000	0.000	0.017	0.000	64.356	0.000	0.053	0.015	8.630
0.636	0.218	0.000	0.000	0.000	2.627	0.000	0.000	0.000	0.000	0.075	114.603	0.000	0.000	0.019	7.311
0.200	0.137	0.000	0.000	0.000	3.036	0.000	0.000	0.000	0.000	0.000	75.006	0.000	0.000	0.365	6.665
0.375	0.096	0.000	0.000	0.000	2.952	0.000	0.273	0.000	0.018	0.000	61.375	0.000	0.000	0.000	7.589
0.775	0.298	0.000	0.000	0.000	1.824	0.000	0.000	0.000	0.019	0.069	40.299	0.000	0.000	0.018	8.425
2.075	0.284	0.000	0.000	0.000	2.538	0.000	0.000	0.000	0.000	0.000	54.369	0.000	0.000	0.000	9.206
0.197	0.236	0.000	0.000	0.000	3.154	0.000	0.000	0.000	0.000	0.070	83.276	0.000	0.000	0.000	10.630
0.628	0.072	0.000	0.000	0.000	3.637	0.000	0.000	0.000	0.020	0.000	75.324	0.000	0.000	0.019	5.975
2.528	0.402	0.000	0.000	0.000	3.240	0.000	0.000	0.000	0.000	0.000	46.162	0.000	0.057	0.017	9.468
1.045	0.215	0.000	0.000	0.000	5.366	0.000	0.000	0.000	0.041	0.000	44.465	0.000	0.000	0.000	12.157
0.907	0.093	0.000	0.000	0.000	3.347	0.000	0.000	0.000	0.000	0.000	71.124	0.000	0.000	0.017	9.310
2.761	0.000	0.000	0.000	0.000	1.942	0.000	0.251	0.000	0.017	0.000	58.706	0.000	0.000	0.000	8.316
1.249	0.134	0.000	0.000	0.000	2.963	0.000	0.000	0.000	0.000	0.000	40.914	0.000	0.000	0.043	7.276
0.470	0.161	0.000	0.000	0.000	2.198	0.000	0.000	0.000	0.023	0.000	67.179	0.000	0.074	0.021	9.081
0.000	0.529	0.000	0.000	0.000	1.137	0.000	0.000	0.000	0.020	0.073	62.924	0.000	0.000	0.000	5.822
0.818	0.140	0.000	0.000	0.000	5.638	0.000	0.000	0.000	0.000	0.000	60.760	0.000	0.000	0.112	8.910
1.535	0.131	0.000	0.000	0.000	2.124	0.000	0.000	0.000	0.000	0.068	51.753	0.000	0.000	0.351	9.777
0.000	0.376	0.000	0.000	0.000	2.545	0.000	0.000	0.000	0.054	0.000	44.834	0.000	0.000	0.033	8.440
1.193	0.306	0.000	0.000	0.000	1.010	0.000	0.000	0.000	0.000	0.000	85.209	0.000	0.000	0.000	8.157
0.631	0.216	0.000	0.000	0.000	3.151	0.000	0.229	0.000	0.108	0.000	61.651	0.000	0.000	0.490	7.557
0.439	0.125	0.116	0.000	0.000	5.761	0.000	0.000	0.000	0.014	0.000	72.340	0.000	0.000	0.000	9.096
0.950	0.217	0.000	0.000	0.000	2.151	0.000	0.000	0.000	0.062	0.000	52.970	0.000	0.000	0.043	10.366
1.205	0.284	0.000	0.000	0.000	1.995	0.000	0.219	0.000	0.000	0.053	62.938	0.000	0.000	0.000	7.244
0.000	0.244	0.000	0.000	0.000	2.610	0.000	0.000	0.000	0.046	0.000	80.471	0.000	0.000	0.000	6.536
0.686	0.440	0.000	0.036	0.000	2.876	0.000	0.000	0.000	0.034	0.000	59.460	0.000	0.000	0.000	3.762
1.101	0.188	0.000	0.000	0.000	1.458	0.000	0.640	0.000	0.000	0.078	63.326	0.000	0.000	0.000	7.850
0.673	0.230	0.000	0.000	0.000	1.552	0.000	0.000	0.000	0.000	0.000	81.698	0.000	0.000	0.000	8.541
0.498	0.170	0.000	0.000	0.000	2.329	0.000	0.362	0.000	0.024	0.088	64.659	0.000	0.000	0.000	6.951
0.280	0.526	0.000	0.000	0.236	4.314	0.000	0.000	0.000	0.027	0.000	54.218	0.000	0.000	0.026	7.631
2.498	0.117	0.000	0.000	0.000	2.081	0.000	0.000	0.074	0.000	0.000	54.162	0.000	0.000	0.000	10.880
1.899	0.027	0.000	0.000	0.000	2.243	0.000	0.000	0.000	0.046	0.000	66.943	0.000	0.000	0.014	9.326
0.974	0.111	0.000	0.000	0.000	3.602	0.000	0.000	0.000	0.000	0.000	44.077	0.000	0.000	0.000	7.488
0.579	0.198	0.000	0.000	0.000	2.660	0.000	0.421	0.000	0.000	0.000	69.089	0.000	0.000	0.053	7.038
0.151	0.284	0.000	0.000	0.000	1.610	0.000	0.000	0.000	0.074	0.000	118.136	0.000	0.000	0.028	7.314
2.044	0.140	0.000	0.000	0.000	3.036	0.000	0.297	0.000	0.020	0.000	52.080	0.000	0.000	0.000	8.459
2.388	0.063	0.000	0.000	0.000	1.796	0.000	0.267	0.000	0.090	0.000	57.601	0.000	0.000	0.000	9.364
0.902	0.093	0.000	0.000	0.000	1.988	0.000	0.000	0.000	0.018	0.000	74.115	0.000	0.000	0.000	14.644

ENSG00000206917	ENSG00000241102	ENSG00000139926	ENSG00000227161	ENSG00000281460	ENSG00000111405	ENSG00000168594	ENSG00000196267	ENSG00000164111	ENSG00000225484	ENSG00000227518	ENSG00000171202	ENSG00000245112	ENSG00000274124	ENSG00000166013	ENSG00000240089
0.000	0.000	2.003	0.000	0.535	0.185	0.050	0.904	114.921	0.600	0.000	15.676	0.210	0.238	0.000	0.000
0.000	0.059	3.906	0.000	0.000	0.102	0.000	1.071	273.217	0.595	0.000	24.835	0.045	0.135	0.000	0.000
0.000	0.000	4.058	0.000	0.000	0.084	0.013	1.854	97.595	0.435	0.000	39.235	0.079	0.120	0.000	0.000
0.000	0.000	3.684	0.437	0.000	0.325	0.000	2.340	123.587	0.210	0.015	22.682	0.045	0.544	0.000	0.000
0.000	0.000	2.198	0.487	0.000	0.493	0.000	1.162	284.612	0.384	0.000	42.365	0.050	0.303	0.000	0.025
0.000	0.000	1.872	0.577	0.034	0.244	0.006	2.184	59.276	0.453	0.019	17.980	0.118	0.538	0.000	0.000
0.000	0.000	3.854	0.650	0.000	0.102	0.000	1.554	303.654	0.518	0.000	19.833	0.000	0.067	0.000	0.000
0.000	0.000	2.237	0.229	0.000	1.426	0.015	0.796	153.322	0.492	0.000	24.915	0.094	0.000	0.000	0.000
0.000	0.000	1.516	0.265	0.000	0.853	0.004	1.155	188.117	0.398	0.000	37.355	0.000	0.165	0.000	0.000
0.000	0.000	2.309	0.000	0.000	0.363	0.025	1.092	211.751	0.522	0.000	32.859	0.079	0.239	0.000	0.000
0.000	0.000	1.804	0.000	0.000	1.610	0.009	0.895	173.845	0.477	0.013	30.838	0.019	0.471	0.000	0.000
0.000	0.000	2.220	0.196	0.000	0.248	0.006	1.379	246.138	0.452	0.000	17.634	0.161	0.183	0.000	0.000
0.000	0.000	5.390	0.202	0.035	1.501	0.000	2.209	292.325	0.342	0.000	15.611	0.145	0.314	0.000	0.000
0.000	0.051	2.711	0.189	0.000	0.602	0.000	2.411	245.544	0.275	0.025	15.808	0.155	0.470	0.000	0.000
0.000	0.000	3.376	0.000	0.147	0.580	0.000	1.098	184.944	0.260	0.000	22.220	0.086	0.000	0.000	0.000
0.000	0.000	3.032	0.379	0.000	0.233	0.003	1.501	139.795	0.518	0.006	13.159	0.058	0.118	0.000	0.000
0.000	0.000	3.469	0.000	0.000	0.379	0.060	1.920	186.692	0.393	0.007	16.809	0.134	0.339	0.000	0.000
0.000	0.000	2.036	0.259	0.000	0.225	0.063	0.989	168.976	0.744	0.034	43.688	0.000	0.242	0.000	0.000
0.000	0.000	4.060	0.000	0.000	0.314	0.000	1.320	386.140	0.376	0.000	17.568	0.061	0.306	0.000	0.000
0.000	0.000	3.565	0.000	0.000	0.527	0.000	1.122	250.069	0.331	0.000	18.212	0.087	0.393	0.000	0.000
0.000	0.000	1.491	0.000	0.000	0.976	0.004	1.155	199.159	0.390	0.015	75.843	0.092	0.349	0.000	0.000
0.000	0.000	4.235	0.929	0.284	0.505	0.023	1.709	53.703	0.617	0.031	15.244	0.215	0.505	0.000	0.000
0.000	0.000	4.390	0.227	0.000	0.411	0.140	1.369	127.712	0.485	0.000	33.575	0.070	0.212	0.000	0.000
0.000	0.000	3.508	0.000	0.000	0.105	0.003	0.719	285.151	0.357	0.006	24.012	0.040	0.000	0.000	0.000
0.000	0.000	2.575	0.645	0.000	0.257	0.004	1.513	215.154	0.589	0.007	21.065	0.022	0.334	0.000	0.000
0.000	0.000	1.580	0.224	0.000	0.332	0.004	1.369	96.039	0.447	0.015	19.068	0.046	0.069	0.000	0.000
0.000	0.000	1.823	0.253	0.177	0.247	0.008	1.063	192.724	0.369	0.008	21.868	0.026	0.393	0.000	0.000
0.000	0.000	3.054	0.000	0.000	0.371	0.036	0.977	173.212	0.758	0.000	20.409	0.137	0.415	0.000	0.046
0.000	0.000	1.930	0.222	1.279	1.469	0.000	1.738	391.197	0.415	0.015	24.093	0.046	0.896	0.000	0.000
0.000	0.000	1.399	0.446	0.078	0.646	0.000	2.079	288.109	0.353	0.015	28.641	0.092	0.208	0.000	0.000
0.000	0.000	0.744	0.183	0.032	0.046	0.000	0.801	219.092	0.473	0.006	40.890	0.094	0.057	0.000	0.000
0.000	0.000	2.215	0.384	0.000	0.230	0.000	0.861	192.459	0.244	0.019	16.760	0.079	0.836	0.033	0.000
0.000	0.000	0.845	0.224	0.078	1.449	0.004	1.032	184.253	0.451	0.000	28.860	0.046	0.139	0.000	0.000
0.000	0.000	1.199	0.216	0.000	0.390	0.000	1.153	147.766	0.408	0.021	32.400	0.000	0.402	0.000	0.000
0.000	0.000	1.730	0.000	0.000	0.511	0.000	1.227	218.338	0.359	0.030	24.143	0.047	0.283	0.000	0.000
0.000	0.000	2.627	0.206	0.000	0.664	0.003	1.159	198.571	0.431	0.007	23.416	0.000	0.192	0.000	0.000
0.000	0.000	5.949	0.197	0.344	2.203	0.003	1.233	190.192	0.294	0.013	10.759	0.202	0.735	0.011	0.000
0.000	0.000	5.970	0.590	0.034	0.413	0.000	1.721	126.436	0.516	0.013	20.705	0.081	0.428	0.000	0.000
0.000	0.000	5.307	0.000	0.041	0.357	0.149	1.507	157.652	0.387	0.016	29.809	0.217	0.365	0.000	0.000
0.000	0.000	4.487	0.485	0.000	0.448	0.004	1.972	355.556	0.354	0.016	21.984	0.124	0.603	0.000	0.025
0.000	0.000	1.232	0.482	0.000	0.952	0.000	0.573	211.954	0.411	0.000	24.067	0.074	0.300	0.000	0.000
0.000	0.000	3.506	0.408	0.000	0.096	0.010	0.536	327.061	0.390	0.020	20.218	0.105	0.317	0.000	0.000
0.000	0.000	3.819	0.224	0.000	0.219	0.004	1.456	254.136	0.420	0.015	27.290	0.092	0.349	0.000	0.000
0.000	0.000	5.154	0.385	0.000	0.335	0.000	1.430	288.294	0.561	0.026	17.884	0.079	0.598	0.000	0.000
0.000	0.000	4.302	0.205	0.036	0.275	0.003	2.105	268.249	0.379	0.007	19.550	0.021	1.467	0.000	0.000
0.000	0.000	4.207	0.200	0.000	0.681	0.007	1.280	210.579	0.335	0.013	19.687	0.062	0.560	0.000	0.000
0.000	0.000	15.652	0.661	0.000	0.965	0.000	0.811	68.031	0.459	0.029	25.773	0.000	0.822	0.000	0.023
0.000	0.000	5.347	0.223	0.234	0.291	0.087	1.361	122.946	0.701	0.000	17.953	0.137	0.485	0.000	0.000
0.000	0.000	1.675	0.193	0.000	0.336	0.000	1.883	203.272	0.355	0.006	23.429	0.040	0.000	0.000	0.000
0.000	0.000	1.890	0.000	0.000	0.245	0.000	0.680	230.105	0.524	0.012	25.315	0.094	0.114	0.000	0.000
0.000	0.000	0.963	0.184	0.000	0.758	0.063	0.982	102.656	0.578	0.030	47.523	0.132	0.057	0.000	0.000
0.000	0.000	2.154	0.197	0.069	0.365	0.000	0.961	157.791	0.467	0.000	25.638	0.142	0.000	0.000	0.000
0.000	0.000	4.916	0.402	0.000	0.408	0.026	1.240	187.687	0.324	0.007	36.447	0.124	0.437	0.000	0.000
0.000	0.000	1.702	0.888	0.000	0.360	0.000	2.233	451.250	0.377	0.006	23.587	0.073	0.221	0.000	0.000
0.000	0.000	1.557	0.000	0.000	0.652	0.741	1.593	124.082	0.658	0.011	48.995	0.302	0.102	0.000	0.000
0.000	0.000	1.879	0.000	0.745	0.339	0.028	1.558	123.266	0.482	0.028	31.841	0.000	0.265	0.000	0.000
0.000	0.000	2.974	0.212	0.000	0.591	0.003	1.322	293.756	0.384	0.014	27.911	0.087	0.329	0.000	0.000
0.000	0.000	2.765	0.200	0.000	0.261	0.013	1.423	428.883	0.382	0.007	18.975	0.144	0.311	0.000	0.000
0.000	0.000	2.768	0.000	0.000	0.223	0.000	1.395	211.796	0.705	0.000	18.615	0.020	0.000	0.000	0.000
0.000	0.000	2.884	0.000	0.000	0.578	0.000	1.035	78.169	0.520	0.000	29.637	0.038	0.173	0.000	0.000
0.000	0.000	1.911	0.214	0.075	0.652	0.003	1.485	214.794	0.407	0.007	21.315	0.154	0.267	0.000	0.000
0.000	0.000	1.942	0.340	0.000	0.148	0.006	1.454	208.162	0.394	0.011	35.517	0.244	0.423	0.000	0.000
0.000	0.000	6.573	0.177	0.031	0.629	0.095	1.498	104.940	0.633	0.024	17.712	0.164	0.716	0.000	0.000
0.000	0.000	2.506	0.190	0.033	0.083	0.000	1.185	184.309	0.448	0.025	15.767	0.039	0.177	0.000	0.000
0.000	0.000	0.946	0.000	0.000	2.315	0.017	1.452	263.107	0.283	0.000	33.245	0.065	0.000	0.000	0.000
0.000	0.000	1.877	0.272	0.000	0.561	0.009	2.741	107.463	0.309	0.000	21.518	0.084	0.761	0.000	0.000
0.000	0.000	2.606	0.000	0.000	0.081	0.000	1.833	435.012	0.446	0.012	23.142	0.153	0.348	0.000	0.000
0.000	0.000	1.071	0.000	0.000	0.315	0.000	1.592	664.767	0.353	0.000	35.430	0.077	0.077	0.000	0.000
0.000	0.000	2.851	0.000	0.000	0.227	0.011	1.852	164.338	0.267	0.022	17.177	0.000	0.411	0.000	0.000
0.000	0.000	1.181	0.000	0.000	0.189	0.004	0.704	255.020	0.292	0.000	41.096	0.000	0.000	0.000	0.000
0.000	0.000	2.132	0.000	0.000	0.165	0.003	1.231	354.887	0.290	0.006	25.122	0.059	0.059	0.000	0.000
0.000	0.000	1.776	0.385	0.000	2.466	0.000	0.844	212.897	0.326	0.000	50.396	0.000	0.239	0.000	0.000
0.000	0.000	2.747	0.174	0.000	0.297	0.017	0.658	317.879	0.401	0.000	23.384	0.054	0.163	0.000	0.000
0.000	0.000	1.597	0.000	0.060	0.174	0.000	1.524	414.592	0.484	0.011	33.856	0.035	0.214	0.000	0.000
0.000	0.000	2.695	0.000	0.000	0.104	0.000	1.894	186.110	0.076	0.000	14.772	0.108	0.000	0.000	0.000
0.000	0.000	4.986	0.316	0.055	2.860	0.000	1.755	359.327	0.239	0.000	27.773	0.000	0.295	0.000	0.000
0.000	0.000	2.955	0.221	0.039	0.504	0.011	1.443	333.120	0.308	0.000	38.538	0.023	0.343	0.000	0.000
0.000	0.000	4.789	0.000	0.000	0.096	0.022	1.967	59.997	0.357	0.000	17.993	0.000	0.823	0.000	0.000
0.000	0.000	2.978	0.000	0.000	0.947	0.000	1.113	366.799	0.252	0.009	59.334	0.057	0.778	0.000	0.000
0.000	0.000	3.106	0.000	0.000	0.753	0.000	1.433	159.749	0.197	0.000	36.054	0.114	0.690	0.000	0.000
0.000	0.000	2.065	0.000	0.000	2.063	0.008	1.343	280.501	0.306	0.008	61.753	0.052	0.079	0.000	0.000
0.000	0.000	1.040	0.000	0.000	0.852	0.003	1.263	220.569	0.346	0.000	34.968	0.000	0.186	0.000	0.000
0.000	0.000	5.931	0.000	0.124	0.480	0.112	1.608	119.286	0.462	0.000	20.372	0.049	0.368	0.000	0.000
0.000	0.000	2.425	0.536	0.000	1.165	0.000	1.710	131.658	0.308	0.000	17.756	0.028	0.250	0.000	0.000
0.000	0.000	3.350	0.224	0.000	0.300	0.033	1.356	109.201	0.286	0.007	14.006	0.230	0.139	0.000	0.000
0.000	0.000	3.596	0.000	0.000	0.034	0.004	1.278	267.765	0.488	0.000	21.442	0.000	0.000	0.000	0.000
0.000	0.000	1.477	0.187	0.000	0.163	0.375	0.898	71.463	0.846	0.019	10.170	0.307	0.756	0.000	0.019
0.000	0.000	3.063	0.195	0.000	0.198	0.000	1.240	141.530	0.463	0.000	20.144	0.080	0.303	0.000	0.000
0.000	0.000	2.829	0.000	0.000	0.497	0.000	1.121	454.764	0.335	0.006	15.746	0.055	0.333	0.000	0.000
0.000	0.000	2.723	0.347	0.000	1.346	0.006	0.913	337.148	0.251	0.000	17.209	0.107	0.432	0.000	0.000
0.000	0.000	3.391	0.742	0.000	0.316	0.000	1.619	167.345	0.544	0.049	21.617	0.057	0.461	0.000	0.000
0.000	0.000	0.899	0.191	0.134	0.721	0.000	1.295	268.149	0.267	0.006	23.102	0.079	0.179	0.000	0.000
0.000	0.000	0.660	0.000	0.000	0.234	0.045	0.659	124.631	0.358	0.000	25.116	0.076	0.230	0.000	0.000
0.000	0.000	3.075	0.194	0.102	0.084	0.006	1.661	123.760	0.329	0.006	15.702	0.139	0.241	0.000	0.000
0.000	0.000	1.503	0.374	4.474	1.170	0.067	1.085	81.036	0.554	0.006	23.891	0.096	0.232	0.000	0.000
0.000	0.000	3.041	0.650	0.000	0.345	0.004	1.263	360.822	0.276	0.029	23.233	0.044	0.202	0.000	0.000
0.000	0.000	2.459	0.000	0.119	0.214	0.118	1.111	171.342	0.443	0.008	34.777	0.117	0.141	0.013	0.000
0.000	0.000	2.738	0.000	0.000	0.268	0.030	1.397	132.721	0.341	0.031	25.161	0.000	0.576	0.000	0.000
0.000	0.000	2.977	0.000	0.000	0.340	0.019	1.045	197.700	0.439	0.039	24.358	0.060	0.364	0.000	0.061
0.000	0.000	1.392	0.455	0.000	0.593	0.004	1.014	345.907	0.304	0.015	20.729	0.117	0.141	0.000	0.000
0.000	0.000	3.599	0.453	0.040	0.353	0.085	1.207	170.780	0.241	0.015	14.767	0.070	0.070	0.013	0.000
0.000	0.000	3.916	0.296	0.000	0.622	0.000	1.002	517.079	0.448	0.020	79.181	0.000	0.184	0.000	0.000
0.000	0.000	3.369	0.000	0.000	0.578	0.000	1.183	157.126	0.353	0.026	21.657	0.000	0.060	0.000	0.000
0.000	0.000	2.143	0.000	0.043	0.449	0.020	1.167	171.765	0.054	0.000	23.210	0.127	0.231	0.000	0.000
0.000	0.000	5.240	0.853	0.000	1.153	0.000	1.358	305.397	0.419	0.009	27.353	0.029	0.354	0.000	0.000
0.000	0.000	2.264	0.498	0.000	0.271	0.000	0.672	260.222	0.390	0.017	19.362	0.000	0.000	0.000	0.052
0.000	0.000	1.999	0.000	0.040	0.273	0.134	0.751	149.003	0.229	0.023	33.151	0.000	0.213	0.000	0.000
0.000	0.000	2.229	0.000	0.106	0.191	0.020	0.569	169.393	0.558	0.007	54.156	0.042	0.189	0.023	0.000
0.000	0.000	2.488	0.000	0.000	0.041	0.000	1.381	299.208	0.411	0.006	27.811	0.098	0.236	0.000	0.000
0.000	0.000	2.504	0.000	0.000	0.164	0.004	1.366	279.986	0.421	0.008	43.241	0.000	0.074	0.000	0.000
0.000	0.000	1.006	0.000	0.000	0.699	0.011	0.836	427.769	0.295	0.000	43.577	0.000	0.000	0.000	0.000
0.000	0.000	2.270	0.000	0.000	0.381	0.003	1.825	415.171	0.422	0.000	23.259	0.065	0.197	0.000	0.000
0.000	0.000	2.909	0.218	0.000	1.308	0.018	0.955	131.742	0.342	0.000	22.052	0.067	0.203	0.000	0.000
0.000	0.000	4.367	0.000	0.000	0.490	0.030	1.386	192.654	0.254	0.046	17.376	0.000	0.580	0.000	0.000
0.000	0.000	1.308	0.665	0.000	0.112	0.000	1.225	216.921	0.422	0.007	18.389	0.023	0.275	0.000	0.000
0.000	0.000	1.992	0.000	0.000	0.264	0.004	0.618	198.209	0.320	0.008	18.846	0.048	0.293	0.000	0.000
0.000	0.000	1.633	0.406	0.071	0.279	0.003	1.136	356.883	0.308	0.007	19.622	0.021	0.315	0.000	0.000
0.000	0.000	2.861	0.470	0.000	1.021	0.008	1.299	200.283	0.369	0.023	19.620	0.024	0.584	0.000	0.000
0.000	0.000	2.539	0.204	0.000	0.118	0.000	2.097	534.264	0.381	0.007	28.449	0.105	0.063	0.000	0.000
0.000	0.000	4.830	0.194	0.000	0.133	0.000	1.697	443.144	0.361	0.006	18.188	0.119	0.000	0.000	0.000
0.104	0.000	3.655	0.000	0.061	0.140	0.206	0.994	165.640	0.457	0.006	21.009	0.072	0.218	0.000	0.000
0.000	0.000	1.787	0.000	0.092	0.402	0.030	1.093	219.266	0.410	0.026	25.907	0.163	0.082	0.000	0.000
0.000	0.000	8.026	0.231	0.000	0.126	0.008	0.894	142.159	0.459	0.015	10.771	0.071	0.000	0.000	0.000
0.000	0.000	1.389	0.000	0.080	1.764	0.000	1.360	252.920	0.415	0.008	37.503	0.071	0.286	0.000	0.000
0.128	0.000	2.799	0.000	0.000	1.109	0.007	0.823	469.776	0.306	0.007	16.740	0.044	0.268	0.000	0.000
0.000	0.000	1.530	0.000	0.000	0.633	0.007	0.865	129.003	0.584	0.007	28.723	0.042	0.575	0.000	0.021
0.000	0.000	2.742	0.000	0.000	0.753	0.000	1.670	297.925	0.282	0.045	26.591	0.000	0.208	0.000	0.000
0.000	0.000	2.206	0.177	0.000	0.578	0.000	1.633	259.421	0.260	0.029	20.240	0.164	0.661	0.000	0.000
0.000	0.000	3.497	0.000	0.000	0.077	0.000	1.116	253.905	0.525	0.000	25.604	0.068	0.204	0.000	0.000
0.000	0.000	2.333	0.356	0.031	0.425	0.049	2.277	162.908	0.340	0.030	29.430	0.219	0.608	0.000	0.000
0.000	0.000	3.252	0.339	0.000	0.509	0.000	1.737	352.305	0.368	0.028	17.332	0.069	0.368	0.000	0.000
0.000	0.000	2.856	0.000	0.000	0.521	0.000	0.853	205.032	0.322	0.000	25.775	0.109	0.000	0.000	0.000
0.000	0.000	3.718	0.000	0.000	0.460	0.000	1.246	243.454	0.375	0.000	35.843	0.099	0.000	0.000	0.000
0.000	0.000	1.468	0.495	0.000	0.188	0.020	1.023	84.266	0.437	0.016	28.742	0.152	0.308	0.000	0.000
0.000	0.000	2.988	0.756	0.044	0.967	0.000	1.676	207.050	0.364	0.017	27.316	0.000	0.470	0.000	0.000
0.000	0.000	4.243	0.280	0.000	0.304	0.014	1.740	171.940	0.451	0.009	16.380	0.086	0.261	0.000	0.000
0.000	0.000	2.927	0.000	0.000	1.479	0.005	0.912	173.222	0.417	0.010	17.079	0.097	0.684	0.000	0.000
0.000	0.000	5.221	0.000	0.045	0.157	0.017	1.062	108.190	0.418	0.017	22.868	0.105	0.317	0.000	0.000
0.000	0.000	2.839	0.356	0.000	0.438	0.000	0.699	287.490	0.451	0.006	34.229	0.091	0.332	0.000	0.000
0.000	0.000	3.661	0.000	0.000	1.281	0.003	0.883	232.387	0.360	0.000	24.225	0.075	0.170	0.010	0.000
0.000	0.000	2.786	0.976	0.000	0.365	0.000	0.848	251.873	0.507	0.011	22.365	0.100	0.405	0.000	0.000
0.000	0.000	2.823	0.170	0.000	0.227	0.003	0.775	199.740	0.498	0.000	23.582	0.017	0.053	0.000	0.000
0.000	0.000	1.718	0.000	0.161	0.449	0.000	0.630	151.982	0.290	0.023	19.663	0.212	0.428	0.000	0.000
0.000	0.000	2.335	0.000	0.036	0.964	0.003	1.510	307.986	0.432	0.034	20.888	0.169	0.449	0.000	0.000
0.000	0.000	1.929	0.000	0.000	0.330	0.000	0.885	478.844	0.394	0.000	19.978	0.083	0.126	0.000	0.000

ENSG00000277754	ENSG00000234455	ENSG00000128534	ENSG00000275297	ENSG00000225325	ENSG00000218089	ENSG00000010704	ENSG00000228360	ENSG00000200388	ENSG00000276592	ENSG00000268717	ENSG00000213778	ENSG00000233425	ENSG00000130300	ENSG00000186976	ENSG00000201843
0.229	0.000	2.276	0.040	0.000	0.000	0.580	0.900	0.221	0.000	0.000	0.000	0.000	43.164	0.315	0.000
0.000	0.026	4.124	0.011	0.000	0.000	2.566	1.224	0.000	0.000	0.000	0.066	0.000	11.293	0.263	0.000
0.000	0.000	4.217	0.020	0.000	0.016	0.449	1.567	0.000	0.024	0.000	0.156	0.000	30.740	0.101	0.000
0.000	0.000	3.885	0.069	0.000	0.000	1.339	0.926	0.379	0.000	0.000	0.044	0.000	5.772	0.357	0.000
0.000	0.029	3.337	0.025	0.058	0.000	3.136	0.687	0.000	0.000	0.000	0.000	0.000	8.191	0.243	0.000
0.000	0.000	5.103	0.151	0.000	0.000	0.759	1.153	0.000	0.000	0.000	0.097	0.000	23.442	0.196	0.000
0.000	0.000	5.042	0.011	0.052	0.000	3.112	1.298	0.000	0.000	0.000	0.087	0.000	17.120	0.486	0.000
0.103	0.027	1.835	0.000	0.000	0.000	3.002	1.050	0.000	0.000	0.078	0.000	0.000	15.430	0.221	0.000
0.000	0.221	3.679	0.166	0.000	0.000	1.751	2.987	0.000	0.000	0.000	0.053	0.000	30.704	0.246	0.000
0.000	0.000	4.091	0.000	0.092	0.000	6.083	1.902	0.000	0.000	0.000	0.388	0.000	36.048	0.122	0.000
0.000	0.000	4.331	0.020	0.045	0.000	3.371	1.001	0.164	0.000	0.000	0.019	0.000	11.055	0.142	0.000
0.000	0.047	3.939	0.041	0.000	0.000	1.735	0.760	0.170	0.000	0.000	0.099	0.000	33.985	0.402	0.000
0.000	0.290	2.867	0.021	0.000	0.000	1.396	1.355	0.000	0.000	0.000	0.020	0.000	14.981	0.234	0.000
0.000	0.000	2.814	0.030	0.068	0.000	1.108	1.000	0.164	0.000	0.000	0.076	0.000	37.299	0.191	0.000
0.000	0.000	2.987	0.000	0.000	0.000	1.670	2.080	0.730	0.000	0.000	0.170	0.000	23.365	0.124	0.000
0.000	0.000	2.696	0.010	0.000	0.000	4.610	0.734	0.000	0.000	0.000	0.019	0.000	14.184	0.268	0.000
0.000	0.000	3.317	0.068	0.000	0.000	0.616	1.001	0.000	0.000	0.000	0.000	0.000	26.919	0.129	0.000
0.000	0.000	2.460	0.163	0.000	0.000	1.599	1.371	0.224	0.000	0.000	0.026	0.000	6.651	0.085	0.000
0.000	0.094	3.714	0.000	0.024	0.016	2.093	1.321	0.000	0.000	0.000	0.040	0.000	11.955	0.217	0.000
0.000	0.428	2.413	0.022	0.025	0.000	2.259	0.818	0.000	0.000	0.000	0.021	0.000	10.785	0.225	0.000
0.000	0.027	2.390	0.059	0.000	0.000	3.061	2.378	0.389	0.000	0.000	0.000	0.000	10.439	0.110	0.000
0.209	0.000	4.415	0.109	0.000	0.000	0.516	0.983	0.000	0.000	0.000	0.094	0.018	5.669	0.158	0.000
0.000	0.027	4.186	0.012	0.000	0.000	1.307	1.601	0.197	0.000	0.000	0.069	0.000	9.092	0.136	0.000
0.000	0.000	3.960	0.010	0.000	0.000	4.861	0.953	0.334	0.000	0.000	0.039	0.000	32.394	0.302	0.000
0.000	0.000	3.603	0.045	0.026	0.018	2.061	0.758	0.000	0.000	0.000	0.043	0.000	59.005	0.175	0.000
0.000	0.000	4.092	0.058	0.000	0.000	2.346	1.261	0.194	0.000	0.000	0.023	0.000	44.254	0.585	0.000
0.000	0.242	3.455	0.026	0.000	0.000	3.318	2.764	0.000	0.000	0.000	0.025	0.000	12.042	0.587	0.000
0.000	0.000	5.305	0.023	0.000	0.000	1.194	1.413	0.000	0.000	0.000	0.090	0.000	42.501	0.161	0.000
0.000	0.053	4.110	0.093	0.027	0.000	1.565	1.877	0.192	0.000	0.000	0.045	0.000	9.301	0.601	0.000
0.000	0.080	4.005	0.175	0.000	0.000	0.896	1.337	0.000	0.000	0.000	0.045	0.000	14.955	0.142	0.000
0.000	0.088	4.162	0.058	0.000	0.000	2.727	2.264	0.159	0.000	0.000	0.000	0.000	26.633	0.222	0.000
0.000	0.000	3.620	0.010	0.069	0.000	1.376	0.949	0.166	0.024	0.000	0.039	0.000	12.333	0.248	0.000
0.100	0.374	2.501	0.047	0.000	0.000	1.884	1.656	0.000	0.000	0.000	0.023	0.000	9.720	0.395	0.000
0.000	0.026	4.457	0.011	0.000	0.000	2.910	1.520	0.000	0.000	0.000	0.000	0.000	61.922	0.584	0.000
0.000	0.000	3.917	0.024	0.000	0.000	4.185	1.124	0.000	0.000	0.000	0.138	0.000	20.953	0.320	0.000
0.000	0.000	3.317	0.000	0.000	0.000	3.397	0.873	0.536	0.000	0.000	0.000	0.000	9.057	0.292	0.000
0.088	0.000	5.797	0.041	0.000	0.000	3.096	2.014	0.000	0.000	0.000	0.040	0.000	5.610	0.558	0.000
0.000	0.000	3.542	0.000	0.000	0.000	2.998	0.486	0.511	0.024	0.000	0.099	0.000	16.228	0.207	0.000
0.211	0.028	3.965	0.012	0.000	0.020	1.652	1.655	0.000	0.029	0.000	0.095	0.000	33.723	0.154	0.000
0.544	0.116	4.475	0.000	0.029	0.000	0.771	0.427	0.000	0.000	0.000	0.000	0.000	13.208	0.080	0.000
0.000	0.000	4.215	0.000	0.000	0.000	2.927	0.425	0.000	0.000	0.000	0.049	0.000	5.450	0.454	0.000
0.092	0.000	4.432	0.053	0.024	0.000	3.867	0.647	0.000	0.000	0.000	0.041	0.000	29.471	0.300	0.000
0.000	0.000	3.728	0.035	0.000	0.000	2.197	1.266	0.388	0.000	0.000	0.000	0.000	12.846	0.277	0.000
0.000	0.069	4.408	0.020	0.000	0.000	2.724	0.475	0.167	0.000	0.000	0.019	0.015	6.975	0.332	0.000
0.092	0.025	3.130	0.021	0.000	0.000	2.125	0.651	0.355	0.025	0.000	0.000	0.000	19.544	0.635	0.000
0.000	0.000	3.925	0.052	0.000	0.000	3.974	0.776	0.000	0.000	0.000	0.020	0.000	30.883	0.249	0.000
0.000	0.000	5.107	0.000	0.000	0.000	0.621	1.399	0.191	0.000	0.000	0.044	0.000	22.280	0.080	0.000
0.000	0.000	3.424	0.023	0.000	0.000	0.831	1.022	0.000	0.028	0.000	0.090	0.017	27.399	0.111	0.000
0.087	0.000	3.419	0.061	0.000	0.000	2.294	1.228	0.000	0.000	0.000	0.078	0.000	18.649	0.217	0.000
0.000	0.000	3.006	0.000	0.000	0.000	4.381	0.966	0.000	0.000	0.000	0.092	0.000	18.078	0.122	0.000
0.000	0.022	3.224	0.038	0.000	0.015	1.519	2.267	0.000	0.000	0.000	0.000	0.000	26.873	0.202	0.000
0.000	0.047	3.932	0.000	0.000	0.000	5.281	1.184	0.000	0.000	0.000	0.099	0.000	31.628	0.199	0.000
0.000	0.000	4.848	0.032	0.000	0.000	3.560	1.631	0.174	0.000	0.000	0.121	0.000	57.498	0.242	0.000
0.000	0.042	3.119	0.000	0.021	0.000	2.044	0.940	0.154	0.000	0.000	0.054	0.000	9.533	0.340	0.000
0.000	0.000	4.400	0.068	0.000	0.000	1.655	1.269	0.283	0.000	0.000	0.231	0.000	20.378	0.263	0.000
0.000	0.000	6.337	0.045	0.000	0.000	0.393	1.503	0.000	0.000	0.000	0.086	0.000	6.133	0.039	0.000
0.000	0.076	5.436	0.022	0.000	0.000	3.248	0.822	0.000	0.000	0.000	0.000	0.000	6.775	0.098	0.000
0.000	0.143	3.517	0.042	0.000	0.000	1.865	1.412	0.173	0.000	0.000	0.081	0.000	12.752	0.177	0.000
0.000	0.000	5.585	0.073	0.000	0.000	3.399	0.842	0.000	0.000	0.000	0.100	0.015	56.642	0.735	0.000
0.000	0.465	4.046	0.010	0.000	0.000	4.681	0.851	0.000	0.000	0.000	0.056	0.000	4.357	1.056	0.000
0.193	0.026	3.963	0.045	0.026	0.000	1.224	0.756	0.186	0.000	0.000	0.043	0.000	6.976	0.173	0.000
0.000	0.000	3.263	0.107	0.041	0.000	1.076	1.320	0.000	0.000	0.000	0.034	0.000	7.030	0.174	0.000
0.000	0.042	3.071	0.074	0.000	0.000	0.581	1.312	0.000	0.000	0.000	0.036	0.014	2.840	0.173	0.183
0.000	0.113	4.008	0.020	0.000	0.016	3.009	1.541	0.329	0.000	0.000	0.096	0.015	25.817	0.252	0.000
0.000	0.000	4.788	0.033	0.000	0.000	3.014	1.195	0.000	0.000	0.000	0.107	0.032	37.720	0.202	0.000
0.000	0.747	2.201	0.000	0.000	0.000	0.739	1.439	0.000	0.000	0.000	0.082	0.000	28.578	0.086	0.000
0.000	0.089	3.176	0.117	0.000	0.000	0.580	1.974	0.000	0.000	0.000	0.150	0.029	15.966	0.161	0.000
0.000	1.040	1.589	0.013	0.000	0.000	2.466	0.614	0.000	0.000	0.000	0.050	0.000	4.626	0.079	0.000
0.000	0.198	3.275	0.035	0.079	0.000	3.623	0.816	0.000	0.000	0.000	0.100	0.000	2.129	0.154	0.000
0.000	0.718	2.995	0.027	0.000	0.000	2.839	2.211	0.000	0.000	0.000	0.000	0.000	1.066	0.080	0.000
0.000	0.000	4.365	0.070	0.023	0.016	3.182	1.209	0.330	0.000	0.000	0.115	0.000	18.610	0.337	0.000
0.000	0.000	1.665	0.060	0.046	0.000	3.650	1.493	0.000	0.000	0.000	0.078	0.000	5.765	0.144	0.000
0.157	0.063	3.539	0.009	0.000	0.000	3.575	1.415	0.000	0.000	0.000	0.088	0.000	37.333	0.370	0.000
0.000	0.000	4.929	0.000	0.000	0.000	2.773	1.576	0.298	0.000	0.000	0.035	0.000	13.235	0.222	0.000
0.000	0.000	2.523	0.027	0.000	0.000	3.061	1.110	0.227	0.000	0.000	0.000	0.000	15.270	0.372	0.000
0.142	0.113	2.403	0.050	0.000	0.000	1.399	1.226	0.000	0.000	0.000	0.032	0.000	2.260	0.287	0.000
0.000	0.000	3.263	0.058	0.079	0.000	2.729	2.103	0.000	0.000	0.000	0.111	0.000	15.231	0.145	0.000
0.000	0.000	7.944	0.000	0.000	0.000	1.200	2.803	0.000	0.000	0.000	0.133	0.000	25.915	0.120	0.000
0.000	0.100	2.364	0.145	0.000	0.000	4.032	0.883	0.000	0.000	0.000	0.084	0.000	4.847	0.170	0.000
0.000	0.000	3.930	0.073	0.365	0.000	5.121	1.468	0.000	0.000	0.000	0.056	0.000	10.658	0.352	0.000
0.000	0.517	3.334	0.106	0.122	0.000	3.944	0.987	0.220	0.000	0.000	0.026	0.000	7.686	0.245	0.000
0.000	0.119	3.010	0.031	0.000	0.000	2.731	1.548	0.173	0.000	0.000	0.080	0.000	8.729	0.289	0.000
0.000	0.028	3.359	0.037	0.000	0.000	2.182	1.838	0.000	0.029	0.000	0.048	0.000	10.117	0.158	0.000
0.000	0.000	2.986	0.014	0.000	0.000	5.599	1.040	0.000	0.000	0.000	0.054	0.000	17.633	0.511	0.000
0.101	0.054	2.417	0.035	0.000	0.000	1.374	0.869	0.000	0.028	0.000	0.000	0.000	22.676	0.157	0.000
0.107	0.000	3.178	0.025	0.000	0.000	4.539	0.588	0.000	0.000	0.000	0.096	0.000	26.146	0.156	0.000
0.000	0.000	5.232	0.069	0.000	0.016	0.912	0.396	0.162	0.023	0.000	0.094	0.000	14.900	0.229	0.000
0.000	0.000	4.537	0.020	0.000	0.016	1.164	0.964	0.000	0.000	0.000	0.059	0.000	16.298	0.385	0.000
0.000	0.213	2.448	0.037	0.000	0.000	3.008	0.566	0.000	0.022	0.000	0.108	0.000	23.883	0.311	0.000
0.156	0.124	2.398	0.018	0.000	0.000	1.597	0.613	0.000	0.000	0.000	0.035	0.000	5.423	0.177	0.000
0.000	0.111	5.541	0.078	0.000	0.000	0.380	1.897	0.321	0.023	0.000	0.056	0.000	3.192	0.172	0.000
0.000	0.000	3.310	0.030	0.023	0.000	1.037	1.080	0.000	0.000	0.000	0.135	0.000	9.514	0.237	0.000
0.000	0.022	3.517	0.068	0.000	0.000	0.622	0.521	0.000	0.000	0.000	0.130	0.000	19.925	0.151	0.000
0.000	0.000	5.964	0.193	0.000	0.000	2.829	2.735	0.000	0.000	0.000	0.137	0.000	3.948	0.404	0.000
0.084	0.000	2.449	0.049	0.000	0.000	0.498	1.054	0.000	0.000	0.000	0.056	0.014	5.095	0.205	0.000
0.097	0.000	4.361	0.125	0.000	0.000	2.001	1.223	0.000	0.000	0.000	0.065	0.017	25.767	0.324	0.000
0.000	0.027	3.306	0.047	0.000	0.000	0.291	1.362	0.000	0.000	0.000	0.069	0.000	48.843	0.198	0.000
0.000	0.000	3.558	0.145	0.000	0.000	0.791	2.613	0.802	0.000	0.000	0.000	0.000	24.005	0.493	0.000
0.000	0.000	5.328	0.031	0.000	0.000	2.485	2.688	0.000	0.000	0.000	0.118	0.000	17.612	0.505	0.000
0.102	0.082	3.597	0.036	0.027	0.000	2.073	1.043	0.000	0.000	0.039	0.092	0.000	20.873	0.433	0.000
0.000	0.000	4.498	0.012	0.000	0.000	2.453	1.200	0.196	0.000	0.039	0.091	0.000	31.935	0.254	0.000
0.000	0.035	3.820	0.046	0.071	0.000	8.047	1.880	1.026	0.000	0.000	0.030	0.000	7.464	0.181	0.000
0.000	0.000	5.784	0.050	0.046	0.000	2.371	1.493	0.000	0.000	0.000	0.058	0.000	17.550	0.700	0.000
0.000	0.030	3.920	0.052	0.000	0.000	3.374	0.787	0.430	0.031	0.000	0.000	0.019	4.842	0.998	0.000
0.000	0.034	4.601	0.015	0.000	0.000	2.785	1.404	0.000	0.000	0.000	0.029	0.000	16.297	0.174	0.000
0.000	0.119	3.448	0.000	0.060	0.000	1.294	0.879	0.000	0.000	0.000	0.151	0.000	23.882	0.294	0.000
0.000	0.000	4.079	0.048	0.000	0.000	2.725	0.885	0.000	0.028	0.000	0.069	0.000	7.774	0.352	0.000
0.000	0.024	3.247	0.032	0.000	0.000	0.228	1.285	0.000	0.000	0.000	0.020	0.016	29.365	0.126	0.000
0.000	0.000	3.514	0.030	0.000	0.000	1.747	0.938	0.165	0.023	0.000	0.057	0.000	20.720	0.125	0.000
0.000	0.000	3.347	0.037	0.029	0.000	2.934	1.260	0.206	0.000	0.000	0.096	0.000	35.289	0.162	0.000
0.000	0.134	3.633	0.047	0.000	0.000	2.970	1.109	0.389	0.000	0.000	0.000	0.000	3.477	0.135	0.000
0.095	0.000	2.751	0.033	0.000	0.000	2.060	0.818	0.000	0.000	0.000	0.064	0.000	98.423	0.318	0.000
0.000	0.026	3.363	0.091	0.000	0.000	2.486	0.538	0.000	0.027	0.000	0.044	0.000	15.235	0.281	0.000
0.000	0.000	3.620	0.024	0.000	0.000	2.391	1.151	0.000	0.000	0.000	0.047	0.000	71.471	0.259	0.000
0.000	0.000	3.408	0.035	0.000	0.000	2.344	1.250	0.000	0.000	0.000	0.022	0.000	12.044	0.344	0.000
0.000	0.028	2.579	0.025	0.000	0.000	3.080	0.581	0.000	0.000	0.000	0.047	0.000	34.765	0.317	0.000
0.000	0.000	2.469	0.032	0.049	0.000	0.534	1.145	0.000	0.000	0.000	0.102	0.000	6.695	0.322	0.000
0.000	0.028	4.525	0.061	0.000	0.000	2.470	1.574	0.000	0.000	0.000	0.071	0.000	4.864	0.330	0.000
0.092	0.024	3.464	0.011	0.000	0.017	3.390	1.510	0.177	0.000	0.000	0.246	0.000	22.699	0.141	0.000
0.000	0.023	3.422	0.000	0.000	0.000	2.585	1.299	0.168	0.000	0.000	0.117	0.000	21.378	0.213	0.000
0.079	0.000	2.742	0.055	0.000	0.000	1.505	0.804	0.000	0.000	0.000	0.053	0.000	17.088	0.308	0.000
0.000	0.000	2.553	0.028	0.000	0.000	1.363	1.584	0.229	0.000	0.000	0.080	0.000	21.827	0.266	0.000
0.000	0.000	5.223	0.000	0.000	0.000	2.808	1.224	0.000	0.000	0.000	0.117	0.018	62.669	0.165	0.000
0.103	0.027	2.849	0.096	0.000	0.000	2.863	1.378	0.000	0.000	0.000	0.023	0.000	5.743	0.800	0.000
0.000	0.129	4.325	0.023	0.000	0.000	1.854	1.521	0.000	0.000	0.037	0.043	0.000	3.322	0.259	0.000
0.000	0.000	2.255	0.065	0.000	0.000	2.426	1.523	0.178	0.000	0.000	0.062	0.000	31.063	0.184	0.000
0.000	0.000	2.891	0.000	0.000	0.000	3.014	0.946	0.000	0.083	0.000	0.068	0.000	77.284	0.236	0.000
0.000	0.000	2.855	0.074	0.021	0.015	2.457	1.125	0.000	0.000	0.000	0.089	0.014	16.150	0.667	0.000
0.000	0.020	5.136	0.000	0.000	0.000	2.911	1.334	0.285	0.000	0.000	0.050	0.013	29.230	0.578	0.000
0.000	0.064	3.348	0.065	0.000	0.000	1.003	1.632	0.154	0.000	0.000	0.072	0.000	39.379	0.212	0.184
0.000	0.020	2.512	0.018	0.041	0.000	2.176	0.537	0.147	0.000	0.000	0.034	0.000	60.013	0.405	0.000
0.000	0.064	2.107	0.028	0.000	0.000	1.986	1.316	0.000	0.000	0.000	0.161	0.000	40.807	0.121	0.000
0.000	0.046	3.450	0.181	0.000	0.000	2.298	1.834	0.000	0.000	0.000	0.078	0.000	8.453	0.155	0.000
0.000	0.030	4.336	0.039	0.000	0.000	0.706	2.618	0.000	0.000	0.000	0.075	0.038	17.115	0.185	0.000
0.113	0.000	3.842	0.000	0.030	0.000	2.757	0.978	0.218	0.000	0.000	0.051	0.000	7.053	0.214	0.000
0.000	0.000	3.217	0.015	0.000	0.000	2.578	1.283	0.242	0.000	0.000	0.056	0.000	39.918	0.263	0.289
0.000	0.075	2.813	0.033	0.038	0.000	2.337	1.109	0.272	0.000	0.000	0.095	0.000	10.065	0.239	0.000
0.000	0.000	4.377	0.027	0.000	0.000	0.353	1.170	0.221	0.000	0.000	0.129	0.000	31.510	0.122	0.000
0.000	0.021	5.252	0.028	0.000	0.000	2.179	1.693	0.000	0.000	0.000	0.197	0.000	42.196	0.240	0.000
0.000	0.022	3.201	0.038	0.000	0.000	2.194	1.672	0.158	0.000	0.000	0.092	0.000	2.402	0.482	0.000
0.146	0.039	4.112	0.034	0.000	0.000	2.442	1.836	0.000	0.000	0.000	0.098	0.000	60.966	0.328	0.000
0.000	0.061	3.915	0.000	0.000	0.014	5.618	1.914	0.147	0.000	0.000	0.051	0.000	17.634	0.379	0.000
0.000	0.027	2.999	0.036	0.000	0.000	2.477	3.321	0.000	0.000	0.000	0.046	0.018	20.345	0.573	0.000
0.000	0.099	4.698	0.043	0.000	0.000	2.085	0.874	0.000	0.000	0.000	0.062	0.000	10.171	0.297	0.000
0.000	0.097	2.791	0.021	0.000	0.000	2.833	1.572	0.175	0.000	0.000	0.020	0.000	9.388	0.149	0.000

ENSG00000251011	ENSG00000164877	ENSG00000278202	ENSG00000230161	ENSG00000132953	ENSG00000258304	ENSG00000201372	ENSG00000213451	ENSG00000228723	ENSG00000250493	ENSG00000236197	ENSG00000204856	ENSG00000278999	ENSG00000106624	ENSG00000117650	ENSG00000258479
0.009	6.462	0.158	0.000	2.308	0.063	0.000	0.000	0.000	0.000	0.867	3.524	0.022	107.329	2.127	0.016
0.015	6.689	0.188	0.000	1.327	0.162	0.197	0.000	0.000	0.010	0.041	5.840	0.000	114.176	2.352	0.081
0.000	1.779	0.048	0.000	2.164	0.048	0.000	0.000	0.039	0.015	0.160	9.947	0.008	11.159	9.568	0.048
0.008	2.661	0.297	0.000	3.332	0.054	0.000	0.000	0.000	0.003	0.735	5.333	0.009	116.615	3.695	0.081
0.009	8.783	0.180	0.000	1.670	0.061	0.000	0.000	0.000	0.004	0.101	5.890	0.000	276.348	0.633	0.196
0.027	5.208	0.546	0.000	5.498	0.048	0.000	0.000	0.026	0.027	0.203	7.493	0.083	2.880	6.888	0.095
0.015	9.763	0.054	0.000	2.264	0.000	0.000	0.000	0.000	0.007	0.025	7.125	0.019	192.246	4.743	0.054
0.073	2.778	0.028	0.000	1.568	0.000	0.000	0.000	0.000	0.014	1.634	5.689	0.010	40.915	0.230	0.043
0.009	3.820	0.033	0.000	1.860	0.000	0.240	0.000	0.000	0.012	0.560	8.653	0.011	57.417	3.641	0.082
0.027	6.718	0.000	0.000	1.637	0.000	0.000	0.000	0.052	0.036	0.145	5.645	0.033	43.687	0.869	0.048
0.020	14.766	0.023	0.000	2.124	0.047	0.000	0.000	0.013	0.009	0.172	6.082	0.016	47.534	0.753	0.082
0.042	14.190	0.169	0.000	3.718	0.000	0.355	0.000	0.013	0.015	0.052	4.204	0.025	75.966	4.065	0.085
0.000	3.650	0.050	0.000	1.617	0.000	0.000	0.000	0.000	0.006	1.565	4.260	0.026	131.718	2.382	0.038
0.007	7.437	0.164	0.000	1.823	0.000	0.000	0.000	0.000	0.041	0.221	3.058	0.057	149.790	2.056	0.058
0.060	4.297	0.000	0.000	2.055	0.000	0.000	0.000	0.000	0.013	1.018	4.542	0.000	77.683	3.433	0.156
0.000	3.635	0.047	0.000	2.866	0.000	0.000	0.000	0.013	0.003	0.236	2.341	0.025	126.289	0.166	0.035
0.000	2.700	0.081	0.000	3.369	0.054	0.000	0.000	0.000	0.003	0.049	5.614	0.000	53.329	5.862	0.095
0.009	1.495	0.032	0.000	1.084	0.000	0.000	0.000	0.017	0.008	0.890	21.772	0.000	9.216	4.921	0.080
0.014	13.324	0.049	0.000	1.218	0.098	0.000	0.000	0.000	0.012	0.328	3.570	0.017	39.143	1.699	0.073
0.000	7.053	0.078	0.000	2.346	0.052	0.191	0.000	0.014	0.006	0.501	4.307	0.027	84.441	2.490	0.156
0.024	2.755	0.000	0.000	0.859	0.112	0.000	0.000	0.000	0.010	2.180	13.830	0.000	15.003	7.154	0.056
0.008	2.214	0.402	0.000	4.120	0.000	0.000	0.000	0.016	0.025	0.675	5.819	0.030	14.086	3.283	0.158
0.144	2.405	0.028	0.000	2.243	0.113	0.206	0.000	0.000	0.010	0.660	10.475	0.049	18.361	5.832	0.098
0.075	15.206	0.048	0.000	2.030	0.000	0.000	0.000	0.013	0.003	0.073	3.537	0.000	189.920	4.326	0.024
0.000	5.560	0.053	0.000	1.664	0.000	0.000	0.000	0.014	0.003	0.106	3.946	0.028	129.246	1.650	0.040
0.000	12.702	0.138	0.000	2.734	0.000	0.203	0.000	0.000	0.010	0.633	5.355	0.019	192.817	6.115	0.055
0.009	6.799	0.031	0.000	2.004	0.189	0.229	0.000	0.034	0.000	0.802	5.157	0.000	105.576	1.790	0.047
0.016	8.041	0.110	0.000	2.097	0.111	0.404	0.000	0.000	0.027	0.471	7.261	0.039	89.025	7.045	0.055
0.000	16.253	0.082	0.000	2.132	0.055	0.402	0.000	0.000	0.010	1.038	6.178	0.077	123.833	1.786	0.219
0.000	6.965	0.083	0.000	2.675	0.111	0.202	0.000	0.000	0.017	0.236	6.897	0.029	48.719	3.899	0.248
0.045	4.474	0.091	0.000	1.621	0.046	0.166	0.000	0.012	0.003	0.679	9.001	0.016	130.060	4.736	0.000
0.000	10.091	0.190	0.000	2.745	0.048	0.174	0.000	0.013	0.009	0.044	4.110	0.067	176.801	1.741	0.119
0.016	7.258	0.055	0.000	1.250	0.000	0.203	0.000	0.030	0.010	0.751	7.114	0.019	163.404	0.575	0.028
0.053	19.916	0.000	0.000	1.621	0.054	0.000	0.000	0.000	0.020	0.252	4.632	0.019	267.614	1.459	0.053
0.016	11.627	0.112	0.000	3.343	0.000	0.000	0.000	0.000	0.007	0.086	4.057	0.039	298.895	2.938	0.028
0.095	13.161	0.127	0.000	2.304	0.051	0.374	0.000	0.014	0.013	1.969	5.419	0.018	89.337	0.536	0.102
0.049	8.801	0.268	0.000	2.279	0.049	0.000	0.000	0.013	0.057	3.591	4.822	0.043	341.849	1.395	0.183
0.021	3.268	0.243	0.000	2.567	0.049	0.000	0.000	0.013	0.015	0.802	4.116	0.017	129.048	4.154	0.134
0.033	6.706	0.116	0.000	2.161	0.000	0.000	0.000	0.000	0.007	2.560	9.530	0.010	57.221	6.110	0.087
0.017	6.894	0.389	0.000	4.075	0.000	0.439	0.000	0.000	0.015	0.366	8.770	0.053	157.469	7.938	0.255
0.051	10.941	0.089	0.000	2.767	0.060	0.219	0.000	0.033	0.022	0.219	7.945	0.052	89.862	1.217	0.060
0.036	25.188	0.756	0.000	3.465	0.000	0.185	0.000	0.014	0.013	0.100	8.654	0.053	85.031	1.731	0.013
0.063	4.920	0.139	0.000	2.362	0.000	0.203	0.000	0.000	0.007	0.059	8.187	0.029	62.853	3.255	0.014
0.007	20.309	0.214	0.000	3.736	0.048	0.175	0.000	0.000	0.003	0.749	5.806	0.017	200.293	2.389	0.048
0.029	8.290	0.253	0.000	4.088	0.204	0.000	0.000	0.000	0.013	0.093	6.917	0.027	57.770	1.993	0.165
0.021	9.874	0.000	0.000	2.068	0.000	0.000	0.000	0.013	0.061	2.085	5.210	0.026	125.587	4.603	0.235
0.047	2.033	0.681	0.000	4.339	0.000	0.000	0.000	0.015	0.000	0.641	6.288	0.048	35.536	10.206	0.177
0.016	4.410	0.303	0.000	3.191	0.055	0.809	0.000	0.000	0.021	1.238	7.078	0.029	47.254	2.509	0.152
0.014	5.154	0.263	0.000	2.236	0.096	0.000	0.000	0.000	0.009	0.044	7.146	0.017	207.497	4.030	0.012
0.039	5.045	0.000	0.000	1.720	0.045	0.000	0.000	0.000	0.000	0.083	5.108	0.016	31.255	1.213	0.023
0.110	2.916	0.068	0.000	1.766	0.000	0.000	0.000	0.000	0.011	0.964	14.213	0.048	23.374	3.631	0.045
0.042	4.792	0.049	0.000	1.195	0.000	0.000	0.000	0.013	0.003	0.313	6.782	0.034	36.757	1.287	0.061
0.000	3.526	0.124	0.000	2.301	0.050	0.000	0.000	0.014	0.006	0.471	8.832	0.017	68.940	9.305	0.062
0.000	9.430	0.066	0.000	2.098	0.044	0.000	0.000	0.000	0.005	0.094	5.603	0.008	61.143	2.194	0.044
0.012	1.278	0.243	0.000	1.995	0.163	0.000	0.000	0.022	0.035	0.927	13.626	0.014	17.351	20.878	0.142
0.000	1.412	0.105	0.000	1.842	0.212	0.000	0.000	0.000	0.013	3.250	8.168	0.000	21.756	4.487	0.000
0.007	19.220	0.183	0.000	2.380	0.000	0.000	0.000	0.000	0.000	0.256	8.620	0.018	280.677	1.965	0.157
0.000	3.219	0.099	0.000	1.714	0.000	0.000	0.000	0.013	0.000	0.242	6.121	0.009	65.447	7.666	0.037
0.021	5.107	0.172	0.000	3.116	0.049	0.000	0.000	0.013	0.009	4.348	4.513	0.034	55.384	5.472	0.221
0.013	2.994	0.115	0.000	2.557	0.000	0.000	0.000	0.000	0.037	2.389	9.708	0.000	80.690	5.611	0.011
0.000	6.866	0.238	0.000	4.293	0.107	0.000	0.000	0.000	0.112	6.348	5.455	0.009	72.834	3.743	0.080
0.012	2.788	0.000	0.000	2.150	0.000	0.617	0.000	0.046	0.010	0.090	7.768	0.015	56.587	9.618	0.042
0.000	5.906	0.219	0.000	2.990	0.000	0.161	0.000	0.012	0.027	0.261	8.962	0.038	84.350	4.713	0.471
0.013	11.267	0.211	0.000	2.628	0.047	0.000	0.000	0.000	0.309	0.251	3.687	0.033	110.333	4.536	0.235
0.022	3.613	0.131	0.000	2.759	0.000	0.000	0.023	0.000	0.003	8.689	6.810	0.028	113.760	5.480	0.065
0.010	2.303	0.000	0.000	1.538	0.068	0.000	0.000	0.000	0.000	1.304	9.161	0.000	15.775	4.143	0.067
0.000	3.560	0.000	0.000	1.720	0.000	0.000	0.000	0.025	0.000	0.239	9.779	0.032	46.738	10.429	0.069
0.009	3.907	0.092	0.000	0.613	0.124	0.000	0.000	0.000	0.004	0.056	14.012	0.011	31.277	4.731	0.062
0.152	3.790	0.041	0.000	1.604	0.000	0.000	0.000	0.000	0.005	0.150	4.847	0.000	214.282	3.710	0.020
0.000	3.103	0.000	0.000	1.064	0.130	0.000	0.000	0.018	0.008	0.779	8.973	0.011	45.032	7.564	0.065
0.000	7.321	0.047	0.000	1.685	0.000	0.000	0.000	0.013	0.000	0.065	5.608	0.008	118.741	4.713	0.035
0.027	2.275	0.000	0.000	0.843	0.000	0.000	0.000	0.104	0.000	0.450	10.982	0.017	61.748	0.410	0.000
0.031	18.479	0.043	0.000	1.634	0.043	0.000	0.000	0.000	0.005	0.356	3.688	0.023	77.403	1.121	0.032
0.012	10.067	0.127	0.000	1.404	0.256	0.000	0.000	0.000	0.005	0.130	6.613	0.000	106.679	2.585	0.064
0.000	8.493	0.097	0.000	2.250	0.000	0.000	0.000	0.000	0.008	0.109	3.603	0.000	222.448	0.419	0.049
0.011	3.618	0.039	0.000	2.612	0.079	0.286	0.000	0.043	0.010	2.445	6.256	0.000	26.242	3.693	0.195
0.000	3.013	0.082	0.000	1.781	0.055	0.000	0.000	0.015	0.003	0.409	8.348	0.010	25.245	6.014	0.068
0.047	2.644	0.164	0.000	5.848	0.000	0.000	0.000	0.000	0.000	0.150	5.973	0.057	80.292	9.629	0.000
0.088	2.231	0.000	0.000	2.416	0.138	0.252	0.000	0.038	0.009	1.166	15.566	0.012	30.569	4.551	0.086
0.000	3.323	0.000	0.000	1.994	0.000	0.503	0.000	0.000	0.004	0.073	6.881	0.000	190.044	2.226	0.000
0.000	2.107	0.000	0.000	1.572	0.000	0.000	0.000	0.034	0.000	0.384	15.139	0.022	39.142	4.289	0.063
0.000	2.840	0.074	0.000	2.014	0.050	0.181	0.000	0.040	0.003	0.437	9.016	0.009	43.360	1.463	0.037
0.025	5.105	0.117	0.000	1.965	0.000	0.215	0.000	0.032	0.007	1.601	4.381	0.031	196.006	1.670	0.161
0.000	4.164	0.033	0.000	1.654	0.133	0.486	0.000	0.000	0.004	0.648	4.317	0.012	314.337	0.933	0.050
0.008	2.926	0.111	0.000	2.257	0.056	0.000	0.000	0.000	0.003	0.896	3.827	0.000	61.744	2.576	0.055
0.017	11.318	0.088	0.000	1.765	0.059	0.000	0.000	0.000	0.007	0.135	3.144	0.000	92.044	0.656	0.059
0.020	6.234	0.301	0.000	7.884	0.093	0.000	0.000	0.025	0.029	0.191	6.469	0.008	28.026	6.153	0.012
0.000	4.045	0.000	0.000	2.480	0.000	0.177	0.000	0.039	0.003	2.049	5.399	0.025	269.633	11.110	0.121
0.000	6.349	0.132	0.000	2.263	0.000	0.000	0.000	0.000	0.008	0.195	4.592	0.015	49.286	3.822	0.055
0.000	7.726	0.215	0.000	2.188	0.000	0.000	0.000	0.000	0.005	0.131	4.393	0.000	73.133	0.860	0.064
0.026	7.589	0.390	0.000	2.748	0.092	0.000	0.000	0.012	0.020	0.763	8.735	0.145	118.022	8.287	0.229
0.014	6.499	0.095	0.000	1.003	0.000	0.000	0.000	0.026	0.032	0.434	4.917	0.017	129.128	2.904	0.024
0.013	3.520	0.023	0.000	1.713	0.092	0.000	0.000	0.037	0.006	0.886	5.175	0.000	8.256	2.050	0.023
0.027	1.962	0.335	0.000	5.558	0.000	0.351	0.000	0.013	0.030	0.344	9.253	0.025	174.590	3.973	0.060
0.046	5.079	0.231	0.000	2.489	0.000	0.678	0.000	0.013	0.014	2.265	5.741	0.049	4.605	0.187	0.150
0.023	10.523	0.241	0.000	2.427	0.054	0.393	0.000	0.000	0.063	0.327	6.896	0.028	277.915	5.862	0.147
0.008	2.409	0.168	0.000	2.637	0.000	0.206	0.000	0.031	0.021	2.958	7.529	0.010	6.073	1.658	0.169
0.817	5.536	0.458	0.000	3.319	0.000	0.000	0.000	0.624	0.085	0.524	7.961	0.040	70.154	8.329	0.057
0.041	6.873	0.507	0.000	3.113	0.146	0.000	0.000	0.000	0.009	3.165	6.010	0.000	157.072	4.170	0.399
0.040	11.573	0.253	0.000	2.730	0.170	0.206	0.000	0.015	0.021	0.112	4.922	0.010	87.295	1.918	0.000
0.016	14.151	0.140	0.000	2.040	0.000	0.206	0.000	0.015	0.014	0.925	5.185	0.010	191.888	2.210	0.126
0.157	5.752	0.000	0.000	1.781	0.000	0.000	0.000	0.020	0.009	3.510	13.426	0.000	33.989	3.433	0.165
0.000	11.989	0.071	0.000	1.599	0.191	0.000	0.000	0.052	0.003	0.116	7.320	0.042	108.663	3.479	0.059
0.018	10.061	0.092	0.000	2.222	0.062	0.000	0.000	0.134	0.023	2.191	4.595	0.021	183.023	1.212	0.123
0.020	4.853	0.246	0.000	2.971	0.071	0.000	0.000	0.019	0.022	0.279	5.680	0.000	32.178	2.094	0.281
0.123	9.450	0.000	0.000	2.711	0.000	0.000	0.000	0.000	0.023	0.433	4.301	0.000	61.127	3.154	0.185
0.097	3.367	0.056	0.000	1.386	0.057	0.000	0.000	0.046	0.077	3.979	6.067	0.020	163.010	3.510	0.099
0.000	1.206	0.050	0.000	2.360	0.000	0.000	0.000	0.000	0.012	1.804	13.409	0.009	7.958	2.842	0.188
0.027	8.940	0.047	0.000	2.905	0.000	0.345	0.000	0.000	0.003	0.086	9.180	0.016	53.376	4.419	0.012
0.000	6.722	0.029	0.055	1.117	0.000	0.000	0.000	0.000	0.004	0.072	7.324	0.000	52.300	2.552	0.044
0.016	9.462	0.028	0.000	0.863	0.000	0.000	0.000	0.015	0.000	0.771	10.347	0.000	117.466	1.428	0.042
0.000	5.228	0.156	0.000	2.408	0.000	0.191	0.000	0.014	0.013	0.080	6.845	0.046	54.789	4.557	0.104
0.008	6.460	0.081	0.000	2.442	0.108	0.000	0.000	0.044	0.010	0.452	5.116	0.019	100.194	2.306	0.162
0.000	7.463	0.058	0.000	3.003	0.000	0.000	0.000	0.000	0.036	0.106	4.206	0.040	118.220	5.817	0.202
0.023	7.836	0.082	0.000	2.165	0.055	0.000	0.000	0.000	0.010	0.418	6.884	0.010	5.126	6.283	0.069
0.017	8.437	0.116	0.000	1.736	0.000	0.000	0.000	0.000	0.000	0.107	3.431	0.010	62.833	0.833	0.015
0.021	5.667	0.075	0.000	2.519	0.101	0.000	0.000	0.000	0.016	0.092	6.542	0.009	60.274	3.727	0.025
0.000	2.178	0.203	0.000	3.151	0.058	0.000	0.000	0.016	0.007	0.479	8.212	0.010	141.855	3.755	0.015
0.007	3.117	0.151	0.000	2.452	0.152	0.000	0.000	0.000	0.000	0.069	8.497	0.000	65.646	4.715	0.038
0.027	4.081	0.024	0.000	3.052	0.000	0.000	0.000	0.013	0.003	0.051	4.140	0.017	141.217	4.582	0.012
0.000	2.374	0.087	0.000	2.165	0.000	0.000	0.000	0.047	0.013	0.430	4.950	0.008	100.575	4.403	0.043
0.009	2.373	0.228	0.000	1.761	0.000	0.000	0.000	0.000	0.012	0.499	5.611	0.034	85.723	4.941	0.082
0.025	3.728	0.000	0.000	2.415	0.000	0.000	0.000	0.000	0.000	0.052	3.457	0.020	243.173	3.060	0.000
0.000	1.270	0.170	0.000	2.021	0.171	0.000	0.000	0.015	0.004	2.013	7.520	0.000	119.059	3.320	0.156
0.015	10.534	0.133	0.000	1.753	0.054	0.000	0.000	0.015	0.033	1.083	5.129	0.028	643.529	3.988	0.080
0.189	8.264	0.000	0.000	1.845	0.000	0.000	0.000	0.000	0.003	0.334	7.587	0.027	33.816	1.444	0.025
0.047	7.686	0.166	0.000	2.174	0.000	0.000	0.000	0.000	0.000	0.278	6.072	0.029	77.902	2.248	0.124
0.006	12.754	0.329	0.000	2.753	0.088	0.161	0.000	0.012	0.011	9.772	4.922	0.023	324.803	3.222	0.066
0.017	10.100	0.061	0.000	2.476	0.000	0.000	0.000	0.011	0.263	2.154	5.532	0.021	63.447	5.312	0.031
0.000	4.467	0.220	0.000	2.357	0.044	0.000	0.000	0.000	0.044	0.363	7.541	0.023	7.091	5.932	0.033
0.018	6.764	0.167	0.000	2.023	0.000	0.000	0.000	0.011	0.008	0.051	3.513	0.007	119.794	1.395	0.042
0.075	2.760	0.000	0.000	1.477	0.133	0.000	0.000	0.036	0.008	0.242	4.771	0.000	103.826	3.809	0.099
0.007	2.419	0.071	0.000	2.042	0.000	0.000	0.000	0.013	0.000	0.771	9.690	0.017	34.527	5.776	0.179
0.009	11.940	0.275	0.000	1.154	0.000	0.224	0.000	0.000	0.034	0.196	10.713	0.064	90.859	2.419	0.031
0.062	9.178	0.125	0.000	1.902	0.000	0.228	0.000	0.000	0.008	0.619	6.012	0.011	176.796	1.998	0.062
0.010	7.299	0.035	0.000	1.610	0.139	0.000	0.000	0.094	0.009	0.317	5.028	0.036	143.443	2.789	0.087
0.022	15.630	0.155	0.000	1.672	0.000	0.000	0.000	0.021	0.014	0.297	3.687	0.068	94.229	1.734	0.000
0.027	2.391	0.063	0.000	3.103	0.000	0.000	0.000	0.000	0.035	0.511	8.479	0.011	43.594	8.374	0.111
0.006	5.569	0.395	0.000	4.340	0.088	0.322	0.000	0.012	0.003	0.450	8.433	0.031	35.248	9.374	0.671
0.032	3.916	0.068	0.000	3.010	0.000	0.000	0.000	0.012	0.020	4.413	6.283	0.000	106.835	2.886	0.045
0.023	5.718	0.080	0.000	2.630	0.000	0.000	0.000	0.000	0.000	0.209	4.311	0.014	101.746	2.794	0.060
0.084	11.995	0.063	0.000	2.132	0.000	0.000	0.000	0.000	0.010	2.971	5.294	0.015	142.627	2.950	0.042
0.000	3.734	0.057	0.053	1.475	0.000	0.000	0.000	0.031	0.095	0.425	7.785	0.010	105.743	1.612	0.000
0.007	21.296	0.255	0.000	2.887	0.051	0.187	0.000	0.014	0.032	0.585	5.804	0.009	137.064	4.768	0.204
0.007	5.641	0.050	0.000	2.055	0.000	0.184	0.000	0.000	0.009	0.199	6.425	0.009	84.536	5.626	0.050

ENSG00000264519	ENSG00000204387	ENSG00000248583	ENSG00000273001	ENSG00000072518	ENSG00000125841	ENSG00000196139	ENSG00000224003	ENSG00000138279	ENSG00000263510	ENSG00000155957	ENSG00000257467	ENSG00000259914	ENSG00000229238	ENSG00000230098	ENSG00000200884
0.000	32.802	0.037	0.771	8.253	32.994	4.651	0.182	38.413	0.000	8.746	0.049	0.000	0.000	0.157	0.000
0.000	66.562	0.000	0.801	5.755	26.488	1.709	0.129	37.909	0.000	10.397	0.135	0.000	0.000	0.045	0.000
0.000	98.133	0.056	0.745	8.790	18.909	1.160	0.115	24.208	0.000	9.191	0.000	0.000	0.000	0.000	0.000
0.000	58.623	0.000	0.330	8.837	27.085	0.685	0.248	20.187	0.000	9.042	0.115	0.030	0.000	0.000	0.000
0.000	102.227	0.000	1.062	5.143	28.421	2.420	0.116	39.780	0.000	18.672	0.058	0.000	0.000	0.000	0.000
0.000	44.678	0.418	1.033	10.129	21.520	1.061	0.390	24.002	0.000	5.455	0.129	0.027	0.011	0.000	0.000
0.000	37.688	0.000	0.545	8.781	31.412	2.222	0.542	39.376	0.000	13.123	0.187	0.000	0.000	0.044	0.206
0.000	70.962	0.033	0.922	6.054	39.082	3.014	0.287	49.584	0.000	14.225	0.055	0.000	0.000	0.094	0.000
0.000	74.516	0.038	0.622	5.022	31.333	2.412	0.189	29.706	0.000	13.136	0.114	0.037	0.000	0.000	0.000
0.259	66.880	0.000	0.517	6.144	14.623	5.479	0.413	50.127	0.000	20.193	0.037	0.000	0.000	0.000	0.000
0.000	47.932	0.000	0.635	4.806	25.714	7.860	0.259	35.487	0.000	22.136	0.172	0.026	0.011	0.117	0.000
0.000	69.368	0.000	0.921	9.687	51.135	1.126	0.105	27.112	0.000	4.966	0.094	0.000	0.000	0.040	0.000
0.000	52.644	0.146	0.339	7.131	40.409	1.180	0.205	23.857	0.000	10.637	0.058	0.000	0.000	0.000	0.000
0.000	60.323	0.082	0.508	9.134	47.934	0.833	0.169	27.644	0.000	8.671	0.054	0.104	0.011	0.000	0.000
0.000	62.938	0.000	0.848	7.357	47.368	3.857	0.201	25.500	0.000	8.494	0.040	0.000	0.000	0.000	0.000
0.000	33.168	0.000	0.413	9.537	24.999	2.309	0.101	38.999	0.000	8.910	0.209	0.000	0.000	0.078	0.000
0.000	76.816	0.000	0.586	8.383	35.880	0.516	0.351	18.588	0.000	3.782	0.105	0.000	0.025	0.000	0.000
0.000	140.460	0.000	0.739	3.855	47.889	9.931	0.046	48.954	0.000	21.735	0.037	0.000	0.256	0.000	0.000
0.000	55.746	0.000	0.331	5.988	48.569	1.394	0.094	42.390	0.000	8.709	0.057	0.000	0.000	0.041	0.000
0.000	57.585	0.092	0.707	8.345	37.556	0.552	0.075	28.316	0.000	7.798	0.020	0.000	0.000	0.000	0.000
0.000	126.325	0.000	0.830	3.471	27.940	4.478	0.054	56.793	0.000	17.517	0.022	0.031	0.000	0.000	0.000
0.000	44.271	0.034	1.403	13.377	41.703	1.098	0.249	27.521	0.000	4.520	0.122	0.128	0.040	0.000	0.000
0.000	105.360	0.000	0.724	6.188	31.673	1.948	0.446	29.851	0.000	13.739	0.131	0.000	0.013	0.000	0.000
0.000	47.885	0.000	0.162	8.284	35.929	0.773	0.081	35.584	0.000	10.919	0.028	0.027	0.011	0.000	0.000
0.145	83.922	0.000	0.397	7.301	44.992	1.657	0.128	29.439	0.234	10.801	0.041	0.000	0.000	0.044	0.204
0.000	51.089	0.000	0.413	10.097	46.073	0.676	0.107	26.483	0.000	7.284	0.129	0.000	0.000	0.000	0.000
0.000	48.427	0.000	0.255	6.530	31.308	0.624	0.060	33.413	0.000	11.214	0.061	0.000	0.015	0.000	0.000
0.000	105.720	0.000	0.896	8.110	39.451	1.488	0.212	24.303	0.000	13.139	0.021	0.062	0.000	0.000	0.000
0.000	76.041	0.032	0.744	6.649	46.421	2.072	0.291	22.239	0.000	12.578	0.149	0.000	0.013	0.000	0.000
0.000	109.580	0.032	0.711	7.443	33.010	1.695	0.770	21.754	0.000	12.985	0.171	0.062	0.000	0.000	0.000
0.000	142.141	0.106	1.139	7.229	75.291	0.515	0.164	26.720	0.000	14.048	0.000	0.000	0.011	0.075	0.000
0.000	59.360	0.028	0.581	8.412	53.115	0.634	0.149	24.334	0.000	10.923	0.055	0.000	0.000	0.039	0.000
0.000	86.204	0.000	2.627	6.629	50.766	2.867	0.266	34.921	0.000	12.646	0.075	0.062	0.013	0.000	0.000
0.000	75.722	0.031	0.542	5.958	46.397	1.652	0.244	29.595	0.000	15.795	0.031	0.000	0.000	0.000	0.000
0.000	69.176	0.066	0.687	8.193	36.682	0.601	0.108	32.964	0.000	12.112	0.022	0.000	0.000	0.094	0.000
0.000	67.865	0.030	1.972	7.259	64.990	1.220	0.147	34.049	0.000	14.308	0.020	0.000	0.012	0.000	0.000
0.000	49.736	0.143	1.190	9.593	34.513	3.518	0.059	17.384	0.000	7.096	0.349	0.000	0.000	0.000	0.000
0.000	100.636	0.171	0.561	8.815	37.069	1.399	0.223	30.838	0.000	11.541	0.057	0.082	0.011	0.000	0.000
0.000	70.603	0.204	1.339	8.586	31.243	1.743	0.084	29.426	0.000	11.171	0.090	0.032	0.000	0.000	0.000
0.082	61.065	0.175	2.522	9.554	27.125	0.950	0.347	20.232	0.000	8.501	0.592	0.033	0.000	0.050	0.000
0.081	31.653	0.000	0.728	8.005	45.037	0.817	0.273	31.712	0.000	17.104	0.370	0.033	0.000	0.000	0.000
0.000	63.044	0.030	1.197	7.412	58.408	1.250	0.061	29.552	0.000	8.521	0.029	0.000	0.000	0.000	0.000
0.000	76.490	0.097	0.565	7.919	43.200	0.716	0.120	27.047	0.000	11.301	0.301	0.000	0.000	0.046	0.000
0.000	53.426	0.000	0.743	8.384	33.182	0.951	0.229	28.994	0.000	11.126	0.074	0.000	0.022	0.079	0.000
0.000	47.972	0.059	0.895	7.459	30.339	0.695	0.147	24.709	0.000	10.788	0.147	0.028	0.000	0.000	0.195
0.067	55.624	0.000	1.041	9.499	43.631	1.875	0.072	27.802	0.000	10.499	0.221	0.000	0.000	0.000	0.000
0.074	193.518	0.064	0.592	8.422	22.494	5.034	0.131	29.100	0.000	16.195	0.158	0.000	0.013	0.000	0.000
0.000	61.101	0.032	0.748	6.337	25.861	2.165	0.465	28.460	0.000	7.162	0.192	0.031	0.000	0.046	0.000
0.000	76.539	0.028	0.714	8.463	40.374	0.379	0.012	28.564	0.000	9.665	0.028	0.000	0.000	0.040	0.000
0.000	51.899	0.000	0.368	6.567	25.987	2.566	0.109	43.890	0.000	16.943	0.017	0.076	0.000	0.038	0.174
0.000	90.789	0.159	1.787	7.799	48.991	5.040	0.022	46.864	0.000	12.523	0.141	0.051	0.021	0.113	0.000
0.000	62.031	0.000	0.431	5.981	35.093	1.980	0.047	39.885	0.000	14.104	0.028	0.000	0.000	0.000	0.000
0.000	87.793	0.058	0.472	6.099	38.093	1.537	0.084	36.129	0.000	15.672	0.096	0.000	0.012	0.000	0.000
0.000	62.197	0.000	0.447	7.107	51.324	0.522	0.085	33.034	0.000	9.860	0.043	0.000	0.000	0.037	0.000
0.110	63.597	0.000	2.196	7.483	36.390	7.170	0.058	35.319	0.000	7.204	0.172	0.000	0.000	0.000	0.000
0.000	83.448	0.000	0.429	6.058	23.676	6.860	0.203	52.281	0.000	7.953	0.041	0.000	0.000	0.000	0.000
0.000	121.127	0.000	1.493	6.871	39.812	0.618	0.189	32.091	0.000	13.321	0.020	0.000	0.000	0.000	0.000
0.000	40.479	0.000	0.605	6.029	42.828	2.289	0.072	30.554	0.000	7.377	0.048	0.000	0.000	0.000	0.000
0.000	56.019	0.029	2.236	8.141	26.795	0.796	0.178	38.224	0.000	7.587	0.057	0.055	0.000	0.000	0.000
0.000	59.357	0.054	0.093	7.396	38.080	0.739	0.044	34.629	0.000	7.378	0.062	0.000	0.011	0.114	0.000
0.072	82.752	0.155	1.332	11.350	48.551	11.983	0.102	32.771	0.000	6.320	0.123	0.000	0.025	0.000	0.000
0.000	84.787	0.049	0.171	5.829	38.819	1.029	0.182	23.074	0.000	10.458	0.000	0.000	0.000	0.000	0.000
0.000	83.941	0.000	1.575	11.036	38.973	1.339	0.475	10.212	0.000	6.230	0.221	0.000	0.144	0.036	0.000
0.064	62.677	0.000	0.446	10.101	41.345	3.940	0.147	25.054	0.000	6.604	0.036	0.000	0.022	0.039	0.000
0.000	94.784	0.123	0.853	7.414	36.925	1.587	0.088	32.043	0.000	12.618	0.010	0.000	0.000	0.000	0.000
0.000	72.434	0.039	0.867	10.228	58.491	2.548	0.081	24.528	0.000	9.348	0.078	0.075	0.016	0.000	0.259
0.126	144.360	0.108	0.250	7.479	45.007	1.343	0.022	23.087	0.000	10.339	0.036	0.000	0.000	0.000	0.000
0.000	97.491	0.000	0.459	4.838	68.188	0.244	0.030	27.438	0.000	14.853	0.024	0.000	0.000	0.000	0.000
0.000	60.552	0.000	0.222	7.834	37.193	0.507	0.079	29.710	0.000	13.111	0.000	0.000	0.019	0.068	0.000
0.000	125.596	0.038	0.044	4.821	34.959	3.376	0.062	39.792	0.000	19.371	0.000	0.000	0.000	0.000	0.000
0.000	90.586	0.000	0.639	6.691	46.136	1.005	0.034	28.434	0.000	9.168	0.018	0.000	0.011	0.000	0.000
0.000	67.254	0.111	0.646	3.882	40.393	4.482	0.092	50.603	0.000	20.833	0.055	0.000	0.000	0.079	0.000
0.000	51.191	0.025	0.820	5.474	44.886	1.426	0.166	27.348	0.000	9.476	0.184	0.000	0.000	0.000	0.000
0.000	80.271	0.000	1.038	7.282	80.495	1.061	0.205	25.070	0.000	7.963	0.082	0.000	0.000	0.071	0.000
0.000	39.905	0.038	0.572	8.765	35.021	0.502	0.141	21.602	0.000	7.998	0.038	0.000	0.000	0.000	0.000
0.000	71.959	0.046	0.742	6.225	27.187	4.077	0.207	26.244	0.000	15.667	0.091	0.000	0.000	0.000	0.000
0.000	80.339	0.000	0.185	4.027	37.421	2.450	0.079	42.697	0.000	17.148	0.053	0.061	0.000	0.000	0.000
0.000	140.445	0.383	0.222	7.327	21.812	0.790	0.237	13.780	0.000	5.372	0.000	0.000	0.000	0.000	1.259
0.000	98.891	0.201	0.280	3.990	28.844	3.756	0.066	42.628	0.000	22.876	0.107	0.000	0.000	0.000	0.000
0.000	76.109	0.080	0.093	2.825	27.037	0.951	0.099	41.416	0.000	42.474	0.106	0.000	0.000	0.000	0.000
0.086	99.305	0.074	0.171	1.848	17.811	4.675	0.061	63.508	0.000	52.906	0.049	0.000	0.000	0.314	0.000
0.000	62.928	0.000	0.335	5.555	46.240	3.462	0.119	32.380	0.000	30.009	0.038	0.000	0.000	0.000	0.000
0.000	54.891	0.069	0.636	7.343	39.674	4.080	0.621	29.878	0.258	10.246	0.068	0.000	0.000	0.000	0.000
0.000	52.153	0.000	0.270	6.613	27.857	1.570	0.160	31.556	0.000	10.013	0.000	0.037	0.000	0.000	0.000
0.000	40.481	0.032	1.541	9.231	23.783	3.076	0.214	30.317	0.000	6.543	0.290	0.093	0.000	0.046	0.000
0.000	53.226	0.069	0.280	6.796	32.093	3.051	0.156	31.800	0.000	12.604	0.103	0.000	0.000	0.293	0.000
0.000	59.539	0.000	1.476	11.324	30.284	0.630	0.145	25.853	0.000	4.568	0.152	0.103	0.000	0.038	0.000
0.000	68.590	0.000	0.491	7.904	43.312	4.909	0.151	43.169	0.000	9.662	0.094	0.000	0.000	0.000	0.000
0.000	52.453	0.129	1.436	7.040	46.791	0.440	0.170	21.936	0.000	8.286	0.017	0.000	0.000	0.000	0.000
0.000	94.617	0.101	0.699	6.142	25.914	1.413	0.207	28.581	0.000	7.916	0.183	0.048	0.000	0.000	0.000
0.062	62.858	0.027	1.214	8.795	38.524	1.236	0.597	15.429	0.000	4.248	0.329	0.000	0.000	0.076	0.000
0.064	55.832	0.055	1.831	7.676	51.333	0.893	0.251	23.624	0.000	6.755	0.193	0.000	0.000	0.157	0.000
0.000	87.904	0.000	0.248	11.318	29.790	16.534	0.055	33.603	0.000	10.400	0.053	0.026	0.000	0.000	0.000
0.000	31.712	0.168	0.553	5.355	39.901	0.685	0.092	16.389	0.000	3.695	0.037	0.027	0.034	0.000	0.000
0.063	96.625	0.000	2.916	6.134	34.115	12.899	0.423	57.195	0.000	6.221	0.125	0.052	0.000	0.000	0.178
0.000	139.967	0.031	1.455	8.039	52.837	2.146	0.207	24.888	0.000	5.611	0.104	0.060	0.000	0.000	0.000
0.000	80.087	0.000	0.457	5.405	43.432	0.808	0.162	23.498	0.000	5.229	0.098	0.000	0.013	0.000	0.000
0.000	59.699	0.134	0.466	7.809	37.117	1.341	1.103	20.023	0.000	8.641	0.178	0.128	0.000	0.190	0.000
0.197	93.948	0.170	1.377	6.439	44.817	9.262	0.454	29.739	0.000	8.919	0.084	0.081	0.000	0.000	0.000
0.000	69.787	0.000	1.184	7.094	40.580	0.717	0.068	26.357	0.000	9.784	0.033	0.000	0.013	0.000	0.000
0.000	51.764	0.000	0.723	5.961	34.342	0.913	0.446	23.245	0.000	9.564	0.065	0.000	0.000	0.000	0.000
0.000	112.473	0.043	0.398	2.268	18.253	3.626	0.035	72.303	0.000	38.863	0.028	0.000	0.000	0.000	0.000
0.000	34.583	0.167	1.033	6.492	39.513	6.153	0.550	24.116	0.000	14.401	0.212	0.000	0.000	0.040	0.000
0.000	51.763	0.108	0.250	7.567	35.000	3.939	0.266	32.859	0.000	11.546	0.071	0.000	0.029	0.051	0.000
0.000	52.771	0.082	0.763	6.126	47.543	0.515	0.119	20.516	0.000	10.320	0.232	0.000	0.000	0.058	0.000
0.000	77.795	0.072	0.251	8.207	45.940	0.944	0.119	20.484	0.000	6.671	0.072	0.000	0.000	0.102	0.000
0.000	61.089	0.000	0.115	6.748	68.423	1.982	0.272	31.006	0.000	13.300	0.033	0.000	0.040	0.000	0.000
0.068	137.486	0.059	1.835	7.996	36.019	43.126	0.096	32.528	0.000	11.510	0.000	0.000	0.000	0.042	0.000
0.064	80.273	0.055	0.733	9.354	72.853	0.546	0.057	23.837	0.000	9.647	0.009	0.000	0.000	0.000	0.000
0.000	141.788	0.034	0.479	6.977	55.198	1.288	0.043	28.840	0.000	12.058	0.080	0.000	0.000	0.000	0.000
0.000	75.780	0.033	1.017	2.566	73.671	31.301	0.067	36.128	0.000	9.133	0.000	0.000	0.000	0.000	0.000
0.000	73.854	0.031	0.636	6.054	47.176	1.552	0.100	29.015	0.000	11.941	0.020	0.000	0.012	0.043	0.000
0.000	69.495	0.000	0.877	7.374	42.623	1.377	0.182	32.037	0.000	10.474	0.083	0.000	0.013	0.000	0.000
0.000	59.550	0.000	0.157	9.871	29.441	1.688	0.167	22.743	0.000	8.124	0.112	0.064	0.027	0.000	0.000
0.075	45.634	0.000	1.524	6.952	62.241	2.061	0.000	38.792	0.000	10.538	0.170	0.000	0.000	0.000	0.000
0.079	62.053	0.068	0.197	5.780	56.160	1.863	0.070	32.575	0.000	13.402	0.034	0.000	0.000	0.048	0.000
0.068	40.239	0.176	1.260	8.050	42.599	0.920	0.109	25.370	0.000	6.818	0.126	0.000	0.000	0.167	0.000
0.000	85.941	0.068	0.828	5.529	35.966	5.828	0.070	38.809	0.000	14.140	0.315	0.000	0.014	0.000	0.000
0.000	100.603	0.000	0.479	5.992	26.313	1.658	0.061	31.726	0.000	14.286	0.029	0.000	0.000	0.000	0.000
0.065	59.577	0.000	0.260	9.529	41.996	0.235	0.058	22.258	0.000	7.460	0.000	0.000	0.000	0.000	0.000
0.000	38.864	0.025	0.353	9.193	38.947	1.072	0.063	30.068	0.000	6.568	0.059	0.000	0.000	0.000	0.000
0.000	72.186	0.038	1.374	6.714	34.797	0.765	0.079	29.985	0.000	8.273	0.038	0.000	0.138	0.000	0.000
0.000	48.640	0.033	0.155	5.803	30.900	2.311	0.097	24.507	0.000	10.666	0.089	0.000	0.000	0.048	0.000
0.000	43.941	0.067	0.386	5.121	36.207	2.608	0.082	52.943	0.000	22.755	0.077	0.000	0.013	0.000	0.000
0.000	77.697	0.000	0.760	9.199	37.503	1.745	0.141	24.253	0.000	7.808	0.062	0.000	0.000	0.000	0.000
0.000	56.061	0.030	2.278	8.010	59.591	5.873	0.147	47.763	0.000	11.448	0.138	0.000	0.012	0.127	0.000
0.000	86.236	0.000	0.675	6.246	26.543	6.054	0.200	33.746	0.000	12.514	0.064	0.000	0.000	0.000	0.000
0.000	65.618	0.000	0.744	6.633	30.315	0.846	0.084	26.074	0.000	8.209	0.051	0.049	0.021	0.036	0.000
0.055	63.557	0.000	0.966	9.344	40.930	11.767	0.029	43.428	0.000	8.573	0.008	0.000	0.000	0.000	0.000
0.060	72.811	0.000	0.806	8.485	46.745	1.652	0.127	21.308	0.000	7.529	0.153	0.000	0.041	0.000	0.000
0.057	54.472	0.025	0.426	8.044	47.729	0.984	0.171	26.820	0.000	10.946	0.032	0.000	0.000	0.035	0.000
0.000	94.404	0.154	0.089	4.773	44.234	0.647	0.127	31.329	0.000	10.174	0.051	0.074	0.000	0.438	0.000
0.000	82.318	0.084	0.259	4.690	41.827	1.874	0.057	36.246	0.000	20.876	0.055	0.000	0.000	0.040	0.000
0.000	105.221	0.000	1.785	11.507	66.533	1.328	0.118	18.186	0.000	7.193	0.083	0.068	0.029	0.102	0.000
0.085	77.777	0.146	0.423	5.629	57.619	1.240	0.240	28.862	0.000	9.561	0.024	0.035	0.015	0.000	0.000
0.000	58.880	0.000	0.657	6.999	38.543	0.864	0.233	33.828	0.000	10.156	0.054	0.000	0.000	0.000	0.000
0.000	25.017	0.045	1.266	8.411	33.114	1.720	0.225	30.676	0.000	10.670	0.226	0.000	0.000	0.065	0.000
0.086	82.704	0.296	0.985	11.488	37.761	1.141	0.274	17.247	0.000	5.320	0.514	0.000	0.000	0.052	0.000
0.060	68.465	0.000	1.492	11.469	56.966	1.064	0.064	23.371	0.000	7.459	0.017	0.000	0.000	0.000	0.000
0.061	60.836	0.079	1.193	6.242	41.264	1.635	0.011	29.576	0.000	10.855	0.044	0.000	0.011	0.000	0.000
0.110	95.203	0.000	0.874	7.760	44.221	1.788	0.136	30.506	0.000	7.592	0.000	0.045	0.000	0.000	0.000
0.000	51.468	0.049	0.398	7.070	34.689	1.430	0.040	27.531	0.000	10.108	0.065	0.000	0.000	0.000	0.000
0.000	33.238	0.000	1.195	10.057	70.359	1.301	0.027	21.157	0.000	9.817	0.044	0.000	0.000	0.047	0.000
0.000	81.154	0.149	0.727	8.830	45.832	1.530	0.061	23.561	0.000	6.529	0.049	0.000	0.000	0.000	0.000
0.068	71.284	0.000	0.918	7.512	47.323	0.263	0.145	22.752	0.000	7.369	0.019	0.000	0.000	0.000	0.000

ENSG00000104524	ENSG00000074054	ENSG00000254959	ENSG00000254929	ENSG00000280029	ENSG00000274066	ENSG00000169906	ENSG00000279752	ENSG00000145863	ENSG00000253030	ENSG00000239961	ENSG00000170471	ENSG00000263987	ENSG00000066427	ENSG00000253054	ENSG00000200422
4.654	9.495	0.000	0.122	0.113	0.000	0.000	0.000	0.007	0.000	0.285	5.552	0.000	0.576	0.000	0.000
6.798	4.621	0.000	0.039	0.014	0.301	0.000	0.000	0.045	0.000	0.029	4.786	0.000	0.576	0.000	0.000
11.114	8.302	0.000	0.035	0.025	0.268	0.000	0.000	0.010	0.234	0.078	4.865	0.000	0.770	0.000	0.000
3.728	14.581	0.000	0.065	0.014	0.909	0.000	0.000	0.017	0.000	1.691	4.755	0.000	0.706	0.000	0.000
4.627	4.153	0.000	0.131	0.031	0.337	0.000	0.000	0.000	0.000	4.077	3.462	0.000	0.645	0.000	0.000
4.738	13.376	0.000	0.195	0.061	0.533	0.000	0.000	0.005	0.233	4.811	7.473	0.000	1.311	0.000	0.000
6.500	5.387	0.000	0.103	0.000	0.600	0.000	0.000	0.000	0.788	4.044	5.447	0.000	0.658	0.000	0.000
5.348	5.286	0.000	0.014	0.000	0.000	0.000	0.000	0.030	0.000	2.845	3.528	0.000	0.483	0.370	0.000
6.354	4.039	0.000	0.063	0.000	0.000	0.056	0.000	0.000	0.000	1.703	3.016	0.000	0.472	0.428	0.000
4.215	2.860	0.000	0.046	0.000	0.000	0.000	0.000	0.000	0.000	0.516	2.705	0.000	0.670	0.000	0.000
5.512	5.394	0.000	0.023	0.000	0.262	0.000	0.000	0.005	0.000	2.664	4.196	0.000	0.613	0.612	0.255
5.020	10.513	0.000	0.012	0.025	0.543	0.000	0.000	0.010	0.000	1.007	7.751	0.000	0.782	0.000	0.000
5.870	6.654	0.000	0.048	0.013	0.000	0.000	0.000	0.000	0.000	0.894	5.362	0.000	0.601	0.000	0.000
6.501	8.451	0.000	0.068	0.024	0.524	0.000	0.000	0.015	0.000	4.418	6.806	0.000	0.899	0.000	0.000
7.104	5.812	0.000	0.101	0.053	0.000	0.000	0.000	0.022	0.000	1.619	3.865	0.000	0.493	0.000	0.000
3.005	6.865	0.000	0.068	0.000	0.262	0.040	0.000	0.005	0.229	0.939	7.537	0.000	0.795	0.000	0.000
4.901	9.131	0.000	0.026	0.000	0.302	0.000	0.000	0.000	0.000	0.907	5.043	0.000	0.583	0.000	0.000
6.817	2.335	0.000	0.062	0.016	0.359	0.000	0.000	0.000	0.314	0.648	2.210	0.000	0.441	0.000	0.000
7.363	4.956	0.000	0.012	0.000	0.546	0.000	0.000	0.000	0.000	12.640	3.445	0.000	0.532	0.000	0.000
6.233	8.115	0.000	0.038	0.027	0.292	0.000	0.000	0.000	0.000	0.499	4.104	0.000	0.480	0.341	0.000
11.690	2.118	0.000	0.040	0.000	0.311	0.000	0.000	0.023	0.000	14.506	2.213	0.000	0.371	0.000	0.000
3.743	21.282	0.000	0.097	0.044	0.000	0.000	0.000	0.108	1.690	1.941	8.622	0.000	1.118	0.375	0.000
5.605	5.398	0.000	0.068	0.000	0.314	0.000	0.000	0.012	0.000	5.313	3.453	0.000	0.703	0.367	0.000
8.723	9.005	0.000	0.035	0.000	0.268	0.000	0.000	0.000	0.000	0.336	4.795	0.000	0.297	0.000	0.000
4.692	4.609	0.000	0.000	0.000	0.000	0.000	0.000	0.000	0.000	0.259	4.577	0.290	0.613	0.000	0.000
8.889	8.712	0.000	0.067	0.014	0.000	0.000	0.000	0.000	0.000	0.399	5.606	0.000	0.525	0.000	0.000
7.732	6.172	0.000	0.075	0.000	0.350	0.000	0.000	0.000	0.000	0.203	4.050	0.000	0.486	0.000	0.000
7.293	7.629	0.000	0.027	0.000	1.234	0.000	0.000	0.000	0.540	3.421	3.174	0.000	0.655	0.000	0.000
5.787	4.940	0.007	0.026	0.000	0.307	0.000	0.000	0.000	0.000	0.763	5.193	0.000	1.125	0.000	0.000
6.093	7.455	0.000	0.053	0.000	0.309	0.000	0.000	0.000	0.270	0.717	3.625	0.000	1.093	0.360	0.000
10.764	4.896	0.000	0.120	0.000	0.000	0.039	0.000	0.000	0.000	1.024	5.076	0.000	0.539	0.000	0.000
5.281	10.434	0.000	0.011	0.024	0.000	0.000	0.019	0.005	0.233	0.558	6.293	0.000	1.452	0.000	0.000
7.301	3.860	0.000	0.000	0.000	0.930	0.000	0.000	0.017	0.000	46.236	2.807	0.000	0.638	0.000	0.000
7.172	4.553	0.000	0.013	0.014	0.000	0.000	0.000	0.000	0.000	0.385	3.255	0.000	0.494	0.000	0.000
5.008	7.546	0.000	0.027	0.000	0.631	0.000	0.000	0.000	0.552	2.928	5.581	0.000	0.535	0.000	0.000
5.493	7.234	0.000	0.086	0.079	1.143	0.000	0.000	0.032	0.000	6.043	6.764	0.278	0.644	0.000	0.000
13.730	9.017	0.027	0.047	0.050	0.819	0.042	0.000	0.005	0.239	2.613	6.669	0.000	1.548	0.000	0.000
5.310	6.648	0.000	0.070	0.025	0.272	0.000	0.000	0.000	0.000	2.838	8.359	0.000	0.808	0.000	0.000
5.857	8.526	0.000	0.056	0.015	0.650	0.000	0.000	0.006	0.853	0.681	6.022	0.000	0.829	0.000	0.000
3.522	10.634	0.000	0.072	0.077	0.336	0.000	0.024	0.000	0.588	0.379	6.011	0.000	1.037	0.392	0.000
6.115	11.656	0.016	0.043	0.031	0.334	0.000	0.000	0.006	0.000	2.349	6.469	0.000	0.563	0.000	0.000
6.424	17.511	0.000	0.109	0.026	0.000	0.043	0.000	0.032	0.247	0.428	8.742	0.000	0.717	0.330	0.000
5.593	8.870	0.000	0.067	0.014	0.000	0.000	0.000	0.012	1.088	3.397	7.936	0.000	0.881	0.363	0.000
3.621	9.450	0.000	0.138	0.049	0.267	0.041	0.000	0.010	0.700	9.409	7.121	0.000	0.697	0.000	0.000
4.846	15.555	0.014	0.073	0.052	0.569	0.000	0.000	0.000	0.497	1.677	9.883	0.000	1.254	0.000	0.000
9.189	6.279	0.000	0.096	0.064	0.554	0.000	0.000	0.005	0.243	4.791	5.680	0.000	0.780	0.000	0.000
4.836	10.693	0.000	0.158	0.014	0.611	0.000	0.000	0.000	1.336	0.138	7.229	0.000	1.912	0.000	0.000
4.840	12.419	0.000	0.067	0.028	0.000	0.000	0.000	0.006	0.270	0.926	5.140	0.000	1.120	0.360	0.000
5.845	9.552	0.000	0.035	0.025	0.268	0.000	0.000	0.000	0.234	0.147	6.116	0.000	0.682	0.000	0.000
5.235	3.734	0.000	0.011	0.023	0.000	0.000	0.000	0.000	0.221	0.277	3.803	0.000	0.518	0.295	0.000
6.517	7.548	0.006	0.044	0.000	0.509	0.039	0.000	0.048	0.223	4.241	4.263	0.000	0.479	0.000	0.000
7.202	6.782	0.000	0.118	0.000	0.547	0.000	0.000	0.005	0.239	0.776	3.729	0.000	0.503	0.000	0.000
5.909	4.880	0.007	0.084	0.013	0.557	0.043	0.000	0.005	0.244	0.665	4.952	0.000	0.665	0.000	0.000
5.424	11.442	0.000	0.053	0.056	0.000	0.000	0.000	0.005	0.000	2.032	6.839	0.000	0.590	0.000	0.000
5.802	7.607	0.000	0.293	0.062	0.000	0.000	0.000	0.000	0.397	0.073	5.745	0.000	0.491	0.000	0.000
4.128	17.705	0.000	0.051	0.000	0.000	0.000	0.000	0.000	0.000	0.381	7.505	0.000	0.642	0.000	0.000
6.036	6.996	0.000	0.139	0.000	0.000	0.000	0.000	0.000	0.000	1.836	4.302	0.000	0.697	0.000	0.000
6.063	6.089	0.000	0.036	0.013	0.000	0.000	0.000	0.000	0.000	0.939	6.096	0.000	0.431	0.000	0.000
5.602	8.219	0.000	0.036	0.000	0.551	0.000	0.000	0.000	0.482	2.923	6.326	0.000	0.654	0.322	0.000
8.433	9.380	0.000	0.044	0.047	1.028	0.000	0.000	0.000	0.225	0.224	5.416	0.000	0.339	0.000	0.000
8.009	11.244	0.000	0.154	0.000	0.297	0.000	0.000	0.006	0.000	0.374	6.143	0.000	0.460	0.000	0.000
5.351	7.065	0.000	0.000	0.000	0.000	0.000	0.000	0.000	1.238	0.152	3.555	0.000	0.465	0.550	0.000
4.629	12.555	0.000	0.053	0.011	0.245	0.000	0.000	0.009	0.644	2.200	5.716	0.000	0.752	0.000	0.000
3.743	10.304	0.000	0.023	0.000	0.000	0.000	0.000	0.000	0.230	0.059	5.649	0.000	0.766	0.307	0.000
3.836	9.208	0.000	0.051	0.013	0.587	0.000	0.000	0.000	0.770	0.917	4.945	0.000	0.474	0.000	0.000
7.156	6.860	0.000	0.049	0.035	0.754	0.000	0.000	0.000	0.000	5.468	5.028	0.000	0.528	0.000	0.000
6.860	4.823	0.000	0.067	0.000	0.000	0.000	0.000	0.000	0.000	5.667	3.239	0.000	0.522	0.000	0.000
8.218	3.971	0.000	0.000	0.000	0.000	0.000	0.000	0.000	0.302	0.144	3.039	0.000	0.266	0.000	0.000
5.883	9.641	0.000	0.039	0.000	0.000	0.000	0.000	0.000	0.000	0.561	6.144	0.000	0.469	0.000	0.000
9.005	4.388	0.000	0.031	0.000	0.000	0.000	0.000	0.000	0.000	0.654	3.213	0.000	0.273	0.000	0.000
5.430	7.602	0.000	0.045	0.000	1.055	0.000	0.000	0.005	0.000	0.051	4.555	0.000	0.385	0.000	0.000
9.297	2.297	0.000	0.092	0.000	0.000	0.000	0.000	0.020	0.000	1.083	2.238	0.000	0.381	0.000	0.000
6.034	11.482	0.000	0.042	0.000	0.000	0.000	0.000	0.000	0.000	0.086	4.427	0.000	0.516	0.282	0.000
8.572	8.224	0.000	0.021	0.087	0.000	0.000	0.000	0.000	0.417	2.227	6.711	0.000	0.683	0.000	0.000
3.073	9.444	0.000	0.031	0.017	0.000	0.000	0.000	0.000	0.000	0.305	4.690	0.000	0.677	0.000	0.000
5.016	6.832	0.000	0.000	0.000	0.438	0.000	0.000	0.000	0.000	0.268	4.714	0.000	0.683	0.000	0.000
7.910	2.797	0.000	0.040	0.000	0.612	0.000	0.000	0.000	0.000	10.527	2.711	0.000	0.545	0.000	0.000
3.168	13.251	0.000	0.158	0.000	0.000	0.000	0.000	0.000	0.000	0.178	7.357	0.000	1.335	0.000	0.000
8.069	3.560	0.000	0.066	0.000	0.386	0.000	0.000	0.043	0.675	0.522	4.594	0.000	0.727	0.000	0.000
4.106	3.579	0.009	0.033	0.000	0.384	0.000	0.000	0.000	0.000	0.421	4.414	0.000	0.552	0.000	0.000
4.800	2.233	0.000	0.030	0.000	0.705	0.000	0.000	0.000	0.000	0.170	2.498	0.000	0.423	0.000	0.000
4.861	4.105	0.000	0.071	0.051	0.276	0.000	0.000	0.010	0.000	1.016	5.221	0.000	0.488	0.000	0.000
4.766	7.747	0.000	0.057	0.030	0.000	0.000	0.000	0.012	0.000	1.640	4.117	0.000	0.776	0.000	0.000
4.505	8.414	0.000	0.032	0.051	0.371	0.000	0.000	0.000	0.000	1.964	6.316	0.000	0.605	0.433	0.000
3.737	9.636	0.000	0.027	0.043	0.310	0.000	0.000	0.012	0.000	0.490	5.042	0.000	0.737	0.362	0.000
3.294	6.249	0.000	0.028	0.000	0.330	0.000	0.000	0.000	0.000	0.138	5.591	0.000	0.393	0.000	0.000
5.327	12.343	0.000	0.156	0.024	0.000	0.000	0.000	0.000	0.227	0.878	5.801	0.000	0.697	0.000	0.000
5.673	32.800	0.000	0.047	0.037	0.270	0.000	0.000	0.000	0.237	2.154	4.981	0.000	0.630	0.000	0.000
3.034	14.106	0.000	0.053	0.011	0.494	0.000	0.000	0.000	0.000	0.175	5.239	0.000	0.580	0.000	0.000
4.850	10.169	0.000	0.021	0.022	1.925	0.000	0.000	0.009	0.421	1.862	3.846	0.000	0.741	0.000	0.000
4.092	13.628	0.000	0.044	0.012	1.285	0.000	0.000	0.014	0.674	0.472	5.846	0.000	1.087	0.000	0.000
7.184	7.641	0.000	0.057	0.024	0.000	0.000	0.000	0.000	0.464	0.043	3.198	0.000	0.732	0.309	0.000
8.297	15.286	0.000	0.033	0.012	0.256	0.000	0.000	0.000	0.224	1.246	4.114	0.000	0.433	0.000	0.000
7.157	12.725	0.000	0.058	0.037	0.537	0.000	0.000	0.010	0.235	1.204	9.011	0.000	0.451	0.000	0.000
4.117	11.763	0.000	0.112	0.083	0.518	0.000	0.000	0.015	0.227	0.242	5.131	0.000	0.915	0.302	0.000
7.492	7.752	0.000	0.091	0.041	0.300	0.000	0.000	0.006	0.000	0.242	4.308	0.000	0.980	0.350	0.000
6.070	11.100	0.000	0.014	0.043	0.315	0.000	0.000	0.000	0.551	0.030	5.716	0.000	0.558	0.000	0.000
5.039	11.451	0.000	0.111	0.059	0.000	0.197	0.090	0.000	2.246	0.165	6.062	0.000	0.745	0.000	0.000
7.904	8.385	0.000	0.035	0.037	0.000	0.125	0.000	0.000	0.000	2.095	4.616	0.000	1.161	0.000	0.000
4.871	8.758	0.000	0.041	0.000	0.631	0.049	0.000	0.006	0.276	2.694	4.989	0.000	0.722	0.000	0.000
4.963	9.311	0.008	0.135	0.144	0.314	0.000	0.000	0.006	0.000	0.233	6.399	0.000	0.979	0.000	0.000
9.765	1.650	0.000	0.018	0.000	0.000	0.000	0.000	0.000	0.000	0.661	3.324	0.000	0.481	0.000	0.000
8.726	8.735	0.000	0.034	0.000	0.533	0.000	0.000	0.000	0.000	0.017	4.233	0.000	0.878	0.622	0.000
6.569	7.911	0.008	0.059	0.000	0.344	0.000	0.000	0.006	0.000	0.410	6.154	0.000	0.446	0.000	0.000
6.886	9.413	0.000	0.034	0.018	0.788	0.000	0.000	0.000	0.000	0.127	7.430	0.000	0.715	0.460	0.000
4.991	7.373	0.000	0.030	0.000	0.690	0.000	0.000	0.000	0.000	1.447	4.472	0.000	0.679	0.000	0.000
9.909	10.397	0.000	0.027	0.000	0.316	0.000	0.000	0.024	0.000	0.153	7.049	0.000	0.386	0.000	0.000
14.352	7.711	0.000	0.109	0.013	0.000	0.000	0.000	0.000	0.491	0.733	4.707	0.000	0.742	0.000	0.000
6.472	8.950	0.000	0.125	0.000	0.263	0.000	0.000	0.000	0.000	0.382	7.567	0.000	0.402	0.000	0.000
9.953	5.391	0.000	0.028	0.000	0.000	0.000	0.000	0.019	0.000	0.234	2.881	0.000	0.458	0.000	0.000
9.157	2.711	0.000	0.040	0.000	0.311	0.000	0.000	0.000	0.000	3.762	3.335	0.000	0.329	0.000	0.000
5.191	10.454	0.000	0.025	0.000	0.876	0.000	0.000	0.005	0.255	0.122	6.978	0.000	0.621	0.341	0.000
5.357	6.884	0.000	0.052	0.041	0.603	0.000	0.000	0.011	0.000	17.314	4.533	0.000	0.651	0.000	0.000
4.532	9.008	0.000	0.056	0.089	1.292	0.099	0.000	0.000	0.565	1.709	5.106	0.000	0.632	0.000	0.000
5.358	9.246	0.000	0.053	0.000	0.614	0.000	0.000	0.011	0.000	0.040	7.620	0.000	0.386	0.358	0.000
6.998	7.897	0.000	0.042	0.015	0.326	0.000	0.000	0.000	0.000	0.547	5.471	0.000	0.509	0.000	0.000
3.434	15.885	0.000	0.048	0.013	0.281	0.043	0.000	0.032	0.000	3.110	5.688	0.000	0.770	0.328	0.000
5.817	7.221	0.008	0.056	0.030	0.651	0.000	0.000	0.012	0.000	1.081	6.369	0.000	0.482	0.000	0.000
6.876	7.665	0.000	0.073	0.026	0.565	0.000	0.000	0.016	0.000	1.749	5.042	0.000	0.599	0.000	0.000
6.093	10.633	0.007	0.069	0.012	0.000	0.000	0.000	0.000	0.235	0.052	5.959	0.000	0.528	0.000	0.000
10.190	9.460	0.006	0.010	0.022	0.000	0.000	0.000	0.000	0.213	0.737	6.434	0.000	0.411	0.000	0.000
3.372	7.679	0.000	0.016	0.017	0.000	0.000	0.000	0.007	0.000	0.425	3.894	0.000	0.405	0.000	0.000
8.150	5.950	0.016	0.028	0.015	0.962	0.000	0.000	0.012	0.561	0.031	5.682	0.000	0.450	0.000	0.000
11.988	4.452	0.000	0.027	0.000	0.318	0.000	0.000	0.000	0.000	1.704	3.686	0.000	0.337	0.000	0.000
5.481	9.377	0.007	0.026	0.027	0.299	0.000	0.000	0.000	0.000	0.463	5.633	0.000	0.740	0.349	0.000
5.104	6.964	0.000	0.037	0.000	0.570	0.000	0.000	0.091	0.249	2.987	4.411	0.000	0.542	0.000	0.000
6.095	6.932	0.000	0.000	0.000	2.787	0.000	0.000	0.000	0.000	3.056	3.498	0.000	0.902	0.000	0.000
6.498	7.200	0.006	0.053	0.023	0.491	0.000	0.000	0.014	0.430	0.531	4.334	0.239	0.577	0.000	0.000
7.585	6.796	0.000	0.049	0.021	0.456	0.000	0.000	0.000	0.000	0.632	5.883	0.000	0.344	0.000	0.000
6.974	9.868	0.000	0.117	0.034	1.233	0.000	0.000	0.000	0.431	0.151	8.165	0.000	0.958	0.000	0.000
5.618	11.800	0.000	0.020	0.021	0.469	0.000	0.000	0.004	0.000	0.113	6.296	0.000	0.690	0.274	0.000
7.077	4.922	0.000	0.032	0.000	0.000	0.000	0.000	0.000	0.000	0.048	3.236	0.000	0.422	0.000	0.000
2.920	4.359	0.000	0.034	0.000	0.000	0.000	0.000	0.000	0.000	15.738	5.074	0.000	0.496	0.000	0.000
7.190	8.459	0.008	0.059	0.047	0.686	0.000	0.000	0.000	0.000	0.884	6.132	0.000	0.620	0.000	0.000
5.622	6.185	0.000	0.000	0.000	0.698	0.000	0.000	0.000	0.306	2.027	4.911	0.000	0.618	0.000	0.000
4.208	7.073	0.000	0.050	0.000	0.000	0.000	0.000	0.000	0.000	0.238	4.362	0.000	0.834	0.000	0.000
9.078	11.132	0.000	0.038	0.000	0.435	0.067	0.000	0.024	0.381	0.365	5.315	0.000	0.623	0.508	0.000
7.379	11.786	0.000	0.091	0.016	0.707	0.000	0.000	0.026	0.309	0.935	7.406	0.000	0.658	0.000	0.000
7.380	8.407	0.000	0.064	0.056	0.000	0.000	0.000	0.000	0.216	0.175	8.209	0.000	0.761	0.287	0.000
7.742	8.182	0.006	0.022	0.012	0.000	0.000	0.000	0.028	0.000	0.725	5.039	0.000	0.623	0.000	0.000
6.247	7.229	0.000	0.097	0.000	0.000	0.000	0.000	0.000	0.789	0.523	4.683	0.000	0.487	0.000	0.000
6.288	11.397	0.006	0.040	0.011	0.705	0.000	0.000	0.004	0.000	0.212	4.488	0.000	0.514	0.000	0.000
14.401	11.310	0.000	0.055	0.029	1.273	0.000	0.000	0.000	0.000	5.521	6.727	0.000	0.386	0.000	0.000
7.215	10.672	0.000	0.025	0.052	0.286	0.000	0.000	0.016	0.000	1.393	6.128	0.000	0.719	0.000	0.000
4.901	8.517	0.000	0.000	0.013	0.000	0.000	0.000	0.000	0.000	0.317	4.202	0.000	0.417	0.000	0.000

ENSG00000257075	ENSG00000227023	ENSG00000168333	ENSG00000262141	ENSG00000267323	ENSG00000163435	ENSG00000198356	ENSG00000237679	ENSG00000240731	ENSG00000229782	ENSG00000235594	ENSG00000238367	ENSG00000002330	ENSG00000236165	ENSG00000258672	ENSG00000199121
0.000	0.000	0.397	0.158	0.063	0.089	64.391	0.000	4.809	0.067	0.000	0.000	29.575	0.283	0.000	0.675
0.000	0.000	0.031	0.670	0.027	0.228	65.942	0.074	3.107	0.000	0.000	0.000	24.455	0.240	0.000	0.956
0.000	0.000	0.891	0.596	0.167	0.086	85.714	0.022	5.968	0.152	0.000	0.000	30.376	0.250	0.000	0.170
0.000	0.000	0.124	0.135	0.054	0.124	129.876	0.000	9.125	0.000	0.000	0.000	28.100	0.283	0.000	0.193
0.000	0.000	0.023	0.150	0.151	0.492	86.275	0.028	5.258	0.000	0.000	0.000	42.366	0.315	0.000	0.215
0.000	0.000	0.018	0.238	0.119	0.109	56.374	0.044	16.912	0.202	0.000	0.000	20.290	0.497	0.038	0.678
0.000	0.000	0.010	0.401	0.241	0.073	55.669	0.025	11.099	0.057	0.000	0.000	19.612	0.360	0.000	0.573
0.000	0.000	0.097	0.424	0.085	0.192	77.026	0.026	2.014	0.060	0.000	0.000	31.384	0.339	0.000	0.202
0.000	0.000	1.147	0.491	0.033	0.196	114.847	0.000	4.988	0.278	0.000	0.000	32.929	0.538	0.000	0.000
0.000	0.000	0.073	1.428	0.191	0.094	89.512	0.000	4.065	0.000	0.000	0.000	21.480	0.996	0.000	0.000
0.000	0.000	0.036	0.351	0.070	0.163	69.937	0.065	4.566	0.099	0.000	0.000	26.367	0.315	0.037	0.334
0.000	0.000	0.009	0.605	0.146	0.145	83.114	0.067	8.074	0.103	0.000	0.000	20.551	0.253	0.000	0.864
0.000	0.000	0.467	0.375	0.200	0.319	70.212	0.000	6.553	0.053	0.000	0.000	25.602	0.374	0.040	0.178
0.000	0.000	0.187	0.117	0.422	0.390	88.910	0.000	10.405	0.297	0.000	0.000	27.981	0.559	0.000	1.167
0.000	0.000	0.754	0.521	0.000	1.818	82.417	0.000	3.176	0.000	0.000	0.000	44.594	0.623	0.000	0.743
0.000	0.000	0.036	0.000	0.094	0.462	42.794	0.065	2.996	0.000	0.000	0.000	15.348	0.175	0.000	0.334
0.034	0.000	0.298	0.135	0.108	0.109	118.229	0.025	5.539	0.057	0.000	0.000	25.310	0.081	0.000	0.192
0.000	0.000	0.012	0.640	0.193	0.087	277.978	0.029	1.888	0.000	0.000	0.000	58.355	1.675	0.000	0.228
0.000	0.000	0.046	0.609	0.147	0.043	112.299	0.000	5.400	0.103	0.000	0.000	28.417	0.328	0.000	0.522
0.000	0.000	0.119	0.260	0.026	0.293	72.655	0.024	7.839	0.055	0.000	0.000	28.739	0.584	0.000	0.186
0.000	0.000	4.731	0.694	0.362	0.036	191.677	0.026	2.371	0.118	0.000	0.000	54.741	1.204	0.000	0.198
0.000	0.000	0.000	0.431	0.230	0.047	55.670	0.026	13.308	0.122	0.000	0.000	21.092	0.215	0.000	0.205
0.000	0.000	0.032	0.421	0.112	0.080	107.374	0.052	7.585	0.238	0.000	0.000	24.552	0.671	0.000	0.200
0.000	0.000	0.009	0.000	0.072	0.029	70.626	0.000	2.377	0.000	0.000	0.000	32.478	0.642	0.000	0.681
0.000	0.000	0.192	1.062	0.107	0.736	85.521	0.000	5.240	0.000	0.000	0.000	25.184	0.079	0.000	0.000
0.000	0.000	0.221	0.138	0.083	0.606	78.991	0.000	9.773	0.234	0.000	0.000	31.061	0.578	0.088	0.394
0.000	0.000	0.036	0.781	0.063	0.126	91.341	0.058	8.449	0.066	0.000	0.000	34.253	0.981	0.000	0.000
0.000	0.000	0.189	1.100	0.165	0.108	79.529	0.000	9.619	0.583	0.000	0.000	31.623	0.082	0.000	0.000
0.000	0.000	0.073	0.411	0.082	0.260	123.509	0.025	15.208	0.058	0.000	0.000	49.627	0.614	0.000	0.586
0.000	0.000	0.032	0.551	0.304	0.135	171.668	0.000	15.631	0.175	0.000	0.000	53.874	0.659	0.044	1.180
0.000	0.000	0.199	0.113	0.091	0.040	122.840	0.042	3.779	0.096	0.000	0.000	53.449	0.745	0.036	0.000
0.000	0.000	0.344	0.831	0.143	0.200	94.008	0.022	7.824	0.151	0.000	0.000	24.131	0.710	0.038	0.847
0.000	0.000	0.011	0.138	0.139	0.115	107.883	0.000	9.384	0.176	0.000	0.000	56.243	0.248	0.000	0.394
0.000	0.000	0.193	0.399	0.053	0.413	82.463	0.025	8.773	0.000	0.000	0.000	44.916	0.119	0.000	0.190
0.071	0.000	0.322	0.844	0.113	0.749	66.863	0.000	8.807	0.239	0.000	0.000	26.959	0.168	0.000	0.000
0.000	0.000	0.058	0.000	0.077	0.195	59.593	0.023	7.564	0.000	0.000	0.000	35.944	0.267	0.041	0.000
0.000	0.000	0.928	0.000	0.341	0.098	53.656	0.045	21.128	0.103	0.000	0.000	27.027	0.764	0.039	4.341
0.000	0.000	0.083	0.364	0.292	0.167	73.861	0.000	20.162	0.103	0.000	0.000	21.018	0.400	0.039	1.561
0.000	0.000	1.636	0.580	0.145	0.076	86.365	0.000	9.196	0.431	0.000	0.000	27.831	0.390	0.046	0.000
0.000	0.000	0.651	0.150	0.120	0.065	72.677	0.000	14.678	0.127	0.000	0.000	17.962	0.134	0.000	1.069
0.000	0.000	0.023	0.298	0.090	0.075	64.144	0.055	9.514	0.126	0.000	0.000	30.089	0.223	0.047	1.276
0.000	0.000	0.019	0.000	0.101	0.113	60.556	0.000	7.376	0.107	0.000	0.000	23.729	0.113	0.000	0.539
0.000	0.000	0.000	0.139	0.194	0.073	136.408	0.026	8.792	0.000	0.000	0.000	30.965	0.373	0.000	1.187
0.000	0.000	0.018	0.000	0.119	0.060	56.753	0.000	12.045	0.000	0.000	0.000	23.833	0.285	0.038	0.509
0.000	0.000	0.116	0.253	0.229	0.094	102.983	0.023	17.269	0.000	0.000	0.000	23.090	0.227	0.161	0.724
0.000	0.000	0.085	0.618	0.149	0.912	77.558	0.000	9.754	0.000	0.000	0.000	37.519	0.407	0.039	0.353
0.000	0.000	0.021	0.408	0.082	0.080	68.293	0.050	17.164	0.116	0.000	0.000	34.735	0.692	0.000	0.971
0.000	0.000	0.137	0.827	0.138	0.078	76.274	0.025	13.840	0.234	0.000	0.000	22.996	0.577	0.000	0.786
0.000	0.000	0.173	0.597	0.024	0.485	73.123	0.088	8.940	0.000	0.000	0.000	34.632	0.322	0.000	0.341
0.000	0.000	0.009	0.338	0.045	0.909	59.721	0.042	2.707	0.096	0.000	0.000	27.000	0.708	0.000	0.000
0.000	0.000	0.121	0.681	0.136	0.074	97.156	0.000	6.367	0.433	0.000	0.000	40.228	0.339	0.000	0.486
0.000	0.000	0.009	0.488	0.098	0.032	74.349	0.045	3.026	0.207	0.000	0.000	28.601	0.511	0.000	0.870
0.000	0.000	0.095	0.124	0.025	0.358	109.167	0.023	3.486	0.105	0.000	0.000	33.643	0.409	0.000	0.355
0.000	0.000	0.042	0.549	0.198	0.125	105.894	0.000	5.804	0.000	0.000	0.000	29.765	0.394	0.000	0.157
0.000	0.000	1.279	0.202	0.243	0.057	163.354	0.000	6.824	0.000	0.000	0.000	35.286	0.544	0.000	0.289
0.000	0.000	0.683	0.527	0.211	0.080	82.968	0.097	11.565	0.000	0.000	0.000	25.894	0.315	0.000	0.000
0.000	0.000	0.190	0.393	0.210	0.114	74.515	0.048	9.528	0.056	0.000	0.000	40.228	0.352	0.042	0.000
0.000	0.000	0.311	0.495	0.074	0.097	81.385	0.068	10.311	0.052	0.000	0.000	19.733	0.814	0.000	0.000
0.000	0.000	0.225	0.246	0.123	0.035	73.215	0.023	5.947	0.104	0.000	0.000	23.582	0.257	0.000	0.175
0.000	0.000	0.201	0.229	0.115	0.215	85.148	0.021	1.725	0.000	0.000	0.000	34.582	0.480	0.037	0.000
0.000	0.000	3.172	0.397	0.133	0.104	83.253	0.098	9.915	0.225	0.000	0.000	36.292	0.475	0.000	0.567
0.000	0.000	0.048	0.841	0.295	0.064	123.319	0.000	7.866	0.000	0.000	0.000	35.630	0.251	0.000	0.300
0.000	0.000	0.876	0.219	0.197	0.088	68.964	0.020	13.042	0.186	0.000	0.000	29.222	0.458	0.035	0.156
0.000	0.000	0.805	0.117	0.165	0.290	53.096	0.022	14.939	0.199	0.000	0.000	22.458	0.491	0.000	1.172
0.000	0.000	8.657	0.785	0.052	0.080	98.087	0.024	5.479	0.055	0.000	0.000	39.663	0.430	0.000	0.373
0.000	0.000	28.790	0.336	0.135	0.062	120.742	0.000	4.101	0.071	0.000	0.000	45.981	0.201	0.000	0.240
0.000	0.000	0.123	0.922	0.323	0.050	221.668	0.085	5.531	0.098	0.000	0.000	66.820	0.414	0.000	0.329
0.000	0.000	0.258	0.768	0.246	0.040	216.293	0.000	2.563	0.065	0.000	0.000	65.567	0.505	0.000	0.219
0.000	0.000	0.031	1.021	0.123	0.058	107.174	0.038	6.481	0.000	0.000	0.000	34.460	0.244	0.000	0.000
0.000	0.000	0.025	0.968	0.162	0.004	187.335	0.000	2.100	0.000	0.000	0.000	102.011	0.627	0.000	0.000
0.000	0.000	0.045	0.588	0.189	0.108	94.777	0.043	2.153	0.349	0.000	0.000	39.699	0.598	0.000	0.000
0.000	0.000	0.689	0.475	0.048	0.997	196.234	0.000	0.967	0.303	0.000	0.000	61.169	0.213	0.076	0.000
0.000	0.000	0.082	0.431	0.065	0.209	51.660	0.020	4.778	0.046	0.000	0.000	26.677	0.193	0.034	0.154
0.000	0.000	0.113	0.000	0.298	0.134	83.132	0.000	4.492	0.090	0.000	0.000	43.297	0.318	0.000	0.000
0.000	0.000	0.037	0.486	0.195	0.255	63.502	0.030	3.360	0.000	0.000	0.000	22.975	0.388	0.000	0.925
0.000	0.000	0.030	0.390	0.039	0.060	125.363	0.036	12.067	0.000	0.000	0.000	40.764	0.117	0.186	0.000
0.034	0.000	0.083	0.682	0.191	0.494	225.788	0.025	3.607	0.058	0.000	0.000	41.911	0.571	0.043	0.974
0.000	0.000	0.000	0.818	0.164	0.107	59.177	0.000	9.651	0.000	0.000	0.000	14.742	0.245	0.000	0.000
0.000	0.000	3.237	0.859	0.138	0.176	242.540	0.000	2.796	0.219	0.000	0.000	52.817	0.463	0.000	0.000
0.000	0.000	0.078	1.028	0.000	1.545	97.161	0.000	2.300	0.145	0.000	0.000	34.487	0.564	0.000	0.245
0.000	0.000	2.265	1.257	0.536	0.384	187.218	0.000	0.959	0.133	0.000	0.000	48.037	0.376	0.000	0.000
0.000	0.000	0.085	0.493	0.074	0.624	116.795	0.000	1.654	0.052	0.000	0.000	49.115	0.258	0.000	0.352
0.000	0.000	0.022	0.146	0.029	0.134	58.659	0.000	9.702	0.186	0.000	0.000	24.763	0.175	0.000	0.627
0.000	0.000	0.038	0.166	0.066	0.098	64.837	0.000	6.669	0.000	0.000	0.000	22.609	0.644	0.000	0.946
0.000	0.000	11.342	0.138	0.055	0.069	43.620	0.000	5.515	0.059	0.000	0.000	19.867	0.166	0.044	0.000
0.000	0.000	0.067	0.147	0.088	0.228	40.994	0.081	5.741	0.062	0.000	0.000	25.771	0.176	0.000	0.210
0.029	0.000	0.644	0.347	0.209	0.056	71.315	0.107	18.670	0.147	0.000	0.000	18.546	0.553	0.110	0.165
0.000	0.000	0.313	0.121	0.169	0.076	82.238	0.067	12.411	0.102	0.000	0.000	34.776	0.469	0.000	0.344
0.000	0.000	0.185	0.110	0.044	0.036	62.366	0.041	5.646	0.000	0.000	0.000	25.254	0.428	0.000	0.157
0.000	0.000	0.311	0.215	0.258	0.117	73.703	0.040	5.848	0.000	0.000	0.000	28.917	0.193	0.000	0.613
0.000	0.000	0.035	0.458	0.138	0.150	60.514	0.042	20.037	0.389	0.000	0.000	27.422	0.171	0.036	0.818
0.000	0.000	7.332	0.237	0.119	0.274	66.870	0.000	14.721	0.000	0.000	0.000	37.628	0.212	0.038	0.675
0.000	0.000	0.009	0.228	0.000	0.020	123.536	0.000	4.503	0.048	0.000	0.000	38.692	0.341	0.000	0.163
0.030	0.000	0.000	0.359	0.192	0.078	51.210	0.066	11.105	0.000	0.000	0.000	13.296	0.322	0.000	0.342
0.000	0.000	0.018	0.346	0.139	0.076	52.758	0.021	14.649	0.098	0.000	0.000	26.771	0.380	0.037	0.989
0.000	0.000	0.408	0.268	0.161	0.038	67.909	0.049	12.691	0.000	0.000	0.000	37.470	0.200	0.000	0.765
0.000	0.000	0.064	0.842	0.309	0.031	95.217	0.000	8.275	0.000	0.000	0.000	48.775	0.378	0.000	0.601
0.000	0.000	2.792	0.572	0.000	0.162	58.450	0.105	18.151	0.486	0.000	0.000	30.817	0.684	0.000	0.817
0.000	0.000	0.635	0.000	0.000	0.229	70.243	0.133	17.086	0.307	0.000	0.000	28.727	0.217	0.000	0.517
0.000	0.000	0.171	0.281	0.028	0.279	83.363	0.000	7.490	0.298	0.000	0.000	28.324	0.378	0.000	0.201
0.000	0.000	0.182	0.000	0.056	0.128	51.533	0.000	10.370	0.119	0.000	0.000	19.173	0.545	0.000	0.600
0.000	0.000	0.042	1.098	0.220	0.100	156.501	0.000	1.563	0.078	0.000	0.000	47.217	0.930	0.000	0.000
0.000	0.000	0.335	0.238	0.167	2.143	67.554	0.044	10.151	0.101	0.000	0.000	47.773	0.142	0.000	0.509
0.115	0.000	0.035	0.153	0.092	0.077	65.153	0.028	7.976	0.000	0.000	0.000	33.895	0.367	0.000	0.437
0.000	0.000	0.080	0.176	0.070	0.065	66.362	0.032	15.576	0.224	0.000	0.000	36.122	0.315	0.000	1.003
0.000	0.000	0.070	0.000	0.123	0.060	67.287	0.113	11.143	0.000	0.000	0.000	32.767	0.184	0.000	0.439
0.000	0.000	0.150	0.000	0.028	0.129	51.693	0.000	5.846	0.000	0.000	0.000	42.887	0.126	0.000	0.000
0.000	0.000	0.610	0.000	0.351	0.046	125.520	0.023	2.798	0.106	0.000	0.000	48.596	0.262	0.000	0.179
0.000	0.000	0.027	0.235	0.165	0.031	84.266	0.000	9.360	0.000	0.000	0.000	38.755	0.281	0.037	0.000
0.000	0.000	0.056	0.588	0.118	0.700	159.260	0.027	2.752	0.000	0.000	0.000	69.407	0.924	0.000	0.210
0.000	0.000	0.032	0.416	0.167	0.021	131.878	0.000	1.072	0.000	0.000	0.000	66.531	0.539	0.000	0.198
0.000	0.000	0.069	0.000	0.261	0.026	124.522	0.024	6.460	0.110	0.000	0.000	28.456	0.273	0.000	0.000
0.000	0.000	0.000	0.269	0.054	0.056	81.656	0.025	9.902	0.114	0.000	0.000	30.167	0.040	0.000	0.192
0.000	0.000	0.154	0.864	0.116	0.069	81.350	0.159	10.547	0.000	0.000	0.000	25.874	0.517	0.000	0.822
0.034	0.000	0.240	0.137	0.137	0.105	95.117	0.025	5.622	0.000	0.000	0.000	40.822	0.205	0.000	0.000
0.000	0.000	0.044	0.872	0.175	0.340	108.105	0.027	3.606	0.062	0.000	0.000	37.645	0.304	0.000	0.000
0.000	0.021	0.038	0.501	0.176	0.099	85.294	0.023	8.158	0.053	0.000	0.000	26.239	0.412	0.000	0.716
0.000	0.000	0.011	0.435	0.116	0.139	72.270	0.000	4.664	0.062	0.000	0.000	18.973	0.477	0.046	0.000
0.000	0.000	0.010	0.252	0.126	0.025	116.882	0.070	3.791	0.000	0.000	0.000	30.841	0.188	0.000	0.360
0.000	0.000	0.064	0.599	0.096	0.149	91.603	0.022	7.163	0.000	0.000	0.000	24.138	0.215	0.038	0.513
0.000	0.000	0.380	0.108	0.109	0.054	68.312	0.040	6.259	0.138	0.000	0.000	23.857	0.324	0.000	0.000
0.000	0.000	4.790	0.163	0.131	0.057	75.518	0.090	5.110	0.069	0.000	0.000	27.672	0.439	0.000	0.466
0.000	0.000	0.022	0.286	0.172	0.081	54.589	0.000	1.803	0.000	0.000	0.000	16.807	0.342	0.000	0.816
0.000	0.000	0.022	0.284	0.000	0.236	128.780	0.000	2.887	0.000	0.000	0.000	59.356	1.401	0.000	0.203
0.000	0.000	0.173	0.000	0.187	0.081	102.344	0.000	14.037	0.057	0.000	0.000	32.604	0.239	0.042	0.761
0.000	0.022	0.252	0.508	0.127	0.053	67.965	0.023	4.186	0.054	0.000	0.000	27.916	0.304	0.000	0.181
0.000	0.000	1.674	0.829	0.166	0.127	112.951	0.076	8.088	0.000	0.000	0.000	31.453	0.372	0.000	0.000
0.000	0.000	0.568	0.438	0.198	0.308	78.208	0.020	12.517	0.093	0.000	0.000	26.054	0.360	0.000	0.313
0.000	0.000	3.780	0.203	0.204	0.038	98.935	0.037	4.792	0.000	0.015	0.000	37.748	0.759	0.000	0.290
0.000	0.000	1.550	0.440	0.110	0.158	73.288	0.041	7.958	0.140	0.000	0.000	34.331	0.296	0.035	0.000
0.000	0.000	0.096	0.209	0.147	0.135	101.307	0.077	6.721	0.044	0.000	0.000	27.484	0.219	0.000	0.448
0.000	0.000	0.276	0.329	0.132	0.086	92.119	0.000	2.545	0.000	0.000	0.000	44.807	0.098	0.000	0.000
0.000	0.000	0.835	1.071	0.286	0.016	148.263	0.044	5.372	0.050	0.000	0.000	37.734	0.605	0.000	0.170
0.000	0.026	2.133	0.306	0.306	0.113	78.789	0.056	16.537	0.649	0.000	0.000	39.156	0.503	0.000	0.436
0.000	0.000	0.095	0.311	0.219	0.411	127.913	0.000	8.360	0.066	0.000	0.000	50.939	0.419	0.000	0.889
0.000	0.000	0.290	0.519	0.069	0.121	90.478	0.064	7.172	0.000	0.000	0.000	30.002	0.879	0.055	0.740
0.000	0.000	0.059	0.000	0.039	0.119	63.940	0.143	8.292	0.165	0.000	0.000	32.216	0.348	0.000	0.000
0.000	0.000	2.561	0.158	0.158	0.210	77.816	0.029	11.993	0.134	0.000	0.000	25.958	0.519	0.000	0.225
0.000	0.000	1.223	0.110	0.022	0.067	85.321	0.040	11.082	0.093	0.000	0.000	31.769	0.526	0.035	0.157
0.000	0.000	0.094	0.000	0.113	0.084	75.134	0.062	10.905	0.096	0.000	0.000	32.587	0.303	0.000	0.322
0.000	0.000	0.015	0.201	0.081	0.119	102.050	0.000	4.906	0.000	0.000	0.347	31.565	0.180	0.000	0.287
0.000	0.000	0.040	0.105	0.126	0.037	62.533	0.039	4.475	0.000	0.000	0.000	28.094	0.627	0.000	0.449
0.000	0.000	4.155	0.000	0.057	0.375	52.203	0.078	6.406	0.060	0.000	0.000	39.287	0.255	0.000	0.405
0.000	0.000	0.185	0.893	0.153	0.206	90.206	0.023	18.051	0.108	0.000	0.000	39.363	0.572	0.041	0.728
0.000	0.000	0.477	0.125	0.025	0.046	91.336	0.023	7.994	0.000	0.000	0.000	33.302	0.262	0.000	0.357

ENSG00000199874	ENSG00000115307	ENSG00000053747	ENSG00000202231	ENSG00000267794	ENSG00000229917	ENSG00000131943	ENSG00000264994	ENSG00000203620	ENSG00000204120	ENSG00000276770	ENSG00000249045	ENSG00000251443	ENSG00000118017	ENSG00000217060	ENSG00000278961
0.208	35.865	0.082	0.000	0.000	0.134	6.958	0.000	0.099	4.813	0.000	0.000	0.048	0.056	0.171	0.000
0.000	73.888	0.903	0.000	0.000	0.483	6.385	0.000	0.000	3.145	0.000	0.000	0.000	0.036	0.106	0.000
0.157	57.726	0.044	0.000	0.000	0.658	8.867	0.000	0.000	4.442	0.000	0.000	0.000	0.021	0.024	0.000
0.000	50.934	0.053	0.000	0.000	0.029	8.849	0.250	0.019	4.827	0.000	0.000	0.000	0.012	0.067	0.000
0.000	60.526	0.161	0.177	0.000	0.064	5.942	0.000	0.000	3.656	0.000	0.000	0.000	0.000	0.030	0.000
0.157	50.815	0.097	0.000	0.000	0.151	6.652	0.000	0.000	6.993	0.000	0.000	0.000	0.032	0.094	0.000
0.176	51.929	0.791	0.000	0.000	0.114	6.268	0.247	0.000	3.705	0.000	0.000	0.000	0.047	0.026	0.000
0.187	44.084	0.115	0.000	0.000	0.030	7.171	0.000	0.010	3.307	0.000	0.000	0.000	0.025	0.042	0.000
0.216	64.818	0.097	0.000	0.000	0.035	6.530	0.000	0.000	2.235	0.000	0.000	0.000	0.014	0.032	0.024
0.000	69.984	0.133	0.000	0.000	0.101	5.405	0.000	0.000	2.595	0.000	0.000	0.000	0.000	0.141	0.000
0.000	63.150	0.151	0.138	0.000	0.000	6.606	0.000	0.081	1.073	0.000	0.000	0.000	0.000	0.058	0.000
0.000	52.734	0.094	0.000	0.000	0.026	10.693	0.000	0.008	5.632	0.000	0.000	0.000	0.021	0.060	0.000
0.000	45.033	0.038	0.000	0.000	0.079	8.069	0.000	0.026	3.854	0.000	0.000	0.000	0.033	0.062	0.019
0.000	48.014	0.209	0.000	0.000	0.050	9.238	0.000	0.024	5.393	0.000	0.000	0.000	0.031	0.104	0.000
0.000	53.118	0.157	0.000	0.000	0.000	5.729	0.000	0.000	4.008	0.000	0.000	0.000	0.000	0.154	0.000
0.000	36.311	0.361	0.000	0.000	0.050	6.274	0.000	0.008	6.374	0.000	0.000	0.000	0.249	0.081	0.000
0.000	48.585	0.050	0.000	0.000	0.000	8.672	0.000	0.009	4.022	0.000	0.000	0.000	0.012	0.067	0.000
0.000	122.373	0.032	0.000	0.000	0.034	9.528	0.000	0.000	1.980	0.318	0.000	0.000	0.028	0.047	0.000
0.161	63.876	0.328	0.000	0.177	0.620	6.009	0.000	0.008	2.944	0.000	0.000	0.000	0.011	0.048	0.000
0.000	45.219	0.115	0.000	0.000	0.083	6.774	0.000	0.000	4.768	0.000	0.000	0.040	0.012	0.064	0.000
0.000	90.495	0.058	0.000	0.000	0.029	8.810	0.000	0.251	2.110	0.000	0.000	0.000	0.025	0.027	0.000
0.000	29.740	0.193	0.000	0.000	0.213	10.465	0.000	0.020	5.973	0.285	0.000	0.000	0.064	0.057	0.000
0.185	57.114	0.111	0.000	0.000	0.089	6.328	0.000	0.088	2.768	0.000	0.000	0.000	0.062	0.042	0.042
0.000	67.446	0.053	0.000	0.000	0.051	6.153	0.000	0.025	4.336	0.000	0.000	0.000	0.000	0.035	0.000
0.000	55.862	0.105	0.000	0.000	0.535	8.397	0.000	0.009	3.700	0.000	0.000	0.000	0.024	0.066	0.000
0.000	44.974	0.173	0.000	0.000	0.029	7.684	0.000	0.019	5.184	0.000	0.000	0.000	0.073	0.068	0.000
0.000	58.488	0.296	0.000	0.000	0.033	5.220	0.000	0.000	3.606	0.310	0.000	0.000	0.014	0.046	0.000
0.000	57.599	0.073	0.000	0.000	0.058	7.506	0.000	0.019	3.089	0.000	0.000	0.000	0.000	0.054	0.000
0.181	67.152	0.078	0.000	0.000	0.029	9.972	0.000	0.029	3.247	0.000	0.000	0.000	0.012	0.041	0.000
0.363	86.666	0.042	0.000	0.000	0.029	10.539	0.000	0.000	3.773	0.000	0.000	0.042	0.012	0.014	0.000
0.000	99.776	0.121	0.000	0.000	0.024	9.046	0.000	0.008	3.241	0.000	0.000	0.000	0.070	0.011	0.017
0.000	66.030	0.291	0.000	0.000	0.025	7.868	0.000	0.000	5.255	0.000	0.000	0.000	0.042	0.082	0.000
0.000	68.247	0.066	0.000	0.000	0.000	8.211	0.000	0.067	3.041	0.000	0.000	0.000	0.024	0.000	0.000
0.000	69.838	0.071	0.000	0.000	0.028	8.135	0.000	0.000	3.126	0.000	0.000	0.000	0.024	0.026	0.000
0.000	48.405	0.291	0.000	0.000	0.060	7.938	0.000	0.039	5.367	0.000	0.000	0.000	0.025	0.111	0.000
0.168	43.745	0.120	0.000	0.000	0.000	8.801	0.000	0.080	4.831	0.000	0.000	0.000	0.056	0.075	0.000
0.321	56.812	0.773	0.000	0.000	0.077	6.623	0.000	0.017	5.998	0.000	0.000	0.037	0.108	0.108	0.018
0.000	53.081	0.116	0.000	0.000	0.052	6.998	0.000	0.000	6.564	0.483	0.000	0.000	0.022	0.060	0.000
0.191	53.009	0.108	0.000	0.000	0.031	7.286	0.000	0.000	4.673	0.000	0.000	0.044	0.000	0.086	0.000
0.000	51.196	0.064	0.000	0.000	0.032	6.037	0.277	0.031	5.005	0.000	0.000	0.000	0.066	0.074	0.000
0.000	50.922	0.213	0.000	0.000	0.126	5.206	0.000	0.000	5.939	0.000	0.000	0.045	0.066	0.118	0.000
0.166	56.805	0.124	0.000	0.000	0.027	7.830	0.233	0.009	1.111	0.000	0.000	0.038	0.033	0.075	0.000
0.000	64.012	0.092	0.000	0.000	0.088	8.131	0.000	0.116	4.722	0.000	0.000	0.042	0.012	0.055	0.000
0.000	47.830	0.072	0.140	0.000	0.101	6.090	0.000	0.008	5.833	0.000	0.000	0.000	0.000	0.094	0.018
0.167	49.322	0.099	0.000	0.000	1.533	10.071	0.000	0.000	6.464	0.000	0.000	0.000	0.146	0.088	0.000
0.000	48.540	0.544	0.000	0.000	0.079	5.785	0.000	0.026	4.966	0.000	0.000	0.038	0.011	0.085	0.000
0.000	113.868	0.046	0.000	0.000	0.087	3.271	0.251	0.009	5.849	0.000	0.000	0.000	0.000	0.094	0.000
0.182	61.362	0.091	0.000	0.000	0.088	5.626	0.000	0.010	5.476	0.274	0.000	0.000	0.061	0.122	0.000
0.000	47.805	0.218	0.000	0.000	0.025	8.366	0.000	0.025	7.307	0.000	0.000	0.000	0.032	0.094	0.000
0.000	51.602	0.111	0.000	0.000	0.048	6.342	0.000	0.102	3.621	0.000	0.000	0.000	0.070	0.056	0.017
0.000	52.050	0.124	0.000	0.000	0.120	11.130	0.000	0.016	3.248	0.000	0.000	0.000	0.060	0.078	0.000
0.000	55.907	0.092	0.000	0.000	0.078	7.178	0.000	0.085	3.660	0.000	0.000	0.000	0.043	0.120	0.000
0.000	61.820	0.078	0.000	0.000	0.079	7.781	0.000	0.009	3.838	0.000	0.000	0.000	0.033	0.159	0.000
0.000	53.127	0.061	0.000	0.000	0.070	10.770	0.000	0.008	5.399	0.000	0.000	0.000	0.029	0.163	0.000
0.000	61.257	0.089	0.000	0.000	0.129	10.386	0.000	0.014	3.146	0.000	0.000	0.061	0.090	0.140	0.000
0.000	63.806	0.067	0.000	0.000	0.112	6.225	0.000	0.000	5.872	0.000	0.000	0.000	0.047	0.000	0.000
0.000	55.861	0.097	0.000	0.000	0.167	7.935	0.483	0.009	4.031	0.000	0.000	0.000	0.070	0.129	0.020
0.000	55.444	0.152	0.000	0.000	0.236	5.754	0.228	0.000	3.684	0.000	0.000	0.000	0.044	0.098	0.000
0.324	37.512	0.188	0.000	0.000	0.078	5.251	0.000	0.009	5.638	0.000	0.000	0.000	0.033	0.097	0.000
0.000	53.724	0.861	0.000	0.000	0.097	7.422	0.000	0.000	6.306	0.000	0.000	0.000	0.000	0.045	0.000
0.000	57.719	0.240	0.156	0.000	0.112	6.289	0.000	0.000	6.433	0.263	0.000	0.000	0.023	0.052	0.000
0.000	78.872	0.085	0.000	0.000	0.000	7.143	0.388	0.029	3.535	0.000	0.000	0.000	0.037	0.083	0.031
0.144	42.549	0.177	0.000	0.000	0.255	6.041	0.000	0.030	4.960	0.000	0.000	0.000	0.039	0.043	0.016
0.155	57.922	1.861	0.000	0.000	0.473	4.889	0.000	0.033	5.735	0.000	0.000	0.000	0.073	0.023	0.000
0.000	63.709	0.287	0.000	0.000	0.055	8.316	0.000	0.000	5.062	0.000	0.000	0.000	0.012	0.090	0.000
0.000	51.780	0.057	0.000	0.000	0.143	7.270	0.000	0.023	3.302	0.000	0.000	0.000	0.045	0.066	0.025
0.000	70.255	0.029	0.000	0.000	0.049	10.294	0.000	0.016	2.627	0.000	0.000	0.000	0.000	0.046	0.000
0.000	94.091	0.136	0.000	0.000	0.033	11.331	0.000	0.011	2.179	0.000	0.000	0.000	0.000	0.076	0.000
0.000	68.107	0.111	0.000	0.000	0.043	7.759	0.000	0.000	5.764	0.000	0.000	0.000	0.000	0.262	0.000
0.000	78.739	0.101	0.000	0.000	0.068	7.184	0.000	0.034	2.600	0.000	0.000	0.000	0.029	0.048	0.000
0.000	54.458	0.085	0.000	0.000	0.100	7.623	0.000	0.000	4.217	0.000	0.000	0.000	0.000	0.116	0.000
0.000	57.194	0.144	0.000	0.000	0.050	8.231	0.000	0.000	1.914	0.000	0.000	0.000	0.021	0.023	0.000
0.000	46.101	0.090	0.000	0.000	0.069	7.135	0.000	0.015	3.744	0.000	0.000	0.000	0.086	0.032	0.000
0.000	70.113	0.143	0.000	0.000	0.135	8.543	0.000	0.000	4.207	0.000	0.000	0.000	0.000	0.021	0.000
0.000	41.922	0.224	0.000	0.000	0.069	8.796	0.000	0.000	6.409	0.000	0.000	0.000	0.000	0.128	0.000
0.000	56.066	0.048	0.000	0.000	0.124	7.584	0.000	0.000	4.436	0.000	0.000	0.000	0.035	0.116	0.000
0.180	92.095	0.095	0.000	0.000	0.058	10.065	0.000	0.000	2.365	0.000	0.000	0.041	0.000	0.040	0.000
0.000	81.106	0.029	0.000	0.000	0.174	12.493	0.000	0.000	4.826	0.000	0.000	0.000	0.000	0.000	0.000
0.000	87.174	0.102	0.000	0.000	0.438	9.062	0.000	0.012	2.657	0.000	0.000	0.000	0.015	0.119	0.026
0.000	70.347	0.163	0.000	0.000	0.073	6.595	0.000	0.012	3.075	0.000	0.000	0.000	0.030	0.118	0.026
0.000	76.884	0.066	0.186	0.000	0.033	12.141	0.000	0.000	2.194	0.000	0.000	0.095	0.042	0.031	0.000
0.000	57.908	0.132	0.145	0.000	0.078	9.245	0.000	0.043	3.485	0.000	0.000	0.000	0.000	0.061	0.000
0.000	59.232	0.138	0.000	0.000	0.062	6.750	0.000	0.010	4.613	0.000	0.000	0.000	0.013	0.029	0.022
0.000	41.346	0.400	0.000	0.000	0.070	7.161	0.306	0.023	6.621	0.000	0.000	0.101	0.029	0.065	0.000
0.000	30.764	0.143	0.000	0.000	0.000	6.253	0.000	0.058	6.268	0.000	0.066	0.000	0.012	0.068	0.000
0.194	46.962	2.089	0.000	0.000	0.062	7.532	0.000	0.010	3.992	0.000	0.000	0.000	0.156	0.029	0.022
0.000	42.426	0.063	0.136	0.000	0.049	6.008	0.000	0.000	5.722	0.460	0.000	0.000	0.000	0.137	0.000
0.796	49.637	3.349	0.000	0.000	0.026	4.429	0.223	0.008	4.912	0.000	0.000	0.000	0.192	0.060	0.000
0.145	47.250	0.090	0.000	0.000	0.093	8.281	0.203	0.000	5.289	0.000	0.000	0.000	0.029	0.087	0.000
0.283	41.109	0.149	0.000	0.000	0.137	7.279	0.000	0.000	4.138	0.000	0.000	0.000	0.000	0.042	0.000
0.756	58.043	0.185	0.000	0.167	0.122	6.582	0.212	0.008	4.649	0.228	0.000	0.000	0.010	0.045	0.000
0.312	48.969	1.891	0.000	0.000	0.000	6.042	0.000	0.016	3.542	0.000	0.000	0.000	0.073	0.058	0.018
0.151	63.459	0.130	0.000	0.000	0.048	7.490	0.000	0.000	5.928	0.000	0.000	0.000	0.051	0.079	0.000
0.000	40.255	0.054	0.000	0.000	0.051	6.415	0.000	0.000	8.038	0.000	0.000	0.000	0.011	0.095	0.000
0.152	38.524	0.240	0.000	0.000	0.098	5.766	0.000	0.008	5.202	0.000	0.000	0.000	0.113	0.125	0.000
0.177	60.942	0.148	0.000	0.000	0.028	6.216	0.000	0.009	4.200	0.266	0.000	0.000	0.000	0.079	0.000
0.000	86.480	0.202	0.166	0.000	0.089	8.782	0.259	0.000	5.412	0.000	0.000	0.000	0.012	0.111	0.000
1.510	55.517	0.252	0.000	0.000	0.243	7.516	0.000	0.000	5.878	0.000	0.000	0.000	0.051	0.113	0.257
0.000	72.245	0.147	0.000	0.000	0.077	4.461	0.000	0.025	4.746	0.000	0.000	0.000	0.128	0.179	0.000
0.185	50.893	0.080	0.000	0.000	0.089	9.562	0.000	0.000	5.234	0.000	0.000	0.043	0.025	0.139	0.000
0.000	45.680	0.441	0.000	0.204	0.089	7.148	0.000	0.010	5.039	0.278	0.000	0.000	0.050	0.083	0.000
0.241	93.990	0.040	0.000	0.266	0.000	6.878	0.000	0.000	2.691	0.000	0.000	0.000	0.032	0.000	0.000
0.000	52.768	1.886	0.000	0.000	0.000	6.630	0.000	0.000	4.106	0.000	0.000	0.000	0.084	0.000	0.000
0.000	44.470	0.201	0.000	0.000	0.033	6.029	0.000	0.021	5.778	0.609	0.000	0.000	0.041	0.076	0.000
0.232	76.478	0.063	0.000	0.000	0.335	5.436	0.000	0.024	4.508	0.000	0.000	0.000	0.016	0.052	0.000
0.000	46.697	0.050	0.000	0.000	0.000	7.441	0.000	0.000	4.269	0.000	0.000	0.000	0.027	0.061	0.000
0.186	59.492	0.260	0.000	0.000	0.030	3.065	0.000	0.020	4.660	0.000	0.000	0.000	0.037	0.028	0.000
0.165	72.119	0.168	0.000	0.000	0.053	8.796	0.231	0.000	3.842	0.000	0.000	0.000	0.022	0.037	0.000
0.000	63.333	0.023	0.000	0.000	0.000	7.571	0.000	0.000	4.765	0.000	0.000	0.000	0.031	0.081	0.000
0.000	79.920	0.073	0.000	0.000	0.000	11.015	0.000	0.102	2.784	0.000	0.000	0.000	0.000	0.029	0.000
0.000	77.114	0.039	0.000	0.000	0.029	6.321	0.000	0.010	2.620	0.000	0.000	0.000	0.012	0.041	0.000
0.000	54.812	0.121	0.000	0.000	0.110	9.159	0.000	0.000	5.160	0.000	0.000	0.000	0.035	0.000	0.000
0.177	53.402	0.106	0.000	0.000	0.057	6.569	0.000	0.000	4.905	0.000	0.000	0.000	0.060	0.027	0.000
0.000	48.909	0.144	0.000	0.000	0.122	8.159	0.000	0.000	5.091	0.000	0.000	0.087	0.153	0.256	0.000
0.000	50.919	0.391	0.000	0.000	0.029	6.696	0.000	0.010	3.522	0.000	0.000	0.083	0.012	0.054	0.000
0.000	48.597	0.133	0.000	0.000	0.093	7.111	0.000	0.010	3.612	0.000	0.000	0.044	0.039	0.014	0.000
0.000	40.530	0.295	0.000	0.000	0.106	10.020	0.000	0.044	5.309	0.249	0.000	0.000	0.000	0.161	0.000
0.000	40.057	0.394	0.000	0.000	0.123	6.534	0.000	0.061	5.996	0.000	0.000	0.000	0.013	0.158	0.000
0.000	60.920	0.035	0.000	0.000	0.107	10.361	0.000	0.009	5.198	0.000	0.000	0.000	0.033	0.174	0.019
0.000	39.865	0.026	0.000	0.000	0.076	9.915	0.000	0.000	7.002	0.000	0.000	0.000	0.011	0.130	0.000
0.000	37.754	0.117	0.000	0.000	0.023	6.811	0.000	0.023	6.328	0.000	0.000	0.000	0.077	0.139	0.000
0.000	47.296	0.178	0.000	0.000	0.069	7.340	0.000	0.057	4.621	0.000	0.000	0.000	0.000	0.097	0.000
0.000	51.825	0.066	0.000	0.000	0.091	7.783	0.000	0.010	6.238	0.000	0.000	0.000	0.051	0.042	0.000
0.000	65.344	0.173	0.000	0.000	0.030	7.215	0.000	0.030	3.927	0.000	0.000	0.000	0.000	0.056	0.000
0.176	55.206	1.149	0.000	0.000	0.085	7.204	0.000	0.009	6.083	0.000	0.000	0.000	0.083	0.079	0.000
0.000	42.020	0.189	0.000	0.000	0.728	9.938	0.000	0.009	3.041	0.000	0.000	0.000	0.000	0.088	0.000
0.000	62.964	0.082	0.000	0.000	0.088	6.917	0.000	0.000	3.757	0.000	0.000	0.000	0.000	0.245	0.000
0.000	65.384	0.521	0.000	0.000	0.093	6.983	0.000	0.015	4.282	0.000	0.000	0.033	0.126	0.108	0.016
0.134	55.693	0.169	0.120	0.000	0.022	6.135	0.000	0.007	4.780	0.202	0.000	0.000	0.054	0.030	0.000
0.145	52.543	0.160	0.000	0.000	0.093	7.855	0.203	0.069	5.548	0.000	0.000	0.000	0.146	0.152	0.033
0.000	45.149	0.061	0.000	0.000	0.067	9.931	0.000	0.000	5.017	0.208	0.000	0.000	0.056	0.072	0.000
0.000	52.200	0.161	0.000	0.000	0.140	6.759	0.608	0.000	2.810	0.000	0.000	0.000	0.058	0.065	0.049
0.000	50.739	0.082	0.000	0.000	0.126	7.777	0.000	0.033	2.991	0.000	0.000	0.000	0.000	0.094	0.000
0.000	70.250	0.101	0.000	0.000	0.032	8.772	0.000	0.117	3.851	0.000	0.000	0.000	0.081	0.060	0.046
0.411	77.301	0.148	0.000	0.000	0.000	11.232	0.000	0.000	3.475	0.000	0.000	0.000	0.041	0.092	0.000
0.000	51.172	0.347	0.000	0.000	0.220	11.017	0.000	0.000	3.858	0.000	0.000	0.000	0.169	0.171	0.026
0.769	45.507	0.694	0.000	0.000	0.124	8.038	0.000	0.000	5.067	0.000	0.000	0.059	0.034	0.038	0.029
0.000	43.363	0.159	0.000	0.000	0.167	10.145	0.000	0.000	5.768	0.000	0.000	0.000	0.056	0.031	0.000
0.145	51.429	0.162	0.130	0.000	0.070	3.736	0.203	0.008	5.961	0.000	0.000	0.000	0.039	0.174	0.000
0.000	41.510	0.294	0.000	0.000	0.024	7.462	0.000	0.031	5.401	0.000	0.000	0.000	0.000	0.111	0.000
0.000	51.541	0.070	0.000	0.000	0.128	8.100	0.000	0.014	5.124	0.000	0.000	0.000	0.036	0.079	0.000
0.138	48.637	0.051	0.000	0.000	0.067	7.726	0.000	0.007	4.771	0.000	0.000	0.000	0.019	0.124	0.000
0.000	49.377	0.374	0.000	0.000	0.090	5.294	0.000	0.000	3.524	0.000	0.000	0.000	0.050	0.126	0.000
0.000	62.391	0.121	0.000	0.000	0.622	7.803	0.000	0.018	6.277	0.253	0.000	0.000	0.057	0.101	0.000
0.165	59.165	0.054	0.000	0.000	0.053	6.706	0.000	0.017	4.878	0.000	0.000	0.000	0.011	0.062	0.000

ENSG00000255134	ENSG00000182545	ENSG00000175319	ENSG00000226957	ENSG00000110321	ENSG00000259090	ENSG00000182628	ENSG00000139180	ENSG00000137501	ENSG00000254309	ENSG00000137691	ENSG00000250365	ENSG00000220745	ENSG00000162694	ENSG00000234693	ENSG00000140873
0.000	0.206	0.055	0.114	91.354	0.062	28.688	3.285	5.206	0.000	0.944	0.166	0.100	8.827	0.000	0.356
0.000	0.070	0.000	0.000	91.115	0.000	10.922	3.469	4.485	0.000	1.606	0.129	0.170	7.243	0.022	0.088
0.000	0.156	0.000	0.115	93.655	0.031	52.043	5.016	1.568	0.000	0.311	0.230	0.000	7.614	0.000	0.238
0.000	0.071	0.000	0.163	118.208	0.035	34.172	2.753	1.919	0.000	0.200	0.178	0.000	9.125	0.044	0.737
0.000	0.039	0.000	0.000	98.077	0.020	22.814	4.783	7.998	0.000	8.542	0.092	0.000	5.195	0.073	0.028
0.000	0.016	0.000	0.172	65.218	0.031	31.752	4.768	0.796	0.000	0.331	0.604	0.000	9.538	0.019	0.148
0.000	0.053	0.000	0.227	127.175	0.000	12.402	4.388	6.510	0.000	1.849	0.235	0.085	11.109	0.240	0.050
0.000	0.167	0.000	0.000	90.838	0.019	11.587	4.759	4.460	0.000	0.436	0.174	0.000	4.706	0.046	0.253
0.000	0.107	0.000	0.079	78.561	0.000	34.297	5.850	7.093	0.000	0.281	0.100	0.104	11.513	0.000	0.277
0.000	0.062	0.000	0.000	85.194	0.000	10.658	5.048	5.100	0.000	1.609	0.417	0.303	5.657	0.155	0.056
0.020	0.046	0.000	0.085	84.449	0.046	17.420	4.265	4.371	0.000	2.670	0.164	0.000	6.992	0.038	0.044
0.000	0.111	0.000	0.117	115.976	0.048	23.972	3.928	6.140	0.000	0.287	0.127	0.000	8.739	0.020	0.996
0.000	0.114	0.000	0.091	96.497	0.082	24.643	3.901	2.466	0.000	0.356	0.263	0.000	9.318	0.061	1.759
0.000	0.183	0.000	0.085	110.104	0.015	18.042	3.124	3.409	0.000	1.012	0.246	0.000	7.758	0.019	0.500
0.000	0.068	0.000	0.252	84.406	0.068	16.355	3.995	2.524	0.000	1.173	0.000	0.331	7.016	0.000	0.196
0.000	0.184	0.000	0.000	104.439	0.031	9.780	2.371	4.051	0.000	1.165	0.164	0.000	3.619	0.038	0.371
0.000	0.141	0.047	0.000	112.488	0.053	37.357	4.162	3.339	0.000	0.240	0.095	0.086	9.043	0.000	0.488
0.027	0.063	0.000	0.000	72.832	0.063	35.932	20.045	1.845	0.000	2.174	0.281	0.000	11.349	0.052	0.056
0.000	0.000	0.000	0.029	66.507	0.064	14.938	5.714	4.340	0.000	0.405	0.171	0.000	6.761	0.020	0.905
0.000	0.136	0.000	0.032	107.794	0.017	19.515	6.282	4.910	0.000	0.085	0.171	0.000	6.428	0.000	0.070
0.000	0.018	0.000	0.000	100.581	0.018	28.129	11.123	5.363	0.000	2.594	0.097	0.000	11.005	0.023	0.307
0.024	0.150	0.101	0.208	97.787	0.019	30.166	7.385	3.182	0.000	0.162	0.491	0.000	9.079	0.023	0.189
0.000	0.018	0.000	0.034	92.697	0.037	39.180	8.885	2.235	0.000	1.031	0.209	0.089	7.578	0.046	0.171
0.000	0.062	0.000	0.029	137.032	0.000	14.248	4.372	5.042	0.000	2.561	0.125	0.000	7.787	0.058	0.283
0.000	0.139	0.000	0.000	99.498	0.017	17.737	3.550	5.584	0.000	0.347	0.151	0.084	6.586	0.043	0.390
0.000	0.361	0.000	0.100	112.870	0.018	18.795	4.129	7.950	0.076	1.556	0.169	0.000	7.230	0.000	0.419
0.000	0.041	0.000	0.000	84.917	0.000	27.760	3.806	4.662	0.000	5.688	0.096	0.000	10.733	0.102	0.173
0.000	0.108	0.000	0.067	100.392	0.072	26.401	4.172	3.062	0.000	0.245	0.121	0.000	7.083	0.045	0.252
0.000	0.108	0.000	0.099	83.756	0.000	21.461	4.633	6.161	0.000	0.845	0.420	0.000	9.021	0.268	0.155
0.000	0.108	0.000	0.167	92.571	0.018	21.220	5.938	2.298	0.000	0.221	0.350	0.000	7.766	0.067	0.165
0.000	0.193	0.000	0.027	87.665	0.015	21.366	5.802	5.277	0.000	0.759	0.109	0.000	9.170	0.018	0.320
0.000	0.016	0.042	0.086	82.340	0.000	19.974	3.313	3.486	0.000	1.014	0.312	0.000	7.192	0.039	3.004
0.023	0.018	0.000	0.067	66.111	0.000	17.321	4.866	2.859	0.000	0.574	0.266	0.000	7.157	0.045	0.136
0.000	0.070	0.000	0.032	78.869	0.035	19.928	5.677	8.990	0.000	0.237	0.082	0.000	7.217	0.043	0.081
0.000	0.294	0.000	0.000	152.633	0.111	21.765	3.624	12.352	0.000	1.034	0.197	0.000	8.539	0.000	0.291
0.000	0.050	0.000	0.031	126.531	0.033	20.747	3.914	7.631	0.000	4.193	0.145	0.000	7.699	0.124	0.024
0.000	0.223	0.043	0.059	79.797	0.000	34.200	2.270	2.601	0.000	6.378	0.395	0.000	8.040	0.059	0.718
0.000	0.048	0.000	0.059	100.899	0.016	24.607	3.664	1.211	0.000	0.303	0.351	0.000	12.185	0.000	0.069
0.000	0.095	0.000	0.000	113.298	0.038	33.237	4.833	5.393	0.000	0.292	0.343	0.000	11.947	0.000	0.637
0.000	0.157	0.052	0.072	118.754	0.020	29.066	4.109	6.557	0.000	0.231	0.210	0.000	10.626	0.000	0.591
0.025	0.039	0.000	0.072	117.249	0.020	30.013	6.218	9.871	0.000	2.360	0.196	0.000	10.075	0.097	0.063
0.000	0.379	0.000	0.000	124.466	0.000	21.962	3.841	17.942	0.000	4.519	0.077	0.000	8.037	0.041	0.320
0.000	0.091	0.000	0.101	142.429	0.055	12.156	5.230	2.348	0.076	0.271	0.219	0.000	11.400	0.000	0.290
0.000	0.078	0.000	0.144	136.946	0.078	25.097	3.217	4.720	0.000	1.227	0.281	0.000	11.229	0.039	0.249
0.000	0.099	0.000	0.184	129.906	0.033	30.599	3.853	2.586	0.000	2.443	0.222	0.000	11.139	0.021	0.721
0.000	0.210	0.000	0.000	82.893	0.032	33.629	3.702	2.711	0.000	0.301	0.282	0.000	7.182	0.040	0.195
0.000	0.000	0.000	0.263	65.080	0.018	19.627	5.744	1.787	0.000	1.784	0.322	0.000	24.475	0.044	0.061
0.000	0.036	0.000	0.133	66.144	0.000	31.851	2.329	4.419	0.000	0.229	0.254	0.000	8.975	0.000	0.557
0.000	0.141	0.000	0.029	137.185	0.063	23.402	5.180	5.830	0.000	1.254	0.105	0.000	10.662	0.039	0.351
0.000	0.310	0.040	0.000	92.349	0.074	10.801	3.828	4.030	0.000	0.756	0.188	0.143	3.872	0.000	0.469
0.000	0.119	0.000	0.027	138.299	0.060	25.634	5.267	7.224	0.000	0.464	0.219	0.000	9.505	0.000	0.184
0.000	0.463	0.000	0.000	104.303	0.016	17.191	4.511	8.829	0.000	2.894	0.331	0.000	6.140	0.000	0.883
0.000	0.081	0.000	0.030	148.542	0.033	37.511	6.234	5.069	0.000	5.981	0.218	0.000	13.698	0.000	0.155
0.000	0.144	0.000	0.027	129.005	0.043	23.660	4.007	5.513	0.000	0.313	0.115	0.000	8.039	0.018	0.330
0.000	0.026	0.000	0.098	137.630	0.053	38.724	8.573	3.372	0.000	2.997	0.390	0.000	10.564	0.000	0.299
0.000	0.069	0.000	0.255	97.740	0.069	31.749	2.920	3.097	0.000	0.344	0.185	0.000	16.571	0.000	0.136
0.000	0.068	0.000	0.095	101.523	0.034	15.286	4.793	9.995	0.000	0.109	0.321	0.000	6.954	0.043	0.154
0.000	0.162	0.000	0.179	76.677	0.000	31.916	7.031	3.748	0.000	0.689	0.228	0.000	13.929	0.020	0.855
0.000	0.096	0.000	0.000	135.353	0.065	21.595	3.258	6.168	0.000	1.137	0.129	0.000	8.739	0.040	1.664
0.000	0.000	0.000	0.028	137.717	0.030	31.580	6.046	1.187	0.000	7.132	0.191	0.000	11.966	0.037	0.024
0.000	0.156	0.000	0.032	142.598	0.035	21.336	2.933	2.402	0.000	0.071	0.267	0.000	9.290	0.000	0.100
0.000	0.028	0.000	0.254	102.488	0.055	35.717	6.193	2.206	0.000	1.671	0.240	0.000	10.084	0.034	0.069
0.000	0.100	0.000	0.132	127.884	0.014	16.816	4.565	2.098	0.000	0.195	0.355	0.000	7.226	0.018	0.062
0.000	0.046	0.000	0.227	91.675	0.015	21.432	2.488	1.905	0.000	1.691	0.185	0.000	8.537	0.038	0.615
0.000	0.068	0.000	0.000	158.745	0.034	32.707	4.729	2.415	0.000	4.220	0.115	0.000	11.601	0.000	0.867
0.000	0.044	0.000	0.122	71.821	0.044	29.624	5.590	1.391	0.000	0.269	0.191	0.107	7.548	0.027	0.115
0.000	0.121	0.000	0.000	160.825	0.000	46.312	5.523	2.290	0.000	0.219	0.121	0.000	8.601	0.000	0.623
0.000	0.101	0.000	0.000	100.704	0.000	25.543	7.748	0.835	0.000	0.611	0.242	0.000	10.925	0.000	0.683
0.000	0.107	0.000	0.049	109.899	0.054	23.445	6.038	0.493	0.000	0.061	0.197	0.000	11.698	0.067	0.178
0.000	0.021	0.000	0.000	137.267	0.042	31.243	7.551	0.632	0.000	1.618	0.198	0.103	9.518	0.026	0.015
0.020	0.077	0.041	0.028	113.693	0.031	25.094	5.141	11.755	0.000	2.665	0.113	0.000	8.656	0.000	0.155
0.000	0.062	0.000	0.554	85.727	0.125	34.196	8.736	18.862	0.000	6.402	0.167	0.000	7.822	0.039	0.067
0.000	0.085	0.000	0.026	101.671	0.000	14.770	3.160	7.517	0.000	2.780	0.208	0.068	8.376	0.068	0.058
0.000	0.111	0.000	0.103	101.866	0.028	27.167	4.120	4.034	0.000	0.164	0.279	0.000	8.766	0.035	0.105
0.000	0.254	0.057	0.000	111.834	0.021	15.067	2.164	7.336	0.000	0.452	0.128	0.000	7.413	0.026	0.442
0.000	0.000	0.000	0.000	129.676	0.051	24.405	6.993	5.881	0.000	0.255	0.103	0.000	10.815	0.000	0.069
0.000	0.161	0.000	0.000	82.568	0.054	29.020	6.751	2.407	0.000	0.316	0.060	0.000	12.722	0.067	1.103
0.000	0.000	0.000	0.396	86.547	0.000	15.752	3.610	1.494	0.000	1.553	0.143	0.000	17.220	0.133	0.539
0.000	0.000	0.181	0.000	110.260	0.023	32.463	8.809	8.613	0.000	4.975	0.422	0.000	11.823	0.000	0.505
0.000	0.045	0.000	0.041	91.427	0.022	30.140	11.187	4.703	0.000	4.758	0.165	0.000	12.341	0.195	0.197
0.000	0.103	0.055	0.000	106.172	0.000	42.908	9.003	9.508	0.000	2.871	0.220	0.000	15.988	0.000	0.096
0.000	0.097	0.000	0.000	139.335	0.065	28.584	5.513	5.447	0.000	6.695	0.302	0.078	8.909	0.040	0.148
0.000	0.019	0.051	0.071	60.200	0.019	29.572	4.577	1.574	0.000	1.128	0.231	0.000	5.675	0.048	0.148
0.000	0.303	0.000	0.000	101.772	0.043	15.034	3.977	5.918	0.000	0.658	0.131	0.105	9.990	0.027	0.421
0.000	0.036	0.000	0.067	83.737	0.000	26.727	3.019	4.560	0.000	0.730	0.182	0.000	5.249	0.023	0.735
0.000	0.019	0.000	0.036	108.941	0.019	16.366	3.039	5.169	0.000	1.761	0.090	0.000	4.426	0.024	0.038
0.000	0.061	0.000	0.140	78.132	0.015	35.879	2.748	1.179	0.063	0.110	0.203	0.000	6.245	0.038	0.593
0.000	0.095	0.000	0.058	89.920	0.063	20.102	8.805	0.951	0.000	0.980	0.106	0.153	7.779	0.020	0.184
0.000	0.086	0.000	0.080	97.909	0.043	20.070	3.780	4.570	0.000	0.771	0.174	0.000	6.186	0.000	0.642
0.000	0.140	0.000	0.052	88.899	0.028	21.982	3.297	4.791	0.000	1.769	0.207	0.000	4.969	0.000	0.303
0.000	0.075	0.000	0.527	88.605	0.045	47.120	4.343	2.280	0.000	0.048	0.261	0.000	9.659	0.112	0.304
0.000	0.031	0.000	0.172	70.397	0.016	18.693	4.278	5.762	0.000	5.605	0.249	0.000	6.450	0.096	0.061
0.000	0.030	0.040	0.028	123.092	0.015	26.583	5.056	5.729	0.000	0.447	0.040	0.000	7.429	0.019	0.040
0.000	0.000	0.000	0.116	57.134	0.047	46.970	3.371	2.503	0.000	3.771	0.063	0.000	14.421	0.039	0.189
0.000	0.060	0.040	0.168	63.452	0.045	18.457	9.682	3.037	0.000	0.952	0.192	0.073	7.360	0.019	0.062
0.000	0.158	0.000	0.162	74.364	0.018	23.576	2.296	3.171	0.000	1.938	0.188	0.000	6.572	0.087	0.501
0.000	0.147	0.000	0.000	87.820	0.018	23.301	6.012	1.623	0.000	1.290	0.209	0.000	8.453	0.000	0.049
0.000	0.075	0.000	0.554	124.371	0.075	34.440	5.126	2.416	0.000	1.866	0.251	0.000	7.860	0.186	0.551
0.000	0.095	0.000	0.000	91.067	0.095	29.457	2.960	3.089	0.000	1.160	0.190	0.000	10.550	0.118	0.230
0.000	0.110	0.000	0.000	120.115	0.055	20.731	3.294	7.848	0.000	1.809	0.160	0.000	9.962	0.000	0.367
0.000	0.183	0.000	0.136	104.949	0.018	16.956	2.991	4.644	0.077	1.712	0.221	0.000	7.702	0.160	0.356
0.000	0.144	0.000	0.000	87.037	0.072	29.660	10.849	13.687	0.000	1.682	0.289	0.000	8.199	0.000	1.342
0.000	0.047	0.000	0.086	85.952	0.000	30.881	4.249	6.655	0.000	10.426	0.198	0.000	6.141	0.310	0.201
0.000	0.000	0.000	0.000	113.168	0.060	28.621	3.178	2.154	0.000	13.221	0.148	0.000	7.837	0.100	0.072
0.000	0.207	0.000	0.255	96.095	0.000	29.376	4.674	1.870	0.000	0.823	0.216	0.000	8.164	0.000	0.562
0.000	0.242	0.000	0.074	114.394	0.000	22.599	3.781	3.016	0.000	0.164	0.324	0.000	7.178	0.050	0.470
0.000	0.240	0.000	0.034	105.670	0.000	28.512	5.069	4.206	0.000	6.402	0.111	0.000	7.245	0.092	0.060
0.000	0.164	0.000	0.000	143.527	0.000	23.653	4.925	5.729	0.000	2.107	0.132	0.000	8.518	0.081	0.185
0.000	0.184	0.000	0.028	142.394	0.031	27.861	3.796	6.556	0.000	0.160	0.247	0.075	8.713	0.000	0.409
0.000	0.096	0.000	0.000	85.130	0.039	20.388	8.973	6.650	0.000	2.111	0.181	0.094	7.276	0.048	0.446
0.000	0.036	0.000	0.000	116.262	0.018	23.244	7.075	4.664	0.000	0.872	0.195	0.000	4.699	0.045	0.016
0.000	0.068	0.046	0.189	117.167	0.000	24.892	4.361	3.162	0.000	1.444	0.228	0.000	10.219	0.042	0.548
0.000	0.000	0.000	0.033	112.985	0.071	23.398	4.156	2.187	0.000	0.311	0.259	0.000	7.745	0.000	0.323
0.000	0.264	0.000	0.000	142.156	0.076	28.235	3.365	2.497	0.000	0.325	0.076	0.000	8.721	0.000	1.619
0.000	0.072	0.048	0.033	113.484	0.036	23.091	5.159	3.402	0.000	0.292	0.216	0.000	9.903	0.022	0.402
0.000	0.171	0.000	0.000	113.091	0.000	12.344	3.854	5.327	0.000	4.051	0.115	0.092	6.363	0.047	0.564
0.000	0.033	0.000	0.030	128.659	0.000	28.042	2.794	4.495	0.000	0.223	0.066	0.000	8.929	0.000	0.121
0.000	0.057	0.000	0.035	135.952	0.076	23.114	5.518	4.598	0.000	1.894	0.165	0.000	11.149	0.024	0.031
0.000	0.198	0.000	0.000	155.278	0.066	30.709	5.146	5.350	0.000	4.714	0.132	0.000	13.278	0.000	0.533
0.000	0.031	0.000	0.000	125.075	0.031	28.965	4.024	9.145	0.000	0.114	0.137	0.000	11.513	0.000	1.918
0.000	0.184	0.000	0.000	143.519	0.028	25.629	3.489	3.126	0.000	1.723	0.218	0.000	6.353	0.000	0.303
0.000	0.064	0.000	0.039	78.610	0.043	19.839	3.476	4.930	0.000	0.377	0.072	0.000	8.050	0.000	0.318
0.000	0.243	0.000	0.035	107.982	0.000	12.142	3.352	1.064	0.000	1.238	0.125	0.000	7.573	0.070	0.134
0.000	0.056	0.000	0.000	119.880	0.019	35.999	5.323	3.090	0.000	1.137	0.174	0.090	14.375	0.069	0.023
0.000	0.105	0.000	0.097	85.438	0.017	19.956	3.763	1.671	0.000	1.225	0.339	0.000	8.357	0.000	0.266
0.000	0.283	0.045	0.031	76.434	0.000	10.805	4.708	10.171	0.000	1.447	0.223	0.000	7.367	0.000	0.367
0.000	0.271	0.000	0.000	99.410	0.054	22.313	5.082	3.470	0.000	1.253	0.254	0.000	9.714	0.067	1.373
0.000	0.043	0.000	0.000	75.465	0.058	21.652	4.011	5.406	0.000	3.917	0.278	0.000	8.402	0.036	0.525
0.000	0.080	0.000	0.025	96.006	0.013	19.143	5.391	3.212	0.056	3.862	0.214	0.000	6.284	0.017	0.313
0.000	0.216	0.000	0.027	154.362	0.043	39.249	4.285	3.128	0.000	4.955	0.260	0.000	9.571	0.107	0.437
0.000	0.342	0.000	0.051	108.365	0.014	20.268	3.813	5.241	0.000	0.217	0.147	0.066	7.907	0.017	1.377
0.000	0.172	0.000	0.000	122.556	0.043	28.237	4.708	3.103	0.000	0.293	0.202	0.000	7.509	0.107	0.302
0.000	0.031	0.000	0.000	116.875	0.078	22.764	8.160	2.938	0.000	0.353	0.177	0.000	18.681	0.000	4.274
0.000	0.080	0.000	0.148	83.677	0.020	12.700	4.695	2.358	0.000	0.254	0.362	0.000	5.083	0.050	0.363
0.000	0.143	0.000	0.038	89.653	0.000	15.415	6.684	1.662	0.000	0.148	0.232	0.000	8.794	0.000	0.337
0.000	0.068	0.000	0.042	103.866	0.045	23.960	3.529	4.269	0.000	1.999	0.273	0.000	7.670	0.028	0.280
0.000	0.051	0.000	0.094	103.602	0.000	28.325	4.078	6.067	0.000	0.380	0.289	0.000	5.827	0.032	0.320
0.000	0.206	0.000	0.038	80.360	0.083	29.764	3.959	2.340	0.000	0.542	0.249	0.000	8.204	0.026	0.315
0.000	0.086	0.000	0.053	139.141	0.043	23.094	4.449	5.954	0.000	3.701	0.221	0.000	7.832	0.036	0.509
0.000	0.044	0.000	0.027	133.075	0.030	32.266	3.110	3.057	0.000	6.581	0.267	0.000	10.460	0.018	0.098
0.034	0.210	0.000	0.000	117.605	0.026	23.864	3.475	5.542	0.000	1.169	0.141	0.000	7.797	0.000	0.775
0.000	0.247	0.000	0.051	115.375	0.055	15.107	3.812	7.423	0.000	0.920	0.073	0.000	6.772	0.000	0.209
0.000	0.093	0.000	0.069	113.975	0.019	28.237	4.048	2.298	0.000	4.351	0.162	0.000	8.922	0.023	0.160
0.000	0.050	0.000	0.093	104.353	0.017	24.601	4.262	5.587	0.000	0.461	0.302	0.081	9.882	0.104	0.282
0.000	0.131	0.000	0.091	115.939	0.082	21.652	4.859	4.213	0.000	0.111	0.110	0.000	11.246	0.041	0.138

ENSG00000187857	ENSG00000227018	ENSG00000243364	ENSG00000104863	ENSG00000177465	ENSG00000136715	ENSG00000226854	ENSG00000254203	ENSG00000259586	ENSG00000188487	ENSG00000112081	ENSG00000145198	ENSG00000222004	ENSG00000263958	ENSG00000253978	ENSG00000234272
0.000	0.121	1.548	3.290	0.618	7.901	0.000	0.000	0.000	0.029	24.645	0.574	0.000	3.847	1.378	0.000
0.000	0.083	4.965	4.141	0.517	5.423	0.000	0.000	0.000	0.435	34.836	0.268	0.000	0.079	0.628	0.072
0.000	0.018	2.951	3.846	0.634	11.316	0.000	0.000	0.000	0.076	67.323	0.562	0.000	50.017	0.707	0.000
0.000	0.021	2.167	5.616	0.422	9.869	0.000	0.000	0.000	0.093	47.533	0.544	0.021	0.119	2.194	0.000
0.000	0.023	3.270	2.962	1.679	6.493	0.000	0.000	0.000	0.007	31.910	0.239	0.012	0.000	3.801	0.081
0.000	0.073	1.447	3.958	0.553	12.651	0.000	0.000	0.000	0.255	140.062	0.573	0.009	3.458	1.632	0.192
0.000	0.021	5.836	3.402	0.791	9.082	0.000	0.000	0.000	0.514	32.597	0.088	0.010	0.000	0.877	0.144
0.000	0.065	2.937	5.855	1.401	5.710	0.000	0.000	0.000	0.091	23.881	1.644	0.000	0.915	2.694	0.382
0.000	0.101	2.825	4.452	0.902	5.307	0.000	0.000	0.000	0.015	40.993	0.376	0.013	16.633	3.113	0.000
0.000	0.000	4.496	3.666	1.866	5.024	0.000	0.000	0.000	0.131	30.180	0.196	0.037	0.280	1.040	0.257
0.000	0.090	4.201	3.603	0.746	5.815	0.000	0.000	0.000	0.144	23.353	0.247	0.018	0.756	1.752	0.000
0.000	0.093	1.655	3.957	0.660	11.295	0.000	0.000	0.000	0.017	31.309	1.350	0.019	0.890	1.587	0.261
0.000	0.058	3.400	5.101	0.374	9.551	0.000	0.000	0.000	0.034	34.155	0.421	0.000	0.000	4.600	0.135
0.000	0.090	2.570	5.346	0.427	11.298	0.000	0.000	0.000	0.011	30.596	0.867	0.018	0.172	4.412	0.000
0.000	0.160	2.653	3.077	1.453	9.269	0.000	0.000	0.000	0.119	28.830	1.783	0.000	0.765	5.037	0.281
0.000	0.126	2.776	1.634	0.653	7.544	0.000	0.000	0.000	0.043	16.723	0.330	0.036	0.034	0.620	0.000
0.000	0.062	1.006	5.205	0.457	10.994	0.000	0.000	0.000	0.092	43.922	0.706	0.010	0.000	1.852	0.000
0.000	0.000	3.482	11.440	0.628	5.574	0.000	0.000	0.000	0.022	63.428	0.386	0.000	7.906	8.444	0.000
0.000	0.019	6.708	4.593	0.559	6.770	0.000	0.000	0.000	0.011	23.033	0.310	0.000	0.143	5.171	0.000
0.000	0.080	5.115	3.219	0.459	10.313	0.017	0.000	0.000	0.000	28.520	0.268	0.030	0.000	1.951	0.070
0.000	0.043	5.487	10.394	1.522	4.471	0.000	0.000	0.133	0.019	55.129	0.711	0.000	1.918	3.900	0.000
0.000	0.110	1.106	5.179	0.754	16.167	0.000	0.000	0.000	0.046	28.072	3.087	0.011	5.611	0.269	0.155
0.000	0.022	2.214	6.580	0.951	6.700	0.018	0.000	0.000	0.179	56.756	0.972	0.022	0.371	2.495	0.000
0.000	0.037	4.093	3.269	0.460	8.972	0.000	0.000	0.000	0.812	28.663	0.157	0.000	0.035	0.037	0.000
0.000	0.082	3.500	4.912	0.689	7.886	0.000	0.074	0.000	0.079	33.191	0.342	0.010	0.117	1.451	0.000
0.000	0.043	2.592	3.784	0.863	11.700	0.000	0.000	0.000	0.044	30.708	0.549	0.064	5.887	0.905	0.149
0.000	0.048	8.523	4.504	0.270	7.867	0.000	0.000	0.000	0.079	27.582	0.300	0.012	2.709	2.535	0.169
0.000	0.000	1.675	6.921	0.603	9.567	0.000	0.000	0.000	0.277	39.311	1.213	0.021	16.575	3.090	0.148
0.000	0.042	4.495	10.009	0.583	7.444	0.000	0.000	0.000	0.106	30.943	1.006	0.021	0.081	3.976	0.000
0.000	0.021	2.448	11.251	0.787	8.877	0.018	0.000	0.000	0.013	45.390	1.204	0.000	0.243	3.010	0.000
0.000	0.017	4.113	3.592	0.746	8.921	0.014	0.000	0.000	0.021	44.222	0.491	0.026	9.561	4.102	0.000
0.000	0.018	2.996	5.009	0.300	9.378	0.000	0.000	0.000	0.016	30.714	0.673	0.000	0.070	1.594	0.000
0.000	0.021	4.688	10.566	1.341	6.785	0.000	0.000	0.000	0.158	25.514	1.677	0.043	2.843	7.546	0.075
0.000	0.020	4.005	5.163	0.460	6.874	0.000	0.000	0.000	0.030	28.798	0.171	0.021	3.445	1.330	0.072
0.000	0.433	2.761	0.856	0.635	9.601	0.000	0.000	0.000	0.000	39.242	0.223	0.000	0.083	0.790	0.152
0.000	0.118	5.352	2.316	1.219	6.667	0.000	0.000	0.000	0.029	29.035	0.621	0.000	0.487	3.220	0.069
0.000	0.243	4.431	4.325	1.229	11.897	0.000	0.000	0.000	0.072	24.063	1.417	0.075	0.393	3.152	0.066
0.000	0.075	4.833	4.690	0.630	12.732	0.000	0.000	0.000	0.022	35.777	0.186	0.038	3.322	1.327	0.131
0.000	0.112	3.603	5.166	0.694	9.403	0.000	0.000	0.000	0.053	47.432	0.822	0.011	8.139	3.664	0.313
0.000	0.069	1.712	3.970	0.388	10.489	0.000	0.000	0.000	0.007	49.935	0.455	0.000	7.000	1.122	0.000
0.000	0.138	2.500	2.869	0.366	8.067	0.000	0.000	0.000	0.075	21.769	1.413	0.012	0.701	1.349	0.000
0.000	0.194	3.381	2.977	0.711	7.957	0.000	0.000	0.000	0.006	31.722	0.645	0.000	0.592	2.201	0.000
0.000	0.085	1.671	3.785	0.700	10.074	0.000	0.000	0.000	0.006	46.727	0.820	0.000	0.407	0.303	0.075
0.000	0.110	3.105	2.951	0.561	9.509	0.000	0.000	0.000	0.011	32.408	0.717	0.018	0.210	0.631	0.000
0.000	0.117	2.584	5.505	0.674	10.543	0.000	0.000	0.000	0.116	42.988	1.278	0.010	0.037	0.475	0.137
0.021	0.152	6.361	3.199	0.797	9.568	0.000	0.000	0.000	0.294	31.455	0.467	0.000	0.509	6.520	0.000
0.000	0.042	23.571	4.218	0.262	20.159	0.000	0.000	0.000	0.012	42.755	0.354	0.053	0.040	0.765	0.147
0.000	0.127	1.730	3.615	0.522	10.203	0.035	0.000	0.000	0.025	41.761	0.849	0.011	10.450	1.978	0.074
0.000	0.110	4.499	3.616	0.071	11.854	0.000	0.000	0.000	0.044	44.239	0.442	0.000	1.756	0.932	0.129
0.000	0.069	3.407	4.041	1.057	5.589	0.000	0.000	0.000	0.031	23.452	0.355	0.000	0.298	0.282	0.000
0.000	0.070	1.608	13.604	2.511	7.534	0.000	0.000	0.000	0.088	40.694	3.391	0.000	4.736	1.806	0.000
0.000	0.319	3.260	3.397	0.462	5.522	0.000	0.000	0.000	0.072	31.089	0.381	0.019	0.179	1.637	0.132
0.000	0.115	4.786	3.493	0.347	8.570	0.000	0.000	0.000	0.017	58.861	0.204	0.029	7.303	3.024	0.000
0.000	0.186	2.019	4.561	0.482	7.665	0.000	0.000	0.000	0.000	34.605	0.385	0.008	0.032	2.296	0.118
0.000	0.156	2.111	6.880	1.491	9.152	0.000	0.000	0.000	0.277	46.709	0.892	0.000	3.804	1.640	0.218
0.000	0.162	2.880	1.556	0.070	11.702	0.000	0.000	0.000	0.024	46.583	0.447	0.000	3.096	0.986	0.284
0.000	0.121	3.725	4.630	0.522	8.359	0.000	0.000	0.000	0.030	36.744	0.298	0.000	0.038	3.064	0.212
0.000	0.152	3.566	3.577	0.230	8.804	0.000	0.000	0.000	0.045	33.437	0.488	0.019	0.036	0.926	0.000
0.000	0.057	3.070	2.267	0.408	15.501	0.000	0.000	0.000	0.095	29.716	0.128	0.019	7.911	0.575	0.000
0.000	0.176	4.845	0.975	0.213	10.832	0.000	0.000	0.000	0.037	32.808	0.204	0.009	0.000	5.546	0.000
0.000	0.143	4.677	2.812	0.493	13.499	0.000	0.000	0.000	0.157	37.577	0.744	0.010	0.195	3.515	0.000
0.000	0.065	1.594	4.932	0.140	6.649	0.000	0.000	0.000	0.115	65.198	0.253	0.000	12.546	2.034	0.113
0.000	0.051	1.551	6.488	0.276	14.679	0.000	0.000	0.000	2.266	32.036	1.064	0.000	5.727	2.049	0.177
0.000	0.126	9.409	2.890	0.710	14.286	0.000	0.000	0.000	0.225	29.781	0.277	0.018	0.035	1.905	0.000
0.000	0.040	7.495	4.065	0.478	12.744	0.000	0.000	0.000	0.084	56.781	0.362	0.000	3.192	0.408	0.000
0.000	0.026	1.984	14.101	0.648	8.940	0.000	0.000	0.000	0.023	42.510	0.691	0.000	2.520	3.042	0.000
0.000	0.000	1.433	12.798	0.567	7.778	0.000	0.000	0.000	0.032	61.883	0.512	0.000	0.407	1.151	0.000
0.000	0.024	3.285	15.771	0.531	4.568	0.020	0.000	0.000	0.028	46.352	0.274	0.000	0.045	2.350	0.000
0.000	0.094	3.809	3.916	0.095	13.224	0.000	0.000	0.000	0.000	36.279	0.313	0.016	0.000	1.275	0.000
0.000	0.174	3.089	2.529	0.504	5.782	0.000	0.000	0.000	0.081	39.399	0.221	0.000	0.047	4.231	0.174
0.000	0.054	6.243	3.002	0.594	8.547	0.015	0.000	0.000	0.016	38.398	0.548	0.000	0.899	1.542	0.000
0.000	0.073	5.201	9.518	2.339	3.201	0.000	0.000	0.000	0.413	32.780	0.762	0.000	1.747	2.523	0.000
0.000	0.050	3.590	2.992	0.229	5.672	0.000	0.000	0.000	0.010	22.658	0.118	0.000	0.063	5.483	0.058
0.000	0.033	5.887	3.861	0.565	10.011	0.000	0.000	0.000	0.000	37.061	0.564	0.016	0.187	4.706	0.115
0.000	0.100	2.416	2.771	0.345	7.287	0.000	0.000	0.000	0.007	23.624	0.186	0.000	0.095	0.152	0.000
0.000	0.030	3.179	6.223	0.454	9.692	0.000	0.000	0.000	0.018	49.472	0.433	0.000	0.172	7.859	0.000
0.000	0.021	5.444	9.262	1.288	4.986	0.000	0.000	0.000	0.037	57.395	0.269	0.011	0.120	1.192	0.000
0.000	0.000	15.257	3.151	1.251	17.391	0.000	0.000	0.000	0.112	40.787	0.157	0.000	0.481	2.809	1.324
0.000	0.106	5.171	8.928	1.348	4.968	0.000	0.000	0.000	0.031	63.774	0.410	0.000	1.011	4.399	0.093
0.000	0.026	3.217	2.855	0.422	4.135	0.022	0.000	0.000	0.047	40.658	0.061	0.000	0.353	6.848	0.000
0.000	0.097	2.911	9.713	0.648	3.070	0.040	0.000	0.000	0.036	64.390	0.103	0.000	0.046	3.238	0.000
0.000	0.000	2.803	3.863	0.672	5.468	0.000	0.000	0.000	0.023	53.817	0.270	0.000	0.109	3.539	0.066
0.000	0.000	3.365	5.675	0.846	8.957	0.000	0.000	0.000	0.000	33.086	0.798	0.000	0.774	3.471	0.000
0.000	0.102	5.147	3.071	1.024	7.950	0.000	0.186	0.000	0.000	24.287	0.440	0.013	0.195	0.569	0.179
0.000	0.064	1.307	2.640	0.929	9.459	0.000	0.000	0.000	0.038	24.386	1.285	0.000	1.220	1.382	0.000
0.000	0.045	3.619	1.053	1.632	7.601	0.000	0.000	0.000	0.880	26.341	0.197	0.000	0.043	1.010	0.000
0.000	0.089	0.791	2.620	0.323	13.221	0.029	0.000	0.000	0.021	38.917	0.355	0.009	0.068	0.433	0.125
0.000	0.019	3.898	2.355	0.673	22.199	0.000	0.000	0.000	0.022	35.901	0.169	0.019	0.071	1.054	0.065
0.000	0.102	3.355	3.946	0.234	12.407	0.000	0.000	0.000	0.025	28.368	0.504	0.000	0.097	4.848	0.000
0.000	0.066	1.014	3.825	0.713	8.089	0.000	0.000	0.000	0.049	25.663	0.882	0.000	0.252	3.751	0.000
0.000	0.018	0.698	6.320	0.305	12.031	0.000	0.000	0.000	0.115	37.168	1.532	0.018	1.078	1.788	0.124
0.020	0.018	5.749	3.549	0.244	8.408	0.000	0.000	0.000	0.038	26.438	0.324	0.000	0.695	2.658	0.064
0.000	0.018	2.497	3.475	0.144	17.509	0.000	0.000	0.000	0.271	31.989	0.544	0.018	5.906	3.313	0.000
0.000	0.111	5.835	2.445	0.302	11.883	0.015	0.000	0.000	0.016	30.125	0.771	0.019	2.992	1.981	0.000
0.000	0.142	1.535	5.366	0.322	10.954	0.000	0.000	0.000	0.053	18.090	0.968	0.009	2.885	2.234	0.125
0.000	0.124	7.890	5.049	0.579	12.159	0.017	0.000	0.000	0.055	41.254	0.620	0.010	0.079	3.051	0.144
0.000	0.086	3.227	4.863	0.168	10.185	0.000	0.000	0.000	0.006	30.727	0.219	0.011	3.919	0.394	0.000
0.000	0.264	2.418	5.278	0.456	12.016	0.364	0.000	0.000	0.105	46.224	0.596	0.044	23.717	1.786	0.000
0.000	0.279	9.138	3.730	0.481	8.893	0.000	0.000	0.000	0.083	38.572	0.476	0.000	0.852	3.391	0.000
0.000	0.043	2.883	3.475	0.178	9.867	0.000	0.000	0.000	0.006	29.492	0.493	0.000	0.083	1.536	0.076
0.000	0.043	2.734	2.384	0.512	8.555	0.000	0.000	0.000	0.090	31.500	0.380	0.022	0.947	2.011	0.000
0.000	0.056	2.888	7.520	0.815	4.151	0.000	0.000	0.000	0.000	66.438	0.045	0.028	0.161	13.075	0.197
0.000	0.000	5.466	3.321	0.221	8.000	0.000	0.000	0.000	0.158	30.172	0.645	0.000	0.070	3.042	0.000
0.000	0.118	3.866	3.142	0.387	9.647	0.019	0.000	0.000	0.105	24.653	0.479	0.012	0.135	1.913	0.083
0.000	0.108	2.555	3.807	0.245	9.713	0.000	0.000	0.000	0.048	28.556	0.520	0.000	0.000	5.483	0.095
0.000	0.047	1.301	4.825	0.614	9.383	0.078	0.000	0.000	0.098	29.250	0.540	0.000	3.892	0.865	0.000
0.000	0.000	3.976	1.729	0.318	8.813	0.000	0.000	0.000	0.090	30.590	0.371	0.000	0.041	3.695	0.000
0.000	0.039	3.032	2.856	1.081	10.835	0.000	0.000	0.000	0.343	51.024	0.456	0.000	15.150	4.372	0.000
0.000	0.018	0.963	4.269	0.219	9.910	0.000	0.000	0.000	0.005	49.048	0.370	0.000	0.035	0.513	0.000
0.000	0.023	6.894	9.868	0.939	6.913	0.000	0.000	0.000	0.061	50.901	0.367	0.000	1.816	1.745	0.079
0.000	0.000	5.173	2.870	0.498	5.284	0.000	0.000	0.000	0.006	42.206	0.171	0.000	0.326	10.997	0.075
0.000	0.060	2.751	2.740	3.037	8.992	0.000	0.000	0.000	0.024	33.721	0.303	0.000	0.115	1.422	0.000
0.000	0.041	2.692	3.674	0.510	8.958	0.000	0.000	0.000	0.055	33.954	0.542	0.000	0.830	2.897	0.000
0.000	0.044	1.862	2.529	0.919	10.789	0.000	0.000	0.000	0.040	40.112	0.316	0.000	0.085	1.439	0.155
0.000	0.253	4.781	3.383	0.482	8.166	0.000	0.000	0.000	0.231	29.365	0.390	0.011	0.000	2.307	0.000
0.000	0.022	2.511	2.720	0.425	6.188	0.000	0.000	0.000	0.046	26.542	0.354	0.011	0.000	1.043	0.000
0.000	0.096	0.888	5.872	0.942	11.558	0.000	0.000	0.000	0.132	32.809	1.700	0.000	2.728	0.548	0.135
0.000	0.223	3.988	3.666	0.502	8.948	0.000	0.000	0.000	0.033	35.076	0.970	0.000	3.115	2.401	0.157
0.000	0.078	3.116	4.760	0.511	8.044	0.000	0.000	0.000	0.017	50.434	0.259	0.000	0.000	1.533	0.000
0.000	0.092	2.814	2.950	0.271	13.217	0.015	0.000	0.000	0.016	35.724	0.204	0.009	0.035	1.010	0.129
0.000	0.067	2.304	2.620	0.382	10.621	0.000	0.000	0.000	0.059	29.792	0.383	0.000	4.365	1.319	0.000
0.000	0.201	2.333	3.726	0.325	8.441	0.000	0.000	0.000	0.030	39.966	0.331	0.038	3.886	1.120	0.088
0.000	0.000	15.336	1.191	0.399	11.204	0.000	0.000	0.000	0.013	30.904	0.078	0.000	0.168	0.892	0.000
0.000	0.131	3.335	4.831	0.642	8.124	0.000	0.000	0.000	0.019	46.229	0.210	0.000	0.042	1.949	0.153
0.000	0.021	2.716	2.446	0.859	13.687	0.017	0.000	0.000	0.079	33.816	1.128	0.031	0.313	3.826	0.072
0.000	0.117	2.827	7.682	2.525	7.295	0.000	0.000	0.000	0.029	20.182	1.911	0.000	1.793	1.983	0.137
0.000	0.064	2.575	7.272	0.358	7.079	0.000	0.000	0.000	0.151	43.840	0.772	0.000	0.365	0.905	0.000
0.000	0.067	3.581	4.463	1.172	9.378	0.000	0.000	0.000	0.045	31.831	0.600	0.017	0.869	4.615	0.118
0.000	0.016	5.088	2.556	0.250	17.336	0.000	0.000	0.000	0.005	25.800	0.234	0.000	2.120	1.236	0.000
0.000	0.068	3.593	3.819	3.229	12.920	0.000	0.000	0.000	0.040	45.522	1.334	0.009	0.162	3.877	0.000
0.000	0.048	3.562	4.515	0.334	9.593	0.000	0.000	0.000	0.024	31.173	0.361	0.000	0.061	2.056	0.282
0.000	0.253	3.439	3.451	1.073	6.456	0.000	0.000	0.000	0.105	32.967	0.135	0.051	1.646	1.028	0.000
0.000	0.055	4.541	6.724	0.728	5.878	0.000	0.000	0.000	0.005	54.411	0.238	0.000	0.070	3.528	0.000
0.000	0.047	2.451	10.102	0.589	12.072	0.000	0.000	0.000	0.189	32.819	1.202	0.012	3.460	2.051	0.082
0.000	0.048	5.555	9.190	0.807	7.435	0.000	0.000	0.000	0.028	36.109	0.333	0.000	0.183	2.041	0.084
0.000	0.160	3.524	3.902	0.425	9.130	0.044	0.000	0.000	0.040	30.989	0.351	0.000	4.269	3.184	0.093
0.000	0.120	4.900	3.408	0.684	8.282	0.000	0.000	0.000	1.163	18.450	1.335	0.000	0.457	1.394	0.105
0.000	0.097	1.019	2.627	0.409	11.690	0.000	0.000	0.000	0.346	47.002	0.912	0.000	0.185	1.378	0.000
0.000	0.051	3.660	2.371	0.161	13.249	0.000	0.000	0.000	0.005	39.812	0.280	0.000	0.032	4.801	0.119
0.000	0.035	4.999	2.472	1.325	8.550	0.000	0.000	0.000	0.041	33.887	0.849	0.000	0.662	5.591	0.061
0.000	0.031	2.349	2.233	0.935	9.434	0.000	0.000	0.000	0.046	38.017	0.303	0.000	0.177	2.573	0.108
0.000	0.097	3.191	3.003	0.376	7.076	0.000	0.000	0.000	0.263	31.520	0.144	0.000	0.031	0.294	0.057
0.000	0.131	7.426	2.427	0.198	11.936	0.018	0.000	0.000	0.058	26.363	0.525	0.000	1.794	1.683	0.000
0.000	0.079	2.584	6.350	0.542	12.279	0.000	0.000	0.000	0.000	37.683	0.947	0.020	0.225	2.985	0.069
0.000	0.077	4.933	4.283	0.574	8.558	0.000	0.000	0.000	0.006	34.626	0.329	0.000	0.074	2.110	0.068

ENSG00000238121	ENSG00000277515	ENSG00000271174	ENSG00000160886	ENSG00000201000	ENSG00000221887	ENSG00000254308	ENSG00000255533	ENSG00000116514	ENSG00000206852	ENSG00000165474	ENSG00000100505	ENSG00000137440	ENSG00000267315	ENSG00000119227	ENSG00000229814
0.087	0.000	0.000	0.018	0.000	0.059	0.000	0.000	11.019	0.000	27.972	26.648	0.000	0.000	3.738	0.520
0.042	0.000	0.000	0.265	0.000	0.404	0.000	0.146	14.367	0.000	31.048	3.611	0.000	0.240	1.835	1.011
0.024	0.000	0.000	0.139	0.000	0.090	0.000	0.000	14.266	0.000	0.286	19.764	0.000	0.000	1.703	1.012
0.091	0.000	0.000	0.042	0.000	0.080	0.000	0.147	13.202	0.000	7.798	36.076	0.000	0.161	1.203	0.319
0.131	0.000	0.000	0.058	0.000	1.964	0.000	0.000	15.155	0.000	1.498	10.169	0.000	0.090	1.235	0.496
0.070	0.000	0.000	0.028	0.000	0.275	0.000	0.065	15.236	0.000	0.453	26.647	0.000	0.000	3.311	0.224
0.360	0.000	0.000	0.041	0.000	0.144	0.000	0.073	27.768	0.000	11.491	9.072	0.015	0.160	2.309	0.189
0.101	0.000	0.000	0.055	0.000	0.867	0.000	0.000	13.057	0.000	1.565	12.278	0.000	0.000	2.103	0.467
0.103	0.000	0.000	0.038	0.000	0.378	0.000	0.089	10.475	0.000	31.075	12.641	0.000	0.098	3.829	0.308
0.132	0.000	0.000	0.074	0.000	0.537	0.000	0.000	14.772	0.000	6.435	5.045	0.000	0.000	0.977	0.449
0.222	0.000	0.000	0.073	0.000	0.748	0.000	0.318	13.661	0.000	14.358	8.403	0.000	0.209	3.431	0.165
0.273	0.000	0.000	0.028	0.000	0.260	0.000	0.132	18.341	0.000	1.793	38.883	0.014	0.072	3.634	0.228
0.044	0.000	0.000	0.019	0.000	0.101	0.000	0.000	12.442	0.000	5.114	25.750	0.000	0.075	4.067	0.177
0.032	0.000	0.000	0.045	0.000	0.207	0.000	0.381	19.667	0.000	2.849	29.376	0.013	0.139	4.546	0.165
0.041	0.000	0.000	0.081	0.000	0.140	0.000	0.283	10.020	0.000	8.216	14.426	0.000	0.000	3.156	0.736
0.300	0.000	0.000	0.041	0.000	0.415	0.000	0.064	24.379	0.000	2.171	10.337	0.095	0.070	2.601	0.331
0.011	0.000	0.000	0.052	0.000	0.094	0.000	0.000	12.272	0.000	3.315	28.085	0.000	0.080	2.822	0.191
0.038	0.000	0.000	0.019	0.000	0.318	0.000	0.000	10.505	0.000	0.621	5.936	0.000	0.000	2.872	0.830
0.077	0.000	0.000	0.028	0.000	0.498	0.000	0.132	20.501	0.000	2.127	27.543	0.000	0.073	2.501	0.345
0.031	0.000	0.000	0.010	0.000	0.035	0.000	0.354	21.791	0.198	0.590	42.396	0.000	0.078	1.789	0.246
0.038	0.000	0.000	0.124	0.000	5.516	0.000	0.075	13.611	0.000	0.557	7.202	0.000	0.249	2.274	0.654
0.045	0.000	0.000	0.039	0.000	0.370	0.000	0.156	13.000	0.000	0.882	21.507	0.000	0.171	3.908	0.135
0.061	0.000	0.000	0.043	0.000	0.264	0.000	0.229	10.492	0.214	3.283	11.527	0.000	0.000	1.686	0.595
0.071	0.000	0.000	0.333	0.000	0.122	0.000	0.000	15.599	0.000	53.050	4.593	0.000	0.142	0.268	0.394
0.047	0.000	0.000	0.242	0.000	0.264	0.000	0.072	17.695	0.000	2.577	16.694	0.000	0.159	3.344	0.626
0.071	0.000	0.000	0.005	0.000	0.111	0.000	0.075	16.862	0.000	7.557	15.398	0.000	0.165	3.160	0.130
0.142	0.000	0.000	0.073	0.000	0.218	0.000	0.085	14.073	0.000	16.660	9.095	0.000	0.093	2.894	0.442
0.033	0.000	0.000	0.053	0.000	0.355	0.000	0.000	11.464	0.000	4.127	21.049	0.000	0.164	2.635	1.167
0.087	0.000	0.000	0.032	0.181	0.464	0.000	1.190	8.181	0.000	1.931	27.560	0.000	0.000	6.757	0.387
0.011	0.000	0.000	0.059	0.000	0.237	0.000	0.374	10.001	0.000	0.312	27.348	0.016	0.000	6.122	0.195
0.040	0.000	0.000	0.070	0.000	2.358	0.000	0.062	13.808	0.000	1.099	15.261	0.000	0.068	5.545	0.588
0.033	0.000	0.000	0.018	0.000	0.070	0.000	0.000	16.077	0.000	0.591	16.820	0.000	0.425	3.373	0.448
0.071	0.000	0.000	0.102	0.000	0.631	0.000	0.075	12.615	0.000	17.936	9.893	0.000	0.082	4.944	0.326
0.063	0.000	0.000	0.052	0.000	0.365	0.000	0.289	16.723	0.000	1.731	8.221	0.000	0.159	3.353	0.063
0.133	0.000	0.000	0.022	0.000	0.091	0.000	0.000	15.020	0.000	1.874	14.895	0.000	0.168	0.962	0.663
0.055	0.000	0.000	0.123	0.000	0.883	0.000	0.000	15.676	0.000	6.073	24.576	0.000	0.228	4.148	0.240
0.072	0.000	0.000	0.184	0.000	0.517	0.000	0.463	8.957	0.000	4.273	46.615	0.000	0.218	4.639	0.172
0.182	0.000	0.000	0.075	0.000	0.346	0.000	0.066	22.707	0.000	0.102	24.182	0.000	0.073	4.705	0.057
0.017	0.000	0.000	0.034	0.000	0.397	0.000	0.079	14.481	0.000	2.860	28.937	0.000	0.000	1.637	0.273
0.071	0.000	0.000	0.041	0.000	0.161	0.000	0.325	8.557	0.000	8.592	60.723	0.017	0.179	1.479	0.282
0.124	0.000	0.000	0.064	0.000	0.224	0.000	0.162	12.037	0.000	11.392	19.851	0.000	0.000	3.999	0.421
0.045	0.000	0.000	0.015	0.000	0.582	0.000	0.068	9.696	0.000	6.704	39.128	0.015	0.075	3.688	0.119
0.049	0.000	0.000	0.011	0.000	0.216	0.000	0.075	11.978	0.000	0.206	36.799	0.000	0.000	1.430	0.131
0.061	0.000	0.000	0.009	0.000	0.230	0.000	0.065	12.084	0.000	2.100	48.388	0.000	0.071	2.384	0.168
0.080	0.000	0.000	0.069	0.000	0.109	0.000	0.689	12.785	0.000	6.559	45.081	0.000	0.303	3.848	0.299
0.078	0.000	0.000	0.168	0.000	1.243	0.000	0.000	20.708	0.000	0.248	33.789	0.000	0.074	2.454	0.233
0.113	0.000	0.000	0.053	0.000	0.037	0.000	0.000	8.630	0.000	0.211	2.418	0.000	0.325	0.479	0.963
0.033	0.091	0.000	0.032	0.000	0.141	0.000	0.075	12.090	0.000	1.337	33.713	0.000	0.000	2.290	0.455
0.009	0.000	0.000	0.014	0.000	0.328	0.000	0.065	23.059	0.000	1.229	20.036	0.000	0.214	2.336	0.225
0.067	0.000	0.000	0.114	0.000	0.813	0.000	0.061	13.808	0.000	6.443	8.982	0.000	0.269	2.041	0.798
0.094	0.000	0.000	0.106	0.000	1.165	0.000	0.062	14.111	0.000	1.945	8.952	0.000	0.135	3.921	0.588
0.135	0.000	0.000	0.005	0.000	0.177	0.000	0.066	17.550	0.000	8.758	23.827	0.000	0.073	1.443	0.518
0.162	0.000	0.000	0.048	0.000	0.147	0.000	0.000	16.697	0.000	3.856	25.123	0.000	0.074	2.994	0.468
0.065	0.000	0.000	0.013	0.000	0.130	0.000	0.000	16.758	0.000	3.359	31.037	0.000	0.066	3.499	0.155
0.056	0.000	0.000	0.157	0.000	1.706	0.000	0.000	15.042	0.000	3.204	15.718	0.000	0.000	1.364	0.953
0.000	0.000	0.000	0.000	0.000	0.085	0.331	0.000	9.027	0.000	0.715	33.797	0.000	0.000	1.648	0.993
0.062	0.000	0.000	0.046	0.000	0.091	0.000	0.355	9.652	0.000	2.167	38.694	0.015	0.156	2.201	0.432
0.039	0.000	0.000	0.048	0.000	0.086	0.000	0.202	13.866	0.000	1.528	22.488	0.029	0.221	3.162	0.292
0.024	0.000	0.000	0.029	0.000	0.905	0.000	0.000	11.220	0.000	0.493	32.029	0.014	0.073	2.205	0.290
0.127	0.000	0.000	0.013	0.000	0.031	0.000	0.249	15.411	0.000	1.750	18.993	0.013	0.137	1.278	0.216
0.058	0.000	0.000	0.051	0.000	1.076	0.000	0.432	12.568	0.000	2.305	19.489	0.000	0.237	3.615	0.062
0.150	0.000	0.000	0.033	0.000	0.904	0.000	0.114	9.825	0.000	0.666	14.334	0.000	0.251	1.614	1.189
0.000	0.072	0.000	0.025	0.000	0.341	0.000	0.059	9.808	0.000	2.669	39.583	0.013	0.000	2.815	0.258
0.107	0.000	0.000	0.046	0.000	1.028	0.000	0.191	17.852	0.000	1.420	25.035	0.000	0.070	6.030	0.443
0.052	0.000	0.000	0.010	0.000	0.239	0.000	0.000	10.267	0.000	4.273	14.419	0.000	0.078	1.791	0.432
0.027	0.000	0.000	0.020	0.000	0.660	0.000	0.091	10.188	0.000	1.098	23.302	0.000	0.000	2.769	0.396
0.046	0.000	0.000	0.063	0.000	0.471	0.000	0.000	14.488	0.000	0.790	34.401	0.000	0.138	2.254	0.326
0.030	0.000	0.000	0.012	0.000	0.083	0.000	0.083	9.058	0.000	0.616	31.477	0.000	0.092	2.502	0.507
0.065	0.000	0.000	0.024	0.000	0.000	0.000	0.222	25.094	0.000	0.119	40.354	0.000	0.000	1.458	0.385
0.038	0.000	0.000	0.006	0.000	1.241	0.000	0.000	12.183	0.000	0.261	13.349	0.000	0.000	4.277	0.457
0.019	0.000	0.000	0.027	0.000	0.171	0.000	0.000	16.159	0.000	1.195	32.574	0.000	0.070	1.282	0.277
0.160	0.000	0.000	0.065	0.000	0.320	0.000	0.000	12.671	0.000	10.381	3.831	0.000	0.142	3.906	0.897
0.038	0.000	0.000	0.017	0.000	0.400	0.000	0.059	13.108	0.000	54.265	27.395	0.000	0.257	0.933	0.457
0.034	0.000	0.000	0.074	0.000	1.085	0.000	0.000	21.998	0.000	0.371	28.157	0.000	0.000	6.251	0.501
0.058	0.000	0.000	0.019	0.000	0.165	0.000	0.000	17.091	0.000	4.328	30.927	0.037	0.000	1.877	0.305
0.039	0.000	0.000	0.030	0.000	0.409	0.000	0.106	7.057	0.000	3.245	47.730	0.000	0.117	3.798	0.552
0.065	0.000	0.000	0.016	0.000	0.374	0.000	0.000	24.809	0.000	1.739	11.389	0.000	0.407	3.255	0.836
0.065	0.000	0.000	0.349	0.000	0.132	0.000	0.000	6.891	0.000	1.006	3.547	0.000	0.488	1.360	1.157
0.095	0.000	0.000	0.113	0.000	2.246	0.000	0.000	10.794	0.000	1.579	16.037	0.000	0.205	3.118	0.324
0.075	0.000	0.000	0.013	0.000	0.009	0.000	0.093	15.114	0.000	1.951	5.680	0.000	0.102	1.961	1.050
0.180	0.000	0.000	0.012	0.000	1.301	0.000	0.085	6.249	0.000	1.332	5.280	0.000	0.188	3.174	1.037
0.054	0.000	0.000	0.014	0.000	0.603	0.000	0.067	11.793	0.000	0.694	10.568	0.000	0.074	2.531	0.523
0.110	0.000	0.000	0.023	0.000	0.551	0.000	0.000	12.526	0.000	17.853	16.579	0.000	0.000	2.003	0.207
0.072	0.000	0.000	0.058	0.000	0.303	0.000	0.000	15.593	0.000	14.700	14.169	0.000	0.099	2.470	0.312
0.060	0.000	0.000	0.038	0.000	1.183	0.000	0.000	10.421	0.000	3.276	15.245	0.032	0.000	2.672	0.196
0.012	0.000	0.000	0.017	0.000	0.609	0.000	0.000	11.709	0.000	8.636	6.160	0.017	0.088	2.530	0.277
0.069	0.000	0.000	0.031	0.000	0.298	0.000	0.000	6.009	0.000	0.180	18.571	0.013	0.069	1.996	0.000
0.067	0.000	0.000	0.061	0.000	1.537	0.000	0.000	18.927	0.000	3.292	15.838	0.000	0.000	0.254	0.114
0.013	0.000	0.000	0.030	0.000	0.071	0.000	0.120	11.650	0.000	6.763	66.869	0.013	0.197	3.506	0.104
0.008	0.000	0.000	0.158	0.000	0.692	0.000	0.350	10.314	0.000	5.483	30.233	0.000	0.000	4.816	0.506
0.063	0.000	0.000	0.009	0.000	0.172	0.000	0.062	6.030	0.000	1.430	37.536	0.013	0.205	6.274	0.108
0.037	0.000	0.000	0.078	0.000	0.051	0.000	0.321	18.242	0.000	0.712	22.350	0.000	0.141	6.043	0.502
0.041	0.000	0.000	0.115	0.000	0.239	0.000	0.000	12.578	0.000	0.266	21.457	0.000	0.000	0.876	0.269
0.080	0.000	0.000	0.167	0.000	0.161	0.000	0.065	9.097	0.000	0.891	25.296	0.000	0.000	1.189	0.282
0.119	0.000	0.000	0.125	0.000	0.273	0.072	0.000	9.485	0.000	0.284	37.837	0.000	0.138	4.465	0.816
0.079	0.000	0.000	0.052	0.000	0.072	0.000	0.000	12.550	0.000	0.615	39.348	0.000	0.080	3.156	0.126
0.017	0.000	0.000	0.011	0.000	0.038	0.000	0.000	17.706	0.000	0.200	76.938	0.000	0.168	1.495	0.397
0.023	0.000	0.000	0.000	0.000	1.077	0.000	0.000	7.743	0.000	1.962	17.693	0.000	0.000	3.273	0.539
0.286	0.000	0.000	0.070	0.000	0.584	0.000	0.197	9.999	0.000	4.779	25.278	0.042	0.432	1.877	1.024
0.072	0.000	0.000	0.076	0.000	0.204	0.000	0.000	12.591	0.000	2.219	43.644	0.000	0.252	2.681	0.530
0.127	0.000	0.000	0.081	0.000	0.218	0.000	0.152	13.002	0.000	1.930	24.439	0.000	0.167	1.589	0.198
0.036	0.000	0.000	0.192	0.000	0.275	0.000	0.099	9.430	0.000	0.959	15.158	0.000	0.000	1.887	0.517
0.207	0.000	0.000	0.230	0.000	1.936	0.000	0.387	6.305	0.000	9.387	12.631	0.000	0.142	3.010	0.504
0.042	0.000	0.000	0.071	0.000	0.470	0.000	0.000	20.449	0.000	1.209	16.577	0.000	0.091	2.719	0.361
0.042	0.000	0.000	0.061	0.000	1.237	0.000	0.000	7.143	0.000	5.057	39.370	0.000	0.105	5.592	0.083
0.012	0.000	0.000	0.000	0.000	0.662	0.000	0.000	17.332	0.000	1.414	40.025	0.036	0.000	3.718	0.581
0.072	0.000	0.000	0.060	0.000	0.083	0.000	0.000	10.511	0.000	1.067	10.338	0.000	0.084	2.735	0.133
0.010	0.000	0.000	0.005	0.000	0.074	0.000	0.000	9.179	0.000	0.825	17.654	0.000	0.149	1.975	0.177
0.009	0.000	0.000	0.014	0.000	0.139	0.000	0.000	14.902	0.000	8.636	54.768	0.000	0.070	1.745	0.166
0.012	0.000	0.000	0.029	0.000	0.372	0.000	0.160	11.043	0.000	40.195	14.983	0.017	0.088	1.554	0.555
0.049	0.000	0.000	0.022	0.000	0.798	0.000	0.075	7.993	0.000	0.422	19.333	0.000	0.083	1.363	0.327
0.031	0.000	0.000	0.061	0.000	0.133	0.000	0.000	11.809	0.000	9.844	50.647	0.000	0.000	1.757	0.123
0.043	0.000	0.000	0.016	0.000	0.318	0.000	0.000	14.458	0.000	0.322	33.809	0.016	0.000	1.657	0.190
0.114	0.000	0.132	0.045	0.000	0.077	0.000	0.470	10.451	0.000	2.106	31.257	0.000	0.000	3.057	1.087
0.043	0.000	0.000	0.027	0.000	1.619	0.000	0.074	16.621	0.000	6.038	21.946	0.000	0.163	3.747	0.323
0.034	0.000	0.000	0.068	0.000	0.125	0.000	0.000	11.470	0.000	14.356	23.791	0.000	0.000	3.638	0.274
0.064	0.000	0.000	0.005	0.000	0.128	0.000	0.000	13.785	0.000	6.139	46.483	0.000	0.299	4.260	0.177
0.092	0.000	0.000	0.056	0.000	0.117	0.000	0.315	11.132	0.000	2.920	19.324	0.017	0.173	1.895	0.890
0.040	0.000	0.000	0.024	0.000	0.792	0.000	0.137	8.591	0.000	6.673	33.355	0.000	0.451	2.703	0.594
0.000	0.000	0.000	0.005	0.000	0.167	0.000	0.065	11.602	0.000	2.309	55.360	0.000	0.072	2.753	0.678
0.073	0.000	0.000	0.021	0.000	0.623	0.000	0.059	11.903	0.000	6.800	17.312	0.000	0.129	4.085	0.358
0.052	0.000	0.000	0.095	0.000	1.079	0.000	0.000	12.583	0.000	4.433	18.858	0.000	0.097	3.821	0.615
0.396	0.000	0.000	0.166	0.000	0.023	0.000	0.078	7.602	0.000	18.884	1.287	0.000	0.000	0.538	0.202
0.039	0.000	0.000	0.011	0.000	0.176	0.000	0.154	8.530	0.000	6.933	8.222	0.033	0.169	1.097	0.468
0.053	0.000	0.000	0.031	0.000	0.401	0.000	0.217	16.944	0.000	0.319	40.815	0.000	0.159	2.639	0.063
0.116	0.000	0.000	0.118	0.000	1.175	0.000	0.000	14.660	0.000	1.438	14.202	0.000	0.000	4.436	0.539
0.196	0.000	0.000	0.048	0.000	0.178	0.000	0.000	14.079	0.000	6.726	22.533	0.000	0.000	3.261	0.586
0.121	0.000	0.000	0.153	0.000	0.230	0.000	0.119	15.706	0.000	1.821	15.820	0.000	0.000	4.305	0.516
0.032	0.000	0.000	0.004	0.000	0.169	0.000	0.055	18.910	0.000	3.003	54.596	0.000	0.000	1.321	0.239
0.039	0.000	0.000	0.013	0.000	0.372	0.000	0.060	11.253	0.000	2.887	13.134	0.013	0.000	6.417	0.466
0.054	0.000	0.000	0.012	0.000	0.157	0.000	0.114	22.886	0.000	3.383	34.169	0.000	0.187	4.357	0.247
0.026	0.000	0.000	0.013	0.000	1.594	0.000	0.000	14.225	0.000	4.091	22.025	0.152	0.197	2.850	0.621
0.047	0.000	0.054	0.014	0.000	0.262	0.000	0.000	15.897	0.000	0.793	26.033	0.000	0.071	2.327	1.010
0.018	0.000	0.000	0.018	0.000	0.797	0.000	0.332	14.260	0.000	1.246	29.290	0.000	0.182	6.031	0.360
0.055	0.000	0.000	0.344	0.000	0.117	0.000	0.254	10.359	0.000	6.426	23.211	0.000	0.186	3.152	0.440
0.164	0.000	0.000	0.047	0.000	0.948	0.000	0.000	9.931	0.000	4.037	25.958	0.000	0.310	3.128	0.571
0.200	0.000	0.000	0.090	0.000	0.386	0.000	0.211	17.170	0.000	17.057	14.300	0.000	0.116	3.987	0.275
0.037	0.000	0.000	0.024	0.000	0.365	0.000	0.086	10.613	0.000	0.806	36.638	0.000	0.188	4.834	0.743
0.056	0.000	0.000	0.017	0.000	0.301	0.000	0.119	10.236	0.000	1.009	46.437	0.000	0.197	7.676	0.622
0.058	0.000	0.000	0.035	0.000	0.200	0.000	0.061	10.056	0.000	0.998	28.278	0.000	0.000	4.990	0.159
0.024	0.000	0.000	0.070	0.000	0.703	0.000	0.218	11.042	0.000	2.406	25.601	0.000	0.000	2.870	0.569
0.075	0.000	0.000	0.016	0.000	0.225	0.000	0.057	11.735	0.000	18.626	12.067	0.000	0.250	1.475	0.642
0.028	0.000	0.000	0.292	0.000	0.702	0.000	0.000	14.314	0.000	4.195	19.025	0.000	0.169	2.921	0.134
0.025	0.000	0.000	0.138	0.000	0.144	0.000	0.346	11.691	0.000	0.586	44.806	0.015	0.000	3.828	0.240
0.025	0.000	0.000	0.019	0.000	0.020	0.000	0.136	31.733	0.000	3.620	64.762	0.000	0.149	2.320	0.531

ENSG00000211959	ENSG00000230575	ENSG00000199540	ENSG00000198324	ENSG00000258162	ENSG00000127588	ENSG00000053372	ENSG00000083814	ENSG00000182021	ENSG00000229508	ENSG00000143624	ENSG00000090905	ENSG00000148943	ENSG00000185015	ENSG00000267170	ENSG00000198218
0.175	0.000	0.000	6.598	0.171	0.327	13.550	3.497	0.029	0.000	6.821	4.554	4.081	0.994	0.226	11.850
2.476	0.000	0.000	3.825	0.000	0.150	31.385	1.799	0.013	0.000	4.329	3.038	6.396	2.451	0.192	9.390
1.014	0.000	0.000	6.737	0.130	0.076	33.604	5.870	0.041	0.000	3.359	3.751	8.158	0.529	0.128	15.838
0.250	0.000	0.000	4.211	0.184	0.345	24.594	7.569	0.013	0.000	6.981	5.809	7.939	1.056	0.242	14.732
2.055	0.000	0.000	4.220	0.010	0.024	18.851	4.290	0.000	0.128	4.287	2.143	6.565	1.251	0.054	6.790
1.887	0.000	0.168	6.715	0.953	1.384	16.682	8.080	0.033	0.000	12.507	9.896	8.045	1.194	0.170	19.093
2.471	0.000	0.000	4.918	0.018	0.107	18.732	2.694	0.012	0.000	6.339	6.487	6.505	1.090	0.144	11.744
1.045	0.000	0.000	2.935	0.192	1.693	13.839	2.944	0.009	0.000	3.408	2.445	7.136	0.631	0.304	9.510
0.000	0.000	0.000	3.819	0.378	0.365	21.886	3.743	0.010	0.000	4.225	3.795	9.095	1.528	0.176	12.380
38.679	0.000	0.000	3.313	0.032	0.000	15.781	2.877	0.000	0.202	3.089	2.285	6.886	1.325	0.853	9.811
2.419	0.000	0.000	3.070	0.048	0.037	17.305	3.145	0.025	0.000	3.821	4.942	7.879	2.023	0.293	12.366
0.179	0.000	0.000	5.332	0.008	0.695	15.933	4.977	0.079	0.000	9.251	5.969	6.162	1.286	0.174	15.790
0.323	0.000	0.000	5.263	0.017	0.339	18.366	5.713	0.008	0.000	8.497	5.179	7.994	0.461	0.000	14.795
0.043	0.000	0.000	7.257	0.032	0.447	14.856	8.038	0.015	0.000	8.429	7.441	6.510	1.553	0.042	15.191
0.577	0.000	0.000	7.586	0.035	0.997	12.569	4.026	0.016	0.221	6.077	2.232	5.125	1.375	0.746	11.889
2.117	0.000	0.000	3.528	0.040	0.000	5.965	3.669	0.004	0.000	6.099	5.577	5.435	0.747	0.042	11.918
0.000	0.000	0.000	6.570	0.110	0.237	23.796	6.192	0.008	0.000	5.766	4.274	8.291	0.578	0.048	15.980
1.124	0.066	0.000	3.694	0.239	0.179	56.808	6.518	0.020	0.000	2.838	1.214	8.995	0.691	0.230	9.085
0.045	0.000	0.000	2.321	0.033	0.155	21.509	4.229	0.008	0.052	5.364	3.772	5.748	1.131	0.175	13.810
0.000	0.000	0.000	6.697	0.000	0.145	26.507	4.567	0.008	0.000	6.027	4.953	6.275	1.024	0.093	13.707
0.103	0.000	0.000	2.114	0.170	0.598	40.221	3.881	0.000	0.000	2.492	1.297	9.382	1.585	0.199	9.167
0.106	0.000	0.000	6.737	0.078	2.908	16.111	5.396	0.004	0.061	9.107	9.691	7.117	0.656	0.103	19.314
11.913	0.000	0.000	4.506	0.410	1.275	24.283	4.357	0.004	0.000	2.587	3.806	8.382	0.705	0.251	11.509
0.705	0.000	0.000	4.725	0.000	0.000	26.758	2.190	0.048	0.000	4.720	3.171	7.706	0.623	0.385	10.867
0.196	0.000	0.000	4.901	0.009	0.275	17.828	4.534	0.000	0.000	6.690	3.388	5.844	1.033	0.095	12.171
0.000	0.000	0.000	4.702	0.009	0.815	19.690	4.002	0.013	0.000	7.245	6.299	6.585	1.690	0.099	15.380
11.366	0.000	0.000	3.684	0.011	0.150	16.144	3.777	0.005	0.000	6.591	3.194	6.339	0.940	0.224	14.447
0.203	0.000	0.000	7.040	0.187	0.395	24.479	4.498	0.017	0.000	4.020	3.565	4.964	0.883	0.493	17.705
0.708	0.000	0.000	6.052	0.102	0.175	22.424	7.689	0.021	0.349	7.229	7.137	6.605	1.112	0.049	11.961
0.000	0.000	0.000	5.939	0.009	0.571	31.350	5.290	0.030	0.000	7.024	5.359	5.374	1.267	0.148	13.265
0.000	0.000	0.000	8.960	0.039	0.018	15.198	2.362	0.007	0.000	4.756	3.063	6.652	1.043	0.000	7.541
0.044	0.000	0.000	5.630	0.016	0.303	14.387	6.175	0.004	0.000	11.322	8.073	7.193	1.096	0.213	16.041
9.952	0.000	0.000	8.260	0.357	0.816	14.826	4.636	0.000	0.000	3.560	2.143	6.852	0.926	0.347	10.200
1.476	0.000	0.000	5.044	0.072	0.064	17.852	4.522	0.000	0.056	4.345	4.642	5.984	1.558	0.143	11.826
0.208	0.000	0.000	3.573	0.076	0.045	19.978	4.925	0.000	0.000	7.283	6.663	7.986	0.926	0.907	8.992
0.000	0.000	0.000	4.260	0.130	0.305	18.786	4.767	0.000	0.054	7.903	3.405	7.469	2.000	0.183	13.433
2.562	0.000	0.000	2.921	0.347	0.699	13.069	3.893	0.011	0.000	16.745	18.138	4.127	1.399	0.174	20.843
59.467	0.000	0.000	4.705	0.165	0.407	23.414	6.027	0.008	0.052	11.863	5.895	6.254	0.687	0.000	19.564
0.161	0.000	0.000	5.565	0.325	0.717	27.553	4.877	0.023	0.062	6.045	5.012	7.402	0.889	0.156	16.043
0.166	0.000	0.000	4.344	0.377	0.693	22.335	4.979	0.000	0.000	9.174	8.751	10.155	1.425	0.161	18.674
0.055	0.000	0.000	6.716	0.344	0.309	15.360	2.550	0.005	0.000	8.746	7.332	7.485	2.349	0.160	13.736
0.000	0.000	0.000	4.747	0.000	0.261	22.881	3.997	0.012	0.000	6.501	6.202	7.292	2.210	0.045	14.583
0.000	0.000	0.000	4.130	0.019	0.354	20.669	6.264	0.069	0.000	12.348	5.996	8.247	0.925	0.149	16.371
0.044	0.000	0.000	5.589	0.097	0.266	14.153	4.975	0.007	0.000	10.046	8.925	8.225	1.908	0.000	15.871
0.702	0.000	0.000	4.754	0.017	0.647	21.569	8.890	0.047	0.000	16.710	10.144	8.642	1.051	0.136	18.809
0.137	0.000	0.000	2.979	0.092	0.256	18.836	3.819	0.027	0.000	10.545	7.417	6.205	1.485	0.044	16.513
0.050	0.056	0.000	5.920	0.000	0.087	30.141	2.063	0.000	0.000	13.060	9.413	8.229	0.131	0.098	18.713
0.051	0.000	0.000	5.843	0.693	0.593	22.195	4.097	0.004	0.000	9.456	7.377	4.507	0.935	0.099	17.947
21.005	0.000	0.000	5.569	0.138	0.248	23.666	6.914	0.026	0.000	6.434	6.403	8.489	0.947	0.214	17.094
6.167	0.000	0.000	2.661	0.046	0.288	16.442	2.357	0.011	0.000	5.082	2.372	5.138	0.686	0.283	10.112
1.844	0.000	0.000	5.405	0.131	2.950	25.232	4.145	0.014	0.000	4.591	3.614	6.834	0.818	0.732	13.784
1.126	0.050	0.000	3.504	0.033	0.058	22.393	2.944	0.019	0.000	5.668	2.629	6.895	2.120	0.306	10.809
2.202	0.000	0.000	7.652	0.228	0.159	24.743	4.814	0.000	0.000	4.687	3.420	6.892	1.266	0.312	15.148
0.284	0.000	0.000	5.492	0.000	0.088	20.311	5.967	0.041	0.000	7.464	5.541	6.518	2.179	0.157	12.015
0.373	0.000	0.000	5.685	0.014	0.226	30.545	4.798	0.088	0.000	3.878	3.485	9.573	1.768	0.435	10.977
0.000	0.000	0.000	3.198	0.107	0.168	23.670	2.785	0.033	0.223	10.165	4.595	5.678	1.226	0.755	18.882
0.193	0.000	0.000	4.879	0.053	0.063	25.542	3.938	0.004	0.000	8.329	5.663	8.008	1.275	0.469	13.164
0.137	0.000	0.000	2.732	0.034	1.874	27.568	4.452	0.050	0.000	8.572	6.385	4.970	1.064	0.266	16.936
0.091	0.000	0.000	5.338	0.050	0.137	16.392	3.031	0.004	0.000	7.214	5.692	6.485	2.104	0.396	14.246
0.212	0.000	0.000	5.092	0.234	0.201	11.301	2.594	0.004	0.000	7.941	4.887	7.636	2.370	0.247	9.020
0.000	0.000	0.000	3.849	0.063	0.528	24.272	4.352	0.008	0.056	8.627	3.804	7.263	1.475	0.047	14.654
7.998	0.000	0.000	6.343	0.014	0.067	39.270	4.307	0.111	0.089	5.479	3.529	7.005	1.140	0.226	14.260
0.000	0.000	0.155	6.221	0.060	0.227	20.305	4.816	0.031	0.000	6.884	7.055	9.562	0.721	0.235	17.034
0.824	0.048	0.000	4.045	0.008	0.094	19.011	4.037	0.040	0.000	10.710	6.490	4.853	1.483	0.084	15.355
0.000	0.000	0.000	5.682	0.098	0.271	27.338	3.924	0.000	0.000	7.536	4.410	10.425	1.631	0.234	19.134
0.000	0.000	0.000	4.388	0.183	0.134	19.734	6.244	0.000	0.000	3.554	2.688	7.023	0.549	0.422	11.624
0.000	0.000	0.000	6.309	0.063	0.331	25.991	7.069	0.007	0.000	4.661	2.852	6.167	0.733	0.578	14.793
0.114	0.000	0.000	3.273	0.021	0.000	33.384	8.142	0.010	0.000	3.936	1.516	8.167	0.916	0.220	8.586
0.000	0.000	0.000	9.104	0.000	0.065	24.244	5.485	0.032	0.000	9.239	4.442	6.390	0.673	0.439	12.389
0.417	0.000	0.000	3.633	0.000	0.000	57.486	2.353	0.005	0.068	3.251	1.544	6.577	0.467	0.521	6.823
0.043	0.000	0.000	6.544	0.040	0.038	23.923	4.439	0.011	0.050	5.870	3.129	6.704	1.236	0.084	12.264
69.119	0.000	0.000	2.121	0.000	1.024	25.885	3.318	0.000	0.101	2.704	0.957	6.959	0.770	0.426	7.501
0.557	0.000	0.000	3.448	0.029	0.034	15.217	3.224	0.017	0.000	7.317	4.374	6.518	1.980	0.193	10.249
0.000	0.000	0.000	6.309	0.014	0.136	25.461	4.833	0.020	0.090	6.745	5.259	7.581	1.544	0.381	16.892
0.180	0.000	0.000	6.220	0.011	0.026	13.653	5.659	0.015	0.000	6.495	4.771	6.678	2.516	0.232	12.246
0.000	0.000	0.000	4.039	0.013	0.187	28.180	7.323	0.006	0.000	7.774	5.967	7.648	0.510	0.000	13.419
0.403	0.000	0.000	3.441	0.037	0.414	44.190	7.568	0.059	0.000	4.042	1.984	5.442	0.802	0.342	11.799
1.511	0.000	0.000	6.918	0.000	0.131	26.858	3.128	0.000	0.347	8.974	11.049	5.069	0.488	0.293	23.057
3.175	0.000	0.000	2.192	0.012	0.192	38.728	5.065	0.043	0.073	4.287	2.590	11.086	1.587	0.246	8.284
0.000	0.000	0.000	2.594	0.000	0.000	26.918	4.137	0.011	0.000	3.886	2.091	8.151	0.701	0.184	8.143
6.619	0.000	0.000	2.063	0.064	0.050	27.570	5.771	0.044	0.000	1.653	1.366	13.559	2.218	0.507	7.440
0.910	0.000	0.000	7.017	0.000	0.039	22.329	4.870	0.000	0.000	4.195	2.875	7.247	1.435	0.000	10.477
0.703	0.000	0.000	7.984	0.507	0.654	16.111	5.048	0.014	0.000	3.507	4.860	7.787	1.018	0.105	11.218
0.184	0.000	0.000	4.828	0.000	0.132	10.883	5.006	0.010	0.000	8.072	7.582	6.074	1.496	0.297	13.482
1.380	0.000	0.000	4.130	0.226	0.199	12.379	4.349	0.009	0.000	5.621	6.102	8.142	0.735	0.298	13.361
0.272	0.000	0.000	4.601	0.020	0.000	17.569	2.392	0.018	0.000	4.758	2.870	8.001	1.574	0.158	10.976
0.043	0.000	0.000	6.276	0.519	0.959	25.139	3.161	0.076	0.000	10.965	7.782	4.643	0.821	0.041	21.035
0.446	0.199	0.000	5.222	0.025	0.038	20.901	2.997	0.000	0.051	4.557	5.469	5.162	1.363	0.043	12.268
0.000	0.000	0.000	8.259	0.434	0.422	12.074	4.784	0.014	0.000	11.283	6.362	5.648	1.358	0.158	15.893
0.000	0.000	0.000	3.057	0.073	0.308	14.601	3.529	0.013	0.000	7.006	4.041	6.367	1.073	0.462	11.095
0.000	0.000	0.000	6.020	0.467	0.823	18.318	7.017	0.018	0.000	10.973	12.883	8.533	1.386	0.123	17.470
0.000	0.000	0.000	3.906	0.064	0.245	16.747	3.959	0.000	0.000	11.593	6.959	6.322	1.753	0.127	7.527
0.126	0.000	0.000	5.353	0.031	0.237	19.951	2.308	0.004	0.048	4.933	4.210	7.473	1.248	0.164	15.433
0.088	0.000	0.000	2.774	0.138	1.242	19.546	3.800	0.011	0.000	10.803	12.182	3.854	0.731	0.129	18.807
0.043	0.000	0.000	3.931	0.518	0.368	14.688	2.771	0.004	0.000	8.122	8.945	4.952	0.725	0.248	11.476
1.880	0.055	0.000	35.975	0.009	0.235	17.344	3.923	0.012	0.000	9.482	7.738	6.032	2.518	0.096	15.477
0.000	0.000	0.000	4.299	0.076	0.179	39.559	4.983	0.074	0.000	6.943	7.876	5.812	2.088	0.201	16.123
1.057	0.000	0.000	6.467	0.428	0.730	21.389	5.341	0.000	0.000	7.770	8.156	13.220	0.926	0.205	13.231
0.535	0.000	0.000	2.774	0.049	0.116	17.991	2.978	0.000	0.000	7.535	9.703	8.101	2.561	0.389	13.596
0.052	0.000	0.000	6.113	0.019	0.314	20.662	6.986	0.009	0.119	8.955	5.568	7.970	2.519	0.252	13.164
1.501	0.000	0.000	6.144	0.029	0.268	15.150	4.054	0.017	0.000	7.227	9.177	5.836	2.335	0.151	16.973
0.135	0.000	0.000	1.871	0.012	0.058	39.223	4.155	0.000	0.000	1.311	1.544	12.302	3.959	0.328	7.312
3.381	0.000	0.000	4.869	0.000	0.152	14.322	4.168	0.011	0.000	7.137	8.476	7.857	2.697	0.000	12.558
0.057	0.000	0.000	3.127	0.135	0.098	16.006	3.527	0.005	0.000	6.617	6.231	8.106	2.745	0.055	14.464
0.065	0.000	0.000	4.226	0.096	0.448	33.923	5.950	0.005	0.000	9.997	5.346	6.966	0.778	0.252	15.417
0.455	0.000	0.000	6.370	0.314	0.639	20.329	4.625	0.029	0.131	7.432	5.477	6.639	1.040	0.441	14.563
0.729	0.000	0.000	5.990	0.077	0.225	21.481	2.017	0.000	0.000	5.153	4.749	8.386	2.345	0.101	10.871
0.046	0.000	0.000	10.277	0.085	0.220	28.759	2.796	0.047	0.000	6.842	2.111	7.556	0.856	0.134	19.115
0.043	0.000	0.000	6.483	0.000	0.037	27.088	5.422	0.018	0.000	6.461	4.011	7.317	0.640	0.126	17.666
0.163	0.000	0.000	5.380	0.010	0.188	44.160	5.820	0.014	0.000	4.162	1.284	4.695	0.334	0.422	9.403
0.769	0.000	0.000	3.311	0.038	0.022	25.713	3.761	0.004	0.059	2.324	1.280	8.561	1.162	0.199	7.853
0.000	0.000	0.000	7.060	0.000	0.125	23.915	4.884	0.020	0.000	5.619	4.479	9.338	1.002	0.140	13.822
0.050	0.000	0.000	5.699	0.046	0.322	21.783	4.520	0.004	0.000	6.960	5.304	7.959	1.303	0.241	14.881
0.639	0.000	0.000	6.648	0.157	0.598	17.985	6.762	0.009	0.000	7.982	4.111	6.407	0.655	0.207	14.510
0.253	0.000	0.000	5.651	0.000	0.284	26.581	3.516	0.060	0.000	8.763	5.009	5.757	1.415	0.098	14.879
0.966	0.000	0.000	4.548	0.010	0.093	15.257	4.335	0.009	0.247	5.581	2.881	6.011	1.073	0.313	10.033
0.046	0.052	0.000	2.834	0.043	0.580	14.654	5.801	0.023	0.000	8.453	5.877	8.405	0.733	0.270	14.747
0.268	0.000	0.000	5.594	0.217	0.811	21.190	4.658	0.000	0.000	6.808	5.662	7.783	0.973	0.260	17.165
1.535	0.000	0.000	5.224	0.017	0.181	33.745	6.718	0.063	0.000	6.560	3.738	7.369	0.934	0.226	15.948
0.044	0.000	0.000	6.083	0.000	0.153	22.787	5.895	0.026	0.051	7.494	5.774	6.941	0.905	0.258	17.556
65.693	0.000	0.153	5.335	0.110	0.398	20.348	3.685	0.003	0.000	5.500	5.436	7.102	1.979	0.039	16.376
0.060	0.000	0.000	4.139	0.055	0.156	25.535	4.083	0.061	0.000	5.550	3.302	4.994	1.108	0.292	17.320
35.700	0.000	0.000	16.855	0.000	0.000	19.899	0.616	0.009	0.000	7.317	3.185	2.466	0.162	0.051	11.273
0.420	0.000	0.000	2.957	0.000	0.023	27.554	3.944	0.004	0.000	5.202	2.903	9.598	1.701	0.458	11.721
0.000	0.000	0.000	7.164	0.045	0.787	19.785	8.535	0.021	0.170	10.650	7.456	6.087	1.207	0.382	13.794
0.516	0.000	0.000	2.974	0.112	3.021	16.592	3.474	0.000	0.000	5.909	3.363	4.890	0.967	0.228	12.415
0.000	0.000	0.000	4.470	0.000	0.463	21.019	5.764	0.013	0.000	7.867	5.168	6.465	1.306	0.594	13.247
0.081	0.000	0.000	6.108	0.015	0.367	19.303	4.810	0.020	0.000	9.695	6.901	5.249	0.865	0.196	14.171
0.038	0.000	0.000	5.708	0.055	0.130	19.920	2.795	0.009	0.000	6.655	5.341	5.555	2.841	0.255	12.705
0.122	0.000	0.000	8.261	0.045	0.509	11.771	4.998	0.010	0.000	7.373	7.885	7.726	1.392	0.236	18.628
0.077	0.000	0.000	5.339	0.007	0.100	15.395	6.750	0.010	0.000	9.466	6.275	7.933	1.467	0.225	14.481
1.095	0.000	0.000	4.923	0.022	0.053	17.517	3.470	0.000	0.000	3.617	2.118	7.324	1.300	0.472	9.460
0.044	0.000	0.000	3.685	0.032	0.057	28.803	3.914	0.004	0.050	5.549	2.638	7.783	0.896	0.427	11.938
0.000	0.000	0.000	10.167	0.280	1.146	28.640	5.286	0.033	0.000	8.014	5.283	5.968	0.799	0.219	14.776
0.058	0.000	0.000	5.230	0.021	0.174	26.075	6.444	0.039	0.066	7.292	5.078	6.388	0.579	0.000	11.727
2.107	0.000	0.000	5.604	0.000	0.221	12.321	5.410	0.027	0.147	6.423	5.651	6.259	2.062	0.248	15.676
0.932	0.000	0.000	4.290	0.106	0.372	11.602	4.833	0.042	0.000	7.275	7.245	6.490	1.324	0.418	13.743
0.291	0.000	0.000	6.968	0.461	1.912	21.965	6.427	0.000	0.000	8.592	5.875	6.944	0.694	0.452	18.840
0.041	0.000	0.000	5.394	0.000	0.210	28.824	2.700	0.003	0.000	9.340	3.121	5.887	1.367	0.158	16.909
0.042	0.000	0.000	6.430	0.145	0.413	17.479	4.316	0.011	0.000	7.462	5.825	7.115	1.366	0.323	15.213
0.074	0.000	0.000	6.029	0.041	0.289	19.496	3.378	0.000	0.000	5.603	4.038	4.444	1.011	0.288	13.524
0.077	0.000	0.000	5.271	0.014	0.067	26.493	4.001	0.036	0.000	6.383	3.560	5.102	1.024	0.300	12.534
0.000	0.000	0.000	11.255	0.058	0.068	17.297	2.700	0.004	0.060	7.624	5.802	6.458	1.116	0.305	14.313
0.283	0.000	0.000	8.376	0.260	1.200	23.342	6.315	0.012	0.000	11.164	7.465	5.800	1.112	0.320	18.037
0.092	0.000	0.000	7.585	0.009	0.140	12.237	4.663	0.016	0.000	6.739	3.894	5.366	1.031	0.449	14.336

ENSG00000222536	ENSG00000234287	ENSG00000258449	ENSG00000273821	ENSG00000264089	ENSG00000183324	ENSG00000164576	ENSG00000279187	ENSG00000122188	ENSG00000079841	ENSG00000080618	ENSG00000163492	ENSG00000169255	ENSG00000254180	ENSG00000230107	ENSG00000114125
0.000	5.047	0.051	0.214	0.000	0.000	10.043	0.060	0.077	0.503	0.028	0.042	13.721	0.043	0.216	16.657
0.000	18.154	0.000	0.137	0.000	0.000	5.663	0.000	0.059	0.138	0.036	0.010	10.705	0.146	0.258	23.784
0.000	19.538	0.038	0.446	0.000	0.000	5.313	0.038	0.041	0.289	0.053	0.084	15.761	0.032	0.229	21.248
0.000	10.401	0.043	0.000	0.000	0.069	5.999	0.000	0.053	0.994	0.216	0.110	10.865	0.000	0.185	13.653
0.000	13.420	0.000	0.460	0.000	0.025	7.410	0.000	0.103	0.026	0.027	0.018	8.678	0.122	0.413	26.143
0.000	8.485	0.076	0.444	0.000	0.000	7.809	0.331	0.064	0.527	0.243	0.117	10.965	0.065	0.098	12.269
0.000	8.895	0.000	0.773	0.000	0.045	6.983	0.034	0.315	0.033	0.048	0.039	8.716	0.036	0.698	25.793
0.000	10.454	0.000	0.337	0.000	0.000	5.614	0.107	0.049	0.961	0.063	0.118	11.259	0.077	0.039	19.285
0.000	16.107	0.157	0.722	0.000	0.028	6.719	0.021	0.024	0.604	0.189	0.066	12.286	0.178	0.718	26.983
0.000	24.321	0.000	0.485	0.000	0.000	3.473	0.090	0.280	0.076	0.085	0.019	5.841	0.000	0.653	32.481
0.000	9.792	0.000	0.437	0.000	0.040	4.335	0.030	0.126	0.109	0.042	0.033	9.314	0.032	0.417	27.560
0.000	7.751	0.039	0.329	1.056	0.021	6.178	0.038	0.475	0.504	0.065	0.036	11.858	0.066	0.498	15.248
0.000	5.844	0.040	0.722	0.000	0.042	5.989	0.032	0.153	1.211	0.133	0.123	11.195	0.102	0.137	13.067
0.000	6.113	0.037	0.397	0.000	0.000	9.719	0.096	0.040	0.305	0.031	0.050	11.289	0.190	0.385	14.371
0.413	35.574	0.000	0.177	0.000	0.088	6.908	0.000	0.128	0.677	0.046	0.037	7.561	0.000	0.143	14.698
0.000	7.343	0.075	0.278	0.000	0.000	8.148	0.074	0.465	0.106	0.042	0.050	4.503	0.032	0.225	13.889
0.214	8.053	0.000	0.275	0.000	0.000	7.503	0.043	0.000	1.346	0.072	0.051	15.734	0.256	0.111	14.914
0.000	14.980	0.051	0.544	0.000	0.027	3.296	0.000	0.016	0.100	0.057	0.015	11.397	0.000	0.351	26.265
0.000	12.599	0.000	0.331	0.000	0.000	4.524	0.031	0.066	0.067	0.022	0.037	7.040	0.232	0.234	16.152
0.000	5.692	0.000	0.354	0.000	0.000	9.088	0.033	0.026	0.008	0.046	0.054	11.945	0.318	0.107	14.032
0.000	15.384	0.000	0.472	0.000	0.024	3.610	0.053	0.034	0.326	0.062	0.021	22.493	0.189	0.191	34.129
0.000	7.156	0.046	0.146	0.000	0.000	12.234	0.354	0.028	3.265	0.051	0.127	7.794	0.039	0.039	11.841
0.000	13.467	0.090	0.286	0.000	0.024	4.598	0.035	0.145	0.716	0.075	0.038	14.909	0.152	0.115	24.070
0.000	8.666	0.000	0.486	0.000	0.000	4.766	0.015	0.100	0.048	0.000	0.008	13.494	0.032	0.033	26.369
0.000	11.447	0.000	0.451	0.000	0.022	6.821	0.017	0.072	0.308	0.035	0.031	6.540	0.180	0.255	20.446
0.000	5.101	0.088	0.375	0.000	0.000	8.361	0.061	0.054	1.448	0.098	0.059	11.869	0.000	0.872	12.717
0.000	16.635	0.100	0.690	0.000	0.000	6.214	0.020	0.238	0.272	0.153	0.048	9.666	0.085	0.171	22.658
0.000	20.487	0.000	0.467	0.000	0.000	4.570	0.104	0.014	0.797	0.024	0.048	9.702	0.224	0.302	20.168
0.000	7.673	0.088	0.279	0.000	0.000	6.284	0.087	0.202	0.249	0.207	0.049	7.335	0.223	0.150	17.471
0.000	15.095	0.000	0.328	0.000	0.000	4.085	0.157	0.183	0.259	0.061	0.067	10.831	0.262	0.189	22.409
0.000	13.533	0.000	0.693	0.000	0.038	8.611	0.086	0.033	1.066	0.091	0.021	15.611	0.062	0.187	37.635
0.000	7.895	0.076	0.202	0.000	0.000	6.140	0.113	0.035	0.137	0.063	0.012	14.011	0.129	0.065	13.174
0.000	11.823	0.044	0.282	0.000	0.023	6.653	0.017	0.027	0.575	0.135	0.038	8.426	0.225	0.569	20.835
0.000	11.395	0.085	1.403	0.000	0.045	6.053	0.051	0.235	0.129	0.071	0.007	8.595	0.072	0.256	21.993
0.000	12.638	0.000	0.764	0.000	0.000	7.581	0.125	0.041	0.066	0.075	0.029	16.317	0.076	0.309	17.999
0.000	7.293	0.082	1.125	0.000	0.043	6.972	0.081	0.081	0.757	0.034	0.054	9.681	0.138	0.454	18.199
0.000	6.816	0.039	1.158	0.000	0.062	10.713	0.077	0.125	0.408	0.022	0.215	9.675	0.000	0.267	8.982
0.000	7.480	0.000	0.826	0.000	0.041	7.474	0.046	0.644	0.124	0.119	0.111	11.222	0.033	0.367	18.068
0.000	8.567	0.046	0.443	0.000	0.000	7.253	0.073	0.057	0.870	0.077	0.177	16.624	0.000	0.278	21.410
0.000	6.085	0.000	0.407	0.000	0.000	5.374	0.057	0.059	0.474	0.160	0.053	12.224	0.244	0.123	14.806
0.000	5.596	0.095	0.405	0.000	0.025	6.628	0.075	0.132	0.554	0.079	0.030	12.443	0.121	0.777	17.245
0.000	5.970	0.000	0.984	0.000	0.085	7.905	0.032	0.037	0.196	0.067	0.036	15.540	0.068	0.277	18.206
0.000	8.532	0.000	0.283	0.000	0.047	7.553	0.053	0.048	0.248	0.049	0.069	8.129	0.226	0.304	17.283
0.000	5.272	0.076	0.566	0.000	0.020	7.933	0.090	0.105	0.222	0.085	0.087	13.704	0.097	0.294	12.276
0.000	6.789	0.122	0.517	72.407	0.021	11.727	0.080	0.106	0.114	0.135	0.173	15.289	0.310	0.035	13.955
0.000	4.947	0.158	0.630	0.000	0.000	7.453	0.070	0.085	0.326	0.022	0.340	7.804	0.034	0.102	12.808
0.000	26.316	0.000	0.370	0.000	0.069	6.011	0.121	0.027	0.401	0.036	0.014	11.996	0.037	0.224	12.353
0.000	9.754	0.088	0.047	0.000	0.000	8.008	0.096	0.041	0.868	0.061	0.062	8.573	0.075	0.151	19.683
0.000	14.195	0.000	0.325	0.000	0.000	7.357	0.015	0.029	0.324	0.021	0.034	14.809	0.195	0.066	17.676
0.000	19.723	0.036	0.460	0.000	0.038	4.952	0.057	0.133	0.222	0.020	0.048	5.516	0.092	0.062	25.875
0.000	14.457	0.073	0.385	0.000	0.000	7.472	0.036	0.368	2.675	0.050	0.033	32.248	0.154	0.311	26.757
0.097	19.976	0.039	1.078	0.000	0.000	5.715	0.023	0.072	0.138	0.000	0.151	12.608	0.133	0.335	28.599
0.000	18.122	0.040	0.464	0.000	0.063	6.146	0.071	0.171	0.500	0.066	0.050	21.988	0.135	0.068	28.892
0.000	12.435	0.000	0.298	0.000	0.000	7.939	0.035	0.027	0.007	0.039	0.064	15.878	0.089	0.211	19.369
0.000	26.136	0.000	0.756	0.000	0.000	6.949	0.038	0.069	0.844	0.036	0.018	24.355	0.220	0.499	35.549
0.000	53.491	0.000	0.358	0.000	0.000	9.558	0.167	0.026	0.310	0.094	0.091	18.794	0.286	0.000	20.779
0.000	13.134	0.000	0.178	0.000	0.000	6.716	0.124	0.039	0.116	0.047	0.016	11.013	0.107	0.144	27.226
0.000	18.349	0.079	0.756	0.000	0.000	10.267	0.047	0.085	0.235	0.055	0.082	14.606	0.168	0.034	18.564
0.000	11.574	0.039	0.960	0.536	0.021	8.427	0.155	0.030	0.543	0.033	0.057	14.079	0.334	0.304	14.656
0.000	13.268	0.037	0.195	0.000	0.000	12.844	0.007	0.197	0.531	0.031	0.190	5.060	0.125	0.346	10.483
0.000	14.520	0.085	1.306	0.000	0.022	6.174	0.126	0.078	0.298	0.082	0.182	10.587	0.216	0.182	12.056
0.000	20.448	0.202	0.286	0.000	0.071	5.262	0.053	0.093	0.418	0.037	0.011	15.045	0.000	0.288	28.451
0.000	7.277	0.035	0.149	0.000	0.000	7.389	0.152	0.021	1.804	0.068	0.053	11.887	0.327	0.120	12.279
0.000	9.754	0.113	0.319	0.256	0.000	8.864	0.134	0.092	0.135	0.031	0.156	8.822	0.064	0.161	17.920
0.000	11.516	0.042	0.622	0.000	0.000	7.588	0.041	0.064	0.254	0.070	0.109	19.735	0.107	0.000	22.283
0.000	34.864	0.000	0.400	0.000	0.000	6.901	0.000	0.000	0.380	0.075	0.039	11.270	0.046	0.000	19.356
0.000	14.900	0.074	0.470	0.000	0.000	6.053	0.044	0.023	0.634	0.041	0.037	17.457	0.250	0.063	19.018
0.000	19.868	0.000	0.104	0.000	0.000	5.171	0.019	0.000	0.019	0.014	0.012	15.849	0.083	0.000	23.527
0.000	12.578	0.000	0.208	0.000	0.000	10.460	0.039	0.080	0.018	0.073	0.083	14.109	0.055	0.056	15.645
0.000	19.275	0.000	0.055	0.000	0.000	4.958	0.010	0.040	0.158	0.000	0.043	18.867	0.132	0.177	35.295
0.000	18.031	0.000	0.200	0.000	0.020	6.676	0.007	0.023	0.092	0.031	0.013	18.214	0.032	0.129	25.660
0.000	14.345	0.000	0.646	0.000	0.040	4.354	0.015	0.268	0.359	0.127	0.024	14.607	0.129	0.261	38.192
0.085	14.597	0.000	0.842	0.000	0.018	3.627	0.055	0.111	0.087	0.010	0.023	7.256	0.000	0.325	18.672
0.000	14.513	0.000	1.660	0.000	0.108	8.233	0.054	0.083	0.162	0.000	0.013	10.792	0.000	0.175	21.560
0.000	9.973	0.000	0.165	0.000	0.000	8.343	0.031	0.119	0.060	0.000	0.010	5.946	0.132	0.133	14.191
0.000	7.811	0.063	0.398	0.000	0.000	6.483	0.049	0.057	0.137	0.035	0.083	12.711	0.106	0.161	14.831
0.000	21.924	0.000	0.139	0.000	0.000	3.903	0.009	0.060	0.090	0.036	0.028	15.597	0.111	0.225	32.181
0.000	48.362	0.000	0.556	1.784	0.277	4.013	0.000	0.241	0.844	0.073	0.075	7.278	0.000	0.000	19.267
0.000	15.662	0.000	0.175	0.375	0.029	5.385	0.011	0.068	0.173	0.122	0.014	27.875	0.187	0.236	36.049
0.000	16.356	0.000	0.582	0.000	0.029	6.035	0.011	0.093	0.021	0.015	0.098	10.666	0.233	0.235	30.124
0.000	18.289	0.000	0.320	0.000	0.000	3.847	0.010	0.154	0.021	0.140	0.010	33.825	0.043	0.043	61.230
0.000	14.112	0.000	0.293	0.000	0.021	7.879	0.031	0.042	0.133	0.066	0.088	19.319	0.000	0.068	27.708
0.000	7.478	0.047	0.249	0.000	0.000	5.285	0.074	0.172	0.390	0.078	0.049	7.431	0.079	0.442	19.193
0.000	8.566	0.053	0.225	0.000	0.028	9.027	0.031	0.154	0.191	0.044	0.120	14.081	0.090	0.227	20.782
0.000	6.304	0.089	0.611	0.000	0.023	9.243	0.166	0.082	0.810	0.049	0.032	6.393	0.188	0.114	13.330
0.000	5.160	0.000	0.450	0.000	0.000	5.409	0.046	0.029	0.065	0.026	0.015	7.107	0.000	0.161	17.412
0.000	7.048	0.037	0.314	0.000	0.000	11.787	0.132	0.023	0.429	0.226	0.014	8.788	0.063	0.095	13.388
0.000	9.282	0.000	1.189	0.000	0.041	8.073	0.038	0.036	0.083	0.000	0.335	10.493	0.131	0.199	11.017
0.000	10.784	0.071	0.524	0.000	0.075	6.508	0.014	0.043	0.480	0.029	0.047	13.988	0.060	0.151	13.751
0.170	17.968	0.069	0.292	0.000	0.036	7.421	0.109	0.074	0.240	0.038	0.052	8.127	0.058	0.118	14.035
0.000	5.502	0.000	0.350	0.000	0.019	8.827	0.196	0.039	0.789	0.244	0.089	8.522	0.031	0.252	17.861
0.000	13.035	0.000	0.764	0.000	0.000	4.077	0.060	0.087	0.164	0.053	0.065	9.469	0.064	0.195	20.688
0.000	8.573	0.000	0.621	0.000	0.000	5.420	0.072	0.034	0.137	0.061	0.276	4.361	0.031	0.094	19.559
0.000	17.915	0.077	0.651	0.000	0.000	8.667	0.083	0.053	0.919	0.106	0.101	5.389	0.098	0.000	11.052
0.000	19.261	0.000	0.314	0.000	0.000	8.556	0.146	0.045	0.473	0.154	0.134	7.469	0.094	0.095	14.484
0.000	10.966	0.086	1.411	0.000	0.045	6.184	0.161	0.092	0.135	0.012	0.117	9.228	0.182	0.000	15.286
0.000	17.968	0.000	0.715	0.000	0.000	4.252	0.027	0.110	0.169	0.062	0.034	11.485	0.000	0.231	22.014
0.000	7.397	0.000	0.583	0.000	0.000	7.021	0.181	0.028	1.798	0.458	0.029	11.746	0.000	0.157	18.239
0.000	18.729	0.000	3.692	0.000	0.000	5.437	0.137	0.107	0.236	0.161	0.077	9.501	0.295	0.596	17.001
0.000	13.588	0.135	0.382	0.000	0.000	7.240	0.009	0.055	0.174	0.037	0.027	11.866	0.305	0.270	15.534
0.000	8.107	0.045	0.904	0.000	0.000	9.406	0.080	0.179	0.177	0.062	0.075	11.204	0.076	0.077	16.507
0.000	16.669	0.000	1.181	0.000	0.000	3.482	0.035	0.036	0.027	0.081	0.041	25.136	0.199	0.954	44.728
0.000	5.488	0.533	0.687	0.000	0.000	6.337	0.060	0.111	0.085	0.021	0.037	6.499	0.161	0.391	18.629
0.000	7.168	0.049	0.312	0.000	0.000	9.336	0.058	0.211	0.532	0.014	0.240	18.365	0.042	0.378	18.542
0.000	8.653	0.113	0.358	0.000	0.030	5.839	0.011	0.034	0.559	0.094	0.086	11.664	0.000	0.000	16.941
0.244	13.836	0.000	0.628	0.671	0.000	7.419	0.078	0.091	0.170	0.027	0.025	11.415	0.084	0.084	17.523
0.000	5.119	0.135	1.197	0.000	0.000	5.208	0.036	0.125	0.780	0.025	0.070	12.149	0.077	0.193	21.315
0.000	13.938	0.040	1.658	0.000	0.000	5.256	0.016	0.068	0.131	0.044	0.052	10.989	0.034	0.172	27.099
0.000	7.373	0.000	0.958	0.000	0.000	7.736	0.082	0.017	0.130	0.042	0.023	16.829	0.064	0.032	22.967
0.000	15.125	0.000	0.300	0.000	0.000	4.168	0.009	0.007	0.067	0.026	0.018	9.541	0.040	0.242	38.000
0.000	13.750	0.000	1.697	0.302	0.047	4.178	0.000	0.054	0.007	0.012	0.024	14.551	0.226	0.076	26.988
0.000	9.698	0.042	0.177	0.284	0.000	9.703	0.016	0.013	0.035	0.127	0.024	14.766	0.141	0.179	18.120
0.000	4.720	0.000	0.503	0.000	0.000	6.434	0.026	0.092	0.623	0.012	0.049	11.667	0.110	0.222	14.729
0.000	11.885	0.000	0.783	0.000	0.049	6.609	0.036	0.085	0.283	0.026	0.041	8.813	0.078	0.395	17.363
0.000	13.482	0.044	0.605	0.000	0.000	6.502	0.026	0.081	0.017	0.036	0.004	12.667	0.186	0.000	19.336
0.000	14.138	0.047	0.938	0.000	0.000	5.763	0.037	0.050	0.018	0.026	0.024	9.398	0.079	0.120	21.193
0.000	8.259	0.000	0.043	0.000	0.000	11.830	0.024	0.062	1.438	0.178	0.048	13.763	0.034	0.034	16.224
0.000	20.644	0.047	0.296	0.000	0.000	9.756	0.018	0.064	1.062	0.039	0.465	21.961	0.079	0.080	18.313
0.000	20.316	0.000	0.685	0.000	0.021	6.484	0.016	0.049	0.116	0.011	0.040	19.698	0.205	0.138	22.790
0.000	13.055	0.038	0.285	0.000	0.000	7.885	0.030	0.035	0.015	0.021	0.040	9.739	0.195	0.000	16.961
0.000	11.142	0.069	0.700	0.000	0.000	7.571	0.041	0.239	0.593	0.010	0.094	14.126	0.059	0.208	16.392
0.000	19.991	0.000	0.111	0.000	0.028	7.475	0.000	0.032	0.361	0.043	0.010	12.181	0.044	0.269	27.424
0.000	31.158	0.000	0.194	0.000	0.048	7.203	0.009	0.414	0.176	0.000	0.025	11.101	0.039	0.275	15.241
0.000	17.916	0.000	0.917	0.000	0.000	7.199	0.027	0.474	0.378	0.151	0.193	12.105	0.116	0.273	26.394
0.000	9.188	0.085	0.724	0.000	0.045	8.086	0.126	0.052	0.396	0.047	0.137	10.797	0.181	0.146	13.833
0.000	16.351	0.204	0.302	0.000	0.043	5.954	0.048	0.100	1.367	0.045	0.052	10.950	0.035	0.244	19.157
0.000	26.781	0.000	0.282	0.000	0.070	5.218	0.000	0.142	0.193	0.074	0.109	13.791	0.225	0.114	25.275
0.000	13.284	0.175	0.447	0.000	0.037	8.431	0.083	0.134	0.268	0.088	0.020	10.034	0.178	0.240	16.064
0.000	9.193	0.033	1.243	0.000	0.017	5.708	0.026	0.075	0.200	0.018	0.012	9.775	0.000	0.502	14.442
0.000	13.061	0.000	0.971	0.000	0.019	7.288	0.132	0.076	0.545	0.029	0.172	17.760	0.030	0.211	16.072
0.000	10.753	0.067	0.284	0.000	0.018	8.634	0.040	0.062	0.108	0.000	0.055	9.483	0.256	0.316	14.181
0.000	16.425	0.000	0.560	0.000	0.000	4.876	0.042	0.534	0.097	0.000	0.007	13.549	0.179	0.000	23.333
0.000	55.311	0.038	0.202	0.000	0.020	5.083	0.000	0.006	0.075	0.074	0.042	21.754	0.162	0.261	28.642
0.000	17.315	0.000	1.299	0.000	0.000	6.512	0.184	0.023	0.865	0.081	0.040	14.296	0.415	0.210	20.512
0.000	13.613	0.000	0.635	0.000	0.000	3.767	0.049	0.061	0.034	0.014	0.043	9.444	0.085	0.000	20.189
0.000	17.030	0.055	0.294	0.000	0.000	5.231	0.055	0.161	0.096	0.031	0.033	11.725	0.047	0.095	20.872
0.000	10.401	0.000	0.396	0.000	0.000	6.029	0.061	0.219	0.486	0.035	0.022	11.835	0.105	0.213	20.972
0.000	34.754	0.000	0.429	0.000	0.000	8.414	0.190	0.015	1.510	0.084	0.083	13.730	0.086	0.130	17.101
0.000	17.586	0.000	0.821	0.000	0.000	5.674	0.035	0.022	0.158	0.068	0.019	15.879	0.030	0.090	21.770
0.000	16.507	0.180	0.613	0.000	0.038	10.284	0.107	0.100	0.541	0.040	0.060	14.190	0.031	0.278	13.527
0.159	21.786	0.000	0.820	0.000	0.034	7.121	0.064	0.069	0.109	0.000	0.059	9.274	0.109	0.221	18.606
0.000	25.288	0.000	0.142	0.000	0.000	5.735	0.027	0.057	0.076	0.019	0.012	12.800	0.171	0.086	26.460
0.000	8.125	0.182	2.797	0.000	0.024	6.702	0.054	0.265	0.145	0.013	0.082	9.533	0.000	0.117	14.279
0.000	12.798	0.000	0.477	0.000	0.000	7.416	0.073	0.038	0.168	0.113	0.014	12.177	0.104	0.105	16.764
0.000	24.510	0.040	0.383	0.000	0.021	5.746	0.024	0.025	0.017	0.056	0.022	8.002	0.170	0.069	17.181

ENSG00000165516	ENSG00000244537	ENSG00000244060	ENSG00000213638	ENSG00000279204	ENSG00000271779	ENSG00000048392	ENSG00000235429	ENSG00000227892	ENSG00000109854	ENSG00000137273	ENSG00000255326	ENSG00000227107	ENSG00000239620	ENSG00000263382	ENSG00000187833
4.797	0.000	0.000	1.084	1.283	0.000	8.404	0.000	0.000	4.876	1.935	0.456	0.217	0.000	0.000	0.000
4.995	0.000	0.000	1.308	0.348	0.083	10.616	0.052	0.000	3.558	2.136	0.291	0.061	0.000	0.000	0.000
6.858	0.123	0.031	1.273	0.720	0.000	5.695	0.000	0.000	4.460	9.857	0.259	0.091	0.000	0.000	0.000
5.819	0.000	0.070	1.489	0.446	0.084	6.287	0.000	0.000	1.274	4.958	0.163	0.041	0.035	0.000	0.000
7.228	0.000	0.000	0.433	0.137	0.187	15.387	0.000	0.000	13.197	0.604	0.725	0.069	0.000	0.000	0.000
6.246	0.000	0.093	2.018	0.233	0.074	6.495	0.000	0.000	3.201	0.785	0.860	0.190	0.000	0.029	0.012
3.512	0.000	0.070	1.361	1.295	0.000	16.391	0.000	0.000	6.795	2.660	0.323	0.102	0.000	0.000	0.000
5.332	0.000	0.000	0.785	0.268	0.000	14.239	0.000	0.000	8.498	1.747	0.546	0.086	0.000	0.000	0.000
5.103	0.000	0.128	0.815	1.032	0.101	8.296	0.000	0.000	8.040	7.336	0.789	0.037	0.000	0.000	0.008
5.527	0.000	0.124	1.102	0.701	0.000	13.441	0.184	0.000	23.696	2.221	0.918	0.073	0.000	0.000	0.000
3.787	0.000	0.183	0.525	0.451	0.000	21.907	0.000	0.020	20.841	2.057	0.592	0.080	0.000	0.000	0.000
4.803	0.000	0.158	1.240	0.450	0.000	8.235	0.000	0.000	4.946	4.988	0.438	0.129	0.032	0.000	0.006
5.747	0.000	0.033	0.859	0.447	0.078	11.501	0.000	0.021	1.458	9.935	0.241	0.038	0.000	0.000	0.000
6.292	0.000	0.030	1.697	0.303	0.145	9.143	0.000	0.000	2.380	5.199	0.704	0.036	0.000	0.000	0.000
4.336	0.000	0.136	1.279	0.183	0.000	7.498	0.000	0.000	3.068	3.401	0.377	0.040	0.000	0.000	0.000
3.939	0.000	0.000	1.034	0.386	0.145	23.622	0.000	0.000	10.921	2.216	0.761	0.045	0.000	0.000	0.000
5.344	0.000	0.035	1.420	0.378	0.000	6.830	0.000	0.000	4.942	5.237	0.293	0.062	0.000	0.000	0.000
12.821	0.000	0.000	1.146	0.371	0.000	5.042	0.000	0.000	6.092	2.390	0.425	0.000	0.000	0.000	0.000
5.471	0.000	0.000	1.667	0.530	0.000	22.826	0.000	0.000	5.511	5.605	0.147	0.000	0.000	0.000	0.000
4.211	0.000	0.034	0.813	0.210	0.081	7.694	0.000	0.000	1.360	5.590	0.126	0.050	0.000	0.000	0.007
6.228	0.000	0.036	0.740	0.341	0.000	9.962	0.108	0.000	5.688	2.860	0.502	0.011	0.000	0.000	0.000
8.335	0.000	0.112	1.269	0.101	0.089	6.372	0.000	0.000	2.312	1.751	1.280	0.230	0.000	0.000	0.000
6.844	0.000	0.037	0.994	0.649	0.000	7.352	0.000	0.000	7.650	1.872	0.440	0.021	0.000	0.000	0.000
2.672	0.000	0.000	0.661	1.372	0.000	9.884	0.000	0.000	12.019	6.714	0.374	0.064	0.000	0.000	0.006
4.267	0.027	0.000	1.948	0.512	0.000	9.591	0.000	0.000	6.098	4.595	0.352	0.041	0.035	0.000	0.000
3.331	0.000	0.036	1.909	0.484	0.171	11.645	0.000	0.000	5.201	3.658	0.799	0.253	0.000	0.000	0.000
4.086	0.000	0.081	0.789	0.581	0.000	7.385	0.000	0.000	12.396	2.455	0.301	0.048	0.000	0.000	0.000
5.699	0.000	0.072	3.879	0.733	0.000	5.268	0.000	0.000	9.231	6.969	0.133	0.063	0.072	0.000	0.000
6.704	0.028	0.143	1.230	0.423	0.085	4.484	0.000	0.000	6.742	6.263	0.462	0.052	0.000	0.034	0.000
7.179	0.000	0.430	1.750	0.570	0.000	5.122	0.000	0.000	1.701	9.878	0.399	0.021	0.000	0.000	0.000
4.064	0.000	0.059	1.543	0.668	0.000	6.419	0.000	0.000	17.044	12.331	0.601	0.086	0.030	0.000	0.000
7.232	0.000	0.155	1.217	0.108	0.074	8.319	0.000	0.000	12.763	1.960	0.372	0.072	0.000	0.000	0.000
5.177	0.000	0.036	1.542	0.184	0.000	5.559	0.000	0.000	4.381	2.410	0.766	0.032	0.000	0.000	0.000
3.184	0.000	0.035	1.093	1.121	0.000	7.558	0.000	0.000	7.033	4.129	0.321	0.112	0.000	0.000	0.000
3.737	0.000	0.000	0.553	0.493	0.000	13.337	0.000	0.000	6.787	3.270	0.746	0.064	0.000	0.000	0.000
6.261	0.000	0.000	0.670	0.268	0.079	20.866	0.000	0.000	11.818	2.195	0.860	0.097	0.000	0.000	0.000
12.787	0.000	0.127	1.110	0.094	0.075	10.706	0.000	0.000	1.557	4.856	1.819	0.223	0.000	0.030	0.006
5.189	0.000	0.063	0.810	0.256	0.377	10.661	0.000	0.000	5.769	3.018	0.410	0.111	0.000	0.000	0.000
7.770	0.000	0.075	0.824	0.498	0.000	10.721	0.000	0.000	3.718	13.027	0.769	0.166	0.000	0.000	0.000
5.712	0.000	0.117	0.715	0.294	0.000	7.839	0.000	0.000	2.513	5.138	0.542	0.114	0.000	0.000	0.000
3.269	0.000	0.039	0.669	0.157	0.000	21.743	0.000	0.000	6.758	1.840	0.647	0.045	0.000	0.000	0.000
4.575	0.000	0.000	0.495	0.663	0.078	14.318	0.000	0.000	8.970	5.849	0.729	0.240	0.000	0.000	0.006
6.017	0.000	0.036	0.603	0.632	0.086	21.909	0.000	0.000	2.318	11.937	0.401	0.032	0.000	0.000	0.000
5.355	0.000	0.000	0.693	0.242	0.074	21.395	0.000	0.000	3.118	8.640	0.688	0.045	0.000	0.000	0.000
7.013	0.000	0.099	1.032	0.667	0.157	16.996	0.000	0.000	1.209	7.597	1.039	0.058	0.000	0.000	0.000
6.797	0.000	0.000	0.642	0.208	0.000	11.145	0.000	0.000	8.874	3.124	0.865	0.075	0.000	0.000	0.000
10.450	0.000	0.284	2.025	0.812	0.084	6.579	0.000	0.023	3.247	49.144	0.886	0.073	0.036	0.000	0.000
7.866	0.028	0.108	1.702	0.503	0.000	5.754	0.053	0.000	3.271	7.091	0.399	0.042	0.000	0.000	0.007
3.709	0.000	0.000	0.956	0.260	0.000	8.045	0.000	0.000	6.095	3.559	0.346	0.064	0.000	0.000	0.000
4.528	0.023	0.000	1.282	0.332	0.000	15.197	0.000	0.000	16.253	2.138	0.190	0.034	0.000	0.000	0.000
7.992	0.000	0.030	0.677	0.525	0.000	9.004	0.000	0.019	8.488	2.484	0.410	0.182	0.000	0.000	0.000
5.372	0.000	0.064	0.659	0.565	0.076	18.915	0.000	0.000	4.217	4.474	1.206	0.140	0.000	0.000	0.000
7.322	0.000	0.000	0.558	0.810	0.000	11.292	0.048	0.000	8.380	4.627	0.509	0.057	0.000	0.000	0.000
6.220	0.000	0.000	1.079	0.339	0.068	9.527	0.043	0.000	2.169	10.047	0.424	0.042	0.000	0.027	0.000
5.015	0.083	0.000	2.795	0.241	0.125	4.799	0.078	0.000	31.567	1.524	0.829	0.015	0.000	0.000	0.000
9.341	0.000	0.137	0.443	0.185	0.000	9.834	0.204	0.000	3.044	2.495	0.254	0.000	0.000	0.000	0.054
6.199	0.000	0.068	0.716	0.156	0.000	12.951	0.051	0.000	13.363	1.362	0.600	0.070	0.000	0.000	0.000
4.118	0.000	0.064	1.258	0.373	0.000	8.959	0.000	0.000	3.214	6.916	0.298	0.066	0.000	0.000	0.000
4.912	0.000	0.000	0.811	0.612	0.000	8.738	0.000	0.000	14.587	2.999	0.415	0.019	0.000	0.000	0.000
4.430	0.000	0.000	0.740	0.314	0.000	12.784	0.000	0.000	11.614	1.448	0.387	0.009	0.000	0.000	0.000
3.755	0.000	0.000	1.283	0.232	0.000	7.393	0.000	0.000	7.072	4.775	0.607	0.081	0.035	0.000	0.000
4.858	0.000	0.000	1.933	0.177	0.000	6.674	0.000	0.000	3.245	2.641	0.101	0.032	0.000	0.000	0.000
6.774	0.045	0.057	1.467	0.154	0.136	5.667	0.000	0.000	4.036	1.752	0.713	0.200	0.000	0.000	0.011
5.359	0.000	0.000	1.573	0.469	0.146	6.130	0.045	0.000	13.358	1.651	0.311	0.090	0.061	0.000	0.000
3.947	0.000	0.034	0.716	0.587	0.081	9.257	0.000	0.000	8.215	4.836	0.473	0.040	0.000	0.000	0.000
6.964	0.000	0.087	0.460	0.519	0.000	9.282	0.000	0.000	4.593	2.835	0.203	0.013	0.000	0.000	0.009
7.432	0.000	0.000	1.731	0.501	0.000	8.042	0.000	0.000	2.342	8.306	0.278	0.000	0.000	0.000	0.000
7.057	0.000	0.000	1.726	0.323	0.000	9.010	0.000	0.000	6.426	4.115	0.185	0.035	0.080	0.000	0.008
6.658	0.000	0.000	1.463	0.244	0.000	17.032	0.000	0.000	1.617	2.787	0.148	0.031	0.000	0.000	0.000
3.278	0.000	0.000	0.917	0.136	0.000	10.101	0.063	0.000	3.306	2.815	0.195	0.012	0.000	0.000	0.008
6.003	0.000	0.031	0.792	0.239	0.073	9.489	0.000	0.000	12.465	2.879	0.511	0.000	0.000	0.000	0.006
6.444	0.000	0.000	0.200	0.284	0.000	8.349	0.000	0.000	14.760	1.468	1.204	0.127	0.000	0.000	0.000
3.871	0.000	0.000	0.613	0.529	0.000	19.313	0.000	0.000	3.297	6.823	0.312	0.140	0.000	0.000	0.000
6.609	0.000	0.055	0.745	0.089	0.132	9.057	0.000	0.000	2.479	2.851	0.256	0.065	0.000	0.000	0.011
3.799	0.000	0.042	0.921	0.182	0.000	9.952	0.000	0.000	3.282	4.558	0.313	0.049	0.000	0.000	0.000
5.725	0.000	0.000	0.411	0.137	0.000	7.948	0.000	0.000	0.986	5.717	0.706	0.030	0.000	0.000	0.000
8.651	0.000	0.000	2.030	0.488	0.000	8.196	0.000	0.000	3.783	5.161	0.625	0.010	0.107	0.000	0.000
4.657	0.000	0.000	1.263	0.631	0.000	3.867	0.317	0.000	2.664	53.159	0.197	0.125	0.214	0.000	0.338
11.787	0.000	0.000	0.326	0.133	0.000	14.242	0.133	0.000	8.191	2.209	0.539	0.000	0.000	0.000	0.018
6.604	0.000	0.134	0.265	0.373	0.000	18.377	0.000	0.000	3.117	1.870	0.207	0.013	0.000	0.000	0.000
6.139	0.000	0.000	0.353	0.529	0.000	15.598	0.061	0.000	29.290	1.331	0.303	0.000	0.000	0.039	0.000
4.791	0.000	0.000	0.458	0.233	0.000	14.039	0.000	0.000	7.118	2.946	0.386	0.019	0.000	0.000	0.000
4.497	0.000	0.000	1.448	0.246	0.091	5.421	0.000	0.000	4.082	1.261	0.071	0.056	0.000	0.000	0.000
3.383	0.000	0.000	0.674	0.569	0.000	11.783	0.000	0.000	11.277	4.756	0.360	0.316	0.000	0.000	0.000
3.582	0.000	0.108	1.117	0.204	0.086	10.862	0.000	0.000	6.478	2.473	0.667	0.084	0.000	0.000	0.007
1.809	0.000	0.038	0.413	0.278	0.000	30.588	0.000	0.000	9.259	2.037	0.284	0.045	0.000	0.000	0.000
3.617	0.000	0.030	4.293	0.170	0.144	5.873	0.090	0.000	1.571	2.573	0.586	0.026	0.000	0.000	0.000
4.567	0.000	0.000	0.855	0.262	0.000	6.962	0.000	0.000	4.502	1.740	0.378	0.018	0.000	0.000	0.006
5.721	0.000	0.086	0.588	0.580	0.205	9.795	0.000	0.000	7.896	9.181	0.558	0.126	0.000	0.000	0.000
5.870	0.044	0.112	1.024	0.181	0.133	10.196	0.000	0.000	4.963	4.406	0.518	0.016	0.000	0.000	0.000
4.659	0.000	0.179	0.683	0.362	0.569	7.476	0.000	0.000	5.003	5.699	0.497	0.131	0.000	0.000	0.006
2.500	0.024	0.000	1.486	0.216	0.073	5.472	0.000	0.000	4.521	1.104	0.542	0.217	0.000	0.000	0.000
3.450	0.000	0.030	3.444	0.144	0.000	7.387	0.000	0.000	2.326	1.844	0.110	0.009	0.000	0.056	0.006
4.400	0.000	0.094	1.017	0.118	0.000	6.198	0.000	0.000	1.196	1.126	0.982	0.146	0.000	0.000	0.025
6.178	0.000	0.240	0.405	1.288	0.072	6.275	0.045	0.000	1.328	7.160	0.612	0.150	0.000	0.000	0.030
6.399	0.000	0.035	1.353	0.526	0.166	9.891	0.000	0.000	2.308	6.624	0.775	0.082	0.000	0.000	0.000
3.922	0.000	0.110	2.255	0.433	0.000	6.631	0.000	0.024	0.882	3.334	0.068	0.086	0.000	0.000	0.000
2.810	0.000	0.149	3.052	0.281	0.710	8.014	0.222	0.000	1.863	4.723	0.276	0.175	0.000	0.000	0.000
6.242	0.000	0.000	0.559	0.203	0.000	7.174	0.140	0.000	11.343	6.759	0.873	0.359	0.000	0.000	0.000
4.829	0.000	0.000	0.977	0.335	0.000	10.323	0.000	0.000	7.819	5.288	0.339	0.021	0.000	0.000	0.007
5.430	0.000	0.000	0.934	0.531	0.000	9.173	0.000	0.000	5.756	4.203	0.473	0.086	0.073	0.000	0.014
7.830	0.000	0.000	0.257	0.757	0.000	17.301	0.000	0.000	7.845	2.049	0.529	0.000	0.000	0.000	0.000
3.401	0.000	0.031	0.918	0.392	0.000	12.141	0.000	0.000	15.537	0.802	0.860	0.136	0.031	0.000	0.000
4.194	0.000	0.000	0.742	0.161	0.000	14.003	0.000	0.000	11.583	3.640	0.702	0.094	0.000	0.038	0.000
3.765	0.000	0.091	0.210	0.431	0.109	5.523	0.000	0.000	1.095	8.336	0.297	0.121	0.000	0.000	0.018
4.500	0.000	0.080	0.778	0.432	0.000	8.668	0.000	0.000	7.983	8.464	0.371	0.235	0.080	0.000	0.000
2.552	0.029	0.000	0.653	0.217	0.000	6.990	0.000	0.000	5.410	1.952	0.408	0.022	0.037	0.000	0.007
9.294	0.000	0.000	1.729	0.386	0.000	5.950	0.000	0.021	1.137	3.313	0.784	0.162	0.000	0.000	0.006
6.235	0.000	0.000	1.195	0.264	0.146	9.748	0.000	0.000	7.137	4.130	0.198	0.054	0.000	0.000	0.000
5.172	0.000	0.038	1.249	0.506	0.274	4.159	0.000	0.000	11.609	4.932	0.568	0.022	0.000	0.000	0.000
4.909	0.000	0.000	0.496	0.224	0.000	7.584	0.000	0.000	30.981	3.037	0.167	0.011	0.000	0.000	0.000
4.523	0.000	0.000	1.032	0.886	0.000	10.639	0.000	0.000	2.426	5.000	0.251	0.099	0.000	0.000	0.000
5.473	0.000	0.070	0.661	0.179	0.000	7.738	0.000	0.000	5.980	2.192	0.227	0.062	0.000	0.000	0.000
4.900	0.000	0.150	0.869	0.687	0.000	7.387	0.000	0.000	5.790	8.397	0.278	0.066	0.000	0.000	0.000
4.677	0.000	0.000	0.893	0.221	0.000	6.367	0.000	0.000	1.429	0.452	0.462	0.094	0.000	0.000	0.000
5.533	0.000	0.000	1.224	0.418	0.000	14.230	0.000	0.000	3.818	3.704	0.245	0.067	0.038	0.000	0.000
5.820	0.000	0.131	1.285	0.334	0.000	7.940	0.000	0.000	5.425	7.209	0.605	0.287	0.033	0.000	0.006
5.486	0.000	0.000	0.469	0.428	0.000	12.811	0.000	0.000	7.105	5.439	0.350	0.022	0.038	0.000	0.000
6.501	0.000	0.000	0.946	0.486	0.000	9.830	0.000	0.000	3.309	4.602	0.304	0.096	0.000	0.000	0.000
4.685	0.000	0.000	0.916	0.261	0.074	9.811	0.000	0.000	2.777	6.166	0.260	0.018	0.000	0.000	0.000
3.499	0.000	0.028	1.027	0.281	0.000	13.285	0.000	0.000	2.403	3.275	0.575	0.190	0.028	0.000	0.006
2.730	0.000	0.000	1.672	0.298	0.000	6.021	0.000	0.000	1.575	2.565	0.275	0.100	0.000	0.000	0.000
2.608	0.000	0.000	1.445	0.792	0.000	8.168	0.000	0.000	2.648	6.455	0.345	0.109	0.075	0.000	0.000
4.939	0.000	0.000	0.658	0.219	0.000	13.244	0.000	0.000	17.880	0.897	0.616	0.011	0.000	0.000	0.007
5.333	0.000	0.035	1.319	0.178	0.000	6.599	0.000	0.000	2.438	3.511	0.289	0.061	0.000	0.000	0.007
6.696	0.026	0.000	0.562	0.481	0.079	9.687	0.049	0.000	9.929	4.853	0.797	0.126	0.033	0.000	0.007
6.189	0.000	0.108	0.814	1.337	0.514	10.152	0.000	0.000	10.069	9.487	0.200	0.000	0.000	0.000	0.000
3.853	0.000	0.114	1.222	0.192	0.000	7.767	0.000	0.000	3.916	1.966	0.660	0.042	0.000	0.000	0.000
4.870	0.000	0.079	1.233	0.171	0.000	7.108	0.000	0.000	8.353	1.619	0.318	0.008	0.000	0.000	0.000
4.341	0.000	0.029	1.273	0.339	0.000	7.555	0.000	0.000	1.795	4.711	0.477	0.017	0.000	0.000	0.006
5.139	0.000	0.054	1.160	0.543	0.065	10.988	0.000	0.000	2.565	4.670	0.454	0.040	0.000	0.000	0.000
4.898	0.000	0.000	0.763	0.601	0.000	15.612	0.000	0.000	2.718	5.911	0.318	0.000	0.000	0.081	0.000
5.923	0.000	0.031	0.710	0.384	0.000	15.604	0.000	0.000	8.442	3.254	0.660	0.009	0.000	0.000	0.000
5.763	0.000	0.080	1.094	0.193	0.000	5.536	0.000	0.000	3.758	2.085	0.553	0.210	0.000	0.000	0.000
3.859	0.000	0.122	1.585	0.557	0.000	8.464	0.000	0.000	6.136	6.472	0.413	0.012	0.000	0.000	0.000
6.606	0.000	0.090	1.480	0.534	0.000	7.050	0.067	0.000	10.684	3.897	0.375	0.092	0.000	0.000	0.000
3.703	0.000	0.051	1.090	0.395	0.000	16.858	0.075	0.000	2.703	5.130	0.983	0.148	0.051	0.000	0.000
7.281	0.000	0.000	1.826	0.387	0.000	5.555	0.000	0.000	1.540	4.799	0.646	0.132	0.041	0.000	0.000
5.741	0.000	0.057	0.478	0.193	0.136	7.623	0.043	0.000	19.943	1.767	0.318	0.059	0.000	0.000	0.000
8.149	0.000	0.029	0.482	0.166	0.000	10.909	0.000	0.000	1.922	1.561	1.005	0.095	0.000	0.000	0.000
4.748	0.000	0.105	1.185	0.973	0.000	7.629	0.000	0.000	7.967	5.181	0.533	0.015	0.053	0.049	0.000
4.232	0.000	0.000	0.654	0.492	0.130	10.022	0.000	0.000	13.662	6.521	0.505	0.040	0.000	0.000	0.005
4.429	0.000	0.037	1.006	0.219	0.000	9.119	0.000	0.000	4.221	2.200	1.164	0.130	0.000	0.035	0.007
6.424	0.000	0.066	1.137	0.125	0.000	11.003	0.000	0.022	1.088	5.612	0.400	0.068	0.000	0.000	0.000
5.255	0.000	0.000	1.371	0.395	0.000	9.349	0.000	0.000	6.542	4.035	0.483	0.000	0.000	0.000	0.006

ENSG00000248692	ENSG00000213089	ENSG00000123636	ENSG00000238193	ENSG00000201815	ENSG00000272506	ENSG00000199709	ENSG00000248669	ENSG00000259421	ENSG00000260586	ENSG00000200999	ENSG00000249818	ENSG00000117906	ENSG00000213216	ENSG00000251656	ENSG00000122176
0.078	0.240	1.631	0.105	0.000	0.000	0.530	0.000	0.000	0.000	0.000	0.000	9.340	0.780	0.161	1.316
0.000	1.166	0.698	0.059	0.000	0.102	0.301	0.000	0.000	0.000	0.000	0.000	12.051	1.049	0.000	23.019
0.059	0.208	1.524	0.040	0.000	0.000	0.000	0.000	0.000	0.000	0.000	0.000	23.299	1.180	0.000	1.002
0.022	0.823	2.557	0.030	0.000	0.034	0.152	0.000	0.000	0.000	0.000	0.000	9.147	0.613	0.000	6.128
0.025	0.425	0.893	0.150	0.223	0.038	0.506	0.000	0.091	0.000	0.000	0.000	10.075	0.929	0.051	6.567
0.217	0.129	3.354	0.066	0.000	0.241	0.000	0.000	0.072	0.000	0.000	0.000	11.125	0.539	0.000	0.699
0.000	0.436	1.167	0.030	0.000	0.034	0.750	0.000	0.081	0.030	0.000	0.000	9.626	0.441	0.000	23.837
0.023	0.215	0.746	0.078	0.629	0.000	0.000	0.000	0.000	0.000	0.000	0.000	8.463	0.408	0.048	16.443
0.000	0.107	1.169	0.018	0.000	0.125	0.000	0.000	0.000	0.000	0.000	0.000	10.740	0.674	0.167	8.275
0.000	0.414	0.998	0.053	0.000	0.000	1.067	0.000	0.000	0.000	0.000	0.000	8.979	1.373	0.000	13.590
0.019	0.305	1.195	0.169	0.000	0.119	0.000	0.000	0.000	0.000	0.000	0.000	9.490	0.289	0.000	18.210
0.140	0.395	1.638	0.054	0.000	0.031	0.136	0.000	0.000	0.000	0.000	0.000	9.884	0.499	0.000	8.779
0.000	0.299	1.779	0.194	0.000	0.032	0.140	0.000	0.000	0.000	0.000	0.000	6.396	0.154	0.128	33.603
0.077	0.610	1.813	0.091	0.000	0.208	0.393	0.000	0.000	0.000	0.000	0.000	7.851	0.289	0.040	6.388
0.086	0.679	0.662	0.520	0.000	0.132	0.584	0.000	0.000	0.000	0.000	0.000	6.664	2.574	0.000	4.123
0.000	0.458	1.815	0.130	0.000	0.149	0.131	0.051	0.000	0.000	0.000	0.000	5.570	0.193	0.040	7.510
0.045	0.147	1.064	0.090	0.000	0.069	0.302	0.000	0.000	0.000	0.000	0.000	9.348	0.389	0.046	9.459
0.027	0.070	0.595	0.036	0.000	0.041	0.180	0.000	0.000	0.000	0.000	0.000	12.660	1.121	0.055	2.399
0.000	0.450	0.735	0.162	0.000	0.031	0.000	0.000	0.000	0.000	0.000	0.000	7.026	0.301	0.042	4.709
0.043	0.368	0.900	0.043	0.000	0.033	0.000	0.000	0.000	0.000	0.256	0.000	7.580	0.322	0.000	20.635
0.000	0.453	0.908	0.169	0.000	0.106	0.623	0.000	0.000	0.000	0.000	0.000	13.978	0.972	0.000	14.025
0.048	0.218	4.359	0.143	0.000	0.219	0.000	0.000	0.000	0.000	0.000	0.000	12.042	0.177	0.000	5.384
0.046	0.183	1.081	0.109	0.000	0.000	0.000	0.000	0.000	0.000	0.275	0.000	14.692	0.867	0.000	5.507
0.020	0.311	0.722	0.079	0.000	0.000	0.401	0.000	0.000	0.027	0.000	0.000	11.225	0.393	0.000	22.049
0.022	0.433	0.921	0.118	0.000	0.034	0.149	0.058	0.000	0.000	0.000	0.000	6.314	0.493	0.045	15.338
0.114	0.480	1.520	0.061	0.000	0.105	0.000	0.000	0.000	0.000	0.000	0.000	6.392	0.341	0.000	8.580
0.026	0.306	1.295	0.104	0.000	0.238	1.051	0.000	0.000	0.035	0.000	0.000	8.044	0.837	0.053	16.608
0.137	0.419	0.714	0.183	0.000	0.070	0.000	0.000	0.000	0.000	0.000	0.000	9.774	1.133	0.000	5.283
0.227	0.387	2.053	0.122	0.000	0.278	0.154	0.000	0.000	0.000	0.000	0.000	9.802	0.282	0.093	37.840
0.023	0.479	1.874	0.031	0.000	0.105	0.154	0.000	0.000	0.000	0.270	0.000	18.453	0.454	0.000	8.350
0.188	0.345	0.832	0.138	0.000	0.029	0.254	0.000	0.000	0.000	0.222	0.000	11.051	0.747	0.116	19.159
0.039	0.491	2.113	0.105	0.000	0.181	0.133	0.000	0.000	0.000	0.233	0.000	14.782	0.196	0.000	5.688
0.023	0.000	1.095	0.015	0.000	0.105	0.155	0.000	0.000	0.000	0.000	0.000	7.620	0.285	0.000	15.084
0.088	0.290	0.914	0.030	0.000	0.169	0.000	0.000	0.000	0.000	0.000	0.000	9.756	0.658	0.091	105.857
0.047	0.795	1.756	0.156	0.000	0.071	0.000	0.000	0.000	0.000	0.000	0.000	14.471	3.592	0.000	20.487
0.063	0.415	1.900	0.071	0.000	0.065	0.429	0.056	0.000	0.000	0.000	0.000	10.904	0.472	0.043	31.585
0.000	0.529	3.662	0.040	0.000	0.371	0.546	0.000	0.000	0.000	0.239	0.000	7.831	0.251	0.125	40.847
0.040	0.317	2.641	0.040	0.000	0.278	0.681	0.053	0.000	0.000	0.238	0.000	7.929	0.300	0.083	55.777
0.024	0.378	1.939	0.096	0.000	0.110	0.000	0.063	0.000	0.000	0.000	0.000	7.611	0.717	0.148	5.713
0.025	0.358	2.663	0.050	0.000	0.114	0.336	0.000	0.000	0.000	0.000	0.000	8.483	0.555	0.051	22.742
0.025	0.227	2.139	0.017	0.000	0.076	0.334	0.000	0.000	0.000	0.000	0.000	8.653	0.553	0.000	41.180
0.104	0.712	1.330	0.098	0.000	0.160	0.424	0.000	0.000	0.000	0.000	0.000	16.311	0.467	0.043	15.440
0.046	0.573	2.371	0.062	0.000	0.035	0.155	0.000	0.000	0.000	0.000	0.000	11.813	0.742	0.047	10.917
0.079	0.672	2.633	0.079	0.000	0.060	0.400	0.000	0.000	0.000	0.000	0.000	11.018	0.343	0.081	18.458
0.000	0.441	4.810	0.084	0.000	0.129	0.000	0.000	0.000	0.000	0.249	0.000	14.085	0.470	0.000	38.305
0.020	0.591	1.584	0.041	0.000	0.157	0.277	0.000	0.000	0.000	0.000	0.000	14.378	0.255	0.253	43.381
0.023	0.326	2.723	0.045	0.000	0.069	0.000	0.000	0.000	0.184	0.000	0.000	18.963	1.122	0.093	129.185
0.137	0.240	2.659	0.092	0.000	0.245	0.000	0.000	0.000	0.000	0.000	0.000	10.565	0.965	0.000	0.764
0.040	0.545	2.663	0.066	0.000	0.061	0.402	0.000	0.000	0.000	0.000	0.000	12.527	0.738	0.041	53.238
0.019	0.466	1.132	0.150	0.000	0.029	0.000	0.098	0.000	0.000	0.000	0.000	9.738	0.883	0.038	16.201
0.019	0.395	1.268	0.126	0.000	0.058	0.382	0.049	0.000	0.000	0.000	0.000	19.557	0.701	0.000	7.027
0.020	0.716	1.184	0.108	0.181	0.062	0.137	0.000	0.000	0.000	0.000	0.000	10.717	0.955	0.042	70.269
0.103	0.378	1.700	0.138	0.000	0.000	0.139	0.000	0.000	0.000	0.000	0.000	18.664	0.563	0.170	38.338
0.036	0.430	1.541	0.207	0.000	0.028	0.123	0.000	0.000	0.000	0.000	0.000	9.502	0.588	0.000	8.757
0.000	0.396	0.657	0.830	0.000	0.051	0.000	0.000	0.000	0.000	0.000	0.000	17.099	1.083	0.069	73.331
0.175	0.572	3.226	0.467	0.000	0.134	0.000	0.000	0.000	0.000	0.000	0.000	21.181	2.170	0.000	4.720
0.022	0.512	1.439	0.102	0.000	0.066	1.027	0.000	0.000	0.000	0.000	0.000	11.519	0.647	0.045	15.500
0.021	0.323	1.325	0.137	0.000	0.126	0.139	0.000	0.000	0.000	0.000	0.000	13.753	0.408	0.084	18.787
0.000	0.748	1.538	0.136	0.000	0.000	0.138	0.000	0.000	0.000	0.000	0.000	11.770	0.456	0.084	6.230
0.000	0.274	1.885	0.178	0.000	0.000	0.129	0.000	0.000	0.000	0.000	0.000	10.941	0.378	0.235	10.787
0.000	0.375	1.789	0.162	0.000	0.067	0.000	0.000	0.000	0.000	0.000	0.000	8.794	0.819	0.000	12.374
0.070	0.411	0.808	0.117	0.000	0.160	0.707	0.000	0.000	0.000	0.000	0.000	17.590	0.780	0.000	9.821
0.127	0.452	1.701	0.097	0.000	0.028	0.859	0.000	0.000	0.000	0.215	0.000	6.053	0.451	0.000	4.641
0.019	1.046	1.430	0.130	0.000	0.030	0.263	0.000	0.000	0.000	0.000	0.000	6.065	0.290	0.040	26.094
0.000	0.341	2.255	0.145	0.000	0.066	0.880	0.000	0.000	0.000	0.000	0.000	16.370	1.132	0.045	27.899
0.028	0.183	1.199	0.131	0.000	0.043	0.000	0.000	0.000	0.000	0.000	0.000	9.721	0.554	0.115	4.766
0.076	0.251	1.061	0.128	0.000	0.000	0.000	0.000	0.000	0.000	0.000	0.000	16.556	0.380	0.000	28.183
0.051	0.267	1.151	0.068	0.000	0.156	0.172	0.000	0.000	0.000	0.000	0.000	15.481	0.760	0.000	32.142
0.034	0.533	2.173	0.091	0.000	0.052	0.229	0.000	0.000	0.000	0.000	0.000	8.615	0.505	0.139	1.909
0.054	0.456	0.517	0.233	0.000	0.000	0.000	0.070	0.000	0.000	0.000	0.000	16.407	0.798	0.055	1.952
0.097	0.384	0.946	0.117	0.000	0.000	0.000	0.000	0.000	0.000	0.231	0.000	13.405	0.872	0.000	9.657
0.039	0.155	0.612	0.079	0.000	0.362	0.000	0.000	0.000	0.053	0.000	0.000	15.088	1.372	0.081	27.538
0.125	0.797	0.978	0.108	0.000	0.055	0.000	0.000	0.000	0.000	0.000	0.000	9.991	0.711	0.037	31.733
0.106	0.323	1.115	0.212	0.000	0.054	0.714	0.000	0.000	0.000	0.000	0.000	11.444	1.225	0.000	12.561
0.107	0.458	1.579	0.126	0.000	0.041	0.727	0.000	0.000	0.000	0.000	0.000	5.468	0.400	0.000	74.620
0.000	0.849	2.676	0.087	0.000	0.099	0.000	0.000	0.000	0.000	0.000	0.000	11.424	0.483	0.067	63.901
0.068	0.267	1.033	0.076	0.000	0.069	0.153	0.000	0.000	0.000	0.000	0.000	12.502	1.293	0.093	46.114
0.000	1.601	1.841	0.182	0.000	0.208	0.000	0.000	0.000	0.184	0.000	0.000	12.373	2.360	0.000	260.339
0.057	0.374	1.762	0.076	0.000	0.175	0.193	0.000	0.000	0.000	0.000	0.000	25.579	1.204	0.117	10.484
0.028	0.522	1.359	0.152	0.000	0.131	0.961	0.000	0.000	0.000	0.000	0.000	12.266	1.413	0.000	39.794
0.078	0.342	0.961	0.227	0.000	0.279	0.353	0.000	0.000	0.000	0.000	0.000	29.496	1.878	0.000	11.227
0.102	0.402	1.375	0.027	0.000	0.000	0.415	0.000	0.000	0.000	0.000	0.000	16.894	0.762	0.000	9.089
0.121	0.255	1.332	0.130	0.000	0.037	0.000	0.000	0.000	0.000	0.000	0.000	6.196	0.784	0.000	4.477
0.000	0.144	2.350	0.092	0.000	0.000	0.186	0.000	0.000	0.000	0.000	0.000	7.517	0.205	0.000	22.673
0.184	0.271	1.877	0.092	0.000	0.105	0.000	0.000	0.000	0.000	0.000	0.000	6.967	0.456	0.047	3.971
0.000	0.959	0.817	0.016	0.000	0.037	0.165	0.000	0.000	0.000	0.000	0.000	11.766	0.606	0.000	67.545
0.230	0.402	0.961	0.064	0.000	0.088	0.130	0.000	0.000	0.000	0.000	0.000	9.869	0.334	0.000	1.201
0.020	0.708	0.901	0.120	0.000	0.031	0.270	0.000	0.000	0.000	0.000	0.000	7.488	0.497	0.041	4.296
0.037	0.431	1.201	0.073	0.000	0.028	0.000	0.000	0.067	0.000	0.000	0.000	8.409	0.272	0.038	0.920
0.000	0.513	1.957	0.071	0.000	0.000	0.000	0.000	0.000	0.000	0.000	0.000	10.666	0.707	0.073	4.407
0.399	1.021	2.275	0.089	0.000	0.378	0.000	0.000	0.000	0.000	0.000	0.000	10.028	0.425	0.000	7.700
0.059	0.874	0.820	0.026	0.000	0.210	0.265	0.000	0.000	0.000	0.000	0.000	8.287	0.292	0.081	24.129
0.000	0.323	1.702	0.127	0.000	0.000	0.000	0.000	0.000	0.000	0.000	0.000	7.510	0.423	0.000	6.637
0.020	0.182	4.982	0.093	0.000	0.456	0.134	0.000	0.000	0.000	0.000	0.000	9.677	0.641	0.123	3.249
0.172	0.201	3.022	0.282	0.000	0.147	0.000	0.050	0.000	0.000	0.000	0.000	9.935	1.046	0.118	1.566
0.022	0.757	1.191	0.030	0.000	0.068	0.150	0.058	0.000	0.000	0.000	0.000	12.175	0.497	0.046	47.016
0.047	0.122	0.608	0.420	0.000	0.071	0.000	0.000	0.000	0.000	0.000	0.000	12.556	0.636	0.000	4.113
0.000	0.498	1.667	0.063	0.000	0.291	1.283	0.000	0.000	0.000	0.000	0.000	11.727	1.179	0.000	18.453
0.060	1.260	2.082	0.201	0.000	0.092	0.000	0.000	0.000	0.000	0.000	0.000	10.850	2.238	0.000	31.708
0.047	0.520	1.458	0.172	0.000	0.179	0.000	0.000	0.000	0.000	0.000	0.000	10.893	0.695	0.048	25.904
0.186	0.396	1.691	0.078	0.000	0.107	0.000	0.000	0.000	0.000	0.000	0.000	9.257	0.231	0.000	30.205
0.000	0.318	1.741	0.041	0.000	0.046	0.000	0.000	0.000	0.000	0.000	0.000	21.574	1.206	0.000	28.313
0.000	0.439	0.896	0.079	0.000	0.272	0.666	0.000	0.000	0.000	0.000	0.000	13.489	1.812	0.041	35.520
0.025	0.267	1.378	0.068	0.000	0.000	0.515	0.000	0.000	0.000	0.000	0.000	7.267	0.821	0.052	36.085
0.000	0.535	2.298	0.214	0.000	0.134	0.000	0.000	0.000	0.000	0.000	0.000	10.204	1.013	0.000	59.734
0.051	0.201	1.020	0.205	0.000	0.000	0.000	0.000	0.000	0.000	0.000	0.000	9.744	0.381	0.000	49.673
0.093	0.490	1.095	0.109	0.000	0.036	0.474	0.000	0.000	0.000	0.000	0.000	5.827	0.465	0.000	8.515
0.083	0.272	1.163	0.305	0.000	0.064	0.000	0.000	0.000	0.000	0.000	0.000	16.947	1.392	0.043	10.269
0.078	0.255	1.321	0.065	0.000	0.030	0.000	0.000	0.000	0.000	0.000	0.000	12.995	0.194	0.000	4.912
0.000	0.352	0.617	0.016	0.000	0.037	0.330	0.000	0.000	0.000	0.000	0.000	9.089	1.273	0.050	20.011
0.023	0.181	0.393	0.123	0.000	0.000	0.000	0.000	0.000	0.000	0.000	0.000	11.816	0.800	0.000	3.967
0.108	0.340	1.219	0.101	0.000	0.099	0.438	0.000	0.000	0.000	0.000	0.000	10.926	0.268	0.044	41.864
0.045	0.205	1.334	0.015	0.000	0.000	0.453	0.000	0.000	0.000	0.000	0.000	9.525	0.443	0.046	10.814
0.143	0.627	1.543	0.192	0.000	0.073	0.000	0.000	0.000	0.000	0.000	0.000	10.551	1.187	0.000	40.068
0.023	0.208	0.675	0.091	0.000	0.000	0.460	0.000	0.000	0.000	0.000	0.000	11.379	0.282	0.047	10.049
0.072	0.600	0.871	0.129	0.000	0.037	0.326	0.000	0.000	0.000	0.000	0.000	11.405	0.359	0.000	29.655
0.042	0.463	1.881	0.111	0.000	0.064	0.281	0.000	0.000	0.000	0.000	0.000	9.396	0.362	0.000	12.327
0.000	0.473	2.484	0.129	0.000	0.074	0.163	0.000	0.000	0.000	0.000	0.000	14.302	1.017	0.099	29.673
0.000	0.685	2.076	0.098	0.000	0.032	0.000	0.000	0.000	0.000	0.000	0.000	22.256	0.727	0.043	26.894
0.079	0.443	1.942	0.160	0.000	0.000	0.000	0.000	0.000	0.000	0.000	0.000	11.462	0.543	0.000	107.023
0.090	0.377	1.123	0.156	0.000	0.000	0.000	0.000	0.000	0.000	0.000	0.000	8.917	0.581	0.000	15.698
0.027	0.745	0.737	0.072	0.000	0.249	0.000	0.000	0.000	0.000	0.000	0.000	9.742	0.740	0.000	24.735
0.047	0.466	1.453	0.111	0.000	0.000	0.160	0.000	0.000	0.000	0.000	0.039	11.040	0.295	0.000	387.513
0.000	0.340	1.887	0.331	0.000	0.000	0.159	0.000	0.000	0.000	0.000	0.000	18.111	0.994	0.194	911.676
0.000	0.377	1.986	0.089	0.000	0.102	0.000	0.000	0.000	0.000	0.000	0.000	7.197	0.549	0.091	16.022
0.021	0.359	0.923	0.169	0.000	0.032	0.000	0.000	0.000	0.000	0.000	0.000	9.285	0.471	0.000	17.135
0.069	0.360	1.373	0.138	0.000	0.210	0.000	0.181	0.000	0.000	0.000	0.000	13.762	1.707	0.000	115.654
0.000	0.452	1.336	0.109	0.000	0.056	0.368	0.000	0.067	0.000	0.215	0.000	9.396	0.406	0.037	9.096
0.000	0.861	0.815	0.090	0.000	0.000	0.228	0.000	0.000	0.000	0.000	0.000	4.414	0.335	0.069	2.115
0.109	0.454	1.835	0.085	0.000	0.028	0.370	0.000	0.000	0.000	0.000	0.000	13.042	0.589	0.038	8.430
0.087	0.659	1.719	0.093	0.000	0.106	0.000	0.000	0.000	0.000	0.000	0.000	8.192	0.517	0.000	8.125
0.109	0.215	0.726	0.183	0.000	0.084	0.739	0.000	0.000	0.000	0.000	0.000	12.903	0.679	0.000	308.039
0.099	0.569	1.414	0.132	0.000	0.000	0.133	0.000	0.000	0.000	0.000	0.000	17.684	0.834	0.000	66.728
0.152	0.233	0.597	0.271	0.000	0.078	0.686	0.000	0.000	0.000	0.000	0.000	10.673	1.071	0.157	1.928
0.026	0.169	1.097	0.121	0.000	0.000	0.349	0.000	0.000	0.000	0.000	0.000	10.138	0.449	0.000	8.449
0.029	0.564	1.242	0.153	0.000	0.132	0.000	0.000	0.000	0.000	0.000	0.000	10.007	0.855	0.059	11.406
0.032	0.507	1.498	0.215	0.000	0.148	1.089	0.000	0.000	0.000	0.000	0.000	12.627	0.640	0.000	12.793
0.131	0.309	1.522	0.245	0.000	0.000	0.000	0.069	0.000	0.000	0.000	0.000	7.328	0.455	0.054	15.100
0.127	0.549	1.498	0.110	0.000	0.084	0.246	0.000	0.000	0.000	0.000	0.000	6.013	0.724	0.000	5.524
0.019	0.367	1.619	0.062	0.000	0.000	0.379	0.000	0.000	0.000	0.000	0.000	11.944	0.696	0.077	9.527
0.333	0.568	1.151	0.178	0.000	0.000	0.902	0.000	0.000	0.000	0.000	0.000	10.942	1.243	0.000	13.427
0.122	0.706	1.050	0.128	0.155	0.080	0.117	0.000	0.000	0.000	0.000	0.000	17.497	0.820	0.036	20.629
0.000	0.432	1.681	0.205	0.000	0.036	0.159	0.000	0.000	0.000	0.278	0.000	13.115	0.292	0.097	26.539
0.042	0.333	1.964	0.113	0.000	0.130	0.143	0.000	0.000	0.000	0.000	0.000	11.970	0.473	0.000	26.948
0.104	0.653	1.078	0.181	0.000	0.064	0.000	0.000	0.000	0.000	0.000	0.000	8.930	0.929	0.000	56.701

ENSG00000232353	ENSG00000180770	ENSG00000138134	ENSG00000021574	ENSG00000254824	ENSG00000129450	ENSG00000064995	ENSG00000224028	ENSG00000188690	ENSG00000261617	ENSG00000234862	ENSG00000248984	ENSG00000172974	ENSG00000253818	ENSG00000051620	ENSG00000168496
0.000	0.000	1.867	8.128	0.000	4.659	18.907	0.000	5.823	0.145	0.000	0.000	4.158	0.000	2.371	11.536
0.000	0.000	1.138	4.585	0.000	8.362	23.080	0.000	4.184	0.020	0.000	0.000	4.657	0.052	6.212	9.859
0.000	0.000	1.137	10.509	0.000	3.688	34.295	0.000	5.777	0.021	0.000	0.000	3.742	0.000	2.914	33.001
0.000	0.000	0.544	8.975	0.000	1.429	20.815	0.000	3.421	0.160	0.000	0.000	3.227	0.000	3.477	15.901
0.000	0.024	1.713	4.776	0.000	8.570	19.120	0.000	5.581	0.080	0.000	0.000	2.947	0.000	7.671	6.819
0.000	0.000	1.676	11.442	0.000	5.194	26.939	0.000	4.787	0.056	0.000	0.000	3.303	0.000	1.988	24.275
0.000	0.000	2.544	4.444	0.269	8.081	16.057	0.000	5.134	0.024	0.000	0.000	3.099	0.000	2.989	20.798
0.000	0.000	1.328	5.804	0.000	10.429	14.018	0.000	9.481	0.159	0.000	0.000	3.833	0.055	3.482	5.585
0.000	0.000	1.502	5.825	0.000	5.449	22.794	0.000	7.929	0.325	0.058	0.000	2.972	0.063	4.358	7.024
0.000	0.000	1.335	5.862	0.000	17.981	24.403	0.000	9.640	0.028	0.000	0.000	4.834	0.368	4.524	4.975
0.000	0.000	1.045	5.413	0.235	6.219	18.743	0.000	5.782	0.132	0.000	0.000	3.105	0.136	3.035	7.113
0.000	0.000	1.229	6.628	0.000	4.730	17.224	0.000	3.859	0.039	0.000	0.000	2.696	0.000	2.681	18.797
0.000	0.000	0.530	7.049	0.000	2.858	18.232	0.000	4.360	0.122	0.000	0.000	2.404	0.000	2.977	15.225
0.000	0.000	0.624	6.565	0.000	4.103	17.039	0.000	3.652	0.256	0.000	0.000	2.789	0.000	4.050	10.679
0.000	0.000	0.918	4.781	0.000	7.049	13.914	0.000	7.443	0.000	0.000	0.000	4.638	0.000	3.701	27.208
0.000	0.000	1.492	5.546	0.235	17.656	11.073	0.000	3.488	0.256	0.000	0.000	2.334	0.045	2.586	4.889
0.000	0.000	0.951	9.937	0.000	2.243	18.839	0.000	3.827	0.020	0.000	0.000	3.340	0.000	2.846	20.307
0.000	0.000	1.554	11.396	0.000	2.989	38.460	0.000	16.214	0.005	0.000	0.000	6.247	0.186	2.463	9.097
0.000	0.000	0.965	4.457	0.000	8.730	12.382	0.000	6.345	0.040	0.000	0.000	3.538	0.000	2.197	8.566
0.000	0.000	0.627	4.616	0.000	5.258	12.357	0.000	3.034	0.066	0.000	0.000	2.924	0.000	1.860	12.763
0.000	0.000	1.124	6.171	0.000	3.657	37.961	0.000	14.088	0.049	0.000	0.000	4.502	0.000	4.874	8.320
0.000	0.000	1.083	12.676	0.000	1.038	16.427	0.000	4.531	0.055	0.000	0.000	3.989	0.000	1.327	19.920
0.000	0.000	1.145	7.778	0.000	4.144	29.565	0.000	7.009	0.021	0.000	0.000	2.499	0.000	3.197	11.469
0.000	0.000	1.576	4.767	0.000	5.141	14.793	0.000	4.347	0.007	0.000	0.000	4.294	1.015	2.365	20.927
0.000	0.000	0.966	5.552	0.000	14.987	17.327	0.000	3.989	0.047	0.000	0.000	3.100	0.000	3.493	9.752
0.000	0.000	1.807	6.883	0.000	8.991	18.648	0.000	4.507	0.176	0.000	0.000	3.002	0.053	2.510	16.037
0.000	0.000	1.194	8.156	0.000	4.771	18.142	0.000	4.547	0.403	0.000	0.000	2.282	0.906	2.777	6.917
0.000	0.000	0.929	6.112	0.000	3.503	25.222	0.000	6.701	0.065	0.000	0.000	6.910	0.000	2.481	17.232
0.000	0.000	0.945	4.636	0.000	2.524	23.163	0.000	4.567	0.317	0.000	0.000	2.001	0.000	3.881	10.881
0.000	0.000	0.544	5.389	0.000	2.146	27.360	0.000	5.189	0.061	0.000	0.000	2.455	0.107	5.024	11.773
0.000	0.000	1.052	5.552	0.000	5.150	24.048	0.000	6.553	0.037	0.000	0.000	4.018	0.000	4.014	14.558
0.000	0.000	0.398	4.613	0.000	1.645	18.629	0.000	2.966	0.070	0.000	0.000	2.433	0.000	3.047	13.225
0.000	0.000	1.595	5.126	0.000	4.792	16.446	0.000	9.628	0.250	0.000	0.000	2.683	0.000	4.367	6.398
0.000	0.000	1.465	4.198	0.000	4.067	20.322	0.000	5.661	0.107	0.000	0.000	2.041	0.000	3.064	9.769
0.000	0.000	1.012	6.326	0.000	6.364	19.783	0.000	4.606	0.158	0.000	0.000	4.259	0.000	3.977	13.255
0.000	0.000	1.053	6.387	0.000	6.545	15.745	0.000	4.455	0.060	0.000	0.000	2.497	0.000	4.480	6.956
0.000	0.000	1.616	4.990	0.000	2.054	16.222	0.000	2.852	0.108	0.000	0.000	2.536	0.047	2.055	22.922
0.000	0.000	1.922	7.366	0.000	6.423	18.072	0.000	4.815	0.155	0.000	0.000	2.078	0.094	1.717	14.148
0.000	0.000	0.946	11.310	0.292	3.325	30.417	0.051	5.933	0.107	0.000	0.000	2.738	0.000	2.988	14.463
0.000	0.000	0.476	9.791	0.301	3.111	21.903	0.000	3.086	0.080	0.000	0.000	2.615	0.000	3.237	16.280
0.000	0.000	1.143	6.982	0.000	6.007	10.971	0.000	5.176	0.057	0.000	0.000	2.177	0.000	2.010	10.063
0.000	0.000	0.922	5.958	0.000	7.172	22.461	0.000	4.512	0.052	0.000	0.000	2.626	0.049	2.684	12.414
0.000	0.000	0.595	7.429	0.000	1.988	21.632	0.000	4.362	0.021	0.000	0.000	2.198	0.000	3.456	10.860
0.000	0.000	2.838	8.776	0.000	5.501	20.162	0.000	3.615	0.070	0.000	0.000	2.119	0.000	3.087	11.273
0.000	0.000	0.469	8.844	0.000	1.489	28.474	0.000	3.672	0.083	0.000	0.000	2.033	0.049	4.650	10.069
0.000	0.000	0.878	5.822	0.000	5.221	15.710	0.000	4.338	0.037	0.000	0.000	2.071	0.048	2.586	31.554
0.000	0.000	2.379	15.333	0.000	0.286	40.782	0.000	6.801	0.004	0.000	0.000	3.881	0.000	5.535	27.650
0.000	0.000	1.591	7.758	0.000	5.562	20.560	0.000	7.449	0.057	0.000	0.000	3.952	0.000	2.065	14.008
0.000	0.000	1.056	6.422	0.000	3.065	18.921	0.000	3.252	0.060	0.000	0.000	4.705	0.046	2.586	21.237
0.000	0.000	1.061	4.916	0.000	20.974	16.118	0.000	5.217	0.020	0.000	0.000	4.985	0.044	4.099	9.269
0.000	0.000	1.746	9.754	0.000	4.931	33.365	0.000	11.290	0.074	0.000	0.000	6.610	0.088	4.193	13.922
0.000	0.000	1.402	4.199	0.000	10.740	20.228	0.000	4.889	0.011	0.000	0.000	5.412	0.000	4.292	12.527
0.000	0.000	1.101	11.053	0.000	3.809	27.866	0.000	6.945	0.052	0.000	0.000	4.360	0.048	2.143	22.843
0.000	0.000	0.264	7.501	0.000	7.045	14.725	0.000	3.201	0.052	0.000	0.000	3.247	0.000	3.964	8.791
0.000	0.000	2.771	8.749	0.000	0.812	28.945	0.000	11.762	0.060	0.000	0.000	7.565	0.000	6.442	23.463
0.000	0.085	0.144	14.098	0.000	1.693	25.899	0.000	8.114	0.016	0.000	0.000	11.823	0.000	2.251	15.646
0.000	0.000	0.920	6.831	0.000	5.616	21.874	0.000	3.480	0.019	0.000	0.000	3.522	0.000	4.577	9.967
0.000	0.000	0.930	6.745	0.000	4.588	15.876	0.087	5.164	0.059	0.000	0.000	5.642	0.000	2.846	17.064
0.000	0.000	0.613	8.204	0.000	5.060	18.912	0.000	7.367	0.080	0.000	0.000	5.080	0.000	3.265	29.053
0.000	0.000	1.304	7.750	0.000	4.381	20.116	0.000	3.484	0.034	0.000	0.000	4.474	0.000	2.459	21.527
0.000	0.000	0.237	7.059	0.000	1.761	19.418	0.000	4.835	0.059	0.000	0.000	2.975	0.205	4.240	24.863
0.000	0.034	0.741	7.230	0.000	2.873	31.039	0.000	4.571	0.037	0.000	0.000	3.933	0.081	3.553	13.531
0.000	0.035	0.913	10.553	0.441	1.272	22.086	0.000	3.215	0.019	0.000	0.000	3.744	0.042	2.200	13.333
0.000	0.000	0.367	4.976	0.236	8.308	24.056	0.000	3.519	0.059	0.000	0.000	2.990	0.000	5.092	20.654
0.000	0.000	0.815	10.927	0.263	1.876	28.905	0.000	5.162	0.326	0.000	0.000	2.494	0.000	5.417	16.201
0.000	0.000	0.688	9.620	0.000	2.494	21.155	0.000	5.306	0.100	0.000	0.000	6.019	0.000	1.669	12.669
0.000	0.000	0.464	8.302	0.000	2.508	18.711	0.000	6.613	0.041	0.000	0.000	4.929	0.000	4.022	22.766
0.000	0.000	0.378	6.002	0.000	1.112	21.865	0.000	3.730	0.000	0.000	0.000	5.696	0.000	6.765	12.608
0.000	0.000	1.385	6.233	0.000	2.653	19.204	0.000	4.394	0.012	0.000	0.000	4.915	0.000	2.936	22.444
0.000	0.000	0.709	5.463	0.000	4.401	25.673	0.000	5.690	0.033	0.000	0.000	5.694	0.000	9.223	15.449
0.000	0.038	0.345	7.811	0.237	5.462	17.250	0.000	3.786	0.021	0.000	0.000	3.982	0.000	1.935	14.837
0.000	0.000	1.299	4.583	0.000	4.925	22.915	0.000	10.519	0.225	0.085	0.000	3.220	0.368	3.997	5.378
0.000	0.000	0.594	4.466	0.000	4.658	16.009	0.000	3.752	0.048	0.000	0.000	3.764	0.000	3.890	7.210
0.000	0.000	0.860	6.159	0.000	4.977	22.221	0.000	4.999	0.044	0.000	0.000	2.157	0.000	2.659	22.228
0.000	0.026	0.563	5.522	0.000	10.117	12.008	0.000	2.393	0.024	0.000	0.000	2.136	0.000	4.440	6.158
0.000	0.000	0.339	7.554	0.393	0.664	21.316	0.000	4.512	0.046	0.000	0.000	2.331	0.000	5.557	12.812
0.000	0.000	1.450	6.784	0.000	7.129	23.480	0.000	6.762	0.028	0.000	0.000	3.598	0.053	5.661	11.056
0.000	0.000	2.093	7.960	1.647	0.810	32.603	0.000	2.514	0.024	0.293	0.000	4.520	0.000	2.357	26.335
0.000	0.000	0.593	7.428	0.346	2.083	37.950	0.000	7.462	0.112	0.000	0.000	3.002	0.266	6.371	7.176
0.000	0.000	1.851	8.899	0.000	5.098	17.746	0.000	5.878	0.173	0.000	0.000	2.505	0.000	6.091	5.408
0.000	0.025	1.545	7.432	0.000	2.624	41.058	0.000	7.908	0.196	0.000	0.000	3.725	0.729	5.993	4.013
0.000	0.000	0.933	8.803	0.000	4.153	15.355	0.000	4.698	0.263	0.000	0.000	2.438	0.000	6.710	7.534
0.000	0.000	1.544	7.236	0.000	3.755	13.915	0.000	5.741	0.078	0.000	0.000	2.608	0.000	1.999	10.820
0.000	0.027	1.180	5.173	0.000	9.750	15.191	0.000	3.993	0.137	0.000	0.000	2.420	0.000	2.212	5.992
0.000	0.000	0.828	8.585	0.279	3.739	14.313	0.000	4.699	0.972	0.000	0.000	2.417	0.054	2.664	13.220
0.000	0.000	0.927	4.295	0.000	7.509	14.098	0.000	3.332	0.100	0.000	0.000	1.755	0.000	6.163	18.965
0.000	0.000	1.134	8.333	0.000	2.095	22.370	0.000	8.171	0.017	0.000	0.000	5.522	0.000	1.269	27.288
0.000	0.000	1.096	4.391	0.000	3.430	13.290	0.000	7.382	0.011	0.000	0.000	5.266	0.000	2.150	45.633
0.000	0.000	0.208	5.643	0.000	1.328	12.785	0.000	3.645	0.003	0.000	0.000	3.337	0.000	1.977	21.846
0.224	0.000	0.362	5.532	0.000	2.415	11.877	0.000	3.261	0.191	0.000	0.000	4.398	0.000	3.422	7.586
0.000	0.000	0.697	7.498	0.000	0.956	26.866	0.000	4.798	0.129	0.000	0.000	2.940	0.089	3.105	19.155
0.000	0.000	0.720	3.188	0.238	2.282	16.983	0.000	4.235	0.175	0.000	0.000	3.857	0.000	4.614	21.147
0.000	0.000	0.941	9.168	0.000	3.493	24.973	0.000	4.909	0.152	0.000	0.000	3.498	0.044	1.000	24.044
0.000	0.000	0.963	13.109	0.000	0.689	12.113	0.000	3.555	0.149	0.000	0.070	2.966	0.000	1.100	13.406
0.000	0.037	1.757	6.779	0.000	0.878	18.020	0.000	8.002	0.274	0.000	0.000	7.735	0.045	2.693	5.529
0.000	0.000	0.605	2.678	0.000	2.724	17.511	0.000	3.523	0.091	0.000	0.000	2.649	0.052	3.476	29.899
0.000	0.023	1.613	7.145	0.000	1.067	31.341	0.000	4.656	0.021	0.000	0.000	4.175	0.000	3.074	10.640
0.000	0.000	1.506	8.788	0.000	1.592	23.365	0.000	5.300	0.186	0.000	0.000	3.466	0.000	3.383	28.382
0.000	0.000	0.953	7.122	0.000	4.501	22.202	0.000	3.980	0.064	0.000	0.000	5.098	0.000	2.800	23.976
0.000	0.000	0.579	7.688	0.000	4.337	18.592	0.000	3.179	0.229	0.000	0.000	4.259	0.000	4.041	13.057
0.000	0.000	1.405	6.489	0.282	4.755	18.875	0.000	3.400	0.191	0.000	0.000	1.822	0.000	3.141	9.275
0.000	0.000	0.454	7.377	0.000	2.896	32.592	0.000	8.305	0.027	0.000	0.000	3.064	0.000	8.009	5.250
0.000	0.000	1.169	4.460	0.000	4.843	10.758	0.000	4.157	0.243	0.000	0.000	1.758	0.230	3.068	28.380
0.000	0.000	1.761	6.782	0.000	2.292	14.081	0.000	4.533	0.359	0.000	0.000	2.266	0.059	3.560	8.716
0.000	0.000	0.798	5.090	0.000	0.532	22.657	0.000	5.335	0.000	0.000	0.000	2.598	0.000	5.084	14.180
0.000	0.000	0.780	5.813	0.000	2.950	18.842	0.000	4.358	0.055	0.000	0.000	3.401	0.000	3.001	17.027
0.000	0.000	0.969	6.816	0.000	6.152	19.799	0.000	4.250	0.058	0.000	0.000	2.084	0.109	2.371	17.392
0.000	0.000	0.453	8.004	0.000	1.609	26.111	0.000	9.456	0.044	0.000	0.000	3.811	0.000	2.332	20.183
0.000	0.000	0.429	8.720	0.000	2.214	21.945	0.000	10.120	0.010	0.000	0.000	2.887	0.000	3.204	21.163
0.000	0.000	0.325	4.399	0.000	6.495	22.569	0.000	7.798	0.022	0.000	0.000	3.224	0.000	4.690	22.638
0.000	0.000	0.307	5.138	0.000	1.535	13.328	0.000	4.081	0.004	0.000	0.000	2.422	0.054	3.138	8.850
0.000	0.000	0.336	7.434	0.000	0.966	24.422	0.000	4.621	0.035	0.000	0.000	2.459	0.050	3.771	14.714
0.000	0.000	0.771	7.231	0.000	4.707	18.511	0.000	3.869	0.052	0.000	0.000	2.013	0.000	1.752	17.189
0.000	0.000	0.637	6.517	0.000	6.734	22.159	0.000	5.141	0.026	0.000	0.000	4.364	0.111	2.332	20.828
0.000	0.000	0.673	5.400	0.000	5.230	12.008	0.000	6.262	0.069	0.000	0.000	5.805	0.000	2.625	20.377
0.000	0.000	0.458	4.653	0.000	6.767	15.077	0.000	4.726	0.034	0.000	0.000	3.315	0.056	3.814	7.514
0.000	0.000	1.239	8.295	0.000	2.109	15.048	0.000	5.001	0.019	0.000	0.000	3.843	0.048	3.503	12.486
0.000	0.024	1.413	8.778	0.000	1.687	15.375	0.000	4.947	0.159	0.000	0.000	4.527	0.000	4.579	10.282
0.000	0.000	0.679	8.929	0.000	3.684	26.594	0.000	5.801	0.019	0.000	0.000	5.028	0.438	4.470	23.476
0.000	0.000	0.580	8.107	0.000	2.022	17.856	0.000	2.630	0.004	0.000	0.000	3.757	0.000	1.912	26.899
0.000	0.000	0.431	6.811	0.000	4.275	17.197	0.000	4.818	0.109	0.000	0.000	4.847	0.210	2.297	27.326
0.000	0.000	1.548	6.320	0.000	3.249	25.082	0.000	5.009	0.039	0.000	0.000	5.147	0.063	3.985	15.383
0.000	0.000	0.816	5.433	0.000	4.066	14.120	0.000	6.254	0.013	0.000	0.000	3.159	2.045	4.161	12.988
0.000	0.000	1.671	9.080	0.286	1.896	17.081	0.000	5.861	0.097	0.000	0.000	4.351	0.165	4.620	14.246
0.000	0.022	0.698	5.975	0.000	1.714	19.066	0.000	3.911	0.126	0.000	0.000	2.826	0.000	2.842	21.114
0.000	0.000	1.997	5.878	0.000	6.836	17.046	0.000	8.601	0.143	0.091	0.000	6.182	0.049	3.109	10.522
0.000	0.000	1.039	5.452	0.834	5.559	24.369	0.000	4.386	0.037	0.000	0.000	5.389	0.000	4.629	9.659
0.000	0.018	0.712	4.783	0.000	4.348	17.837	0.000	4.087	0.276	0.000	0.000	3.630	0.000	2.489	11.942
0.000	0.000	0.227	5.268	0.000	2.820	15.049	0.000	9.300	0.084	0.000	0.000	5.195	0.000	2.521	29.609
0.000	0.000	1.228	9.401	0.000	1.862	27.032	0.000	5.593	0.101	0.000	0.000	5.034	0.000	3.291	32.932
0.000	0.000	1.296	6.542	0.000	4.876	16.907	0.000	3.525	0.071	0.000	0.000	3.224	0.000	3.127	9.926
0.000	0.000	0.548	5.981	0.000	5.804	17.771	0.000	4.336	0.029	0.000	0.000	3.756	0.000	4.564	15.671
0.000	0.000	1.418	8.855	0.000	4.856	30.741	0.000	4.511	0.035	0.000	0.000	4.729	0.000	5.962	10.848
0.000	0.000	0.748	7.835	0.000	3.015	24.452	0.000	6.983	0.222	0.000	0.000	5.284	0.059	1.578	22.031
0.000	0.000	0.441	4.750	0.313	3.225	19.495	0.000	3.882	0.069	0.000	0.000	4.384	0.000	2.726	10.881
0.000	0.000	0.910	6.467	0.000	6.371	19.343	0.000	6.496	0.067	0.000	0.000	4.251	0.000	3.300	13.012
0.000	0.000	1.138	5.707	0.391	4.022	10.515	0.000	5.337	0.058	0.000	0.000	4.360	0.000	2.284	9.202
0.000	0.000	0.983	11.250	0.000	1.267	22.013	0.000	3.786	0.014	0.000	0.000	4.917	0.000	2.061	31.668
0.000	0.000	0.499	6.104	0.000	2.289	14.530	0.000	6.533	0.026	0.000	0.000	5.051	0.000	2.407	21.974
0.000	0.000	0.445	7.851	0.000	2.020	17.250	0.000	5.067	0.297	0.000	0.000	3.473	0.000	4.543	12.105
0.000	0.000	0.514	6.358	0.000	5.880	18.140	0.000	5.502	0.077	0.000	0.000	5.589	0.078	3.856	18.394
0.000	0.000	0.650	5.039	0.000	5.092	18.792	0.000	3.682	0.003	0.000	0.000	4.201	0.000	3.682	14.830
0.000	0.000	1.772	10.658	0.000	2.931	17.357	0.000	4.034	0.059	0.000	0.083	3.743	0.000	1.844	13.845
0.000	0.000	0.339	7.535	0.000	1.964	19.629	0.000	4.985	0.008	0.000	0.000	4.204	0.000	1.767	25.071
0.000	0.000	0.270	6.228	0.000	2.771	17.428	0.000	3.891	0.000	0.000	0.000	5.421	0.000	3.900	20.582

ENSG00000180155	ENSG00000196242	ENSG00000260433	ENSG00000265123	ENSG00000213924	ENSG00000207370	ENSG00000226797	ENSG00000196119	ENSG00000207391	ENSG00000218109	ENSG00000238223	ENSG00000274833	ENSG00000244568	ENSG00000167978	ENSG00000116132	ENSG00000169019
10.848	0.000	0.000	0.000	0.041	0.438	0.142	0.000	0.000	0.014	0.000	0.352	0.000	31.775	12.645	8.170
7.202	0.000	0.000	0.000	0.000	0.186	0.000	0.000	0.000	0.036	0.000	0.179	0.000	20.666	28.971	13.202
2.444	0.000	0.000	0.000	0.015	0.166	0.071	0.000	0.000	0.000	0.000	1.969	0.000	25.182	11.732	8.357
3.956	0.000	0.000	0.074	0.000	0.000	0.000	0.000	0.000	0.072	0.000	0.000	0.000	33.057	14.729	4.926
6.777	0.000	0.000	0.000	0.000	0.627	0.000	0.000	0.000	0.000	0.000	0.067	0.000	14.380	16.752	11.119
5.751	0.000	0.018	0.130	0.000	0.990	0.071	0.000	0.000	0.021	0.000	1.378	0.000	87.031	4.938	7.900
3.443	0.007	0.000	0.073	0.000	0.186	0.000	0.000	0.000	0.000	0.000	0.179	0.000	32.400	41.689	16.696
12.360	0.000	0.000	0.077	0.000	0.197	0.000	0.000	0.000	0.063	0.026	0.252	0.000	15.409	6.872	12.453
3.877	0.000	0.025	0.000	0.000	0.227	0.000	0.000	0.000	0.015	0.000	0.146	0.000	16.846	10.572	13.591
3.279	0.000	0.000	0.000	0.124	0.000	0.000	0.000	0.000	0.000	0.000	0.212	0.000	21.207	14.779	21.790
9.993	0.007	0.018	0.000	0.000	0.000	0.000	0.000	0.000	0.000	0.000	0.104	0.063	22.063	14.873	16.873
8.770	0.007	0.000	0.000	0.016	0.168	0.000	0.000	0.000	0.021	0.000	0.108	0.000	43.158	10.281	7.511
7.368	0.000	0.000	0.204	0.032	0.174	0.000	0.000	0.192	0.011	0.000	0.111	0.000	27.461	9.869	4.841
8.318	0.000	0.000	0.000	0.000	0.000	0.000	0.000	0.000	0.000	0.000	0.208	0.000	44.459	11.238	5.597
14.196	0.029	0.000	0.000	0.068	0.723	0.156	0.000	0.000	0.000	0.000	0.000	0.000	18.303	18.930	7.728
16.385	0.007	0.000	0.064	0.015	0.162	0.035	0.000	0.000	0.000	0.021	0.000	0.000	41.764	18.362	9.262
3.155	0.000	0.000	0.147	0.017	0.000	0.000	0.000	0.000	0.000	0.000	0.180	0.000	31.200	8.208	5.670
1.136	0.000	0.000	0.087	0.000	0.000	0.000	0.000	0.000	0.028	0.000	1.142	0.000	9.997	11.877	11.925
7.372	0.014	0.000	0.000	0.000	0.000	0.000	0.000	0.000	0.011	0.000	0.217	0.000	27.590	19.518	10.959
10.146	0.000	0.000	0.000	0.017	0.181	0.000	0.000	0.000	0.000	0.000	0.232	0.000	34.278	7.743	5.684
3.573	0.008	0.000	0.076	0.000	0.000	0.000	0.000	0.000	0.012	0.000	0.557	0.000	7.273	8.796	31.297
23.593	0.008	0.000	0.000	0.000	0.399	0.301	0.020	0.000	0.076	0.026	1.024	0.000	83.193	6.626	4.733
4.834	0.000	0.000	0.153	0.000	0.390	0.000	0.000	0.000	0.012	0.000	0.688	0.000	24.940	4.803	13.736
14.101	0.000	0.000	0.000	0.000	0.166	0.036	0.000	0.000	0.000	0.000	0.106	0.000	19.288	29.711	9.215
3.895	0.007	0.020	0.000	0.000	0.000	0.000	0.000	0.000	0.000	0.000	0.059	0.000	24.435	12.751	8.737
11.032	0.031	0.000	0.000	0.000	0.000	0.000	0.000	0.000	0.025	0.000	0.678	0.000	47.744	11.100	8.446
13.476	0.000	0.000	0.085	0.000	0.000	0.000	0.000	0.000	0.000	0.000	0.209	0.000	30.349	13.708	12.218
5.970	0.000	0.000	0.000	0.036	0.000	0.000	0.000	0.000	0.000	0.000	0.981	0.000	26.157	4.927	6.723
6.238	0.015	0.000	0.075	0.000	0.000	0.041	0.019	0.000	0.012	0.000	0.244	0.000	34.836	10.058	7.939
4.018	0.008	0.021	0.075	0.000	0.383	0.000	0.000	0.000	0.012	0.000	1.352	0.000	38.810	8.612	9.943
5.529	0.006	0.000	0.062	0.015	0.000	0.000	0.000	0.000	0.000	0.000	0.657	0.000	24.879	4.686	11.997
9.406	0.000	0.018	0.194	0.000	0.000	0.000	0.000	0.000	0.000	0.000	0.265	0.000	52.855	11.102	4.750
15.595	0.000	0.000	0.075	0.000	0.000	0.000	0.000	0.000	0.012	0.000	1.171	0.074	23.898	6.110	11.139
11.160	0.007	0.000	0.145	0.000	0.185	0.000	0.000	0.000	0.000	0.000	0.178	0.000	22.224	8.411	8.345
8.312	0.000	0.000	0.000	0.000	0.000	0.000	0.039	0.000	0.000	0.000	0.000	0.000	36.454	16.136	11.324
13.590	0.014	0.000	0.000	0.000	0.000	0.000	0.000	0.000	0.000	0.000	0.398	0.000	27.412	12.446	12.184
33.097	0.014	0.000	0.000	0.016	0.169	0.000	0.000	0.000	0.032	0.022	0.109	0.000	87.966	34.972	5.689
6.263	0.000	0.000	0.000	0.016	0.000	0.036	0.000	0.000	0.000	0.000	0.217	0.000	49.562	8.068	9.948
6.899	0.000	0.000	0.000	0.019	0.000	0.000	0.000	0.000	0.000	0.000	0.517	0.000	33.973	9.841	13.748
2.803	0.000	0.000	0.082	0.000	0.416	0.045	0.000	0.000	0.027	0.000	0.735	0.000	51.136	8.619	7.974
14.398	0.000	0.000	0.081	0.000	0.207	0.000	0.000	0.000	0.026	0.000	0.066	0.000	40.362	11.004	9.007
12.061	0.000	0.019	0.000	0.000	0.000	0.000	0.000	0.000	0.011	0.000	0.112	0.000	30.572	20.188	8.206
5.937	0.008	0.042	0.000	0.000	0.193	0.000	0.000	0.000	0.012	0.000	0.309	0.000	37.931	11.962	11.087
6.605	0.013	0.018	0.195	0.015	0.000	0.071	0.000	0.000	0.032	0.000	0.159	0.000	46.002	14.391	10.140
4.346	0.000	0.038	0.000	0.000	0.528	0.038	0.000	0.000	0.000	0.000	0.565	0.000	76.688	20.593	7.808
21.139	0.000	0.000	0.000	0.000	0.172	0.000	0.000	0.000	0.033	0.000	0.110	0.000	46.920	29.852	9.868
0.595	0.106	0.000	0.000	0.000	0.757	0.000	0.000	0.000	0.012	0.025	0.061	0.000	57.783	10.756	14.534
3.499	0.000	0.000	0.075	0.000	0.000	0.041	0.000	0.000	0.012	0.000	0.369	0.000	57.751	8.044	5.585
6.571	0.000	0.000	0.000	0.000	0.000	0.036	0.000	0.000	0.000	0.000	0.266	0.000	31.625	21.086	7.752
10.434	0.019	0.000	0.123	0.000	0.000	0.000	0.000	0.000	0.000	0.021	0.050	0.000	18.317	20.920	14.149
18.190	0.006	0.000	0.000	0.000	0.000	0.000	0.000	0.000	0.020	0.000	0.304	0.000	17.835	4.406	16.398
10.315	0.000	0.018	0.000	0.016	0.508	0.000	0.000	0.000	0.011	0.000	0.163	0.000	19.011	16.228	12.145
3.290	0.007	0.000	0.000	0.000	0.173	0.000	0.000	0.000	0.000	0.000	0.111	0.000	15.786	9.648	17.346
7.077	0.012	0.000	0.060	0.028	0.305	0.033	0.000	0.000	0.010	0.000	0.441	0.000	29.415	9.720	8.558
1.629	0.011	0.092	0.000	0.026	0.281	0.061	0.000	0.000	0.000	0.000	1.082	0.000	27.605	16.477	30.479
1.807	0.000	0.000	0.000	0.000	0.000	0.000	0.000	0.000	0.000	0.000	1.174	0.000	28.273	6.727	14.184
5.985	0.000	0.000	0.071	0.051	0.000	0.039	0.000	0.000	0.000	0.000	0.233	0.000	29.974	13.857	14.299
8.285	0.000	0.000	0.000	0.016	0.172	0.000	0.000	0.000	0.011	0.000	0.055	0.000	61.876	12.578	13.621
7.871	0.007	0.037	0.000	0.016	0.171	0.074	0.000	0.000	0.000	0.000	0.000	0.000	40.265	8.955	9.481
9.241	0.000	0.000	0.000	0.030	0.000	0.000	0.000	0.000	0.010	0.000	0.051	0.000	23.067	6.790	11.057
13.733	0.000	0.000	0.072	0.017	0.184	0.040	0.000	0.000	0.188	0.000	0.355	0.000	45.142	22.294	7.998
2.541	0.000	0.000	0.000	0.000	0.000	0.063	0.000	0.000	0.000	0.000	0.281	0.000	26.039	7.820	13.193
6.097	0.006	0.000	0.060	0.028	1.064	0.033	0.000	0.000	0.126	0.000	0.293	0.000	44.708	5.061	4.281
10.647	0.007	0.000	0.000	0.030	0.000	0.035	0.000	0.000	0.010	0.000	0.052	0.000	42.901	19.478	7.021
2.924	0.007	0.000	0.071	0.000	0.000	0.000	0.018	0.000	0.023	0.000	0.175	0.000	29.935	4.831	13.674
7.584	0.000	0.000	0.275	0.022	0.467	0.000	0.000	0.000	0.045	0.000	0.225	0.090	15.696	6.626	6.543
2.894	0.000	0.000	0.000	0.000	0.000	0.000	0.000	0.000	0.020	0.000	0.925	0.000	15.676	6.056	9.712
2.305	0.000	0.000	0.084	0.020	0.000	0.000	0.000	0.000	0.000	0.000	0.480	0.000	9.564	5.448	15.150
4.817	0.000	0.000	0.111	0.027	0.000	0.000	0.000	0.000	0.000	0.000	0.091	0.000	29.529	17.348	6.520
3.465	0.000	0.000	0.176	0.000	0.000	0.048	0.000	0.248	0.000	0.000	0.216	0.000	8.216	2.738	19.239
4.440	0.000	0.000	0.064	0.015	0.000	0.035	0.000	0.000	0.000	0.000	0.052	0.000	17.625	10.740	9.379
5.186	0.000	0.000	0.130	0.000	0.330	0.000	0.000	0.000	0.000	0.000	0.106	0.000	5.124	10.656	20.840
13.938	0.000	0.000	0.000	0.014	0.000	0.032	0.000	0.000	0.000	0.000	0.096	0.000	26.379	15.549	8.920
10.959	0.000	0.000	0.000	0.000	0.590	0.000	0.000	0.000	0.000	0.000	0.284	0.000	25.105	9.093	8.930
8.018	0.000	0.000	0.000	0.021	0.000	0.000	0.000	0.000	0.000	0.000	0.072	0.000	35.716	26.005	4.666
6.805	0.000	0.030	0.106	0.025	0.000	0.000	0.027	0.000	0.000	0.000	0.435	0.000	35.120	12.851	7.149
3.455	0.030	0.000	0.000	0.000	0.190	0.041	0.000	0.000	0.000	0.000	0.609	0.000	9.714	15.824	21.771
1.936	0.000	0.000	0.000	0.106	0.000	0.000	0.000	0.000	0.073	0.000	0.000	0.000	40.952	8.023	8.875
2.788	0.010	0.000	0.094	0.000	0.000	0.000	0.000	0.000	0.000	0.000	0.077	0.000	9.119	11.333	27.321
2.134	0.010	0.052	0.187	0.000	0.000	0.000	0.000	0.000	0.000	0.000	0.000	0.000	6.014	18.532	14.186
1.263	0.009	0.000	0.000	0.000	0.218	0.000	0.022	0.000	0.000	0.000	0.351	0.000	3.413	12.284	32.975
4.690	0.000	0.000	0.000	0.016	0.000	0.000	0.000	0.000	0.000	0.000	0.055	0.000	11.978	13.101	11.602
12.063	0.024	0.000	0.000	0.000	0.407	0.000	0.000	0.000	0.000	0.000	0.392	0.000	24.361	16.928	6.427
7.768	0.000	0.000	0.090	0.000	0.000	0.000	0.000	0.000	0.000	0.000	0.000	0.000	37.134	11.980	15.932
11.623	0.000	0.000	0.000	0.000	0.385	0.000	0.000	0.000	0.012	0.000	0.123	0.000	37.877	9.758	5.646
25.280	0.000	0.000	0.080	0.000	0.204	0.000	0.020	0.000	0.000	0.000	0.131	0.000	24.237	30.385	7.751
4.701	0.000	0.018	0.000	0.030	0.321	0.069	0.000	0.000	0.000	0.000	1.186	0.000	92.132	9.387	4.955
10.645	0.000	0.000	0.066	0.000	0.335	0.036	0.000	0.000	0.000	0.000	0.054	0.000	35.931	9.075	6.369
5.605	0.049	0.000	0.000	0.014	0.153	0.000	0.000	0.000	0.000	0.000	0.098	0.000	41.525	9.025	6.248
12.095	0.000	0.000	0.000	0.000	0.298	0.064	0.000	0.000	0.000	0.000	0.000	0.000	38.861	11.991	5.723
5.722	0.000	0.000	0.125	0.000	0.478	0.069	0.000	0.000	0.020	0.000	0.664	0.000	54.560	7.344	4.084
8.850	0.000	0.018	0.129	0.000	0.329	0.000	0.000	0.000	0.000	0.000	0.158	0.000	40.238	17.795	6.992
3.581	0.000	0.000	0.062	0.000	0.000	0.000	0.000	0.000	0.000	0.000	0.102	0.000	38.352	15.018	5.439
16.123	0.000	0.000	0.000	0.031	0.000	0.036	0.000	0.000	0.064	0.000	0.320	0.000	63.386	4.158	5.387
8.412	0.006	0.000	0.063	0.030	0.000	0.035	0.016	0.000	0.000	0.000	0.309	0.000	49.516	21.669	4.875
17.177	0.007	0.000	0.000	0.017	0.186	0.000	0.000	0.000	0.000	0.000	0.179	0.000	49.485	27.592	8.274
6.259	0.000	0.000	0.076	0.036	0.195	0.084	0.000	0.000	0.000	0.000	0.501	0.000	39.494	33.856	7.945
5.667	0.000	0.000	2.183	0.000	0.795	0.686	0.000	0.000	0.000	0.000	0.000	0.308	51.777	14.553	7.985
8.596	0.000	0.000	0.000	0.000	0.503	0.000	0.000	0.000	0.064	0.000	0.808	0.000	48.249	18.209	11.783
7.090	0.000	0.000	0.000	0.018	0.000	0.000	0.000	0.000	0.000	0.000	0.063	0.000	34.748	10.714	7.348
7.542	0.000	0.042	0.076	0.018	0.389	0.000	0.000	0.000	0.000	0.000	0.000	0.000	47.853	20.227	6.488
1.198	0.010	0.000	0.000	0.000	0.000	0.000	0.000	0.000	0.000	0.000	0.245	0.000	4.126	15.680	28.964
19.889	0.000	0.000	0.000	0.000	0.000	0.036	0.000	0.000	0.000	0.000	0.371	0.000	29.032	23.343	8.453
20.292	0.009	0.000	0.000	0.000	0.000	0.000	0.000	0.000	0.000	0.000	0.068	0.000	39.907	29.576	13.606
7.120	0.000	0.000	0.096	0.000	0.488	0.053	0.000	0.000	0.000	0.000	0.157	0.000	40.087	12.830	6.450
11.516	0.000	0.000	0.000	0.000	0.000	0.000	0.000	0.000	0.000	0.000	0.137	0.000	37.907	12.759	4.899
7.668	0.000	0.000	0.154	0.018	0.000	0.000	0.000	0.000	0.025	0.000	0.126	0.000	30.604	17.587	11.826
3.463	0.000	0.000	0.000	0.000	0.695	0.000	0.000	0.000	0.000	0.000	2.064	0.000	24.712	6.010	12.565
6.837	0.000	0.000	0.000	0.000	0.000	0.000	0.000	0.000	0.000	0.000	0.262	0.000	25.432	9.375	9.342
4.409	0.000	0.045	0.080	0.019	0.000	0.000	0.000	0.000	0.000	0.000	0.394	0.000	9.648	17.502	11.037
5.532	0.000	0.000	0.000	0.018	0.000	0.000	0.000	0.000	0.000	0.000	0.062	0.000	7.141	10.741	11.789
5.956	0.000	0.000	0.142	0.000	0.181	0.039	0.000	0.000	0.000	0.024	0.000	0.000	26.325	9.855	10.265
8.763	0.015	0.000	0.000	0.017	0.000	0.040	0.000	0.000	0.000	0.000	0.120	0.000	32.292	7.501	7.473
4.741	0.000	0.000	0.157	0.075	0.000	0.086	0.000	0.000	0.000	0.000	0.386	0.000	49.643	12.513	8.273
6.490	0.000	0.000	0.075	0.000	0.000	0.041	0.000	0.000	0.000	0.000	0.366	0.000	27.146	28.801	8.870
12.959	0.000	0.022	0.000	0.000	0.000	0.000	0.000	0.000	0.013	0.000	0.130	0.000	19.553	17.244	8.191
11.212	0.007	0.000	0.068	0.033	0.348	0.075	0.000	0.000	0.011	0.000	0.112	0.000	41.390	10.364	6.453
8.751	0.000	0.000	0.000	0.019	0.000	0.000	0.000	0.000	0.026	0.000	0.129	0.000	25.328	12.176	15.433
4.427	0.000	0.000	0.000	0.033	0.000	0.000	0.000	0.000	0.000	0.000	0.000	0.000	17.269	13.134	13.571
6.095	0.000	0.000	0.065	0.078	0.166	0.000	0.000	0.000	0.011	0.000	0.000	0.000	36.613	26.440	6.465
14.788	0.000	0.000	0.000	0.014	0.602	0.000	0.015	0.000	0.010	0.000	0.338	0.000	35.246	12.223	8.122
5.434	0.000	0.025	0.000	0.042	0.000	0.049	0.000	0.000	0.000	0.000	0.073	0.000	24.497	13.567	8.558
7.493	0.000	0.000	0.000	0.019	0.000	0.000	0.000	0.000	0.000	0.000	0.000	0.000	21.651	19.363	6.164
8.322	0.000	0.000	0.077	0.018	0.000	0.000	0.000	0.000	0.101	0.000	0.696	0.000	14.632	12.767	21.668
10.257	0.000	0.000	0.145	0.017	0.000	0.040	0.000	0.000	0.071	0.000	0.178	0.000	52.719	17.249	7.285
20.056	0.000	0.000	0.000	0.033	0.000	0.000	0.000	0.000	0.113	0.000	0.113	0.000	20.735	11.301	8.868
6.354	0.000	0.000	0.000	0.000	0.000	0.000	0.000	0.000	0.000	0.000	0.000	0.000	38.677	12.367	15.026
7.656	0.012	0.000	0.119	0.014	0.000	0.000	0.000	0.000	0.000	0.000	0.049	0.000	42.132	6.658	7.692
14.906	0.000	0.000	0.000	0.026	0.141	0.000	0.000	0.000	0.009	0.000	0.453	0.000	34.868	11.772	10.285
10.581	0.000	0.000	0.060	0.014	0.153	0.033	0.015	0.000	0.029	0.000	0.196	0.000	43.563	11.682	7.876
5.231	0.000	0.047	0.057	0.000	0.000	0.031	0.000	0.000	0.000	0.000	0.140	0.000	35.915	21.608	6.852
5.275	0.000	0.000	0.718	0.000	0.000	0.197	0.000	0.000	0.000	0.000	0.294	0.000	13.951	10.196	11.273
1.899	0.000	0.000	0.000	0.046	0.000	0.000	0.000	0.000	0.000	0.000	0.000	0.000	11.815	17.213	22.013
5.802	0.000	0.000	0.750	0.000	0.425	0.046	0.000	0.000	0.000	0.000	0.954	0.000	40.944	4.947	5.123
5.602	0.009	0.000	0.085	0.000	0.000	0.000	0.000	0.000	0.000	0.000	0.278	0.000	32.307	10.222	12.860
5.623	0.000	0.000	0.188	0.067	0.480	0.104	0.000	0.000	0.000	0.000	0.154	0.000	30.751	18.317	10.021
25.972	0.000	0.000	0.212	0.025	0.000	0.000	0.000	0.000	0.052	0.000	0.520	0.000	36.253	13.564	6.905
6.694	0.000	0.048	0.000	0.061	0.000	0.000	0.022	0.000	0.042	0.000	0.141	0.000	52.195	17.805	6.182
6.849	0.000	0.000	0.000	0.029	0.000	0.066	0.000	0.000	0.000	0.000	0.196	0.000	27.982	7.455	8.731
14.394	0.000	0.000	0.000	0.000	0.000	0.000	0.000	0.000	0.030	0.000	0.050	0.000	29.110	11.981	9.073
5.608	0.000	0.000	0.000	0.000	0.279	0.000	0.000	0.000	0.000	0.000	0.269	0.000	27.953	10.224	7.525
7.927	0.000	0.000	0.000	0.014	0.000	0.000	0.000	0.000	0.000	0.000	0.187	0.000	23.337	10.156	5.151
16.047	0.000	0.000	0.000	0.000	0.000	0.000	0.000	0.000	0.013	0.000	0.696	0.000	35.797	8.318	7.847
12.221	0.014	0.019	0.139	0.000	0.000	0.038	0.000	0.000	0.000	0.000	0.000	0.000	54.589	14.109	5.952
6.698	0.000	0.000	0.000	0.032	0.000	0.000	0.000	0.000	0.000	0.000	0.223	0.000	22.500	11.388	6.744

ENSG00000264769	ENSG00000224711	ENSG00000124613	ENSG00000261821	ENSG00000274569	ENSG00000240882	ENSG00000198824	ENSG00000244230	ENSG00000258505	ENSG00000135740	ENSG00000261553	ENSG00000223883	ENSG00000146122	ENSG00000201086	ENSG00000270832	ENSG00000238616
0.440	0.000	1.075	0.000	0.000	0.343	5.993	0.000	0.000	0.502	0.101	0.000	13.935	0.000	0.044	0.000
0.150	0.000	0.609	0.009	0.000	0.103	3.350	0.000	0.000	0.804	0.081	0.000	3.564	0.000	0.037	0.000
0.133	0.000	1.484	0.000	0.047	0.214	13.858	0.000	0.000	0.712	0.126	0.000	2.576	0.000	0.000	0.000
0.377	0.000	1.659	0.000	0.053	0.259	9.055	0.000	0.000	1.120	0.076	0.000	18.223	0.000	0.000	0.000
0.042	0.000	1.611	0.005	0.000	0.019	6.479	0.000	0.000	0.240	0.034	0.000	2.793	0.000	0.042	0.000
0.563	0.000	1.281	0.004	0.093	0.258	13.836	0.066	0.000	5.335	0.165	0.030	9.793	0.000	0.000	0.000
0.187	0.000	1.160	0.013	0.000	0.154	4.723	0.000	0.000	1.446	0.111	0.000	1.695	0.000	0.037	0.000
0.079	0.000	1.443	0.000	0.000	0.091	4.224	0.078	0.000	0.484	0.144	0.000	20.064	0.000	0.000	0.000
0.000	0.000	0.770	0.005	0.000	0.188	4.394	0.181	0.000	0.526	0.172	0.000	7.554	0.000	0.000	0.000
0.133	0.000	0.634	0.000	0.187	0.304	3.977	0.000	0.000	0.703	0.179	0.000	3.483	0.000	0.000	0.000
0.457	0.000	0.763	0.026	0.000	0.150	4.388	0.130	0.000	1.748	0.136	0.000	7.408	0.000	0.000	0.000
0.405	0.000	1.258	0.012	0.095	0.201	8.470	0.000	0.000	0.407	0.182	0.000	4.419	0.000	0.033	0.000
0.244	0.000	1.915	0.012	0.246	0.224	7.818	0.069	0.000	0.521	0.141	0.000	29.602	0.000	0.000	0.000
0.261	0.000	2.417	0.004	0.000	0.164	4.894	0.065	0.000	0.684	0.057	0.000	36.777	0.154	0.000	0.000
0.145	0.000	1.116	0.000	0.000	0.133	6.583	0.000	0.000	0.377	0.039	0.000	13.996	0.000	0.000	0.000
0.620	0.000	1.189	0.004	0.046	0.105	6.747	0.000	0.000	0.400	0.114	0.000	16.430	0.000	0.065	0.000
0.113	0.000	1.441	0.000	0.000	0.259	10.973	0.075	0.000	0.645	0.182	0.000	2.684	0.000	0.000	0.000
0.045	0.000	0.411	0.000	0.000	0.266	7.254	0.089	0.035	1.577	0.138	0.000	15.977	0.000	0.000	0.000
0.068	0.000	0.550	0.008	0.000	0.140	3.150	0.135	0.000	0.727	0.073	0.000	9.370	0.000	0.034	0.000
0.254	0.000	0.922	0.000	0.000	0.317	9.450	0.144	0.000	0.370	0.142	0.000	7.145	0.000	0.036	0.000
0.000	0.000	0.906	0.009	0.000	0.142	2.733	0.616	0.000	0.458	0.156	0.000	11.967	0.000	0.000	0.000
0.720	0.000	1.431	0.005	0.000	0.184	10.614	0.080	0.000	1.106	0.248	0.000	26.515	0.000	0.000	0.000
0.078	0.000	1.626	0.004	0.000	0.287	9.025	0.156	0.000	0.557	0.105	0.000	8.649	0.000	0.039	0.000
0.067	0.000	0.655	0.008	0.094	0.137	6.591	0.066	0.000	0.626	0.031	0.000	2.095	0.000	0.000	0.000
0.111	0.000	1.126	0.000	0.000	0.102	6.139	0.000	0.000	0.385	0.135	0.000	6.130	0.000	0.000	0.000
0.616	0.000	2.050	0.018	0.054	0.265	10.047	0.000	0.000	0.908	0.208	0.000	28.844	0.000	0.000	0.000
0.174	0.000	1.638	0.015	0.000	0.360	4.621	0.347	0.000	1.593	0.076	0.000	15.812	0.206	0.000	0.000
0.230	0.000	1.527	0.009	0.108	0.633	10.879	0.305	0.000	0.916	0.165	0.000	6.408	0.000	0.000	0.000
0.306	0.000	1.899	0.035	0.000	0.140	4.660	0.152	0.000	1.123	0.077	0.000	27.562	0.000	0.076	0.000
0.269	0.000	1.016	0.022	0.108	0.123	7.741	0.000	0.000	1.181	0.057	0.000	7.114	0.000	0.000	0.000
0.063	0.000	1.406	0.000	0.045	0.232	5.762	0.189	0.000	0.709	0.170	0.000	2.279	0.000	0.000	0.000
0.662	0.000	2.464	0.011	0.000	0.137	7.160	0.263	0.000	0.974	0.120	0.000	1.923	0.000	0.000	0.000
0.077	0.000	1.050	0.000	0.000	0.141	3.448	0.153	0.000	0.804	0.078	0.000	11.776	0.000	0.000	0.000
0.074	0.000	1.427	0.046	0.000	0.119	4.846	0.074	0.000	0.791	0.140	0.033	2.985	0.000	0.000	0.000
0.392	0.000	2.667	0.009	0.000	0.216	10.828	0.156	0.000	0.301	0.053	0.000	3.641	0.000	0.000	0.000
0.391	0.000	2.226	0.028	0.050	0.114	7.273	0.000	0.000	0.971	0.129	0.000	9.173	0.000	0.035	0.000
1.018	0.000	3.062	0.209	0.048	0.311	5.252	0.135	0.000	2.481	0.046	0.000	160.458	0.160	0.000	0.000
0.339	0.000	1.405	0.004	0.048	0.202	8.346	0.000	0.000	0.658	0.160	0.000	15.713	0.000	0.000	0.000
0.283	0.000	2.627	0.009	0.000	0.130	10.191	0.000	0.000	0.983	0.125	0.000	9.622	0.000	0.040	0.000
0.543	0.000	1.449	0.000	0.059	0.077	9.971	0.166	0.000	0.607	0.090	0.000	3.720	0.000	0.083	0.000
0.748	0.000	1.123	0.005	0.059	0.076	7.554	0.165	0.000	0.708	0.129	0.000	8.637	0.000	0.041	0.000
0.737	0.000	2.249	0.088	0.050	0.145	7.958	1.886	0.000	1.302	0.057	0.000	4.034	0.000	0.000	0.000
0.270	0.000	2.003	0.000	0.109	0.124	9.269	0.000	0.000	0.373	0.062	0.000	3.670	0.000	0.000	0.000
0.464	0.000	2.260	0.004	0.047	0.122	10.185	0.000	0.000	0.451	0.080	0.000	5.493	0.157	0.000	0.359
1.060	0.000	3.198	0.020	0.000	0.178	10.629	0.000	0.000	0.551	0.090	0.000	4.340	0.000	0.000	0.000
0.483	0.000	2.814	0.008	0.000	0.190	10.306	0.137	0.000	1.393	0.046	0.000	166.087	0.326	0.000	0.000
1.784	0.000	1.813	0.095	0.589	0.192	8.731	0.000	0.000	1.132	0.041	0.034	13.127	0.000	0.113	0.000
0.384	0.000	1.602	0.009	0.217	0.300	10.666	0.076	0.000	0.950	0.083	0.000	14.909	0.000	0.000	0.000
0.200	0.000	2.173	0.023	0.000	0.138	11.853	0.000	0.000	0.547	0.081	0.000	3.882	0.000	0.000	0.000
0.283	0.000	0.403	0.000	0.000	0.087	4.946	0.188	0.000	0.239	0.187	0.000	10.376	0.446	0.000	0.000
0.348	0.000	1.234	0.007	0.045	0.305	7.507	0.063	0.000	0.864	0.239	0.000	18.032	0.000	0.000	0.000
0.068	0.000	1.010	0.000	0.000	0.172	3.838	0.068	0.000	0.353	0.105	0.000	3.112	0.000	0.000	0.000
0.139	0.000	2.255	0.004	0.049	0.159	10.135	0.069	0.000	0.554	0.173	0.000	7.679	0.000	0.000	0.000
0.245	0.000	1.594	0.000	0.043	0.225	5.859	0.122	0.000	0.188	0.045	0.000	4.687	0.000	0.000	0.000
0.169	0.000	0.650	0.000	0.000	0.414	12.920	0.561	0.000	1.482	0.243	0.000	2.988	0.000	0.000	0.000
0.000	0.000	2.873	0.000	0.000	0.202	5.649	0.876	0.000	0.229	0.000	0.000	99.091	0.000	0.000	0.000
0.255	0.000	2.130	0.000	0.051	0.151	9.099	0.073	0.000	0.542	0.054	0.000	1.052	0.000	0.072	0.000
0.241	0.000	1.032	0.008	0.049	0.332	6.940	0.000	0.000	0.487	0.051	0.000	6.825	0.000	0.034	0.000
0.480	0.000	0.798	0.004	0.145	0.189	9.671	0.068	0.000	0.399	0.083	0.000	10.997	0.000	0.000	0.000
0.128	0.000	2.401	0.000	0.226	0.161	5.303	0.000	0.000	0.139	0.034	0.000	33.787	0.000	0.000	0.000
0.369	0.000	3.287	0.013	0.052	0.254	13.141	0.074	0.000	0.990	0.129	0.000	75.019	0.000	0.037	0.000
0.000	0.000	1.488	0.007	0.000	0.242	7.327	0.117	0.000	0.719	0.118	0.000	2.318	0.000	0.058	0.000
0.244	0.000	2.275	0.010	0.000	0.350	9.293	0.121	0.000	1.119	0.148	0.000	24.199	0.000	0.000	0.000
0.851	0.000	2.184	0.004	0.092	0.285	8.684	0.000	0.000	1.173	0.075	0.088	2.831	0.000	0.000	0.000
0.219	0.000	2.489	0.029	0.000	0.201	9.507	0.000	0.000	0.261	0.113	0.033	37.528	0.000	0.000	0.000
0.094	0.000	0.748	0.005	0.000	0.237	6.578	1.585	0.000	0.949	0.069	0.042	16.814	0.000	0.046	0.000
0.129	0.000	1.048	0.000	0.000	0.265	8.181	0.384	0.000	0.247	0.121	0.000	4.369	0.000	0.000	0.000
0.043	0.000	1.003	0.000	0.000	0.157	4.639	0.085	0.000	0.294	0.115	0.000	0.940	0.000	0.000	0.000
0.114	0.000	2.667	0.000	0.000	0.366	12.327	0.000	0.000	0.225	0.038	0.000	1.495	0.000	0.000	0.000
0.000	0.000	1.111	0.000	0.000	0.165	3.207	0.358	0.000	0.056	0.079	0.040	1.827	0.000	0.000	0.000
0.033	0.000	1.100	0.000	0.000	0.075	9.328	0.131	0.000	0.204	0.053	0.000	1.654	0.000	0.033	0.000
0.000	0.000	0.579	0.030	0.094	0.152	2.112	0.132	0.000	0.503	0.054	0.000	14.956	0.000	0.000	0.000
0.301	0.000	1.519	0.003	0.042	0.083	5.217	0.000	0.000	0.690	0.101	0.027	4.375	0.000	0.000	0.000
0.415	0.000	2.186	0.007	0.167	0.136	4.309	0.000	0.000	1.218	0.040	0.000	1.258	0.000	0.000	0.000
0.226	0.000	1.899	0.015	0.000	0.124	8.109	0.000	0.000	0.371	0.055	0.000	3.081	0.000	0.045	0.000
0.109	0.000	2.891	0.006	0.154	0.225	6.554	0.000	0.000	0.271	0.044	0.000	32.374	0.000	0.000	0.000
0.114	0.000	0.696	0.009	0.054	0.122	5.931	0.000	0.000	0.549	0.159	0.000	3.547	0.000	0.000	0.000
0.228	0.000	5.448	0.182	0.322	0.523	10.810	0.454	0.000	2.702	0.031	0.000	12.130	0.000	0.000	0.000
0.144	0.000	1.474	0.000	0.000	0.154	4.600	0.286	0.000	0.468	0.207	0.000	5.890	0.000	0.000	0.000
0.048	0.000	2.065	0.000	0.135	0.044	3.535	0.000	0.000	0.199	0.122	0.000	2.873	0.000	0.047	0.000
0.044	0.033	1.261	0.000	0.000	0.040	2.999	0.349	0.000	0.241	0.041	0.000	3.082	0.000	0.000	0.000
0.034	0.000	1.993	0.012	0.097	0.095	5.552	0.000	0.000	0.211	0.097	0.000	2.043	0.000	0.034	0.000
0.041	0.000	2.153	0.009	0.000	0.262	6.384	0.000	0.000	0.958	0.066	0.000	50.790	0.000	0.000	0.000
0.277	0.000	2.055	0.016	0.000	0.021	4.931	0.092	0.000	0.518	0.087	0.000	12.637	0.000	0.046	0.000
0.232	0.000	1.312	0.004	0.000	0.195	7.662	0.000	0.000	0.453	0.036	0.000	33.703	0.000	0.000	0.000
0.123	0.000	1.265	0.000	0.058	0.056	8.450	0.000	0.000	0.162	0.044	0.000	1.978	0.000	0.000	0.000
0.774	0.000	1.558	0.000	0.136	0.385	27.393	0.064	0.000	0.459	0.152	0.000	21.515	0.000	0.064	0.000
0.404	0.000	1.481	0.050	0.095	0.185	12.747	0.401	0.027	0.374	0.159	0.000	5.432	0.000	0.033	0.000
0.338	0.000	1.979	0.000	0.087	0.169	9.346	0.061	0.000	0.792	0.112	0.000	10.382	0.000	0.000	0.000
0.239	0.000	1.275	0.007	0.000	0.220	5.969	0.000	0.000	0.957	0.056	0.000	27.672	0.000	0.000	0.000
0.415	0.000	1.979	0.007	0.000	0.308	7.960	0.191	0.000	2.403	0.129	0.000	3.307	0.151	0.063	0.000
0.165	0.000	0.968	0.000	0.047	0.560	3.148	0.131	0.000	0.959	0.053	0.000	11.927	0.000	0.065	0.000
0.223	0.000	1.957	0.000	0.090	0.161	7.561	0.000	0.000	0.843	0.133	0.000	25.684	0.000	0.032	0.000
0.301	0.000	2.365	0.000	0.000	0.184	13.801	0.000	0.000	1.308	0.058	0.000	13.648	0.000	0.000	0.000
0.676	0.000	0.840	0.007	0.000	0.192	5.431	0.064	0.000	1.334	0.061	0.000	66.391	0.152	0.064	0.000
0.672	0.000	2.257	0.004	0.053	0.446	10.454	0.074	0.000	2.076	0.111	0.000	37.643	0.000	0.037	0.000
0.274	0.000	2.365	0.009	0.000	0.449	8.397	0.000	0.000	0.618	0.090	0.000	1.828	0.185	0.000	0.000
0.160	0.000	1.877	0.000	0.225	0.512	11.284	3.809	0.000	1.111	0.279	0.000	9.929	0.000	0.633	0.000
0.606	0.000	1.999	0.000	0.285	0.510	6.516	0.000	0.000	1.826	0.095	0.000	13.463	0.000	0.000	0.000
0.274	0.000	2.013	0.013	0.055	0.180	8.217	0.000	0.000	0.436	0.095	0.000	4.129	0.000	0.000	0.000
0.313	0.000	2.789	0.004	0.055	0.323	5.325	0.155	0.000	1.733	0.047	0.000	14.909	0.000	0.000	0.000
0.102	0.000	0.636	0.012	0.000	0.140	3.587	0.000	0.000	0.132	0.069	0.000	2.430	0.000	0.000	0.000
0.265	0.000	1.212	0.128	0.047	0.061	5.811	0.000	0.000	0.689	0.071	0.000	6.644	0.000	0.000	0.000
0.342	0.000	2.921	0.019	0.000	0.059	4.711	0.000	0.000	0.999	0.046	0.000	49.073	0.000	0.000	0.000
0.294	0.000	1.826	0.000	0.138	0.180	7.399	0.195	0.000	1.038	0.046	0.000	3.175	0.000	0.049	0.000
0.343	0.000	1.900	0.000	0.000	0.158	8.934	0.000	0.000	0.571	0.093	0.000	7.569	0.000	0.000	0.000
0.196	0.030	2.224	0.004	0.000	0.270	3.495	0.000	0.000	0.143	0.101	0.000	25.568	0.000	0.000	0.000
0.174	0.000	1.621	0.000	0.049	0.240	7.622	0.069	0.000	1.482	0.089	0.000	38.827	0.000	0.000	0.000
0.196	0.000	2.025	0.000	0.046	0.225	11.380	0.065	0.000	0.371	0.225	0.000	1.971	0.000	0.000	0.000
0.041	0.000	0.841	0.023	0.000	0.132	6.440	0.082	0.000	0.588	0.160	0.000	2.826	0.000	0.000	0.000
0.039	0.000	1.261	0.000	0.000	0.071	3.127	0.154	0.000	0.109	0.156	0.035	0.857	0.000	0.000	0.000
0.182	0.000	2.013	0.004	0.051	0.050	8.399	0.000	0.000	0.490	0.059	0.000	4.409	0.000	0.036	0.000
0.263	0.000	2.241	0.009	0.000	0.138	6.708	0.075	0.000	0.518	0.147	0.000	35.900	0.000	0.000	0.000
0.643	0.000	1.666	0.000	0.000	0.406	9.825	0.959	0.000	0.626	0.141	0.000	9.405	0.000	0.080	0.000
0.114	0.000	0.796	0.022	0.162	0.175	7.743	0.076	0.000	0.456	0.031	0.069	2.133	0.000	0.076	0.000
0.162	0.000	1.095	0.005	0.057	0.260	4.891	0.242	0.000	0.286	0.147	0.000	4.367	0.000	0.000	0.000
0.489	0.000	1.507	0.000	0.049	0.128	8.362	0.070	0.000	0.766	0.137	0.000	6.118	0.000	0.035	0.000
0.162	0.000	2.513	0.005	0.000	0.241	8.889	0.161	0.000	0.404	0.093	0.000	51.776	0.000	0.000	0.000
0.035	0.000	2.004	0.008	0.000	0.274	8.282	0.140	0.000	0.332	0.123	0.000	4.759	0.000	0.000	0.000
0.200	0.000	1.634	0.000	0.000	0.383	12.617	0.066	0.000	0.368	0.104	0.000	5.383	0.000	0.000	0.000
0.212	0.000	1.858	0.017	0.085	0.222	8.672	1.022	0.000	0.317	0.041	0.000	10.958	0.000	0.000	0.000
0.091	0.000	1.839	0.005	0.000	0.230	7.653	0.091	0.000	0.241	0.110	0.000	13.684	0.000	0.000	0.000
0.159	0.000	1.476	0.000	0.056	0.183	6.355	0.000	0.000	0.493	0.038	0.000	10.934	0.000	0.000	0.000
0.119	0.000	0.811	0.050	0.000	0.127	6.364	0.000	0.000	0.119	0.107	0.000	64.410	0.000	0.000	0.000
0.297	0.000	2.012	0.000	0.105	0.170	11.611	0.222	0.000	1.216	0.075	0.000	53.200	0.000	0.000	0.000
0.248	0.000	1.090	0.020	0.050	0.130	4.697	0.000	0.000	1.108	0.095	0.000	15.475	0.000	0.000	0.000
0.116	0.000	0.861	0.000	0.000	0.424	5.941	0.689	0.000	0.804	0.062	0.000	6.631	0.000	0.115	0.000
0.519	0.000	2.273	0.007	0.043	0.462	8.076	0.182	0.000	1.098	0.062	0.000	6.328	0.000	0.000	0.000
0.425	0.000	0.356	0.006	0.080	0.143	7.555	0.113	0.000	0.232	0.065	0.000	3.937	0.000	0.000	0.000
0.153	0.000	4.170	0.000	0.216	0.548	8.208	0.122	0.000	1.522	0.087	0.028	17.185	0.000	0.030	0.000
0.233	0.000	1.607	0.000	0.000	0.294	7.629	0.348	0.000	0.427	0.102	0.000	3.879	0.000	0.000	0.000
0.184	0.000	1.262	0.000	0.000	0.042	5.703	3.472	0.000	0.258	0.099	0.000	5.765	0.000	0.000	0.000
0.033	0.000	1.217	0.000	0.000	0.152	5.361	0.000	0.000	0.141	0.130	0.000	9.367	0.000	0.000	0.000
0.810	0.000	0.923	0.010	0.000	0.293	8.498	1.357	0.000	1.355	0.121	0.000	16.435	0.000	0.042	0.000
0.347	0.000	1.016	0.005	0.000	0.199	5.451	0.259	0.000	0.424	0.117	0.000	7.923	0.000	0.043	0.000
0.145	0.000	1.892	0.000	0.068	0.265	5.193	1.822	0.000	0.626	0.045	0.000	11.761	0.000	0.096	0.000
0.920	0.000	1.097	0.043	0.076	0.248	4.064	0.323	0.000	1.063	0.080	0.000	12.939	0.000	0.000	0.000
0.264	0.000	2.033	0.000	0.062	0.141	10.161	0.000	0.000	1.142	0.148	0.000	8.718	0.000	0.044	0.000
0.306	0.000	1.509	0.003	0.173	0.225	13.497	0.000	0.000	0.433	0.149	0.000	2.863	0.000	0.000	0.000
0.126	0.000	3.000	0.011	0.044	0.173	7.730	0.000	0.000	0.574	0.063	0.000	8.669	0.000	0.031	0.000
0.168	0.000	1.587	0.006	0.000	0.129	9.066	0.223	0.000	0.291	0.121	0.000	13.851	0.000	0.000	0.000
0.292	0.000	1.130	0.010	0.000	0.121	7.304	0.116	0.000	0.565	0.106	0.000	2.397	0.000	0.000	0.158
0.277	0.000	1.876	0.014	0.056	0.272	5.598	0.000	0.000	0.682	0.053	0.000	13.505	0.000	0.000	0.000
0.925	0.000	1.549	0.004	0.000	0.277	10.599	0.000	0.000	1.400	0.115	0.000	4.825	0.000	0.070	0.000
0.070	0.000	1.659	0.000	0.000	0.192	7.653	0.069	0.000	0.308	0.085	0.000	1.468	0.000	0.000	0.000

ENSG00000227973	ENSG00000278895	ENSG00000232059	ENSG00000248537	ENSG00000229494	ENSG00000230377	ENSG00000105497	ENSG00000204287	ENSG00000278983	ENSG00000138722	ENSG00000254917	ENSG00000268744	ENSG00000227416	ENSG00000238066	ENSG00000136758	ENSG00000202478
0.875	0.013	0.771	0.063	0.000	0.000	1.810	803.874	0.091	1.114	0.000	0.057	0.000	0.000	16.611	0.000
0.744	0.000	0.510	0.054	0.000	0.000	1.169	1441.366	0.053	0.260	0.000	0.049	0.000	0.000	16.776	0.000
0.804	0.000	0.454	0.000	0.000	0.000	2.421	773.243	0.190	2.230	0.019	0.094	0.044	0.000	18.314	0.000
0.589	0.000	2.863	0.054	0.000	0.000	2.550	357.904	0.126	0.851	0.000	0.115	0.000	0.000	9.584	0.000
1.192	0.024	0.490	0.000	0.000	0.000	1.404	1057.601	0.047	0.330	0.024	0.055	0.000	0.000	12.867	0.000
0.942	0.009	0.774	0.000	0.000	0.000	3.707	557.270	0.216	0.220	0.000	0.086	0.087	0.000	19.051	0.000
0.477	0.021	0.509	0.000	0.000	0.000	1.644	2916.180	0.053	0.983	0.000	0.040	0.049	0.000	15.976	0.000
0.449	0.011	0.307	0.000	0.000	0.000	1.102	1436.470	0.113	2.568	0.023	0.086	0.000	0.000	9.595	0.000
0.389	0.000	0.533	0.000	0.000	0.000	1.944	797.114	0.036	1.225	0.000	0.129	0.060	0.000	9.414	0.000
0.943	0.000	1.293	0.000	0.000	0.000	1.111	2855.521	0.021	2.116	0.000	0.086	0.000	0.000	14.302	0.000
0.881	0.000	1.334	0.000	0.000	0.000	1.839	1057.988	0.021	0.733	0.019	0.057	0.000	0.000	11.151	0.000
0.720	0.000	0.066	0.049	0.000	0.000	1.801	796.535	0.171	2.335	0.000	0.081	0.089	0.000	12.107	0.000
0.347	0.010	0.271	0.000	0.000	0.000	2.805	844.033	0.083	1.787	0.000	0.083	0.000	0.000	7.913	0.000
0.648	0.000	0.317	0.000	0.000	0.000	3.043	693.943	0.010	0.849	0.000	0.127	0.043	0.000	9.656	0.000
0.825	0.000	1.414	0.000	0.000	0.000	1.459	1068.162	0.115	1.053	0.000	0.126	0.000	0.000	9.314	0.000
0.510	0.000	0.191	0.000	0.000	0.000	2.157	1588.481	0.062	1.273	0.000	0.064	0.000	0.000	15.817	0.000
0.909	0.000	0.147	0.000	0.000	0.000	2.756	179.581	0.263	1.594	0.000	0.065	0.000	0.000	9.611	0.000
0.444	0.000	1.044	0.000	0.000	0.000	1.645	1445.507	0.028	0.575	0.000	0.126	0.000	0.000	9.436	0.000
0.628	0.000	0.596	0.049	0.000	0.000	1.738	1038.604	0.016	1.777	0.040	0.103	0.000	0.000	10.353	0.000
0.465	0.000	0.424	0.000	0.000	0.000	2.051	426.812	0.081	1.180	0.021	0.079	0.143	0.000	8.950	0.000
0.826	0.011	0.528	0.000	0.000	0.000	1.341	612.762	0.049	0.878	0.000	0.101	0.000	0.000	7.297	0.000
0.626	0.000	1.169	0.000	0.000	0.000	2.672	156.229	0.171	0.372	0.023	0.087	0.210	0.000	16.559	0.000
0.945	0.011	0.381	0.056	0.020	0.000	1.452	1403.670	0.186	0.683	0.000	0.051	0.051	0.000	8.979	0.000
0.757	0.000	0.454	0.096	0.000	0.000	0.950	739.044	0.016	0.588	0.000	0.072	0.000	0.000	13.757	0.000
0.684	0.011	0.361	0.053	0.000	0.000	2.013	910.760	0.147	2.876	0.000	0.008	0.049	0.000	10.921	0.000
0.438	0.000	0.525	0.055	0.000	0.000	2.345	529.134	0.183	1.200	0.000	0.033	0.152	0.000	10.182	0.000
0.495	0.000	0.933	0.063	0.000	0.000	2.195	1470.080	0.035	1.428	0.000	0.095	0.114	0.000	8.631	0.000
1.308	0.022	0.896	0.000	0.000	0.000	1.705	403.517	0.134	1.316	0.000	0.050	0.000	0.000	10.442	0.000
0.706	0.011	0.372	0.000	0.000	0.000	2.404	728.563	0.364	0.457	0.000	0.182	0.100	0.000	7.950	0.000
1.201	0.000	0.224	0.055	0.000	0.000	2.336	439.723	0.293	0.852	0.000	0.092	0.151	0.000	7.848	0.000
0.943	0.000	0.431	0.091	0.000	0.000	1.010	762.672	0.030	1.608	0.018	0.082	0.000	0.000	7.397	0.000
0.753	0.000	0.774	0.000	0.000	0.000	1.956	203.204	0.236	0.805	0.000	0.180	0.217	0.000	9.083	0.000
0.602	0.000	1.126	0.000	0.000	0.000	1.698	655.325	0.073	0.551	0.000	0.142	0.000	0.081	7.221	0.209
0.581	0.000	0.434	0.053	0.000	0.000	1.495	1177.612	0.342	1.157	0.022	0.113	0.097	0.000	8.114	0.000
0.669	0.000	0.458	0.000	0.000	0.000	1.033	479.349	0.112	1.643	0.000	0.051	0.000	0.000	13.217	0.000
0.404	0.010	0.346	0.000	0.018	0.000	1.766	662.510	0.085	1.426	0.000	0.023	0.140	0.000	11.020	0.000
1.061	0.039	0.727	0.000	0.000	0.000	3.068	319.582	0.221	0.385	0.000	0.125	0.134	0.071	11.803	0.000
0.674	0.000	0.330	0.000	0.017	0.000	2.834	1505.509	0.172	0.478	0.000	0.132	0.000	0.000	10.657	0.000
0.804	0.000	0.709	0.000	0.000	0.000	2.760	619.796	0.045	3.618	0.024	0.079	0.053	0.000	11.804	0.000
1.247	0.000	0.244	0.000	0.000	0.000	2.336	284.024	0.199	1.882	0.000	0.073	0.000	0.000	19.481	0.000
0.591	0.000	0.324	0.060	0.000	0.000	1.218	664.899	0.026	1.753	0.000	0.027	0.055	0.000	12.004	0.000
0.449	0.000	0.547	0.000	0.000	0.000	1.251	376.258	0.167	3.136	0.000	0.030	0.000	0.000	10.555	0.000
0.440	0.000	0.226	0.000	0.000	0.000	3.650	387.899	0.061	3.538	0.000	0.092	0.051	0.000	10.316	0.000
0.424	0.000	0.258	0.000	0.000	0.000	2.696	250.739	0.158	1.362	0.000	0.036	0.044	0.000	12.942	0.000
1.156	0.000	0.689	0.000	0.000	0.000	4.283	593.115	0.073	3.131	0.000	0.092	0.000	0.000	10.946	0.000
0.490	0.000	0.336	0.000	0.000	0.000	1.968	740.453	0.060	1.095	0.020	0.067	0.136	0.000	9.747	0.000
0.540	0.000	1.183	0.000	0.000	0.000	0.838	46.049	0.018	1.222	0.000	0.058	0.100	0.000	28.903	0.000
1.202	0.000	0.748	0.000	0.000	0.000	2.758	457.889	0.335	2.049	0.000	0.050	0.252	0.000	15.112	0.000
0.758	0.000	0.909	0.000	0.000	0.000	2.396	487.665	0.037	1.212	0.000	0.043	0.000	0.000	12.247	0.000
1.207	0.000	0.858	0.045	0.000	0.000	1.353	2266.881	0.040	2.774	0.000	0.109	0.000	0.000	12.782	0.000
0.765	0.000	0.986	0.046	0.000	0.000	1.736	412.492	0.025	2.023	0.018	0.096	0.083	0.000	10.931	0.000
1.016	0.000	1.126	0.000	0.000	0.000	1.463	1909.938	0.027	3.302	0.000	0.052	0.000	0.000	11.592	0.000
1.182	0.000	1.080	0.000	0.000	0.000	1.983	1623.489	0.033	2.976	0.000	0.128	0.000	0.000	12.898	0.000
0.783	0.000	0.716	0.000	0.000	0.000	3.374	898.588	0.034	1.855	0.000	0.146	1.406	0.000	10.904	0.000
1.202	0.000	1.428	0.081	0.000	0.000	1.558	191.315	0.304	1.100	0.033	0.061	0.074	0.000	11.600	0.000
0.626	0.042	1.144	0.000	0.000	0.000	1.945	209.576	0.000	1.425	0.000	0.064	0.000	0.000	21.346	0.000
0.571	0.000	0.284	0.105	0.000	0.000	1.614	406.208	0.075	1.264	0.000	0.055	0.000	0.000	11.283	0.000
1.275	0.000	0.470	0.000	0.000	0.000	2.347	551.082	0.088	2.762	0.000	0.052	0.091	0.072	11.972	0.000
1.413	0.010	0.200	0.000	0.000	0.000	1.677	451.038	0.109	2.612	0.000	0.052	0.090	0.000	19.025	0.000
1.636	0.009	0.311	0.000	0.016	0.000	2.219	1039.128	0.000	0.519	0.019	0.090	0.000	0.067	9.073	0.000
0.525	0.011	0.144	0.000	0.000	0.000	1.752	148.449	0.076	1.042	0.022	0.016	0.048	0.000	13.524	0.000
0.917	0.000	1.142	0.000	0.000	0.000	2.133	1081.801	0.112	1.366	0.034	0.051	0.000	0.123	8.819	0.000
0.911	0.009	0.357	0.000	0.000	0.000	2.379	194.337	0.436	0.923	0.018	0.073	0.160	0.000	9.131	0.000
0.884	0.009	0.829	0.000	0.000	0.000	1.711	783.002	0.119	1.679	0.000	0.021	0.043	0.000	12.358	0.000
0.985	0.000	0.711	0.000	0.000	0.000	1.744	178.091	0.029	2.542	0.000	0.016	0.000	0.000	12.405	0.000
0.933	0.000	1.461	0.000	0.000	0.000	1.830	441.158	0.156	0.594	0.000	0.081	0.000	0.000	6.992	0.000
0.822	0.000	0.501	0.000	0.000	0.000	2.627	526.017	0.031	1.445	0.037	0.056	0.000	0.135	6.793	0.000
0.670	0.000	0.334	0.000	0.000	0.000	2.130	218.445	0.027	0.644	0.000	0.167	0.000	0.000	5.749	0.000
1.053	0.000	0.555	0.000	0.000	0.000	2.823	775.580	0.027	1.717	0.000	0.062	0.000	0.000	9.926	0.000
1.024	0.013	0.088	0.000	0.000	0.000	1.117	1635.627	0.021	0.818	0.026	0.059	0.000	0.000	9.533	0.000
0.653	0.009	0.447	0.000	0.000	0.000	1.838	619.026	0.010	1.548	0.000	0.057	0.000	0.000	10.430	0.000
0.848	0.000	1.033	0.095	0.000	0.000	1.156	2871.350	0.021	0.867	0.039	0.029	0.000	0.000	8.865	0.000
0.726	0.009	0.820	0.000	0.000	0.000	1.139	1054.044	0.005	1.241	0.000	0.039	0.000	0.000	9.663	0.000
0.842	0.000	0.231	0.000	0.000	0.000	2.403	560.527	0.197	0.847	0.000	0.077	0.155	0.000	9.437	0.000
0.193	0.026	0.264	0.000	0.000	0.000	2.634	745.958	0.029	1.454	0.000	0.088	0.000	0.000	11.078	0.000
1.006	0.000	1.060	0.000	0.000	0.000	2.507	250.380	0.164	0.706	0.000	0.165	0.071	0.000	8.598	0.000
0.379	0.000	0.148	0.055	0.000	0.000	1.978	1278.871	0.030	1.878	0.000	0.066	0.000	0.000	8.238	0.000
1.298	0.000	2.223	0.000	0.000	0.000	2.136	109.695	0.326	0.210	0.000	0.149	0.299	0.000	15.219	0.000
0.341	0.000	0.560	0.069	0.000	0.000	1.379	869.071	0.053	0.519	0.000	0.250	0.126	0.000	9.746	0.000
1.563	0.000	0.559	0.000	0.000	0.000	1.423	1615.063	0.038	1.748	0.000	0.073	0.000	0.000	10.940	0.000
0.623	0.000	2.049	0.126	0.022	0.000	1.815	2338.289	0.007	3.887	0.026	0.114	0.000	0.000	9.652	0.000
0.879	0.010	0.669	0.000	0.000	0.000	1.572	1080.610	0.000	2.742	0.000	0.112	0.000	0.000	8.580	0.000
0.812	0.000	0.318	0.000	0.000	0.000	1.823	494.089	0.285	0.555	0.000	0.080	0.000	0.000	11.655	0.000
0.657	0.000	0.270	0.000	0.000	0.000	2.901	1094.732	0.103	4.460	0.000	0.100	0.000	0.097	12.185	0.000
1.097	0.000	0.451	0.000	0.000	0.000	2.544	491.240	0.337	0.229	0.000	0.109	0.000	0.000	10.618	0.000
0.758	0.000	0.559	0.059	0.000	0.000	1.227	730.999	0.065	0.436	0.000	0.036	0.000	0.000	11.436	0.000
1.054	0.018	0.314	0.046	0.000	0.000	2.418	111.215	0.225	0.484	0.000	0.147	0.000	0.000	17.811	0.000
1.195	0.000	0.524	0.048	0.000	0.000	1.623	615.510	0.165	1.007	0.000	0.022	0.088	0.000	16.480	0.000
0.393	0.000	0.299	0.000	0.000	0.000	2.222	192.316	0.049	2.081	0.018	0.113	0.000	0.064	7.654	0.000
0.936	0.000	0.933	0.000	0.000	0.000	1.954	258.863	0.057	0.367	0.000	0.091	0.079	0.000	8.268	0.000
0.999	0.018	0.622	0.046	0.000	0.000	3.089	95.989	1.283	0.810	0.019	0.118	0.335	0.000	8.659	0.000
1.079	0.000	0.835	0.000	0.000	0.000	1.278	137.457	0.126	0.209	0.000	0.029	0.087	0.000	7.886	0.000
0.860	0.000	0.558	0.000	0.000	0.000	1.308	899.149	0.091	0.319	0.000	0.083	0.042	0.000	10.107	0.000
0.570	0.000	0.781	0.048	0.000	0.000	3.831	165.732	0.069	0.136	0.000	0.022	0.438	0.000	11.834	0.000
1.098	0.000	0.439	0.000	0.000	0.000	2.149	236.610	0.276	0.789	0.000	0.098	0.169	0.000	12.490	0.000
0.849	0.000	0.727	0.000	0.000	0.000	1.802	560.150	0.124	0.237	0.000	0.081	0.000	0.000	10.152	0.000
1.113	0.000	0.610	0.000	0.000	0.000	2.314	182.296	0.012	1.347	0.023	0.059	0.051	0.082	10.502	0.000
1.361	0.000	1.554	1.378	0.081	0.000	1.919	396.342	0.506	0.326	0.000	0.139	0.000	0.000	7.507	0.000
0.574	0.000	0.984	0.000	0.000	0.000	1.876	713.576	0.353	1.083	0.000	0.066	0.000	0.000	10.487	0.000
1.003	0.000	0.611	0.000	0.000	0.000	1.862	386.566	0.044	1.526	0.023	0.119	0.000	0.000	10.131	0.000
0.944	0.045	0.761	0.000	0.020	0.000	2.159	695.430	0.180	1.920	0.023	0.085	0.103	0.000	12.260	0.000
0.363	0.000	0.398	0.000	0.000	0.000	1.293	2342.321	0.049	2.408	0.030	0.100	0.000	0.000	9.323	0.000
1.366	0.009	0.646	0.000	0.000	0.000	1.885	1536.531	0.247	2.215	0.000	0.050	0.043	0.000	9.186	0.000
0.364	0.000	0.499	0.000	0.000	0.000	2.007	897.535	0.102	0.873	0.000	0.019	0.000	0.000	9.880	0.000
0.627	0.000	0.382	0.000	0.000	0.000	2.256	112.281	0.140	1.852	0.000	0.138	0.000	0.000	8.332	0.000
1.587	0.000	1.003	0.000	0.000	0.000	1.771	429.153	0.204	1.377	0.050	0.056	0.000	0.000	9.238	0.000
0.726	0.000	0.153	0.000	0.000	0.000	0.987	1560.585	0.243	0.602	0.000	0.026	0.052	0.000	9.522	0.000
0.744	0.010	0.476	0.000	0.000	0.000	0.998	173.735	0.077	0.599	0.000	0.068	0.046	0.000	14.419	0.000
0.745	0.000	0.383	0.000	0.000	0.000	1.882	319.556	0.031	1.515	0.000	0.007	0.000	0.000	9.429	0.000
0.642	0.000	0.559	0.000	0.000	0.000	1.692	483.839	0.026	0.914	0.000	0.125	0.000	0.000	9.704	0.000
0.605	0.000	0.452	0.000	0.000	0.000	0.724	780.858	0.000	0.458	0.000	0.059	0.000	0.000	10.840	0.000
0.929	0.000	0.495	0.052	0.000	0.000	2.238	143.467	0.029	1.346	0.000	0.150	0.000	0.000	11.770	0.000
0.427	0.000	0.365	0.000	0.000	0.000	1.864	572.817	0.149	0.525	0.000	0.041	0.000	0.000	8.314	0.000
1.028	0.000	0.783	0.000	0.000	0.000	2.734	1160.212	0.229	1.305	0.000	0.087	0.000	0.168	11.261	0.000
1.085	0.000	0.446	0.000	0.000	0.000	1.618	490.200	0.036	0.523	0.000	0.025	0.100	0.000	15.502	0.000
0.461	0.000	0.474	0.000	0.000	0.000	1.268	1723.844	0.006	3.342	0.000	0.053	0.000	0.000	9.447	0.000
0.845	0.010	0.545	0.000	0.000	0.000	1.698	275.645	0.122	1.218	0.000	0.083	0.000	0.000	10.570	0.000
1.093	0.000	0.789	0.000	0.000	0.000	2.098	670.857	0.006	1.783	0.000	0.079	0.000	0.000	11.287	0.000
1.099	0.010	0.821	0.000	0.000	0.000	2.676	660.622	0.022	2.365	0.000	0.092	0.000	0.000	13.347	0.000
0.807	0.000	0.521	0.000	0.017	0.000	2.871	202.376	0.011	1.471	0.019	0.073	0.000	0.070	11.657	0.000
0.988	0.009	0.648	0.000	0.000	0.000	1.948	754.679	0.034	2.140	0.018	0.072	0.040	0.000	11.850	0.000
0.647	0.000	1.064	0.000	0.000	0.000	2.057	352.136	0.022	1.274	0.026	0.030	0.000	0.000	11.413	0.000
1.077	0.011	2.175	0.000	0.000	0.000	1.123	406.522	0.006	1.365	0.000	0.009	0.052	0.000	15.608	0.000
1.576	0.011	2.159	0.000	0.000	0.000	2.043	772.080	0.019	1.145	0.023	0.043	0.000	0.000	10.826	0.000
0.687	0.021	0.869	0.000	0.000	0.000	1.420	304.904	0.083	0.543	0.000	0.056	0.000	0.000	8.975	0.000
1.461	0.000	0.483	0.000	0.000	0.000	1.341	1099.735	0.079	3.056	0.000	0.054	0.047	0.000	11.089	0.000
0.493	0.000	1.125	0.000	0.000	0.000	2.348	971.714	0.073	9.042	0.000	0.025	0.000	0.000	9.576	0.000
0.695	0.000	0.595	0.044	0.000	0.000	1.851	1081.593	0.053	0.730	0.000	0.027	0.080	0.000	10.188	0.000
1.128	0.000	0.110	0.041	0.000	0.048	1.565	359.129	0.144	1.942	0.000	0.037	0.074	0.000	17.021	0.000
0.959	0.000	0.537	0.000	0.000	0.000	2.483	329.074	0.054	0.686	0.000	0.040	0.040	0.064	11.733	0.000
0.539	0.000	0.625	0.042	0.000	0.000	3.239	1623.609	0.028	0.983	0.000	0.076	0.191	0.000	9.957	0.000
0.653	0.000	0.716	0.000	0.000	0.000	1.601	1849.021	0.117	2.496	0.000	0.040	0.000	0.000	8.453	0.000
1.179	0.000	0.776	0.000	0.000	0.000	1.538	614.877	0.011	2.105	0.019	0.079	0.044	0.000	11.807	0.000
1.030	0.012	0.664	0.000	0.000	0.000	2.164	185.657	0.156	0.209	0.000	0.074	0.112	0.089	7.475	0.000
0.926	0.000	0.508	0.000	0.000	0.000	2.657	422.306	0.172	1.970	0.000	0.132	0.000	0.000	7.242	0.000
0.754	0.000	1.127	0.069	0.000	0.000	2.264	1052.465	0.176	1.158	0.000	0.052	0.000	0.000	11.497	0.000
1.232	0.000	1.055	0.156	0.000	0.000	1.723	634.964	0.223	1.151	0.032	0.000	0.000	0.113	11.221	0.000
1.000	0.000	1.371	0.000	0.000	0.000	1.379	174.734	0.244	1.312	0.000	0.076	0.000	0.000	9.095	0.000
0.828	0.000	0.895	0.044	0.000	0.000	1.042	111.451	0.088	0.219	0.036	0.033	0.241	0.000	13.359	0.000
0.625	0.000	0.979	0.000	0.000	0.000	1.669	344.108	0.070	0.404	0.000	0.020	0.041	0.000	9.592	0.000
0.717	0.000	0.874	0.000	0.000	0.000	1.246	561.951	0.053	1.467	0.000	0.085	0.000	0.000	11.257	0.000
0.665	0.000	0.683	0.000	0.000	0.000	1.584	403.213	0.028	2.484	0.000	0.051	0.000	0.000	9.670	0.000
0.844	0.000	0.231	0.000	0.000	0.000	1.232	155.773	0.019	0.663	0.000	0.077	0.052	0.000	10.724	0.000
0.758	0.000	0.485	0.000	0.000	0.000	2.350	194.522	0.147	0.941	0.021	0.077	0.093	0.000	9.008	0.000
0.993	0.000	0.612	0.000	0.000	0.000	1.276	367.875	0.055	1.644	0.000	0.030	0.000	0.000	8.127	0.000

ENSG00000127774	ENSG00000058729	ENSG00000142273	ENSG00000215126	ENSG00000100368	ENSG00000280398	ENSG00000263512	ENSG00000274987	ENSG00000170425	ENSG00000232860	ENSG00000069011	ENSG00000278107	ENSG00000173826	ENSG00000271763	ENSG00000125812	ENSG00000221962
5.375	3.059	0.000	0.126	1.678	0.000	0.000	0.553	2.028	0.300	1.882	1.436	0.112	0.000	5.939	0.000
6.731	4.161	2.545	0.186	2.874	0.000	0.000	0.217	3.687	0.087	5.082	1.221	0.030	0.000	5.974	0.000
7.648	3.963	0.000	0.301	1.124	0.000	0.000	0.386	3.759	0.127	3.158	4.635	0.040	0.000	5.530	0.000
4.277	3.301	0.000	0.231	0.390	0.000	0.000	1.021	3.402	0.075	0.592	2.064	0.025	0.048	6.141	0.000
4.818	3.839	0.015	0.164	2.067	0.000	0.000	0.608	1.480	0.112	3.590	3.061	0.028	0.000	3.793	0.000
4.778	5.404	0.012	0.438	1.992	0.000	0.000	1.731	2.384	0.347	0.489	1.942	0.031	0.042	7.006	0.099
4.096	5.199	0.239	0.481	4.519	0.000	0.210	0.613	2.947	0.124	2.165	2.329	0.005	0.047	6.097	0.000
4.044	2.903	0.000	0.122	2.426	0.000	0.000	0.382	2.125	0.092	0.259	2.502	0.095	0.000	5.494	0.000
8.278	3.672	0.033	0.395	1.171	0.000	0.000	1.808	5.142	0.083	0.051	2.541	0.067	0.000	4.741	0.000
7.871	3.653	0.189	0.359	7.188	0.000	0.000	0.642	2.665	0.088	0.383	1.275	0.000	0.000	4.295	0.000
5.563	3.831	0.058	0.364	3.452	0.000	0.000	1.073	1.396	0.081	0.204	3.039	0.083	0.041	4.957	0.000
5.338	3.568	0.012	0.256	3.603	0.000	0.190	0.588	9.774	0.270	2.349	1.589	0.027	0.000	7.399	0.050
6.604	3.539	0.012	0.247	1.251	0.000	0.000	1.146	4.343	0.110	1.100	2.242	0.023	0.000	7.000	0.052
4.794	3.637	0.023	0.215	1.992	0.000	0.000	0.599	5.679	0.309	5.568	2.222	0.070	0.041	7.859	0.000
8.289	2.733	0.000	0.102	1.226	0.000	0.000	0.421	2.627	0.024	1.071	3.068	0.039	0.000	5.430	0.000
4.130	3.220	0.058	0.147	15.982	0.000	0.000	0.536	2.209	0.179	0.392	1.880	0.039	0.000	7.191	0.049
6.937	4.594	0.013	0.218	0.711	0.000	0.000	0.436	3.751	0.181	0.374	1.445	0.075	0.000	5.541	0.000
11.079	3.444	0.000	0.295	0.437	0.000	0.000	0.475	2.315	0.059	1.911	1.072	0.030	0.000	3.216	0.000
6.341	4.025	0.073	0.167	2.237	0.000	0.000	1.314	1.117	0.220	0.485	2.415	0.014	0.043	4.701	0.000
4.918	3.827	0.039	0.138	1.124	0.000	0.000	0.808	1.851	0.242	0.501	2.233	0.005	0.000	6.755	0.000
11.195	3.850	0.014	0.182	0.427	0.000	0.000	0.337	4.673	0.084	1.037	1.116	0.047	0.000	4.109	0.000
4.658	3.960	0.029	0.280	0.946	0.000	0.000	0.542	2.906	0.233	1.912	1.845	0.128	0.101	6.730	0.000
6.441	5.441	0.000	0.199	1.087	0.000	0.000	0.454	1.363	0.091	1.060	3.719	0.042	0.000	4.227	0.291
4.919	4.364	0.012	0.342	1.783	0.000	0.000	0.161	2.775	0.155	8.945	1.374	0.018	0.000	4.570	0.000
5.667	4.422	0.026	0.249	3.682	0.000	0.000	0.287	4.013	0.092	1.217	1.993	0.213	0.000	6.301	0.055
6.484	3.747	0.000	0.361	2.320	0.000	0.000	1.006	2.193	0.327	0.945	2.072	0.088	0.049	7.001	0.000
5.070	1.627	0.031	0.245	2.967	0.000	0.000	1.138	1.278	0.203	3.010	2.050	0.035	0.000	5.444	0.000
9.657	3.649	0.055	0.382	0.751	0.000	0.000	0.223	4.908	0.089	0.197	4.568	0.072	0.000	3.983	0.000
7.410	2.740	0.041	0.406	0.989	0.000	0.000	1.071	1.888	0.064	1.758	4.220	0.061	0.096	6.550	0.000
7.199	3.526	0.014	0.553	0.631	0.000	0.000	0.557	1.873	0.070	3.030	5.941	0.010	0.000	4.671	0.000
13.324	3.078	0.045	0.229	0.719	0.000	0.000	0.611	4.233	0.142	0.274	2.671	0.059	0.000	5.617	0.000
8.193	3.534	0.035	0.181	0.615	0.000	0.000	1.121	0.877	0.231	0.228	2.545	0.027	0.000	7.551	0.000
8.969	2.961	0.014	0.378	0.989	0.000	0.000	0.671	2.253	0.077	0.210	2.924	0.196	0.000	4.633	0.000
7.557	3.169	0.053	0.386	1.559	0.000	0.000	1.113	2.163	0.105	0.155	3.781	0.025	0.047	4.557	0.000
3.559	5.015	0.000	0.140	1.316	0.000	0.000	0.834	2.561	0.261	0.226	3.390	0.031	0.000	5.988	0.000
5.227	4.238	0.000	0.396	2.668	0.000	0.000	0.928	2.399	0.213	0.120	4.027	0.057	0.000	8.254	0.000
6.259	4.773	0.012	0.652	1.795	0.000	0.000	0.361	0.846	0.728	0.740	2.216	0.100	0.043	15.086	0.000
5.134	4.340	0.000	0.149	3.956	0.000	0.191	1.114	1.540	0.265	1.222	1.920	0.027	0.000	7.500	0.000
7.131	5.013	0.000	0.427	1.220	0.000	0.000	0.704	3.488	0.195	0.207	2.796	0.081	0.000	6.706	0.060
4.097	4.709	0.000	0.217	0.607	0.000	0.000	1.010	4.009	0.229	1.587	0.762	0.028	0.053	6.692	0.000
3.589	3.546	0.044	0.167	1.923	0.000	0.000	4.100	0.954	0.256	0.147	1.677	0.072	0.000	6.774	0.000
5.090	4.154	0.188	0.333	1.447	0.000	0.000	0.713	4.821	0.316	3.502	2.092	0.146	0.089	7.017	0.000
5.800	4.235	0.000	0.324	0.918	0.000	0.000	0.598	3.514	0.257	0.384	1.893	0.036	0.000	7.415	0.000
4.152	4.330	0.000	0.309	1.203	0.000	0.000	0.578	2.752	0.287	0.856	2.071	0.013	0.000	4.095	0.000
5.072	5.627	0.000	0.199	1.317	0.000	0.000	2.462	2.535	0.523	2.454	1.290	0.028	0.045	9.652	0.000
4.215	4.642	0.012	0.394	3.191	0.000	0.000	0.667	1.917	0.470	0.287	2.053	0.032	0.087	7.538	0.000
3.613	3.313	0.014	0.175	0.171	0.000	0.214	0.404	0.880	0.120	0.067	1.678	0.010	0.144	5.978	0.000
6.328	3.821	0.000	0.356	2.046	0.000	0.000	0.520	4.231	0.243	0.080	2.694	0.056	0.048	6.073	0.000
7.097	5.147	0.024	0.294	0.716	0.000	0.000	0.644	2.161	0.144	9.225	2.144	0.192	0.000	5.836	0.000
7.231	3.544	0.034	0.225	6.606	0.000	0.000	0.517	3.360	0.073	1.473	1.481	0.029	0.000	4.846	0.094
6.299	4.289	0.011	0.255	1.293	0.000	0.000	0.673	4.552	0.063	0.695	1.793	0.211	0.000	4.991	0.000
4.806	4.695	0.230	0.208	2.260	0.000	0.000	0.823	2.387	0.300	1.451	2.091	0.059	0.000	4.995	0.000
6.035	6.578	0.037	0.383	2.550	0.000	0.000	0.670	2.278	0.190	2.294	1.198	0.028	0.000	5.629	0.000
6.925	4.435	0.044	0.204	1.423	0.000	0.000	0.622	4.188	0.270	4.040	1.294	0.008	0.039	7.095	0.000
9.080	6.812	0.000	0.620	0.279	0.000	0.000	0.654	13.922	0.450	0.307	1.733	0.113	0.000	4.648	0.000
2.580	8.249	0.000	0.422	0.347	0.000	0.000	0.852	1.815	0.098	0.918	1.129	0.059	0.000	9.231	0.000
6.974	5.109	0.026	0.176	1.438	0.000	0.000	0.318	2.134	0.140	1.100	1.999	0.122	0.000	5.476	0.000
6.236	7.121	0.025	0.280	1.029	0.000	0.000	1.501	2.709	0.178	1.040	1.524	0.028	0.000	6.624	0.000
4.540	4.702	0.024	0.325	1.563	0.000	0.193	0.464	4.704	0.239	0.253	3.424	0.018	0.000	7.624	0.000
6.693	4.622	0.057	0.187	1.231	0.000	0.180	0.588	0.120	0.149	3.614	1.290	0.034	0.000	7.182	0.000
5.761	3.429	0.013	0.478	0.474	0.000	0.000	0.214	1.867	0.338	1.090	2.449	0.044	0.000	11.198	0.000
12.102	4.445	0.000	0.359	0.469	0.000	0.000	0.454	3.428	0.039	0.667	2.084	0.008	0.000	3.981	0.000
5.965	4.441	0.011	0.737	0.454	0.000	0.000	0.886	2.532	0.203	2.784	1.964	0.147	0.000	6.145	0.000
3.857	3.610	0.117	0.385	3.136	0.000	0.553	0.760	1.611	0.321	0.499	3.678	0.166	0.000	6.418	0.000
7.904	5.934	0.013	0.117	0.394	0.000	0.000	0.670	1.666	0.212	0.076	4.135	0.020	0.000	9.063	0.000
3.671	3.308	0.033	0.145	0.586	0.000	0.000	0.816	2.513	0.055	1.698	1.801	0.050	0.000	6.355	0.000
9.035	3.341	0.000	0.223	0.615	0.000	0.000	0.435	2.312	0.107	6.184	1.791	0.017	0.000	4.584	0.000
11.263	3.731	0.031	0.042	0.265	0.000	0.000	0.415	3.821	0.057	5.703	1.070	0.046	0.000	4.685	0.000
8.550	3.836	0.000	0.130	0.731	0.000	0.000	0.551	0.654	0.085	0.110	2.408	0.015	0.000	6.382	0.000
17.165	4.826	0.000	0.095	0.790	0.000	0.000	0.218	1.092	0.112	5.513	0.994	0.012	0.000	4.999	0.000
8.669	5.353	0.012	0.211	0.547	0.000	0.185	0.381	1.745	0.131	2.609	2.615	0.039	0.000	4.337	0.000
6.601	2.890	0.024	0.101	1.650	0.000	0.000	1.026	2.502	0.121	3.253	1.847	0.098	0.000	4.182	0.000
5.104	2.805	0.129	0.155	1.367	0.000	0.000	0.552	5.598	0.140	0.371	2.685	0.028	0.000	4.042	0.000
8.498	4.224	0.000	0.273	0.754	0.000	0.333	1.318	2.431	0.069	5.508	5.405	0.079	0.000	7.153	0.000
4.002	3.675	0.354	0.122	1.821	0.000	0.000	0.393	3.140	0.263	1.659	2.778	0.012	0.000	5.242	0.067
4.384	4.072	0.000	0.298	0.215	0.000	0.000	1.948	6.271	0.082	0.532	2.510	0.022	0.137	8.336	0.081
7.799	3.589	0.000	0.330	1.135	0.000	0.643	0.294	3.627	0.089	1.903	0.804	0.015	0.000	3.799	0.000
3.508	4.786	0.163	0.309	0.257	0.000	0.000	1.104	1.460	0.190	1.061	1.754	0.000	0.000	6.537	0.000
8.738	3.973	0.000	0.413	0.464	0.000	0.000	0.603	6.590	0.104	2.075	1.381	0.013	0.000	6.594	0.000
5.564	5.233	0.017	0.223	0.947	0.038	0.538	3.375	5.032	0.048	1.157	2.526	0.013	0.000	5.093	0.000
5.776	4.305	0.094	0.344	0.582	0.000	0.000	0.763	2.747	0.051	0.935	1.305	0.023	0.000	4.043	0.000
5.736	4.803	0.012	0.227	0.712	0.000	0.000	1.762	1.411	0.103	0.320	2.113	0.018	0.000	7.965	0.000
3.466	3.929	0.000	0.356	0.818	0.000	0.230	0.316	2.017	0.048	1.328	3.881	0.033	0.000	4.592	0.000
5.512	3.672	0.016	0.153	3.308	0.000	0.000	0.849	2.650	0.277	0.681	1.109	0.031	0.000	4.960	0.000
3.108	3.404	0.014	0.153	1.225	0.000	0.217	0.747	2.972	0.199	1.101	2.632	0.098	0.000	5.989	0.000
3.242	3.809	0.132	0.211	2.899	0.000	0.000	0.476	3.936	0.055	0.776	3.860	0.027	0.000	6.110	0.000
5.878	4.942	0.000	0.386	0.969	0.000	0.545	1.123	1.743	0.392	5.144	3.408	0.013	0.000	6.181	0.000
7.141	5.424	0.000	0.228	1.227	0.000	0.000	0.455	2.138	0.140	0.016	4.362	0.054	0.000	5.719	0.050
5.083	4.124	0.000	0.193	0.446	0.025	0.000	0.713	8.502	0.271	0.660	2.420	0.066	0.039	5.908	0.000
4.469	3.515	0.021	0.078	0.701	0.000	0.000	1.042	3.883	0.149	1.343	3.335	0.064	0.000	5.211	0.000
2.316	4.590	0.023	1.004	0.252	0.000	0.180	1.638	1.375	0.101	0.594	4.727	0.098	0.040	5.564	0.000
5.530	3.247	0.106	0.202	0.353	0.000	0.000	0.479	4.659	0.604	0.169	4.595	0.062	0.000	5.303	0.098
4.800	3.723	0.000	0.150	0.835	0.000	0.179	0.493	0.513	0.127	5.042	2.538	0.000	0.000	4.691	0.047
3.643	5.358	0.000	1.080	0.316	0.000	0.000	0.808	0.516	0.439	0.867	1.636	0.089	0.000	8.920	0.000
2.003	3.791	0.000	0.241	0.729	0.000	0.000	1.432	0.689	0.273	0.031	2.598	0.052	0.000	8.645	0.144
9.268	3.989	0.133	0.194	0.509	0.000	0.000	0.361	4.376	0.379	0.108	3.372	0.040	0.047	8.478	0.056
8.679	3.163	0.000	0.377	0.410	0.000	0.220	0.530	4.326	0.300	0.194	1.466	0.016	0.099	7.572	0.000
8.176	4.081	0.000	0.674	0.665	0.000	0.000	2.161	1.796	0.239	5.091	4.292	0.021	0.201	6.084	1.188
4.140	5.642	0.000	1.105	1.694	0.000	0.000	0.391	2.741	0.218	0.453	1.455	0.081	0.127	8.034	0.000
4.563	4.473	0.028	0.253	1.205	0.000	0.000	0.455	6.696	0.183	0.409	3.992	0.157	0.000	5.357	0.000
4.147	4.237	0.042	0.273	1.100	0.000	0.000	0.567	1.905	0.273	1.240	3.490	0.084	0.049	6.783	0.000
6.386	4.192	0.018	0.312	1.120	0.000	0.000	0.888	3.479	0.034	0.147	2.058	0.007	0.000	3.806	0.000
5.071	4.080	0.780	0.479	1.358	0.000	0.000	1.282	2.231	0.072	4.485	5.445	0.040	0.084	6.192	0.099
5.067	3.314	0.000	0.317	2.035	0.000	0.000	0.413	1.405	0.590	1.966	1.026	0.011	0.000	6.943	0.000
1.937	4.196	0.017	0.279	0.138	0.000	0.000	1.090	4.973	0.171	0.071	2.259	0.033	0.000	10.057	0.000
4.022	4.093	0.031	0.167	0.686	0.000	0.000	0.747	2.823	0.157	0.454	3.794	0.046	0.000	5.901	0.000
5.207	4.246	0.014	0.296	1.332	0.000	0.000	0.950	0.989	0.170	0.794	3.775	0.063	0.000	7.909	0.000
11.388	4.550	0.000	0.189	0.361	0.000	0.000	0.405	4.557	0.139	4.469	1.441	0.019	0.000	7.070	0.000
7.335	5.330	0.000	0.128	0.538	0.000	0.000	0.443	2.435	0.174	2.321	3.271	0.022	0.000	8.024	0.000
11.472	3.110	0.088	0.153	0.753	0.000	0.000	0.397	11.163	0.089	13.886	2.049	0.143	0.000	3.733	0.000
8.156	4.694	0.014	0.090	0.375	0.000	0.000	0.262	1.212	0.013	0.012	2.490	0.010	0.000	6.920	0.000
5.103	5.732	0.000	0.138	0.540	0.000	0.000	0.878	3.742	0.151	3.347	2.407	0.024	0.000	8.433	0.000
3.268	3.370	0.013	0.234	1.094	0.000	0.211	0.327	1.714	0.187	0.054	1.189	0.015	0.000	5.133	0.000
4.000	3.667	0.029	0.274	2.406	0.000	0.000	0.622	3.936	0.134	1.224	3.242	0.021	0.000	5.846	0.000
6.091	4.343	0.585	0.281	0.971	0.000	0.000	0.332	4.102	0.413	15.269	2.090	0.071	0.000	6.529	0.000
6.468	3.085	0.058	0.195	1.994	0.000	0.000	0.549	2.898	0.162	1.618	1.908	0.054	0.000	4.168	0.000
5.503	3.835	0.000	0.191	0.571	0.000	0.000	0.271	2.623	0.239	0.533	1.948	0.028	0.000	6.896	0.000
4.979	4.896	0.029	0.272	0.793	0.000	0.000	0.744	1.277	0.296	0.467	2.139	0.049	0.000	5.102	0.000
5.683	5.930	0.013	0.202	1.032	0.028	0.000	0.408	6.403	0.088	1.757	2.733	0.028	0.000	5.198	0.000
5.185	5.018	0.000	0.157	0.433	0.000	0.000	0.614	3.908	0.161	5.459	2.086	0.009	0.042	5.360	0.000
2.412	3.572	0.000	0.252	1.463	0.000	0.000	0.438	1.614	0.282	3.857	1.394	0.036	0.000	7.939	0.045
3.864	4.949	0.016	0.417	0.512	0.000	0.000	0.440	4.137	0.439	4.940	1.137	0.012	0.000	6.058	0.000
5.049	4.051	0.014	0.175	2.029	0.000	0.000	0.578	3.519	0.053	0.595	1.149	0.059	0.000	6.122	0.000
10.839	4.881	0.000	0.131	0.557	0.000	0.000	0.728	1.284	0.211	7.008	1.711	0.021	0.050	6.833	0.000
3.645	4.034	0.000	0.123	0.519	0.000	0.209	0.539	2.206	0.235	0.125	3.641	0.035	0.000	6.552	0.000
3.917	2.582	0.025	0.205	1.863	0.000	0.000	0.480	4.348	0.130	0.443	1.396	0.261	0.089	4.886	0.000
5.244	3.523	0.000	0.394	1.938	0.000	0.000	0.559	2.906	0.135	1.204	2.331	0.031	0.000	4.286	0.000
7.379	3.947	0.152	0.420	1.420	0.000	0.172	0.709	3.074	0.463	0.764	3.316	0.073	0.077	5.996	0.000
5.827	4.739	0.030	0.207	1.135	0.000	0.000	0.356	2.613	0.189	7.889	2.150	0.023	0.000	5.208	0.000
7.719	6.483	0.415	0.540	0.683	0.000	0.000	0.563	4.465	0.275	3.927	1.796	0.070	0.000	9.979	0.091
4.975	4.064	0.021	0.190	1.901	0.000	0.000	0.959	2.826	0.252	6.238	2.213	0.012	0.000	7.910	0.000
7.666	4.126	0.065	0.060	1.489	0.000	0.000	0.178	5.027	0.046	0.236	1.235	0.049	0.000	4.799	0.000
7.409	5.392	0.000	0.270	0.904	0.000	0.000	0.578	1.721	0.210	0.176	2.423	0.004	0.000	6.439	0.000
7.489	1.814	0.000	0.374	0.638	0.000	0.000	0.825	1.872	0.177	0.273	3.481	0.103	0.054	7.085	0.000
8.837	3.004	0.015	0.141	0.450	0.000	0.000	0.462	2.022	0.065	4.895	2.044	0.006	0.000	6.685	0.065
7.059	3.611	0.052	0.308	3.116	0.000	0.000	0.466	3.224	0.313	1.322	3.196	0.039	0.000	5.604	0.072
8.404	3.335	0.270	0.276	2.006	0.000	0.000	0.890	5.509	0.216	3.134	1.873	0.087	0.068	7.059	0.161
3.702	3.586	0.000	0.429	0.485	0.000	0.495	1.063	1.795	0.117	0.818	2.070	0.082	0.000	6.652	0.000
4.419	5.097	0.000	0.274	0.469	0.000	0.000	0.889	2.529	0.199	9.688	3.738	0.106	0.039	9.750	0.000
3.657	3.777	0.056	0.298	0.584	0.000	0.000	0.334	1.241	0.162	0.045	3.501	0.092	0.159	6.342	0.000
5.623	3.637	0.020	0.146	1.061	0.000	0.000	0.434	7.758	0.131	0.324	3.555	0.037	0.142	5.454	0.084
6.096	3.375	0.365	0.106	0.892	0.000	0.000	0.622	2.373	0.117	3.167	2.807	0.023	0.000	3.496	0.000
8.314	3.470	0.028	0.174	0.860	0.000	0.000	0.536	1.581	0.481	4.499	1.977	0.090	0.050	11.475	0.000
7.004	4.574	0.000	0.287	0.653	0.000	0.000	1.101	1.585	0.320	5.925	2.323	0.048	0.045	6.098	0.000
8.128	4.537	0.025	0.093	0.436	0.000	0.197	0.203	3.167	0.134	3.605	3.488	0.023	0.000	5.332	0.052

ENSG00000273674	ENSG00000226520	ENSG00000265565	ENSG00000199335	ENSG00000265995	ENSG00000109911	ENSG00000238457	ENSG00000229332	ENSG00000185670	ENSG00000235201	ENSG00000205636	ENSG00000199075	ENSG00000136111	ENSG00000153707	ENSG00000251501	ENSG00000101000
0.074	0.000	0.000	0.000	0.000	5.209	0.000	0.044	1.249	0.000	0.000	0.000	2.390	6.095	0.000	5.343
0.047	0.000	0.000	0.000	0.000	5.500	0.000	0.000	0.723	0.000	0.000	0.000	2.484	2.370	0.000	31.688
0.077	0.064	0.000	0.000	0.000	7.284	0.000	0.033	1.936	0.000	0.000	0.000	2.447	4.200	0.000	4.849
0.127	0.000	0.000	0.198	0.000	13.568	0.000	0.038	1.806	0.000	0.000	0.000	4.146	9.163	0.000	4.974
0.027	0.000	0.000	0.000	0.000	9.838	0.000	0.000	1.438	0.000	0.000	0.000	2.031	1.261	0.000	15.845
0.098	0.064	0.296	0.174	0.000	6.809	0.000	0.000	2.384	0.000	0.000	0.000	5.023	8.065	0.000	3.265
0.047	0.072	0.000	0.000	0.000	6.691	0.000	0.000	1.744	0.000	0.000	0.000	5.309	2.746	0.000	22.061
0.166	0.000	0.353	0.000	0.000	8.149	0.000	0.000	1.374	0.000	0.000	0.000	4.194	7.379	0.000	10.597
0.308	0.087	0.000	0.240	0.000	9.428	0.000	0.000	1.687	0.000	0.000	0.000	3.474	6.436	0.015	13.635
0.084	0.127	0.000	0.000	0.000	6.364	0.000	0.000	1.562	0.000	0.000	0.000	4.972	1.698	0.000	17.738
0.069	0.000	0.000	0.343	0.000	7.049	0.000	0.000	1.486	0.000	0.000	0.477	3.463	2.645	0.000	21.124
0.057	0.000	0.000	0.355	0.000	10.131	0.000	0.000	1.992	0.000	0.000	0.000	2.811	7.956	0.000	8.755
0.044	0.033	0.000	0.000	0.000	12.867	0.000	0.279	1.968	0.000	0.000	0.000	4.103	5.864	0.011	9.339
0.172	0.000	0.000	0.000	0.000	11.909	0.000	0.065	1.917	0.000	0.000	0.000	3.322	6.522	0.010	5.782
0.000	0.000	0.000	0.000	0.000	9.355	0.000	0.000	2.075	0.000	0.000	0.000	2.860	3.004	0.000	16.612
0.048	0.031	0.000	0.172	0.000	5.973	0.000	0.033	1.853	0.000	0.000	0.000	6.135	4.044	0.000	9.002
0.087	0.000	0.000	0.198	0.000	10.618	0.000	0.000	1.391	0.000	0.000	0.000	2.362	8.671	0.000	5.450
0.339	0.000	0.000	0.000	0.000	11.948	0.000	0.089	2.244	0.000	0.000	0.000	1.431	1.251	0.000	19.277
0.129	0.033	0.000	0.715	0.000	7.610	0.000	0.102	1.428	0.000	0.000	0.248	3.981	2.448	0.000	10.668
0.031	0.035	0.000	0.000	0.000	9.572	0.000	0.000	1.991	0.000	0.000	0.000	2.932	1.303	0.000	6.248
0.278	0.000	0.000	0.204	0.000	13.770	0.000	0.000	2.155	0.000	0.000	0.000	1.205	4.375	0.012	16.346
0.059	0.077	0.000	0.000	0.000	6.072	0.000	0.000	2.355	0.000	0.000	0.585	3.976	11.370	0.013	2.440
0.082	0.000	0.000	0.000	0.000	8.184	0.361	0.039	1.282	0.000	0.000	0.000	2.338	3.787	0.012	10.892
0.035	0.000	0.000	0.000	0.000	7.317	0.000	0.067	1.169	0.000	0.000	0.243	1.977	3.123	0.000	22.618
0.078	0.036	0.000	0.000	0.000	7.771	0.000	0.000	1.644	0.000	0.000	0.000	3.263	1.852	0.000	11.322
0.041	0.000	0.000	0.405	0.000	7.552	0.000	0.000	1.884	0.000	0.000	0.282	3.155	4.903	0.000	8.605
0.073	0.042	0.000	0.458	0.000	9.448	0.000	0.044	1.648	0.000	0.000	0.000	3.344	1.665	0.000	14.970
0.065	0.000	0.000	0.000	0.000	7.647	0.000	0.000	1.909	0.000	0.000	0.000	2.607	3.956	0.024	7.719
0.242	0.000	0.000	0.000	0.000	11.880	0.000	0.038	2.453	0.000	0.000	0.279	3.656	2.233	0.000	9.347
0.251	0.000	0.000	0.202	0.000	10.116	0.000	0.038	2.086	0.000	0.000	0.000	2.287	1.811	0.000	9.385
0.193	0.030	0.000	0.000	0.000	12.185	0.000	0.000	1.886	0.000	0.000	0.000	1.980	3.002	0.000	11.877
0.217	0.000	0.000	0.000	0.000	8.275	0.000	0.000	2.155	0.000	0.000	0.000	1.316	1.791	0.000	4.162
0.187	0.000	0.000	0.000	0.000	10.498	0.000	0.039	2.287	0.000	0.000	0.000	2.394	5.809	0.000	9.220
0.078	0.036	0.000	0.977	0.000	7.159	0.000	0.037	1.861	0.000	0.000	0.000	1.325	1.966	0.000	13.584
0.066	0.000	0.000	0.000	0.000	9.948	0.000	0.000	2.360	0.000	0.000	0.000	2.427	3.797	0.000	8.024
0.045	0.000	0.000	0.748	0.000	13.040	0.000	0.000	1.941	0.000	0.000	0.000	3.820	3.525	0.034	11.022
0.200	0.033	0.000	0.714	0.000	8.452	0.000	0.204	2.704	0.000	0.000	0.496	4.240	23.419	0.000	3.060
0.093	0.065	0.000	0.178	0.000	10.943	0.000	0.068	2.125	0.000	0.000	0.000	5.939	4.321	0.000	5.632
0.102	0.000	0.000	0.000	0.000	9.824	0.000	0.000	2.229	0.000	0.000	0.000	4.423	10.485	0.026	10.061
0.106	0.040	0.373	0.000	0.000	11.654	0.000	0.000	2.233	0.000	0.000	0.000	3.312	6.701	0.000	4.151
0.105	0.000	0.000	0.000	0.000	9.846	0.000	0.000	1.523	0.000	0.000	0.000	3.808	3.971	0.000	8.972
0.015	0.000	0.000	0.185	0.000	9.631	0.000	0.000	1.612	0.000	0.000	0.257	2.186	1.708	0.000	19.903
0.073	0.074	0.345	0.000	0.000	13.138	0.000	0.000	1.501	0.000	0.000	0.000	3.670	2.758	0.000	10.313
0.077	0.000	0.296	0.175	0.000	13.439	0.000	0.033	2.058	0.000	0.000	0.000	4.591	6.150	0.000	8.574
0.253	0.102	0.000	0.558	0.000	13.908	0.000	0.035	2.294	0.000	0.000	0.258	5.102	2.275	0.000	9.167
0.182	0.000	0.000	0.000	0.000	12.200	0.000	0.000	2.950	0.000	0.000	0.000	3.279	2.490	0.000	8.393
0.048	0.109	0.000	0.000	0.000	2.841	0.000	0.000	1.577	0.000	0.000	0.000	7.781	14.677	0.000	18.993
0.113	0.037	0.000	0.000	0.000	6.823	0.000	0.038	1.776	0.000	0.000	0.000	4.057	7.805	0.000	7.268
0.021	0.032	0.000	0.000	0.000	10.616	0.000	0.000	2.330	0.000	0.000	0.000	2.113	2.583	0.000	6.205
0.040	0.000	0.000	0.000	0.000	8.523	0.000	0.032	1.121	0.000	0.000	0.000	3.878	3.568	0.000	18.550
0.127	0.182	0.000	0.499	0.000	7.986	0.000	0.000	1.261	0.000	0.000	0.000	3.023	5.457	0.000	7.914
0.057	0.033	0.000	0.000	0.000	9.458	0.000	0.034	0.898	0.000	0.000	0.000	4.369	1.753	0.000	28.923
0.080	0.033	0.619	0.000	0.000	14.833	0.000	0.069	1.479	0.000	0.000	0.000	2.873	4.661	0.000	12.802
0.052	0.000	0.000	0.161	0.000	11.766	0.000	0.061	2.394	0.000	0.000	0.000	2.298	0.903	0.000	8.629
0.190	0.000	0.000	0.297	0.000	13.258	0.000	0.113	1.242	0.000	0.000	0.000	1.163	5.508	0.000	4.002
0.217	0.000	0.000	0.000	0.000	9.761	0.000	0.000	1.901	0.000	0.000	0.000	2.309	4.102	0.047	3.986
0.131	0.000	0.000	0.192	0.000	10.732	0.000	0.000	2.228	0.000	0.000	0.000	1.987	2.238	0.000	6.707
0.131	0.099	0.308	0.181	0.000	9.922	0.000	0.035	1.119	0.000	0.000	0.000	3.429	2.025	0.000	7.345
0.036	0.000	0.306	0.000	0.000	10.258	0.000	0.034	1.377	0.000	0.000	0.000	4.575	4.571	0.000	6.325
0.094	0.000	0.000	0.336	0.000	14.623	0.000	0.160	1.822	0.000	0.000	0.000	2.825	2.793	0.000	18.424
0.101	0.000	0.991	0.000	0.000	14.342	0.000	0.222	2.330	0.000	0.000	0.000	3.277	5.416	0.000	5.138
0.148	0.000	0.000	0.617	0.000	15.676	0.000	0.117	1.291	0.000	0.000	0.000	2.077	1.524	0.000	4.407
0.058	0.000	0.000	0.000	0.000	10.608	0.000	0.000	1.837	0.000	0.000	0.000	2.163	10.453	0.000	2.957
0.055	0.000	0.877	0.000	0.000	6.269	0.000	0.033	1.156	0.000	0.000	0.000	2.175	1.266	0.000	14.897
0.077	0.000	1.956	0.000	0.000	14.288	0.000	0.256	2.055	0.000	0.053	0.000	1.844	6.693	0.000	8.089
0.059	0.000	0.000	0.493	0.000	8.619	0.000	0.094	1.797	0.000	0.000	0.000	2.148	6.862	0.000	4.834
0.163	0.000	0.000	0.000	0.000	10.763	0.000	0.000	1.891	0.000	0.000	0.000	2.457	5.275	0.020	7.677
0.136	0.000	0.000	0.225	0.000	15.667	0.000	0.000	2.241	0.000	0.000	0.000	1.058	0.900	0.014	6.121
0.048	0.000	0.000	0.000	0.000	10.306	0.000	0.000	1.954	0.000	0.000	0.000	3.252	1.501	0.000	5.235
0.123	0.043	0.000	0.000	0.000	14.215	0.000	0.000	1.286	0.000	0.000	0.000	2.675	0.897	0.000	11.521
0.028	0.000	0.000	0.173	0.000	13.198	0.000	0.000	1.423	0.000	0.000	0.000	1.373	5.168	0.000	6.137
0.098	0.000	0.000	0.000	0.000	10.210	0.000	0.000	1.427	0.000	0.000	0.000	1.699	2.092	0.000	21.072
0.038	0.000	0.000	0.000	0.000	8.616	0.000	0.241	1.415	0.000	0.000	0.000	2.076	1.173	0.000	10.368
0.075	0.000	0.000	0.312	0.000	12.546	0.000	0.000	1.932	0.000	0.000	0.000	1.577	3.478	0.000	10.265
0.029	0.000	0.000	0.475	0.000	7.152	0.000	0.000	1.960	0.000	0.000	0.000	2.448	1.653	0.000	8.829
0.138	0.000	0.486	0.000	0.000	17.056	0.000	0.000	1.803	0.000	0.000	0.000	2.283	8.970	0.000	6.752
0.088	0.073	0.340	0.200	0.000	11.149	0.000	0.114	1.663	0.000	0.000	0.000	3.368	1.734	0.000	19.179
0.000	0.219	0.000	1.201	0.000	8.047	0.000	0.000	2.110	0.000	0.000	0.000	6.676	2.815	0.000	32.306
0.263	0.000	0.000	0.000	0.000	17.524	0.000	0.192	2.176	0.000	0.000	0.000	1.483	1.920	0.000	10.230
0.111	0.000	0.000	0.000	0.000	8.780	0.000	0.096	1.165	0.000	0.000	0.000	2.324	1.920	0.000	13.374
0.305	0.042	0.000	0.000	0.000	11.331	0.000	0.000	1.592	0.000	0.000	0.000	2.420	2.726	0.000	13.473
0.101	0.000	0.000	0.362	0.000	14.578	0.000	0.034	1.387	0.000	0.000	0.000	2.226	2.373	0.000	10.850
0.043	0.039	0.000	0.644	0.000	7.786	0.000	0.123	1.537	0.000	0.000	0.000	3.613	4.507	0.000	6.255
0.068	0.000	0.000	0.243	0.000	7.031	0.000	0.046	1.507	0.000	0.000	0.000	4.656	3.023	0.000	8.334
0.090	0.111	0.000	0.203	0.000	7.744	0.000	0.039	1.758	0.000	0.056	0.000	2.170	13.098	0.000	2.885
0.052	0.079	0.000	0.000	0.000	6.262	0.000	0.000	0.917	0.000	0.000	0.000	1.699	0.802	0.000	48.955
0.224	0.000	0.000	0.170	0.000	6.716	0.000	0.000	2.716	0.000	0.000	0.000	4.090	11.631	0.010	1.481
0.064	0.032	0.301	0.000	0.066	8.047	0.000	0.000	1.708	0.000	0.000	0.000	5.733	4.197	0.000	10.746
0.065	0.029	0.275	0.000	0.000	10.218	0.000	0.062	1.524	0.000	0.000	0.000	3.302	4.118	0.000	5.068
0.227	0.000	0.000	0.315	0.000	11.281	0.000	0.000	1.801	0.000	0.000	0.438	3.699	7.016	0.000	6.798
0.121	0.061	0.000	0.672	0.000	7.960	0.000	0.096	1.424	0.000	0.000	0.234	3.477	9.890	0.000	4.563
0.090	0.032	0.000	0.174	0.000	6.737	0.000	0.165	2.074	0.000	0.000	0.000	0.792	1.128	0.000	5.001
0.040	0.000	0.000	0.000	0.000	10.546	0.000	0.000	1.820	0.000	0.000	0.000	3.507	1.324	0.000	6.075
0.225	0.000	0.298	0.000	0.000	6.541	0.000	0.033	0.971	0.000	0.000	0.000	5.992	5.782	0.000	2.078
0.306	0.062	0.000	0.000	0.000	7.277	0.000	0.064	1.267	0.000	0.000	0.235	4.325	3.912	0.031	5.539
0.102	0.036	0.000	0.196	0.000	11.038	0.000	0.037	2.725	0.000	0.000	0.000	2.394	7.308	0.000	5.662
0.074	0.038	0.000	0.000	0.000	5.996	0.000	0.000	1.290	0.000	0.000	0.000	1.644	4.120	0.012	6.030
0.034	0.306	0.000	0.000	0.000	7.812	4.418	0.000	2.495	0.000	0.000	0.000	2.874	10.019	0.000	3.532
0.149	0.000	0.000	0.531	0.000	12.427	0.000	0.101	2.360	0.000	0.000	0.000	4.321	4.669	0.000	11.599
0.083	0.000	0.350	0.000	0.000	11.070	0.000	0.000	1.668	0.000	0.000	0.287	1.896	5.571	0.000	5.108
0.066	0.037	0.000	0.000	0.000	8.434	0.000	0.117	0.933	0.000	0.000	0.000	2.781	3.968	0.000	6.053
0.215	0.098	0.912	0.268	0.000	11.558	0.000	0.204	1.664	0.000	0.000	0.000	2.869	3.963	0.000	18.472
0.105	0.032	0.000	0.000	0.000	7.080	0.000	0.000	1.650	0.219	0.000	0.000	2.192	1.968	0.000	15.143
0.072	0.000	0.000	0.450	0.000	10.948	0.000	0.043	1.660	0.000	0.000	0.000	3.696	6.444	0.000	10.456
0.103	0.000	0.438	0.773	0.000	10.080	0.000	0.000	1.821	0.215	0.000	0.000	4.362	4.714	0.000	12.337
0.054	0.000	0.000	0.452	0.000	8.625	0.000	0.000	1.357	0.000	0.000	0.000	2.969	6.463	0.000	8.103
0.008	0.038	0.000	0.000	0.000	9.412	0.000	0.000	1.844	0.000	0.000	0.000	1.811	3.173	0.000	23.847
0.103	0.000	0.000	0.000	0.000	8.725	0.000	0.070	3.537	0.000	0.000	0.000	1.905	2.030	0.011	4.603
0.117	0.031	0.000	0.000	0.000	12.485	0.000	0.000	1.723	0.000	0.000	0.000	1.578	3.543	0.000	4.048
0.087	0.000	0.000	0.216	0.000	8.722	0.000	0.000	1.421	0.000	0.000	0.000	1.523	0.751	0.000	15.282
0.033	0.037	0.346	0.407	0.000	31.327	0.000	0.000	1.392	0.000	0.000	0.000	1.508	2.500	0.000	12.391
0.054	0.035	0.000	0.000	0.000	12.158	0.000	0.073	1.367	0.000	0.000	0.000	1.983	5.135	0.000	8.157
0.111	0.000	0.000	0.000	0.000	14.513	0.000	0.038	1.880	0.000	0.000	0.000	3.793	7.363	0.000	6.360
0.017	0.000	0.000	0.000	0.000	8.052	0.000	0.000	1.946	0.000	0.000	0.000	2.908	7.101	0.000	7.718
0.105	0.000	0.000	0.201	0.000	8.260	0.000	0.000	1.457	0.000	0.000	0.000	1.204	1.543	0.000	6.137
0.009	0.000	0.000	0.000	0.000	8.421	0.000	0.081	1.322	0.000	0.000	0.000	2.707	0.595	0.000	17.688
0.059	0.034	0.000	0.368	0.000	11.025	0.000	0.000	2.275	0.000	0.000	0.000	3.101	12.501	0.000	4.439
0.213	0.000	0.000	0.000	0.000	15.066	0.000	0.000	1.062	0.000	0.000	0.000	7.780	5.856	0.000	20.777
0.156	0.000	0.000	0.000	0.000	14.231	0.000	0.000	1.465	0.000	0.000	0.000	3.237	1.894	0.000	12.234
0.042	0.000	0.000	0.176	0.000	14.553	0.000	0.000	1.399	0.000	0.000	0.000	2.791	3.802	0.021	3.418
0.064	0.000	0.270	0.159	0.000	9.001	0.000	0.030	1.469	0.000	0.000	0.000	2.882	5.892	0.000	6.386
0.134	0.000	0.000	0.239	0.000	5.622	0.000	0.046	1.538	0.000	0.000	0.000	1.404	3.416	0.000	5.378
0.109	0.038	0.000	0.000	0.000	4.066	0.000	0.000	0.838	0.000	0.000	0.000	5.245	1.528	0.000	27.123
0.401	0.000	0.000	0.000	0.000	18.882	0.000	0.040	1.318	0.000	0.000	0.000	2.407	0.692	0.000	16.434
0.047	0.000	0.000	0.000	0.000	11.586	0.000	0.074	2.574	0.000	0.000	0.000	4.352	3.449	0.012	5.308
0.127	0.000	0.000	0.186	0.000	6.707	0.000	0.035	1.382	0.000	0.000	0.259	3.707	7.631	0.000	9.960
0.268	0.000	0.000	1.216	0.000	9.321	0.000	0.000	2.234	0.000	0.000	0.000	5.177	3.150	0.000	19.347
0.122	0.000	0.273	0.161	0.000	9.120	0.000	0.000	2.630	0.000	0.000	0.670	2.735	4.926	0.000	6.582
0.066	0.000	0.253	0.000	0.000	9.342	0.000	0.000	1.886	0.062	0.000	0.000	1.236	1.286	0.000	12.753
0.091	0.000	0.000	0.000	0.000	10.514	0.000	0.061	2.515	0.000	0.000	0.000	2.315	7.078	0.000	7.361
0.105	0.028	0.000	0.000	0.000	11.350	0.000	0.000	1.991	0.000	0.042	0.000	3.280	3.991	0.000	6.139
0.039	0.000	0.000	0.000	0.000	8.832	0.000	0.092	1.266	0.000	0.000	0.000	2.283	1.414	0.000	12.697
0.168	0.000	0.000	0.000	0.000	14.733	0.000	0.000	0.798	0.000	0.048	0.000	4.848	2.796	0.011	9.687
0.135	0.041	0.381	0.000	0.000	4.207	0.000	0.000	3.204	0.000	0.000	0.000	1.543	5.592	0.000	3.222
0.082	0.000	0.000	0.914	0.000	11.446	0.000	0.000	1.811	0.000	0.000	0.317	2.401	0.626	0.000	6.692
0.153	0.046	0.000	0.254	0.000	8.146	0.000	0.000	2.035	0.000	0.000	0.352	2.699	4.364	0.000	7.470
0.057	0.052	0.000	0.285	0.000	7.597	0.000	0.000	1.912	0.000	0.000	0.000	3.471	3.585	0.000	8.752
0.083	0.000	0.000	0.463	0.000	8.311	0.000	0.000	2.085	0.000	0.000	0.000	3.454	6.728	0.000	3.599
0.097	0.000	0.000	0.000	0.000	14.768	0.000	0.031	1.448	0.000	0.000	0.000	1.614	3.386	0.000	3.736
0.060	0.000	0.000	0.496	0.000	17.088	0.000	0.031	1.337	0.000	0.000	0.000	2.840	9.467	0.000	6.324
0.059	0.000	0.000	0.295	0.000	11.849	0.000	0.000	1.631	0.000	0.000	0.000	2.318	6.604	0.000	8.594
0.062	0.056	0.000	0.000	0.000	10.067	0.000	0.029	1.066	0.000	0.000	0.000	1.828	1.373	0.000	15.998
0.033	0.038	0.000	0.000	0.000	10.321	0.000	0.000	2.502	0.000	0.000	0.289	1.258	2.814	0.000	20.625
0.083	0.034	0.318	0.748	0.000	13.209	0.000	0.000	1.405	0.000	0.000	0.000	1.864	4.265	0.000	4.872
0.118	0.000	0.000	0.000	0.000	13.764	0.000	0.000	1.297	0.000	0.000	0.000	1.948	4.164	0.000	5.409

ENSG00000280515	ENSG00000241479	ENSG00000232301	ENSG00000259088	ENSG00000261476	ENSG00000250037	ENSG00000227312	ENSG00000276449	ENSG00000226403	ENSG00000261291	ENSG00000054967	ENSG00000229809	ENSG00000214244	ENSG00000075290	ENSG00000270317	ENSG00000259782
0.024	0.000	0.000	0.718	0.058	0.121	0.000	0.365	4.785	0.000	2.819	3.717	0.139	0.000	0.000	0.030
0.000	0.000	0.000	0.235	0.025	0.000	0.000	0.171	0.140	0.000	3.435	3.926	0.094	0.010	0.000	0.050
0.000	0.000	0.000	0.962	0.000	0.061	0.000	0.547	1.540	0.037	3.389	5.967	0.315	0.053	0.000	0.022
0.000	0.079	0.000	0.284	0.025	0.000	0.000	1.277	1.179	0.042	1.890	5.334	0.095	0.020	0.000	0.025
0.011	0.000	0.000	0.474	0.000	0.000	0.068	0.305	0.367	0.000	2.020	5.442	0.132	0.034	0.000	0.113
0.000	0.035	0.049	1.374	0.263	0.030	0.000	1.139	0.870	0.110	3.844	5.621	0.146	0.168	0.075	0.111
0.020	0.000	0.000	0.938	0.123	0.000	0.000	0.246	0.047	0.124	4.202	4.606	0.141	0.060	0.000	0.201
0.000	0.000	0.000	0.843	0.026	0.000	0.000	0.227	2.518	0.087	2.709	4.953	0.050	0.021	0.000	0.000
0.012	0.048	0.000	0.688	0.030	0.126	0.000	0.873	3.252	0.000	2.172	5.435	0.029	0.024	0.000	0.061
0.000	0.000	0.000	0.334	0.000	0.000	0.000	0.135	0.996	0.000	4.125	6.995	0.335	0.035	0.000	0.179
0.000	0.069	0.000	0.369	0.000	0.030	0.053	0.304	1.958	0.072	3.215	5.666	0.062	0.017	0.000	0.022
0.009	0.036	0.000	0.848	0.156	0.031	0.000	0.686	1.858	0.037	3.986	4.539	0.149	0.036	0.076	0.136
0.028	0.000	0.000	0.875	0.023	0.000	0.056	0.879	0.261	0.077	1.951	4.268	0.044	0.000	0.000	0.047
0.027	0.171	0.000	0.327	0.151	0.060	0.000	1.159	0.489	0.036	4.493	5.672	0.144	0.061	0.000	0.110
0.000	0.000	0.000	0.182	0.000	0.000	0.235	0.344	0.363	0.000	1.861	4.677	0.366	0.039	0.000	0.195
0.018	0.034	0.024	0.901	0.043	0.000	0.000	0.171	0.204	0.072	4.788	4.063	0.041	0.026	0.073	0.000
0.000	0.000	0.028	0.756	0.025	0.069	0.000	0.586	1.600	0.083	2.234	3.957	0.071	0.040	0.000	0.051
0.000	0.000	0.000	0.280	0.000	0.041	0.000	0.567	2.346	0.000	1.491	10.599	0.000	0.024	0.101	0.090
0.000	0.036	0.025	0.726	0.045	0.031	0.110	0.270	4.207	0.037	2.998	4.941	0.064	0.000	0.000	0.114
0.010	0.000	0.000	0.319	0.048	0.000	0.000	0.689	0.273	0.080	3.467	3.611	0.000	0.000	0.000	0.098
0.000	0.000	0.029	0.486	0.000	0.000	0.063	0.302	4.988	0.000	1.669	8.161	0.122	0.010	0.000	0.078
0.000	0.000	0.000	0.704	0.053	0.037	0.000	1.119	0.701	0.044	2.452	3.995	0.177	0.053	0.090	0.054
0.000	0.000	0.058	1.278	0.103	0.000	0.000	0.629	1.565	0.173	2.061	6.139	0.123	0.010	0.000	0.237
0.000	0.000	0.000	0.209	0.000	0.000	0.000	0.158	0.666	0.037	4.790	3.909	0.063	0.000	0.000	0.045
0.000	0.000	0.000	0.419	0.000	0.034	0.000	0.276	1.668	0.000	4.347	4.125	0.070	0.049	0.000	0.000
0.021	0.000	0.029	0.678	0.076	0.035	0.000	0.548	1.686	0.043	4.077	5.867	0.073	0.021	0.000	0.052
0.000	0.000	0.000	1.532	0.029	0.040	0.000	0.325	1.907	0.048	2.455	7.113	0.275	0.046	0.000	0.029
0.000	0.000	0.057	1.253	0.152	0.000	0.124	0.351	1.919	0.000	4.473	5.670	0.242	0.020	0.000	0.000
0.021	0.040	0.000	0.720	0.050	0.070	0.124	1.598	1.338	0.042	3.122	8.720	0.072	0.051	0.000	0.180
0.000	0.000	0.000	0.434	0.076	0.282	0.000	1.321	0.288	0.000	2.275	9.436	0.073	0.010	0.000	0.233
0.009	0.000	0.000	0.953	0.000	0.029	0.000	0.262	8.618	0.070	3.694	9.509	0.020	0.000	0.000	0.021
0.018	0.000	0.000	0.957	0.066	0.030	0.054	1.111	1.491	0.073	2.380	5.920	0.042	0.018	0.075	0.111
0.010	0.000	0.000	0.484	0.076	0.000	0.000	0.685	8.676	0.000	2.388	6.056	0.024	0.010	0.000	0.181
0.000	0.000	0.000	0.607	0.000	0.102	0.060	0.528	1.068	0.041	2.245	8.249	0.094	0.040	0.000	0.075
0.000	0.000	0.000	0.394	0.000	0.072	0.000	0.412	0.098	0.000	2.723	4.629	0.099	0.042	0.000	0.000
0.000	0.075	0.053	0.714	0.047	0.000	0.000	0.325	0.667	0.000	3.027	4.763	0.112	0.000	0.000	0.048
0.018	0.036	0.051	4.604	0.291	0.000	0.000	0.512	0.382	0.000	2.556	7.828	0.043	0.045	0.000	0.160
0.000	0.000	0.000	1.022	0.045	0.000	0.110	0.689	0.848	0.000	4.444	4.877	0.086	0.072	0.000	0.046
0.000	0.043	0.000	2.235	0.134	0.037	0.000	0.425	1.315	0.045	2.440	5.977	0.179	0.022	0.091	0.027
0.046	0.176	0.031	0.892	0.110	0.384	0.000	0.778	2.873	0.092	3.466	4.705	0.079	0.011	0.000	0.197
0.000	0.000	0.000	0.574	0.082	0.000	0.000	0.324	8.317	0.046	3.937	4.991	0.079	0.033	0.000	0.224
0.019	0.000	0.000	0.618	0.046	0.032	0.114	0.327	1.010	0.078	3.268	4.244	0.089	0.019	0.000	0.165
0.032	0.041	0.029	0.437	0.077	0.000	0.000	0.681	0.822	0.000	2.767	4.980	0.073	0.021	0.000	0.026
0.036	0.035	0.000	1.167	0.088	0.030	0.000	0.568	2.116	0.000	2.590	3.978	0.042	0.018	0.075	0.067
0.058	0.000	0.000	0.533	0.187	0.032	0.114	1.317	3.759	0.000	2.534	4.943	0.089	0.066	0.000	0.214
0.019	0.036	0.000	2.123	0.091	0.032	0.000	0.228	0.906	0.076	4.359	6.863	0.109	0.009	0.000	0.116
0.021	0.040	0.028	0.716	0.201	0.000	0.000	0.064	0.760	0.042	2.383	4.928	0.216	0.020	0.000	0.102
0.021	0.000	0.029	0.676	0.051	0.000	0.000	0.618	2.740	0.000	2.850	7.233	0.097	0.092	0.000	0.155
0.000	0.000	0.000	0.754	0.044	0.000	0.054	0.310	0.417	0.000	2.634	6.322	0.063	0.009	0.000	0.090
0.000	0.000	0.023	0.830	0.021	0.000	0.000	0.096	1.141	0.104	4.852	5.975	0.099	0.025	0.000	0.000
0.009	0.000	0.024	0.795	0.021	0.058	0.000	0.327	3.364	0.000	3.209	5.320	0.020	0.059	0.071	0.043
0.019	0.072	0.000	0.855	0.000	0.062	0.000	0.150	1.787	0.000	3.450	5.707	0.021	0.018	0.000	0.000
0.009	0.000	0.000	0.958	0.046	0.000	0.056	0.264	1.517	0.000	2.578	6.128	0.175	0.009	0.000	0.047
0.000	0.000	0.000	0.385	0.182	0.056	0.000	0.539	1.838	0.068	2.339	4.105	0.097	0.008	0.069	0.041
0.000	0.178	0.000	0.708	0.186	0.518	0.000	0.497	5.642	0.062	2.214	9.470	0.249	0.075	0.000	0.152
0.000	0.000	0.000	0.923	0.000	0.000	0.000	0.398	1.470	0.000	1.713	2.926	0.000	0.000	0.000	0.000
0.000	0.000	0.000	0.963	0.072	0.067	0.000	0.408	1.005	0.040	2.850	5.839	0.138	0.049	0.000	0.049
0.009	0.109	0.000	0.477	0.182	0.000	0.000	0.538	1.941	0.000	2.251	5.665	0.152	0.018	0.078	0.023
0.009	0.000	0.000	0.646	0.068	0.031	0.000	0.232	1.843	0.076	2.920	3.695	0.151	0.027	0.000	0.000
0.009	0.000	0.000	0.803	0.021	0.029	0.000	0.211	1.560	0.035	3.416	7.031	0.101	0.017	0.000	0.022
0.010	0.078	0.028	1.904	0.049	0.034	0.060	0.394	0.740	0.000	2.899	5.725	0.163	0.020	0.000	0.050
0.000	0.000	0.000	0.442	0.000	0.054	0.000	0.655	5.647	0.129	1.749	6.441	0.185	0.094	0.000	0.039
0.000	0.000	0.023	0.805	0.040	0.000	0.000	0.729	0.687	0.034	3.549	4.342	0.096	0.016	0.069	0.123
0.000	0.000	0.024	0.658	0.173	0.030	0.000	0.416	1.515	0.036	3.823	4.156	0.103	0.026	0.000	0.088
0.000	0.000	0.000	0.917	0.000	0.067	0.000	0.328	9.994	0.000	1.864	5.692	0.092	0.000	0.000	0.025
0.000	0.000	0.000	1.295	0.000	0.000	0.000	0.532	6.097	0.000	2.988	4.904	0.177	0.000	0.000	0.000
0.018	0.000	0.000	0.727	0.000	0.000	0.000	0.425	5.950	0.000	3.961	6.428	0.203	0.000	0.000	0.000
0.000	0.000	0.000	0.646	0.000	0.000	0.000	0.319	25.036	0.000	2.557	7.326	0.054	0.000	0.097	0.115
0.016	0.000	0.000	0.215	0.038	0.000	0.000	0.425	8.696	0.000	3.075	5.220	0.072	0.000	0.000	0.153
0.000	0.047	0.000	0.283	0.000	0.000	0.000	0.160	39.862	0.000	2.641	6.026	0.057	0.000	0.000	0.000
0.000	0.000	0.000	0.948	0.000	0.030	0.000	0.233	1.806	0.000	1.957	5.901	0.104	0.009	0.000	0.000
0.000	0.000	0.000	0.417	0.000	0.000	0.000	0.079	1.659	0.000	2.456	10.767	0.042	0.000	0.000	0.089
0.000	0.032	0.000	1.133	0.040	0.000	0.000	0.239	2.782	0.000	3.605	4.629	0.057	0.032	0.000	0.081
0.000	0.000	0.044	0.744	0.000	0.000	0.000	0.522	2.815	0.000	3.539	7.732	0.187	0.000	0.000	0.080
0.000	0.000	0.000	0.624	0.060	0.000	0.000	0.543	6.895	0.000	3.645	3.887	0.086	0.000	0.000	0.091
0.030	0.000	0.000	0.752	0.072	0.000	0.000	0.618	0.953	0.000	0.911	5.626	0.034	0.044	0.000	0.037
0.000	0.000	0.000	0.669	0.000	0.035	0.000	0.329	1.238	0.042	2.463	9.587	0.048	0.000	0.000	0.154
0.062	0.000	0.000	2.294	0.000	0.000	0.000	1.237	0.856	0.000	1.077	2.082	0.576	0.000	0.000	0.000
0.000	0.000	0.000	0.723	0.032	0.000	0.000	0.260	4.977	0.000	1.669	8.043	0.061	0.013	0.108	0.322
0.000	0.050	0.000	0.360	0.032	0.000	0.000	0.292	2.511	0.053	1.260	4.184	0.030	0.000	0.000	0.064
0.000	0.000	0.000	0.716	0.000	0.000	0.071	0.304	12.612	0.048	0.933	6.702	0.138	0.012	0.000	0.029
0.000	0.072	0.000	0.173	0.000	0.000	0.000	0.390	3.783	0.000	1.778	6.137	0.065	0.000	0.000	0.046
0.000	0.043	0.030	1.026	0.000	0.037	0.000	0.615	1.685	0.090	2.209	6.893	0.000	0.011	0.184	0.027
0.025	0.000	0.000	0.580	0.092	0.000	0.149	0.532	3.409	0.051	3.472	4.594	0.029	0.012	0.000	0.031
0.021	0.081	0.000	1.503	0.051	0.071	0.000	0.399	2.414	0.000	2.697	4.448	0.049	0.010	0.000	0.026
0.000	0.086	0.000	0.464	0.000	0.000	0.000	0.285	0.359	0.091	3.330	2.275	0.104	0.011	0.092	0.028
0.009	0.034	0.048	0.608	0.170	0.059	0.000	0.907	2.582	0.036	4.132	5.038	0.041	0.198	0.073	0.065
0.018	0.071	0.000	1.226	0.178	0.000	0.000	0.268	1.809	0.074	4.388	4.760	0.085	0.009	0.000	0.000
0.008	0.000	0.000	0.540	0.122	0.141	0.000	0.635	1.307	0.000	3.173	4.428	0.116	0.016	0.069	0.041
0.000	0.063	0.000	1.654	0.040	0.000	0.000	0.477	1.572	0.066	2.856	5.951	0.151	0.032	0.000	0.000
0.017	0.067	0.000	1.646	0.106	0.293	0.000	1.591	11.112	0.176	3.619	6.587	0.101	0.034	0.000	0.107
0.000	0.000	0.025	1.534	0.022	0.061	0.000	0.520	0.413	0.146	4.071	6.094	0.062	0.018	0.000	0.044
0.000	0.033	0.047	1.200	0.000	0.029	0.000	0.070	0.876	0.035	3.767	9.010	0.100	0.000	0.000	0.000
0.027	0.035	0.000	1.721	0.088	0.031	0.000	0.696	1.755	0.111	1.633	6.001	0.295	0.107	0.000	0.112
0.009	0.068	0.000	0.971	0.000	0.059	0.000	0.540	1.410	0.036	3.443	4.706	0.122	0.060	0.290	0.043
0.031	0.000	0.000	2.487	0.123	0.000	0.000	0.259	0.934	0.124	3.093	8.582	0.047	0.020	0.000	0.050
0.000	0.000	0.000	1.278	0.026	0.072	0.000	0.550	5.874	0.216	3.373	3.461	0.123	0.010	0.000	0.026
0.087	0.672	0.000	0.601	0.000	0.147	0.000	1.324	3.793	0.528	4.049	6.091	0.201	0.255	0.000	0.107
0.000	0.000	0.075	2.411	0.267	0.093	0.000	0.325	0.758	0.000	2.725	7.241	0.319	0.000	0.000	0.136
0.000	0.000	0.000	1.035	0.000	0.108	0.000	0.897	3.188	0.000	2.412	3.979	0.049	0.021	0.088	0.105
0.032	0.000	0.000	0.785	0.077	0.108	0.063	0.503	1.222	0.043	2.066	6.373	0.099	0.083	0.000	0.263
0.000	0.000	0.000	0.321	0.000	0.047	0.083	0.147	2.425	0.056	1.072	11.087	0.097	0.000	0.000	0.172
0.000	0.000	0.025	1.708	0.022	0.000	0.054	0.343	1.866	0.037	3.031	9.542	0.021	0.053	0.000	0.045
0.000	0.045	0.000	0.591	0.000	0.039	0.000	0.362	0.641	0.000	2.248	5.776	0.081	0.046	0.000	0.057
0.027	0.052	0.000	0.554	0.032	0.000	1.110	0.788	1.716	0.054	2.945	2.180	0.186	0.013	0.000	0.132
0.094	0.000	0.000	0.971	0.000	0.079	0.000	0.553	5.692	0.284	2.752	4.381	0.217	0.069	0.000	0.000
0.000	0.000	0.000	0.790	0.026	0.072	0.000	0.280	0.983	0.043	2.656	7.260	0.074	0.042	0.088	0.079
0.000	0.073	0.000	1.052	0.000	0.000	0.000	0.195	6.284	0.000	2.404	6.716	0.044	0.028	0.079	0.000
0.027	0.000	0.000	0.823	0.000	0.090	0.000	0.361	1.106	0.072	2.907	6.543	0.041	0.000	0.000	0.044
0.000	0.000	0.000	0.361	0.027	0.000	0.000	0.327	1.026	0.000	3.609	5.478	0.155	0.011	0.092	0.000
0.000	0.000	0.000	0.681	0.000	0.000	0.000	0.026	2.129	0.000	1.612	10.143	0.098	0.010	0.087	0.052
0.020	0.076	0.000	0.912	0.024	0.033	0.000	0.523	5.677	0.000	2.179	4.144	0.069	0.019	0.082	0.024
0.000	0.000	0.000	0.377	0.025	0.000	0.000	0.432	1.642	0.000	2.211	4.450	0.142	0.020	0.000	0.000
0.000	0.000	0.000	0.404	0.053	0.000	0.000	0.721	2.111	0.266	4.329	4.100	0.203	0.064	0.000	0.000
0.000	0.000	0.000	0.815	0.025	0.035	0.000	0.213	4.297	0.000	4.408	4.592	0.193	0.010	0.000	0.000
0.000	0.000	0.000	0.713	0.027	0.037	0.000	0.220	0.659	0.089	2.671	7.646	0.077	0.032	0.000	0.055
0.019	0.000	0.000	0.483	0.023	0.161	0.000	0.681	8.704	0.000	3.692	3.796	0.132	0.028	0.000	0.047
0.000	0.170	0.000	1.373	0.000	0.000	0.000	0.459	0.658	0.045	1.327	4.892	0.204	0.000	0.000	0.027
0.000	0.000	0.000	0.883	0.000	0.065	0.000	0.417	1.494	0.000	2.049	5.364	0.155	0.000	0.000	0.000
0.000	0.000	0.025	0.294	0.000	0.031	0.000	0.668	1.337	0.037	2.639	3.895	0.190	0.018	0.000	0.067
0.008	0.032	0.000	0.835	0.080	0.000	0.000	0.302	1.777	0.067	4.101	3.945	0.114	0.024	0.000	0.081
0.000	0.000	0.000	0.572	0.030	0.000	0.000	0.432	4.326	0.050	2.861	2.991	0.345	0.024	0.000	0.000
0.000	0.000	0.000	0.551	0.000	0.000	0.000	0.149	0.050	0.044	1.484	3.215	0.075	0.000	0.000	0.000
0.000	0.000	0.000	0.796	0.000	0.000	0.000	0.329	2.526	0.000	1.361	7.881	0.050	0.011	0.000	0.000
0.010	0.000	0.028	2.008	0.196	0.034	0.000	0.636	0.976	0.164	2.952	6.526	0.117	0.030	0.084	0.075
0.010	0.037	0.026	0.668	0.023	0.065	0.000	0.336	1.951	0.000	3.070	4.118	0.179	0.038	0.000	0.048
0.063	0.000	0.000	0.581	0.000	0.106	0.000	0.333	5.637	0.000	3.127	6.261	0.292	0.062	0.000	0.155
0.033	0.032	0.023	0.499	0.061	0.028	0.000	0.585	0.917	0.034	2.184	6.649	0.096	0.016	0.000	0.164
0.015	0.000	0.000	0.320	0.000	0.026	0.000	0.178	0.567	0.000	3.922	4.479	0.107	0.045	0.000	0.019
0.017	0.097	0.023	1.387	0.142	0.000	0.000	0.909	1.035	0.237	3.761	5.069	0.135	0.033	0.000	0.062
0.016	0.000	0.000	0.257	0.019	0.000	0.047	0.618	1.459	0.000	3.327	4.687	0.074	0.023	0.000	0.039
0.025	0.000	0.000	0.462	0.061	0.000	0.000	0.218	1.494	0.101	2.797	6.380	0.174	0.024	0.207	0.000
0.000	0.000	0.000	0.542	0.000	0.030	0.000	0.304	9.756	0.037	2.659	4.616	0.105	0.018	0.000	0.022
0.000	0.000	0.032	1.178	0.028	0.078	0.000	0.917	1.653	0.047	5.944	8.160	0.135	0.068	0.000	0.201
0.024	0.000	0.000	0.546	0.057	0.120	0.000	0.625	3.097	0.000	2.765	7.270	0.164	0.069	0.000	0.029
0.026	0.000	0.000	1.818	0.064	0.133	0.000	0.417	0.905	0.160	3.276	5.773	0.152	0.039	0.000	0.000
0.000	0.000	0.000	0.544	0.286	0.000	0.088	0.330	2.100	0.000	4.827	4.235	0.068	0.014	0.000	0.073
0.000	0.046	0.033	1.768	0.116	0.000	0.000	0.961	5.446	0.049	2.549	5.699	0.167	0.012	0.000	0.000
0.000	0.000	0.000	2.618	0.020	0.084	0.000	0.369	0.805	0.000	4.079	5.135	0.155	0.008	0.000	0.041
0.009	0.000	0.000	1.263	0.000	0.000	0.000	0.309	1.651	0.000	1.295	4.300	0.099	0.008	0.000	0.085
0.015	0.000	0.000	1.832	0.037	0.000	0.000	0.285	0.982	0.062	2.781	5.033	0.283	0.000	0.000	0.000
0.000	0.061	0.000	0.367	0.000	0.000	0.000	0.178	4.131	0.000	3.354	4.326	0.111	0.008	0.000	0.020
0.000	0.042	0.000	2.636	0.131	0.000	0.000	0.255	0.594	0.000	2.918	9.875	0.100	0.021	0.000	0.080
0.010	0.000	0.000	0.894	0.117	0.065	0.000	0.844	1.201	0.000	3.626	7.365	0.180	0.028	0.000	0.191
0.000	0.073	0.000	1.053	0.023	0.000	0.000	0.408	3.014	0.039	2.624	5.780	0.220	0.009	0.000	0.000

ENSG00000231466	ENSG00000137776	ENSG00000244753	ENSG00000132002	ENSG00000138376	ENSG00000118655	ENSG00000178104	ENSG00000261670	ENSG00000261314	ENSG00000074219	ENSG00000187166	ENSG00000018236	ENSG00000204959	ENSG00000180673	ENSG00000269345	ENSG00000170270
0.159	6.948	0.175	36.113	2.945	4.083	8.351	0.022	0.000	5.191	0.095	68.606	0.049	0.189	0.000	7.356
0.135	5.933	0.148	99.985	1.602	3.146	5.751	0.014	0.000	14.193	0.049	6.791	0.200	0.064	0.000	14.022
0.264	8.280	0.099	39.118	3.776	6.594	2.065	0.081	0.000	16.172	0.000	20.780	0.000	0.057	1.055	13.796
0.163	8.467	0.187	75.470	3.162	4.858	6.766	0.042	0.000	18.398	0.082	7.553	0.005	0.172	0.000	21.402
0.091	6.345	0.083	146.459	3.104	3.576	2.085	0.036	0.000	6.645	0.145	2.293	0.021	0.132	0.000	19.531
0.311	9.640	0.033	19.683	6.004	7.929	4.935	0.093	0.052	14.972	0.215	51.452	0.025	0.123	2.408	7.742
0.108	8.347	0.037	43.061	3.205	6.146	3.372	0.014	0.030	15.786	0.129	12.028	0.046	0.043	0.000	12.521
0.057	4.202	0.078	39.838	1.380	3.062	4.529	0.010	0.000	2.997	0.085	28.483	0.010	0.034	0.104	8.143
0.395	3.509	0.272	49.334	2.759	3.531	5.474	0.022	0.000	8.158	0.138	27.945	0.011	0.026	0.000	16.283
0.192	4.278	0.264	50.079	1.638	3.053	2.394	0.000	0.105	6.093	0.057	3.152	0.099	0.228	0.000	20.849
0.165	6.075	0.194	62.768	2.200	2.801	5.052	0.032	0.052	7.578	0.325	13.539	0.109	0.056	0.043	10.389
0.049	9.978	0.101	71.664	4.241	5.865	3.022	0.045	0.000	9.425	0.029	4.239	0.042	0.106	0.089	8.135
0.201	5.731	0.035	72.676	2.586	4.977	3.293	0.021	0.000	12.924	0.030	36.369	0.013	0.100	0.000	12.812
0.141	8.649	0.129	137.385	4.048	5.292	4.617	0.032	0.000	8.807	0.141	13.742	0.024	0.112	0.043	9.360
0.105	5.160	0.288	79.031	3.125	9.085	4.568	0.000	0.000	10.174	0.063	11.519	0.072	0.249	0.000	13.455
0.188	9.448	0.065	51.554	1.664	4.002	4.920	0.036	0.026	2.563	0.028	3.320	0.097	0.103	0.000	5.775
0.109	6.003	0.075	60.846	4.470	7.098	4.212	0.028	0.030	7.672	0.065	26.330	0.009	0.118	0.000	9.613
0.064	1.365	0.222	55.785	2.142	2.526	2.597	0.038	0.035	31.270	0.019	13.699	0.017	0.038	0.885	19.992
0.049	4.811	0.169	107.195	4.120	3.436	4.837	0.029	0.000	11.301	0.103	8.584	0.017	0.097	0.000	16.920
0.131	6.907	0.072	57.679	4.933	4.265	6.961	0.022	0.000	15.923	0.110	14.872	0.027	0.062	0.000	10.558
0.140	1.934	0.192	62.018	3.104	2.782	3.536	0.014	0.000	15.322	0.050	9.512	0.000	0.011	0.051	27.649
0.520	12.959	0.000	22.235	3.116	6.050	5.797	0.069	0.000	11.915	0.243	36.309	0.040	0.183	0.000	4.647
0.028	4.191	0.039	75.458	2.289	3.755	3.161	0.053	0.000	8.015	0.051	23.424	0.000	0.078	0.052	16.996
0.024	5.910	0.033	62.196	2.161	3.270	2.448	0.008	0.000	17.364	0.029	1.402	0.351	0.029	0.000	10.532
0.027	6.986	0.074	121.314	2.375	4.562	3.806	0.082	0.000	8.949	0.016	9.779	0.028	0.064	0.000	10.407
0.250	7.362	0.268	106.630	3.684	5.602	5.078	0.047	0.000	4.525	0.033	46.625	0.010	0.044	0.000	9.093
0.157	4.296	0.216	44.746	1.722	3.578	13.148	0.021	0.000	10.121	0.170	37.362	0.049	0.087	0.173	11.261
0.111	4.907	0.152	71.265	2.178	4.361	2.335	0.019	0.000	7.056	0.033	14.321	0.048	0.088	0.000	8.455
0.303	9.102	0.493	54.072	1.453	2.892	2.826	0.084	0.000	11.494	0.430	22.418	0.019	0.022	0.000	14.962
0.499	7.983	0.038	50.294	2.983	3.222	2.343	0.089	0.000	25.747	0.083	3.926	0.014	0.044	0.000	14.860
0.046	5.500	0.157	64.227	2.941	5.770	2.984	0.012	0.000	5.265	0.027	20.887	0.031	0.018	0.000	13.765
0.406	10.453	0.033	53.477	3.930	3.757	5.011	0.114	0.000	6.387	0.143	11.391	0.000	0.104	0.000	12.422
0.139	6.576	0.268	126.126	1.090	2.340	4.602	0.057	0.031	8.093	0.083	24.648	0.005	0.011	0.051	15.337
0.027	6.335	0.111	43.225	1.960	2.886	4.169	0.009	0.029	5.443	0.032	10.703	0.009	0.011	0.000	9.836
0.113	8.990	0.467	60.511	3.493	5.423	2.971	0.000	0.000	7.615	0.170	2.004	0.019	0.157	0.104	6.427
0.205	9.304	0.071	62.280	1.978	4.015	4.676	0.030	0.028	5.013	0.108	10.212	0.004	0.122	0.047	11.090
0.171	12.559	0.168	36.601	3.450	6.733	6.488	0.017	0.054	7.370	0.088	33.650	0.034	0.320	0.000	9.037
0.196	9.475	0.067	30.757	5.222	7.838	4.370	0.029	0.027	19.260	0.161	17.794	0.013	0.116	0.000	10.711
0.146	4.938	0.080	49.836	3.289	5.244	4.704	0.074	0.000	6.094	0.035	34.084	0.025	0.139	0.374	17.628
0.271	9.133	0.166	99.538	5.398	6.080	3.903	0.031	0.000	11.621	0.018	13.547	0.057	0.096	0.000	11.470
0.210	9.145	0.248	69.577	2.649	3.782	8.246	0.051	0.000	3.854	0.306	16.782	0.021	0.024	0.055	7.135
0.076	11.476	0.035	105.723	2.069	4.596	1.384	0.017	0.000	9.341	0.106	3.008	0.031	0.070	0.000	11.273
0.446	8.369	0.038	80.613	3.708	3.727	1.901	0.024	0.000	6.359	0.100	1.890	0.014	0.166	0.000	21.002
0.168	9.814	0.033	42.202	3.829	4.351	6.923	0.004	0.053	16.335	0.043	3.507	0.029	0.133	0.000	10.210
0.816	12.885	0.105	49.062	3.621	4.772	1.942	0.104	0.000	19.030	0.321	5.272	0.031	0.182	0.000	11.759
0.174	7.490	0.240	82.406	7.057	7.029	6.384	0.008	0.000	12.419	0.224	18.184	0.013	0.197	0.000	17.288
3.178	10.546	0.188	38.750	5.812	8.234	2.574	0.074	0.030	68.948	0.082	0.961	0.005	0.087	3.411	13.723
0.388	8.524	0.343	49.588	3.176	4.984	5.699	0.118	0.000	8.586	0.083	50.964	0.014	0.121	2.132	9.333
0.096	10.813	0.165	99.519	4.247	5.355	5.483	0.012	0.000	12.134	0.115	13.380	0.012	0.114	0.088	13.327
0.136	6.057	0.062	68.050	1.346	4.095	2.495	0.012	0.000	4.897	0.014	2.657	0.047	0.108	0.125	10.681
0.274	6.548	0.063	39.582	1.438	3.833	3.859	0.027	0.025	2.677	0.123	28.426	0.028	0.190	0.334	12.686
0.074	7.285	0.101	108.044	2.219	4.408	1.861	0.012	0.054	5.269	0.074	5.329	0.030	0.146	0.000	15.598
0.050	8.168	0.275	40.214	4.123	6.770	7.283	0.017	0.000	20.480	0.060	42.407	0.034	0.257	0.000	26.038
0.088	9.129	0.091	74.650	3.500	4.741	4.177	0.037	0.048	13.041	0.080	0.927	0.011	0.123	0.000	15.441
0.285	4.976	0.224	107.787	2.746	6.162	3.201	0.076	0.000	2.205	0.049	50.372	0.035	0.306	0.224	13.558
0.000	9.040	0.000	35.143	3.979	6.315	17.561	0.072	0.000	8.045	0.064	31.039	0.000	0.462	5.045	9.743
0.079	6.862	0.036	105.562	3.732	5.116	5.738	0.031	0.000	10.832	0.016	7.111	0.014	0.115	0.000	12.784
0.075	7.733	0.137	57.547	3.139	6.450	10.502	0.097	0.000	3.688	0.090	21.796	0.026	0.128	0.046	13.426
0.099	11.572	0.238	47.517	4.009	7.339	7.415	0.025	0.000	13.380	0.045	3.388	0.017	0.372	0.000	9.963
0.208	7.711	0.000	60.656	8.217	5.276	7.994	0.012	0.025	16.481	0.374	29.287	0.024	0.165	0.000	11.712
0.160	6.350	0.147	54.273	6.377	7.296	5.846	0.009	0.029	12.306	0.032	16.092	0.000	0.169	0.000	6.687
0.000	5.769	0.233	118.060	2.921	4.272	2.634	0.072	0.000	8.483	0.025	22.153	0.000	0.084	0.000	14.671
0.176	4.925	0.061	64.865	3.673	4.413	5.750	0.071	0.000	3.206	0.053	41.224	0.015	0.192	0.040	6.000
0.094	5.979	0.097	140.043	5.551	7.645	5.817	0.012	0.052	11.870	0.071	2.420	0.012	0.140	0.000	13.858
0.132	5.395	0.072	70.697	4.810	5.790	6.804	0.063	0.000	22.264	0.016	12.893	0.005	0.094	1.157	10.552
0.068	3.212	0.140	38.120	2.765	3.776	5.074	0.017	0.000	6.029	0.020	19.958	0.012	0.067	0.000	12.642
0.232	4.946	0.064	56.801	4.420	5.455	4.626	0.016	0.000	7.553	0.056	29.704	0.000	0.165	0.000	13.232
0.000	3.165	0.000	208.920	3.177	2.908	4.890	0.021	0.000	15.438	0.037	12.365	0.000	0.098	0.000	23.519
0.288	8.434	0.113	37.001	7.534	7.131	8.471	0.021	0.000	10.979	0.000	0.642	0.007	0.195	0.000	11.979
0.065	4.162	0.089	84.943	3.128	5.472	5.889	0.022	0.000	8.289	0.019	22.204	0.011	0.129	0.000	14.578
0.047	7.520	0.098	54.689	4.279	4.743	5.135	0.008	0.026	17.047	0.000	1.994	0.008	0.122	0.000	18.194
0.000	1.620	0.329	92.045	0.837	1.734	4.874	0.008	0.000	5.862	0.057	19.107	0.008	0.095	0.000	21.995
0.108	6.420	0.060	81.211	1.643	2.830	3.031	0.007	0.071	4.633	0.104	6.391	0.090	0.129	0.000	8.342
0.085	7.259	0.235	93.668	4.012	6.144	1.753	0.007	0.000	16.894	0.077	1.228	0.007	0.051	0.078	19.560
0.228	10.164	0.179	105.639	2.176	3.828	2.687	0.083	0.036	7.006	0.000	1.144	0.039	0.077	0.000	6.722
0.157	8.479	0.216	47.244	3.430	4.083	3.420	0.087	0.000	13.585	0.330	21.675	0.014	0.125	0.000	15.588
0.055	2.600	0.076	151.174	3.831	4.822	2.531	0.014	0.000	23.231	0.066	9.692	0.009	0.076	0.000	24.427
0.494	8.520	0.227	45.305	3.091	7.695	6.576	0.000	0.000	114.797	0.000	2.846	0.680	0.261	0.302	7.792
0.104	3.103	0.143	83.947	4.724	2.425	4.195	0.029	0.000	27.809	0.062	4.477	0.006	0.069	0.000	44.336
0.000	2.982	0.332	82.389	3.393	3.674	4.485	0.023	0.038	8.371	0.041	24.420	0.006	0.096	0.000	24.250
0.000	1.546	0.131	57.637	3.782	2.349	5.127	0.005	0.000	6.227	0.000	27.314	0.005	0.038	0.000	44.348
0.050	5.375	0.068	97.329	2.494	3.968	4.720	0.013	0.000	7.064	0.060	3.419	0.017	0.128	0.045	26.103
0.118	5.582	0.162	38.872	3.215	3.464	5.315	0.025	0.065	6.044	0.124	14.633	0.015	0.047	0.000	11.328
0.100	6.430	0.092	55.868	2.786	4.594	14.737	0.045	0.037	10.375	0.160	18.118	0.023	0.079	0.000	9.825
0.111	9.894	0.000	49.489	2.807	4.946	4.779	0.052	0.031	2.304	0.017	16.142	0.038	0.066	0.102	6.683
0.030	8.073	0.204	90.807	3.584	5.103	3.347	0.030	0.032	4.338	0.000	1.060	0.209	0.023	0.000	9.941
0.582	10.010	0.096	51.636	3.956	5.653	2.906	0.067	0.000	1.499	0.070	25.361	0.000	5.532	0.043	4.169
0.073	7.670	0.534	45.362	6.866	11.322	6.511	0.004	0.027	7.955	0.102	4.739	0.038	0.106	0.000	10.468
0.266	10.449	0.275	45.754	5.240	5.316	7.421	0.011	0.024	13.291	0.253	11.095	0.027	0.149	0.000	9.532
0.173	7.356	0.119	49.370	2.921	3.446	9.367	0.007	0.000	2.352	0.259	9.178	0.030	0.205	0.079	9.138
0.254	11.773	0.063	24.673	6.264	5.090	4.179	0.094	0.051	1.986	0.263	17.029	0.016	0.064	0.000	9.960
0.024	6.629	0.098	79.755	4.796	6.688	10.729	0.057	0.026	9.247	0.143	5.200	0.012	0.047	0.000	11.570
0.069	5.982	0.126	54.641	3.202	4.785	9.336	0.023	0.000	2.261	0.055	47.644	0.020	0.118	0.000	4.783
0.072	17.382	0.033	26.106	4.862	7.992	14.806	0.119	0.000	23.840	0.087	13.422	0.033	0.095	0.044	8.602
0.139	8.678	0.128	26.578	3.579	3.717	10.107	0.083	0.025	1.356	0.307	47.641	0.032	0.221	0.808	7.099
0.108	8.354	0.074	100.318	6.134	6.894	4.868	0.037	0.059	13.638	0.049	0.620	0.009	0.139	0.000	10.858
0.282	8.361	0.155	42.616	3.968	6.491	7.863	0.072	0.000	11.716	0.000	15.445	0.044	0.101	2.223	9.659
0.576	9.503	0.317	34.050	5.433	5.494	3.333	0.078	0.000	1.236	0.138	51.182	0.079	0.091	2.109	5.760
0.364	12.274	0.100	32.562	5.257	4.964	3.697	0.037	0.000	9.043	0.131	5.001	0.013	0.202	0.000	18.968
0.113	10.584	0.195	98.743	4.380	5.237	3.747	0.053	0.000	8.065	0.153	3.741	0.015	0.101	0.000	10.308
0.141	6.859	0.194	80.382	3.784	5.163	6.250	0.057	0.031	6.819	0.034	14.481	0.078	0.536	1.033	8.614
0.000	2.503	0.203	65.551	4.687	1.742	3.796	0.006	0.000	10.380	0.000	3.439	0.000	0.088	0.000	38.003
0.167	8.724	0.889	91.456	4.789	4.267	8.351	0.016	0.000	9.398	0.100	3.551	0.276	0.028	0.000	21.424
0.062	7.784	0.042	48.379	3.152	4.469	6.422	0.005	0.000	11.711	0.056	15.525	0.037	0.195	0.169	11.422
0.071	10.772	0.049	25.169	1.905	4.970	1.579	0.096	0.039	2.989	0.594	6.442	0.037	0.070	0.000	13.240
0.062	7.590	0.085	29.337	2.661	4.858	2.757	0.021	0.000	2.043	0.112	28.493	0.000	0.074	0.000	8.250
0.113	4.906	0.000	44.651	2.549	6.368	5.877	0.024	0.000	4.209	0.051	16.421	0.010	0.045	0.104	13.957
0.252	5.479	0.069	39.411	3.900	5.527	3.164	0.077	0.028	11.400	0.015	63.970	0.009	0.229	1.429	8.134
0.071	7.564	0.033	45.742	4.165	7.537	1.703	0.032	0.000	2.394	0.028	34.561	0.000	0.066	0.000	12.443
0.030	2.766	0.041	186.804	2.509	4.261	1.455	0.025	0.000	40.439	0.053	2.731	0.005	0.047	0.054	24.316
0.000	3.351	0.154	45.227	3.493	3.366	5.363	0.000	0.000	4.779	0.000	4.421	0.005	0.022	0.000	22.155
0.157	9.171	0.108	64.354	4.565	5.841	5.787	0.018	0.057	6.605	0.031	10.015	0.045	0.125	0.096	16.453
0.054	8.473	0.298	28.743	3.435	5.226	11.592	0.009	0.000	10.629	0.130	11.334	0.023	0.075	0.000	10.247
0.464	10.026	0.000	37.793	3.579	6.291	3.807	0.030	0.000	7.013	0.070	16.797	0.010	0.138	0.106	10.152
0.028	7.444	0.114	110.711	4.721	8.873	9.151	0.005	0.000	7.528	0.066	2.687	0.000	0.055	0.000	14.020
0.029	5.508	0.201	97.103	3.159	3.926	4.276	0.005	0.032	8.798	0.070	1.303	0.025	0.139	0.000	12.437
0.606	8.066	0.104	80.605	3.857	4.021	7.696	0.034	0.000	1.678	0.151	88.565	0.030	0.160	0.000	10.728
0.000	10.946	0.281	26.401	2.472	4.633	13.317	0.010	0.000	11.303	0.175	49.498	0.045	0.162	0.000	11.217
0.051	11.418	0.209	55.449	3.523	6.238	2.293	0.030	0.056	18.314	0.015	8.232	0.026	0.121	0.000	20.785
0.265	11.446	0.066	76.768	4.929	7.943	2.559	0.020	0.026	22.083	0.087	12.469	0.021	0.287	0.000	10.023
0.131	8.304	0.120	64.909	2.858	6.171	5.554	0.044	0.000	4.048	0.065	31.075	0.064	0.233	0.160	6.959
0.164	8.357	0.000	91.907	2.267	5.540	5.264	0.045	0.000	2.149	0.020	27.788	0.062	0.208	0.481	8.191
0.489	9.303	0.158	31.093	1.162	4.990	2.099	0.010	0.000	54.853	0.052	1.735	0.124	0.080	0.000	7.002
0.086	8.265	0.079	29.830	2.461	4.429	13.005	0.029	0.000	15.114	0.034	39.801	0.025	0.125	0.000	25.898
0.107	8.810	0.221	44.722	4.600	5.761	9.046	0.005	0.059	4.324	0.209	17.239	0.014	0.117	0.000	10.804
0.230	6.471	0.035	55.411	1.102	3.296	3.046	0.052	0.000	5.911	0.169	17.955	0.022	0.101	0.000	8.626
0.083	5.486	0.459	85.230	3.409	3.004	3.014	0.170	0.000	6.024	0.150	11.277	0.029	0.099	0.000	16.739
0.066	9.292	0.243	89.274	2.565	5.425	5.324	0.049	0.024	13.940	0.145	8.818	0.019	0.131	0.000	9.029
0.041	6.530	0.056	61.147	3.832	5.581	5.324	0.017	0.022	13.951	0.037	2.616	0.025	0.089	0.000	8.761
0.177	7.912	0.243	40.129	5.848	8.996	4.724	0.023	0.000	24.315	0.119	8.089	0.027	0.070	0.041	15.671
0.189	8.007	0.203	118.152	4.276	5.213	2.637	0.032	0.046	9.995	0.051	5.194	0.029	0.133	0.000	10.813
0.066	4.019	0.182	40.923	4.790	4.908	3.926	0.011	0.000	8.763	0.000	7.282	0.023	0.184	0.000	17.610
0.120	8.011	0.132	107.901	3.067	6.112	10.184	0.008	0.053	16.888	0.043	22.399	0.008	0.152	0.000	26.176
0.431	4.869	0.127	47.392	1.906	6.419	3.074	0.042	0.000	2.520	0.037	8.123	0.026	0.061	0.000	9.690
0.157	5.589	0.129	61.555	3.405	3.729	4.643	0.043	0.000	19.335	0.113	4.007	0.022	0.062	0.000	12.795
0.174	5.311	0.144	63.397	4.577	4.643	4.544	0.024	0.000	8.388	0.084	10.013	0.054	0.193	0.064	12.113
0.117	7.269	0.161	56.237	1.864	2.832	7.671	0.007	0.000	4.972	0.164	26.163	0.235	0.108	0.000	7.027
0.730	6.009	0.175	64.880	3.170	7.068	6.702	0.086	0.104	3.132	0.095	36.002	0.016	0.226	0.058	7.379
0.133	10.640	0.122	110.355	5.862	7.376	2.201	0.083	0.000	6.957	0.040	1.517	0.000	0.245	0.000	8.923
0.068	8.391	0.062	44.447	3.145	4.471	5.046	0.012	0.000	10.992	0.177	8.288	0.035	0.260	0.000	16.552
0.202	9.237	0.167	84.207	2.872	6.113	3.060	0.034	0.000	8.929	0.097	5.566	0.000	0.176	0.000	13.457
0.232	7.480	0.203	53.166	2.082	3.954	2.893	0.032	0.000	8.109	0.013	7.979	0.015	0.100	0.000	10.919
0.114	7.609	0.157	23.443	2.097	5.807	9.960	0.024	0.000	9.577	0.017	18.549	0.010	0.136	0.000	12.683
0.385	11.654	0.106	41.908	3.590	6.322	4.685	0.026	0.028	15.164	0.154	14.671	0.013	0.224	0.047	12.197
0.101	8.809	0.173	57.031	3.356	7.577	11.815	0.026	0.000	14.794	0.060	4.003	0.009	0.200	0.000	10.560

ENSG00000048052	ENSG00000100055	ENSG00000080166	ENSG00000251281	ENSG00000166619	ENSG00000273628	ENSG00000237433	ENSG00000272627	ENSG00000280333	ENSG00000265194	ENSG00000092208	ENSG00000189241	ENSG00000268621	ENSG00000101951	ENSG00000250619	ENSG00000118849
2.014	4.467	0.325	0.058	11.977	0.978	0.393	0.050	0.185	0.049	3.020	29.839	0.000	0.000	0.000	1.671
1.473	6.079	0.039	0.050	7.600	0.384	0.549	0.043	0.000	0.112	4.006	20.169	0.204	0.000	0.000	21.343
1.853	4.554	1.728	0.000	14.051	0.285	0.638	0.114	0.000	0.012	7.457	29.204	0.086	0.000	0.000	0.712
3.432	1.446	0.205	0.000	12.708	0.451	0.217	0.043	0.158	0.141	6.059	21.620	0.012	0.011	0.000	0.958
1.684	7.562	0.375	0.000	9.514	0.072	0.161	0.048	0.000	0.000	3.113	24.892	0.189	0.000	0.000	8.877
0.998	6.715	4.901	0.000	12.108	2.608	0.487	0.114	0.418	0.773	5.996	27.333	0.309	0.000	0.000	0.083
1.882	15.687	0.145	0.000	13.654	0.255	0.477	0.043	1.254	0.271	4.371	16.828	0.036	0.000	0.000	3.607
1.407	10.810	0.362	0.000	19.660	1.283	0.530	0.090	0.000	0.015	3.825	62.510	0.000	0.000	0.000	5.484
2.124	4.600	0.337	0.000	13.264	0.780	1.457	0.104	0.192	0.094	6.610	18.160	0.044	0.000	0.032	3.593
0.972	14.675	0.059	0.000	7.900	0.000	1.357	0.000	0.558	0.025	4.840	16.647	0.000	0.000	0.000	37.962
1.150	6.687	0.338	0.000	11.388	0.725	0.438	0.261	0.000	0.030	4.155	16.422	0.031	0.000	0.000	8.679
2.332	4.716	0.068	0.000	13.884	0.578	0.431	0.077	0.567	0.006	3.820	26.398	0.011	0.000	0.000	3.553
3.197	4.628	0.211	0.000	17.040	0.477	0.334	0.040	0.146	0.078	4.012	28.895	0.056	0.000	0.000	0.588
1.326	5.298	0.056	0.000	15.818	0.279	0.354	0.037	0.274	0.043	3.310	28.821	0.042	0.000	0.023	1.288
0.733	7.335	0.325	0.000	15.007	0.745	1.020	0.000	0.000	0.000	4.131	28.380	0.000	0.000	0.000	2.230
1.257	25.756	0.168	1.857	10.330	0.279	0.229	0.112	0.548	0.079	1.929	33.349	0.126	0.000	0.000	7.726
4.089	2.453	0.758	0.000	10.570	0.322	0.457	0.043	0.000	0.091	5.878	28.477	0.036	0.000	0.000	1.761
0.950	5.164	2.952	0.000	13.465	1.222	2.111	0.153	0.000	0.025	10.303	10.752	0.000	0.000	0.000	0.438
1.868	9.560	0.035	0.000	12.399	0.407	0.391	0.039	0.000	0.051	4.109	20.584	0.153	0.000	0.000	0.898
2.112	5.535	0.106	0.000	9.698	0.062	0.255	0.041	0.153	0.000	3.892	12.343	0.012	0.000	0.000	0.987
1.783	3.778	0.046	0.000	8.722	0.994	1.039	0.088	0.000	0.029	9.451	15.786	0.062	0.000	0.000	2.183
2.782	2.223	1.135	0.000	20.463	1.575	0.409	0.046	0.168	0.052	3.702	82.099	0.502	0.000	0.000	0.463
4.882	5.452	9.003	0.052	14.659	1.472	1.049	0.000	0.821	0.044	8.433	29.949	0.000	0.000	0.027	4.489
1.884	5.359	0.060	0.044	9.201	0.057	0.425	0.000	0.000	0.000	2.145	21.369	0.000	0.000	0.000	12.999
1.113	15.415	0.097	0.000	12.297	0.190	0.260	0.000	0.000	0.028	3.607	26.003	0.012	0.000	0.000	4.989
1.279	8.899	1.207	0.051	13.112	0.461	0.246	0.088	0.162	0.180	2.697	23.734	0.012	0.000	0.054	5.789
3.002	9.763	1.729	0.000	15.194	0.522	0.501	0.000	0.183	0.122	3.070	22.515	0.028	0.000	0.000	1.711
2.009	3.735	5.123	0.000	11.522	0.525	1.127	0.088	0.000	0.057	6.105	25.261	0.025	0.000	0.000	1.950
1.156	4.310	0.673	0.000	19.360	1.111	0.391	0.044	0.000	0.014	2.853	19.957	0.000	0.000	0.000	1.933
2.338	3.359	0.324	0.000	11.443	0.986	0.982	0.000	0.323	0.007	6.047	17.757	0.000	0.011	0.000	0.832
1.848	4.764	0.205	0.000	19.873	0.108	0.646	0.072	0.000	0.024	3.927	20.869	0.010	0.000	0.022	1.146
1.079	2.051	0.099	0.000	14.199	0.453	0.550	0.000	0.278	0.062	3.843	24.386	0.064	0.000	0.000	0.392
1.008	4.960	0.351	0.000	20.642	0.989	0.394	0.132	0.162	0.007	2.921	32.958	0.000	0.000	0.000	1.901
0.988	6.392	0.252	0.000	13.250	0.381	0.237	0.085	0.156	0.000	2.749	15.838	0.000	0.000	0.000	4.039
2.894	5.611	0.304	0.000	11.569	0.403	0.501	0.000	1.318	0.000	2.284	28.565	0.000	0.000	0.000	24.088
1.815	8.277	0.459	0.000	24.610	0.790	0.454	0.081	0.149	0.046	3.333	41.011	0.011	0.000	0.000	1.270
0.958	3.894	0.514	0.000	17.716	0.232	0.260	0.077	0.713	0.127	4.345	22.246	0.033	0.000	0.000	0.547
2.971	12.836	0.321	0.000	19.094	0.232	0.108	0.116	0.000	0.171	4.452	27.000	0.000	0.000	0.000	2.175
4.076	5.245	0.606	0.000	16.573	0.415	0.284	0.138	0.000	0.113	8.466	30.993	0.000	0.000	0.000	1.577
2.002	3.022	0.524	0.000	8.512	0.857	0.320	0.143	0.175	0.296	6.806	21.905	0.027	0.000	0.058	1.367
1.984	5.547	0.397	0.000	15.717	0.498	0.159	0.095	0.000	0.054	2.171	22.210	0.000	0.012	0.029	2.730
2.722	7.426	0.218	0.000	13.620	0.961	0.157	0.080	0.295	0.092	5.083	29.162	0.000	0.000	0.000	2.405
3.667	3.454	0.046	0.000	13.055	0.132	0.420	0.000	0.000	0.022	5.989	22.940	0.012	0.000	0.000	0.934
4.926	4.895	0.213	0.000	9.250	0.341	0.276	0.000	0.279	0.056	4.440	21.244	0.000	0.000	0.000	0.651
1.452	3.100	0.214	0.000	12.401	1.089	0.700	0.000	0.445	0.013	4.684	27.759	0.000	0.000	0.000	0.258
0.633	6.483	0.625	0.000	12.418	0.354	0.419	0.039	0.145	0.039	5.296	27.710	0.000	0.000	0.024	0.816
0.432	0.538	0.142	0.000	17.146	0.065	0.606	0.390	0.797	0.128	6.930	32.751	0.000	0.000	0.053	0.803
3.321	9.822	0.505	0.000	13.234	1.118	0.393	0.088	0.484	0.237	4.869	30.049	0.309	0.000	0.000	0.687
1.749	3.355	0.336	0.000	15.289	1.482	0.426	0.038	0.000	0.006	2.622	23.345	0.000	0.000	0.000	3.003
1.049	15.706	0.073	0.000	10.805	0.377	0.362	0.000	0.264	0.041	3.224	17.592	0.020	0.000	0.022	23.689
1.826	3.796	0.203	0.000	25.410	1.678	0.465	0.036	0.000	0.118	5.866	50.051	0.010	0.027	0.000	2.230
1.669	8.594	0.048	0.000	11.384	0.175	0.652	0.039	0.000	0.013	4.152	26.738	0.066	0.000	0.000	5.712
3.600	5.621	0.150	0.000	13.695	0.593	0.775	0.040	0.146	0.006	9.688	14.604	0.011	0.010	0.000	5.058
2.602	4.856	0.030	0.000	10.503	0.210	0.469	0.000	0.000	0.023	4.710	17.306	0.000	0.000	0.000	1.557
0.732	1.308	0.345	0.000	11.168	0.579	1.081	0.129	0.474	0.200	11.306	21.155	0.018	0.000	0.079	1.746
1.091	1.543	0.734	0.000	11.062	0.251	0.938	0.000	0.000	0.110	7.020	45.311	0.000	0.000	0.000	0.222
2.476	5.486	0.599	0.000	9.079	0.125	0.700	0.000	0.460	0.014	8.391	19.040	0.012	0.000	0.026	2.674
3.430	4.642	1.181	0.000	13.114	0.118	0.573	0.000	0.000	0.109	4.608	16.146	0.011	0.000	0.024	0.729
2.405	4.916	1.877	0.000	9.602	0.821	0.307	0.039	0.720	0.115	4.175	26.077	0.033	0.000	0.000	0.561
2.367	3.643	0.410	0.000	19.486	0.492	0.102	0.073	0.269	0.024	4.997	27.033	0.000	0.018	0.000	0.523
1.771	2.431	0.210	0.000	13.719	0.885	0.378	0.084	0.000	0.124	5.652	26.936	0.012	0.149	0.000	0.363
1.145	2.753	3.483	0.000	9.889	0.502	0.936	0.134	0.000	0.175	6.152	18.178	0.000	0.017	0.082	2.332
5.510	1.244	5.959	0.000	22.889	0.836	0.293	0.139	0.256	0.216	6.596	29.584	0.010	0.000	0.000	0.499
1.590	6.803	0.381	0.000	10.953	0.392	0.397	0.037	0.550	0.214	4.460	29.258	0.032	0.009	0.000	5.850
1.806	2.013	1.394	0.000	15.269	1.186	0.536	0.042	0.000	0.048	4.469	34.649	0.070	0.000	0.000	2.076
2.704	2.222	0.231	0.000	27.558	0.160	0.479	0.107	0.197	0.044	3.793	18.544	0.000	0.014	0.033	0.861
3.107	2.226	0.863	0.000	9.992	0.440	0.780	0.000	0.000	0.000	5.273	28.744	0.021	0.000	0.045	1.214
1.435	0.979	0.038	0.000	8.305	0.367	0.904	0.049	0.000	0.000	7.508	19.584	0.000	0.000	0.030	1.554
2.981	2.915	0.213	0.000	14.980	0.195	0.291	0.000	0.000	0.021	2.762	15.671	0.073	0.000	0.000	0.230
0.553	2.583	0.074	0.060	7.277	0.077	0.661	0.051	0.000	0.000	4.343	15.265	0.000	0.000	0.000	0.442
3.902	4.775	0.135	0.000	10.652	0.112	0.650	0.037	0.000	0.000	6.024	13.619	0.021	0.000	0.000	2.023
1.176	7.270	0.797	0.000	14.928	0.794	0.763	0.151	0.000	0.000	4.939	31.524	0.000	0.000	0.000	4.841
0.860	5.737	0.175	0.000	9.734	0.514	0.442	0.034	0.126	0.017	2.840	21.249	0.019	0.000	0.000	1.211
1.660	4.540	0.071	0.000	15.495	0.304	0.416	0.068	0.000	0.000	6.189	13.954	0.000	0.000	0.000	3.042
1.972	10.192	0.125	0.000	7.630	0.309	0.173	0.000	0.380	0.000	2.571	28.781	0.087	0.000	0.000	2.400
2.304	1.124	0.118	0.000	14.351	0.652	0.417	0.000	0.000	0.071	5.580	22.003	0.000	0.000	0.000	0.767
1.076	9.213	0.216	0.000	8.784	0.326	0.973	0.000	0.160	0.007	7.774	18.296	0.012	0.000	0.000	5.297
0.550	1.063	0.051	0.000	28.487	0.390	1.604	0.261	0.000	0.085	6.900	16.685	0.000	0.000	0.000	2.126
1.804	2.711	0.193	0.000	9.129	0.574	0.827	0.109	0.201	0.027	15.091	23.894	0.031	0.000	0.000	2.740
1.255	5.691	0.385	0.000	11.387	1.063	0.336	0.000	0.201	0.018	5.229	19.951	0.015	0.014	0.000	7.257
1.062	4.277	0.268	0.000	7.485	0.300	0.644	0.000	0.000	0.000	9.151	17.435	0.028	0.000	0.000	11.312
1.568	4.158	0.200	0.000	15.207	0.706	0.681	0.039	0.000	0.000	2.392	25.629	0.044	0.000	0.000	4.058
1.357	4.754	0.475	0.054	17.109	1.187	0.287	0.233	0.000	0.030	4.718	33.025	0.013	0.000	0.000	0.925
1.097	8.568	0.462	0.000	12.455	0.237	0.384	0.000	0.194	0.043	2.568	20.727	0.000	0.000	0.000	2.570
2.727	4.982	1.742	0.000	9.643	0.462	0.123	0.044	0.162	0.115	3.518	31.816	0.037	0.022	0.000	2.244
1.771	5.571	0.089	0.000	10.003	0.070	0.210	0.140	0.172	0.115	1.723	23.873	0.013	0.000	0.000	31.660
0.626	2.855	1.081	0.043	8.090	1.269	0.412	0.037	0.406	0.187	3.198	25.789	0.031	0.009	0.045	0.966
1.194	4.306	0.422	0.000	12.421	0.460	0.430	0.077	0.141	0.157	4.726	19.715	0.022	0.000	0.000	1.186
1.980	1.258	0.316	0.000	12.506	0.631	0.314	0.000	0.387	0.000	3.716	15.210	0.119	0.000	0.000	1.183
1.349	3.237	0.179	0.000	9.834	0.410	0.421	0.000	0.000	0.022	3.127	24.706	0.058	0.000	0.000	1.582
2.512	0.969	1.977	0.000	17.123	0.601	0.225	0.255	0.268	0.519	6.052	17.743	0.051	0.000	0.045	2.035
0.846	3.686	0.165	0.000	12.781	0.621	0.105	0.000	0.277	0.135	2.398	17.168	0.011	0.000	0.000	2.018
1.667	3.324	1.518	0.000	13.051	1.035	0.508	0.036	0.134	0.024	3.149	25.295	0.000	0.000	0.000	0.120
3.256	1.596	0.391	0.000	17.880	0.686	0.427	0.076	0.281	0.480	3.319	21.021	0.011	0.010	0.000	1.429
1.148	3.503	0.718	0.000	15.475	1.708	0.823	0.404	0.541	0.102	4.323	30.231	0.021	0.000	0.023	0.332
0.636	2.208	0.457	0.049	10.147	0.511	0.620	0.043	0.471	0.118	6.311	18.148	0.000	0.000	0.000	1.410
2.258	2.292	2.371	0.000	14.447	0.736	0.675	0.045	0.000	0.146	4.089	27.452	0.000	0.000	0.000	0.099
1.174	3.557	0.393	0.000	12.008	1.911	0.510	0.364	0.000	0.327	2.377	25.602	0.000	0.000	0.223	4.700
0.946	5.565	0.196	0.268	14.638	1.555	1.226	0.231	0.000	0.207	5.456	22.291	0.032	0.000	0.000	1.373
2.283	3.360	0.304	0.000	12.071	0.738	0.351	0.045	0.494	0.015	4.201	27.259	0.013	0.000	0.000	3.682
1.628	6.065	0.516	0.000	11.045	0.267	0.325	0.045	0.656	0.000	4.334	25.603	0.038	0.000	0.000	3.157
2.531	3.370	0.114	0.000	6.650	0.611	0.750	0.291	0.000	0.010	11.887	21.380	0.016	0.000	0.000	3.866
0.646	5.851	0.569	0.044	11.918	0.737	0.551	0.114	0.557	0.000	2.889	13.913	0.000	0.000	0.000	585.816
2.117	5.793	1.690	0.000	13.412	0.292	0.328	0.098	0.000	0.032	2.565	32.086	0.014	0.000	0.000	2.076
1.164	0.846	0.234	0.000	25.484	0.838	0.626	0.056	0.000	0.192	2.102	16.825	0.016	0.000	0.034	1.024
1.379	4.534	0.211	0.000	12.971	0.294	0.494	0.098	0.000	0.016	4.352	22.087	0.028	0.025	0.000	2.594
1.584	8.238	0.179	0.000	27.317	0.605	0.352	0.135	0.000	0.110	3.207	23.940	0.000	0.091	0.000	0.683
1.286	1.476	3.918	0.000	9.957	1.134	0.869	0.119	0.147	0.007	8.461	39.686	0.000	0.000	0.000	0.501
2.207	3.267	0.516	0.000	13.153	0.336	0.146	0.037	0.000	0.012	6.181	22.956	0.000	0.000	0.000	2.712
1.437	6.669	0.098	0.000	8.872	0.211	0.996	0.000	0.172	0.008	6.367	12.783	0.026	0.000	0.000	2.045
2.050	2.322	0.167	0.000	17.330	0.199	0.445	0.265	0.163	0.000	4.997	13.554	0.000	0.000	0.000	1.860
1.448	1.214	0.095	0.000	11.475	0.435	0.371	0.124	0.153	0.007	6.312	25.963	0.012	0.000	0.000	1.801
1.790	5.463	0.263	0.000	14.105	0.257	0.144	0.000	0.000	0.000	3.820	25.301	0.060	0.000	0.000	0.491
1.957	5.631	0.132	0.000	14.584	0.687	0.667	0.000	0.000	0.030	4.824	26.996	0.026	0.000	0.169	9.124
1.961	4.586	0.174	0.051	10.982	0.457	0.488	0.000	0.000	0.093	3.810	18.438	0.000	0.000	0.000	4.603
1.020	4.289	0.172	0.000	11.885	0.347	0.440	0.046	0.511	0.030	2.244	21.520	0.000	0.000	0.000	8.255
1.818	2.457	0.175	0.000	13.242	0.120	0.290	0.040	0.000	0.026	2.791	32.512	0.034	0.000	0.049	1.945
2.485	3.244	0.804	0.000	20.634	0.900	0.388	0.046	0.340	0.060	3.654	51.997	0.013	0.000	0.028	1.121
1.955	4.240	1.733	0.000	11.062	1.803	0.875	0.040	0.295	0.020	7.872	28.977	0.023	0.000	0.025	2.282
2.309	1.722	3.177	0.000	10.968	0.343	0.406	0.000	0.421	0.006	5.623	15.319	0.000	0.000	0.023	1.009
1.564	4.938	1.942	0.040	13.051	0.621	0.309	0.138	0.000	0.130	2.513	25.981	0.019	0.000	0.000	3.584
0.892	4.503	1.491	0.000	9.457	0.467	0.698	0.052	0.000	0.017	3.116	26.880	0.000	0.000	0.000	3.208
0.365	5.246	0.140	0.000	7.364	0.341	0.331	0.137	2.345	0.164	3.138	18.267	0.090	0.000	0.000	19.754
1.546	2.793	0.488	0.000	14.183	0.677	0.658	0.045	0.000	0.081	5.475	21.422	0.000	0.000	0.000	2.771
2.115	2.801	0.424	0.000	15.706	0.699	0.475	0.000	0.468	0.076	4.412	25.429	0.036	0.011	0.000	0.954
1.151	7.817	0.188	0.000	22.313	1.516	0.430	0.000	0.000	0.020	3.380	68.883	0.011	0.010	0.000	2.828
0.966	6.556	0.310	0.000	10.637	1.186	1.403	0.132	0.000	0.043	6.499	23.231	0.000	0.000	0.081	1.047
3.471	6.363	0.447	0.000	10.019	0.679	0.312	0.174	0.257	0.091	3.002	17.205	0.059	0.000	0.000	2.192
2.963	4.646	0.791	0.000	11.026	0.097	0.272	0.032	0.000	0.079	4.094	20.991	0.064	0.008	0.000	0.556
1.823	1.975	1.405	0.000	18.535	0.629	0.333	0.175	0.129	0.252	3.667	37.086	0.010	0.000	0.000	1.999
1.908	5.562	0.061	0.000	12.617	0.299	0.298	0.033	0.490	0.065	3.729	26.677	0.028	0.000	0.000	1.946
1.420	6.740	0.130	0.000	8.208	0.157	0.470	0.000	0.000	0.000	5.698	23.709	0.000	0.000	0.000	1.989
1.848	4.033	0.092	0.000	8.917	0.170	1.124	0.076	0.139	0.025	10.204	17.620	0.000	0.019	0.000	1.253
2.287	3.911	0.401	0.000	25.552	0.292	0.572	0.000	0.179	0.103	7.260	16.811	0.055	0.000	0.000	0.859
2.287	3.297	0.126	0.000	14.688	0.372	0.749	0.050	1.095	0.008	4.750	11.628	0.014	0.000	0.000	0.886
1.029	7.560	0.432	0.000	9.789	0.412	0.431	0.000	0.405	0.171	4.845	23.192	0.000	0.000	0.000	6.154
1.196	7.996	0.125	0.000	13.214	0.834	0.173	0.247	0.000	0.141	2.288	23.931	0.104	0.016	0.000	3.749
5.630	1.872	3.659	0.000	22.729	0.978	0.478	0.000	0.000	0.238	6.032	31.572	0.000	0.013	0.000	1.118
2.122	2.601	0.256	0.000	16.634	0.315	0.548	0.070	0.129	0.006	6.785	10.589	0.020	0.000	0.000	1.736
2.455	2.437	0.518	0.000	13.704	0.968	0.361	0.036	0.000	0.012	4.100	28.827	0.020	0.009	0.000	3.939
1.896	4.980	0.251	0.000	11.396	0.192	0.645	0.128	0.000	0.031	3.802	22.274	0.000	0.000	0.000	1.143
0.707	4.739	0.198	0.039	11.249	0.750	0.654	0.033	0.123	0.005	3.437	20.955	0.009	0.000	0.000	1.839
1.895	3.515	0.260	0.000	33.705	0.271	0.228	0.090	0.000	0.052	2.587	18.882	0.000	0.000	0.000	0.797
3.305	2.541	0.273	0.000	12.971	0.183	0.523	0.041	0.000	0.027	5.116	15.483	0.011	0.000	0.000	0.913
3.289	2.587	0.143	0.000	9.569	0.299	0.669	0.000	0.000	0.007	3.780	19.773	0.011	0.000	0.000	1.310

ENSG00000137434	ENSG00000143751	ENSG00000235208	ENSG00000107957	ENSG00000162526	ENSG00000254892	ENSG00000272040	ENSG00000230197	ENSG00000213252	ENSG00000131480	ENSG00000217377	ENSG00000266522	ENSG00000102239	ENSG00000265801	ENSG00000275006	ENSG00000234705
2.352	8.113	0.000	3.935	0.136	0.000	0.679	0.000	0.066	0.158	0.000	0.105	0.026	0.000	0.000	0.074
1.880	9.772	0.000	7.877	0.125	0.000	0.631	0.000	0.028	0.586	0.000	0.036	0.022	0.000	0.000	0.378
2.987	8.943	0.063	1.480	0.239	0.000	0.449	0.000	0.012	0.409	0.000	0.064	0.040	0.000	0.000	0.168
3.480	7.601	0.000	2.108	0.339	0.000	0.691	0.000	0.057	0.751	0.000	0.054	0.187	0.000	0.000	0.191
2.990	6.727	0.000	1.325	0.097	0.000	0.667	0.000	0.079	0.266	0.000	0.000	0.025	0.055	0.000	0.707
2.089	8.263	0.000	2.957	0.885	0.000	0.991	0.000	0.037	1.151	0.000	0.143	0.066	0.000	0.000	0.335
1.996	7.571	0.000	6.549	0.211	0.000	1.008	0.030	0.070	0.292	0.000	0.054	0.015	0.000	0.000	2.830
2.588	5.035	0.000	4.751	0.030	0.000	0.247	0.000	0.059	0.184	0.000	0.057	0.039	0.000	0.000	0.399
5.169	7.904	0.000	1.491	0.164	0.000	0.704	0.037	0.051	0.493	0.000	0.000	0.018	0.000	0.000	0.615
1.014	5.940	0.000	3.591	0.000	0.000	0.448	0.000	0.249	0.422	0.000	0.000	0.026	0.000	0.000	2.461
2.035	6.919	0.000	2.591	0.109	0.000	0.865	0.000	0.086	0.269	0.000	0.031	0.000	0.043	0.000	1.869
1.096	8.065	0.000	2.473	0.234	0.000	0.782	0.027	0.063	0.401	0.000	0.129	0.007	0.000	0.000	0.455
1.109	7.010	0.000	2.010	0.089	0.000	0.538	0.000	0.131	0.339	0.000	0.083	0.014	0.000	0.000	0.294
1.473	7.391	0.000	3.767	0.218	0.069	0.959	0.026	0.037	0.849	0.000	0.078	0.000	0.000	0.000	0.220
1.479	6.908	0.000	2.357	0.112	0.000	0.420	0.000	0.164	0.215	0.000	0.209	0.000	0.000	0.000	0.245
0.374	6.784	0.000	6.265	0.101	0.000	0.535	0.026	0.086	0.926	0.000	0.094	0.013	0.043	0.000	0.660
2.682	8.958	0.000	1.178	0.164	0.000	0.363	0.000	0.056	0.526	0.000	0.036	0.045	0.000	0.000	0.063
8.784	6.246	0.000	0.554	0.092	0.000	0.431	0.000	0.050	0.492	0.000	0.000	0.009	0.000	0.000	0.226
1.320	6.076	0.000	1.375	0.140	0.000	0.377	0.000	0.051	0.274	0.000	0.065	0.000	0.000	0.000	0.802
1.549	7.488	0.000	3.619	0.196	0.077	0.368	0.000	0.068	0.423	0.000	0.104	0.007	0.000	0.000	0.122
7.100	7.342	0.000	1.670	0.030	0.000	0.149	0.000	0.000	0.082	0.000	0.037	0.008	0.000	0.000	0.653
0.943	7.011	0.000	8.862	0.720	0.000	0.579	0.000	0.075	0.729	0.000	0.096	0.040	0.000	0.000	0.067
4.557	4.547	0.000	1.835	0.111	0.000	0.490	0.000	0.015	0.472	0.000	0.037	0.062	0.000	0.000	1.516
2.182	7.816	0.000	5.470	0.043	0.000	0.273	0.027	0.075	0.063	0.000	0.064	0.000	0.000	0.000	0.280
0.967	8.608	0.000	2.286	0.067	0.079	0.482	0.000	0.028	0.534	0.000	0.053	0.022	0.000	0.000	0.437
1.888	8.863	0.000	3.000	0.297	0.000	0.985	0.000	0.058	0.228	0.000	0.055	0.023	0.050	0.000	0.519
3.134	5.058	0.000	1.337	0.112	0.000	0.336	0.000	0.000	0.249	0.000	0.083	0.234	0.114	0.000	1.321
3.419	5.941	0.000	1.748	0.059	0.000	0.555	0.000	0.000	0.227	0.000	0.257	0.015	0.000	0.000	0.129
3.235	4.279	0.000	4.465	0.128	0.000	2.100	0.031	0.000	0.332	0.000	0.037	0.030	0.050	0.000	0.901
6.238	5.489	0.000	1.435	0.257	0.000	1.390	0.000	0.029	0.505	0.000	0.129	0.023	0.000	0.000	0.388
4.125	6.194	0.000	1.274	0.065	0.000	0.335	0.000	0.012	0.328	0.000	0.061	0.000	0.000	0.000	0.266
1.940	6.453	0.000	0.572	0.221	0.070	1.821	0.000	0.000	0.568	0.000	0.063	0.026	0.000	0.000	0.167
0.123	3.645	0.000	3.153	0.059	0.000	0.855	0.000	0.014	0.163	0.000	0.055	0.008	0.000	0.000	0.974
2.507	5.054	0.000	1.862	0.057	0.000	1.164	0.000	0.028	0.149	0.000	0.018	0.015	0.000	0.000	1.001
3.146	9.507	0.000	2.542	0.201	0.000	1.211	0.000	0.059	0.150	0.000	0.150	0.000	0.000	0.000	0.793
1.878	6.122	0.000	2.541	0.137	0.000	0.617	0.000	0.133	0.203	0.000	0.034	0.007	0.000	0.000	0.539
1.944	5.583	0.000	3.361	0.697	0.144	1.080	0.000	0.051	0.216	0.000	0.049	0.216	0.000	0.000	1.258
0.108	8.757	0.000	5.199	0.444	0.000	0.915	0.000	0.064	0.258	0.000	0.032	0.000	0.044	0.000	0.971
1.853	8.484	0.000	2.989	0.197	0.000	0.468	0.000	0.061	0.591	0.000	0.019	0.217	0.000	0.000	0.613
1.835	9.386	0.000	2.816	0.172	0.000	1.108	0.000	0.047	0.734	0.000	0.080	0.008	0.055	0.000	0.493
1.270	7.597	0.000	1.924	0.256	0.000	0.802	0.000	0.218	0.229	0.000	0.060	0.008	0.054	0.000	0.700
0.738	8.699	0.000	2.169	0.162	0.000	0.559	0.000	0.040	0.238	0.000	0.084	0.028	0.000	0.000	0.474
2.412	8.035	0.000	1.997	0.228	0.000	0.615	0.000	0.029	0.172	0.000	0.074	0.000	0.051	0.000	0.391
1.479	7.967	0.000	2.943	0.264	0.000	0.992	0.000	0.062	0.323	0.000	0.048	0.007	0.000	0.000	0.391
1.531	10.767	0.000	2.943	0.445	0.000	1.483	0.000	0.013	0.337	0.000	0.000	0.007	0.000	0.000	0.298
2.130	6.816	0.000	1.542	0.283	0.000	0.482	0.028	0.026	0.475	0.000	0.116	0.089	0.000	0.000	0.465
1.209	20.893	0.000	14.782	0.527	0.081	0.348	0.000	0.057	0.499	0.034	0.073	0.023	0.000	0.000	0.384
2.496	7.411	0.000	3.486	0.336	0.000	0.964	0.000	0.101	0.627	0.000	0.000	0.000	0.000	0.000	0.389
2.419	9.026	0.000	1.232	0.120	0.000	0.530	0.000	0.038	0.134	0.000	0.096	0.013	0.000	0.000	0.112
1.523	5.671	0.000	3.490	0.057	0.000	0.137	0.000	0.059	0.240	0.000	0.045	0.000	0.000	0.174	0.848
4.432	6.425	0.000	4.646	0.106	0.000	0.336	0.000	0.107	0.463	0.000	0.045	0.025	0.041	0.000	0.427
0.845	7.544	0.000	1.604	0.131	0.000	0.295	0.000	0.064	0.303	0.000	0.065	0.007	0.000	0.000	0.860
1.963	8.560	0.000	2.563	0.089	0.000	0.301	0.000	0.026	0.227	0.000	0.083	0.000	0.000	0.000	0.175
1.365	8.324	0.000	1.613	0.094	0.000	0.502	0.000	0.034	0.292	0.000	0.117	0.000	0.000	0.000	0.103
3.375	9.605	0.000	2.524	0.130	0.000	0.789	0.000	0.148	0.621	0.000	0.054	0.045	0.000	0.000	0.000
1.870	12.021	0.000	4.381	0.226	0.000	0.921	0.000	0.276	0.591	0.000	0.281	0.204	0.000	0.810	0.000
1.929	11.889	0.000	3.351	0.047	0.000	0.757	0.000	0.041	0.673	0.000	0.087	0.007	0.000	0.000	0.000
2.723	8.718	0.000	1.756	0.284	0.000	0.715	0.000	0.091	0.658	0.000	0.050	0.021	0.000	0.000	0.233
3.972	8.493	0.000	5.791	0.158	0.000	0.430	0.000	0.103	0.428	0.000	0.148	0.048	0.000	0.000	0.462
2.036	8.666	0.000	1.413	0.115	0.000	0.509	0.000	0.060	0.183	0.000	0.077	0.108	0.000	0.000	0.108
2.094	10.911	0.000	4.211	0.465	0.078	0.606	0.000	0.042	0.720	0.033	0.212	0.037	0.000	0.000	0.374
5.003	8.352	0.000	1.415	0.181	0.000	0.622	0.000	0.044	0.621	0.000	0.169	0.000	0.000	0.000	0.099
1.808	8.649	0.000	2.815	0.298	0.000	0.883	0.025	0.023	1.119	0.000	0.102	0.467	0.000	0.000	0.206
1.626	11.383	0.000	3.268	0.513	0.070	0.931	0.000	0.037	0.381	0.000	0.125	0.026	0.000	0.000	0.662
2.369	8.369	0.000	7.322	0.122	0.000	0.528	0.000	0.041	0.131	0.000	0.035	0.268	0.000	0.000	0.184
2.537	5.402	0.000	2.309	0.108	0.000	0.452	0.000	0.053	0.397	0.000	0.000	0.047	0.000	0.000	0.000
3.479	9.322	0.000	0.880	0.149	0.000	0.310	0.000	0.048	0.095	0.000	0.123	0.000	0.000	0.000	0.108
2.101	7.532	0.000	0.704	0.044	0.000	0.579	0.000	0.080	0.454	0.000	0.041	0.009	0.056	0.000	0.144
0.943	11.256	0.000	1.509	0.117	0.000	0.550	0.000	0.021	0.072	0.000	0.109	0.011	0.000	0.000	0.288
5.732	7.406	0.000	1.142	0.023	0.000	0.239	0.000	0.118	0.057	0.000	0.172	0.000	0.000	0.000	0.076
5.765	10.095	0.000	1.213	0.034	0.000	0.411	0.000	0.025	0.181	0.000	0.063	0.007	0.000	0.000	0.055
1.309	4.498	0.000	1.295	0.102	0.000	0.320	0.000	0.025	0.112	0.000	0.032	0.040	0.000	0.000	0.112
0.612	5.482	0.000	1.576	0.108	0.064	0.507	0.000	0.034	0.140	0.000	0.043	0.018	0.000	0.000	1.013
2.980	5.880	0.000	0.913	0.076	0.000	0.686	0.000	0.067	0.264	0.000	0.028	0.035	0.000	0.000	0.200
0.345	7.799	0.000	2.187	0.116	0.000	0.719	0.000	0.085	0.766	0.000	0.065	0.000	0.000	0.000	0.152
1.803	5.728	0.000	12.471	0.210	0.347	0.867	0.000	0.020	0.277	0.000	0.052	0.000	0.071	0.000	0.092
3.247	4.489	0.000	0.988	0.098	0.000	0.294	0.000	0.014	0.113	0.000	0.036	0.000	0.000	0.000	0.000
1.017	15.868	0.000	5.464	0.000	0.000	0.550	0.000	0.086	0.532	0.000	0.109	0.045	0.000	0.000	0.769
5.403	8.730	0.000	0.643	0.099	0.000	0.902	0.000	0.036	0.223	0.000	0.000	0.029	0.000	0.000	0.242
1.948	7.514	0.000	0.414	0.061	0.000	0.415	0.000	0.144	0.142	0.000	0.000	0.019	0.000	0.000	0.403
6.169	7.021	0.000	0.376	0.045	0.000	0.233	0.000	0.049	0.241	0.000	0.000	0.009	0.000	0.000	0.665
2.976	6.561	0.000	1.219	0.088	0.000	0.249	0.000	0.026	0.197	0.000	0.049	0.027	0.045	0.000	0.405
1.845	5.629	0.000	3.273	0.084	0.000	1.004	0.000	0.000	0.899	0.000	0.020	0.008	0.000	0.000	0.550
1.147	5.992	0.000	3.568	0.202	0.000	0.490	0.000	0.017	0.440	0.000	0.089	0.000	0.000	0.000	0.701
1.008	4.766	0.000	7.235	0.139	0.000	0.652	0.000	0.000	0.384	0.000	0.129	0.146	0.051	0.000	0.455
0.992	7.224	0.000	2.851	0.042	0.000	0.752	0.000	0.031	0.287	0.000	0.039	0.000	0.000	0.000	0.346
1.869	8.755	0.000	3.380	0.249	0.000	1.291	0.000	0.036	0.594	0.000	0.093	0.013	0.042	0.000	0.000
5.183	7.355	0.000	5.884	0.138	0.000	0.600	0.054	0.088	0.242	0.000	0.145	0.020	0.000	0.000	0.680
1.996	5.622	0.000	1.412	0.134	0.000	0.504	0.000	0.012	0.495	0.000	0.103	0.006	0.000	0.000	0.155
0.838	7.255	0.000	2.476	0.277	0.000	1.010	0.000	0.045	0.634	0.000	0.029	0.012	0.000	0.000	0.706
2.340	5.724	0.000	1.418	0.263	0.068	2.250	0.000	0.048	0.995	0.000	0.031	0.038	0.084	0.000	0.377
1.533	8.703	0.000	0.704	0.229	0.000	0.668	0.000	0.025	0.657	0.000	0.142	0.033	0.043	0.000	0.723
1.318	9.016	0.000	1.474	0.155	0.000	0.630	0.000	0.072	1.416	0.000	0.092	0.025	0.000	0.000	0.268
2.743	9.781	0.000	2.247	0.635	0.000	0.773	0.000	0.038	0.290	0.000	0.096	0.013	0.087	0.000	0.732
1.497	6.351	0.000	2.709	0.323	0.000	0.776	0.026	0.097	0.675	0.057	0.031	0.013	0.042	0.000	0.271
3.425	8.288	0.000	3.480	0.317	0.000	0.991	0.000	0.000	0.546	0.000	0.072	0.037	0.000	0.000	0.378
2.517	6.783	0.000	0.989	0.181	0.000	0.245	0.000	0.015	0.713	0.000	0.206	0.016	0.051	0.000	0.198
1.118	7.299	0.000	2.086	0.205	0.000	1.155	0.000	0.000	0.169	0.000	0.076	0.000	0.209	0.000	0.269
5.210	6.308	0.000	1.814	0.259	0.000	1.510	0.000	0.114	0.300	0.000	0.000	0.060	0.000	0.000	3.915
1.473	8.550	0.000	2.311	0.111	0.000	0.964	0.000	0.059	0.407	0.000	0.094	0.016	0.000	0.000	0.396
1.542	7.679	0.000	2.247	0.522	0.000	0.792	0.000	0.073	0.654	0.000	0.056	0.644	0.000	0.000	0.329
3.964	6.539	0.000	0.347	0.026	0.000	0.788	0.000	0.038	0.086	0.000	0.000	0.000	0.000	0.000	1.290
3.905	4.197	0.000	3.482	0.409	0.141	1.567	0.000	0.037	0.162	0.000	0.079	0.040	0.000	0.000	1.844
5.333	6.916	0.000	3.100	0.132	0.000	0.433	0.000	0.080	0.181	0.000	0.082	0.025	0.000	0.000	1.368
11.076	6.182	0.000	1.326	0.189	0.104	1.560	0.000	0.055	0.540	0.000	0.047	0.049	0.064	0.000	0.743
2.351	7.404	0.000	2.384	0.176	0.000	0.497	0.000	0.000	0.218	0.000	0.082	0.000	0.000	0.000	0.000
7.458	6.495	0.000	1.419	0.111	0.000	0.664	0.000	0.015	0.300	0.000	0.000	0.172	0.000	0.000	0.927
2.577	8.171	0.000	1.500	0.063	0.000	0.118	0.000	0.079	0.399	0.000	0.050	0.021	0.000	0.000	0.176
2.043	8.486	0.000	0.795	0.093	0.000	0.537	0.000	0.049	0.118	0.000	0.063	0.013	0.000	0.000	0.166
2.482	2.661	0.000	1.536	0.042	0.000	0.218	0.000	0.031	0.356	0.000	0.020	0.008	0.000	0.000	0.277
5.912	4.219	0.000	0.174	0.030	0.000	0.187	0.000	0.029	0.033	0.000	0.019	0.008	0.000	0.000	0.130
2.150	7.970	0.000	4.407	0.065	0.000	0.578	0.000	0.014	0.200	0.000	0.052	0.000	0.000	0.200	0.122
1.027	6.354	0.000	2.883	0.135	0.000	0.579	0.000	0.042	0.183	0.000	0.054	0.007	0.000	0.000	0.379
1.433	7.806	0.000	5.241	0.165	0.000	0.891	0.000	0.091	0.323	0.000	0.115	0.016	0.000	0.000	0.542
3.816	11.065	0.000	3.069	0.226	0.000	0.368	0.000	0.057	0.283	0.000	0.110	0.030	0.000	0.000	0.322
1.084	6.848	0.000	1.634	0.052	0.000	0.508	0.000	0.030	0.438	0.000	0.000	0.000	0.000	0.000	0.273
0.757	8.432	0.000	3.136	0.153	0.074	0.658	0.000	0.092	0.281	0.000	0.117	0.007	0.000	0.000	0.000
1.856	7.848	0.000	2.811	0.177	0.000	0.625	0.065	0.061	0.146	0.000	0.116	0.016	0.000	0.000	0.136
2.304	10.919	0.000	1.275	0.045	0.000	0.373	0.028	0.040	0.112	0.000	0.084	0.014	0.000	0.000	0.296
1.000	9.861	0.000	3.995	0.069	0.000	0.500	0.000	0.088	0.163	0.000	0.144	0.000	0.000	0.000	0.113
1.848	9.391	0.000	3.544	0.078	0.064	0.248	0.000	0.057	0.301	0.000	0.101	0.030	0.000	0.000	0.153
2.086	9.628	0.000	1.948	0.129	0.000	0.439	0.000	0.034	0.174	0.000	0.044	0.000	0.000	0.000	0.230
0.711	9.559	0.000	20.199	0.082	0.000	0.596	0.000	0.030	0.152	0.000	0.096	0.008	0.000	0.000	0.202
4.387	7.989	0.000	1.956	0.153	0.000	0.363	0.000	0.119	0.059	0.000	0.076	0.000	0.000	0.000	0.067
4.260	6.978	0.000	2.073	0.344	0.000	0.609	0.000	0.014	0.882	0.000	0.071	0.044	0.000	0.000	0.313
1.580	4.467	0.000	5.692	0.146	0.075	0.393	0.000	0.133	0.285	0.000	0.085	0.014	0.000	0.000	0.538
2.060	6.774	0.000	2.174	0.148	0.000	0.502	0.000	0.000	0.367	0.000	0.111	0.000	0.000	0.000	0.389
4.571	9.201	0.000	3.463	0.353	0.000	0.781	0.000	0.046	0.550	0.000	0.088	0.018	0.000	0.000	0.412
3.265	6.319	0.000	2.067	0.124	0.000	0.342	0.000	0.032	0.360	0.000	0.041	0.039	0.000	0.000	0.382
3.475	9.863	0.000	3.881	0.276	0.000	0.503	0.000	0.081	0.520	0.000	0.073	3.076	0.040	0.000	0.310
0.966	8.695	0.000	1.669	0.082	0.000	0.478	0.023	0.044	0.334	0.000	0.168	0.012	0.000	0.000	0.147
2.164	7.311	0.000	2.155	0.071	0.000	0.222	0.000	0.069	0.272	0.000	0.088	0.000	0.120	0.000	0.000
3.593	11.829	0.000	2.241	0.102	0.000	0.144	0.000	0.199	0.176	0.000	0.064	0.000	0.043	0.000	0.056
4.859	6.641	0.000	4.144	0.197	0.000	0.720	0.000	0.080	1.255	0.000	0.102	0.246	0.056	0.000	0.287
1.300	6.328	0.000	1.551	0.179	0.000	0.775	0.000	0.033	0.662	0.000	0.042	0.000	0.000	0.000	0.293
2.518	7.613	0.000	2.814	0.198	0.000	0.558	0.000	0.000	0.807	0.000	0.069	0.029	0.000	0.000	0.244
1.621	7.730	0.000	4.639	0.306	0.000	0.967	0.000	0.081	0.172	0.000	0.104	0.000	0.000	0.000	0.730
2.072	7.841	0.000	2.931	0.169	0.000	1.124	0.000	0.050	0.792	0.000	0.126	0.052	0.058	0.000	0.148
4.975	9.520	0.000	2.692	0.157	0.000	0.739	0.025	0.127	0.377	0.000	0.147	0.006	0.000	0.000	0.310
3.261	6.932	0.000	3.523	0.121	0.000	0.515	0.000	0.083	0.153	0.000	0.030	0.006	0.000	0.173	0.212
2.785	7.186	0.000	3.509	0.101	0.000	0.406	0.000	0.042	0.249	0.000	0.161	0.000	0.000	0.000	0.095
1.712	7.184	0.055	1.804	0.105	0.000	0.352	0.000	0.022	0.303	0.000	0.056	0.000	0.000	0.000	0.443
3.705	7.280	0.000	2.030	0.315	0.000	0.401	0.032	0.045	0.193	0.000	0.095	0.134	0.000	0.000	0.400
2.854	8.804	0.000	1.846	0.356	0.000	1.149	0.000	0.080	0.844	0.000	0.068	0.000	0.000	0.000	0.180
3.068	12.524	0.000	2.964	0.161	0.000	0.371	0.000	0.039	0.200	0.000	0.117	0.000	0.046	0.000	0.059

ENSG00000237094	ENSG00000002587	ENSG00000267592	ENSG00000251209	ENSG00000125124	ENSG00000164542	ENSG00000150687	ENSG00000219773	ENSG00000224781	ENSG00000248120	ENSG00000085741	ENSG00000258740	ENSG00000233487	ENSG00000256969	ENSG00000277215	ENSG00000269397
0.263	2.646	0.110	0.016	10.108	1.832	14.204	0.396	0.455	0.000	0.031	0.000	0.250	0.042	0.103	0.544
0.009	1.054	0.187	0.009	5.411	1.561	12.322	0.530	0.510	0.000	0.351	0.000	0.425	0.000	0.100	0.295
0.004	2.161	0.000	0.000	7.463	1.328	7.123	0.643	0.423	0.000	0.281	0.000	0.378	0.000	0.033	1.960
0.049	1.473	0.377	0.009	12.585	2.737	1.842	0.316	0.177	0.082	2.209	0.000	0.178	0.036	0.013	1.146
0.079	1.908	0.000	0.000	7.635	2.999	10.515	0.189	0.849	0.000	0.029	0.000	0.119	0.000	0.084	0.315
0.144	5.948	0.166	0.008	21.833	1.483	1.413	0.320	0.328	0.000	0.816	0.000	0.157	0.032	0.033	3.407
0.079	1.304	0.000	0.009	7.089	1.152	4.605	0.577	0.263	0.000	0.236	0.000	0.194	0.036	0.087	0.532
0.019	2.998	0.000	0.014	6.939	1.905	4.170	0.381	0.650	0.086	0.065	0.000	0.673	0.000	0.053	0.207
0.048	6.928	0.000	0.000	10.438	1.808	5.574	0.617	0.365	0.000	0.139	0.000	0.475	0.087	0.015	1.643
0.078	2.983	0.000	0.000	4.822	0.533	13.880	0.684	0.812	0.288	0.124	0.000	0.440	0.000	0.044	0.349
0.065	1.178	0.000	0.008	13.347	1.416	5.803	0.273	0.215	0.000	0.031	0.000	0.170	0.031	0.142	0.465
0.059	1.343	0.084	0.016	10.160	1.805	5.666	0.261	0.413	0.073	0.150	0.028	0.352	0.000	0.034	0.988
0.033	2.865	0.000	0.008	10.669	2.040	1.843	0.247	0.492	0.000	0.253	0.000	0.198	0.033	0.047	2.170
0.027	7.229	0.326	0.012	11.067	1.758	5.625	0.399	0.552	0.000	0.176	0.000	0.123	0.000	0.000	0.758
0.068	3.463	0.363	0.000	7.205	1.773	4.349	1.216	1.025	0.000	0.068	0.000	0.137	0.000	0.000	0.272
0.069	1.733	0.163	0.020	8.861	0.988	11.899	0.273	0.307	0.000	0.168	0.000	0.062	0.062	0.044	0.147
0.027	4.262	0.000	0.009	8.847	3.055	5.444	0.606	0.496	0.082	0.185	0.000	0.249	0.036	0.013	1.524
0.016	9.962	0.112	0.016	8.841	1.057	3.925	1.754	0.735	0.000	0.167	0.000	0.782	0.000	0.045	1.625
0.052	2.126	0.000	0.012	22.516	1.893	5.464	0.394	0.336	0.074	0.096	0.000	0.370	0.000	0.000	0.331
0.064	2.854	0.000	0.004	11.712	0.839	4.650	0.211	0.376	0.000	0.162	0.000	0.155	0.000	0.073	0.109
0.018	4.324	0.000	0.038	6.376	1.759	2.812	1.172	0.674	0.000	0.045	0.000	0.568	0.000	0.103	0.872
0.127	1.244	0.701	0.015	8.056	1.708	2.892	0.309	0.377	0.000	0.131	0.000	0.985	0.000	0.053	1.111
0.032	1.423	0.000	0.009	6.081	2.493	2.764	0.504	0.350	0.000	0.587	0.000	0.500	0.075	0.026	1.820
0.012	0.927	0.000	0.004	8.662	1.470	27.944	0.557	0.235	0.000	0.039	0.000	0.221	0.000	0.144	0.387
0.100	4.435	0.093	0.000	7.480	1.212	8.369	0.262	0.209	0.000	0.174	0.000	0.193	0.000	0.025	0.403
0.054	4.495	0.193	0.009	9.024	2.130	4.612	0.273	0.091	0.000	0.190	0.000	0.164	0.037	0.064	1.026
0.031	4.207	0.327	0.011	17.094	2.814	4.492	0.617	0.615	0.000	0.061	0.000	0.247	0.000	0.073	0.474
0.045	2.749	0.192	0.000	9.063	1.399	5.148	0.692	0.469	0.000	0.126	0.000	0.581	0.000	0.000	1.180
0.085	5.140	0.000	0.000	12.192	1.630	2.764	0.369	0.270	0.000	0.215	0.000	0.199	0.000	0.051	0.874
0.032	3.843	0.000	0.014	8.293	2.170	2.582	0.792	0.380	0.083	0.243	0.000	0.491	0.000	0.038	0.879
0.041	7.160	0.158	0.000	16.109	1.727	4.124	0.672	0.238	0.000	0.133	0.000	0.224	0.030	0.200	1.127
0.354	4.728	0.166	0.016	17.709	1.502	8.984	0.171	0.421	0.000	0.008	0.055	0.188	0.000	0.111	1.069
0.009	9.681	0.000	0.037	8.897	1.541	1.178	0.248	0.399	0.000	0.163	0.000	0.456	0.000	0.039	0.260
0.044	3.776	0.000	0.009	10.827	1.494	9.502	0.359	0.245	0.000	0.165	0.000	0.141	0.036	0.050	0.613
0.111	1.725	0.785	0.019	12.704	2.251	4.620	0.354	0.738	0.000	0.037	0.000	0.334	0.000	0.131	0.677
0.088	0.762	0.089	0.009	12.720	2.048	3.571	0.275	0.435	0.000	0.033	0.000	0.252	0.068	0.059	0.360
0.327	4.105	0.085	0.016	21.111	4.976	0.743	0.197	0.271	0.000	0.135	0.000	0.161	0.162	0.057	0.458
0.020	2.217	0.085	0.004	8.708	0.927	1.269	0.218	0.383	0.000	0.286	0.000	0.064	0.000	0.057	1.577
0.047	2.745	0.101	0.015	9.619	2.246	4.070	0.365	0.609	0.000	0.190	0.000	0.134	0.000	0.040	1.092
0.172	3.213	0.209	0.005	16.677	3.561	5.113	0.296	0.452	0.000	0.333	0.000	0.138	0.000	0.000	1.505
0.117	3.612	0.000	0.030	15.188	1.912	4.014	0.294	0.391	0.000	0.039	0.000	0.275	0.040	0.153	0.047
0.054	0.567	0.264	0.000	9.528	2.665	15.553	0.249	0.248	0.000	0.099	0.000	0.083	0.000	0.082	0.461
0.100	0.362	0.097	0.005	15.683	2.380	1.906	0.448	0.673	0.000	0.118	0.000	0.274	0.037	0.077	0.957
0.136	0.736	0.000	0.000	18.809	3.430	3.442	0.128	0.453	0.000	0.054	0.000	0.188	0.000	0.022	0.635
0.274	3.331	0.177	0.013	14.163	1.911	2.143	0.387	0.216	0.000	0.257	0.000	0.134	0.034	0.142	0.822
0.065	2.983	0.086	0.000	12.880	2.660	3.514	0.266	0.325	0.000	0.137	0.000	0.065	0.033	0.069	0.789
0.009	0.429	0.000	0.009	3.040	0.159	4.008	0.538	0.411	0.330	8.572	0.000	0.252	0.000	0.038	0.883
0.212	4.635	0.096	0.005	10.416	0.569	5.163	0.470	0.289	0.000	0.162	0.000	0.164	0.000	0.038	1.600
0.039	4.823	0.083	0.000	16.404	2.843	2.639	0.579	0.768	0.000	0.102	0.000	0.189	0.000	0.000	1.288
0.011	2.492	0.000	0.015	6.901	0.766	9.296	0.466	0.563	0.000	0.155	0.000	0.357	0.060	0.084	0.248
0.033	1.165	0.000	0.027	10.427	3.796	5.098	0.611	1.281	0.000	0.185	0.000	0.899	0.000	0.032	0.641
0.016	1.222	0.255	0.012	13.184	1.355	7.152	0.482	0.592	0.000	0.064	0.000	0.306	0.000	0.023	0.409
0.004	4.735	0.087	0.029	13.397	3.229	9.725	1.026	0.734	0.000	0.138	0.029	0.180	0.033	0.139	0.949
0.025	2.653	0.000	0.004	14.875	2.149	3.954	0.355	0.490	0.133	0.072	0.000	0.116	0.000	0.000	0.057
0.020	2.651	0.000	0.014	9.418	3.293	10.048	0.871	0.663	0.000	0.251	0.000	0.294	0.054	0.169	0.931
0.035	3.898	0.000	0.000	18.562	0.627	2.714	0.378	1.175	0.319	0.103	0.000	0.417	0.000	0.098	1.378
0.103	0.783	0.365	0.009	7.445	3.478	8.650	0.611	0.601	0.000	0.120	0.000	0.294	0.000	0.061	0.575
0.073	4.075	0.518	0.000	20.838	1.913	5.122	0.666	0.666	0.000	0.089	0.000	0.261	0.132	0.058	0.401
0.101	0.534	0.171	0.398	11.449	3.484	12.502	0.331	0.306	0.000	0.169	0.028	0.081	0.033	0.000	0.527
0.015	2.980	0.080	0.008	16.795	4.499	2.806	0.309	0.587	0.000	0.105	0.027	0.151	0.031	0.128	0.384
0.169	3.451	0.092	0.004	17.240	3.388	2.727	0.143	0.417	0.000	0.095	0.000	0.227	0.000	0.111	0.305
0.048	3.957	0.000	0.000	9.445	1.825	2.809	0.944	0.442	0.000	0.137	0.000	0.611	0.056	0.000	2.156
0.154	2.626	0.840	0.004	8.651	4.878	2.579	0.236	0.618	0.000	0.372	0.000	0.159	0.000	0.031	2.325
0.150	2.470	0.409	0.004	13.604	2.804	4.409	0.169	0.401	0.000	0.153	0.000	0.201	0.000	0.153	0.676
0.073	6.096	0.000	0.009	14.143	2.887	5.471	0.376	0.343	0.079	0.274	0.000	0.242	0.000	0.037	1.287
0.006	2.187	0.234	0.011	7.541	2.470	4.547	0.543	0.772	0.102	0.396	0.000	0.333	0.000	0.110	1.847
0.023	2.809	0.161	0.008	11.337	2.748	1.778	1.076	0.666	0.000	0.422	0.000	0.517	0.062	0.064	0.362
0.025	1.134	0.214	0.005	14.958	1.976	1.529	0.939	0.625	0.000	0.241	0.000	0.467	0.000	0.000	0.804
0.007	3.684	0.000	0.014	15.047	2.527	0.654	0.257	0.697	0.000	0.053	0.000	0.054	0.000	0.038	0.235
0.011	0.379	0.113	0.000	8.214	1.258	2.945	0.667	0.572	0.000	0.032	0.000	0.192	0.000	0.135	0.557
0.035	0.776	0.164	0.004	15.425	3.738	8.853	0.824	0.463	0.000	0.208	0.027	0.295	0.000	0.000	0.345
0.008	3.232	0.000	0.040	11.730	4.694	7.694	0.470	1.030	0.000	0.466	0.000	0.220	0.127	0.244	0.124
0.004	1.115	0.226	0.000	14.368	1.552	16.572	0.581	0.622	0.000	0.085	0.000	0.171	0.000	0.120	0.293
0.028	0.527	0.296	0.000	10.158	2.180	4.169	0.267	0.251	0.000	0.042	0.049	0.084	0.000	0.000	1.489
0.058	1.585	0.113	0.000	9.725	1.818	7.754	0.262	0.425	0.000	0.085	0.000	0.171	0.000	0.030	0.475
0.064	0.882	0.000	0.000	13.589	2.405	1.087	0.316	0.282	0.000	0.089	0.000	0.052	0.000	0.000	1.328
0.018	5.456	0.000	0.000	8.247	1.570	3.369	0.809	0.537	0.000	0.143	0.000	0.288	0.073	0.038	0.243
0.751	0.425	0.000	0.111	3.452	0.764	1.972	0.588	1.289	0.000	0.268	0.000	0.648	0.000	0.152	1.798
0.011	3.549	0.240	0.012	14.818	3.068	5.761	0.618	1.106	0.208	0.337	0.000	0.477	0.000	0.160	0.486
0.062	3.859	0.000	0.000	10.948	4.171	12.563	0.493	0.405	0.104	0.011	0.000	0.249	0.000	0.016	0.431
0.005	9.875	0.000	0.117	12.706	2.688	12.154	0.791	1.424	0.286	0.175	0.000	0.166	0.000	0.059	1.497
0.012	3.307	0.344	0.017	11.113	4.980	3.630	0.598	0.485	0.075	0.032	0.029	0.260	0.033	0.034	0.168
0.034	2.979	0.000	0.010	8.989	1.311	3.821	0.158	0.173	0.000	0.201	0.034	0.193	0.391	0.041	1.470
0.049	4.254	0.578	0.006	17.417	1.662	5.968	0.268	0.370	0.000	0.097	0.000	0.153	0.044	0.031	0.312
0.032	0.910	0.000	0.009	9.128	1.895	6.943	0.199	0.363	0.084	0.335	0.000	0.110	0.000	0.052	0.782
0.029	1.331	0.000	0.000	5.341	1.811	20.726	0.106	0.154	0.178	0.048	0.000	0.097	0.000	0.055	0.431
0.235	0.273	0.161	0.000	15.301	1.808	2.146	0.270	0.273	0.000	0.106	0.027	0.168	0.000	0.022	2.554
0.020	0.575	0.084	0.000	10.000	3.822	7.519	0.238	0.348	0.000	0.032	0.028	0.350	0.000	0.135	0.164
0.018	0.671	0.154	0.004	19.030	2.149	5.365	0.396	0.231	0.000	0.086	0.026	0.087	0.000	0.031	1.430
0.105	0.644	0.150	0.000	28.772	1.172	12.390	0.424	0.563	0.000	0.295	0.000	0.170	0.057	0.080	0.809
0.158	2.051	0.080	0.023	18.886	2.609	1.660	0.185	0.391	0.000	0.232	0.000	0.333	0.122	0.000	5.972
0.078	7.347	0.248	0.000	14.239	1.405	9.252	0.064	0.171	0.000	0.039	0.000	0.094	0.158	0.088	0.569
0.034	4.538	0.080	0.000	13.145	1.137	3.633	0.554	0.449	0.069	0.052	0.000	0.181	0.000	0.064	1.780
0.071	3.449	0.167	0.008	22.981	3.834	1.095	0.624	0.393	0.000	0.180	0.000	0.364	0.000	0.056	1.166
0.193	1.210	0.081	0.019	21.104	0.981	6.435	0.705	1.015	0.070	0.468	0.000	0.473	0.000	0.021	1.063
0.175	1.063	0.000	0.005	24.255	2.778	5.537	0.457	0.281	0.000	0.263	0.000	0.124	0.072	0.025	1.191
0.051	8.238	0.098	0.000	11.702	1.375	4.481	0.832	0.479	0.000	0.303	0.000	0.296	0.000	0.118	0.617
0.169	3.509	0.000	0.019	7.175	2.069	4.083	0.308	0.225	0.000	0.337	0.000	0.076	0.000	0.107	2.395
0.166	2.416	0.000	0.008	18.154	4.445	5.572	0.520	1.141	0.000	0.071	0.000	0.191	0.000	0.000	0.720
0.055	3.830	0.000	0.000	13.578	2.792	5.956	0.505	0.517	0.000	0.175	0.000	0.260	0.038	0.065	1.015
0.119	5.723	0.098	0.005	8.048	2.733	8.079	0.352	0.129	0.085	0.055	0.000	0.148	0.000	0.104	0.924
0.024	1.913	0.000	0.000	12.147	3.453	5.756	0.822	0.672	0.111	0.215	0.085	0.411	0.049	0.068	0.823
0.062	1.883	0.166	0.016	8.447	6.467	5.070	0.256	0.296	0.000	0.225	0.000	0.173	0.000	0.077	0.522
0.085	9.104	0.000	0.026	11.182	4.208	7.038	0.275	0.422	0.000	0.230	0.000	0.142	0.164	0.071	0.305
0.081	2.243	0.000	0.000	20.557	1.754	1.075	0.189	0.277	0.000	0.046	0.041	0.070	0.000	0.065	2.114
0.081	2.545	0.000	0.000	10.197	0.934	2.716	0.829	0.525	0.000	0.040	0.000	0.325	0.082	0.000	0.548
0.042	0.829	0.000	0.010	9.824	3.213	4.236	0.253	0.129	0.000	0.065	0.000	0.112	0.000	0.079	0.251
0.045	6.278	0.087	0.000	10.867	1.170	6.711	0.741	0.197	0.000	0.155	0.000	0.314	0.033	0.128	0.982
0.042	3.646	0.000	0.004	11.090	2.260	4.578	0.232	0.170	0.000	0.131	0.000	0.093	0.000	0.098	0.909
0.019	3.716	0.000	0.005	5.031	1.922	12.766	0.687	0.386	0.000	0.154	0.000	0.253	0.000	0.000	0.955
0.000	0.232	0.000	0.000	17.180	2.253	4.851	0.349	0.219	0.000	0.018	0.000	0.110	0.000	0.077	0.334
0.013	2.583	0.000	0.000	14.785	1.814	7.760	0.187	0.205	0.158	0.213	0.000	0.052	0.000	0.012	1.254
0.053	1.854	0.000	0.032	9.139	1.331	1.392	0.193	0.159	0.000	0.053	0.000	0.178	0.036	0.000	0.521
0.047	1.933	0.201	0.010	9.772	1.284	6.578	0.828	0.870	0.000	0.245	0.000	0.609	0.077	0.000	1.146
0.031	3.439	0.191	0.000	22.515	3.146	6.154	0.615	0.323	0.000	0.045	0.000	0.181	0.000	0.064	0.215
0.019	1.084	0.000	0.015	11.968	2.082	14.794	0.365	0.286	0.000	0.076	0.000	0.269	0.000	0.014	0.122
0.053	5.958	0.087	0.013	13.598	1.305	2.924	0.248	0.411	0.000	0.172	0.000	0.348	0.000	0.058	0.932
0.014	2.729	0.203	0.010	19.148	3.445	2.993	0.756	1.086	0.000	0.228	0.000	0.307	0.000	0.041	0.517
0.017	3.865	0.176	0.013	12.070	3.159	3.427	0.769	0.612	0.000	0.239	0.000	0.316	0.000	0.059	1.252
0.043	6.728	0.334	0.000	10.048	1.233	4.029	0.581	0.582	0.073	0.071	0.000	0.174	0.000	0.011	0.138
0.036	3.532	0.227	0.000	8.526	1.947	10.085	0.526	0.583	0.066	0.234	0.000	0.315	0.000	0.040	0.726
0.112	6.133	0.000	0.011	8.594	1.481	10.345	0.674	0.600	0.000	0.181	0.000	0.366	0.000	0.061	0.444
0.009	0.503	0.000	0.010	4.208	1.054	3.559	0.539	0.263	0.000	0.150	0.000	0.151	0.000	0.027	0.284
0.028	5.003	0.198	0.005	16.796	3.081	0.739	0.408	0.727	0.000	0.037	0.000	0.131	0.190	0.119	0.074
0.096	0.876	0.093	0.027	18.988	1.704	1.042	0.096	0.402	0.000	0.061	0.000	0.246	0.000	0.012	0.404
0.025	2.477	0.000	0.034	9.830	1.687	4.631	0.434	1.051	0.000	0.058	0.000	1.023	0.034	0.118	0.306
0.095	2.923	0.000	0.028	12.700	1.556	6.740	0.967	1.305	0.000	0.054	0.000	0.492	0.000	0.000	0.564
0.165	8.481	0.153	0.022	13.969	3.141	4.156	0.275	0.345	0.000	0.043	0.025	0.145	0.000	0.061	0.241
0.057	0.598	0.425	0.000	12.297	2.683	13.684	0.328	0.280	0.000	0.113	0.000	0.080	0.000	0.000	0.245
0.076	1.625	0.230	0.004	9.248	2.906	6.302	0.454	0.317	0.000	0.618	0.051	0.131	0.029	0.061	0.840
0.130	3.754	0.146	0.004	10.936	1.647	5.557	0.301	0.494	0.000	0.233	0.000	0.235	0.000	0.000	0.624
0.043	3.755	0.000	0.011	6.326	1.007	14.408	1.242	0.649	0.000	0.258	0.000	0.174	0.000	0.061	0.724
0.019	1.036	0.249	0.004	15.244	2.824	2.398	1.111	0.953	0.072	0.070	0.000	0.581	0.000	0.033	0.573
0.145	1.948	0.000	0.000	5.837	2.910	4.382	0.686	0.562	0.000	0.200	0.000	0.283	0.041	0.114	1.008
0.077	1.614	0.217	0.021	12.462	1.675	2.742	0.420	0.266	0.000	0.122	0.000	0.082	0.000	0.029	0.668
0.079	7.468	0.241	0.006	12.202	2.013	12.866	0.559	0.522	0.000	0.203	0.000	0.251	0.000	0.048	1.013
0.108	2.557	0.000	0.039	15.979	1.504	11.653	0.314	0.408	0.118	0.140	0.000	0.256	0.000	0.072	0.549
0.103	0.867	0.110	0.027	11.173	2.568	5.971	0.765	0.787	0.000	0.103	0.000	0.271	0.084	0.044	2.244
0.065	0.310	0.077	0.000	15.040	1.874	5.231	0.987	0.606	0.067	0.158	0.025	0.421	0.000	0.051	1.610
0.063	1.568	0.157	0.011	16.918	3.730	2.023	0.486	0.651	0.000	0.118	0.000	0.357	0.120	0.021	0.590
0.119	1.921	0.140	0.007	9.874	2.355	10.004	0.469	0.739	0.000	0.145	0.000	0.319	0.000	0.000	0.694
0.031	1.146	0.000	0.000	14.204	1.621	9.974	0.640	0.523	0.064	0.082	0.000	0.208	0.000	0.039	0.450
0.033	1.640	0.495	0.000	17.957	4.202	6.046	0.178	0.335	0.000	0.223	0.000	0.131	0.227	0.040	0.282
0.054	2.905	0.178	0.009	14.251	1.982	2.944	0.412	0.636	0.000	0.092	0.000	0.471	0.000	0.024	0.547
0.062	3.519	0.175	0.004	15.008	1.572	3.892	0.630	0.805	0.076	0.311	0.000	0.380	0.000	0.000	0.445

ENSG00000251266	ENSG00000274764	ENSG00000005844	ENSG00000241088	ENSG00000243824	ENSG00000251296
0.000	0.000	2.604	0.090	0.596	0.000
0.000	0.000	0.735	0.115	0.887	0.000
0.000	0.000	1.681	0.000	1.354	0.000
0.000	0.000	1.675	0.000	0.511	0.000
0.000	0.000	5.962	0.215	0.427	0.000
0.000	0.000	9.767	0.034	0.637	0.000
0.000	0.000	10.089	0.191	0.843	0.000
0.000	0.000	3.453	0.000	0.535	0.000
0.000	0.000	1.420	0.093	0.928	0.000
0.000	0.000	5.415	0.543	1.500	0.000
0.000	0.000	1.801	0.134	0.406	0.000
0.000	0.000	6.125	0.000	0.878	0.000
0.000	0.000	2.830	0.036	0.709	0.000
0.000	0.000	3.290	0.033	0.442	0.000
0.000	0.000	2.576	0.297	1.313	0.000
0.000	0.000	12.673	0.033	0.405	0.030
0.000	0.000	1.142	0.038	0.255	0.000
0.000	0.000	1.429	0.137	0.807	0.000
0.000	0.000	2.340	0.035	0.883	0.000
0.000	0.000	1.564	0.000	0.205	0.000
0.000	0.000	1.108	0.040	0.744	0.000
0.000	0.000	1.208	0.000	0.362	0.000
0.000	0.000	2.167	0.120	0.751	0.000
0.000	0.000	1.930	0.170	0.827	0.000
0.000	0.000	4.037	0.152	0.377	0.000
0.000	0.000	3.635	0.000	0.305	0.000
0.000	0.000	2.561	0.045	1.083	0.040
0.000	0.000	1.281	0.000	0.867	0.000
0.000	0.000	1.671	0.039	0.432	0.035
0.000	0.000	0.865	0.079	1.216	0.000
0.000	0.000	1.457	0.129	0.429	0.000
0.000	0.000	1.322	0.203	0.599	0.000
0.000	0.000	3.526	0.000	0.653	0.000
0.000	0.000	1.250	0.000	0.588	0.000
0.000	0.000	2.094	0.080	1.064	0.000
0.000	0.000	3.878	0.073	0.482	0.000
0.000	0.000	2.789	0.035	0.077	0.000
0.000	0.000	10.080	0.069	0.230	0.000
0.000	0.000	2.011	0.041	0.594	0.000
0.000	0.000	1.449	0.000	0.378	0.000
0.000	0.000	4.128	0.000	0.329	0.039
0.000	0.000	1.659	0.000	0.357	0.000
0.000	0.000	2.282	0.040	0.349	0.000
0.000	0.000	3.038	0.102	0.337	0.000
0.000	0.000	1.085	0.000	0.479	0.000
0.000	0.000	3.637	0.106	0.390	0.000
0.000	0.000	0.185	0.039	1.245	0.000
0.000	0.000	3.793	0.039	0.695	0.000
0.050	0.000	1.556	0.102	0.791	0.000
0.000	0.000	3.907	0.161	0.889	0.000
0.000	0.000	1.467	0.097	0.536	0.000
0.000	0.000	2.971	0.035	0.808	0.000
0.000	0.000	1.250	0.106	0.548	0.000
0.000	0.000	2.876	0.063	0.415	0.000
0.000	0.000	0.530	0.289	1.529	0.000
0.000	0.000	0.703	0.301	1.162	0.000
0.000	0.000	1.286	0.112	0.660	0.000
0.000	0.000	1.974	0.071	0.468	0.000
0.000	0.000	3.133	0.105	0.659	0.000
0.000	0.000	2.130	0.065	0.795	0.000
0.000	0.000	0.617	0.000	0.585	0.000
0.000	0.000	1.652	0.180	1.127	0.000
0.000	0.000	0.688	0.062	0.587	0.000
0.000	0.000	1.907	0.067	0.370	0.000
0.000	0.000	0.679	0.112	0.330	0.000
0.000	0.000	0.905	0.096	0.636	0.000
0.000	0.000	0.973	0.000	0.509	0.000
0.000	0.000	0.309	0.132	0.388	0.000
0.000	0.000	2.612	0.175	0.515	0.000
0.000	0.000	0.849	0.138	0.763	0.000
0.000	0.000	1.275	0.101	1.038	0.000
0.000	0.000	1.748	0.000	0.450	0.000
0.000	0.000	0.993	0.062	0.713	0.000
0.000	0.000	1.927	0.000	0.603	0.000
0.000	0.000	3.462	0.046	0.511	0.000
0.000	0.000	0.568	0.056	0.492	0.000
0.000	0.000	2.346	0.039	1.720	0.000
0.000	0.000	0.437	0.234	1.548	0.000
0.000	0.000	0.884	0.098	0.975	0.000
0.000	0.000	1.917	0.098	0.919	0.000
0.000	0.000	0.793	0.090	1.140	0.000
0.000	0.000	1.342	0.035	0.466	0.000
0.000	0.000	6.457	0.042	0.646	0.000
0.000	0.000	3.897	0.189	0.366	0.000
0.000	0.000	1.803	0.039	0.262	0.000
0.000	0.000	0.974	0.042	0.463	0.000
0.000	0.000	1.324	0.066	0.437	0.000
0.000	0.000	4.652	0.103	0.380	0.000
0.000	0.000	0.692	0.000	0.347	0.000
0.000	0.000	0.895	0.184	1.488	0.000
0.000	0.000	0.411	0.065	0.542	0.000
0.000	0.000	0.364	0.000	0.634	0.000
0.000	0.000	6.354	0.098	0.648	0.000
0.000	0.000	1.104	0.034	0.642	0.000
0.000	0.000	1.470	0.099	0.764	0.000
0.000	0.000	1.267	0.076	0.211	0.000
0.000	0.000	0.909	0.080	0.885	0.000
0.000	0.000	2.234	0.163	0.721	0.000
0.000	0.000	4.788	0.413	1.827	0.000
0.000	0.000	1.671	0.000	0.842	0.000
0.000	0.000	1.760	0.080	0.442	0.000
0.000	0.000	0.750	0.104	1.269	0.000
0.000	0.000	2.771	0.000	0.487	0.000
0.000	0.012	6.515	0.044	0.483	0.000
0.000	0.000	0.724	0.100	0.222	0.000
0.000	0.000	3.480	0.000	0.873	0.000
0.000	0.000	2.095	0.000	0.133	0.000
0.000	0.000	0.400	0.000	0.552	0.000
0.000	0.000	1.427	0.101	0.481	0.000
0.000	0.000	1.267	0.168	1.438	0.000
0.000	0.000	1.306	0.000	0.394	0.000
0.000	0.000	0.363	0.000	0.410	0.000
0.000	0.000	8.744	0.000	0.254	0.000
0.000	0.000	3.907	0.164	0.727	0.000
0.000	0.000	1.112	0.000	0.906	0.000
0.000	0.000	1.752	0.124	0.825	0.000
0.000	0.000	1.250	0.036	0.316	0.000
0.000	0.000	2.011	0.041	0.869	0.000
0.000	0.000	1.662	0.000	1.032	0.000
0.000	0.000	0.994	0.000	0.755	0.000
0.000	0.000	2.579	0.031	0.547	0.000
0.410	0.000	1.492	0.233	0.772	0.000
0.000	0.000	3.381	0.082	1.127	0.000
0.000	0.000	2.414	0.162	0.537	0.000
0.000	0.011	1.729	0.000	0.462	0.000
0.000	0.000	3.474	0.073	1.042	0.000
0.000	0.000	2.673	0.000	1.306	0.000
0.000	0.000	1.926	0.094	0.587	0.000
0.000	0.000	1.779	0.058	0.352	0.000
0.000	0.000	0.749	0.031	0.728	0.028
0.000	0.000	1.882	0.060	0.297	0.000
0.000	0.000	2.517	0.094	0.935	0.000
0.000	0.000	1.833	0.170	0.975	0.000
0.000	0.000	1.587	0.175	0.819	0.000
0.000	0.000	0.944	0.089	0.393	0.000
0.000	0.000	2.302	0.099	0.436	0.045
0.000	0.000	2.153	0.166	0.367	0.000
0.000	0.000	0.811	0.045	0.845	0.000
0.000	0.000	0.919	0.157	1.178	0.000
0.000	0.000	1.028	0.032	0.639	0.000
0.000	0.000	2.278	0.115	1.711	0.000
0.000	0.000	1.306	0.060	0.727	0.000
0.000	0.000	1.780	0.041	0.403	0.000
0.000	0.000	1.293	0.036	0.603	0.000
0.000	0.000	1.029	0.036	0.987	0.000

Table S4. Summary of the main features of Reboot and similar tools.

Tool	Reboot	HDMAC	Biospear	BhGLM	KM-Plotter
Penalized cox regression	YES	YES	YES	YES	NO
Bootstrap	YES	NO	YES#	NO	NO
Web interface	YES	YES	NO	NO	YES
Detailed Documentation	YES	NO*	YES	NO*	NO*
High-dimensional data	YES	YES+	YES	YES	NO
Evaluation of Clinical parameters	YES	YES	NO	NO	YES
Pre-filtering	YES	NO	NO	NO	NO~
Integrative approach	YES	NO	NO	NO	NO
Validation	YES	NO	NO	NO	NO
Graphical Signature	YES	NO	YES	YES	NO
Graphical Survival Outcome	YES	NO	NO	NO	YES
Command line	YES	NO	YES	YES	NO

(#) Biospear calculates expected survival using bootstrap, but does not attribute significance this way; (*) Detailed documentation available, including usage example, command features, explained parameters and installation guide is proposed. All tools provide some sort of usage guidance, yet maintain implicit some parameters and analysis steps; (+) HDMAC claims to perform high dimension analysis, but all attempts with more than 1000 genes returned errors; (~) KM-Plotter allows user to subset the raw datasets available in order to perform further analysis.

Table S5. Protein-coding genes up-regulated in glioblastoma in comparison to low-grade glioma patients (log2FoldChange ≥ 2 and FDR adjusted p-value < 0.05).

gene.symbol	log2FoldChange	FDR
HOXC13	8.31267	3.08E-187
HOXC11	8.16204	6.40E-194
SAA1	8.13785	2.19E-160
PLA2G2A	7.69151	2.85E-129
HOXD13	7.62622	8.26E-188
SFT2D3	7.56194	9.28E-11
PRAC2	6.91535	2.78E-108
HOXD11	6.84318	3.23E-112
PI3	6.82494	8.89E-146
HOXB8	6.76164	5.14E-115
DAZ2	6.30930	1.50E-15
SAA2-SAA4	6.10032	3.03E-76
LBX1	5.97443	5.57E-91
PITX2	5.93513	3.38E-117
OTP	5.90006	1.60E-135
POSTN	5.89122	1.88E-104
SAA2	5.88792	5.68E-210
C5orf46	5.73150	5.73E-78
C6orf15	5.69659	9.17E-42
NKX2-5	5.64422	3.07E-139
MMP9	5.57707	4.99E-172
CCL7	5.55751	1.23E-60
CA3	5.54211	4.34E-230
HOXD10	5.52211	5.91E-113
IBSP	5.51025	7.14E-85
HOXC8	5.50481	3.11E-97
CHI3L1	5.49907	2.74E-113
HOXC10	5.43846	5.37E-67
HOXC9	5.41247	3.78E-75
KRT75	5.38595	1.66E-92
AL445238.1	5.38364	2.06E-69
CAPN6	5.36698	4.60E-126
HOXB9	5.36390	2.92E-124
NNMT	5.34006	1.39E-224
CD70	5.32724	5.51E-151
IL36B	5.27517	2.13E-54
VGLL2	5.25022	9.48E-109
HIST1H3F	5.17573	8.89E-66
GATA4	5.14183	1.93E-78
DAZ4	5.10323	1.59E-30
FMOD	5.09929	5.24E-212
HOXB3	5.05409	5.72E-129

HOXA5	5.04195	5.70E-90
HOXD12	5.01753	1.61E-88
PRDM13	5.00345	2.14E-54
UNCX	4.97530	4.57E-30
HOXB13	4.95180	5.31E-141
IGFBP2	4.86835	3.23E-264
SCNN1B	4.85387	1.56E-127
HOXA9	4.85242	8.56E-72
SHOX2	4.85171	1.08E-106
MEOX2	4.84083	1.94E-115
LTF	4.82009	3.87E-60
CHRNA9	4.79487	9.24E-115
PTX3	4.78183	5.50E-230
STOML3	4.77738	4.67E-51
HOXA6	4.77692	3.28E-104
OR51E1	4.75088	3.80E-150
MCEMP1	4.73554	2.94E-98
COL3A1	4.70929	2.41E-154
HIST1H2BH	4.70528	8.58E-194
COL1A1	4.70070	3.27E-133
HOXA4	4.66575	2.45E-50
HOXC6	4.65932	1.04E-82
IGF2BP3	4.65073	8.34E-298
HOXA3	4.64331	1.13E-51
GALNT5	4.64184	3.99E-136
HOXA2	4.64103	2.48E-50
SEC61G	4.63731	2.87E-252
CCDC140	4.59655	1.28E-32
EN1	4.57932	5.85E-84
CA9	4.57170	3.60E-134
COL6A2	4.55410	1.02E-232
HOXB4	4.53749	3.55E-84
MYBPH	4.52562	3.95E-151
SIX6	4.52395	4.90E-39
LRRC15	4.52202	2.30E-87
LIF	4.51518	1.21E-172
HIST1H3B	4.47404	5.24E-96
CXCL8	4.46174	2.38E-102
IL2RA	4.44521	3.48E-133
POTEE	4.42879	4.57E-123
CXCL10	4.41041	3.10E-120
COL1A2	4.40471	1.32E-182
TREM1	4.39183	3.49E-106
HOXB5	4.38818	1.83E-50
LOX	4.37588	5.33E-227
MMP7	4.37513	8.59E-75

HIST1H1B	4.37354	4.47E-87
TIMP1	4.37282	1.39E-144
HOXB2	4.34426	1.11E-116
COL6A3	4.29016	1.59E-135
CXCL6	4.28692	4.82E-87
OTOR	4.28107	3.99E-39
MMP13	4.27360	3.16E-99
MMP12	4.26752	1.52E-41
MAB21L2	4.24710	1.09E-54
PPDPFL	4.23814	1.14E-78
SLAMF9	4.23810	9.47E-162
AC008763.3	4.22549	3.82E-13
CAMP	4.22269	5.05E-50
CNPY1	4.22252	4.75E-130
HP	4.19105	7.19E-122
MAGEC2	4.18327	3.90E-51
HIST1H3G	4.17711	5.96E-107
IDO1	4.14817	4.43E-67
HIST1H2AJ	4.13446	2.27E-54
C15orf48	4.13266	1.29E-187
WISP1	4.12861	5.87E-119
TNFAIP6	4.12564	3.16E-201
GDF15	4.12411	2.10E-149
LUM	4.10820	9.17E-144
CHRNA1	4.10693	9.09E-88
ZNF679	4.10616	1.84E-25
HOXA7	4.10495	5.03E-45
PDPN	4.10011	7.54E-158
MFAP2	4.08419	7.97E-114
BPIFB4	4.03207	1.53E-53
CTHRC1	4.03075	3.12E-172
IGF2BP2	4.02947	1.69E-147
ABCC3	4.01352	1.09E-104
RETN	3.99422	1.13E-60
STC1	3.99277	5.78E-145
MMP8	3.97893	2.54E-49
PNLIPRP3	3.97298	2.57E-47
AC112229.3	3.96238	3.59E-21
MSMP	3.95505	1.37E-70
PHLDA2	3.94588	3.55E-137
ADAM12	3.94557	2.99E-221
DAZ1	3.94499	8.11E-30
CCL23	3.94346	6.43E-55
FOXB1	3.91604	6.45E-57
COL5A1	3.89977	7.87E-132
ESM1	3.88452	8.79E-77

DEPDC1	3.88330	1.22E-227
OTX2	3.88197	2.26E-23
FOXA2	3.87297	4.80E-31
IL13RA2	3.86368	6.83E-82
MYBL2	3.85369	2.84E-161
CLEC5A	3.84107	4.89E-92
PRAME	3.83463	1.05E-85
PCOLCE	3.82779	4.31E-221
CYP27B1	3.82291	1.19E-153
DMRTA2	3.81083	1.56E-80
SRY	3.80632	6.56E-24
PITX1	3.79025	1.69E-96
CXCL11	3.78613	7.99E-92
FGF19	3.78566	2.78E-20
EDN2	3.78282	7.19E-63
SERPINE1	3.76472	1.95E-94
F13A1	3.76418	1.27E-91
VEGFA	3.73759	3.94E-241
EPYC	3.73653	9.47E-22
CD1A	3.73571	6.86E-36
FAM183A	3.73164	4.66E-48
PLEK2	3.72202	3.90E-193
DPEP1	3.72172	6.39E-88
HIST1H3I	3.72076	7.33E-39
DPP4	3.70556	3.75E-174
EMP3	3.70284	1.66E-119
MMP1	3.67176	2.99E-40
METTL7B	3.66914	1.05E-90
ADM	3.66113	6.95E-140
HIST1H1D	3.65837	5.21E-83
HIST1H2BI	3.65674	2.11E-40
GPX8	3.64202	1.23E-140
HOXA1	3.63780	3.62E-92
FGF3	3.63624	3.85E-12
CHI3L2	3.63058	8.44E-71
CTAGE6	3.61969	2.84E-26
CENPA	3.61937	6.76E-222
PDX1	3.61821	7.22E-28
AC087632.1	3.61225	1.76E-140
CWH43	3.61087	2.03E-33
AC034102.2	3.60830	4.20E-06
KDELR3	3.60552	3.14E-207
FCGR2B	3.60222	1.49E-103
APCDD1L	3.59324	2.88E-33
GABRR1	3.58574	8.55E-65
OCIAD2	3.58269	3.42E-162

FABP5	3.58203	4.68E-127
HMGA2	3.57920	7.02E-128
S100A4	3.57121	3.00E-146
CSF2	3.56857	7.58E-22
GDF3	3.56562	6.13E-26
BIRC5	3.56028	2.58E-160
FAM92B	3.54097	9.71E-68
COL4A1	3.53991	1.10E-156
CDC20	3.53576	1.21E-210
HCAR3	3.53297	4.81E-48
RARRES2	3.52997	5.85E-92
ALPK2	3.52943	3.48E-104
HIST1H2BM	3.51533	1.33E-38
WT1	3.50449	8.84E-52
G0S2	3.49811	5.31E-81
UBE2C	3.49210	4.00E-140
MARCO	3.49000	1.21E-64
DLGAP5	3.48997	1.38E-139
HIST1H2AI	3.48885	1.68E-62
RRM2	3.47267	1.24E-172
POTEF	3.46244	1.08E-109
CD248	3.45935	1.41E-195
ISL2	3.45615	9.01E-81
CHODL	3.45227	5.19E-106
HOXB7	3.45190	5.45E-102
HOXD9	3.43918	2.01E-42
AQP9	3.43349	9.58E-81
GPR1	3.43316	8.35E-126
TNFRSF12A	3.41921	2.81E-131
CCL11	3.41504	3.96E-09
SPOCD1	3.41244	8.44E-77
GSX2	3.40634	2.12E-54
BMP5	3.40442	3.23E-48
CCL20	3.40000	1.46E-131
AREG	3.39823	9.88E-45
FBXO39	3.39530	1.31E-90
HGD	3.39372	1.67E-103
LIPN	3.37537	9.90E-42
DLK1	3.37386	1.33E-33
MAGEA3	3.37253	1.63E-26
GPR139	3.36918	2.47E-30
E2F7	3.36415	2.63E-182
KIF20A	3.36151	3.63E-147
IL36RN	3.35487	1.61E-21
AGR3	3.34241	2.06E-28
S100A9	3.33271	2.52E-88

ASF1B	3.32919	5.68E-207
STAC	3.32627	1.26E-57
SRPX2	3.32234	1.41E-102
GAGE2E	3.31842	1.48E-34
CCNB2	3.31678	8.65E-173
C22orf42	3.29918	1.06E-85
HIST1H2AL	3.28266	7.01E-55
NOS2	3.27858	4.52E-82
HOXC12	3.27584	4.79E-11
MELK	3.27484	1.38E-165
TFPI2	3.27421	8.54E-75
KRT32	3.27132	2.08E-22
MS4A6E	3.26665	1.89E-41
MCUB	3.26479	1.34E-233
TFAP2B	3.25599	5.20E-22
COL5A2	3.25431	3.37E-174
REG1B	3.25232	1.20E-15
VSTM1	3.25104	3.12E-58
HTRA3	3.24488	1.05E-140
MMP11	3.24321	1.61E-152
CDCA8	3.23189	1.19E-221
CD163	3.23152	2.10E-81
AC012531.2	3.23110	5.40E-18
HOXB6	3.22628	1.83E-59
CCL18	3.22468	2.76E-32
AL645922.1	3.22120	3.54E-23
S100A8	3.20032	1.52E-73
PIMREG	3.19631	6.47E-122
MUC3A	3.19618	2.14E-93
TDO2	3.19382	2.70E-95
TEX101	3.19208	1.67E-134
CSAG3	3.18806	4.75E-44
HOXA10	3.18082	7.65E-30
HIST1H3C	3.17957	7.17E-68
HMX3	3.17887	2.30E-14
PTTG1	3.17766	1.64E-232
HOXC5	3.17685	5.87E-23
BARX1	3.17331	1.47E-44
SPP1	3.17128	6.63E-119
IFI30	3.16787	1.16E-138
LOXL1	3.16310	6.89E-90
LGALS3	3.15861	4.51E-127
MAP1LC3C	3.15574	2.04E-112
MMP19	3.15367	1.77E-75
HIST1H2AH	3.15197	4.01E-44
AP000781.2	3.15159	1.64E-127

COL4A2	3.14731	4.47E-158
AURKB	3.14498	4.53E-121
AC126283.2	3.14402	9.70E-78
SPINK8	3.14293	3.02E-154
HCAR2	3.14142	4.47E-71
NEIL3	3.14096	2.67E-152
TROAP	3.14062	9.49E-149
ANXA1	3.13932	4.91E-88
GAS2L3	3.13877	2.08E-247
ANXA2	3.13503	2.10E-148
SCNN1G	3.13414	5.53E-98
SDC1	3.13168	1.77E-153
CDK4	3.12724	2.21E-112
PLP2	3.12182	5.25E-165
ZAR1	3.11965	3.17E-56
PLAT	3.11622	1.42E-208
CPXCR1	3.11598	2.93E-12
ENTHD1	3.10856	1.91E-45
TMEM270	3.10485	2.30E-45
ARL14EPL	3.10440	6.10E-34
CDCA2	3.10356	8.23E-159
AGTR1	3.09990	1.52E-105
C6orf141	3.09204	6.95E-107
ACOT12	3.08758	6.48E-83
FAM20A	3.07745	6.23E-120
HMX2	3.07492	1.51E-13
TUBA1C	3.07043	7.12E-231
STEAP3	3.06987	8.60E-134
IGFBP3	3.06647	1.51E-87
PDLIM4	3.05591	2.02E-69
TAGLN2	3.05358	7.18E-212
RFPL4A	3.04798	1.52E-10
ADAMDEC1	3.04445	8.94E-34
TBX20	3.04318	6.43E-09
CDC25C	3.03855	2.34E-155
PBK	3.03838	1.32E-106
NODAL	3.03756	5.11E-133
NOX4	3.03746	8.03E-253
HAND2	3.03558	4.53E-26
THBS1	3.03255	7.09E-74
ERVV-1	3.03038	2.34E-25
RARRES1	3.02715	1.40E-103
IL1R2	3.02708	3.26E-69
HJURP	3.01987	6.74E-163
TNFRSF11B	3.01973	1.66E-96
CEP55	3.01895	2.77E-160

COL8A1	3.01503	8.07E-68
UCN2	3.01375	9.53E-68
TMEM71	3.00767	7.05E-127
TNFSF12-TNFSF13	3.00578	3.73E-31
TTK	3.00551	7.54E-137
RAB42	3.00275	2.76E-246
SLPI	2.99449	3.24E-74
CCL13	2.99268	5.14E-35
HIST1H4A	2.99119	8.26E-36
CLEC12A	2.99118	4.53E-64
NAMPT	2.99074	2.91E-170
CLCF1	2.98994	3.25E-97
OTOS	2.98819	3.87E-43
SPAG4	2.98705	8.38E-116
NEK2	2.98337	6.99E-158
CXCL9	2.98272	1.60E-58
BDKRB2	2.98170	7.80E-75
KIF2C	2.98108	1.42E-159
CDC45	2.97576	3.96E-145
CCNB1	2.97485	6.83E-303
EYA4	2.97483	1.17E-53
ESPNL	2.97180	8.25E-66
CER1	2.97099	1.04E-46
PLAU	2.96551	4.16E-85
EBF3	2.96542	3.86E-59
CXCL13	2.96330	2.17E-36
IL6	2.95598	2.65E-51
SPON2	2.95531	1.88E-94
VWDE	2.95280	1.54E-44
TWIST1	2.95144	2.21E-127
CDKN3	2.95005	1.92E-235
RBP1	2.94505	2.99E-79
SP8	2.93905	1.64E-35
PLSCR2	2.93784	2.43E-60
SSX1	2.93320	1.08E-42
SOCS2	2.92906	3.69E-137
AC005832.4	2.92849	4.12E-79
MSR1	2.92786	1.84E-127
TMEM114	2.92595	6.78E-42
CLIC1	2.92533	5.62E-200
GAL	2.92526	3.24E-53
DMP1	2.92366	1.27E-70
PGK2	2.92284	9.37E-36
AC011455.2	2.92244	1.36E-07
PAX3	2.92033	1.87E-36
JCHAIN	2.91255	4.47E-39

CCKAR	2.90941	6.96E-32
KISS1R	2.90900	2.43E-40
FEZF1	2.90891	1.95E-32
NHLH1	2.90863	6.24E-74
ULBP1	2.90785	7.05E-121
HS3ST3B1	2.90559	2.23E-72
TK1	2.90535	3.06E-205
FAM83D	2.90144	3.06E-163
PRRX2	2.90082	3.99E-51
SERPINH1	2.89829	3.48E-196
PLAUR	2.89559	1.67E-163
MEDAG	2.89533	3.38E-53
FBP2	2.89234	9.73E-13
EXOC3L2	2.88407	1.38E-169
FCGBP	2.88069	2.54E-71
TGFBI	2.87424	3.99E-81
KLHDC7A	2.87123	2.46E-28
AC005258.1	2.86560	2.67E-04
CSTA	2.86372	2.68E-64
SERPINB7	2.86025	2.77E-20
AC013470.2	2.85631	1.12E-63
NKX3-2	2.85475	4.61E-56
DEFA4	2.85471	2.61E-16
SH2D4A	2.85141	1.54E-100
LUZP6	2.85022	1.01E-20
DKK1	2.84811	3.04E-39
CCNA2	2.84567	8.63E-207
FCN3	2.84351	1.38E-99
STAP1	2.84251	3.56E-61
GJB2	2.84064	9.39E-48
KIF4A	2.83515	2.76E-170
CENPK	2.83419	1.90E-229
C20orf141	2.83234	3.93E-14
TLX3	2.83175	9.45E-10
SOCS3	2.83064	5.25E-59
AL035460.1	2.83033	2.45E-05
FEV	2.82849	6.59E-36
NCAPG	2.82739	9.39E-128
CSF3	2.82736	2.68E-29
AL022318.4	2.82694	4.24E-09
KIF23	2.82670	3.94E-150
CENPU	2.82095	3.45E-137
AC006486.1	2.82009	9.60E-17
MOXD1	2.81113	3.26E-38
PRL	2.80953	4.12E-15
AQP5	2.80903	8.14E-37

IGLL5	2.80566	8.80E-16
TOP2A	2.80523	3.19E-79
E2F8	2.80392	1.50E-136
BUB1	2.80263	5.91E-145
SERPINA3	2.80235	2.10E-50
SAA4	2.79428	3.60E-30
PKIB	2.79127	1.70E-79
KRT80	2.78940	1.30E-64
ADAM33	2.78804	3.34E-104
DEPDC1B	2.78704	6.54E-174
AL358472.7	2.78640	1.70E-05
LSP1	2.78155	1.56E-110
IL36G	2.78093	6.13E-36
DPYD	2.78023	9.50E-175
MAGEC1	2.77922	8.38E-25
NDC80	2.77119	1.27E-142
ANPEP	2.77055	1.98E-134
HIST2H2AC	2.77044	6.10E-112
TBX1	2.76967	8.52E-79
ULBP3	2.76828	5.57E-82
FPR2	2.76637	5.34E-86
RTL3	2.76560	2.19E-18
SIM1	2.76314	5.47E-39
RNASE2	2.76179	1.26E-66
DMBX1	2.76020	9.42E-98
GAGE13	2.75676	7.76E-10
HOXC4	2.75588	4.02E-84
CLEC18B	2.75576	4.51E-94
NSUN7	2.75556	7.78E-128
IL31RA	2.74420	1.28E-40
PDLIM1	2.74205	2.10E-104
BCAT1	2.74140	3.49E-137
NPPB	2.73875	4.50E-56
AFP	2.73698	3.66E-26
EXO1	2.73183	2.80E-153
ERVV-2	2.72948	3.41E-09
PPY	2.72794	5.47E-31
PRF1	2.72776	6.07E-90
FNDC11	2.72709	5.76E-75
VDR	2.72437	2.63E-94
LILRA5	2.72424	2.80E-97
MAGEA1	2.71754	2.61E-10
MXRA5	2.70976	2.18E-70
AOX1	2.70798	6.97E-97
SERPINA1	2.70632	8.36E-93
LAMB1	2.70624	2.98E-123

IQGAP3	2.70349	8.80E-127
EEF1AKMT3	2.70342	6.98E-102
DES	2.70270	1.32E-34
PXDNL	2.70229	2.52E-107
CDK1	2.69642	4.71E-123
C1orf158	2.69328	5.59E-47
CAVIN3	2.69306	2.39E-117
MAP3K7CL	2.69097	1.01E-107
SKA3	2.68891	3.66E-130
AC005943.1	2.68079	3.52E-51
RHOD	2.68044	2.45E-59
SGO1	2.67913	1.02E-142
TYMS	2.67847	1.38E-170
HBG1	2.67739	4.18E-46
HK3	2.67483	1.15E-124
SKA1	2.67296	1.51E-216
ICOS	2.66881	8.10E-44
EVC2	2.66770	9.37E-87
ADGRE3	2.66744	4.53E-43
HBE1	2.66385	2.28E-110
SMIM3	2.66343	2.28E-161
DEFA1	2.66136	3.12E-23
NUF2	2.66056	2.20E-165
SNAI2	2.65949	1.24E-95
S100A11	2.65905	1.08E-130
ASPM	2.65809	5.19E-85
HNRNPCL2	2.65793	2.48E-26
KLF14	2.65763	1.65E-10
RDM1	2.65692	3.10E-123
HIST1H4L	2.65449	4.53E-07
C7orf57	2.65124	3.78E-34
HIST1H2BO	2.65082	2.79E-61
CYP19A1	2.65023	1.44E-80
PTPN22	2.64361	3.94E-146
FBLIM1	2.64318	7.42E-148
CASP4	2.64087	1.27E-190
C9orf135	2.64072	6.79E-52
RAB34	2.64055	4.34E-107
APOL4	2.64051	3.26E-100
CARD16	2.63928	4.42E-117
CCDC182	2.63765	2.58E-53
CKAP2L	2.63465	2.21E-117
HPD	2.63395	1.61E-49
ORC1	2.63132	6.11E-174
NUSAP1	2.63052	2.79E-118
DPT	2.62831	2.52E-31

KIF18A	2.62758	3.14E-165
AC023055.1	2.62738	2.22E-129
OIP5	2.62494	1.76E-207
CPA4	2.62473	2.64E-117
OLIG3	2.61996	4.25E-07
STEAP1	2.61983	1.21E-75
SECTM1	2.61841	5.68E-81
PIGY	2.60614	3.34E-21
VAV3	2.60037	1.80E-72
CLRN1	2.59911	5.10E-41
CFAP45	2.59597	1.69E-51
ALX1	2.59447	3.48E-22
TCTEX1D1	2.59354	3.04E-36
MROH9	2.59071	1.28E-21
MMP3	2.59050	9.43E-28
SPRY1	2.59038	1.97E-132
HIST1H2AM	2.58998	8.20E-65
CATSPER1	2.58966	1.78E-82
HASPIN	2.58936	2.27E-122
DDIT4L	2.58867	2.21E-35
APOBEC3B	2.58781	1.13E-119
TPX2	2.58633	1.13E-130
HIST1H3J	2.58595	6.35E-39
PLA2G5	2.58554	7.41E-44
CD2	2.58418	4.80E-58
MARCH9	2.58271	6.92E-83
SH2D2A	2.58125	3.87E-93
ASPN	2.58087	2.14E-78
FOSL1	2.58045	4.41E-70
C1orf189	2.58005	2.83E-41
SLC18A3	2.57951	3.31E-18
FMO1	2.57939	4.41E-59
PRG2	2.57719	1.37E-27
NCAPH	2.57663	1.00E-133
LOXL2	2.57648	1.14E-89
PRR33	2.57603	5.19E-65
DEFA3	2.57599	6.66E-13
SPTA1	2.57547	8.09E-38
HIST1H2BJ	2.57387	1.00E-112
ALOX5AP	2.57229	1.13E-94
C11orf88	2.57121	1.37E-46
TRPM8	2.56881	3.66E-53
TMPRSS7	2.56679	5.72E-38
GBP5	2.56601	5.70E-84
CDC6	2.56024	3.43E-203
FOXM1	2.55909	1.92E-113

AL603832.3	2.55731	9.95E-138
ERCC6L	2.55683	4.13E-171
SOCS1	2.55639	1.53E-105
CKS2	2.55632	3.61E-213
MYO1G	2.55441	1.14E-109
SOST	2.55373	1.63E-25
METTL1	2.54726	3.15E-112
HIST1H3H	2.54264	2.32E-85
CHRDL2	2.54069	2.41E-54
LIMS4	2.53898	7.71E-65
CDKN2C	2.53647	1.79E-135
CISH	2.53636	9.52E-101
ANGPT2	2.53563	9.13E-101
MDK	2.53504	5.77E-135
PHOX2B	2.53496	3.13E-09
TEAD2	2.53464	9.52E-163
PINLYP	2.53441	1.24E-118
TRIM48	2.53384	1.52E-07
TERT	2.53279	1.84E-25
NKX2-1	2.53060	7.46E-25
CD3D	2.52984	2.55E-54
CHST4	2.52965	4.83E-32
XCR1	2.52854	1.15E-43
SLN	2.52790	9.97E-30
LGALS1	2.52687	3.89E-132
CCR2	2.52668	3.53E-41
AC020909.1	2.52564	1.63E-06
C1orf232	2.52543	2.32E-19
KLHDC8A	2.52367	6.06E-118
SLC43A3	2.51848	7.77E-165
CD58	2.51767	1.26E-162
RAD51	2.51546	2.33E-176
ITGB3	2.51249	2.90E-77
HILPDA	2.51114	1.15E-143
CD48	2.50955	5.19E-54
DIRAS3	2.50895	4.24E-72
IL1RAP	2.50863	9.67E-121
ITGA5	2.50712	6.00E-147
SEMA3F	2.50269	1.67E-135
HIST1H2AB	2.50261	1.16E-37
AURKA	2.49800	2.91E-253
HMMR	2.49652	2.84E-111
SLC7A3	2.49092	3.97E-44
ROPN1L	2.49015	2.85E-44
DEFA1B	2.49015	1.37E-02
MEST	2.48923	2.49E-140

LYZ	2.48897	1.74E-46
SLAMF1	2.48556	1.11E-50
CATSPERD	2.48535	6.43E-38
SPC24	2.48126	1.27E-147
GTSE1	2.48000	1.02E-128
KIAA0040	2.47858	2.33E-105
SLAMF8	2.47799	4.12E-56
CFB	2.47735	1.60E-90
CD52	2.47705	7.40E-70
TSPAN31	2.47516	1.54E-84
ADAMTS3	2.47420	3.25E-61
RAB32	2.47344	1.46E-137
GBP1	2.47273	5.40E-88
GZMA	2.47115	2.53E-48
TEAD3	2.46559	1.80E-136
PAGE5	2.46268	2.15E-15
FAM3D	2.46243	2.72E-65
FABP7	2.46208	2.60E-76
APOBEC4	2.46093	1.37E-13
RAD54L	2.45746	3.21E-147
CXCR3	2.45670	7.39E-41
DSG2	2.45606	1.56E-31
SBK2	2.45533	7.23E-17
HIST1H4B	2.45517	1.55E-25
CYTL1	2.45358	1.36E-91
FN1	2.45293	6.64E-119
DTL	2.45050	1.37E-95
GPR141	2.44857	1.52E-64
SIT1	2.44796	2.07E-59
PCLAF	2.44679	1.66E-168
EMILIN3	2.44591	3.00E-32
C2orf70	2.44443	4.20E-48
AL645941.2	2.44260	1.28E-12
TNFSF14	2.44175	3.29E-87
SHCBP1	2.43853	1.29E-216
BCL2A1	2.43839	9.97E-54
PPIAL4D	2.43815	1.53E-16
SERPINA5	2.43631	6.75E-25
HMOX1	2.43547	4.72E-108
PLIN2	2.43431	4.36E-161
IL1RN	2.43334	2.61E-44
TMPRSS15	2.43151	1.85E-31
CD300E	2.43121	8.34E-102
ENPEP	2.43077	5.39E-104
SERINC2	2.42738	3.26E-81
TRAT1	2.42682	5.75E-43

CD80	2.42583	1.36E-61
CEACAM16	2.42304	2.34E-14
AC010422.5	2.42096	2.32E-08
GPRC5A	2.41972	4.52E-65
PDCD1LG2	2.41913	1.24E-80
TCTEX1D4	2.41831	2.02E-35
WNT16	2.41818	2.40E-32
EDDM3A	2.41637	2.77E-11
NKX6-1	2.41604	1.36E-77
PMCH	2.41597	4.51E-17
RDH10	2.41534	9.69E-117
EVA1B	2.41046	1.77E-164
UBE2T	2.41032	6.62E-158
VNN2	2.40977	1.63E-78
MND1	2.40908	8.54E-134
RPS4Y2	2.40485	8.83E-25
DLX5	2.40427	4.65E-33
SERPING1	2.40339	2.89E-87
EFEMP2	2.40241	8.81E-111
IFNG	2.40238	2.43E-18
EMILIN2	2.39860	3.45E-86
AL451007.3	2.39833	1.93E-42
GPR65	2.39767	4.66E-73
LDHA	2.39369	7.80E-179
ITGA4	2.39329	1.17E-90
GRB14	2.39290	5.31E-52
ZNF280A	2.39098	2.71E-17
TSTD1	2.38752	5.42E-55
ESPL1	2.38248	1.13E-106
GATA3	2.38237	3.02E-40
MMP14	2.38065	5.66E-103
AC117378.1	2.37994	9.57E-38
ESCO2	2.37790	7.39E-116
ACTBL2	2.37482	4.35E-09
BUB1B	2.37323	2.50E-93
FAM111B	2.37212	4.18E-108
ERP27	2.37203	1.78E-66
ARSJ	2.37153	1.16E-76
PARPBP	2.37063	0.00E+00
MIA	2.37044	4.25E-31
RGS16	2.37043	2.83E-80
SLC16A3	2.36862	1.73E-130
CENPE	2.36758	1.43E-111
HIST1H2AD	2.36473	7.69E-22
WEE1	2.36447	8.58E-142
CALHM6	2.36244	4.44E-69

CSAG2	2.36185	1.07E-26
FOXD2	2.36042	2.18E-76
HIST1H4F	2.35887	9.71E-11
MATN4	2.35541	2.51E-46
WDR38	2.35473	6.36E-27
HIST2H2AA3	2.35436	1.86E-24
PLEKHN1	2.35426	1.42E-55
ISL1	2.35193	2.87E-10
MGP	2.35082	4.68E-38
HSPA6	2.34394	1.23E-51
DOK2	2.34040	9.31E-66
SOD2	2.34035	4.50E-93
FSTL1	2.33976	3.30E-154
C7orf33	2.33954	7.83E-09
CCL26	2.33315	4.00E-56
SIRPG	2.33202	7.24E-60
EGFR	2.33155	1.58E-55
GPR31	2.33012	4.02E-28
OSMR	2.32484	6.39E-98
PMEL	2.32361	8.15E-110
GNGT1	2.31678	1.03E-11
HLA-DRA	2.31645	2.78E-60
GJA3	2.31509	6.23E-27
C2orf66	2.31485	1.03E-86
KIFC1	2.31299	1.64E-80
IL4I1	2.31246	1.20E-83
MS4A6A	2.31195	2.57E-58
LRRC9	2.31159	1.09E-30
OSR2	2.30830	2.85E-21
AC092143.1	2.30787	1.57E-21
PLBD1	2.30715	9.12E-92
FGF21	2.30654	2.16E-10
ARAP3	2.30587	1.31E-166
BGN	2.30529	2.22E-104
GLI1	2.30485	5.73E-45
PHETA2	2.30396	1.36E-187
NT5DC4	2.30342	8.09E-36
CFD	2.30236	8.44E-71
OTX1	2.29921	6.10E-47
CD36	2.29808	1.17E-49
HDC	2.29724	1.02E-21
EBF2	2.29684	9.55E-77
TYMP	2.29462	1.10E-65
FAM19A3	2.29417	4.97E-36
TRIM6-TRIM34	2.29367	1.02E-02
C1orf185	2.29028	1.70E-12

PLK1	2.29020	2.73E-170
FBLN7	2.28867	4.53E-53
OASL	2.28810	3.50E-51
RPL39L	2.28702	1.18E-71
LY96	2.28594	1.22E-83
TULP1	2.28291	4.91E-54
CXCR6	2.28159	4.00E-48
TENT5B	2.28158	2.20E-42
ITLN2	2.28109	9.86E-17
CDKN2A	2.28044	1.18E-34
MPZL2	2.28003	2.63E-68
CELSR1	2.27945	1.58E-54
ITIH6	2.27922	5.46E-08
SFRP4	2.27920	8.65E-51
ADM2	2.27478	2.11E-66
CLDN23	2.27429	2.34E-85
FCGR3A	2.27422	1.39E-63
IRX5	2.27362	3.82E-28
BPIFB3	2.27278	9.11E-13
TMEM158	2.27200	1.27E-81
NKX2-3	2.27065	1.42E-09
TP73	2.27018	2.46E-49
DDIAS	2.26967	1.35E-171
LCTL	2.26589	3.08E-102
SPRY4	2.26543	6.35E-55
BCL3	2.26129	2.94E-129
BTN1A1	2.25739	8.06E-35
FBXO17	2.25694	1.34E-88
P2RY10	2.25538	2.47E-25
DCN	2.25486	7.90E-65
XCL2	2.25401	7.90E-31
DLX2	2.25358	3.76E-28
IL17B	2.24844	1.40E-56
ISG20	2.24742	2.85E-125
ITGBL1	2.24515	4.39E-39
AP002748.4	2.24366	2.16E-09
REG1A	2.24343	1.01E-08
AKAP14	2.24173	2.78E-32
HIST1H4D	2.23980	1.38E-31
TREML4	2.23969	9.54E-23
HIST1H4C	2.23885	1.85E-38
PRG4	2.23540	6.14E-45
DNAH11	2.23313	1.01E-79
ROR2	2.23205	1.48E-46
TAGLN	2.23150	1.97E-87
OAS1	2.22783	1.60E-70

CFAP73	2.22774	3.54E-32
PDZK1IP1	2.22645	3.70E-32
GINS2	2.22409	7.59E-176
TCIM	2.22324	6.75E-66
HOXD3	2.22114	1.18E-24
HSPG2	2.21900	2.91E-81
CENPM	2.21849	9.14E-133
MCM2	2.21609	3.16E-190
SIGLEC7	2.21541	4.89E-70
POC1A	2.21510	1.31E-198
TRIP13	2.21478	8.57E-216
CRABP1	2.21465	3.81E-24
TNMD	2.21205	7.89E-39
KMO	2.20843	4.60E-73
HIST1H2BB	2.20794	1.48E-20
KIF14	2.20771	5.31E-101
GPR18	2.20526	1.03E-51
POTEJ	2.20077	9.24E-03
HAMP	2.20034	8.79E-45
NDUFA4L2	2.19946	1.42E-130
FKBP9	2.19771	2.04E-167
GZMK	2.19607	2.30E-30
TACC3	2.19555	1.91E-156
CD93	2.19543	5.03E-81
LAMC1	2.19399	8.94E-140
HMHB1	2.19238	1.03E-15
LDLRAD2	2.18978	8.51E-54
SCN4A	2.18694	2.09E-34
AC008764.4	2.18694	5.73E-42
C21orf62	2.18630	7.10E-58
HOPX	2.18627	7.16E-49
FST	2.18363	1.17E-52
GAGE12F	2.18358	6.99E-10
SPAG17	2.18268	7.27E-28
MTFR2	2.18228	6.48E-173
PSORS1C2	2.18112	8.28E-24
SFTA2	2.18076	4.10E-44
LXN	2.18026	2.28E-130
IL10	2.17942	2.30E-60
GPX7	2.17891	5.06E-206
CCER2	2.17883	4.63E-61
UPP1	2.17808	5.94E-118
ICAM3	2.17488	2.17E-129
CRIP1	2.17465	2.07E-103
TMSB10	2.17434	2.83E-151
GPR84	2.17198	4.75E-57

KLHL4	2.17186	3.70E-75
VIM	2.17144	1.17E-70
AC187653.1	2.17105	4.03E-10
LGALS7B	2.16853	1.92E-11
ADGRE1	2.16743	1.21E-36
MCTP2	2.16281	2.16E-40
FGFBP2	2.16271	1.09E-30
CFAP161	2.16175	8.62E-34
C1R	2.16145	1.11E-52
TMEM61	2.15740	2.42E-26
SPINK1	2.15561	5.91E-39
HS3ST3A1	2.15335	5.35E-29
PDGFD	2.15307	7.99E-39
STRA6	2.15205	1.43E-57
BEST4	2.15182	3.49E-65
IL22RA2	2.15061	4.90E-07
RAB38	2.15029	2.15E-73
TMSB15A	2.14970	3.23E-39
ADGRE5	2.14895	1.97E-120
FAM20C	2.14779	4.23E-110
AEBP1	2.14734	1.60E-42
PODNL1	2.14637	6.83E-52
KRT7	2.14630	4.55E-30
AC012254.2	2.14611	8.69E-31
LBP	2.14534	1.01E-18
C1RL	2.14316	2.68E-88
ULBP2	2.14279	1.19E-90
CENPF	2.14249	8.30E-72
FRRS1	2.13830	5.75E-95
MSN	2.13824	1.22E-115
PLSCR1	2.13774	9.41E-123
SLC11A1	2.13716	9.16E-66
PSRC1	2.13649	7.01E-128
WDR62	2.13560	5.32E-151
TGM5	2.13494	1.55E-38
S100A12	2.13362	3.53E-28
SLC2A2	2.13322	1.92E-20
CDCP1	2.13157	1.30E-73
CLLU1OS	2.13004	1.68E-20
VASN	2.12929	2.95E-41
Z82206.1	2.12874	2.70E-49
CLCNKB	2.12844	3.02E-54
TFF3	2.12830	2.63E-25
AC104389.5	2.12673	1.65E-14
CD101	2.12447	1.82E-127
HLA-DQA1	2.12239	1.10E-37

CRYBG1	2.12227	1.60E-70
CPVL	2.12051	6.42E-86
AC004233.2	2.12027	4.53E-22
SP6	2.11796	1.03E-77
F2RL2	2.11777	6.40E-30
OMP	2.11715	5.68E-42
EGFLAM	2.11644	2.22E-66
FAM129A	2.11635	3.98E-81
CFH	2.11515	1.92E-76
CIDEB	2.11351	1.01E-108
ETV7	2.11343	2.57E-104
NID2	2.11313	4.46E-79
RAD51AP1	2.11270	1.31E-142
FOXJ1	2.11191	7.51E-42
RFPL4AL1	2.11125	3.22E-06
ECSCR	2.11078	7.06E-102
GJD3	2.10668	1.36E-09
POLE2	2.10511	1.55E-171
KAAG1	2.10462	2.20E-21
OR2B6	2.10403	1.14E-38
SMC4	2.10378	6.16E-208
AC073612.1	2.10204	1.73E-07
ARL9	2.10035	4.05E-68
CAV1	2.10031	2.09E-54
HIST2H2AB	2.09991	3.03E-27
KISS1	2.09865	2.57E-22
TRIM29	2.09748	2.59E-34
CLEC1B	2.09687	3.28E-27
PLEKHS1	2.09630	2.60E-36
CENPW	2.09588	1.15E-135
PRC1	2.09523	3.75E-148
AC006978.2	2.09484	5.10E-57
EN2	2.09355	8.00E-47
RASSF10	2.09280	1.74E-26
AL109827.1	2.09268	2.94E-06
THBD	2.09220	2.27E-55
AC090227.1	2.09158	1.02E-04
MYL4	2.09057	6.91E-73
GATA6	2.08865	2.06E-24
ACTG2	2.08845	5.16E-36
CR1	2.08832	7.59E-44
EPO	2.08793	2.14E-50
FOLR1	2.08743	1.87E-53
PPIC	2.08673	7.07E-129
CELA1	2.08585	1.68E-33
FOXA1	2.08563	5.48E-13

AC010255.3	2.08216	1.93E-26
TMEM26	2.08069	6.24E-66
PTCRA	2.07582	5.83E-41
OLFML1	2.07517	4.33E-77
SLC47A2	2.07510	2.69E-33
TXLNB	2.07355	1.59E-45
TWIST2	2.07315	4.54E-37
BIRC3	2.07276	5.53E-70
APOBEC3F	2.07259	7.84E-122
ITPRIPL1	2.07244	2.60E-103
LRRC8E	2.07213	9.58E-70
NFKBIZ	2.07210	3.79E-91
THEGL	2.06926	9.18E-37
ZNF474	2.06917	1.75E-39
CCDC8	2.06883	4.41E-48
NPNT	2.06670	3.39E-36
POTEG	2.06534	4.17E-18
TUBB6	2.06524	3.79E-88
OBP2A	2.06498	8.17E-19
AL121722.1	2.06484	4.00E-14
CEACAM3	2.06482	2.16E-33
PLVAP	2.06481	1.28E-86
CDK2	2.06233	7.53E-199
PYGL	2.06220	1.54E-112
FAP	2.06187	1.79E-46
IFNB1	2.06179	6.06E-08
SIX3	2.05966	2.03E-28
APOBEC3G	2.05870	6.62E-121
MMP10	2.05831	5.27E-14
SOAT2	2.05827	1.31E-24
ADAMTS7	2.05814	4.58E-66
MMP2	2.05659	2.37E-96
IKBIP	2.05549	2.48E-236
ZP1	2.05396	9.36E-58
MEIKIN	2.05387	1.38E-37
IGFBP4	2.05150	1.51E-99
CARD17	2.04989	2.27E-12
IL7	2.04932	1.70E-82
GPNMB	2.04914	1.88E-36
GZMB	2.04744	1.00E-39
NIPSNAP2	2.04744	1.91E-80
ACOD1	2.04637	9.39E-09
MCM10	2.04563	1.12E-56
SNAI1	2.04428	2.25E-47
TNFSF11	2.04409	9.12E-33
TNFRSF4	2.04393	1.01E-87

TMEM189-UBE2V1	2.04295	1.77E-37
CCR8	2.04265	6.33E-15
APOC2	2.04216	6.70E-60
LRRC36	2.04198	4.81E-104
TOM1L1	2.04065	8.05E-46
ATP23	2.04048	5.17E-75
EOMES	2.03989	5.67E-36
MTRNR2L7	2.03873	1.16E-36
FOXL1	2.03836	1.97E-85
PRSS21	2.03822	1.08E-29
MEOX1	2.03783	1.96E-28
ANKRD53	2.03783	7.10E-117
S100A3	2.03685	2.60E-31
CST2	2.03673	1.25E-08
KCNN4	2.03638	2.75E-47
IGFBP5	2.03606	2.26E-68
KCNE1B	2.03535	9.34E-19
ANG	2.03519	3.32E-65
CLEC2B	2.03461	4.81E-72
DCAF8L2	2.03448	1.10E-21
CTSK	2.03368	1.31E-71
CRABP2	2.03298	1.68E-65
PSORS1C1	2.03238	7.95E-43
FCAMR	2.03224	2.29E-18
DCDC2C	2.02902	1.07E-21
AFAP1L1	2.02834	3.00E-118
ACE	2.02827	3.07E-92
C5AR1	2.02689	3.15E-63
ZBED6CL	2.02679	1.02E-50
SLC27A3	2.02582	5.89E-112
CAPG	2.02367	4.39E-79
HAL	2.02198	3.07E-43
TM4SF1	2.02146	3.59E-72
CPA6	2.02001	1.64E-32
RUNX1	2.01969	1.88E-74
EGFL6	2.01743	1.41E-29
C4orf51	2.01733	2.33E-11
CDC42BPG	2.01580	1.17E-61
HLA-DQB1	2.01575	5.04E-41
GPR171	2.01560	2.01E-38
CDC42EP5	2.01438	5.79E-55
ST14	2.01137	2.48E-52
TLR8	2.00995	1.40E-45
ADAMTSL4	2.00782	1.27E-106
CST7	2.00768	5.38E-56
UBE2S	2.00610	2.01E-166

CASP5	2.00563	1.83E-52
OBP2B	2.00439	3.03E-14
LOXL4	2.00244	7.84E-36
CCL2	2.00238	7.81E-28
WNT10A	2.00116	1.07E-40

FDR: False Discovery Rate.

Table S6. Genes associated with glioblastoma patients' overall survival in Reboot.

gene.symbol	regression.coefficient
PODNL1	0.0114270
IKBIP	0.0103428
OSMR	0.0089689
THBD	0.0073175
SPAG4	0.0072413
HOXD11	0.0071885
CLEC18B	0.0068656
KCNN4	0.0066474
HOXC13	0.0064113
TNFSF12-TNFSF13	0.0055134
HOXD10	0.0053362
PLAUR	0.0050376
SECTM1	0.0049661
RPL39L	0.0048566
ANG	0.0046677
EFEMP2	0.0046008
MMP19	0.0043592
DDIT4L	0.0042875
MPZL2	0.0040587
EN1	0.0040515
SLC16A3	0.0036613
CCDC8	0.0036318
ITGA5	0.0034568
IL6	0.0034342
LOXL1	0.0032843
UPP1	0.0031997
HOXB7	0.0031809
VASN	0.0031462
MMP11	0.0031151
PTX3	0.0031112
LSP1	0.0030449
SLC43A3	0.0028308
ISG20	0.0026915
HOXA2	0.0026319
SOCS3	0.0025745
PDLIM4	0.0025696
MND1	0.0024229
C1RL	0.0023726
STC1	0.0023486
DKK1	0.0023052
STRA6	0.0021638
CLEC2B	0.0021004

CAV1	0.0020853
GAS2L3	0.0020127
RRM2	0.0019973
LIF	0.0019962
MMP2	0.0019744
PLBD1	0.0019181
FOSL1	0.0018222
DIRAS3	0.0017363
PAX3	0.0016939
TWIST1	0.0016822
FEZF1	0.0016798
CLCF1	0.0016679
CD248	0.0016121
FKBP9	0.0015888
TUBA1C	0.0015583
HOXB13	0.0015575
MMP9	0.0015481
FAM20C	0.0015445
SDC1	0.0015131
CD70	0.0015031
CAVIN3	0.0014877
FAM20A	0.0014828
PBK	0.0014662
FCGR2B	0.0014616
PTTG1	0.0014509
TNFRSF12A	0.0014115
FGFBP2	0.0012573
SPON2	0.0012140
IL10	0.0012005
COL5A1	0.0011849
TUBB6	0.0011846
COL8A1	0.0011767
PLA2G5	0.0011626
FBLIM1	0.0011228
SRPX2	0.0010772
CTSK	0.0010118
ADM	0.0010081
CENPM	0.0009951
IGLL5	0.0009822
LOX	0.0009814
F2RL2	0.0009687
KRT7	0.0009598
AQP9	0.0009371
SERPINA1	0.0009191
MDK	0.0009182
ICAM3	0.0009146

HMOX1	0.0008212
CA9	0.0008038
CDKN3	0.0008021
LDHA	0.0007943
HK3	0.0007841
PLAT	0.0007611
HSPG2	0.0007561
GPRC5A	0.0007480
IGFBP5	0.0007333
RUNX1	0.0007332
CAPG	0.0007231
CKS2	0.0007134
C1R	0.0007110
FAP	0.0007039
CARD16	0.0006962
S100A11	0.0006937
CRIP1	0.0006834
SPOCD1	0.0006744
LYZ	0.0006659
BGN	0.0006529
LAMC1	0.0006522
ANXA2	0.0006499
CD52	0.0006293
MMP14	0.0006128
LY96	0.0006002
CLIC1	0.0005587
PHLDA2	0.0005308
ANPEP	0.0005164
MGP	0.0005120
COL6A2	0.0005010
METTL1	0.0005007
COL1A1	0.0004979
PDGFD	0.0004913
LGALS3	0.0004847
SERPING1	0.0004818
AC087632.1	0.0004649
IFI30	0.0004532
POSTN	0.0004495
FABP5	0.0004385
CNPY1	0.0004380
LUM	0.0004376
TYMP	0.0004365
S100A4	0.0004266
RARRES2	0.0004233
TCIM	0.0004224
CCNB1	0.0004157

AEBP1	0.0004046
SERPINH1	0.0004034
CA3	0.0003880
PLP2	0.0003802
SNAI2	0.0003708
PDLIM1	0.0003676
CCL20	0.0003609
NID2	0.0003539
SERPINE1	0.0003402
PRC1	0.0003356
CCL18	0.0003255
TFF3	0.0003252
EMP3	0.0003185
CFB	0.0003115
PCLAF	0.0003067
SLC11A1	0.0003035
IGFBP2	0.0003021
HILPDA	0.0002923
MSR1	0.0002845
TIMP1	0.0002829
FN1	0.0002794
C21orf62	0.0002657
DCN	0.0002642
MOXD1	0.0002545
PIMREG	0.0002385
MAP3K7CL	0.0002384
MARCH9	0.0002209
LGALS1	0.0002137
TOP2A	0.0002120
SLC27A3	0.0002115
TMSB15A	0.0002019
PLAU	0.0001932
PLVAP	0.0001886
ANXA1	0.0001718
TMSB10	0.0001592
CCL2	0.0001564
TYMS	0.0001558
RAB34	0.0001548
FMOD	0.0001462
TGFBI	0.0001454
APOL4	0.0001332
IGFBP3	0.0001331
COL1A2	0.0001237
TM4SF1	0.0001174
MS4A6A	0.0001156
ALOX5AP	0.0001148

HAMP	0.0001139
SPRY4	0.0001124
TSPAN31	0.0001118
HLA-DQB1	0.0001008
AC008764.4	0.0000952
IL1R2	0.0000926
F13A1	0.0000867
METTL7B	0.0000754
S100A9	0.0000738
CXCL8	0.0000730
HIST2H2AA3	0.0000683
C5AR1	0.0000617
CD163	0.0000598
APCDD1L	0.0000577
SPP1	0.0000535
TAGLN2	0.0000521
WISP1	0.0000510
PLIN2	0.0000508
G0S2	0.0000456
IL4I1	0.0000443
CHI3L1	0.0000438
ESM1	0.0000414
PI3	0.0000387
APOC2	0.0000374
CASP4	0.0000322
GPNMB	0.0000315
SERPINA3	0.0000215
VEGFA	0.0000204
CDK4	0.0000182
COL4A1	0.0000179
SLPI	0.0000150
HLA-DRA	0.0000084
PCOLCE	0.0000082
VIM	0.0000072
TAGLN	0.0000046
HOPX	-0.0000035
SOD2	-0.0000057
PDPN	-0.0000074
COL4A2	-0.0000105
LTF	-0.0000112
CPVL	-0.0000178
NNMT	-0.0000184
MEST	-0.0000232
EMILIN3	-0.0000265
CHI3L2	-0.0000280
FABP7	-0.0000353

FCGR3A	-0.0000357
TREM1	-0.0000608
ACE	-0.0000692
CDKN2C	-0.0000772
MEOX2	-0.0000993
SOCS2	-0.0001031
FCGBP	-0.0001367
FAM183A	-0.0001417
FSTL1	-0.0001434
MFAP2	-0.0001444
ADGRE5	-0.0001705
PLA2G2A	-0.0001756
GBP1	-0.0001756
EGFR	-0.0002695
CAMP	-0.0003299
PLSCR1	-0.0003793
GRB14	-0.0005072
HP	-0.0005364
DPP4	-0.0005646
NIPSNAP2	-0.0005833
SHOX2	-0.0005901
PKIB	-0.0007954
NCAPH	-0.0008746
NOS2	-0.0008881
BARX1	-0.0010333
KLHL4	-0.0016877
TEAD2	-0.0026009
RAD54L	-0.0039478
ZNF474	-0.0065595

Table S7. Functional annotation of genes associated with glioblastoma patients' overall survival based on MSigDB Hallmarks.

hallmark	genes.in.list	total.genes	FDR	genes
EPITHELIAL MESENCHYMAL TRANSITION	48	197	3.06E-49	COL5A1 COL1A1 FN1 SERPINE1 COL1A2 COL4A1 COL4A2 IGFBP3 TGFBI LUM LAMC1 LOX TAGLN COL6A2 NNMT MMP2 FAP BGN SERPINH1 FSTL1 POSTN SPP1 NID2 VIM LOXL1 TIMP1 EMP3 ITGA5 MGP VEGFA SDC1 PCOLCE MEST EFEMP2 IGFBP2 ANPEP MMP14 TNFRSF12A FMOD PLAUR IL6 PTX3 DKK1 SNAI2 DCN LGA[illegible]
COAGULATION	18	136	2.55E-13	MMP14 C1R SERPINE1 SERPING1 MMP2 MMP9 THBD MMP11 TIMP1 PLAU PLAT FN1 F2RL2 CTSK ANXA1 APOC2 DPP4 ANG
HYPOXIA	20	194	5.48E-13	ADM VEGFA LDHA SERPINE1 LOX IGFBP3 ISG20 STC1 PLIN2 HMOX1 CAV1 PLAUR S100A4 EGFR BGN ANXA2 TGFBI DCN IL6 COL5A1
KRAS SIGNALING UP	20	194	5.48E-13	G0S2 SLPI MMP11 MMP9 IGFBP3 SPP1 LY96 ACE PLAUR PLAT LIF CCL20 HOXD11 KCNN4 SERPINA3 MPZL2 F13A1 PLVAP PLAU GPNMB
APOPTOSIS	16	159	2.20E-10	CASP4 TNFRSF12A TIMP1 LGALS3 LUM HMOX1 MMP2 PLAT DCN SOD2 BGN IL6 CAV1 ANXA1 TOP2A ISG20
COMPLEMENT	17	195	4.33E-10	C1R SERPINE1 MMP14 SERPING1 TIMP1 DPP4 PLAUR CASP4 FN1 S100A9 PLAT LTF PLSCR1 COL4A2 ANG IL6 LGALS3
INFLAMMATORY RESPONSE	17	197	4.36E-10	CCL2 CCL20 IL6 AQP9 LIF ADM PLAUR MMP14 TIMP1 OSMR IL10 SERPINE1 PDPN ITGA5 MSR1 C5AR1 EMP3
ANGIOGENESIS	9	36	7.40E-10	POSTN FSTL1 STC1 VEGFA THBD SPP1 S100A4 TIMP1 LUM
TNFA SIGNALING VIA NFKB	14	198	1.86E-07	CCL20 PLAUR SOD2 IL6 LIF PTX3 FOSL1 CCL2 SOCS3 SERPINE1 PLAU G0S2 VEGFA CLCF1
GLYCOLYSIS	14	198	1.86E-07	VEGFA LDHA SDC1 IGFBP3 EGFR SPAG4 SLC16A3 COL5A1 TFF3 STC1 ISG20 DCN ANG TGFBI
INTERFERON GAMMA RESPONSE	13	198	1.20E-06	PLSCR1 ISG20 SOCS3 SERPING1 SOD2 IFI30 CCL2 SECTM1 UPP1 IL6 CASP4 C1R METTL7B
IL2 STAT5 SIGNALING	11	195	3.77E-05	SOCS2 IL10 LIF CAPG SPP1 IL1R2 HOPX PLSCR1 PLIN2 F2RL2 SPRY4
ALLOGRAFT REJECTION	11	199	4.21E-05	IL10 HLA-DRA IL6 TIMP1 MMP9 EGFR FCGR2B CCL2 LIF CAPG NOS2
UV RESPONSE DN	9	141	7.62E-05	ANXA2 RUNX1 SERPINE1 COL1A2 CAV1 LAMC1 COL1A1 IGFBP5 SNAI2
IL6 JAK STAT3 SIGNALING	7	87	1.28E-04	SOCS3 OSMR IL6 IL1R2 TNFRSF12A HMOX1 PLA2G2A
ESTROGEN RESPONSE LATE	10	198	1.79E-04	TFF3 ISG20 SERPINA3 CAV1 S100A9 FABP5 MEST MDK TOP2A LTF
CHOLESTEROL HOMEOSTASIS	6	73	3.46E-04	LGALS3 PLSCR1 TNFRSF12A PLAUR FABP5 S100A11
G2M CHECKPOINT	9	195	7.03E-04	TOP2A PRC1 CKS2 PTTG1 CDKN3 CDKN2C RAD54L CDK4 PBK
E2F TARGETS	8	196	3.10E-03	CKS2 TOP2A RRM2 CDKN3 CENPM CDKN2C PTTG1 CDK4

FDR: False Discovery Rate.

Table S8. Functional annotation of genes associated with glioblastoma patients' overall survival based on Gene Ontology (GO) terms for biological processes.

GO.group	GO.term	genes.in.list	total.genes	FDR	genes
inflammatory response	Cellular response to acid chemical	12	218	6.59E-05	VEGFA TEAD2 MMP2 HAMP HOXA2 COL1A1 CCNB1 CDK4 EGFR COL1A2 PDGFD COL4A1
inflammatory response	Inflammatory response	45	856	5.33E-16	CCL2 CCL20 PLA2G5 ANXA1 CXCL8 C5AR1 HP CCL18 OSMR NOS2 IL6 IL10 SERPING1 S100A9 SLC11A1 TIMP1 HAMP RARRES2 FN1 IL1R2 SDC1 SPP1 TREM1 ALOX5AP CHI3L1 EGFR LY96 PBK CD163 SOCS3 SERPINA3 CASP4 SERPINA1 SERPINE1 HMOX1 FCGR2B PLSCR1 LYZ MMP9 ADGRE5 HSPG2 ADM C1R PTX3 PLA2
inflammatory response	Negative regulation of catalytic activity	28	848	4.80E-06	GPRC5A TIMP1 CAV1 SERPINE1 ANXA1 PKIB SERPING1 SERPINH1 CCDC8 SPRY4 SERPINA3 SERPINA1 LGALS3 LTF VEGFA SPOCD1 PTX3 CARD16 HP CDKN2C PI3 SLPI PTTG1 ANXA2 SOCS3 APOC2 PLAUR MMP9
inflammatory response	Regulation of response to external stimulus	39	903	2.86E-11	RARRES2 VEGFA PLAU PLA2G5 ANXA1 ANXA2 CCL18 FAP SERPINE1 OSMR NOS2 LTF SNAI2 CCL2 IL6 SERPING1 PDPN S100A9 CARD16 IL1R2 SPP1 ALOX5AP IL10 EGFR LY96 FABP7 PBK PDGFD THBD SOCS3 C5AR1 CXCL8 CAV1 FCGR2B PLAUR MMP9 PLAT C1R PLA2G2A
inflammatory response	Regulation of response to stimulus	105	4820	1.62E-11	LGALS1 HMOX1 SECTM1 GPRC5A RARRES2 DKK1 CCL2 VEGFA CCL20 IGFBP2 IGFBP5 SOCS2 PLAU G0S2 PLA2G5 ANXA1 IGFBP3 F2RL2 ESM1 VASN PDGFD TCIM ANXA2 SOCS3 SPRY4 PLA2G2A C5AR1 IGLL5 CCL18 FAP CAV1 SERPINE1 GPNMB IL6 OSMR TNFRSF12A NOS2 DCN LTF SNAI2 FCGR2B KCNN4 COL1A1 FN1 I
inflammatory response	Response to abiotic stimulus	40	1309	1.51E-07	MMP2 COL1A1 VEGFA ANXA1 EGFR PCLAF TCIM DPP4 CARD16 CRIP1 ANG NOS2 DCN SNAI2 RAD54L HMOX1 PLAT HAMP CAV1 CA9 PDLIM1 MDK IGFBP2 TWIST1 PLAU POSTN CCNB1 CDK4 ADM MMP14 STC1 PBK VASN FOSL1 THBD CHI3L1 HILPDA STRA6 AQP9 IKBIP
inflammatory response	Response to acid chemical	15	360	1.31E-04	VEGFA TEAD2 MMP2 HAMP HOXA2 DKK1 COL1A1 IGFBP2 CCNB1 CDK4 EGFR COL1A2 PDGFD TYMS COL4A1
inflammatory response	Response to biotic stimulus	38	1085	1.35E-08	NOS2 SLC11A1 LYZ HAMP CAMP CXCL8 IGLL5 LTF CAV1 CCL2 SLPI IL6 IL10 LY96 S100A9 ISG20 CARD16 DCN FAM20A CCL20 GBP1 LOXL1 CDK4 ADM SPON2 PTX3 CA3 THBD PLSCR1 C5AR1 HP SERPINE1 VIM RARRES2 FCGR2B PI3 FOSL1 PLA2G2A
inflammatory response	Response to cytokine	51	1372	1.87E-12	TIMP1 CCL2 CCL20 PLA2G5 ANXA1 CXCL8 CCL18 GBP1 PLVAP IL6 OSMR CASP4 NOS2 SLC11A1 VIM HAMP CAV1 PCOLCE COL1A1 LOX IL1R2 CHI3L1 POSTN CDK4 FOSL1 TYMS SOCS3 SPRY4 PLSCR1 CARD16 PLP2 CLCF1 TNFRSF12A MMP2 HMOX1 MMP9 SOD2 VEGFA FN1 SDC1 SOCS2 TWIST1 F13A1 LIF IL10 RUNX1 C
inflammatory response	Response to drug	30	1082	4.44E-05	ANXA1 STRA6 HMOX1 CAV1 IL6 HP NOS2 SLC11A1 RAD54L LGALS1 HAMP CA9 COL1A1 MDK LOX IGFBP2 SDC1 CCNB1 CDK4 IL10 EGFR PLIN2 PDPN CA3 NNMT PDGFD FOSL1 TYMS APOC2 VIM
inflammatory response	Response to endogenous stimulus	49	1704	2.18E-08	NOS2 TIMP1 SOCS2 ANXA1 VASN SOCS3 SPRY4 SNAI2 CAV1 RARRES2 IGFBP5 SPP1 IL6 IL10 EGFR COL1A2 ANG MMP2 CA9 DKK1 COL1A1 MDK LOX IGFBP2 SDC1 MMP19 POSTN COL4A2 CDK4 ADM MMP14 STC1 HOXB13 PDGFD FOSL1 TYMS FAM20C ANXA2 COL4A1 AQP9 CCL2 CXCL8 VIM GRB14 CASP4 FCGR2B FMOD
inflammatory response	Response to external stimulus	81	2561	1.32E-16	NOS2 SLC11A1 LYZ HAMP RARRES2 CCL2 COL1A1 VEGFA CCL20 PLAU PLA2G5 ANXA1 CAMP CXCL8 ANXA2 IGLL5 CCL18 FAP SERPINE1 IL6 OSMR LTF SNAI2 CAV1 SPP1 SLPI LGALS3 POSTN IL10 SERPING1 LY96 PDPN S100A9 ISG20 CARD16 DCN TYMP HMOX1 HOXA2 FAM20A MDK LOX IGFBP2 IL1R2 GBP1 FEZF1 LOXL
inflammatory response	Response to inorganic substance	20	586	9.90E-05	ANXA1 MMP9 AQP9 ALOX5AP IL6 CRIP1 HP HMOX1 HAMP CAV1 COL1A1 SOD2 IGFBP2 SDC1 CCNB1 CDK4 IL10 EGFR PDGFD FOSL1
inflammatory response	Response to organophosphorus	10	137	3.64E-05	PLA2G5 IGFBP5 COL1A1 SDC1 MMP19 STC1 FOSL1 THBD AQP9 TYMS
inflammatory response	Response to oxygen levels	22	408	3.94E-08	MMP2 VEGFA DPP4 CARD16 ANG NOS2 HMOX1 PLAT CAV1 CA9 PDLIM1 COL1A1 TWIST1 PLAU POSTN CCNB1 CDK4 ADM MMP14 STC1 VASN HILPDA
inflammatory response	Response to reactive oxygen species	13	233	2.73E-05	ANXA1 MMP9 IL6 HP MMP2 HMOX1 COL1A1 SOD2 SDC1 IL10 PDGFD FOSL1 EGFR
inflammatory response	Response to stress	114	4507	1.98E-17	CLIC1 NOS2 MMP2 LYZ HMOX1 HAMP RARRES2 CCL2 VEGFA CCL20 PLAU PLA2G5 ANXA1 PTX3 CAMP PCLAF CXCL8 ANXA2 C5AR1 IGLL5 HP CCL18 FAP CAV1 SERPINE1 OSMR TNFRSF12A LTF MMP9 DKK1 GBP1 SLPI F13A1 TOP2A IL6 IL10 EGFR SERPING1 PDPN S100A9 TCIM DPP4 CARD16 CRIP1 ANG DCN SLC11A1 VI
inflammatory response	Response to wounding	38	716	1.51E-13	CLIC1 PLAU ANXA1 ANXA2 FAP SERPINE1 TNFRSF12A F13A1 PDPN DCN SLC11A1 LGALS1 TIMP1 COL1A1 MDK LOX FN1 SDC1 SPP1 POSTN CCNB1 IL10 EGFR ADM SERPING1 F2RL2 THBD SERPINA1 ITGA5 HMOX1 CAV1 COL5A1 PLAUR PLSCR1 PLAT IL6 HOXB13 COL1A2
inflammatory response	Wound healing	32	585	1.06E-11	CLIC1 PLAU ANXA1 ANXA2 FAP SERPINE1 TNFRSF12A F13A1 PDPN DCN SLC11A1 TIMP1 COL1A1 LOX FN1 SDC1 POSTN CCNB1 EGFR SERPING1 F2RL2 THBD SERPINA1 ITGA5 HMOX1 CAV1 COL5A1 PLAUR PLSCR1 PLAT IL6 COL1A2
developmental process	Anatomical structure morphogenesis	81	2785	1.31E-14	HOXA2 FAM20A VEGFA ANXA1 COL6A2 ESM1 FAM20C ANXA2 IL6 DCN LTF HMOX1 SERPINE1 DKK1 CCL2 FN1 LIF COL5A1 COL4A2 LAMC1 ADM STC1 CXCL8 HOPX ANG TNFRSF12A SNAI2 TYMP TEAD2 FAP MMP2 SRPX2 CAV1 MEOX2 CA9 COL1A1 LOX IGFBP5 SDC1 SPP1 TWIST1 MMP19 HOXC13 FEZF1 HOXD10 CHI3L1 P
developmental process	Animal organ regeneration	9	83	5.69E-06	HMOX1 HAMP CDK4 IL10 EGFR ADM NNMT TYMS C5AR1
developmental process	Blood vessel development	46	687	3.54E-20	COL1A1 VEGFA ANXA1 ESM1 ANXA2 IL6 DCN HMOX1 COL4A2 ADM CXCL8 ANG TNFRSF12A TYMP TEAD2 FAP MMP2 SRPX2 CAV1 MEOX2 LOX FN1 MMP19 LOXL1 COL5A1 CHI3L1 IL10 HSPG2 COL8A1 MMP14 ITGA5 ANPEP PDGFD FOSL1 SOCS3 COL4A1 C5AR1 SERPINE1 TGFBI HOXB13 STRA6 COL1A2 RUNX1 TWIST1 GP
developmental process	Circulatory system development	56	1077	7.15E-20	COL1A1 VEGFA ANXA1 ESM1 ANXA2 IL6 DCN HMOX1 DKK1 SOD2 COL4A2 ADM CXCL8 ANG TNFRSF12A SNAI2 TYMP TEAD2 FAP MMP2 SRPX2 HAMP CAV1 MEOX2 LOX FN1 TWIST1 MMP19 LIF LOXL1 COL5A1 CHI3L1 CCNB1 IL10 HSPG2 COL8A1 MMP14 ITGA5 ANPEP SHOX2 PDGFD FOSL1 SOCS3 COL4A1 C5AR1 SERPINE
developmental process	Connective tissue development	19	280	1.43E-08	COL6A2 ANXA2 STC1 SNAI2 TIMP1 COL1A1 MGP LOX TGFBI COL5A1 CDK4 LUM CTSK SERPINH1 SHOX2 PDGFD TYMS CRIP1 CHI3L1
developmental process	Embryo development	34	1054	5.56E-07	HOXA2 MEOX2 DKK1 FN1 COL5A1 HOPX TEAD2 TWIST1 LIF HOXD10 TOP2A CCNB1 IL10 EGFR ADM MMP14 EN1 SHOX2 TM4SF1 FOSL1 PHLDA2 SOCS3 HOXB7 MMP2 MMP9 MFAP2 COL4A2 COL8A1 CXCL8 RARRES2 COL1A1 STRA6 ITGA5 VEGFA
developmental process	Endodermal cell differentiation	9	51	1.25E-07	DKK1 FN1 COL5A1 MMP2 MMP9 COL4A2 COL8A1 MMP14 ITGA5
developmental process	Muscle structure development	22	645	3.96E-05	DKK1 SOD2 IGFBP3 DCN LGALS1 HAMP CAV1 MEOX2 LOX IGFBP5 SDC1 TWIST1 LIF HOXD10 CCNB1 PAX3 ADM MMP14 SHOX2 STRA6 VEGFA TAGLN
developmental process	Negative regulation of developmental process	31	1017	5.95E-06	ANXA1 ANXA2 DCN DKK1 COL5A1 COL4A2 HOPX VIM MMP11 LGALS1 MMP9 CAV1 HOXA2 IGFBP5 SPP1 SOCS2 TWIST1 POSTN CTSK SERPINE1 MEOX2 SNAI2 TIMP1 GBP1 EGFR VASN LTF RUNX1 IL6 FCGR2B HSPG2
developmental process	Ossification	22	396	2.40E-08	COL1A1 SPP1 LTF MMP2 MMP9 HOXA2 DKK1 MGP LOX IGFBP5 TWIST1 GPNMB CTSK IGFBP3 MMP14 STC1 COL1A2 SHOX2 FAM20C SNAI2 EGFR IL6
developmental process	Osteoblast differentiation	13	218	1.45E-05	SPP1 LTF HOXA2 COL1A1 LOX IGFBP5 TWIST1 GPNMB IGFBP3 SHOX2 FAM20C SNAI2 IL6
developmental process	Regeneration	13	204	7.68E-06	ANXA1 HMOX1 HAMP SPP1 POSTN CCNB1 CDK4 IL10 EGFR ADM NNMT TYMS C5AR1
developmental process	Regulation of developmental process	74	2763	1.84E-11	VEGFA LIF ANXA1 ANXA2 IL6 ACE DCN LTF HMOX1 SRPX2 SERPINE1 DKK1 CCL2 COL1A1 MDK SOD2 COL5A1 COL4A2 IGFBP3 CXCL8 HOPX TNFRSF12A SNAI2 VIM MSR1 TEAD2 MMP11 LGALS1 MMP9 HAMP CAV1 HOXA2 MGP LOX FN1 IGFBP5 SPP1 SOCS2 TWIST1 FEZF1 CHI3L1 POSTN CCNB1 CDK4 IL10 CTSK ADM MMF
developmental process	Regulation of multicellular organismal process	83	3382	3.93E-11	VEGFA SPP1 PLAU ANXA1 ANXA2 CARD16 APOC2 FAP CAV1 SERPINE1 IL6 ACE NOS2 DCN LTF HMOX1 SRPX2 DKK1 COL1A1 MDK SOD2 FN1 LIF COL5A1 COL4A2 HILPDA GPNMB IL10 ADM STC1 PDPN CXCL8 TNFRSF12A SLC11A1 SNAI2 VIM LGALS1 MMP9 HAMP HOXA2 MGP LOX IGFBP5 IL1R2 SOCS2 TWIST1 G0S2 FEZF
developmental process	Reproductive structure development	20	445	2.61E-06	ANXA1 DCN SDC1 MMP19 LIF IL10 EGFR ADM MMP14 STC1 HOXB13 PTX3 FOSL1 PHLDA2 SOCS3 CRIP1 STRA6 VEGFA ANG SPP1
developmental process	Reproductive system development	20	449	2.88E-06	ANXA1 DCN SDC1 MMP19 LIF IL10 EGFR ADM MMP14 STC1 HOXB13 PTX3 FOSL1 PHLDA2 SOCS3 CRIP1 STRA6 VEGFA ANG SPP1
developmental process	Skeletal system development	26	541	1.54E-08	HOXA2 COL1A1 COL6A2 MMP14 ANXA2 LTF STC1 SNAI2 MMP2 MMP9 TIMP1 MGP LOX TGFBI TWIST1 HOXD10 LUM CTSK SERPINH1 SHOX2 TYMS FAM20C HOXB7 COL1A2 CHI3L1 EN1
developmental process	Tissue development	77	2168	2.47E-18	MEOX2 FAM20A BARX1 ANXA1 COL6A2 FAM20C ANXA2 SERPINE1 DKK1 COL1A1 FN1 LIF COL5A1 IL6 TAGLN TAGLN2 STC1 DCN SNAI2 VIM TEAD2 MMP9 TIMP1 HAMP CAV1 CA9 MGP LOX IGFBP5 SDC1 SPP1 TGFBI TWIST1 HOXC13 HOXD10 POSTN CCNB1 CDK4 GPNMB IL10 LUM CTSK EGFR ADM SERPINH1 MMP14 HOXE
developmental process	Tissue remodeling	14	167	1.51E-07	MMP2 SPP1 ANXA1 ACE GPNMB TIMP1 CAV1 IGFBP5 LIF CTSK EGFR MMP14 BGN IL6
developmental process	Tube development	51	1062	1.32E-16	VEGFA ANXA1 ESM1 ANXA2 IL6 DCN HMOX1 LIF COL4A2 ADM CXCL8 ANG TNFRSF12A TYMP TEAD2 FAP MMP2 SRPX2 CAV1 MEOX2 LOX FN1 IGFBP5 SDC1 TWIST1 MMP19 CHI3L1 CCNB1 IL10 HSPG2 COL8A1 EGFR MMP14 ITGA5 EN1 ANPEP SHOX2 TYMS COL4A1 C5AR1 HOXB7 SERPINE1 TGFBI HOXB13 RARRES2 STRA
developmental process	Tube morphogenesis	43	860	1.59E-14	VEGFA ANXA1 ESM1 ANXA2 IL6 DCN HMOX1 COL4A2 ADM CXCL8 ANG TNFRSF12A TYMP TEAD2 FAP MMP2 SRPX2 CAV1 MEOX2 LOX FN1 TWIST1 MMP19 CHI3L1 IL10 HSPG2 COL8A1 EGFR MMP14 ITGA5 ANPEP SHOX2 COL4A1 C5AR1 HOXB7 SERPINE1 TGFBI HOXB13 STRA6 HOXD11 RUNX1 GPNMB CCL2
developmental process	Urogenital system development	14	346	3.15E-04	ANXA1 LIF DCN MMP9 SDC1 HOXB13 ACE PDGFD HOXB7 CRIP1 STRA6 COL4A1 VEGFA HOXD11
cell comunication	Cell surface receptor signaling pathway	84	3287	3.38E-12	GPRC5A PLAT DKK1 CCL2 COL1A1 VEGFA CCL20 IGFBP2 IGFBP5 SOCS2 G0S2 LIF COL4A2 ANXA1 TSPAN31 EGFR IGFBP3 ESM1 VASN CXCL8 PDGFD CLCF1 TCIM SOCS3 SPRY4 C5AR1 IGLL5 CCL18 DCN LTF SNAI2 FCGR2B KCNN4 CAV1 RARRES2 PLVAP LGALS3 IL6 COL1A2 CASP4 CARD16 TNFRSF12A PLAUR VIM LOX I
cell comunication	Intracellular signal transduction	58	3113	3.69E-04	LGALS1 HMOX1 SECTM1 NOS2 TEAD2 CCL2 RAB34 CCL20 ADGRE5 PLA2G5 F2RL2 PDGFD SPRY4 PLA2G2A CCL18 GPNMB IL6 DCN LTF DKK1 VEGFA FN1 LIF PLVAP IL10 EGFR ADM S100A9 COL1A2 CXCL8 CLCF1 TCIM S100A4 C5AR1 CARD16 CRIP1 CAV1 LOX IGFBP5 SOCS2 CHI3L1 IGFBP3 PBK CAVIN3 SOCS3 PLAUR SN
cell comunication	Regulation of cell communication	84	3903	1.37E-08	LGALS1 HMOX1 SECTM1 GPRC5A DKK1 CCL2 CCL20 IGFBP2 IGFBP5 SOCS2 G0S2 PLA2G5 ANXA1 IGFBP3 F2RL2 ESM1 VASN PDGFD TCIM SOCS3 SPRY4 PLA2G2A CCL18 GPNMB IL6 DCN LTF SNAI2 KCNN4 CAV1 SERPINE1 COL1A1 VEGFA FN1 SPP1 PLAU LIF LGALS3 IL10 EGFR ADM S100A9 CXCL8 CLCF1 S100A4 CASP
cell comunication	Regulation of signaling	85	3952	1.10E-08	LGALS1 HMOX1 SECTM1 GPRC5A DKK1 CCL2 CCL20 IGFBP2 IGFBP5 SOCS2 G0S2 PLA2G5 ANXA1 IGFBP3 F2RL2 ESM1 VASN PDGFD TCIM ANXA2 SOCS3 SPRY4 PLA2G2A CCL18 GPNMB IL6 DCN LTF SNAI2 KCNN4 CAV1 SERPINE1 COL1A1 VEGFA FN1 SPP1 PLAU LIF LGALS3 IL10 EGFR ADM S100A9 CXCL8 CLCF1 S100A
cell comunication	Regulation of signaling receptor activity	26	676	8.78E-07	GPRC5A SERPINE1 PLAU TYMP TIMP1 HAMP DKK1 CCL2 MDK VEGFA CCL20 SPP1 LIF GPNMB IL6 IL10 SECTM1 ADM STC1 CXCL8 PDGFD CLCF1 PHLDA2 TNFSF12-TNFSF13 CCL18 FCGR2B
metabolic process	Collagen metabolic process	13	113	1.48E-08	MMP2 MMP11 MMP9 MMP19 MMP14 FAP IL6 CTSK SERPINH1 COL1A2 VIM COL1A1 COL5A1
metabolic process	Glycosaminoglycan catabolic process	6	61	4.87E-04	DCN SDC1 FMOD LUM HSPG2 BGN
metabolic process	Phosphorus metabolic process	66	3597	1.64E-04	NOS2 GPRC5A CAV1 CCL2 VEGFA CCL20 SOCS2 PLA2G5 CCNB1 CDK4 PKIB HK3 CCDC8 PDGFD RRM2 FAM20C ANXA2 SOCS3 SPRY4 PLA2G2A APOC2 AC087632.1 CCL18 LGALS3 GPNMB LTF SRPX2 RARRES2 DKK1 FAM20A FN1 CDKN2C LIF PLVAP SPOCD1 IL6 EGFR IGFBP3 CLCF1 TYMS TCIM C5AR1 ANG SLC11A1 CDK
metabolic process	Positive regulation of cellular metabolic process	72	3482	1.20E-06	NOS2 TEAD2 HOXA2 MEOX2 CCL2 VEGFA CCL20 PLA2G5 ANXA1 PDGFD ANXA2 PLA2G2A CASP4 APOC2 CCL18 CAV1 GPNMB DCN LTF RARRES2 DKK1 COL1A1 FAM20A SPP1 TWIST1 LIF CCNB1 PAX3 IL6 IL10 EGFR RUNX1 S100A9 CLCF1 FOSL1 TCIM PLSCR1 C5AR1 ANG HOXB7 GPRC5A SLC11A1 SNAI2 HMOX1 PCOLCE
metabolic process	Regulation of phosphorus metabolic process	53	1872	8.01E-09	NOS2 GPRC5A CAV1 CCL2 VEGFA CCL20 PLA2G5 CCNB1 PKIB CCDC8 PDGFD ANXA2 SPRY4 PLA2G2A APOC2 CCL18 LGALS3 GPNMB LTF SRPX2 RARRES2 DKK1 FAM20A FN1 CDKN2C LIF SPOCD1 IL6 EGFR IGFBP3 CLCF1 TCIM C5AR1 ANG SLC11A1 LOX SOCS2 CKS2 CHI3L1 CDK4 ACE PBK CAVIN3 FAM20C SOCS3 PLAU
immune system process	Leukocyte migration	28	491	1.06E-10	CCL2 VEGFA CCL20 ANXA1 CXCL8 CCL18 IL6 LGALS3 S100A9 MMP9 MMP14 PDGFD C5AR1 SERPINE1 RARRES2 PLVAP HMOX1 CAV1 COL1A1 GRB14 FN1 SDC1 TREM1 IL10 SLC16A3 ITGA5 COL1A2 THBD
immune system process	Immune system process	95	3539	1.81E-15	RARRES2 CCL2 VEGFA CCL20 PLA2G5 ANXA1 RUNX1 PTX3 CAMP CXCL8 CLCF1 TCIM ANXA2 C5AR1 IGLL5 CCL18 IL6 ACE LTF FCGR2B KCNN4 CAV1 IGFBP2 GBP1 SLPI LIF LGALS3 GPNMB IL10 SERPING1 S100A9 DPP4 HLA-DRA NOS2 SLC11A1 VIM LGALS1 HMOX1 MMP9 LOX BARX1 TOP2A C1RL SECTM1 LY96 MMP14 C
immune system process	Humoral immune response	21	312	2.59E-09	CAMP CXCL8 IGLL5 IL6 LTF SLPI SERPING1 S100A9 C1RL C1R SPON2 CFB RARRES2 FCGR2B SLC11A1 LYZ CCL2 PI3 TREM1 PLA2G2A C5AR1
regulation of proteolysis	Peptide cross-linking	6	63	5.61E-04	FN1 PI3 F13A1 ANXA1 DCN BGN
regulation of proteolysis	Proteolysis	42	1988	3.41E-04	TIMP1 PLAT CPVL SERPINE1 AEBP1 PLAU C1RL CTSK SERPING1 SERPINH1 MMP14 C1R ANPEP ANXA2 SERPINA3 CASP4 SERPINA1 CAV1 ACE LTF FAP MMP2 MMP9 VEGFA IL10 S100A9 DPP4 CARD16 PLAUR MMP11 PCOLCE FN1 IL1R2 MMP19 PI3 SLPI PTTG1 PBK CFB HP CCNB1 C5AR1
regulation of proteolysis	Regulation of peptidyl-tyrosine autophosphorylation	3	6	2.34E-04	VEGFA CAV1 ACE
regulation of proteolysis	Regulation of protein metabolic process	71	3006	1.10E-08	GPRC5A TIMP1 CAV1 SERPINE1 CCL2 VEGFA CCL20 PLA2G5 CCNB1 PKIB SERPING1 SERPINH1 PDGFD ANXA2 SPRY4 PLA2G2A SERPINA3 CASP4 SERPINA1 CCL18 LGALS3 GPNMB LTF PLAT RARRES2 DKK1 FAM20A FN1 IGFBP5 CDKN2C LIF IL6 IL10 EGFR IGFBP3 ACE S100A9 PTX3 CLCF1 TYMS TCIM C5AR1 CARD16 NC
regulation of proteolysis	Regulation of proteolysis	27	853	1.49E-05	TIMP1 SERPINE1 SERPING1 SERPINH1 ANXA2 SERPINA3 CASP4 SERPINA1 CAV1 LTF PLAT VEGFA IL10 S100A9 CARD16 PLAUR PCOLCE FN1 IL1R2 PI3 SLPI MMP14 PTTG1 PBK MMP9 C1R C5AR1
cell motility	Localization of cell	58	1670	4.51E-13	TMSB10 PLAT CCL2 VEGFA CCL20 SDC1 ANXA1 TMSB15A CXCL8 PDGFD CD248 CCL18 IL6 DCN FAP SRPX2 SERPINE1 COL1A1 FN1 IGFBP5 PLAU LGALS3 GPNMB IGFBP3 MMP14 STC1 S100A9 DPP4 TNFRSF12A SNAI2 MMP9 MDK LOX TWIST1 FEZF1 POSTN EGFR ITGA5 PHLDA2 C5AR1 HMOX1 MEOX2 TIMP1 CAV1 RARRE
cell motility	Locomotion	64	1921	1.07E-13	TMSB10 PLAT RARRES2 CCL2 VEGFA CCL20 SDC1 ANXA1 TMSB15A CXCL8 PDGFD CD248 CCL18 IL6 DCN FAP SRPX2 SERPINE1 COL1A1 FN1 IGFBP5 PLAU LGALS3 GPNMB IGFBP3 MMP14 STC1 S100A9 DPP4 TNFRSF12A SNAI2 TYMP MMP9 HOXA2 MDK LOX TWIST1 FEZF1 POSTN EGFR ITGA5 PHLDA2 C5AR1 HMOX1 ME
cell motility	Movement of cell or subcellular component	61	2139	2.55E-10	TMSB10 PLAT CCL2 VEGFA CCL20 SDC1 ANXA1 TMSB15A CXCL8 PDGFD CD248 CCL18 IL6 DCN FAP SRPX2 SERPINE1 COL1A1 FN1 IGFBP5 PLAU LGALS3 GPNMB IGFBP3 MMP14 STC1 S100A9 DPP4 TNFRSF12A SNAI2 MMP9 CAV1 HOXA2 MDK LOX TWIST1 FEZF1 POSTN EGFR ITGA5 PHLDA2 C5AR1 HMOX1 MEOX2 TIMP
cell motility	Regulation of localization	62	2905	3.89E-06	TMSB10 MSR1 HAMP MEST VEGFA ANXA1 TMSB15A PDGFD ANXA2 CARD16 CAV1 DCN MMP9 SERPINE1 DKK1 CCL2 COL1A1 CCL20 FN1 IGFBP5 SPP1 PLAU LIF LGALS3 HILPDA GPNMB IL6 IL10 EGFR IGFBP3 MMP14 STC1 ANG APOC2 SLC11A1 HMOX1 SRPX2 KCNN4 IL1R2 TWIST1 POSTN ITGA5 PTX3 FABP5 PHLDA2 C5A
extracellular matrix organization	Cellular component disassembly	18	573	6.32E-04	CTSK TOP2A FAP PDPN DPP4 SNAI2 CAPG LAMC1 IL6 MMP2 MMP11 MMP9 TIMP1 CAV1 DKK1 MMP19 CCNB1 MMP14
extracellular matrix organization	Extracellular matrix organization	40	392	1.23E-23	MMP2 MMP11 MMP9 COL1A1 TGFBI MMP19 COL5A1 POSTN COL4A2 COL8A1 SERPINH1 MMP14 COL1A2 ANXA2 COL4A1 FAP AEBP1 PDPN DPP4 LOX FMOD LUM PTX3 EFEMP2 LAMC1 IL6 DCN ICAM3 NID2 TIMP1 SERPINE1 FN1 MFAP2 SPP1 LOXL1 COL6A2 HSPG2 CTSK ITGA5 BGN
extracellular matrix organization	Extracellular structure organization	44	460	8.44E-25	MMP2 MMP11 MMP9 COL1A1 TGFBI MMP19 PLA2G5 COL5A1 POSTN COL4A2 COL8A1 SERPINH1 MMP14 COL1A2 ANXA2 COL4A1 APOC2 FAP AEBP1 PDPN DPP4 LOX FMOD LUM PTX3 EFEMP2 SDC1 LAMC1 IL6 DCN ICAM3 NID2 TIMP1 SERPINE1 FN1 MFAP2 SPP1 LOXL1 COL6A2 HSPG2 CTSK ITGA5 BGN PLA2G2A
extracellular matrix organization	Negative regulation of cellular component organization	22	749	3.15E-04	TMSB10 TMSB15A PTTG1 FAP DKK1 LGALS3 DPP4 APOC2 VIM CAPG LGALS1 SPP1 TWIST1 LIF POSTN MMP14 ACE CAV1 GBP1 TOP2A CCNB1 FCGR2B
extracellular matrix organization	Regulation of cellular component organization	55	2653	3.84E-05	TMSB10 CCNB1 ANXA1 TMSB15A PTTG1 CCDC8 ANXA2 APOC2 FAP SRPX2 CAV1 SERPINE1 AEBP1 DKK1 CCL2 VEGFA TWIST1 CDKN2C LIF LGALS3 EGFR MMP14 PDPN FOSL1 DPP4 TNFRSF12A SLC11A1 SNAI2 VIM CAPG LGALS1 HAMP FN1 IGFBP5 SPP1 SOCS2 COL5A1 POSTN IL10 IGFBP3 ACE FBLIM1 PTX3 SHOX2 DCI
lipid transport	Import into cell	24	887	4.85E-04	RAB34 ANXA1 ANXA2 IGLL5 FCGR2B CAV1 SERPINE1 DKK1 VEGFA LGALS3 EGFR ADM CXCL8 APOC2 SLC11A1 MSR1 ICAM3 SPON2 PTX3 CD163 CCL2 HSPG2 FCGR3A HP
lipid transport	Lipid localization	19	409	3.08E-06	MSR1 MEST PLA2G5 ANXA1 STRA6 ANXA2 APOC2 NOS2 SPP1 HILPDA ACE APOL4 AQP9 KCNN4 CAV1 SLC27A3 PLIN2 PLA2G2A IL6
lipid transport	Lipid transport	16	367	4.29E-05	PLA2G5 ANXA1 STRA6 ANXA2 APOC2 NOS2 SPP1 ACE MSR1 APOL4 AQP9 KCNN4 SLC27A3 PLIN2 PLA2G2A CAV1
lipid transport	Monocarboxylic acid transport	10	171	2.06E-04	PLA2G5 ANXA1 STRA6 SLC16A3 NOS2 ACE AQP9 SLC27A3 PLIN2 PLA2G2A
lipid transport	Regulated exocytosis	32	901	1.85E-07	HMOX1 PLAUR LTF SLC11A1 FCGR2B LYZ MMP9 TIMP1 SERPINE1 RARRES2 VEGFA FN1 PLAU ADGRE5 SLPI F13A1 LGALS3 CHI3L1 SERPING1 TAGLN2 HK3 S100A11 S100A9 PTX3 CAMP FABP5 ANPEP ANXA2 SERPINA3 SERPINA1 C5AR1 HP
lipid transport	Vesicle-mediated transport	49	2220	2.83E-05	RAB34 ANXA1 ANXA2 IGLL5 FCGR2B CAV1 SERPINE1 DKK1 VEGFA LGALS3 EGFR ADM CXCL8 APOC2 SLC11A1 MSR1 ICAM3 HMOX1 SPON2 PTX3 CD163 SDC1 CCL2 HSPG2 PLAUR LTF LYZ MMP9 TIMP1 RARRES2 FN1 PLAU ADGRE5 SLPI F13A1 CHI3L1 SERPING1 TAGLN2 HK3 S100A11 S100A9 CAMP FABP5 ANPEP SERPI
cell proliferation	Cell population proliferation	63	2165	4.97E-11	CAV1 VEGFA LIF CCNB1 ANXA1 OSMR EGFR PDGFD CLCF1 ANXA2 ACE LTF SNAI2 HMOX1 TIMP1 SOD2 FN1 IGFBP2 IGFBP5 CDKN2C HILPDA GPNMB IL6 IL10 IGFBP3 STC1 ESM1 C5AR1 DPP4 ANG NOS2 SLC11A1 FAP MMP2 TGFBI TWIST1 PLAU CKS2 FEZF1 CDK4 COL8A1 ADM MMP14 S100A11 FABP7 SHOX2 CD248 FOSL
cell proliferation	Positive regulation of cell population proliferation	33	1022	8.42E-07	VEGFA LIF CCNB1 ANXA1 OSMR EGFR PDGFD CLCF1 ANXA2 LTF HMOX1 TIMP1 FN1 IGFBP2 HILPDA IL6 ESM1 C5AR1 DPP4 ANG MMP2 TWIST1 CDK4 IL10 ADM SHOX2 CD248 IGFBP5 MMP9 PRC1 TSPAN31 LAMC1 FOSL1
cell death	Cell death	54	2415	6.44E-06	G0S2 TOP2A ANXA1 EGFR CASP4 HP TNFRSF12A CCL2 SOD2 VEGFA LGALS3 IL6 IL10 EMP3 S100A9 CLCF1 TCIM PLSCR1 CARD16 CRIP1 SNAI2 TEAD2 FAP LGALS1 HMOX1 MMP9 TIMP1 CAV1 DKK1 MDK LOX TWIST1 CHI3L1 CDK4 IGFBP3 ADM EN1 CD248 FOSL1 SOCS3 C5AR1 GPNMB PLAUR SERPINE1 ITGA5 LTF FCGR2
cell death	Negative regulation of apoptotic process	28	966	4.19E-05	EGFR CCL2 VEGFA LGALS3 IL6 CLCF1 TCIM CARD16 SNAI2 HMOX1 TIMP1 CAV1 DKK1 MDK TWIST1 IL10 EN1 SOCS3 C5AR1 SOD2 PLAUR MMP9 SERPINE1 ITGA5 LTF PDPN SOCS2 ANXA1
cell adhesion	Biological adhesion	47	1548	1.06E-08	CLIC1 ICAM3 SPP1 TGFBI PLAU COL5A1 POSTN ANXA1 GPNMB SPON2 EMILIN3 SNAI2 LTF SRPX2 CAV1 SERPINE1 VEGFA FN1 IGFBP2 LAMC1 IL6 ITGA5 PDPN S100A9 CXCL8 DPP4 TNFRSF12A FAP NID2 LGALS1 PDLIM1 COL1A1 ADGRE5 IL10 COL6A2 COL8A1 MPZL2 MMP14 FBLIM1 S100A11 ANXA2 GBP1 EGFR CCL2 LGA
cell adhesion	Regulation of cell adhesion	27	765	2.44E-06	PLAU ANXA1 EMILIN3 SNAI2 CAV1 SERPINE1 VEGFA FN1 IGFBP2 GPNMB IL6 PDPN CXCL8 DPP4 LGALS1 COL1A1 POSTN IL10 COL8A1 MMP14 ITGA5 GBP1 CCL2 LGALS3 RUNX1 FCGR2B TGFBI
cytokine production	Cytokine production	25	925	3.54E-04	ANXA1 CARD16 NOS2 LTF FN1 HILPDA GPNMB IL6 IL10 SLC11A1 HMOX1 IL1R2 TWIST1 CHI3L1 POSTN LUM LY96 SPON2 C5AR1 SNAI2 SERPINE1 GBP1 RUNX1 FCGR2B S100A9
cytokine production	Regulation of cytokine production	23	852	6.64E-04	ANXA1 CARD16 NOS2 LTF FN1 HILPDA GPNMB IL6 IL10 SLC11A1 HMOX1 IL1R2 TWIST1 POSTN LUM LY96 SPON2 C5AR1 SNAI2 SERPINE1 GBP1 RUNX1 FCGR2B
aging	Aging	15	354	1.09E-04	DCN RAD54L TIMP1 HAMP DKK1 IGFBP2 IGFBP5 COL4A2 IL10 ADM SERPING1 TYMS SERPINE1 SOD2 TWIST1
cell activation	Cell activation	49	1591	2.92E-09	CLIC1 PLA2G5 ANXA1 CLCF1 TCIM IGLL5 CAV1 IGFBP2 GPNMB IL6 IL10 PDPN CXCL8 DPP4 SLC11A1 LGALS1 HMOX1 TIMP1 EGFR MMP14 C5AR1 LGALS3 FCGR2B CCL2 RUNX1 PLSCR1 PLAUR LTF LYZ MMP9 COL1A1 PLAU ADGRE5 SLPI CHI3L1 HK3 S100A11 S100A9 PTX3 CAMP F2RL2 FABP5 COL1A2 ANPEP THBD ANXA2

coagulation	Coagulation	18	364	2.68E-06	CLIC1 PLAU ANXA2 FAP SERPINE1 F13A1 PDPN SERPING1 F2RL2 THBD SERPINA1 CAV1 PLAUR PLSCR1 PLAT COL1A1 IL6 COL1A2
growth	Growth	31	1028	7.29E-06	ANXA1 COL6A2 ANXA2 CDKN2C EGFR MMP14 STC1 CAMP TNFRSF12A HAMP FN1 IGFBP2 IGFBP5 SPP1 SOCS2 POSTN CCNB1 CDK4 IL10 EMP3 IGFBP3 ADM HOXB13 EN1 PTX3 TYMS PHLDA2 SOCS3 STRA6 VEGFA S100A9
multi-multicellular organism process	Multi-multicellular organism process	12	239	1.53E-04	MMP2 MMP9 IGFBP2 IGFBP5 LIF ADM STC1 ITGA5 FOSL1 THBD TIMP1 SPP1
multi-organism process	Multi-organism process	68	2827	1.35E-08	NOS2 SLC11A1 LYZ HAMP CAMP CXCL8 ANXA2 IGLL5 LTF LGALS1 CAV1 CCL2 FN1 SLPI IL6 IL10 LY96 S100A9 PTX3 ISG20 CARD16 DCN VIM SPAG4 FCGR2B MMP2 MMP9 FAM20A MDK CCL20 IGFBP2 IGFBP5 GBP1 NCAPH MMP19 PI3 LIF HOXD10 LOXL1 TOP2A CCNB1 CDK4 KRT7 EGFR ADM STC1 ACE SPON2 ITGA5 EN1 C
regulation of biological quality	Regulation of biological quality	76	4319	1.36E-04	LTF LYZ CLIC1 NOS2 SLC11A1 TMSB10 MSR1 SPAG4 MOXD1 HMOX1 HAMP CAV1 MEST SPP1 PLAU ANXA1 TMSB15A ACE TFF3 HK3 F2RL2 CD52 ANXA2 PLSCR1 C5AR1 FAP SERPINE1 APOC2 SRPX2 KCNN4 CCL2 VEGFA F13A1 LIF HILPDA IL6 ADM STC1 PDPN TNFRSF12A CAPG LGALS1 DKK1 LOX FN1 IGFBP5 SOCS2 G0S
regulation of biological quality	Regulation of biological quality	76	4319	1.36E-04	LTF LYZ CLIC1 NOS2 SLC11A1 TMSB10 MSR1 SPAG4 MOXD1 HMOX1 HAMP CAV1 MEST SPP1 PLAU ANXA1 TMSB15A ACE TFF3 HK3 F2RL2 CD52 ANXA2 PLSCR1 C5AR1 FAP SERPINE1 APOC2 SRPX2 KCNN4 CCL2 VEGFA F13A1 LIF HILPDA IL6 ADM STC1 PDPN TNFRSF12A CAPG LGALS1 DKK1 LOX FN1 IGFBP5 SOCS2 G0S
regulation of body fluid levels	Regulation of body fluid levels	22	534	2.68E-06	CLIC1 PLAU ANXA2 FAP SERPINE1 F13A1 ADM PDPN KCNN4 CAV1 SOCS2 SERPING1 F2RL2 THBD SERPINA1 VEGFA PLAUR PLSCR1 PLAT COL1A1 IL6 COL1A2
regulation of molecular function	Regulation of molecular function	79	3529	1.00E-08	NOS2 GPRC5A TIMP1 CAV1 SERPINE1 CCL2 CCL20 PLA2G5 CCNB1 ANXA1 PKIB SERPING1 SERPINH1 CCDC8 PDGFD ANXA2 SPRY4 SERPINA3 CASP4 SERPINA1 APOC2 CCL18 LGALS3 PLAUR LTF MMP9 DKK1 VEGFA PLAU CDKN2C SLPI SPOCD1 IL6 IL10 EGFR S100A9 PTX3 TCIM PLSCR1 CARD16 HP SLC11A1 TYMP HM
regulation of receptor binding	Regulation of receptor binding	5	24	6.11E-05	ANXA2 MMP9 LOX PHLDA2 IL10
reproduction	Reproduction	40	1513	4.65E-06	MND1 TOP2A ANXA1 PTTG1 DCN SPAG4 MMP2 MMP9 MDK IGFBP2 IGFBP5 SDC1 NCAPH MMP19 CKS2 PI3 LIF HOXD10 CCNB1 IL10 EGFR ADM MMP14 STC1 HOXB13 ACE ITGA5 PTX3 FOSL1 THBD PHLDA2 SOCS3 RPL39L CRIP1 TIMP1 STRA6 VEGFA ANG RAD54L SPP1

FDR: False Discovery Rate.

Table S9. Functional annotation of genes associated with glioblastoma patients' overall survival based on KEGG pathways.

KEGG.pathway	genes.in.list	total.genes	FDR	genes
Proteoglycans in cancer	15	198	2.22E-07	DCN EGFR FN1 HOXD10 HSPG2 ITGA5 LUM MMP2 MMP9 PLAU PLAUR SDC1 TWIST1 VEGFA CAV1
PI3K-Akt signaling pathway	15	353	1.10E-04	CDK4 COL1A1 COL1A2 COL4A1 COL4A2 COL6A2 EGFR FN1 IL6 ITGA5 LAMC1 SPP1 VEGFA PDGFD OSMR
Pathways in cancer	15	528	4.42E-03	CDK4 CKS2 COL4A1 COL4A2 EGFR FN1 HMOX1 IL6 CXCL8 LAMC1 MMP2 MMP9 NOS2 VEGFA RUNX1
Focal adhesion	13	199	8.58E-06	COL1A1 COL1A2 COL4A1 COL4A2 COL6A2 EGFR FN1 ITGA5 LAMC1 SPP1 VEGFA PDGFD CAV1
Complement and coagulation cascades	11	78	6.94E-08	F2RL2 F13A1 SERPINE1 PLAT PLAU PLAUR CFB THBD SERPING1 C1R C5AR1
ECM-receptor interaction	11	82	8.01E-08	COL1A1 COL1A2 COL4A1 COL4A2 COL6A2 FN1 HSPG2 ITGA5 LAMC1 SDC1 SPP1
Transcriptional misregulation in cancer	10	186	4.14E-04	CDKN2C IGFBP3 IL6 CXCL8 MMP9 PAX3 PLAT PLAU IL1R2 RUNX1
Cytokine-cytokine receptor interaction	10	293	7.86E-03	CLCF1 IL6 CXCL8 IL10 LIF TNFRSF12A CCL2 CCL20 IL1R2 OSMR
HIF-1 signaling pathway	9	100	3.37E-05	EGFR HK3 HMOX1 IL6 LDHA NOS2 SERPINE1 TIMP1 VEGFA
Relaxin signaling pathway	9	129	1.37E-04	COL1A1 COL1A2 COL4A1 COL4A2 EGFR MMP2 MMP9 NOS2 VEGFA
Phagosome	8	150	1.63E-03	FCGR2B FCGR3A HLA-DRA ITGA5 MSR1 C1R TUBB6 TUBA1C
MicroRNAs in cancer	8	150	1.63E-03	EGFR HMOX1 HOXD10 ITGA5 MMP9 PLAU VEGFA VIM
IL-17 signaling pathway	7	92	5.59E-04	IL6 CXCL8 MMP9 S100A9 CCL2 CCL20 FOSL1
TNF signaling pathway	7	110	1.47E-03	IL6 LIF MMP9 MMP14 CCL2 CCL20 SOCS3
P53 signaling pathway	5	72	5.90E-03	CDK4 IGFBP3 SERPINE1 RRM2 CCNB1
AGE-RAGE signaling pathway in diabetic complications	13	100	1.17E-08	CDK4 COL1A1 COL1A2 COL4A1 COL4A2 FN1 IL6 CXCL8 MMP2 SERPINE1 CCL2 THBD VEGFA
Human papillomavirus infection	12	330	1.93E-03	CDK4 COL1A1 COL1A2 COL4A1 COL4A2 COL6A2 EGFR FN1 ITGA5 LAMC1 SPP1 VEGFA
Amoebiasis	11	96	2.64E-07	COL1A1 COL1A2 COL4A1 COL4A2 FN1 IL6 CXCL8 IL10 LAMC1 NOS2 IL1R2
Fluid shear stress and atherosclerosis	10	138	5.08E-05	HMOX1 MMP2 MMP9 PLAT CCL2 SDC1 THBD VEGFA IL1R2 CAV1
Pertussis	8	76	3.41E-05	LY96 IL6 CXCL8 IL10 ITGA5 NOS2 SERPING1 C1R
Protein digestion and absorption	8	90	8.18E-05	COL1A1 COL1A2 COL4A1 COL4A2 COL5A1 COL6A2 DPP4 KCNN4
Tuberculosis	8	177	4.33E-03	FCGR2B FCGR3A HLA-DRA IL6 IL10 LSP1 NOS2 CAMP
Staphylococcus aureus infection	7	64	8.18E-05	FCGR2B FCGR3A HLA-DRA IL10 CFB C1R C5AR1
Rheumatoid arthritis	7	89	5.09E-04	CTSK HLA-DRA IL6 CXCL8 CCL2 CCL20 VEGFA
Small cell lung cancer	7	92	5.59E-04	CDK4 CKS2 COL4A1 COL4A2 FN1 LAMC1 NOS2
Chagas disease (American trypanosomiasis)	7	102	1.02E-03	ACE IL6 CXCL8 IL10 NOS2 SERPINE1 CCL2
Bladder cancer	6	41	7.67E-05	TYMP EGFR CXCL8 MMP2 MMP9 VEGFA
Prostate cancer	6	97	4.01E-03	EGFR MMP9 PLAT PLAU IL1R2 PDGFD
Malaria	5	48	1.25E-03	IL6 CXCL8 IL10 CCL2 SDC1

FDR: False Discovery Rate.

Table S10. List of cancer genes associated with glioblastoma patients' overall survival according to COSMIC database.

gene.symbol	somatic.tumour.types	germline.tumour.types	role.in.cancer
CDK4	----	melanoma	oncogene
CDKN2C	glioma, MM	----	TSG
COL1A1	DFSP, aneurysmal bone cyst	----	fusion
EGFR	glioma, NSCLC	NSCLC	oncogene
FCGR2B	ALL	----	oncogene, fusion
FKBP9	glioma	----	potential role
HOXC13	AML	----	oncogene, fusion
HOXD11	AML	----	oncogene, fusion
PAX3	alveolar rhabdomyosarcoma	----	oncogene, fusion
RUNX1	AML, pre B-ALL, T-ALL	----	oncogene, TSG, fusion

TSG: Tumor Suppressor Gene.

Table S11. Drug raw signature target genes list for several tumor types according to TTD, CIViC and TARGET databases.

	BRCA						ESCA						GBM						PAAD					
REBOOT	Genes			Transcripts			Genes			Transcripts			Genes			Transcripts			Genes			Transcripts		
Official Gene Symbol	*CIViC*	*TARGET*	*TTD*	*CIViC*	*TARGET*	*TTD*	*CIViC*	*TARGET*	*TTD*	*CIViC*	*TARGET*	*TTD*	*CIViC*	*TARGET*	*TTD*	*CIViC*	*TARGET*	*TTD*	*CIViC*	*TARGET*	*TTD*	*CIViC*	*TARGET*	*TTD*
ABCC3										X														
ACE															X									
ADM															X									
ADORA2B						X																		
ALAD												X												
ANG															X									
ANPEP															X									
ANXA2															X									
AQP9															X									
ATP1A2						X																		
BGN															X									
BUB1																					X			
C5AR1															X									
CACNA1B						X																		
CAMP															X									
CASP4															X									
CAV1															X						X			
CCL2															X									
CCL20															X									
CCL5			X																					
CCNA2																					X			
CCNB1															X						X			
CD163															X									
CD248															X									
CD52															X									
CDA																					X			
CDK1																					X			
CDK4													X	X	X									
CDKN2C															X									
CHEK1				X																				
CHRNB4						X																		
CLCF1															X									
CLIC1															X									
CNGA1						X																		
COL1A2															X									
CRHR1						X																		
CTSE						X																		
CTSK															X									
CXCL10																					X			
CXCL12																								X
CYP19A1						X																		
CYP46A1																					X			
DCN															X									
DIO1						X																		
DKK1													X		X									
DLL3																					X			
DMD																					X			
DTL																					X			
EGFR													X	X	X									
EPHX2																					X			
ERAP2																					X			X
ESRRG						X																		
F13A1															X									
F2RL2															X									
FABP5															X									
FAP															X									
FCGR2A																						X		X
FCGR2B													X		X									
FCGR3A													X		X									
FES																								X
FN1															X									
FOSL1															X									
GLRX2																		X						
GPBAR1																					X			
GPNMB															X									
HAMP															X									
HCN2																					X			
HLA-DRA													X											

HMGA2													X			
HMOX1					X		X									
HOXB13					X		X									
HP							X									
HTR2A			X													
HTT																X
IGFBP2							X									
IGFBP3							X									
IL10							X									
IL15RA				X												
IL1R2							X									
IL6					X		X									
ITGA2													X			
ITGA5							X									
KCNN4							X									
KLK8			X													
KYNU													X			
LDHA							X									
LGALS1							X									
LGALS3							X									
LIF							X									
LOX							X						X			
LTF							X									
LY6D			X													
LY75													X			
LY96							X									
LYZ							X									
MAP3K4				X												
MET											X	X	X			
MKI67											X		X			
MMP2					X											
MMP9					X											
MSLN			X													
MSR1							X									
MUTYH				X												
MYD88								X	X	X						
NAPRT														X		
NEK2													X			
NOS2							X									
NRG1		X														
NRP2													X			
OSMR							X									
PBK					X		X				X		X			
PDGFD							X									
PLAT							X									
PLAU							X									
PLAUR							X									
PLP2							X									
PNMT			X													
PORCN										X						
POSTN							X									
PSMB8											X					
PTK6													X			
PTTG1					X						X					
QPCT													X			
RB1														X	X	
RBP5																X
RRM2					X		X				X		X			
RUNX1					X	X	X									
S100A4							X									
S100A9							X									
SDC1							X									
SERPINA1							X									
SERPINE1							X									
SERPING1							X									
SERPINH1							X									
SFRP4	X															
SHCBP1													X			
SLC15A3				X												
SLC16A3							X									
SLC52A2	X															
SMN1				X												
SOCS3							X									

SPP1					X			
SPTBN1								X
ST14	X							
STC1					X			
TACR1		X						
TAGLN					X			
TAGLN2					X			
TFF3				X				
TFPI			X					
TIMP1				X				
TNFRSF12A					X			
TNIK			X					
TOP2A				X	X	X	X	
TREM1					X			
TWIST1					X			
TYMP					X			
TYMS				X	X			
VEGFA				X	X			
WISP1					X			
YAP1			X					

"X" means the Reboot official gene symbol is present in the corresponding analysis. For transcripts, Ensembl transcripts IDs were converted to equivalent Ensembl genes IDs. For the TTD database analysis, all 4 categories in the website were considered: (i) success; (ii) patent; (iii) clinical trials; and (iv) research.

Table S12. Transcripts associated with pancreas adenocarcinoma patients' overall survival in Reboot.

transcript.symbol	regression.coefficient
ADAP1-209	0.0243389
ADAP1-211	0.0089557
ADAP1-218	0.0085884
ALMS1-202	-0.0300002
BAIAP2L2-203	0.0140901
BBS1-206	-0.0359856
BCLAF1-204	0.0087351
BCLAF1-205	0.0184988
BID-209	0.0099699
BTN3A3-201	0.0125401
CARD6-201	0.0092825
CENPF-201	0.0819655
CENPF-206	0.0248675
CNOT1-204	-0.0044733
CTTN-210	-0.0000180
CXCL12-201	0.0004189
DNAJA4-203	0.0238143
ERAP2-202	0.0273279
EXOC7-201	-0.0129483
FAM111B-201	0.0329235
FCGR2A-201	0.0042429
FES-201	-0.0069084
FOPNL-201	0.0123006
GBGT1-203	-0.0008816
GNAI2-201	0.0004880
GNAI2-202	-0.0013797
GTF3C2-201	0.0156593
HTT-202	-0.0395587
IFI44-201	0.0060896
IFI44-203	0.0015845
LGALS8-203	0.0057068
LUC7L2-201	0.0077005
LUC7L2-202	-0.0014209
LUC7L2-203	-0.0212371
MALAT1-215	0.0000048
MAMDC4-204	-0.0135653
MCF2L-201	-0.1363477
MLKL-202	0.0371815
NAPRT-201	-0.0015641
NAPRT-203	-0.0019722
NOP53-201	-0.0027888
NRDC-201	0.0188089

NTM-202	-0.0001470
NTM-206	0.0058694
NUP54-201	0.0383425
PDE4DIP-214	0.0201281
PLAGL1-204	-0.0132881
PRSS3-202	0.0060979
RAB3GAP1-203	0.0141703
RB1-201	0.0078102
RBM23-204	-0.0001823
RBP5-201	-0.0221852
SLFN13-205	0.0235015
SNU13-202	0.0015843
SPTBN1-202	0.0052771
SPTBN1-204	-0.0015206
SRRM2-217	-0.0048007
TFDP1-207	-0.0379698
TPRA1-202	-0.0000431
TSC22D1-201	0.0012577
VCL-201	0.0138090
WRB-202	0.0095050

Highlighted are isoforms present in final PAAD 7-tr
